# Convenient Synthesis of *N*-Heterocycle-Fused Tetrahydro-1,4-diazepinones

**DOI:** 10.3390/molecules27248666

**Published:** 2022-12-07

**Authors:** Karolina Dzedulionytė, Melita Veikšaitė, Vít Morávek, Vida Malinauskienė, Greta Račkauskienė, Algirdas Šačkus, Asta Žukauskaitė, Eglė Arbačiauskienė

**Affiliations:** 1 Department of Organic Chemistry, Kaunas University of Technology, Radvilėnų pl. 19A, LT-50254 Kaunas, Lithuania; 2 Department of Chemical Biology, Palacký University, Šlechtitelů 27, CZ-78371 Olomouc, Czech Republic; 3 Institute of Synthetic Chemistry, Kaunas University of Technology, K. Baršausko g. 59, LT-51423 Kaunas, Lithuania

**Keywords:** pyrazole, indole, benzimidazole, fused *N*-heterocycles, regioselective *N*-alkylation, oxirane ring-opening, cyclisation

## Abstract

A general approach towards the synthesis of tetrahydro-4*H*-pyrazolo[1,5-*a*][1,4]diazepin-4-one, tetrahydro[1,4]diazepino[1,2-*a*]indol-1-one and tetrahydro-1*H*-benzo[4,5]imidazo[1,2-*a*][1,4]diazepin-1-one derivatives was introduced. A regioselective strategy was developed for synthesizing ethyl 1-(oxiran-2-ylmethyl)-1*H*-pyrazole-5-carboxylates from easily accessible 3(5)-aryl- or methyl-1*H*-pyrazole-5(3)-carboxylates. Obtained intermediates were further treated with amines resulting in oxirane ring-opening and direct cyclisation—yielding target pyrazolo[1,5-*a*][1,4]diazepin-4-ones. A straightforward two-step synthetic approach was applied to expand the current study and successfully functionalize ethyl 1*H*-indole- and ethyl 1*H*-benzo[*d*]imidazole-2-carboxylates. The structures of fused heterocyclic compounds were confirmed by ^1^H, ^13^C, and ^15^N-NMR spectroscopy and HRMS investigation.

## 1. Introduction

**Figure 1:**
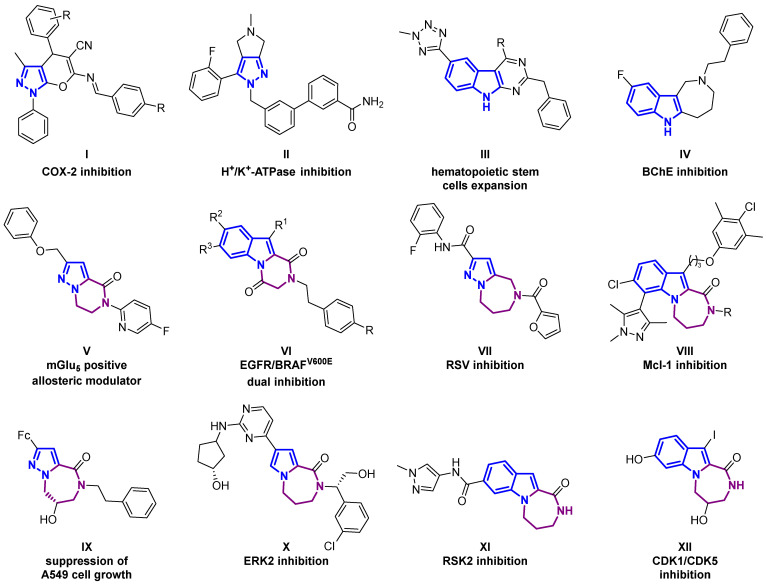
Biologically relevant fused heterocyclic derivatives.

Nitrogen-based heterocyclic compounds are integrated into everyday life, including pharmaceuticals [1,2,3], agrochemicals [4,5], various plastics [6,7], dyes [8,9], and other functional materials [10,11,12,13]. When it comes to drug discovery, the development of effective and inexpensive active ingredients is one of the main goals of researchers in the field of medicinal chemistry. Over the years, great attention has been paid to fused heterocyclic derivatives, which are versatile molecules, exhibiting a wide variety of biological properties including antioxidant, antimicrobial, antiproliferative and other activities [14,15,16,17]. For example, fused pyrano[2,3-*c*]pyrazole derivatives **I** were designed, synthesized and have been identified as prospective COX-2 inhibitors (Figure 1) [18]. A more recent study by Wang et al. encompasses a discovery of novel pyrrolo[3,4-*c*]pyrazol-3-carboxamides, where lead compound **II** exhibited potent inhibition towards H^+^/K^+^-ATPase and in vivo histamine-stimulated gastric acid secretion [19]. In the field of indole-based chemistry, Feng et al. reported 9*H*-pyrimido[4,5-*b*]indol-4-amines **III** as promising non-toxic hematopoietic stem cells ex vivo expansion agents [20]. In the study of Purgatorio and co-workers, azepino[4,3-*b*]indole **IV** was found to be a useful and versatile scaffold for developing new small molecules inhibiting BChE, which is a promising drug target in severe Alzheimer’s disease [21]. Fused systems with two 1,4-distanced nitrogen atoms encompass a variety of biological activities. For instance, Conde-Ceide et al. reported series of 6,7-dihydropyrazolo[1,5-a]pyrazin-4-one derivatives, such as **V**, as mGlu_5_ receptor-positive allosteric modulators with efficacy in preclinical models of schizophrenia [22], whereas 2,3-dihydropyrazino[1,2-*a*]indole-1,4-dione derivatives **VI** were reported to act as dual EGFR/BRAF^V600E^ inhibitors [23]. Among a variety of condensed systems, fusion of diazepine with selected heterocycles is important as it represents the most significant class of compounds in terms of clinical use [24]. Another notable example includes 5,6,7,8-tetrahydro-4*H*-pyrazolo[1,5-*a*][1,4]diazepine-2-carboxamide **VII** as a non-nucleoside inhibitor of the respiratory syncytial virus (RSV) polymerase complex [25] (Figure 1).

Combining the azaheterocycle scaffold into one structure with the diazepinone motif has been shown to result in biologically active compounds. However, to date examples of biologically active pyrazole-, pyrrole-, indole- and benzo[*d*]imidazole-fused diazepinone derivatives are limited. For example, tricyclic indole-diazepinones **VIII** inhibit induced myeloid leukaemia cell differentiation protein (Mcl-1) [26,27]. Chiral ferrocenylpyrazolo[1,5-*a*][1,4]diazepin-4-one **IX** was reported to suppress the growth of A549 lung cancer cells through cell cycle arrest, and H322 together with H1299 lung cancer cells by inducing apoptosis [28]. On the other hand, pyrrole or indole-fused diazepinones **X**–**XII** demonstrated inhibitory activity of various kinases, namely, extracellular signal-regulated kinase 2 (ERK2) [29], ribosomal S6 kinase (RSK) [30,31], cyclin-dependent kinase 1 (CDK1), and cyclin-dependent kinase 5 (CDK5) [32]. The abovementioned fused systems are not very widely investigated; therefore, it may be a promising entry in the field of synthetic and medicinal chemistry.

In our previous studies, we investigated the synthesis and biological activity of various annulated pyrazole systems such as substituted 2*H*-pyrazolo[4,3-*c*]pyridines [33,34,35], benzopyrano[2,3-*c*]pyrazol-4(2*H*)-ones [36], 2*H*-furo[2,3-*c*]pyrazole ring systems [37], and others. In continuation of our previous research on fused heterocycles, herein we report an efficient two-step synthesis of tetrahydro-4*H*-pyrazolo[1,5-*a*][1,4]diazepin-4-ones, tetrahydro[1,4]diazepino[1,2-*a*]indol-1-ones and tetrahydro-1*H*-benzo[4,5]imidazo[1,2-*a*][1,4]-diazepin-1-ones.

## 2. Results and Discussion

It is known that NH-pyrazoles usually exhibit annular *N*,*N*-prototropy [38,39]. *N*-Alkylation of asymmetrically ring-substituted 1*H*-pyrazoles generally results in the formation of a mixture of regioisomeric *N*-substituted products [40,41,42,43] and therefore regioselective *N*-alkylation requires optimization of reaction conditions [44]. Wright et al. has reported that alkylation of ethyl 1*H*-pyrazole-3(5)-carboxylate in the presence of K_2_CO_3_ was found to favor the formation of ethyl 1-substituted pyrazole-3-carboxylates while the formation of 1-substituted-1*H*-pyrazole-5-carboxylates could be sterically redirected by alkylating ethyl 3-(triphenylsilyl)-1*H*-pyrazole-5-carboxylate and removing the triphenylsilyl group with Bu_4_NF [45]. In another study implemented by Xu et al., regioselective *N*-alkylation of NH-pyrazoles was accessed by using MgBr_2_ and *i*-Pr_2_NEt or K_2_CO_3_ and leading to the formation of different regioisomers [46,47].

In this work, the formation of the desired 1-(oxiran-2-ylmethyl)-3-aryl-1*H*-pyrazole-5-carboxylates by alkylation of easily accessible NH-pyrazoles [48] with 2-(chloromethyl)oxirane was optimized using **1a** as a model compound. As shown in Table 1, reaction outcome is highly dependent on the choice of the base and the solvent. First, application of different bases in solvent-free conditions was investigated. When ethyl 3-phenyl-1*H*-pyrazole-5-carboxylate **1a** was treated with 2-(chloromethyl)oxirane in the presence of K_2_CO_3_ at 70 °C (Entry 1) [49], a full conversion of **1a** was only achieved after 16 h affording regioisomer **2a** in 24% yield. Reaction using Cs_2_CO_3_ as a base at lower temperature (Entry 2) [50] proceeded giving isomers **2a** and **3a** in 3:2 ratio. Interestingly, when reaction was conducted using NaH (Entry 3), it proved to proceed in high regioselectivity. However, as the major product the undesired 5-phenyl-1*H*-pyrazole-3-carboxylate **3a** was obtained. Reaction with KOH in the presence of a catalytic amount of TBAB [51] also did not provide an increase in the yield of desired regioisomer **2a** (Entry 4).

**Scheme 1:**
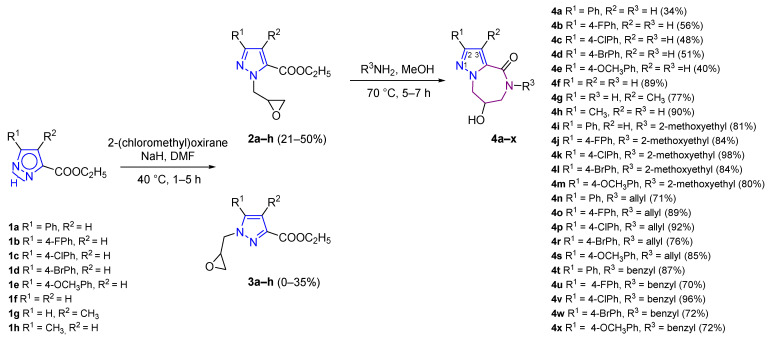
Synthesis of tetrahydro-4*H*-pyrazolo[1,5-*a*][1,4]diazepin-4-ones **4a–x** via *N*-alkylation and subsequent cyclisation reactions.

Subsequently, alkylation reaction conditions were investigated in the presence of solvent. To our satisfaction, both Cs_2_CO_3_ (Entry 5) [52] and NaH (Entry 6) in DMF [53] provided desired isomer **2a** as the major product in high regioselectivity. An attempt to perform reaction in refluxing ACN using Cs_2_CO_3_ as a base [54] gave similar results (Entry 7). Interestingly, we noticed that alkylation using NaH proved to be highly regiospecific as the ratio of obtained products was drastically shifted by changing the reaction media. The use of DMF as a solvent in the presence of NaH was selected as the most suitable approach to synthesize ethyl 1-(oxiran-2-ylmethyl)-3-phenyl-1*H*-pyrazole-5-carboxylates **2a–h** as it required overall reduced reaction time and temperature for full conversion of pyrazoles **1a–h** (Scheme 1). Although the reactions exhibit high regioselectivity, obtained yields did not exceed 53–61% due to instability of products **2a–e** during the purification process.

**Figure 2:**
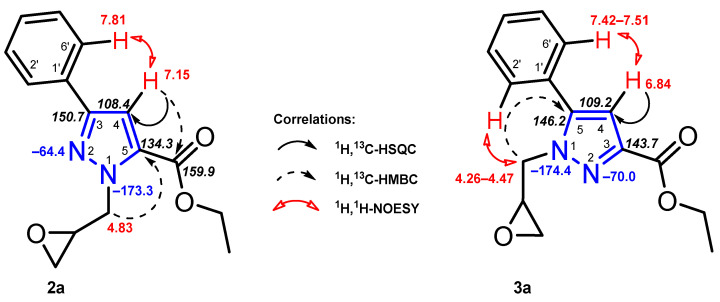
Relevant ^1^H-^13^C-HSQC, ^1^H,^13^C-HMBC, ^1^H,^1^H-NOESY correlations and ^1^H NMR (red), ^13^C NMR (italic), ^15^N NMR (blue) chemical shifts of regioisomers **2a** and **3a**.

Differentiation between major **2a** and minor **3a** isomers was implemented based on ^1^H,^13^C-HSQC, ^1^H,^1^H-NOESY, ^1^H,^13^C- and ^1^H,^15^N-HMBC experimental data. For example, ^1^H,^13^C-HMBC experiment of regioisomer **2a** revealed 3-bond correlation between NCH_2_ protons at δ 4.83 ppm and C-5 of pyrazole ring at δ 134.3 ppm (Figure 2). In the ^1^H,^15^N-HMBC spectrum, the same NCH_2_ protons exhibited 2-bond correlation with N-1 pyrrole-like (δ −173.3 ppm) and 3-bond correlation with N-2 pyridine-like (δ −64.4 ppm) nitrogen atoms. The ^1^H,^1^H-NOESY spectrum indicated close-in-space proton interaction between phenyl ring 2′(6′)-protons (δ 7.81 ppm) and pyrazole 4-H proton at δ 7.15 ppm.

The structure of minor regioisomer **3a** was elucidated in a similar manner. The ^1^H,^13^C-HMBC spectrum indicated that NCH_2_ protons correlate with pyrazole C-5 carbon at −146.2 ppm. Unambiguous distinction amongst both regioisomers can be determined using the ^1^H,^1^H-NOESY experiment. Close-in-space proton interaction was observed between phenyl ring 2′(6′)-H protons and pyrazole 4-H proton at δ 6.84 ppm, whereas regioisomer **3a** additionally exhibited NOEs between aromatic phenyl ring 2′(6′)-protons and NCH_2_ protons at δ 4.26–4.47 ppm.

According to the synthetic strategy, ethyl 1-(oxiran-2-ylmethyl)-3-aryl-1*H*-pyrazole-5-carboxylates **2a–h** were further used to obtain novel fused pyrazole-diazepinone systems via oxirane ring-opening. Due to high ring-strain, epoxides are prone to undergoing ring-opening reactions upon treatment with various nucleophiles [55] alone, or under the use of transition metal or organocatalysts [56,57,58]. On the other side, small heterocycles are also able to react with diverse electrophiles, affording a variety of functionalized molecules. Unlike the nucleophilic ring-opening reactions, the electrophilic ring-opening of small heterocycles cannot proceed by itself [59]. Methods reported in the literature for the epoxide ring-opening with amines are mainly focused on reactions mediated by a range of catalysts, activators, and promoters, in either solvent or solvent-free media [55,60,61,62,63]. In our study, intermediates **2a–h** were treated with various primary amines or ammonia in methanol (Scheme 1). Reaction monitoring indicated that ring closure proceeds rapidly to form a 1,4-diazepinone ring fused to pyrazole as no intermediate or side products have been observed, as opposed to the study reported by Shen et al. [28]. Reactions with primary amines, such as 2-methoxyethyl-, allyl- and benzylamines, afforded pyrazole-diazepinones **4i–x** in good to excellent yields (70–98%), while reactions with ammonia gave derivatives **4a–e** in substantially lower yields of 34–56%. Interestingly, tetrahydro-4*H*-pyrazolo[1,5-*a*][1,4]diazepin-4-ones **4g**,**h** bearing a methyl substituent in either 2- or 3-position as well as 2,3-unsubstituted pyrazole **4f** were obtained in higher yields (70–91%) compared to their phenyl-substituted counterparts **4a–e**.

A possible pyrazole-fused 1,4-diazepinone formation mechanism involves an amine-induced S_N_2 ring-opening at the less sterically hindered site of the oxirane ring [64]. Formed primary or secondary amine rapidly reacts with carbonyl carbon of an ester group, resulting in fused intermediate which further undergoes deprotonation and alkoxy group elimination to form final tetrahydro-4*H*-pyrazolo[1,5-*a*][1,4]diazepin-4-ones **4a–x**.

**Figure 3:**
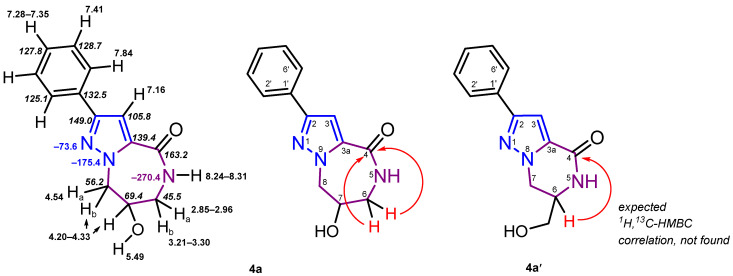
Determination of possible isomeric structures **4a** and **4a′**. Relevant ^1^H,^13^C-HMBC correlations and ^1^H NMR, ^13^C NMR (italic), ^15^N NMR (in blue) chemical shifts of compound **4a**.

To substantiate the structures of novel compounds, an unambiguous assignment of chemical shifts was carried out by investigating combined NMR spectroscopic data, i.e., ^1^H,^13^C-HSQC, ^1^H,^13^C-HMBC, ^1^H,^15^N-HMBC and ^1^H,^1^H-NOESY. In principle, cyclisation of ethyl 1-(oxiran-2-ylmethyl)-1*H*-pyrazole-5-carboxylate **2a** with ammonia could form two different isomers **4a** and **4a′** (Figure 3). The ^1^H,^13^C-HMBC experiment revealed a three-bond correlation between NH proton at δ 8.24–8.31 ppm and C-3a carbon at δ 139.4 ppm. Moreover, the same NH proton exhibited strong three-bond connectivity with tertiary C-7 carbon at 69.4 ppm. Interactions between C-4 carbon and 3-H at δ 7.16 ppm as well as 6-H_a_H_b_ protons were observed. Strong three-bond connectivity was observed in the ^1^H,^15^N-HMBC spectrum between 7-H proton and pyrrole-like nitrogen atom N-9 at δ −175.4 ppm. In case of putative isomer **4a′**, the heteronuclear multiple bond correlations between NH proton and secondary aliphatic carbons should be observed. Furthermore, C-4 carbon would not exhibit three-bond correlation with aliphatic protons connected to a secondary carbon atom.

A brief experiment was performed with minor regioisomer **3a** in order to investigate if cyclisation reaction is possible when carboxylate and oxirane fragments are distanced via an additional nitrogen atom. Ethyl 1*H*-pyrazole-3-carboxylate **3a** was treated with either ammonia or benzyl amine; however, analysis of LC/MS and NMR data indicated that both reactions resulted in the oxirane ring opening without subsequent cyclisation.

**Scheme 2:**
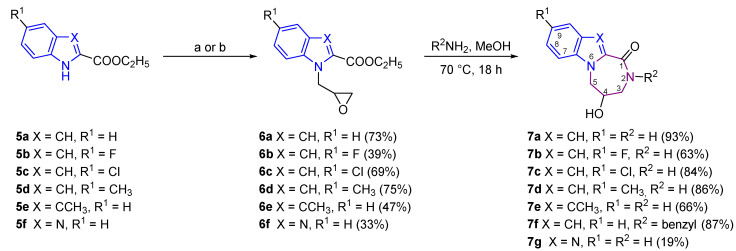
Synthesis of 4-hydroxy-2,3,4,5-tetrahydro-1*H*-[1,4]diazepino[1,2-*a*]indol-1-ones **7a–f** and 4-hydroxy-2,3,4,5-tetrahydro-1*H*-benzo[4,5]imidazo[1,2-*a*][1,4]diazepin-1-one **7g** via 2-step approach. *N*-Alkylation conditions: (a) 2-(chloromethyl)oxirane, KOH, DMF, 40 °C, 1 h (for **5a–e**); (b) 2-(chloromethyl)oxirane, NaH, DMF, 60 °C, 4 h (for **5f**).

Subsequently, we sought to investigate the reactivity of ethyl 1*H*-indole-2-carboxylate **5a–e** and benzo[*d*]imidazole-2-carboxylate **5f** scaffolds and derive target tetrahydro[1,4]diazepino[1,2-*a*]indol-1-ones and tetrahydro-1*H*-benzo[4,5]imidazo[1,2-*a*][1,4]diazepin-1-one utilizing the same straightforward 2-step approach (Scheme 2). *N*-Alkylation of **5a** with 2-(chloromethyl)oxirane using NaH in DMF at 40 °C provided ethyl 1-(oxiran-2-ylmethyl)-1*H*-indole-2-carboxylate **6a** in 54% yield. To increase the yield of target intermediate, several attempts to optimize reaction conditions were undertaken (Table 2). Unfortunately, neither elevation of reaction temperature, nor change of base or solvent have significant impact on the reaction outcome (Entries 2–5). Finally, the use of KOH base in DMF resulted not only in higher reactivity of starting 1*H*-indole-2-carboxylate **5a** (Entry 5) but also suppressed the formation of side products. Conditions utilizing the KOH-DMF system were applied to obtain ethyl 1-(oxiran-2-ylmethyl)-1*H*-indole-2-carboxylates **6a–e** in 39–75% yields (Scheme 2).

Interestingly, in the case benzo[*d*]imidazole-2-carboxylate **5f**, optimized conditions using KOH were not efficient as alkylation product was not obtained, suggesting lower reactivity of the NH group of this compound. Fortunately, *N*-alkylation of benzo[*d*]imidazole-2-carboxylate **5f** was accomplished under primary optimized conditions utilizing NaH-DMF at 60 °C, giving rise to target intermediate **6f** in 33% yield as other alkylation conditions did not provide a better result.

In the next step, applicability of direct ring opening-cyclisation reaction was investigated. Ethyl 1-(oxiran-2-ylmethyl)-1*H*-indole-2-carboxylates **6a–e** and ethyl 1-(oxiran-2-ylmethyl)-1*H*-benzo[*d*]imidazole-2-carboxylate (**6f**) were treated with either ammonia or benzylamine in methanol. Reactions proceeded in the same manner as using pyrazole counterparts and yielded 4-hydroxy-2,3,4,5-tetrahydro-1*H*-[1,4]diazepino[1,2-*a*]indol-1-ones and 4-hydroxy-2,3,4,5-tetrahydro-1*H*-benzo[4,5]imidazo[1,2-*a*][1,4]diazepin-1-one **7a–f** in fair to excellent (63–98%) yields, with an exception of **7g** which was isolated in a mere 19% yield. In comparison, Putey et al. [32] assessed synthesis of 2,3,4,5-tetrahydro[1,4]diazepino[1,2-*a*]indol-1-ones in a 4-step procedure.

**Scheme 3:**
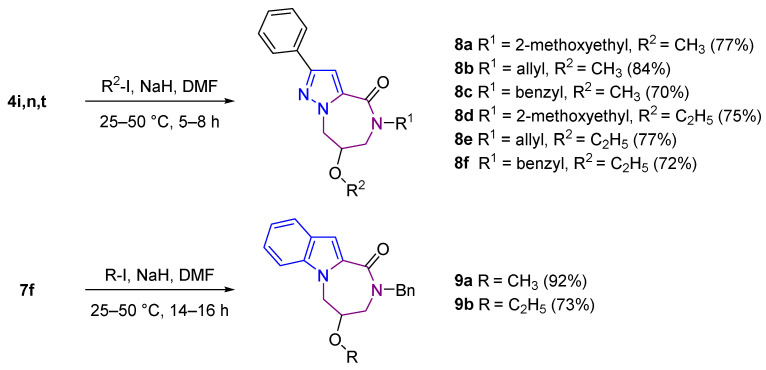
*O*-Alkylation of 5-substituted 7-hydroxy-2-phenyl-5,6,7,8-tetrahydro-4*H*-pyrazolo[1,5-*a*][1,4]diazepin-4-ones **4i**,**n**,**t** and 2-benzyl-4-hydroxy-2,3,4,5-tetrahydro-1*H*-[1,4]diazepino[1,2-*a*]indol-1-one **7f** using methyl and ethyl iodides.

To expand the structural diversity and compound library of fused tetrahydro-4*H*-pyrazolo[1,5-*a*][1,4]diazepin-4-ones and tetrahydro[1,4]diazepino[1,2-*a*]indol-1-ones, 5-substituted 7-hydroxy-2-phenyl-5,6,7,8-tetrahydro-4*H*-pyrazolo[1,5-*a*][1,4]diazepin-4-ones **4i**,**n**,**t** and 2-benzyl-4-hydroxy-2,3,4,5-tetrahydro-1*H*-[1,4]diazepino[1,2-*a*]indol-1-one **7f** were further modified using methyl- and ethyl iodides as *O*-alkylating agents (Scheme 3). Reactions were carried out involving NaH as a base in DMF, where reaction temperature and time were substituent dependent, i.e., compounds bearing benzyl group at 5-position required longer reaction times and higher temperatures. Compounds **8a–f** and **9a**,**b** were obtained in 70–92% yields.

## 3. Materials and Methods

### 3.1. General

The reagents and solvents were purchased from commercial suppliers and used without further purification unless otherwise indicated. Reaction progress was monitored by thin-layer chromatography (TLC) on pre-coated ALUGRAM^®^Xtra SIL G/UV_254_ plates (Macherey—Nagel™, Düren, Germany). The purification of the reaction mixtures was performed using flash chromatography on a glass column, stationary phase—silica gel (high-purity grade 9385, pore size 60 Å, particle size 230–400 mesh, Merck KGaA, Darmstadt, Germany). The ^1^H, ^13^C and ^15^N-NMR spectra were recorded in chloroform-D (CDCl_3_) or dimethyl sulfoxide-*d_6_* (DMSO-*d_6_*) at 25 °C on either Jeol ECA-500 (500 MHz—^1^H NMR, 126 MHz—^13^C NMR) or Jeol EC2 400R (400 MHz—^1^H NMR, 101 MHz—^13^C NMR) spectrometer equipped with a 5 mm Royal probe (JEOL USA, Inc., Peabody, MA, USA), or Bruker Avance III 400 spectrometer (400 MHz—^1^H NMR, 101 MHz—^13^C NMR, 40 MHz—^15^N NMR) using a 5 mm directly detecting BBO probe (Bruker BioSpin AG, Fallanden, Switzerland). Residual solvent signals were used as internal standards, i.e., for DMSO-*d_6_* δ*^1^_H_* = 2.50 and δ*^13^_C_* = 39.52, for CDCl_3_ δ*^1^_H_* = 7.26 and δ*^13^_C_* = 77.16. ^15^N chemical shifts were recalculated using a reference of neat external nitromethane standard (coaxial capillary). ^19^F NMR spectra (376 MHz) were obtained on a Bruker Avance III 400 instrument; here absolute referencing via δ ratio was used. The full and unambiguous assignments of the ^1^H, ^13^C, ^15^N-NMR resonances were achieved using a combination of standard NMR spectroscopic techniques. The following abbreviations are used in reporting NMR data: Ph, phenyl; Ox, oxirane. Melting points were determined using the apparatus DigiMelt MPA160 (Stanford Research Systems Inc., Sunnyvale, CA, USA) or Büchi B-540 (Büchi Labortechnik AG, Flawil, Switzerland) and are provided uncorrected. The IR spectra were recorded on a Bruker TENSOR 27 (Bruker Optik GmbH, Ettlingen, Germany) or Nicolet Impact 410 (SpectraLab Scientific Inc., Markham, ON, Canada) FTIR spectrometer using pressured KBr pellets. HRMS spectra were recorded on a micrOTOF-Q III Bruker (Bruker Daltonik GmbH, Bremen, Germany) or Agilent 6230 TOF LC/MS (Agilent Technologies Inc., Santa Clara, CA, United States) spectrometer in electrospray ionization (ESI) mode. ^1^H, ^13^C, ^19^F, and ^1^H,^15^N-HMBC NMR spectra, as well as HRMS data of new compounds, are provided in Figures S1–S229 of the Supplementary Materials.

### 3.2. Synthetic Procedures

#### 3.2.1. General Procedure for Synthesis of Starting 3(5)-Aryl-1*H*-pyrazole-5(3)-carboxylates (**1a–h**) [48,65]

To a 0.5 M solution of sodium ethoxide (1.1 eq) in ethanol, appropriate acetophenone (1 eq) and diethyl oxalate (1 eq) were added, and the resulting mixture was stirred at room temperature for 16 h in an inert atmosphere. Upon completion, the reaction mixture was quenched with 1 M HCl solution until neutral pH and extracted with ethyl acetate. Organic layer was washed with brine, dried over anhydrous Na_2_SO_4_, filtered, and concentrated under reduced pressure. The residue was purified by column chromatography on silica gel (*n*-hexane/ethyl acetate 15/1, *v*/*v*). Obtained ketoester (1 eq) was dissolved in a mixture of ethanol and acetic acid (7/3, *v*/*v*, 0.2 M), 55% aqueous hydrazine hydrate solution (1.1 eq) was added, and the reaction mixture was stirred at room temperature for 16 h. Subsequently, solvents were evaporated, and residue was dissolved in ethyl acetate and washed with 10% aqueous NaHCO_3_ solution. Organic layer was washed again with brine, dried over anhydrous Na_2_SO_4_, filtered, and concentrated under reduced pressure. The residue was purified by column chromatography on silica gel (*n*-hexane/ethyl acetate/methanol gradient from 8/1/0.1 to 1/1/0.1, *v*/*v*/*v*) to give corresponding pyrazoles **1a–h** in good yields (75–87%).

#### 3.2.2. Synthesis of Ethyl 1-(oxiran-2-ylmethyl)-1*H*-pyrazole-3(5)-carboxylates (**2a–h** and **3a**,**f–h**)

Appropriate pyrazole **1a–h** (1 eq) was dissolved in dry dimethyl formamide (0.4 M) and NaH (1.5 eq; 60% dispersion in mineral oil) was added followed by 2-(chloromethyl)oxirane (1.5 eq). The reaction mixture was stirred at 40 °C for 1–5 h. Upon completion, the reaction mixture was concentrated to approximately 1/3 volume, diluted with ethyl acetate, and washed with brine. Organic layer was separated, dried over anhydrous Na_2_SO_4_, filtered, and concentrated under reduced pressure. The residue was purified by column chromatography.

##### Ethyl 1-(oxiran-2-ylmethyl)-3-phenyl-1*H*-pyrazole-5-carboxylate **2a**

Purified by column chromatography on silica gel (*n*-hexane/ethyl acetate gradient from 10/1 to 8/1, *v*/*v*). White solid, mp 53–54 °C, 60% (3.13 g). R*_f_* = 0.72 (*n*-hexane/ethyl acetate 7/3, *v*/*v*). IR (KBr) ν_max_, cm^−1^: 2981, 1721 (C=O), 1261, 1086, 1073, 764, 758, 693, 436. ^1^H NMR (400 MHz, CDCl_3_) δ_H_ ppm: 1.41 (t, *J* = 7.1 Hz, 3H, CH_3_), 2.58–2.63 (m, 1H, Ox CH_a_H_b_), 2.81 (t, *J* = 4.4 Hz, 1H, CH_a_H_b_), 3.40–3.47 (m, Ox 1H, CH), 4.38 (q, *J* = 7.1 Hz, 2H, CH_2_CH_3_), 4.83 (qd, *J* = 14.2, 4.6 Hz, 2H, NCH_2_), 7.15 (s, 1H, 4-H), 7.29–7.37 (m, 1H, Ph 4-H), 7.38–7.44 (m, 2H, Ph 3,5-H), 7.78–7.84 (m, 2H, Ph 2,6-H). ^13^C NMR (101 MHz, CDCl_3_) δ_C_ ppm: 14.4 (CH_3_), 45.8 (Ox CH_2_), 50.7 (Ox CH), 53.0 (NCH_2_), 61.4 (CH_2_CH_3_), 108.4 (C-4), 125.8 (Ph C-2,6), 128.3 (Ph C-4), 128.8 (Ph C-3,5), 132.5 (Ph C-1), 134.3 (C-5), 150.7 (C-3), 159.9 (C=O). ^15^N NMR (40 MHz, CDCl_3_) δ_N_ ppm: −175.6 (N-1), −66.2 (N-2). HRMS (ESI) for C_15_H_16_N_2_NaO_3_ ([M+Na]^+^): calcd *m/z* 295.1053, found *m/z* 295.1053.

##### Ethyl 3-(4-fluorophenyl)-1-(oxiran-2-ylmethyl)-1*H*-pyrazole-5-carboxylate **2b**

Purified by column chromatography on silica gel (*n*-hexane/ethyl acetate 8/1, *v*/*v*). White solid, mp 98–99 °C, 59% (2.04 g). R*_f_* = 0.56 (*n*-hexane/ethyl acetate 7/3, *v*/*v*). IR (KBr) ν_max_, cm^−1^: 3140, 3061, 2979, 1730 (C=O), 1445, 1263, 1214, 1086, 849, 761. ^1^H NMR (400 MHz, DMSO-*d_6_*) δ_H_ ppm: 1.33 (t, *J* = 7.1 Hz, 3H, CH_3_), 2.48–2.51 (m, 1H, Ox CH_a_H_b_), 2.72 (t, *J* = 4.5 Hz, 1H, Ox CH_a_H_b_), 3.36–3.42 (m, 1H, Ox CH), 4.33 (q, *J* = 7.1 Hz, 2H, CH_2_CH_3_), 4.60 (dd, *J* = 14.5, 5.5 Hz, 1H, NCH_a_), 4.85 (dd, *J* = 14.5, 3.6 Hz, 1H, NCH_b_), 7.21–7.29 (m, 2H, Ph 3,5-H), 7.38 (s, 1H, 4-H), 7.86–7.95 (m, 2H, Ph 2,6-H). ^13^C NMR (101 MHz, DMSO-*d_6_*) δ_C_ ppm: 14.0 (CH_3_), 44.8 (Ox CH_2_), 50.2 (Ox CH), 52.8 (NCH_2_), 61.1 (CH_2_CH_3_), 108.0 (C-4), 115.65 (d, *^2^J_CF_* = 21.6 Hz, Ph C-3,5), 127.35 (d, *^3^J_CF_* = 8.3 Hz, Ph C-2,6), 128.54 (d, *^4^J_CF_* = 3.0 Hz, Ph C-1), 134.1 (C-5), 148.6 (C-3), 159.0 (C=O), 162.07 (d, *J_CF_* = 244.8 Hz, Ph C-4). ^15^N NMR (40 MHz, DMSO-*d_6_*) δ_N_ ppm: −173.3 (N-1), −65.1 (N-2). ^19^F NMR (376 MHz, DMSO-*d_6_*) δ_F_ ppm: −113.8 (Ph 6-F). HRMS (ESI) for C_15_H_15_FN_2_NaO_3_ ([M+Na]^+^): calcd *m/z* 313.0959, found *m/z* 313.0959.

##### Ethyl 3-(4-chlorophenyl)-1-(oxiran-2-ylmethyl)-1*H*-pyrazole-5-carboxylate **2c**

Purified by column chromatography on silica gel (*n*-hexane/ethyl acetate gradient from 8/1 to 7/1, *v*/*v*). Pale yellow solid, mp 110–111 °C, 53% (551 mg). R*_f_* = 0.57 (*n*-hexane/ethyl acetate 7/3, *v*/*v*). IR (KBr) ν_max_, cm^−1^: 3127, 2982, 2960, 1715 (C=O), 1460, 1266, 1123, 1088, 835, 766. ^1^H NMR (400 MHz, DMSO-*d_6_*) δ_H_ ppm: 1.34 (t, *J* = 7.1 Hz, 3H, CH_3_), 2.51–2.53 (m, 1H, Ox CH_a_CH_b_), 2.79 (t, *J* = 4.5 Hz, 1H, Ox CH_a_H_b_), 3.37–3.43 (m, 1H, Ox CH), 4.34 (q, *J* = 7.1 Hz, 2H, CH_2_CH_3_), 4.61 (dd, *J* = 14.5, 5.6 Hz, 1H, NCH_a_), 4.86 (dd, *J* = 14.4, 3.6 Hz, 1H, NCH_b_), 7.42 (s, 1H, 4-H), 7.45–7.51 (m, 2H, Ph 3,5-H), 7.86–7.93 (m, 2H, Ph 2,6-H). ^13^C NMR (101 MHz, DMSO-*d_6_*) δ_C_ ppm: 14.0 (CH_3_), 44.8 (Ox CH_2_), 50.1 (Ox CH), 52.9 (NCH_2_), 61.1 (CH_2_CH_3_), 108.3 (C-4), 127.0 (Ph C-2,6), 128.8 (Ph C-3,5), 130.8 (Ph C-1), 132.7 (Ph C-4), 134.2 (C-5), 148.3 (C-3), 159.0 (C=O). ^15^N NMR (40 MHz, DMSO-*d_6_*) δ_N_ ppm: −172.3 (N-1), −65.2 (N-2). HRMS (ESI) for C_15_H_15_ClN_2_NaO_3_ ([M+Na]^+^): calcd *m/z* 329.0663, found *m/z* 329.0663.

##### Ethyl 3-(4-bromophenyl)-1-(oxiran-2-ylmethyl)-1*H*-pyrazole-5-carboxylate **2d**

Purified by column chromatography on silica gel (*n*-hexane/ethyl acetate gradient from 10/1 to 8/1, *v*/*v*). White solid, mp 120–121 °C, 58% (1.88 g). R*_f_* = 0.60 (*n*-hexane/ethyl acetate 7/3, *v*/*v*). IR (KBr) ν_max_, cm^−1^: 3127, 2979, 1716 (C=O), 1459, 1436, 1267, 1123, 1092, 832, 766. ^1^H NMR (400 MHz, DMSO-*d_6_*) δ_H_ ppm: 1.33 (t, *J* = 7.1 Hz, 3H, CH_3_), 2.48–2.52 (m, 1H, Ox CH_a_H_b_), 2.78 (t, *J* = 4.5 Hz, 1H, Ox CH_a_H_b_), 3.37–3.42 (m, 1H, Ox CH), 4.33 (q, *J* = 7.1 Hz, 2H, CH_2_CH_3_), 4.60 (dd, *J* = 14.5, 5.6 Hz, 1H, NCH_a_), 4.86 (dd, *J* = 14.5, 3.6 Hz, 1H, NCH_b_), 7.43 (s, 1H, 4-H), 7.58–7.64 (m, 2H, Ph 3,5-H), 7.79–7.86 (m, 2H, Ph 2,6-H). ^13^C NMR (101 MHz, DMSO-*d_6_*) δ_C_ ppm: 14.0 (CH_3_), 44.8 (Ox CH_2_), 50.1 (Ox CH), 52.9 (NCH_2_), 61.1 (CH_2_CH_3_), 108.3 (C-4), 121.3 (Ph C-4), 127.3 (Ph C-2,6), 131.2 (Ph C-3,5), 131.7 (Ph C-1), 134.2 (C-5), 148.4 (C-3), 159.0 (C=O). ^15^N NMR (40 MHz, DMSO-*d_6_*) δ_N_ ppm: −172.3 (N-1), −64.8 (N-2). HRMS (ESI) for C_15_H_15_BrN_2_NaO_3_ ([M+Na]^+^): calcd *m/z* 373.0158, found *m/z* 373.0158 and 375.0136.

##### Ethyl 3-(4-methoxyphenyl)-1-(oxiran-2-ylmethyl)-1*H*-pyrazole-5-carboxylate **2e**

Purified by column chromatography on silica gel (*n*-hexane/ethyl acetate 8/1, *v*/*v*). White solid, mp 75–76 °C, 61% (1.9 g). R*_f_* = 0.49 (*n*-hexane/ethyl acetate 7/2 *v*/*v*). IR (KBr) ν_max_, cm^−1^: 3139, 2975, 2938, 2836, 1726 (C=O), 1447, 1086, 1029, 846, 758. ^1^H NMR (400 MHz, DMSO-*d_6_*) δ_H_ ppm: 1.33 (t, *J* = 7.1 Hz, 3H, CH_3_), 2.47–2.50 (m, 1H, Ox CH_a_CH_b_), 2.78 (t, *J* = 4.5 Hz, 1H, Ox CH_a_H_b_), 3.35–3.42 (m, 1H, Ox CH), 3.79 (s, 3H, OCH_3_), 4.33 (q, *J* = 7.1 Hz, 2H, CH_2_CH_3_), 4.59 (dd, *J* = 14.5, 5.5 Hz, 1H, NCH_a_), 4.84 (dd, *J* = 14.4, 3.5 Hz, 1H, NCH_b_), 6.93–7.02 (m, 2H, Ph 3,5-H), 7.30 (s, 1H, 4-H), 7.75–7.83 (m, 2H, Ph 2,6-H). ^13^C NMR (101 MHz, DMSO-*d_6_*) δ_C_ ppm: 14.1 (CH_3_), 44.8 (Ox CH_2_), 50.2 (Ox CH), 52.7 (NCH_2_), 55.2 (OCH_3_), 61.0 (CH_2_CH_3_), 107.5 (C-4), 114.2 (Ph C-3,5), 124.6 (Ph C-1), 126.6 (Ph C-2,6), 133.9 (C-5), 149.4 (C-3), 159.1 (C=O), 159.3 (Ph C-4). ^15^N NMR (40 MHz, DMSO-*d_6_*) δ_N_ ppm: −174.5 (N-1) −67.0 (N-2). HRMS (ESI) for C_16_H_18_N_2_NaO_4_ ([M+Na]^+^): calcd *m/z* 325.1159, found *m/z* 325.1159.

##### Ethyl 1-(oxiran-2-ylmethyl)-1*H*-pyrazole-5-carboxylate **2f**

Purified by column chromatography on silica gel (*n*-hexane/ethyl acetate 4/1, *v*/*v*). Colorless liquid, 21% (30 mg). R*_f_* = 0.57 (*n*-hexane/ethyl acetate 2/1, *v*/*v*). IR (KBr) ν_max_, cm^−1^: 2985, 1722 (C=O), 1518, 1312, 1254, 1121, 1105, 1039, 765. ^1^H NMR (400 MHz, CDCl_3_) δ_H_ ppm: 1.39 (t, *J* = 7.1 Hz, 3H, CH_3_), 2.54–2.56 (m, 1H, Ox CH_a_H_b_), 2.80 (t, *J* = 4.4 Hz, 1H, Ox CH_a_H_b_), 3.37–3.40 (m, 1H, Ox CH), 4.36 (q, *J* = 7.1 Hz, 2H, CH_2_CH_3_), 4,73 (dd, *J* = 14.2, 5.2 Hz, 1H, NCH_a_H_b_), 4.86 (dd, *J* = 14.2, 4.2 Hz, 1H, NCH_a_H_b_), 6.86 (d, *J* = 1.5 Hz, 1H, 4-H), 7.53 (d, *J* = 1.4 Hz, 1H, 3-H). ^13^C NMR (101 MHz, CDCl_3_) δ_C_ ppm: 14.3 (CH_3_), 45.8 (Ox CH_2_), 50.7 (Ox CH), 53.0 (NCH_2_), 61.3 (CH_2_CH_3_), 111.6 (C-4), 133.0 (C-5), 138.8 (C-3), 159.9 (COO). ^15^N NMR (40 MHz, CDCl_3_) δ_N_ ppm: –173.5 (N-1), –60.2 (N-2). HRMS (ESI) for C_9_H_13_N_2_O_3_ ([M+H]^+^): calcd *m/z* 197.0921, found *m/z* 197.0916.

##### Ethyl 4-methyl-1-(oxiran-2-ylmethyl)-1*H*-pyrazole-5-carboxylate **2g**

Purified by column chromatography on silica gel (*n*-hexane/ethyl acetate 4/1, *v*/*v*). Colorless liquid, 26% (36 mg). R*_f_* = 0.47 (*n*-hexane/ethyl acetate 2/1, *v*/*v*). IR (KBr) ν_max_, cm^−1^: 2983, 1716 (C=O), 1449, 1277, 1113, 1042. ^1^H NMR (400 MHz, CDCl_3_) δ_H_ ppm: 1.40 (t, *J* = 7.1 Hz, 3H, CH_2_CH_3_), 2.26 (s, 3H, 4-CH_3_), 2.52–2.54 (m, 1H, Ox CH_a_H_b_), 2.78 (t, *J* = 4.4 Hz, 1H, Ox CH_a_H_b_), 3.34–3.37 (m, 1H, Ox CH), 4.38 (q, *J* = 7.1 Hz, 2H, CH_2_CH_3_), 4.68 (dd, *J* = 14.3, 5.1 Hz, 1H, NCH_a_H_b_), 4.80 (dd, *J* = 14.3, 4.1 Hz, 1H, NCH_a_H_b_), 7.36 (s, 1H, 3-H). ^13^C NMR (101 MHz, CDCl_3_) δ_C_ ppm: 10.9 (4-CH_3_), 14.3 (CH_2_CH_3_), 45.7 (Ox CH_2_), 50.8 (Ox CH), 53.5 (NCH_2_), 61.0 (CH_2_CH_3_), 123.1 (C-4), 130.1 (C-5), 140.2 (C-3), 160.7 (COO). ^15^N NMR (40 MHz, CDCl_3_) δ_N_ ppm: –174.6 (N-1), –63.9 (N-2). HRMS (ESI) for C_10_H_15_N_2_O_3_ ([M+H]^+^): calcd *m/z* 211.1077, found *m/z* 211.1067.

##### Ethyl 3-methyl-1-(oxiran-2-ylmethyl)-1*H*-pyrazole-5-carboxylate **2h**

Purified by column chromatography on silica gel (*n*-hexane/ethyl acetate 4/1, *v*/*v*). Colorless liquid, 24% (32 mg). R*_f_* = 0.42 (*n*-hexane/ethyl acetate 2/1, *v*/*v*). IR (KBr) ν_max_, cm^−1^: 2962, 2909, 1722 (C=O), 1461, 1262, 1085, 767. ^1^H NMR (400 MHz, CDCl_3_) δ_H_ ppm: 1.37 (t, *J* = 7.1 Hz, 3H, CH_2_CH_3_), 2.28 (s, 3H, 3-CH_3_), 2.55–2.57 (m, 1H, Ox CH_a_H_b_), 2.79 (t, *J* = 4.4 Hz, 1H, Ox CH_a_H_b_), 3.34–3.38 (m, 1H, Ox CH), 4.33 (q, *J* = 7.1 Hz, 2H, CH_2_CH_3_), 4.63 (dd, *J* = 14.3, 5.2 Hz, 1H, NCH_a_H_b_), 4.78 (dd, *J* = 14.3, 4.1 Hz, 1H, NCH_a_H_b_), 6.64 (s, 1H, 4-H). ^13^C NMR (101 MHz, CDCl_3_) δ_C_ ppm: 13.5 (3-CH_3_), 14.4 (CH_2_CH_3_), 45.8 (Ox CH_2_), 50.8 (Ox CH), 52.7 (NCH_2_), 61.2 (CH_2_CH_3_), 111.0 (C-4), 133.4 (C-5), 148.0 (C-3), 160.0 (COO). ^15^N NMR (40 MHz, CDCl_3_) δ_N_ ppm: –178.6 (N-1), –64.1 (N-2). HRMS (ESI) for C_10_H_15_N_2_O_3_ ([M+H]^+^): calcd *m/z* 211.1077, found *m/z* 211.1075.

##### Ethyl 1-(oxiran-2-ylmethyl)-5-phenyl-1*H*-pyrazole-3-carboxylate **3a**

Purified by column chromatography on silica gel (*n*-hexane/ethyl acetate gradient from 8/1 to 1/1, *v*/*v*). Colorless resin, 56% (20 mg). R*_f_* = 0.37 (*n*-hexane/ethyl acetate 1/1, *v*/*v*). IR (KBr) ν_max_, cm^−1^: 2986, 1729 (C=O), 1214, 765, 701. ^1^H NMR (400 MHz, CDCl_3_) δ_H_ ppm: 1.41 (t, *J* = 7.1 Hz, 3H, CH_3_), 2.49–2.53 (m, 1H, Ox CH_a_H_b_), 2.81 (t, *J* = 4.3 Hz, 1H, Ox CH_a_H_b_), 3.40–3.45 (m, 1H, Ox CH), 4.26–4.47 (m, 4H, CH_2_CH_3_, NCH_2_), 6.84 (s, 1H, 4-H), 7.42–7.51 (m, 5H, Ph 2,3,4,5,6-H). ^13^C NMR (101 MHz, CDCl_3_) δ_C_ ppm: 14.6 (CH_3_), 46.0 (Ox CH_2_), 50.7 (Ox CH), 51.6 (NCH_2_), 61.2 (CH_2_CH_3_), 109.2 (C-4), 129.0 (Ph C-1,2,6), 129.4 (Ph C-4), 129.5 (Ph C-3,5), 143.7 (C-3), 146.2 (C-5), 162.5 (C=O). ^15^N NMR (40 MHz, CDCl_3_) δ_N_ ppm: −174.4 (N-1), −70.0 (N-2). HRMS (ESI) for C_15_H_16_N_2_NaO_3_ ([M+Na]^+^): calcd *m/z* 295.1053, found *m/z* 295.1051.

##### Ethyl 1-(oxiran-2-ylmethyl)-1*H*-pyrazole-3-carboxylate **3f**

Purified by column chromatography on silica gel (*n*-hexane/ethyl acetate gradient from 4/1 to 2/1, *v*/*v*). Colorless liquid, 28% (39 mg). R*_f_* = 0.49 (*n*-hexane/ethyl acetate 1/2, *v*/*v*). IR (KBr) ν_max_, cm^−1^: 2986, 1721 (C=O), 1375, 1237, 1173, 1154, 1027, 765. ^1^H NMR (400 MHz, CDCl_3_) δ_H_ ppm: 1.40 (t, *J* = 7.1, 3H, CH_3_), 2.49–2.51 (m, 1H, Ox CH_a_H_b_), 2.87 (t, *J* = 4.2 Hz, 1H, Ox CH_a_H_b_), 3.36–3.38 (m, 1H, Ox CH), 4.21 (dd, *J* = 14.7, 6.0 Hz, 1H, NCH_a_H_b_), 4.41 (q, *J* = 7.1 Hz, 2H, CH_2_CH_3_), 4.62 (dd, *J* = 14.7, 2.6 Hz, 1H, NCH_a_H_b_), 6.84 (d, *J* = 2.1 Hz, 1H, 4-H), 7.53 (d, *J* = 2.1 Hz, 1H, 5-H). ^13^C NMR (101 MHz, CDCl_3_) δ_C_ ppm: 14.5 (CH_3_), 45.5 (Ox CH_2_), 50.4 (Ox CH), 54.6 (NCH_2_), 61.2 (CH_2_CH_3_), 109.5 (C-4), 131.7 (C-5), 144.2 (C-3), 162.4 (COO). ^15^N NMR (40 MHz, CDCl_3_) δ_N_ ppm: –171.8 (N-1), –68.9 (N-2). HRMS (ESI) for C_9_H_13_N_2_O_3_ ([M+H]^+^): calcd *m/z* 197.0921, found *m/z* 197.0915.

##### Ethyl 4-methyl-1-(oxiran-2-ylmethyl)-1*H*-pyrazole-3-carboxylate **3g**

Purified by column chromatography on silica gel (*n*-hexane/ethyl acetate gradient from 4/1 to 2/1, *v*/*v*). Colorless liquid, 35% (48 mg). R*_f_* = 0.47 (*n*-hexane/ethyl acetate 1/2, *v*/*v*). IR (KBr) ν_max_, cm^−1^: 2983, 2932, 1716 (C=O), 1448, 1367, 1254, 1108. ^1^H NMR (400 MHz, CDCl_3_) δ_H_ ppm: 1.41 (t, *J* = 7.1 Hz, 3H, CH_2_CH_3_), 2.29 (s, 3H, 4-CH_3_), 2.49–2.51 (m, 1H, Ox CH_a_H_b_), 2.86 (t, *J* = 4.2 Hz, 1H, Ox CH_a_H_b_), 3.33–3.35 (m, 1H, Ox CH), 4.13 (dd, *J* = 14.7, 6.0 Hz, 1H, NCH_a_H_b_), 4.41 (q, *J* = 7.1 Hz, 2H, CH_2_CH_3_), 4.55 (dd, *J* = 14.7, 2.5 Hz, 1H, NCH_a_H_b_), 7.33 (s, 1H, 5-H). ^13^C NMR (101 MHz, CDCl_3_) δ_C_ ppm: 9.9 (4-CH_3_), 14.5 (CH_2_CH_3_), 45.4 (Ox CH_2_), 50.5 (Ox CH), 54.5 (NCH_2_), 60.8 (CH_2_CH_3_), 121.5 (C-4), 131.0 (C-5), 141.4 (C-3), 163.0 (COO). ^15^N NMR (40 MHz, CDCl_3_) δ_N_ ppm: –175.7 (N-1), –69.2 (N-2). HRMS (ESI) for C_10_H_15_N_2_O_3_ ([M+H]^+^): calcd *m/z* 211.1077, found *m/z* 211.1072.

##### Ethyl 5-methyl-1-(oxiran-2-ylmethyl)-1*H*-pyrazole-3-carboxylate **3h**

Purified by column chromatography on silica gel (*n*-hexane/ethyl acetate gradient from 4/1 to 2/1, *v*/*v*). White solid, mp 31–33 °C, 29% (40 mg). R*_f_* = 0.47 (*n*-hexane/ethyl acetate 1/2, *v*/*v*). IR (KBr) ν_max_, cm^−1^: 2985, 1721 (C=O), 1446, 1386, 1228, 1032, 780. ^1^H NMR (400 MHz, CDCl_3_) δ_H_ ppm: 1.39 (t, *J* = 7.1 Hz, 3H, CH_2_CH_3_), 2.34 (s, 3H, 5-CH_3_), 2.47–2.49 (m, 1H, Ox CH_a_H_b_), 2.82 (t, *J* = 4.3 Hz, 1H, Ox CH_a_H_b_), 3.33–3.37 (m, 1H, Ox CH), 4.21 (dd, *J* = 15.0, 5.2 Hz, 1H, NCH_a_H_b_), 4.39 (q, *J* = 7.1 Hz, 2H, CH_2_CH_3_) 4.53 (dd, *J* = 15.0, 2.4 Hz, 1H, NCH_a_H_b_), 6.57 (s, 1H, 4-H). ^13^C NMR (101 MHz, CDCl_3_) δ_C_ ppm: 11.4 (5-CH_3_), 14.5 (CH_2_CH_3_), 45.3 (Ox CH_2_), 50.9 (Ox CH), 51.4 (NCH_2_), 61.0 (CH_2_CH_3_), 108.6 (C-4), 141.3 (C-5), 143.1 (C-3), 162.6 (COO). ^15^N NMR (40 MHz, CDCl_3_) δ_N_ ppm: –173.3 (N-1), –70.8 (N-2). HRMS (ESI) for C_10_H_15_N_2_O_3_ ([M+H]^+^): calcd *m/z* 211.1077, found *m/z* 211.1070.

#### 3.2.3. Synthesis of 7-Hydroxy-5,6,7,8-tetrahydro-4*H*-pyrazolo[1,5-*a*][1,4]diazepin-4-ones (**4a–x**)

**Procedure a**. Carboxylate **2a–h** (1 eq) was dissolved in 2M ammonia (30 eq) solution in methanol, sealed in a pressure tube and stirred at 70 °C for 5–18 h. After completion, excess of ammonia in methanol was evaporated under reduced pressure. The residue was purified by column chromatography.

**Procedure b**. To a solution of carboxylate **2a–e** (1 eq) in methanol (5 M), appropriate primary amine (3 eq) was added, the reaction mixture was sealed in a pressure tube and was stirred at 70 °C for 7 h. After completion, the reaction mixture was poured into water and extracted with ethyl acetate. Combined organic layers were washed with brine, dried over anhydrous Na_2_SO_4_, filtered, and concentrated under reduced pressure. The residue was purified by column chromatography.

##### 7-Hydroxy-2-phenyl-5,6,7,8-tetrahydro-4*H*-pyrazolo[1,5-*a*][1,4]diazepin-4-one **4a**

Purified by column chromatography on silica gel (gradient from *n*-hexane/ethyl acetate/methanol gradient from 1/9/0 to 1/15/0.5, *v*/*v*). White solid, decomp. 246 °C, procedure a −34% (27 mg). R*_f_* = 0.40 (dichloromethane/methanol 100/5, *v*/*v*). IR (KBr) ν_max_, cm^−1^: 3270, 3196, 3074, 2925, 1681 (C=O), 1460, 1440, 771, 752, 685. ^1^H NMR (400 MHz, DMSO-*d_6_*) δ_H_ ppm: 2.85–2.96 (m, 1H, 6-H_a_), 3.21–3.30 (m, 1H, 6-H_b_), 4.20–4.33 (m, 2H, 7-H, 8-H_a_), 4.54 (dd, *J* = 13.8, 4.8 Hz, 1H, 8-H_b_), 5.49 (d, *J* = 3.8 Hz, 1H, OH), 7.16 (s, 1H, 3-H), 7.28–7.35 (m, 1H, Ph 4-H), 7.36–7.45 (m, 2H, Ph 3,5-H), 7.80–7.88 (m, 2H, Ph 2,6-H), 8.24–8.31 (m, 1H, NH). ^13^C NMR (101 MHz, DMSO-*d_6_*) δ_C_ ppm: 45.5 (C-6), 56.2 (C-8), 69.4 (C-7), 105.8 (C-3), 125.1 (Ph C-2,6), 127.8 (Ph C-4), 128.7 (Ph C-3,5), 132.5 (Ph C-1), 139.4 (C-3a), 149.0 (C-2), 163.2 (C-4). ^15^N NMR (40 MHz, DMSO-*d_6_*) δ_N_ ppm: −270.4 (N-5), −175.4 (N-9), −73.6 (N-1). HRMS (ESI) for C_13_H_13_N_3_NaO_2_ ([M+Na]^+^): calcd *m/z* 266.0900, found *m/z* 266.0900.

##### 2-(4-Fluorophenyl)-7-hydroxy-5,6,7,8-tetrahydro-4*H*-pyrazolo[1,5-*a*][1,4]diazepin-4-one **4b**

Purified by column chromatography on silica gel (*n*-hexane/ethyl acetate/methanol gradient from 1/9/0 to 1/15/0.5, *v*/*v*). Pale yellow solid, decomp. 216 °C, procedure a −56% (86 mg). R*_f_* = 0.38 (dichloromethane/methanol 100/5, *v*/*v*). IR (KBr) ν_max_, cm^−1^: 3305, 3206, 3071, 2947, 2923, 1681, 1457, 1442, 841, 809. ^1^H NMR (400 MHz, DMSO-*d_6_*) δ_H_ ppm: 3.74 (dt, *J* = 12.2, 5.8 Hz, 1H, 6-H_a_), 4.09 (dt, *J* = 14.5, 5.4 Hz, 1H, 6-H_b_), 5.02–5.15 (m, 2H, 7-H, 8-H_a_), 5.36 (dd, *J* = 13.9, 5.0 Hz, 1H, 8-H_b_), 8.00 (s, 1H, 3-H), 8.04–8.11 (m, 2H, Ph 3,5-H), 8.67–8.75 (m, 2H, Ph 2,6-H), 9.12–9.18 (m, 1H, NH). ^13^C NMR (101 MHz, DMSO-*d_6_*) δ_C_ ppm: 45.5 (C-6), 56.2 (C-8), 69.3 (C-7), 105.8 (C-3), 115.59 (d, *^2^J_CF_* = 21.5 Hz, Ph C-3,5), 127.12 (d, *^3^J_CF_* = 8.2 Hz, Ph C-2,6), 129.10 (d, *^4^J_CF_* = 3.0 Hz, Ph C-1), 139.6 (C-3a), 148.2 (C-2), 161.83 (d, *J_CF_* = 244.3 Hz, Ph C-4), 163.1 (C-4). ^15^N NMR (40 MHz, DMSO-*d_6_*) δ_N_ ppm: −270.5 (N-5), −175.3 (N-9), −73.8 (N-1). ^19^F NMR (376 MHz, DMSO-*d_6_*) δ_F_ ppm: −114.4 (Ph 6-F). HRMS (ESI) for C_13_H_12_FN_3_NaO_2_ ([M+Na]^+^): calcd *m/z* 284.0806, found *m/z* 284.0806.

##### 2-(4-Chlorophenyl)-7-hydroxy-5,6,7,8-tetrahydro-4*H*-pyrazolo[1,5-*a*][1,4]diazepin-4-one **4c**

Purified by column chromatography on silica gel (*n*-hexane/ethyl acetate/methanol gradient from 1/9/0 to 1/20/0.5, *v*/*v*). White solid, decomp. 249 °C, procedure a −48% (59 mg). R*_f_* = 0.38 (dichloromethane/methanol 100/5, *v*/*v*). IR (KBr) ν_max_, cm^−1^: 3297, 3210, 3080, 2917, 1683 (C=O), 1451, 1435, 1089, 834, 809. ^1^H NMR (400 MHz, DMSO-*d_6_*) δ_H_ ppm: 2.86–2.95 (m, 1H, 6-H_a_), 3.21–3.30 (m, 1H, 6-H_b_), 4.19–4.31 (m, 2H, 7-H, 8-H_a_), 4.54 (dd, *J* = 13.9, 4.9 Hz, 1H, 8-H_b_), 5.53 (br s, 1H, OH), 7.20 (s, 1H, 3-H), 7.43–7.50 (m, 2H, Ph 3,5-H), 7.82–7.91 (m, 2H, Ph 2,6-H), 8.28–8.33 (m, 1H, NH). ^13^C NMR (101 MHz, DMSO-*d_6_*) δ_C_ ppm: 45.5 (C-6), 56.3 (C-8), 69.3 (C-7), 106.0 (C-3), 126.8 (Ph C-2,6), 128.8 (Ph C-3,5), 131.4 (Ph C-1), 132.3 (Ph C-4), 139.6 (C-3a), 147.9 (C-2), 163.0 (C-4). ^15^N NMR (40 MHz, DMSO-*d_6_*) δ_N_ ppm: −270.4 (N-5), −174.0 (N-9), −69.7 (N-1). HRMS (ESI) for C_13_H_12_ClN_3_NaO_2_ ([M+Na]^+^): calcd *m/z* 300.0510, found *m/z* 300.0510.

##### 2-(4-Bromophenyl)-7-hydroxy-5,6,7,8-tetrahydro-4*H*-pyrazolo[1,5-*a*][1,4]diazepin-4-one **4d**

Purified by column chromatography on silica gel (*n*-hexane/ethyl acetate/methanol gradient from 1/9/0 to 1/12/0.5, *v*/*v*). White solid, decomp. 230 °C, procedure a −51% (50 mg). R*_f_* = 0.40 (dichloromethane/methanol 100/5, *v*/*v*). IR (KBr) ν_max_, cm^−1^: 3292, 3214, 3079, 1680 (C=O), 1455 and 1434 (doublet), 1071, 991, 922, 816 and 807 (doublet). ^1^H NMR (400 MHz, DMSO-*d_6_*) δ_H_ ppm: 2.90 (dt, *J* = 12.2, 5.8 Hz, 1H, 6-H_a_), 3.26 (dt, *J* = 14.4, 5.3 Hz, 1H, 6-H_b_), 4.19–4.33 (m, 2H, 7-H, 8-H_a_), 4.54 (dd, *J* = 13.9, 4.9 Hz, 1H, 8-H_b_), 5.49 (d, *J* = 41 Hz, 1H, OH), 7.20 (s, 1H, 3-H), 7.57–7.64 (m, 2H, Ph 3,5-H), 7.76–7.84 (m, 2H, Ph 2,6-H), 8.29 (t, *J* = 5.1 Hz, 1H, NH). ^13^C NMR (101 MHz, DMSO-*d_6_*) δ_C_ ppm: 45.5 (C-6), 56.3 (C-8), 69.3 (C-7), 106.0 (C-3), 120.8 (Ph C-4), 127.1 (Ph C-2,6), 131.7 (Ph C-3,5), 131.8 (Ph C-1), 139.6 (C-3a), 148.0 (C-2), 163.0 (C-4). ^15^N NMR (40 MHz, DMSO-*d_6_*) δ_N_ ppm: −173.4 (N-9), −72.7 (N-1). HRMS (ESI) for C_13_H_12_BrN_3_NaO_2_ ([M+Na]^+^): calcd *m/z* 344.0005, found *m/z* 344.0005 and 345.9983.

##### 7-Hydroxy-2-(4-methoxyphenyl)-5,6,7,8-tetrahydro-4*H*-pyrazolo[1,5-*a*][1,4]diazepin-4-one **4e**

Purified by column chromatography on silica gel (*n*-hexane/ethyl acetate/methanol gradient from 1/9/0 to 1/10/0.5, *v*/*v*). White solid, decomp. 239 °C, procedure a −40% (38 mg). R*_f_* = 0.41 (dichloromethane/methanol 100/5, *v*/*v*). IR (KBr) ν_max_, cm^−1^: 3200, 3078, 2928, 1681 (C=O), 1463, 1447, 1251, 1176, 1027, 821. ^1^H NMR (400 MHz, DMSO-*d_6_*) δ_H_ ppm: 2.91 (dt, *J* = 11.9, 5.7 Hz, 1H, 6-H_a_), 3.25 (dt, *J* = 14.6, 5.4 Hz, 1H, 6-H_b_), 3.78 (s, 3H, OCH_3_), 4.18–4.32 (m, 2H, 7-H, 8-H_a_), 4.51 (dd, *J* = 14.1, 5.1 Hz, 1H, 8-H_b_), 5.48 (d, *J* = 4.2 Hz, 1H, OH), 6.94–7.00 (m, 2H, Ph 3,5-H), 7.07 (s, 1H, 3-H), 7.73–7.79 (m, 2H, Ph 2,6-H), 8.22–8.27 (m, 1H, NH). ^13^C NMR (101 MHz, DMSO-*d_6_*) δ_C_ ppm: 45.6 (C-6), 55.1 (CH_3_), 56.1 (C-8), 69.4 (C-7), 105.2 (C-3), 114.1 (Ph C-3,5), 125.2 (Ph C-1), 126.4 (Ph C-2,6), 139.3 (C-3a), 149.0 (C-3), 156.0 (Ph C-4), 163.2 (C-4). ^15^N NMR (40 MHz, DMSO-*d_6_*) δ_N_ ppm: −270.1 (N-5), −176.5 (N-9), −74.9 (N-1). HRMS (ESI) for C_14_H_15_N_3_NaO_3_ ([M+Na]^+^): calcd *m/z* 296.1006, found *m/z* 296.1006.

##### 7-Hydroxy-5,6,7,8-tetrahydro-4*H*-pyrazolo[1,5-*a*][1,4]diazepin-4-one **4f**

Purified by column chromatography on silica gel (dichloromethane/methanol 100/5, *v*/*v*). White solid, decomp. 195 °C, procedure a −89% (76 mg). R*_f_* = 0.41 (dichloromethane/methanol 9/1, *v*/*v*). IR (KBr) ν_max_, cm^−1^: 3210, 3127, 3081, 2933, 1685 (C=O), 1387, 1351, 1182, 825. ^1^H NMR (400 MHz, DMSO-*d_6_*) δ_H_ ppm: 2.81–2.87 (m, 1H, 6-H_a_), 3.16–3.22 (m, 1H, 6-H_b_), 4.18 (dd, *J* = 14.0, 4.3 Hz, 1H, 8-H_a_), 4.21–4.27 (m, 1H, 7-H), 4.48 (dd, *J* = 14.0, 5.1 Hz, 1H, 8-H_b_), 5.44 (d, *J* = 4.0 Hz, 1H, OH), 6.66 (d, *J* = 0.7 Hz, 1H, 3-H), 7.47 (d, *J* = 0.6 Hz, 1H, 2-H), 8.22 (s, 1H, NH). ^13^C NMR (101 MHz, DMSO-*d_6_*) δ_C_ ppm: 45.6 (C-6), 55.9 (C-8), 69.4 (C-7), 108.8 (C-3), 137.79 (C-3a), 137.83 (C-2), 163.4 (C-4). ^15^N NMR (40 MHz, DMSO-*d_6_*) δ_N_ ppm: –271.1 (N-5), –173.3 (N-9), –66.0 (N-1). HRMS (ESI) for C_7_H_10_N_3_O_2_ ([M+H]^+^): calcd *m/z* 168.0768, found *m/z* 168.0763.

##### 7-Hydroxy-3-methyl-5,6,7,8-tetrahydro-4*H*-pyrazolo[1,5-*a*][1,4]diazepin-4-one **4g**

Purified by column chromatography on silica gel (dichloromethane/methanol 100/5, *v*/*v*). White solid, decomp. 213 °C, procedure a −77% (67 mg). R*_f_* = 0.45 (dichloromethane/methanol 9/1, *v*/*v*). IR (KBr) ν_max_, cm^−1^: 3301, 3203, 3078, 2932, 1678 (C=O), 1382, 1320, 1247, 1099, 922. ^1^H NMR (400 MHz, DMSO-*d_6_*) δ_H_ ppm: 2.10 (s, 3H, CH_3_), 2.77–2.84 (m, 1H, 6-H_a_), 3.12–3.19 (m, 1H, 6-H_b_), 4.09 (dd, *J* = 14.2, 4.2 Hz, 1H, 8-H_a_), 4.17–4.23 (m, 1H, 7-H), 4.40 (dd, *J* = 14.1, 5.4 Hz, 1H, 8-H_b_), 5.39 (d, *J* = 4.1 Hz, 1H, OH), 7.29 (s, 1H, 2-H), 8.14 (s, 1H, NH). ^13^C NMR (101 MHz, DMSO-*d_6_*) δ_C_ ppm: 8.7 (CH_3_), 45.5 (C-6), 55.7 (C-8), 69.7 (C-7), 119.4 (C-3), 133.8 (C-3a), 138.4 (C-2), 164.0 (COO). ^15^N NMR (40 MHz, DMSO-*d_6_*) δ_N_ ppm: –270.3 (N-5), –176.2 (N-9), –69.1 (N-1). HRMS (ESI) for C_8_H_12_N_3_O_2_ ([M+H]^+^): calcd *m/z* 182.0924, found *m/z* 182.0923.

##### 7-Hydroxy-2-methyl-5,6,7,8-tetrahydro-4*H*-pyrazolo[1,5-*a*][1,4]diazepin-4-one **4h**

Purified by column chromatography on silica gel (dichloromethane/methanol 100/5, *v*/*v*). White solid, decomp. 232 °C, procedure a −90% (78 mg). R*_f_* = 0.44 (dichloromethane/methanol 9/1, *v*/*v*). IR (KBr) ν_max_, cm^−1^: 3254, 3144, 2936, 1689 (C=O), 1635, 1464, 1453, 1135, 1054, 758. ^1^H NMR (400 MHz, DMSO-*d_6_*) δ_H_ ppm: 2.16 (s, 1H, CH_3_), 2.82–2.89 (m, 1H, 6-H_a_), 3.15–3.21 (m, 1H, 6-H_b_), 4.08 (dd, *J* = 14.2, 4.4 Hz, 1H, 8-H_a_), 4.17–4.24 (m, 1H, 7-H), 4.38 (dd, *J* = 14.2, 5.4 Hz, 1H, 8-H_b_), 5.40 (d, *J* = 4.1 Hz, 1H, OH), 6.43 (s, 1H, 3-H), 8.15 (s, 1H, NH). ^13^C NMR (101 MHz, DMSO-*d_6_*) δ_C_ ppm: 13.0 (CH_3_), 45.6 (C-6), 55.5 (C-8), 69.5 (C-7), 108.1 (C-3), 138.4 (C-3a), 146.1 (C-2), 163.4 (COO). ^15^N NMR (40 MHz, DMSO-*d_6_*) δ_N_ ppm: –271.6 (N-5), –178.8 (N-9), –68.9 (N-1). HRMS (ESI) for C_8_H_12_N_3_O_2_ ([M+H]^+^): calcd *m/z* 182.0924, found *m/z* 182.0923.

##### 7-Hydroxy-5-(2-methoxyethyl)-2-phenyl-5,6,7,8-tetrahydro-4*H*-pyrazolo[1,5-*a*][1,4]diazepin-4-one **4i**

Purified by column chromatography on silica gel (*n*-hexane/ethyl acetate 1/9, *v*/*v*). White solid, mp 134–135 °C, procedure b −81% (99 mg). R*_f_* = 0.40 (ethyl acetate). IR (KBr) ν_max_, cm^−1^: 3274, 3135, 2932, 1651 (C=O), 1435, 1296, 1177, 1113, 764, 691. ^1^H NMR (400 MHz, DMSO-*d_6_*) δ_H_ ppm: 3.15 (dd, *J* = 15.0, 7.5 Hz, 1H, 5-CH_a_), 3.29 (s, 3H, CH_3_), 3.41–3.56 (m, 4H, 5-CH_b_, CH_2_O, 6-H_a_), 3.89 (dt, *J* = 13.5, 5.2 Hz, 1H, 6-H_b_), 4.24 (dd, *J* = 14.1, 3.2 Hz, 1H, 8-H_a_), 4.35–4.42 (m, 1H, 7-H), 4.47 (dd, *J* = 14.1, 5.1 Hz, 1H, 8-H_b_), 5.52 (d, *J* = 4.3 Hz, 1H, OH), 7.17 (s, 1H, 3-H), 7.28–7.34 (m, 1H, Ph 4-H), 7.37–7.45 (m, 2H, Ph 3,5-H), 7.81–7.86 (m, 2H, Ph 2,6-H). ^13^C NMR (101 MHz, DMSO-*d_6_*) δ_C_ ppm: 46.8 (C-6), 52.8 (5-CH_2_), 55.9 (C-8), 58.0 (CH_3_), 69.5 (C-7), 69.9 (CH_2_O), 105.4 (C-3), 125.1 (Ph C-2,6), 127.8 (Ph C-4), 128.7 (Ph C-3,5), 132.5 (Ph C-1), 139.3 (C-3a), 149.0 (C-2), 161.6 (C-4). ^15^N NMR (40 MHz, DMSO-*d_6_*) δ_N_ ppm: −264.8 (N-5), −175.6 (N-9), −73.7 (N-1). HRMS (ESI) for C_16_H_19_N_3_NaO_3_ ([M+Na]^+^): calcd *m/z* 324.1319, found *m/z* 324.1319.

##### 2-(4-Fluorophenyl)-7-hydroxy-5-(2-methoxyethyl)-5,6,7,8-tetrahydro-4*H*-pyrazolo[1,5-*a*][1,4]diazepin-4-one **4j**

Purified by column chromatography on silica gel (*n*-hexane/ethyl acetate gradient from 4/1 to 1/5, *v*/*v*). White solid, mp 133–134 °C, procedure b −84% (285 mg). R*_f_* = 0.46 (ethyl acetate). IR (KBr) ν_max_, cm^−1^: 3299, 2941, 2903, 2819, 1620, 1471, 1215, 1113, 827, 806. ^1^H NMR (400 MHz, DMSO-*d_6_*) δ_H_ ppm: 3.14 (dd, *J* = 15.0, 7.5 Hz, 1H, 6-H_a_), 3.28 (s, 3H, CH_3_), 3.39–3.57 (m, 4H, 5-CH_a_, 6-H_b_, CH_2_O), 3.89 (dt, *J* = 10.6, 5.0 Hz, 1H, 5-CH_b_), 4.23 (dd, *J* = 14.1, 2.6 Hz, 1H, 8-H_a_), 4.34–4.50 (m, 2H, 7-H, 8-H_b_), 5.52 (d, *J* = 3.9 Hz, 1H, OH), 7.17 (s, 1H, 3-H), 7.20–7.28 (m, 2H, Ph 3,5-H), 7.84–7.92 (m, 5.9 Hz, 2H, Ph 2,6-H). ^13^C NMR (101 MHz, DMSO-*d_6_*) δ_C_ ppm: 46.8 (5-CH_2_), 52.8 (C-6), 55.9 (C-8), 58.0 (CH_3_), 69.5 (C-7), 69.9 (CH_2_O), 105.4 (C-3), 115.59 (d, *^2^J_CF_* = 21.5 Hz, Ph C-3,5), 127.09 (d, *^3^J_CF_* = 8.2 Hz, Ph C-2,6), 129.1 (d, *^4^J_CF_* = 3.0 Hz, Ph C-1), 139.4 (C-3a), 148.1 (C-2), 161.5 (C-4), 161.82 (d, *J_CF_* = 244.3 Hz, Ph C-4). ^15^N NMR (40 MHz, DMSO-*d_6_*) δ_N_ ppm: −264.6 (N-5), −175.6 (N-9), −75.2 (N-1). ^19^F NMR (376 MHz, DMSO-*d_6_*) δ_F_ ppm: −114.4 (1F, m, Ph 6-F). HRMS (ESI) for C_16_H_18_FN_3_NaO_3_ ([M+Na]^+^): calcd *m/z* 342.1224, found *m/z* 342.1224.

##### 2-(4-Chlorophenyl)-7-hydroxy-5-(2-methoxyethyl)-5,6,7,8-tetrahydro-4*H*-pyrazolo[1,5-*a*][1,4]diazepin-4-one **4k**

Purified by column chromatography on silica gel (*n*-hexane/ethyl acetate gradient from 4/1 to 1/9, *v*/*v*). Pale pink solid, mp 148–149 °C, procedure b −98% (221 mg). R*_f_* = 0.41 (ethyl acetate). IR (KBr) ν_max_, cm^−1^: 3300, 2941, 2877, 1623 (C=O), 1469, 1400, 1111, 1068, 826, 756. ^1^H NMR (400 MHz, DMSO-*d_6_*) δ_H_ ppm: 3.14 (dd, *J* = 14.9, 7.4 Hz, 1H, 6-H_a_), 3.29 (s, 3H, CH_3_), 3.40–3.58 (m, 4H, 5-CH_a_, 6-H_b_, CH_2_O), 3.84–3.94 (m, 1H, 5-CH_b_), 4.24 (dd, *J* = 14.1, 2.5 Hz, 1H, 8-H_a_), 4.35–4.51 (m, 2H, 7-H, 8-H_b_), 5.53 (d, *J* = 3.8 Hz, 1H, OH), 7.21 (s, 1H, 3-H), 7.37–7.54 (m, 2H, Ph 3,5-H), 7.77–7.95 (m, 2H, Ph 2,6-H). ^13^C NMR (101 MHz, DMSO-*d_6_*) δ_C_ ppm: 46.8 (5-CH_2_), 52.8 (C-6), 56.0 (C-8), 58.0 (CH_3_), 69.5 (C-7), 69.9 (CH_2_O), 105.7 (C-3), 126.8 (Ph C-2,6), 128.8 (Ph C-3,5), 131.4 (Ph C-1), 132.3 (Ph C-4), 139.5 (C-3a), 147.9 (C-2), 161.4 (C-4). ^15^N NMR (40 MHz, DMSO-*d_6_*) δ_N_ ppm: −264.3 (N-5), −174.7 (N-9), −74.3 (N-1). HRMS (ESI) for C_16_H_18_ClN_3_NaO_3_ ([M+Na]^+^): calcd *m/z* 358.0929, found *m/z* 358.0929.

##### 2-(4-Bromophenyl)-7-hydroxy-5-(2-methoxyethyl)-5,6,7,8-tetrahydro-4*H*-pyrazolo[1,5-*a*][1,4]diazepin-4-one **4l**

Purified by column chromatography on silica gel (*n*-hexane/ethyl acetate gradient from 3/1 to 1/9, *v*/*v*). White solid, mp 143–144 °C, procedure b −84% (339 mg). R*_f_* = 0.43 (ethyl acetate). IR (KBr) ν_max_, cm^−1^: 3292, 2943, 2876, 1621 (C=O), 1545, 1467, 1107, 1070, 1007, 812. ^1^H NMR (400 MHz, DMSO-*d_6_*) δ_H_ ppm: 3.14 (dd, *J* = 15.0, 7.6 Hz, 1H, 6-H_a_), 3.28 (s, 3H, CH_3_), 3.40–3.57 (m, 4H, 6-H_b_, NCH_a_, CH_2_O), 3.89 (dt, *J* = 10.6, 5.1 Hz, 1H, NCH_b_), 4.23 (dd, *J* = 14.1, 2.9 Hz, 1H, 8-H_a_), 4.35–4.42 (m, 1H, 7-H), 4.47 (dd, *J* = 14.1, 5.0 Hz, 1H, 8-H_b_), 5.53 (d, *J* = 3.2 Hz, 1H, OH), 7.21 (s, 1H, 3-H), 7.56–7.63 (m, 2H, Ph 3,5-H), 7.76–7.83 (m, 2H, Ph 2,6-H). ^13^C NMR (101 MHz, DMSO-*d_6_*) δ_C_ ppm: 46.8 (NCH_2_), 52.8 (C-6), 56.0 (C-8), 58.0 (CH_3_), 69.5 (7-H), 69.9 (CH_2_O), 105.6 (C-3), 120.8 (Ph C-4), 127.1 (Ph C-2,6), 131.7 (Ph C-3,5), 131.8 (Ph C-1), 139.5 (C-3a), 147.9 (C-2), 161.4 (C-4). ^15^N NMR (40 MHz, DMSO-*d_6_*) δ_N_ ppm: −264.5 (N-5), –174.5 (N-9), −73.9 (N-1). HRMS (ESI) for C_16_H_18_BrN_3_NaO_3_ ([M+Na]^+^): calcd *m/z* 402.0424, found *m/z* 402.0424 and 404.0409.

##### 7-Hydroxy-5-(2-methoxyethyl)-2-(4-methoxyphenyl)-5,6,7,8-tetrahydro-4*H*-pyrazolo[1,5-*a*][1,4]diazepin-4-one **4m**

Purified by column chromatography on silica gel (*n*-hexane/ethyl acetate gradient from 3.5/1 to 1/9, *v*/*v*). White solid, mp 147–148 °C, procedure b −80% (274 mg). R*_f_* = 0.38 (ethyl acetate). IR (KBr) ν_max_, cm^−1^: 3212, 2982, 2943, 2882, 2838, 1651 (C=O), 1448, 1249, 1020, 834. ^1^H NMR (400 MHz, DMSO-*d_6_*) δ_H_ ppm: 3.15 (dd, *J* = 15.0, 7.4 Hz, 1H, 6-H_a_), 3.28 (s, 3H, OCH_3_), 3.39–3.57 (m, 4H, 5-CH_a_, CH_2_O, 6-H_b_), 3.78 (s, 3H, Ph 4-OCH_3_), 3.89 (dt, *J* = 13.6, 5.2 Hz, 1H, 5-CH_b_), 4.21 (dd, *J* = 14.0, 3.1 Hz, 1H, 8-H_a_), 4.34–4.47 (m, 2H, 7-H, 8-H_b_), 5.52 (d, *J* = 4.2 Hz, 1H, OH), 6.93–6.99 (m, 2H, Ph 3,5-H), 7.07 (s, 1H, 3-H), 7.73–7.80 (m, 2H, Ph 2,6-H). ^13^C NMR (101 MHz, DMSO-*d_6_*) δ_C_ ppm: 46.8 (5-CH_2_), 52.9 (C-6), 55.1 (CH_3_), 55.8 (C-8), 58.1 (OCH_3_), 69.6 (C-7), 70.0 (CH_2_O), 104.9 (C-3), 114.1 (Ph C-3,5), 125.2 (Ph C-1), 126.4 (Ph C-2,6), 139.2 (C-3a), 148.9 (C-2), 159.0 (Ph C-4), 161.7 (C-4). ^15^N NMR (40 MHz, DMSO-*d_6_*) δ_N_ ppm: −264.5 (N-5), −176.2 (N-9), −77.0 (N-1). HRMS (ESI) for C_17_H_21_N_3_NaO_4_ ([M+Na]^+^): calcd *m/z* 354.1424, found *m/z* 354.1424.

##### 5-Allyl-7-hydroxy-2-phenyl-5,6,7,8-tetrahydro-4*H*-pyrazolo[1,5-*a*][1,4]diazepin-4-one **4n**

Purified by column chromatography on silica gel (*n*-hexane/ethyl acetate 1/9, *v*/*v*). White solid, mp 126–127 °C, procedure b −71% (231 mg). R*_f_* = 0.42 (*n*-hexane/ethyl acetate 3/7, *v*/*v*). IR (KBr) ν_max_, cm^−1^: 3346, 2939, 1619 (C=O), 1470, 1431, 1286, 1055, 942, 749, 690. ^1^H NMR (400 MHz, DMSO-*d_6_*) δ_H_ ppm: 3.09 (dd, *J* = 15.0, 7.3 Hz, 1H, 6-H_a_), 3.38 (dd, *J* = 15.0, 5.1 Hz, 1H, 6-H_b_), 3.94 (dd, *J* = 15.1, 6.4 Hz, 1H, 5-CH_a_), 4.20–4.31 (m, 2H, 5-CH_b_, 8-H_a_), 4.34–4.42 (m, 1H, 7-H), 4.55 (dd, *J* = 14.4, 5.4 Hz, 1H, 8-H_b_), 5.18–5.29 (m, 2H, CH_2_-CH=CH_2_), 5.55 (d, *J* = 4.2 Hz, 1H, OH), 5.87 (ddd, *J* = 22.5, 10.8, 5.9 Hz, 1H, CH_2_-CH=CH_2_), 7.19 (s, 1H, 3-H), 7.29–7.35 (m, 1H, Ph 4-H), 7.37–7.44 (m, 2H, Ph 3,5-H), 7.81–7.88 (m, 2H, Ph 2,6-H). ^13^C NMR (101 MHz, DMSO-*d_6_*) δ_C_ ppm: 49.6 (5-CH_2_), 51.3 (C-6), 55.9 (C-8), 69.3 (C-7), 105.8 (C-3), 117.8 (CH_2_-CH=CH_2_), 125.1 (Ph C-2,6), 127.8 (Ph C-4), 128.7 (Ph C-3,5), 132.5 (Ph C-1), 133.5 (CH_2_-CH=CH_2_), 139.2 (C-3a), 149.0 (C-2), 161.3 (C-4). ^15^N NMR (40 MHz, DMSO-*d_6_*) δ_N_ ppm: −263.8 (N-5), −175.4 (N-9), −73.8 (N-1). HRMS (ESI) for C_16_H_17_N_3_NaO_2_ ([M+Na]^+^): calcd *m/z* 306.1213, found *m/z* 306.1213.

##### 5-Allyl-2-(4-fluorophenyl)-7-hydroxy-5,6,7,8-tetrahydro-4*H*-pyrazolo[1,5-*a*][1,4]diazepin-4-one **4o**

Purified by column chromatography on silica gel (*n*-hexane/ethyl acetate gradient from 7/2 to 1/9, *v*/*v*). White solid, mp 143–144 °C, procedure b −89% (175 mg). R*_f_* = 0.39 (*n*-hexane/ethyl acetate 3/7, *v*/*v*). IR (KBr) ν_max_, cm^−1^: 3230, 2951, 2892, 1651, 1461, 1440, 933, 837, 816, 756. ^1^H NMR (400 MHz, DMSO-*d_6_*) δ_H_ ppm: 3.08 (dd, *J* = 15.0, 7.3 Hz, 1H, 6-H_a_), 3.38 (dd, *J* = 15.0, 5.1 Hz, 1H, 6-H_b_), 3.94 (dd, *J* = 15.2, 6.3 Hz, 1H, 5-CH_a_), 4.19–4.32 (m, 2H, 5-CH_b_, 8-H_a_), 4.34–4.42 (m, 1H, 7-H), 4.54 (dd, *J* = 14.3, 5.3 Hz, 1H, 8-H_b_), 5.18–5.28 (m, *J* = 14.0 Hz, 2H, CH_2_-CH=CH_2_), 5.55 (d, *J* = 4.1 Hz, 1H, OH), 5.87 (dq, *J* = 10.9, 5.8 Hz, 1H, CH_2_-CH=CH_2_), 7.20 (s, 1H, 3-H), 7.21–7.29 (m, 2H, Ph 3,5-H), 7.84–7.92 (m, 2H, Ph 2,6-H). ^13^C NMR (101 MHz, DMSO-*d_6_*) δ_C_ ppm: 49.6 (5-CH_2_), 51.3 (C-6), 55.9 (C-8), 69.3 (C-7), 105.7 (C-3), 115.59 (d, *^2^J_CF_* = 21.5 Hz, Ph C-3,5), 117.8 (CH_2_-CH=CH_2_), 127.10 (d, *^3^J_CF_* = 8.2 Hz, Ph C-2,6), 129.07 (d, *^4^J_CF_* = 3.0 Hz, Ph C-1), 133.5 (CH_2_-CH=CH_2_), 139.2 (C-3a), 148.1 (C-2), 161.3 (C-4), 161.83 (d, *J_CF_* = 244.3 Hz, Ph C-4). ^15^N NMR (40 MHz, DMSO-*d_6_*) δ_N_ ppm: −264.0 (N-5), −175.1 (N-9), −74.3 (N-1). ^19^F NMR (376 MHz, DMSO-*d_6_*) δ_F_ ppm: −114.4 (Ph 6-F). HRMS (ESI) for C_16_H_16_FN_3_NaO_2_ ([M+Na]^+^): calcd *m/z* 324.1119, found *m/z* 324.1119.

##### 5-Allyl-2-(4-chlorophenyl)-7-hydroxy-5,6,7,8-tetrahydro-4*H*-pyrazolo[1,5-*a*][1,4]diazepin-4-one **4p**

Purified by column chromatography on silica gel (*n*-hexane/ethyl acetate gradient from 7/2 to 1/9, *v*/*v*). White solid, mp 179–180 °C, procedure b −92% (188 mg). R*_f_* = 0.42 (*n*-hexane/ethyl acetate 3/7, *v*/*v*). IR (KBr) ν_max_, cm^−1^: 3363, 2939, 2891, 1620, 1470, 1091, 1053, 939, 812, 753. ^1^H NMR (400 MHz, DMSO-*d_6_*) δ_H_ ppm: 3.08 (dd, *J* = 15.0, 7.3 Hz, 1H, 6-H_a_), 3.38 (dd, *J* = 15.0, 5.1 Hz, 1H, 6-H_b_), 3.94 (dd, *J* = 15.1, 6.3 Hz, 1H, 5-H_a_), 4.19–4.42 (m, 3H, 5-CH_b_, 7-H, 8-H_a_), 4.55 (dd, *J* = 14.4, 5.4 Hz, 1H, 8-H_b_), 5.19–5.29 (m, 2H, CH_2_-CH=CH_2_), 5.55 (d, *J* = 4.2 Hz, 1H, OH), 5.86 (ddd, *J* = 22.5, 10.8, 5.9 Hz, 1H, CH_2_-CH=CH_2_), 7.23 (s, 1H, 3-H), 7.43–7.51 (m, 2H, Ph 3,5-H), 7.83–7.91 (m, 2H, Ph 2,6-H). ^13^C NMR (101 MHz, DMSO-*d_6_*) δ_C_ ppm: 49.6 (5-CH_2_), 51.2 (C-6), 56.0 (C-8), 69.3 (C-7), 106.0 (C-3), 117.8 (CH_2_-CH=CH_2_), 126.8 (Ph C-2,6), 128.8 (Ph C-3,5), 131.4 (Ph C-1), 132.3 (Ph C-4), 133.4 (CH_2_-CH=CH_2_), 139.3 (C-3a), 147.9 (C-2), 161.2 (C-4). ^15^N NMR (40 MHz, DMSO-*d_6_*) δ_N_ ppm: −264.1 (N-5), −174.4 (N-9), −73.7 (N-1). HRMS (ESI) for C_16_H_16_ClN_3_NaO_2_ ([M+Na]^+^): calcd *m/z* 340.0823, found *m/z* 340.0823.

##### 5-Allyl-2-(4-bromophenyl)-7-hydroxy-5,6,7,8-tetrahydro-4*H*-pyrazolo[1,5-*a*][1,4]diazepin-4-one **4r**

Purified by column chromatography on silica gel (*n*-hexane/ethyl acetate gradient 1/9, *v*/*v*). White solid, mp 186–187 °C, procedure b −76% (277 mg). R*_f_* = 0.43 (*n*-hexane/ethyl acetate 3/7, *v*/*v*). IR (KBr) ν_max_, cm^−1^: 3366, 2938, 1621 (C=O), 1469, 1434, 1052, 1006, 933, 811, 752. ^1^H NMR (400 MHz, DMSO-*d_6_*) δ_H_ ppm: 3.08 (dd, *J* = 14.9, 7.3 Hz, 1H, 6-H_a_), 3.35–3.42 (m, 1H, 6-H_b_), 3.94 (dd, *J* = 15.1, 6.2 Hz, 1H, 5-CH_a_), 4.19–4.32 (m, 2H, 5-CH_b_, 8-H_a_), 4.34–4.41 (m, 1H, 7-H), 4.55 (dd, *J* = 14.3, 5.3 Hz, 1H, 8-H_b_), 5.18–5.28 (m, 2H, CH_2_-CH=CH_2_), 5.56 (d, *J* = 4.0 Hz, 1H, OH), 5.81–5.92 (m, 1H, CH_2_-CH=CH_2_), 7.23 (s, 1H, 3-H), 7.57–7.63 (m, 2H, Ph 3,5-H), 7.78–7.84 (m, 2H, Ph 2,6-H). ^13^C NMR (101 MHz, DMSO-*d_6_*) δ_C_ ppm: 49.6 (5-CH_2_), 51.2 (C-6), 56.0 (C-8), 69.3 (C-7), 106.0 (C-3), 117.8 (CH_2_-CH=CH_2_), 120.9 (Ph C-4), 127.1 (Ph C-2,6), 131.67 (Ph C-3,5), 131.73 (Ph C-1), 133.4 (CH_2_-CH=CH_2_), 139.3 (C-3a), 147.9 (C-2), 161.2 (C-4). ^15^N NMR (40 MHz, DMSO-*d_6_*) δ_N_ ppm: −263.2 (N-5), −173.1 (N-9), −74.0 (N-1). HRMS (ESI) for C_16_H_16_BrN_3_NaO_2_ ([M+Na]^+^): calcd *m/z* 384.0318, found *m/z* 384.0318 and 386.0303.

##### 5-Allyl-7-hydroxy-2-(4-methoxyphenyl)-5,6,7,8-tetrahydro-4*H*-pyrazolo[1,5-*a*][1,4]diazepin-4-one **4s**

Purified by column chromatography on silica gel (*n*-hexane/ethyl acetate gradient from 4/1 to 1/9, *v*/*v*). White solid, mp 134–135 °C, procedure b −85% (301 mg). R*_f_* = 0.35 (*n*-hexane/ethyl acetate 1.5/3.5, *v*/*v*). IR (KBr) ν_max_, cm^−1^: 3284, 2932, 2835, 1624 (C=O), 1467, 1285, 1061, 1026, 935, 828. ^1^H NMR (400 MHz, DMSO-*d_6_*) δ_H_ ppm: 3.08 (dd, *J* = 15.0, 7.2 Hz, 1H, 6-H_a_), 3.37 (dd, *J* = 14.8, 4.9 Hz, 1H, 6-H_b_), 3.78 (s, 3H, Ph 4-OCH_3_), 3.94 (dd, *J* = 15.1, 6.4 Hz, 1H, 5-CH_a_), 4.20 (dd, *J* = 14.3, 3.8 Hz, 1H, 8-H_a_), 4.28 (dd, *J* = 15.1, 5.3 Hz, 1H, 5-CH_b_), 4.33–4.40 (m, 1H, 7-H), 4.51 (dd, *J* = 14.3, 5.4 Hz, 1H, 8-H_b_), 5.17–5.28 (m, 2H, CH_2_-CH=CH_2_), 5.54 (d, *J* = 4.2 Hz, 1H, OH), 5.86 (ddd, *J* = 22.5, 10.3, 5.9 Hz, 1H, CH_2_-CH=CH_2_), 6.93–7.00 (m, 2H, Ph 3,5-H), 7.10 (s, 1H, 3-H), 7.72–7.80 (m, 2H, Ph 2,6-H). ^13^C NMR (101 MHz, DMSO-*d_6_*) δ_C_ ppm: 49.6 (5-CH_2_), 51.3 (C-6), 55.1 (Ph 4-OCH_3_), 55.8 (C-8), 69.4 (C-7), 105.2 (C-3), 114.1 (Ph C-3,5), 117.8 (CH_2_-CH=CH_2_), 125.2 (Ph C-1), 126.4 (Ph C-2,6), 133.5 (CH_2_-CH=CH_2_), 139.0 (C-3a), 148.9 (C-2), 159.0 (Ph C-4), 161.4 (C-4). ^15^N NMR (40 MHz, DMSO-*d_6_*) δ_N_ ppm: −176.8 (N-9), −75.9 (N-1). HRMS (ESI) for C_17_H_19_N_3_NaO_3_ ([M+Na]^+^): calcd *m/z* 336.1319, found *m/z* 336.1319.

##### 5-Benzyl-7-hydroxy-2-phenyl-5,6,7,8-tetrahydro-4*H*-pyrazolo[1,5-*a*][1,4]diazepin-4-one **4t**

Purified by column chromatography on silica gel (*n*-hexane/ethyl acetate 1/9, *v*/*v*). Pale yellow solid, mp 167–168 °C, procedure b −87% (138 mg). R*_f_* = 0.55 (*n*-hexane/ethyl acetate 3/7, *v*/*v*). IR (KBr) ν_max_, cm^−1^: 3257, 3143, 2892, 1649 (C=O), 1451, 1235, 765 and 754 (doublet), 705 and 692 (doublet). ^1^H NMR (400 MHz, DMSO-*d_6_*) δ_H_ ppm: 3.08 (dd, *J* = 14.9, 7.5 Hz, 1H, 6-H_a_), 3.37 (dd, *J* = 15.0, 5.2 Hz, 1H, 6-H_b_), 4.12–4.19 (m, 1H, 7-H), 4.24 (dd, *J* = 14.4, 3.5 Hz, 1H, 8-H_a_), 4.43–4.56 (m, 2H, 5-CH_a_, 8-H_b_), 4.96 (d, *J* = 14.7 Hz, 1H, 5-CH_b_), 5.51 (d, J = 4.1 Hz, 1H, OH), 7.25 (s, 1H, 3-H), 7.29–7.35 (m, 2H, CPh 4-H, CH_2_Ph 4-H), 7.36–7.44 (m, 6H, CPh 3,5-H, CH_2_Ph 2,3,5,6-H), 7.82–7.88 (m, 2H, CPh 2,6-H). ^13^C NMR (101 MHz, DMSO-*d_6_*) δ_C_ ppm: 50.3 (5-CH_2_), 51.3 (C-6), 55.9 (C-8), 69.3 (C-7), 105.9 (C-3), 125.1 (CPh C-2,6), 127.5 (CH_2_Ph C-4), 127.8 (CPh C-4), 128.1 (CH_2_Ph C-2,6), 128.6 (CH_2_Ph C-3,5), 128.7 (CPh C-3,5), 132.5 (CPh C-1), 137.5 (CH_2_Ph C-1), 139.0 (C-3a), 149.0 (C-2), 161.7 (C-4). ^15^N NMR (40 MHz, DMSO-*d_6_*) δ_N_ ppm: −259.1 (N-5), −176.3 (N-9), −73.3 (N-1). HRMS (ESI) for C_20_H_19_N_3_NaO_2_ ([M+Na]^+^): calcd *m/z* 356.1369, found *m/z* 356.1370.

##### 5-Benzyl-2-(4-fluorophenyl)-7-hydroxy-5,6,7,8-tetrahydro-4*H*-pyrazolo[1,5-*a*][1,4]diazepin-4-one **4u**

Purified by column chromatography on silica gel (*n*-hexane/ethyl acetate gradient from 4/1 to 1/9, *v*/*v*). Pale yellow solid, mp 192–193 °C, procedure b −70% (301 mg). R*_f_* = 0.52 (*n*-hexane/ethyl acetate 1.5/3.5, *v*/*v*). IR (KBr) ν_max_, cm^−1^: 3291, 3144, 2945, 1617 (C=O), 1470, 1442, 1230, 839, 701, 639. ^1^H NMR (400 MHz, DMSO-*d_6_*) δ_H_ ppm: 3.07 (dd, *J* = 14.9, 7.5 Hz, 1H, 6-H_a_), 3.34–3.40 (m, 1H, 6-H_b_), 4.11–4.26 (m, 2H, 7-H, 8-Ha), 4.43–4.55 (m, 2H, 5-CH_a_, 8-H_b_), 4.96 (d, *J* = 14.7 Hz, 1H, 5-CH_b_), 5.50 (d, *J* = 4.0 Hz, 1H, OH), 7.20–7.41 (m, 8H, 3-H, CPh 3,5-H, CH_2_Ph 2,3,4,5,6-H), 7.85–7.93 (m, 2H, CPh 2,6-H). ^13^C NMR (101 MHz, DMSO-*d_6_*) δ_C_ ppm: 50.3 (5-CH_2_), 51.3 (C-6), 55.9 (C-8), 69.2 (C-7), 105.9 (C-3), 115.61 (d, *^2^J_CF_* = 21.5 Hz, CPh C-3,5), 127.10 (d, *^3^J_CF_* = 8.2 Hz, CPh C-2,6), 127.5 (CH_2_Ph C-4), 128.1 (CH_2_Ph C-2,6), 128.6 (CH_2_Ph C-3,5), 129.06 (d, *^4^J_CF_* = 2.8 Hz, CPh C-1), 137.5 (CH_2_Ph C-1), 139.1 (C-3a), 148.2 (C-2), 161.6 (C-4), 161.84 (d, *J_CF_* = 244.3 Hz, CPh C-4). ^15^N NMR (40 MHz, DMSO-*d_6_*) δ_N_ ppm: −259.9 (N-5), −175.3 (N-9), −74.4 (N-1). ^19^F NMR (376 MHz, DMSO-*d_6_*) δ_F_ ppm: −114.3 (CPh 6-F). HRMS (ESI) for C_20_H_18_FN_3_NaO_2_ ([M+Na]^+^): calcd *m/z* 374.1275, found *m/z* 374.1275.

##### 5-Benzyl-2-(4-chlorophenyl)-7-hydroxy-5,6,7,8-tetrahydro-4*H*-pyrazolo[1,5-*a*][1,4]diazepin-4-one **4v**

Purified by column chromatography on silica gel (*n*-hexane/ethyl acetate gradient from 7/2 to 1/9, *v*/*v*). White solid, mp 181–182 °C, procedure b −96% (419 mg). R*_f_* = 0.57 (*n*-hexane/ethyl acetate 3/7, *v*/*v*). IR (KBr) ν_max_, cm^−1^: 3246, 2939, 1619 (C=O), 1455, 1434, 1091, 830 and 819 (doublet), 757, 699. ^1^H NMR (400 MHz, DMSO-*d_6_*) δ_H_ ppm: 3.08 (dd, *J* = 14.9, 7.5 Hz, 1H, 6-H_a_), 3.37 (dd, *J* = 15.0, 5.0 Hz, 1H, 6-H_b_), 4.11–4.27 (m, 2H, 7-H, 8-H_a_), 4.43–4.57 (m, 2H, 5-CH_a_, 8-H_b_), 4.96 (d, *J* = 14.7 Hz, 1H, 5-CH_b_), 5.51 (d, *J* = 3.6 Hz, 1H, OH), 7.28–7.41 (m, 6H, 3-H, CH_2_Ph 2,3,4,5,6-H), 7.44–7.50 (m, 2H, CPh 3,5-H), 7.84–7.91 (m, 2H, CPh 2,6-H). ^13^C NMR (101 MHz, DMSO-*d_6_*) δ_C_ ppm: 50.3 (5-CH_2_), 51.3 (C-6), 56.0 (C-8), 69.2 (C-7), 106.1 (C-3), 126.8 (CPh C-2,6), 127.5 (CH_2_Ph C-4), 128.1 (CH_2_Ph C-2,6), 128.6 (CH_2_Ph C-3,5), 128.8 (CPh C-3,5), 131.4 (CPh C-1), 132.3 (CPh C-4), 137.5 (CH_2_Ph C-1), 139.2 (C-3a), 147.9 (C-2), 161.6 (C-4). ^15^N NMR (40 MHz, DMSO-*d_6_*) δ_N_ ppm: −259.6 (N-5), −174.8 (N-9), −73.4 (N-1). HRMS (ESI) for C_20_H_18_ClN_3_NaO_2_ ([M+Na]^+^): calcd *m/z* 390.0980, found *m/z* 390.0980.

##### 5-Benzyl-2-(4-bromophenyl)-7-hydroxy-5,6,7,8-tetrahydro-4*H*-pyrazolo[1,5-*a*][1,4]diazepin-4-one **4w**

Purified by column chromatography on silica gel (*n*-hexane/ethyl acetate 1/9, *v*/*v*). Pale yellow solid, mp 157–158 °C, procedure b −72% (287 mg). R*_f_* = 0.57 (*n*-hexane/ethyl acetate 3/7, *v*/*v*). IR (KBr) ν_max_, cm^−1^: 3270, 1619 (C=O), 1460, 1429, 1247, 1068, 1009, 816, 756, 701. ^1^H NMR (400 MHz, DMSO-*d_6_*) δ_H_ ppm: 3.08 (dd, *J* = 14.9, 7.6 Hz, 1H, 6-H_a_), 3.34–3.41 (m, 1H, 6-H_b_), 4.12–4.18 (m, 1H, 7-H), 4.20–4.27 (m, 1H, 8-H_a_), 4.46 (d, *J* = 14.7 Hz, 1H, 5-CH_a_), 4.52 (dd, *J* = 14.4, 5.3 Hz, 1H, 8-H_b_), 4.96 (d, *J* = 14.7 Hz, 1H, 5-CH_b_), 5.51 (d, *J* = 4.1 Hz, 1H, OH), 7.28–7.34 (m, 2H, 3-H, CH_2_Ph 4-H), 7.35–7.39 (m, 4H, CH_2_Ph 2,3,5,6-H), 7.56–7.64 (m, 2H, CPh 3,5-H), 7.78–7.85 (m, 2H, CPh 2,6-H). ^13^C NMR (101 MHz, DMSO-*d_6_*) δ_C_ ppm: 50.4 (5-CH_2_), 51.3 (C-6), 56.0 (C-8), 69.2 (C-7), 106.1 (C-3), 120.9 (CPh C-4), 127.1 (CPh C-2,6), 127.5 (CH_2_Ph C-4), 128.2 (CH_2_Ph C-3,5), 128.6 (CH_2_Ph C-2,6), 131.68 (CPh C-3,5), 131.72 (CPh C-1), 137.5 (CH_2_Ph C-1), 139.2 (C-3a), 148.0 (C-2), 161.6 (C-4). ^15^N NMR (40 MHz, DMSO-*d_6_*) δ_N_ ppm: −259.5 (N-5), −174.5 (N-9), −73.7 (N-1). HRMS (ESI) for C_20_H_18_BrN_3_NaO_2_ ([M+Na]^+^): calcd *m/z* 434.0475, found *m/z* 434.0475 and 436.0445.

##### 5-Benzyl-7-hydroxy-2-(4-methoxyphenyl)-5,6,7,8-tetrahydro-4*H*-pyrazolo[1,5-*a*][1,4]diazepin-4-one **4x**

Purified by column chromatography on silica gel (*n*-hexane/ethyl acetate gradient from 3.5/1 to 1/9, *v*/*v*). White solid, mp 179–180 °C, procedure b −72% (313 mg). R*_f_* = 0.46 (*n*-hexane/ethyl acetate 1.5/3.5, *v*/*v*). IR (KBr) ν_max_, cm^−1^: 3204, 1651 (C=O), 1462, 1436, 1250, 1180, 1029, 833, 753, 705. ^1^H NMR (400 MHz, DMSO-*d_6_*) δ_H_ ppm: 3.07 (dd, *J* = 14.9, 7.5 Hz, 1H, 6-H_a_), 3.36–3.40 (m, 1H, 6-H_b_), 3.78 (s, 3H, -OCH_3_), 4.11–4.24 (m, 2H, 7-H, 8-H_a_), 4.42–4.53 (m, 2H, 5-CH_a_, 8-H_b_), 4.96 (d, *J* = 14.7 Hz, 1H, 5-CH_b_), 5.50 (s, 1H, OH), 6.94–7.00 (m, 2H, CPh 3,5-H), 7.15 (s, 1H, 3-H), 7.28–7.34 (m, 1H, CH_2_Ph 4-H), 7.35–7.41 (m, 4H, CH_2_Ph 2,3,5,6-H), 7.74–7.81 (m, 2H, CPh 2,6-H). ^13^C NMR (101 MHz, DMSO-*d_6_*) δ_C_ ppm: 50.4 (5-CH_2_), 51.4 (C-6), 55.1 (OCH_3_), 55.8 (C-8), 69.3 (C-7), 105.3 (C-3), 114.1 (CPh C-3,5), 125.1 (CPh C-1), 126.4 (CPh C-2,6), 127.5 (CH_2_Ph C-4), 128.1 (CH_2_Ph C-2,6), 128.6 (CH_2_Ph C-3,5), 137.6 (CH_2_Ph C-1), 138.9 (C-3a), 149.0 (C-2), 159.0 (CPh C-4), 161.8 (C-4). ^15^N NMR (40 MHz, DMSO-*d_6_*) δ_N_ ppm: −258.9 (N-5), −176.9 (N-9), −75.4 (N-1). HRMS (ESI) for C_21_H_21_N_3_NaO_3_ ([M+Na]^+^): calcd *m/z* 386.1475, found *m/z* 386.1475.

#### 3.2.4. Synthesis of Ethyl 1-(oxiran-2-ylmethyl)-1*H*-indole-2-carboxylates (**6a–e**) and Ethyl 1-(oxiran-2-ylmethyl)-1*H*-benzo[*d*]imidazole-2-carboxylate (**6f**)

**Procedure a**. Indole-2-carboxylate **5a–e** (1 eq) was dissolved in dry dimethyl formamide (0.2 M) and KOH (3 eq; flakes) was added followed by 2-(chloromethyl)oxirane (1.5 eq). The reaction mixture was stirred at 40 °C for 1 h under argon atmosphere. Upon completion, the mixture was concentrated to approximately 1/3 volume, diluted with ethyl acetate, and washed with brine. Organic layer was separated, dried over anhydrous Na_2_SO_4_, filtered, and concentrated under reduced pressure. The residue was purified by column chromatography.

**Procedure b.** Benzimidazole-2-carboxylate **5f** (1 eq) was dissolved in dry dimethyl formamide (0.3 M) and NaH (1.5 eq; 60% dispersion in mineral oil) was added followed by 2-(chloromethyl)oxirane (1.5 eq). The reaction mixture was stirred at 60 °C for 4 h. Upon completion, the reaction mixture was cooled to 0 °C, quenched with water and extracted with ethyl acetate. The combined organic phases were washed with brine, dried over anhydrous Na_2_SO_4_, filtered, and concentrated under reduced pressure. The residue was purified by column chromatography.

##### Ethyl 1-(oxiran-2-ylmethyl)-1*H*-indole-2-carboxylate **6a**

Purified by column chromatography on silica gel (*n*-hexane/ethyl acetate 8/1, *v*/*v*). White solid, mp 47–49 °C, procedure a −73% (94 mg). R*_f_* = 0.29 (*n*-hexane/ethyl acetate 6/1, *v*/*v*). IR (KBr) ν_max_, cm^−1^: 3064, 2974, 2928, 1713 (C=O), 1408, 1265, 1205, 1140, 1096, 744. ^1^H NMR (400 MHz, CDCl_3_) δ_H_ ppm: 1.41 (t, *J* = 7.1 Hz, 3H, CH_3_), 2.49–2.53 (m, 1H, Ox CH_a_H_b_), 2.77 (t, *J* = 4.2 Hz, 1H, Ox CH_a_H_b_), 3.33–3.39 (m, 1H, Ox CH), 4.38 (q, *J* = 7.1 Hz, 2H, CH_2_CH_3_), 4.56 (dd, *J* = 15.1, 5.0 Hz, 1H, NCH_a_H_b_), 5.03 (dd, *J* = 15.1, 2.4 Hz, 1H, NCH_a_H_b_), 7.14–7.18 (m, 1H, 5-H), 7.34–7.37 (m, 2H, 3-H, 6-H), 7.45–7.47 (m, 1H, 7-H), 7.66–7.68 (m, 1H, 4-H). ^13^C NMR (101 MHz, CDCl_3_) δ_C_ ppm: 14.5 (CH_3_), 45.5 (Ox CH_a_H_b_), 46.2 (NCH_a_H_b_), 51.6 (Ox CH), 60.8 (CH_2_CH_3_), 110.9 (C-7), 111.1 (C-3), 121.1 (C-5), 122.7 (C-4), 125.5 (C-6), 126.1 (C-3a), 127.7 (C-2), 140.0 (C-7a), 162.3 (COO). ^15^N NMR (40 MHz, CDCl_3_) δ_N_ ppm: –250.9 (N). HRMS (ESI) for C_14_H_16_NO_3_ ([M+H]^+^): calcd *m/z* 246.1125, found *m/z* 246.1119.

##### Ethyl 5-fluoro-1-(oxiran-2-ylmethyl)-1*H*-indole-2-carboxylate **6b**

Purified by column chromatography on silica gel (*n*-hexane/ethyl acetate 8/1, *v*/*v*). White solid, mp 65–66 °C, procedure a −39% (50 mg). R*_f_* = 0.19 (*n*-hexane/ethyl acetate 6/1, *v*/*v*). IR (KBr) ν_max_, cm^−1^: 2974, 2930, 1713 (C=O), 1527, 1266, 1246, 1177, 1091, 756. ^1^H NMR (400 MHz, CDCl_3_) δ_H_ ppm: 1.41 (t, *J* = 7.1 Hz, 3H, CH_3_), 2.45–2.53 (m, 1H, Ox CH_a_H_b_), 2.79 (t, *J* = 4.2 Hz, 1H, Ox CH_a_H_b_), 3.32–3.40 (m, 1H, Ox CH), 4.38 (q, *J* = 7.1 Hz, 2H, CH_2_CH_3_), 4.47 (dd, *J* = 15.2, 5.3 Hz, 1H, NCH_a_H_b_), 5.08 (dd, *J* = 15.2, 2.4 Hz, 1H, NCH_a_H_b_), 7.07–7.15 (m, 1H, 6-H), 7.24–7.33 (m, 2H, 3-H, 7-H), 7.38–7.45 (m, 1H, 4-H). ^13^C NMR (101 MHz, CDCl_3_) δ_H_ ppm: 14.5 (CH_3_), 45.3 (Ox CH_a_H_b_), 46.6 (NCH_a_H_b_), 51.7 (Ox CH), 61.0 (CH_2_CH_3_), 106.7 (d, *J* = 23.2 Hz, C-7), 110.7 (d, *J* = 5.1 Hz, C-3), 112.1 (d, *J* = 10.1 Hz, C-4), 114.6 (d, *J* = 27.3 Hz, C-6), 126.1 (d, *J* = 11.1 Hz, C-3a), 129.0 (C-2), 136.7 (C-7a), 158.4 (d, *J* = 238.4 Hz, C-5), 162.0 (COO). ^15^N NMR (40 MHz, CDCl_3_) δ_N_ ppm: –250.9 (N). ^19^F NMR (376 MHz, DMSO-*d_6_*) δ_F_ ppm: –127.7 (F). HRMS (ESI) for C_14_H_15_FNO_3_ ([M+H]^+^): calcd *m/z* 264.1030, found *m/z* 264.1031.

##### Ethyl 5-chloro-1-(oxiran-2-ylmethyl)-1*H*-indole-2-carboxylate **6c**

Purified by column chromatography on silica gel (*n*-hexane/ethyl acetate 8/1, *v*/*v*). White solid, mp 79–80 °C, procedure a −69% (72 mg). R*_f_* = 0.23 (*n*-hexane/ethyl acetate 6/1, *v*/*v*). IR (KBr) ν_max_, cm^−1^: 2984, 2928, 1703 (C=O), 1517, 1457, 1252, 1199, 763. ^1^H NMR (400 MHz, CDCl_3_) δ_H_ ppm: 1.42 (t, *J* = 7.1 Hz, 3H, CH_3_), 2.48 (dd, *J* = 4.4, 2.6 Hz, 1H, Ox CH_a_H_b_), 2.78 (t, *J* = 4.3 Hz, 1H, Ox CH_a_H_b_), 3.32–3.38 (m, 1H, Ox CH), 4.38 (q, *J* = 7.1 Hz, 2H, CH_2_CH_3_), 4.47 (dd, *J* = 15.2, 5.4 Hz, 1H, NCH_a_H_b_), 5.07 (dd, *J* = 15.2, 2.6 Hz, 1H, NCH_a_H_b_), 7.25–7.26 (m, 1H, 3-H), 7.28–7.30 (m, 1H, 6-H), 7.39–7.41 (m, 1H, 7-H), 7.62–7.63 (m, 1H, 4-H). ^13^C NMR (101 MHz, CDCl_3_) δ_C_ ppm: 14.5 (CH_3_), 45.3 (Ox CH_a_H_b_), 46.5 (NCH_a_H_b_), 51.6 (Ox CH), 61.1 (CH_2_CH_3_), 110.3 (C-3), 112.3 (C-7), 121.7 (C-4), 125.9 (C-6), 126.8 (C-5), 126.9 (C-3a), 128.8 (C-2), 138.3 (C-7a), 162.0 (COO). ^15^N NMR (40 MHz, CDCl_3_) δ_N_ ppm: –250.0 (N). HRMS (ESI) for C_14_H_15_ClNO_3_ ([M+H]^+^): calcd *m/z* 280.0735, found *m/z* 280.0733.

##### Ethyl 5-methyl-1-(oxiran-2-ylmethyl)-1*H*-indole-2-carboxylate **6d**

Purified by column chromatography on silica gel (*n*-hexane/ethyl acetate 8/1, *v*/*v*). White solid, mp 72–74 °C, procedure a −75% (96 mg). R*_f_* = 0.27 (*n*-hexane/ethyl acetate 6/1, *v*/*v*). IR (KBr) ν_max_, cm^−1^: 2976, 1706 (C=O), 1408, 1263, 1211, 1128, 1096, 763. ^1^H NMR (400 MHz, CDCl_3_) δ_H_ ppm: 1.41 (t, *J* = 7.1 Hz, 3H, CH_2_CH_3_), 2.44 (s, 3H, 5-CH_3_), 2.49 (dd, *J* = 4.4, 2.6 Hz, 1H, Ox CH_a_H_b_), 2.76 (t, *J* = 4.4 Hz, 1H, Ox CH_a_H_b_), 3.32–3.37 (m, 1H, Ox CH), 4.37 (q, *J* = 7.1 Hz, 2H, CH_2_CH_3_), 4.55 (dd, *J* = 15.1, 5.0 Hz, 1H, NCH_a_H_b_), 4.99 (dd, *J* = 15.1, 3.2 Hz, 1H, NCH_a_H_b_), 7.16–7.20 (m, 1H, 6-H), 7.22–7.27 (m, 1H, 3-H), 7.33–7.37 (m, 1H, 7-H), 7.42–7.48 (m, 1H, 4-H). ^13^C NMR (101 MHz, CDCl_3_) δ_H_ ppm: 14.5 (CH_2_CH_3_), 21.5 (5-CH_3_), 45.5 (Ox CH_a_H_b_), 46.2 (NCH_a_H_b_), 51.7 (Ox CH), 60.7 (CH_2_CH_3_), 110.59 (C-7), 110.62 (C-3), 121.9 (C-4), 126.3 (C-3a), 127.5 (C-6), 127.6 (C-2), 130.4 (C-5), 138.5 (C-7a), 162.3 (COO). ^15^N NMR (40 MHz, CDCl_3_) δ_N_ ppm: –251.8 (N). HRMS (ESI) for C_15_H_18_NO_3_ ([M+H]^+^): calcd *m/z* 260.1281, found *m/z* 260.1277.

##### Ethyl 3-methyl-1-(oxiran-2-ylmethyl)-1*H*-indole-2-carboxylate **6e**

Purified by column chromatography on silica gel (petroleum ether/ethyl acetate gradient from 9/1 to 8/1, *v*/*v*). Colorless liquid, procedure a −47% (60 mg). R*_f_* = 0.14 (petroleum ether/ethyl acetate 10/1, *v*/*v*). IR (KBr) ν_max_, cm^−1^: 1698 (C=O), 1273, 1250, 1208, 1129, 1111, 741. ^1^H NMR (500 MHz, CDCl_3_) δ_H_ ppm: 1.44 (t, *J* = 7.2 Hz, 3H, CH_2_CH_3_), 2.49 (dd, *J* = 4.9, 2.8 Hz, 1H, Ox CH_a_H_b_), 2.59 (s, 3H, 5-CH_3_), 2.74–2.77 (m, 1H, OxCH_a_H_b_), 3.33–3.37 (m, 1H, Ox CH), 4.42 (q, *J* = 7.1 Hz, 2H, CH_2_CH_3_), 4.50 (dd, *J* = 15.3, 5.2 Hz, 1H, NCH_a_H_b_), 4.94 (dd, *J* = 15.3, 3.4 Hz, NCH_a_H_b_), 7.13–7.17 (m, 1H, 5-H), 7.33–7.37 (m, 1H, 6-H), 7.39–7.43 (m, 1H, 7-H), 7.64–7.69 (m, 1H, 4-H). ^13^C NMR (125 MHz, CDCl_3_) δ_C_ ppm: 11.0 (3-CH_3_), 14.5 (CH_2_CH_3_),45.4 (Ox CH_a_H_b_), 46.4 (NCH_a_H_b_), 51.8 (Ox CH), 60.6 (CH_2_CH_3_), 110.6 (C-7), 120.2 (C-5), 120.8 (C-4), 121.5 (C_q_), 124.6 (C_q_), 125.7 (C-6), 127.4 (C_q_), 139.1 (C_q_), 163.2 (COO). ^15^N NMR (40 MHz, CDCl_3_) δ_N_ ppm: −254.7 (N). HRMS (ESI) for C_15_H_18_NO_3_ ([M+H]^+^): calcd *m/z* 260.1281, found *m/z* 260.1276.

##### Ethyl 1-(oxiran-2-ylmethyl)-1*H*-benzo[*d*]imidazole-2-carboxylate **6f**

Purified by column chromatography on silica gel (petroleum ether/ethyl acetate gradient from 1/1 to 2/3, *v*/*v*). White solid, mp 49–52 °C, procedure b −33% (86 mg). R*_f_* = 0.37 (petroleum ether/ethyl acetate 1/1, *v*/*v*). IR (KBr) ν_max_, cm^−1^: 2360, 2341, 1709 (C=O), 1494, 1457, 1410, 1338, 1283, 1202, 1138, 743. ^1^H NMR (500 MHz, DMSO-*d_6_*) δ_H_ ppm: 1.34 (t, *J* = 7.2 Hz, 3H, CH_3_), 2.42–2.45 (m, 1H, Ox CH_a_H_b_), 2.72 (t, *J* = 4.4 Hz, OxCH_a_H_b_), 3.33–3.38 m, 1H, Ox CH), 4.37 (q, *J* = 7.1 Hz, 2H, CH_2_CH_3_), 4.58 (dd, *J* = 15.1, 8.7 Hz, 1H, NCH_a_H_b_), 5.03 (dd, *J* = 15.3, 3.1 Hz, NCH_a_H_b_), 7.29–7.34 (m, 1H, 5-H), 7.37–7.42 (m, 1H, 6-H), 7.69–7.72 (m, 1H, 7-H), 7.74–7.78 (m, 1H, 4-H). ^13^C NMR (125 MHz, DMSO-*d_6_*) δ_C_ ppm: 14.5 (CH_3_), 45.2 (Ox CH_a_H_b_), 46.8 (NCH_a_H_b_), 50.9 (Ox CH), 62.2 (CH_2_CH_3_), 112.5 (C-7), 121.4 (C-4), 124.0 (C-5), 125.7 (C-6), 136.8 (C_q_), 141.5 (C_q_), 141.6 (C_q_), 160.1 (COO). ^15^N NMR (40 MHz, DMSO-*d_6_*) δ_N_ ppm: −229.8 (N). HRMS (ESI) for C_13_H_14_N_2_O_3_ ([M+H]^+^): calcd *m/z* 247.1077, found *m/z* 247.1071.

#### 3.2.5. Synthesis of 4-Hydroxy-2,3,4,5-tetrahydro-1*H*-[1,4]diazepino[1,2-*a*]indol-1-ones (**7a–f**) and 4-Hydroxy-2,3,4,5-tetrahydro-1*H*-benzo[4,5]imidazo[1,2-*a*][1,4]diazepin-1-one (**7g**)

**Procedure a.** Ethyl 1-(oxiran-2-ylmethyl)-1*H*-indole-2-carboxylate **6a–e** or ethyl 1-(oxiran-2-ylmethyl)-1*H*-benzo[*d*]imidazole-2-carboxylate **6f** (1 eq) was dissolved in 7M ammonia (100 eq) solution in methanol, sealed in a pressure tube and stirred at 70 °C for 18 h. After completion, the reaction mixture was cooled to 0 °C, quenched with water and extracted with ethyl acetate. The combined organic phases were washed with brine, dried over anhydrous Na_2_SO_4_, filtered, and concentrated under reduced pressure. The residue was purified by column chromatography.

**Procedure b.** To a solution of ethyl 1-(oxiran-2-ylmethyl)-1*H*-indole-2-carboxylate 7a (1eq) in methanol (5 M) benzylamine (3 eq) was added, the reaction mixture was sealed in a pressure tube and stirred at 70 °C for 18 h. After completion, the reaction mixture was poured into water and extracted with ethyl acetate. The combined organic phases were washed with brine, dried over anhydrous Na_2_SO_4_, filtered, and concentrated under reduced pressure. The residue was purified by column chromatography.

##### 4-Hydroxy-2,3,4,5-tetrahydro-1*H*-[1,4]diazepino[1,2-*a*]indol-1-one **7a**

Purified by column chromatography on silica gel (dichloromethane/methanol 100/5, *v*/*v*). White solid, decomp. 216 °C, procedure a −93% (82 mg). R*_f_* = 0.45 (dichloromethane/methanol 9/1, *v*/*v*). IR (KBr) ν_max_, cm^−1^: 3318, 3194, 3049, 2895, 1660 (C=O), 1643 (C=O), 1549, 1461, 1407, 740. ^1^H NMR (400 MHz, DMSO-*d_6_*) δ_H_ ppm: 2.83 (dt, *J* = 12.0, 5.6 Hz, 1H, 3-H_a_), 3.21 (dt, *J* = 11.3, 5.4 Hz, 1H, 3-H_b_), 4.18–4.28 (m, 2H, 5-H_a_, 4-H), 4.42 (dd, *J* = 13.9, 4.1 Hz, 1H, 5-H_b_), 5.37 (d, *J* = 3.8 Hz, 1H, OH), 6.93 (s, 1H, 11-H), 7.06–7.10 (m, 1H, 9-H), 7.25–7.29 (m, 1H, 8-H), 7.55–7.57 (m, 1H, 7-H), 7.62–7.64 (m, 1H, 10-H), 8.08–8.10 (m, 1H, NH). ^13^C NMR (101 MHz, DMSO-*d_6_*) δ_C_ ppm: 45.6 (C-3), 48.2 (C-5), 69.5 (C-4), 105.3 (C-11), 110.5 (C-7), 119.9 (C-9), 121.6 (C-10), 123.5 (C-8), 126.0 (C-10a), 134.7 (C-11a), 137.5 (C-6a), 165.5 (C-1). ^15^N NMR (40 MHz, DMSO-*d_6_*) δ_N_ ppm: –272.6 (N-2), –249.8 (N-6). HRMS (ESI) for C_12_H_13_N_2_O_2_ ([M+H]^+^): calcd *m/z* 217.0972, found *m/z* 217.0969.

##### 9-Fluoro-4-hydroxy-2,3,4,5-tetrahydro-1*H*-[1,4]diazepino[1,2-*a*]indol-1-one **7b**

Purified by column chromatography on silica gel (dichloromethane/methanol 100/5, *v*/*v*). White solid, decomp. 220 °C, procedure a −63% (56 mg). R*_f_* = 0.55 (dichloromethane/methanol 9/1, *v*/*v*). IR (KBr) ν_max_, cm^−1^: 3335, 3283, 3200, 3047, 1647 (C=O), 1548, 1408, 1203, 796. ^1^H NMR (400 MHz, DMSO-*d_6_*) δ_H_ ppm: 2.78–2.87 (m, 1H, 3-H_a_), 3.16–3.26 (m, 1H, 3-H_b_), 4.16–4.30 (m, 2H, 5-H_a_, 4-H), 4.43 (dd, *J* = 14.0, 4.2 Hz, 1H, 5-H_b_), 5.38 (d, *J* = 4.0 Hz, 1H, OH), 6.91 (s, 1H, 11-H), 7.08–7.19 (m, 1H, 8-H), 7.36–7.43 (m, 1H, 10-H), 7.56–7.63 (m, 1H, 7-H), 8.12–8.20 (m, 1H, NH). ^13^C NMR (101 MHz, DMSO-*d_6_*) δ_C_ ppm: 45.6 (C-3), 48.5 (C-5), 69.5 (C-4), 105.0 (d, *^4^J_CF_* = 5.1 Hz, C-11), 105.8 (d, *^2^J_CF_* = 23.3 Hz, C-10), 111.9 (d, *^3^J_CF_* = 9.8 Hz, C-7), 112.1 (d, *^2^J_CF_* = 26.6 Hz, C-8), 126.1 (d, *^3^J_CF_* = 10.6 Hz, C-10a), 134.2 (C-6a), 136.3 (C-11a), 157.2 (d, *J_CF_* = 233.1 Hz, C-9), 165.2 (C-1). ^15^N NMR (40 MHz, DMSO-*d_6_*) δ_N_ ppm: –271.8 (N-2), –250.1 (N-6). HRMS (ESI) for C_12_H_12_FN_2_O_2_ ([M+H]^+^): calcd *m/z* 235.0877, found *m/z* 235.0876.

##### 9-Chloro-4-hydroxy-2,3,4,5-tetrahydro-1*H*-[1,4]diazepino[1,2-*a*]indol-1-one **7c**

Purified by column chromatography on silica gel (dichloromethane/methanol 100/5, *v*/*v*). White solid, decomp. 210 °C, procedure a −84% (75 mg). R*_f_* = 0.52 (dichloromethane/methanol 9/1, *v*/*v*). IR (KBr) ν_max_, cm^−1^: 3325, 3275, 3191, 3046, 1660 (C=O), 1643 (C=O), 1545, 1408, 797. ^1^H NMR (400 MHz, DMSO-*d_6_*) δ_H_ ppm: 2.86–2.75 (m, 1H, 3-H_a_), 3.27–3.14 (m, 1H, 3-H_b_), 4.29–4.16 (m, 2H, 4-H, 5-H_a_), 4.43 (dd, *J* = 13.8, 3.9 Hz, 1H, 5-H_b_), 5.39 (d, *J* = 3.8 Hz, 1H, OH), 6.91 (s, 1H, 11-H), 7.24–7.31 (m, 1H, 8-H), 7.57–7.65 (m, 1H, 7-H), 7.68–7.73 (m, 1H, 10-H), 8.14–8.22 (m, 1H, NH). ^13^C NMR (101 MHz, DMSO-*d_6_*) δ_C_ ppm: 45.5 (C-3), 48.5 (C-5), 69.4 (C-4), 104.7 (C-11), 112.3 (7-C), 120.6 (C-10), 123.5 (C-8), 124.4 (C-9), 127.0 (C-10a), 135.9 (C-6a), 136.1 (C-11a), 165.1 (C-1). ^15^N NMR (40 MHz, DMSO-*d_6_*) δ_N_ ppm: –271.5 (N-2), –248.7 (N-6). HRMS (ESI) for C_12_H_12_ClN_2_O_2_ ([M+H]^+^): calcd *m/z* 251.0582, found *m/z* 251.0580.

##### 4-Hydroxy-9-methyl-2,3,4,5-tetrahydro-1*H*-[1,4]diazepino[1,2-*a*]indol-1-one **7d**

Purified by column chromatography on silica gel (dichloromethane/methanol 100/5, *v*/*v*). White solid, decomp. 225 °C, procedure a −86% (76 mg). R*_f_* = 0.66 (dichloromethane/methanol 9/1, *v*/*v*). IR (KBr) ν_max_, cm^−1^: 3319, 3194, 3046, 1659 (C=O), 1640 (C=O), 1552, 1463, 1407, 790. ^1^H NMR (400 MHz, DMSO-*d_6_*) δ_H_ ppm: 2.38 (s, 3H, CH_3_), 2.78–2.87 (m, 1H, 3-H_a_), 3.15–3.24 (m, 1H, 3-H_b_), 4.16 (dd, *J* = 14.3, 4.3 Hz, 1H, 5-H_a_), 4.20–4.27 (m, 1H, 4-H), 4.38 (dd, *J* = 14.2, 4.7 Hz, 1H, 5-H_b_), 5.35 (d, *J* = 4.1 Hz, 1H, OH), 6.83 (s, 1H, 11-H), 7.06–7.14 (m, 1H, 8-H), 7.37–7.42 (m, 1H, 10-H), 7.41–7.47 (m, 1H, 7-H), 8.02–8.09 (m, 1H, NH). ^13^C NMR (101 MHz, DMSO-*d_6_*) δ_C_ ppm: 21.0 (CH_3_), 45.7 (C-3), 48.3 (C-5), 69.6 (C-4), 104.8 (C-11), 110.2 (C-7), 120.9 (C-10), 125.3 (C-8), 126.2 (C-9), 128.5 (C-10a), 134.6 (C-11a), 136.0 (C-6a), 165.6 (C-1). ^15^N NMR (40 MHz, DMSO-*d_6_*) δ_N_ ppm: –273.1 (N-2), –250.9 (N-6). ^19^F NMR (376 MHz, DMSO-*d_6_*) δ_F_ ppm: –123.9 (F). HRMS (ESI) for C_13_H_15_N_2_O_2_ ([M+H]^+^): calcd *m/z* 231.1128, found *m/z* 231.1124.

##### 4-Hydroxy-11-methyl-2,3,4,5-tetrahydro-1*H*-[1,4]diazepino[1,2-*a*]indol-1-one **7e**

Purified by column chromatography on silica gel (petroleum ether/ethyl acetate/methanol gradient from 1/3/0 to 5/20/1, *v*/*v*/*v*). White solid, decomp. 218 °C, procedure a −66% (66 mg). R*_f_* = 0.42 (ethyl acetate/methanol 10/1, *v*/*v*). IR (KBr) ν_max_, cm^−1^: 3308, 1631 (C=O), 1556, 1461, 1418, 1383, 1362, 1341, 1329, 1259, 1066, 733. ^1^H NMR (500 MHz, DMSO-*d_6_*) δ_H_ ppm: 2.36 (s, 3H, CH_3_), 2.76 (dt, *J* = 14.4, 6.0 Hz, 1H, 3-H_a_), 3.13 (dt, *J* = 14.4, 5.8 Hz, 1H, 3-H_b_), 4.10 (dd, *J* = 14.2, 4.4 Hz, 1H, 5-H_a_), 4.12–4.19 (m, 1H, 4-H), 4.32 (dd, *J* = 14.4, 4.9 Hz, 1H, 5-H_b_), 5.30 (d, *J* = 4.0 Hz, 1H, OH), 7.02–7.10 (m, 1H, 9-H), 7.22–7.29 (m, 1H, 8-H), 7.47–7.52 (m, 1H, 7-H), 7.57–7.62 (m, 1H, 10-H), 7.97–8.02 (m, 1H, NH). ^13^C NMR (125 MHz, DMSO-*d_6_*) δ_C_ ppm: 9.6 (CH_3_), 46.1 (C-3), 48.6 (C-5), 69.4 (C-4), 110.6 (C-7), 115.5 (C_q_), 119.6 (C-9), 120.3 (C-10), 124.3 (C-8), 127.4 (C_q_), 130.3 (C_q_), 137.0 (C_q_), 166.3 (C-1). ^15^N NMR (40 MHz, DMSO-*d_6_*) δ_N_ ppm: −271.4 (N-2), −254.4 (N-6). HRMS (ESI) for C_13_H_15_N_2_O_2_ ([M+H]^+^): calcd *m/z* 231.1128, found *m/z* 231.1126.

##### 2-Benzyl-4-hydroxy-2,3,4,5-tetrahydro-1*H*-[1,4]diazepino[1,2-*a*]indol-1-one **7f**

Purified by column chromatography on silica gel (petroleum ether/ethyl acetate gradient from 7/1 to 1/6, *v*/*v*). White solid, mp 183–184 °C, procedure b −87% (142.1 mg). R*_f_* = 0.53 (petroleum ether/ethyl acetate 3/1, *v*/*v*). IR (KBr) ν_max_, cm^−1^: 3313 (OH), 2360, 2341, 1610 (C=O), 1541, 1421, 1079, 740. ^1^H NMR (400 MHz, DMSO-*d_6_*) δ_H_ ppm: 3.01 (dd, *J* = 14.8, 7.4 Hz, 1H, 3-H_a_), 3.31 (dd, *J* = 14.8, 5.2 Hz, 1H, 3-H_b_), 4.06–4.14 (m, 1H, 4-H), 4.23 (dd, *J* = 14.6, 3.8 Hz, 1H, 5-H_a_), 4.38 (dd, *J* = 14.6, 4.9 Hz, 1H, 5-H_b_), 4.46 (d, *J* = 14.6 Hz, 1H, 2-CH_a_), 5.00 (d, *J* = 14.6 Hz, 1H, 2-CH_b_), 5.40 (d, *J* = 4.0 Hz, 1H, OH), 6.99–7.01 (m, 1H, 11-H), 7.05–7.11 (m, 1H, 9-H), 7.23–7.35 (m, 2H, 8-H, CH_2_Ph 4-H), 7.36–7.41 (m, 4H, CH_2_Ph 2,3,5,6-H), 7.50–7.57 (m, 1H, 7-H), 7.61–7.67 (m, 1H, 10-H). ^13^C NMR (101 MHz, DMSO-*d_6_*) δ_C_ ppm: 48.1 (C-5), 50.4 (CH_2_Ph), 51.4 (C-3), 69.1 (C-4), 105.5 (C-11), 110.4 (C-7), 120.0 (C-9), 121.6 (C-10), 123.6 (C-8), 126.0 (C-10a), 127.4 (CH_2_Ph C-4), 128.2 (CH_2_Ph C-3,5), 128.7 (CH_2_Ph C-2,6), 134.3 (C-11a), 137.3 (C-6a), 137.9 (CH_2_Ph C-1), 163.9 (C-1). ^15^N NMR (40 MHz, DMSO-*d_6_*) δ_N_ ppm: –255.0 (N-6), -265.7 (N-2). HRMS (ESI) for C_19_H_19_N_2_O_2_ ([M+H]^+^): calcd *m/z* 307.1441, found *m/z* 307.1388.

##### 4-Hydroxy-2,3,4,5-tetrahydro-1*H*-benzo[4,5]imidazo[1,2-*a*][1,4]diazepin-1-one **7g**

Purified by column chromatography on silica gel (petroleum ether/ethyl acetate/methanol gradient from 1/5/0 to 1/12/1, *v*/*v*/*v*). White solid, decomp. 190 °C, procedure a −19% (18 mg). R*_f_* = 0.21 (ethyl acetate/methanol 9/1, *v*/*v*). IR (KBr) ν_max_, cm^−1^: 3379, 3220, 1676 (C=O), 1522, 1466, 1458, 1418, 1336, 1079, 741. ^1^H NMR (500 MHz, DMSO-*d_6_*) δ_H_ ppm: 2.75 (dt, *J* = 15.0 Hz, 6.1 Hz, 1H, 3-H_a_), 3.21 (dt, *J* = 14.7 Hz, 5.8 Hz, 1H, 3-H_b_), 4.22–4.31 (m, 2H, 5-H_a_, 4-H), 4.49 (dd, *J* = 14.1 Hz, 4.3 Hz, 1H, 5-H_b_), 5.35–5.55 (br s, 1H, OH), 7.23–7.28 (m, 1H, 9-H), 7.31–7.37 (m, 1H, 8-H), 7.63–7.73 (m, 2H, 7-H, 10-H), 8.56–8.63 (m, 1H, NH). ^13^C NMR (125 MHz, DMSO-*d_6_*) δ_C_ ppm: 45.4 (C-3), 48.2 (C-5), 69.9 (C-4), 111.5 (C-7), 120.7 (C-10), 123.1 (C-9), 124.6 (C-8), 135.2 (C_q_), 142.0 (C_q_), 147.4 (C_q_), 163.2 (C-1). HRMS (ESI) for C_11_H_12_N_3_O_2_ ([M+H]^+^): calcd *m/z* 218.0924, found *m/z* 218.0918.

#### 3.2.6. Synthesis of *O*-Alkylated 5-Substituted 7-Hydroxy-2-phenyl-5,6,7,8-tetrahydro-4*H*-pyrazolo[1,5-*a*][1,4]diazepin-4-ones (**8a–f**) and 2-Benzyl-4-hydroxy-2,3,4,5-tetrahydro-1*H*-[1,4]diazepino[1,2-*a*]indol-1-one (**9a,b**)

Pyrazolo-diazepinone **4i**,**n**,**t** or indole-diazepinone **7f** (1 eq) was dissolved in dimethyl formamide (0.1 M) and NaH (1.5 eq; 60% dispersion in mineral oil) was added followed by appropriate alkylating agent (1.5 eq). The reaction mixture was stirred at 25–50 °C for 5–8 h. Upon completion, the mixture was concentrated to approximately 1/3 volume, diluted with ethyl acetate, and washed with brine. Organic layer was dried over anhydrous Na_2_SO_4_, filtered, and concentrated under reduced pressure. The residue was purified by column chromatography.

##### 7-Methoxy-5-(2-methoxyethyl)-2-phenyl-5,6,7,8-tetrahydro-4*H*-pyrazolo[1,5-*a*][1,4]diazepin-4-one **8a**

Purified by column chromatography on silica gel (*n*-hexane/ethyl acetate 1/1, *v*/*v*). White solid, mp 88–89 °C, 77% (104 mg). R*_f_* = 0.41 (*n*-hexane/ethyl acetate 1/4, *v*/*v*). IR (KBr) ν_max_, cm^−1^: 3120, 2933, 2890, 2828, 1638 (C=O), 1470, 1354, 1016, 771, 700. ^1^H NMR (400 MHz, DMSO-*d_6_*) δ_H_ ppm: 3.23 (dd, *J* = 15.2, 7.6 Hz, 1H, 5-CH_a_), 3.29 (s, 3H, CH_2_OCH_3_), 3.37 (s, 3H, 7-OCH_3_), 3.39–3.47 (m, 1H, 6-H_a_), 3.51–3.62 (m, 3H, 5-CH_b_, CH_2_O), 3.90 (dt, *J* = 13.6, 5.2 Hz, 1H, 6-H_b_), 4.05–4.12 (m, 1H, 7-H), 4.46 (qd, *J* = 14.7, 4.3 Hz, 2H, 8-H_a_H_b_), 7.18 (s, 1H, 3-H), 7.29–7.35 (m, 1H, Ph 4-H), 7.38–7.45 (m, 2H, Ph 3,5-H), 7.8 –7.87 (m, 2H, Ph 2,6-H). ^13^C NMR (101 MHz, DMSO-*d_6_*) δ_C_ ppm: 46.6 (C-6), 50.0 (5-CH_2_), 52.1 (C-8), 56.3 (7-OCH_3_), 58.0 (CH_2_OCH_3_), 69.8 (CH_2_O), 79.2 (C-7), 105.5 (C-3), 125.2 (Ph C-2,6), 127.8 (Ph C-4), 128.7 (Ph C-3,5), 132.4 (Ph C-1), 139.3 (C-3a), 149.1 (C-2), 161.6 (C-4). ^15^N NMR (40 MHz, DMSO-*d_6_*) δ_N_ ppm: −266.0 (N-5), −176.2 (N-9), −74.1 (N-1). HRMS (ESI) for C_17_H_21_N_3_NaO_3_ ([M+Na]^+^): calcd *m/z* 338.1475, found *m/z* 338.1475.

##### 5-Allyl-7-methoxy-2-phenyl-5,6,7,8-tetrahydro-4*H*-pyrazolo[1,5-*a*][1,4]diazepin-4-one **8b**

Purified by column chromatography on silica gel (*n*-hexane/ethyl acetate 3.5/1, *v*/*v*). White solid, mp 89–90 °C, 84% (78 mg). R*_f_* = 0.47 (*n*-hexane/ethyl acetate 1/1, *v*/*v*). IR (KBr) ν_max_, cm^−1^: 2984, 2929, 2826, 1657 (C=O), 1461, 1243, 1206, 1089, 770, 697. ^1^H NMR (400 MHz, DMSO-*d_6_*) δ_H_ ppm: 3.19 (dd, *J* = 15.2, 6.9 Hz, 1H, 6-H_a_), 3.36 (s, 3H, CH_3_), 3.46 (dd, *J* = 15.2, 5.0 Hz, 1H, 6-H_b_), 3.97 (dd, *J* = 15.1, 6.2 Hz, 1H, 5-CH_a_), 4.09 (dt, *J* = 10.4, 5.1 Hz, 1H, 7-H), 4.23 (dd, *J* = 15.1, 5.5 Hz, 1H, 5-CH_b_), 4.40 (dd, *J* = 14.7, 4.0 Hz, 1H, 8-H_a_), 4.58 (dd, *J* = 14.7, 5.4 Hz, 1H, 8-H_b_), 5.18–5.31 (m, 2H, CH_2_-CH=CH_2_), 5.81–5.92 (m, 1H, CH_2_-CH=CH_2_), 7.20 (s, 1H, 3-H), 7.29–7.36 (m, 1H, Ph 4-H), 7.37–7.45 (m, 2H, Ph 3,5-H), 7.81–7.88 (m, 2H, Ph 2,6-H). ^13^C NMR (101 MHz, DMSO-*d_6_*) δ_C_ ppm: 48.4 (C-6), 49.6 (5-CH_2_), 52.3 (C-8), 56.3 (CH_3_), 78.9 (C-7), 105.8 (C-3), 117.9 (CH_2_-CH=CH_2_), 125.1 (Ph C-2,6), 127.9 (Ph C-4), 128.7 (Ph C-3,5), 132.4 (Ph C-1), 133.4 (CH_2_-CH=CH_2_), 139.1 (C-3a), 149.1 (C-2), 161.3 (C-4). ^15^N NMR (40 MHz, DMSO-*d_6_*) δ_N_ ppm: −176.0 (N-9), −73.9 (N-1). HRMS (ESI) for C_17_H_19_N_3_NaO_2_ ([M+Na]^+^): calcd *m/z* 320.1369, found *m/z* 320.1370.

##### 5-Benzyl-7-methoxy-2-phenyl-5,6,7,8-tetrahydro-4*H*-pyrazolo[1,5-*a*][1,4]diazepin-4-one **8c**

Purified by column chromatography on silica gel (*n*-hexane/ethyl acetate 7/2, *v*/*v*). Pale yellow solid, mp 176–177 °C, 70% (57 mg). R*_f_* = 0.41 (*n*-hexane/ethyl acetate 3/2, *v*/*v*). IR (KBr) ν_max_, cm^−1^: 3146, 2988, 2928, 2871, 1651 (C=O), 1449, 1241, 1101, 764, 695. ^1^H NMR (400 MHz, DMSO-*d_6_*) δ_H_ ppm: 3.19 (dd, *J* = 15.2, 7.1 Hz, 1H, 6-H_a_), 3.28 (s, 3H, CH_3_), 3.48 (dd, *J* = 15.2, 5.0 Hz, 1H, 6-H_b_), 3.86–3.93 (m, 1H, 7-H), 4.42 (dd, *J* = 14.8, 3.8 Hz, 1H, 8-H_a_), 4.48–4.59 (m, 2H, 8-H_b_, 5-CH_a_), 4.88 (d, *J* = 14.7 Hz, 1H, 5-CH_b_), 7.26 (s, 1H, 3-H), 7.30–7.35 (m, 2H, CPh 4-H, CH_2_Ph 4-H), 7.36–7.44 (m, 6H, CPh 3,5-H, CH_2_Ph 2,3,5,6-H), 7.82–7.87 (m, 2H, CPh 2,6-H). ^13^C NMR (101 MHz, DMSO-*d_6_*) δ_C_ ppm: 48.6 (C-6), 50.4 (5-CH_2_), 52.3 (C-8), 56.3 (CH_3_), 78.8 (C-7), 106.0 (C-3), 125.1 (CPh C-2,6), 127.5 (CH_2_Ph C-4), 127.9 (CPh C-4), 128.1 (CH_2_Ph C-2,6), 128.6 (CH_2_Ph C-3,5), 128.7 (CPh C-3,5), 132.4 (CPh C-1), 137.4 (CH_2_Ph C-1), 139.0 (C-3a), 149.2 (C-2), 161.7 (C-4). ^15^N NMR (40 MHz, DMSO-*d_6_*) δ_N_ ppm: −261.2 (N-5), −176.7 (N-9), −74.1 (N-1). HRMS (ESI) for C_21_H_21_N_3_NaO_2_ ([M+Na]^+^): calcd *m/z* 370.1526, found *m/z* 370.1526.

##### 7-Ethoxy-5-(2-methoxyethyl)-2-phenyl-5,6,7,8-tetrahydro-4*H*-pyrazolo[1,5-*a*][1,4]diazepin-4-one **8d**

Purified by column chromatography on silica gel (*n*-hexane/ethyl acetate/methanol 1/1/0.05, *v*/*v*). Yellowish resin, 75% (118 mg). R*_f_* = 0.54 (*n*-hexane/ethyl acetate 1/4, *v*/*v*). IR (KBr) ν_max_, cm^−1^: 2977, 2931, 2893, 1647 (C=O), 1461, 1440, 1104, 1077, 769, 695. ^1^H NMR (400 MHz, DMSO-*d_6_*) δ_H_ ppm: 1.14 (t, *J* = 7.0 Hz, 3H, 7-OCH_2_CH_3_), 3.24 (dd, *J* = 15.3, 7.4 Hz, 1H, 5-CH_a_), 3.29 (s, 3H, CH_2_OCH_3_), 3.41–3.49 (m, 1H, 6-H_a_), 3.51–3.68 (m, 5H, 5-CH_b_, CH_2_OCH_3_, 7-OCH_2_), 3.88 (dt, *J* = 13.5, 5.0 Hz, 1H, 6-H_b_), 4.15–4.22 (m, 1H, 7-H), 4.44 (ddd, *J* = 18.2, 14.6, 4.4 Hz, 2H, 8-H_a_H_b_), 7.18 (s, 1H, 3-H), 7.29–7.36 (m, 1H, Ph 4-H), 7.38–7.45 (m, 2H, Ph 3,5-H), 7.81–7.87 (m, 2H, Ph 2,6-H). ^13^C NMR (101 MHz, DMSO-*d_6_*) δ_C_ ppm: 15.3 (7-OCH_2_CH_3_), 46.6 (C-6), 50.4 (5-CH_2_), 52.7 (C-8), 58.0 (CH_2_OCH_3_), 63.9 (7-OCH_2_), 69.8 (CH_2_OCH_3_), 77.4 (C-7), 105.5 (C-3), 125.1 (Ph C-2,6), 127.8 (Ph C-4), 128.7 (Ph C-3,5), 132.4 (Ph C-1), 139.3 (C-3a), 149.1 (C-2), 161.5 (C-4). ^15^N NMR (40 MHz, DMSO-*d_6_*) δ_N_ ppm: −265.2 (N-5), −175.8 (N-9), −74.2 (N-1). HRMS (ESI) for C_18_H_23_N_3_NaO_3_ ([M+Na]^+^): calcd *m/z* 352.1632, found *m/z* 352.1632.

##### 5-Allyl-7-ethoxy-2-phenyl-5,6,7,8-tetrahydro-4*H*-pyrazolo[1,5-*a*][1,4]diazepin-4-one **8e**

Purified by column chromatography on silica gel (*n*-hexane/ethyl acetate 7/2, *v*/*v*). Pale yellow solid, mp 113–144 °C, 77% (116 mg). R*_f_* = 0.59 (*n*-hexane/ethyl acetate 1/1, *v*/*v*). IR (KBr) ν_max_, cm^−1^: 3118, 2980, 1645 (C=O), 1460, 1438, 1396, 1248, 1101, 770, 696. ^1^H NMR (400 MHz, DMSO-*d_6_*) δ_H_ ppm: 1.13 (t, *J* = 6.9 Hz, 3H, CH_3_), 3.20 (dd, *J* = 15.1, 6.8 Hz, 1H, 6-H_a_), 3.44 (dd, *J* = 15.1, 4.7 Hz, 1H, 6-H_b_), 3.50–3.65 (m, 2H, OCH_2_), 3.98 (dd, *J* = 15.1, 6.0 Hz, 1H, 5-CH_a_), 4.15–4.27 (m, 2H, 5-CH_b_, 7-H), 4.37 (dd, *J* = 14.6, 3.8 Hz, 1H, 8-H_a_), 4.58 (dd, *J* = 14.6, 5.4 Hz, 1H, 8-H_b_), 5.18–5.31 (m, 2H, CH_2_-CH=CH_2_), 5.81–5.93 (m, 1H, CH_2_-CH=CH_2_), 7.20 (s, 1H, 3-H), 7.29–7.36 (m, 1H, Ph 4-H), 7.38–7.45 (m, 2H, Ph 3,5-H), 7.81–7.87 (m, 2H, Ph 2,6-H). ^13^C NMR (101 MHz, DMSO-*d_6_*) δ_C_ ppm: 15.3 (CH_3_), 48.8 (C-6), 49.6 (5-CH_2_), 52.9 (C-8), 63.9 (OCH_2_), 77.1 (C-7), 105.8 (C-3), 117.8 (CH_2_-CH=CH_2_), 125.1 (Ph C-2,6), 127.9 (Ph C-4), 128.7 (Ph C-3,5), 132.4 (Ph C-1), 133.4 (CH_2_-CH=CH_2_), 139.1 (C-3a), 149.1 (C-2), 161.3 (C-4). ^15^N NMR (40 MHz, DMSO-*d_6_*) δ_N_ ppm: −265.6 (N-5), −176.4 (N-9), −73.9 (N-1). HRMS (ESI) for C_18_H_21_N_3_NaO_2_ ([M+Na]^+^): calcd *m/z* 334.1526, found *m/z* 334.1526.

##### 5-Benzyl-7-ethoxy-2-phenyl-5,6,7,8-tetrahydro-4*H*-pyrazolo[1,5-*a*][1,4]diazepin-4-one **8f**

Purified by column chromatography on silica gel (*n*-hexane/ethyl acetate 3.5/1, *v*/*v*). Pale yellow solid, mp 115–116 °C, 72% (92 mg). R*_f_* = 0.55 (*n*-hexane/ethyl acetate 3/2, *v*/*v*). IR (KBr) ν_max_, cm^−1^: 3121, 2982, 2871, 1658 (C=O), 1460 and 1440 (doublet), 1239, 1105, 771, 693. ^1^H NMR (400 MHz, DMSO-*d_6_*) δ_H_ ppm: 1.06 (t, *J* = 7.0 Hz, 3H, CH_3_), 3.20 (dd, *J* = 15.2, 7.1 Hz, 1H, 6-H_a_), 3.39–3.58 (m, 3H, 6-H_b_, OCH_2_), 3.95–4.02 (m, 1H, 7-H), 4.38 (dd, *J* = 14.7, 3.8 Hz, 1H, 8-H_a_), 4.48–4.59 (m, 2H, 8-H_b_, 5-CH_a_), 4.88 (d, *J* = 14.7 Hz, 1H, 5-CH_b_), 7.26 (s, 1H, 3-H), 7.29–7.35 (m, 2H, CPh 4-H, CH_2_Ph 4-H), 7.36–7.44 (m, 6H, CPh 3,5-H, CH_2_Ph 2,3,5,6-H), 7.81–7.88 (m, 2H, CPh 2,6-H). ^13^C NMR (101 MHz, DMSO-*d_6_*) δ_C_ ppm: 15.2 (CH_3_), 49.0 (C-6), 50.4 (5-CH_2_), 52.9 (C-8), 63.8 (OCH_2_), 77.0 (C-7), 106.0 (C-3), 125.1 (CPh C-2,6), 127.4 (CH_2_Ph C-4), 127.9 (CPh C-4), 128.1 (CH_2_Ph C-2,6), 128.6 (CH_2_Ph C-3,5), 128.7 (CPh C-3,5), 132.4 (CPh C-1), 137.4 (CH_2_Ph C-1), 149.2 (C-2), 161.7 (C-4). ^15^N NMR (40 MHz, DMSO-*d_6_*) δ_N_ ppm: −261.6 (N-5), −176.1 (N-9), −73.5 (N-1). HRMS (ESI) for C_22_H_23_N_3_NaO_2_ ([M+Na]^+^): calcd *m/z* 384.1682, found *m/z* 384.1682.

##### 2-Benzyl-4-methoxy-2,3,4,5-tetrahydro-1*H*-[1,4]diazepino[1,2-*a*]indol-1-one **9a**

Purified by column chromatography on silica gel (petroleum ether/ethyl acetate gradient from 5/1 to 2/1, *v*/*v*). White solid, mp 97–98 °C, 92% (50 mg). R*_f_* = 0.63 (petroleum ether/ethyl acetate 2/1, *v*/*v*). IR (KBr) ν_max_, cm^−1^: 1642 (C=O), 1541, 1457, 1421, 1099, 741. ^1^H NMR (400 MHz, CDCl_3_) δ_H_ ppm: 3.31–3.37 (m, 5H, CH_3_, 3-H_a_H_b_), 3.68 (p, *J* = 5.2 Hz, 1H, 4-H), 4.27 (dd, *J* = 14.7, 5.2 Hz, 1H, 5-H_a_), 4.33–4.41 (m, 2H, 2-CH_a_, 5-H_b_), 5.26 (d, *J* = 14.6 Hz, 1H, 2-CH_b_), 7.11–7.16 (m, 1H, 9-H), 7.19 (s, 1H, 11-H), 7.28–7.41 (m, 7H, 7-H, 8-H, CH_2_Ph 2,3,4,5,6-H), 7.66–7.71 (m, 1H, 10-H). ^13^C NMR (101 MHz, CDCl_3_) δ_C_ ppm: 45.1 (C-5), 48.5 (C-3), 51.5 (2-CH_2_), 57.0 (CH_3_), 79.7 (C-4), 107.5 (C-11), 109.4 (C-7), 120.5 (C-9), 122.5 (C-10), 124.4 (C-8), 126.9 (C-10a), 127.9 (CH_2_Ph C-4), 128.7 (CH_2_Ph C-3,5), 128.9 (CH_2_Ph C-2,6), 134.0 (C-11a), 137.4 (C-6a), 137.6 (CH_2_Ph C-1), 165.0 (C=O). ^15^N NMR (40 MHz, CDCl_3_): δ_N_ ppm: −259.5 (N-6), −267.8 (N-2). HRMS (ESI) for C_20_H_21_N_2_O_2_ ([M+H]^+^): calcd *m/z* 321.1598, found *m/z* 321.1596.

##### 2-Benzyl-4-ethoxy-2,3,4,5-tetrahydro-1*H*-[1,4]diazepino[1,2-*a*]indol-1-one **9b**

Purified by column chromatography on silica gel (petroleum ether/ethyl acetate gradient from 5/1 to 4/1, *v*/*v*). Colorless resin, 73% (45 mg). R*_f_* = 0.57 (petroleum ether/ethyl acetate 2/1, *v*/*v*). IR (KBr) ν_max_, cm^−1^: 2360, 2341, 1641 (C=O), 1540, 1417, 1100, 741. ^1^H NMR (400 MHz, CDCl_3_) δ_H_ ppm: 1.14–1.20 (m, 3H, CH_3_), 3.29–3.35 (m, 2H, 3-H_a_H_b_), 3.43–3.59 (m, 2H, OCH_2_), 3.73– 3.81 (m, 1H, 4-H), 4.23–4.31 (m, 1H, 5-H_a_), 4.33–4.42 (m, 2H, 2-CH_a_, 5-H_b_), 5.22–5.30 (m, 1H, 2-CH_b_), 7.10–7.21 (m, 2H, 9-H, 11-H), 7.27–7.41 (m, 7H, 7-H, 8-H, CH_2_Ph 2,3,4,5,6-H), 7.66–7.72 (m, 1H, 10-H). ^13^C NMR (101 MHz, CDCl_3_) δ_C_ ppm: 15.5 (CH_3_). 45.5 (C-5), 49.0 (C-3), 51.5 (2-CH_2_), 64.8 (OCH_2_), 77.9 (C-4), 107.4 (C-11), 109.4 (C-7), 120.4 (C-9), 122.5 (C-10), 124.3 (C-8), 126.9 (C-10a), 127.90 (CH_2_Ph C-4), 128.7 (CH_2_Ph C-3,5), 128.9 (CH_2_Ph C-2,6), 134.1 (C-11a), 137.5 (C-6a), 137.6 (CH_2_Ph C-1), 165.1 (C-1). ^15^N NMR (40 MHz, CDCl_3_): δ_N_ ppm: −259.8 (N-6), −268.6 (N-2). HRMS (ESI) for C_21_H_23_N_2_O_2_ ([M+H]^+^): calcd *m/z* 335,1781, found *m/z* 335.1798.

## 4. Conclusions

To summarize, a regioselective strategy was developed for synthesizing ethyl 1-(oxiran-2-ylmethyl)-3-aryl-1*H*-pyrazole-5-carboxylates from easily accessible 3(5)-aryl-1*H*-pyrazole-5(3)-carboxylates. Regioselective alkylation was achieved via optimization using different bases, reaction media and temperatures. Established conditions were applied to the synthesis of novel pyrazolo[1,5-*a*][1,4]diazepin-4-one compound series via ring-opening of the oxirane with amines, and direct cyclisation sequence. Furthermore, the synthetic strategy was further applied to investigate the reactivity of ethyl 1*H*-indole-2-carboxylate and ethyl benzo[*d*]imidazole-2-carboxylate scaffolds which led to the formation of additional fused tetrahydro[1,4]diazepino[1,2-*a*]indol-1-one and tetrahydro-1*H*-benzo[4,5]imidazo[1,2-*a*][1,4]diazepin-1-one derivatives. The structures of all synthesized compounds were confirmed by detailed NMR spectroscopy and HRMS investigations.

## Figures and Tables

**Table 1:** Optimization of reaction conditions to access **2a**.

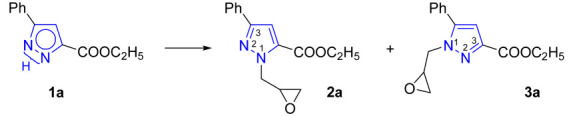							
Entry	2-(Chloromethyl)oxirane	Base	Solvent	Temp.	Time	Yield	
						2a	3a
1	15 eq	1.5 eq K_2_CO_3_	neat	70 °C	16 h	24%	-
2	10 eq	1.1 eq Cs_2_CO_3_	neat	40 °C	5 h	30%	20%
3	10 eq	1.3 eq NaH	neat	40 °C	5 h	-	50%
4 *^a^*	11.5 eq	2.0 eq KOH	neat	40 °C	6 h	7%	33%
5	5 eq	3.0 eq Cs_2_CO_3_	DMF	50 °C	24 h	49%	6%
**6**	**1.5 eq**	**1.5 eq NaH**	**DMF**	**40 °C**	**5 h**	**50%**	**-**
7	2.6 eq	1.4 eq Cs_2_CO_3_	ACN	reflux	5 h	45%	-

*^a^* TBAB was used as a phase transfer catalyst.

**Table 2:** Optimization of reaction conditions between **5a** and 2-(chloromethyl)oxirane.

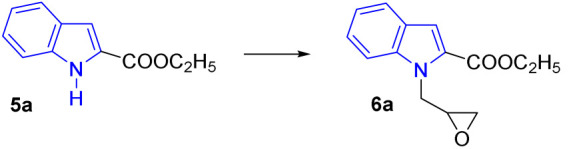						
Entry	2-(Chloromethyl)oxirane	Base	Solvent	Temp.	Time	Yield
1	1.5 eq	1.0 eq NaH	DMF	40 °C	5 h	54%
2	1.5 eq	1.0 eq NaH	DMF	60 °C	4 h	48%
3	1.2 eq	2.0 eq K_2_CO_3_	DMF	90 °C	4 h	40%
4	1.5 eq	2.0 eq K_2_CO_3_	DMF	90 °C	4 h	63%
5	1.5 eq	1.0 eq NaH	DMSO	40 °C	6 h	59%
6	1.5 eq	3.0 eq KOH	DMF	40 °C	1 h	73%

## Data Availability

The data that support the findings of this study are available upon request.

## Supplementary Materials

The following supporting information, containing ^1^H, ^13^C, ^1^H-^15^N-HMBC, ^19^F NMR and HRMS data, can be downloaded at: https://www.mdpi.com/article/10.3390/molecules27248666/s1.

## Sample Availability

Not available.
