# Supplementary material for: Convenient Synthesis of N-Heterocycle-Fused Tetrahydro-1,4-diazepinones
Source: Molecules. 2022 Dec 7;27(24):8666. doi: 10.3390/molecules27248666 (PMC9783606; doi:10.3390/molecules27248666)
Supplement: Supplementary file 1 [file molecules-27-08666-s001.zip › molecules-2067815-supplementary.pdf]

Supporting Information

Convenient Synthesis of *N*-Heterocycle-Fused Tetrahydro-1,4-Diazepinones

Karolina Dzedulionytė [1], Melita Veikšaitė [1], Vít Morávek [2], Vida Malinauskienė [1], Greta Račkauskienė [1,3], Algirdas Šačkus [1,3], Asta Žukauskaitė [1,2,*], and Eglė Arbačiauskienė [1,*]

[1] Department of Organic Chemistry, Kaunas University of Technology, Radvilėnų pl. 19A, LT-50254 Kaunas, Lithuania; karolina.dzedulionyte@ktu.lt (K.D.); melita.veiksaite@ktu.edu (M.V); vida.malinauskiene@ktu.lt (V.M.); greta.ragaite@ktu.lt (G.R.); algirdas.sackus@ktu.lt (A.Š.)
[2] Department of Chemical Biology, Palacký University, Šlechtitelů 27, CZ-78371 Olomouc, Czech Republic; vit.moravek01@upol.cz (V.M.)
[3] Institute of Synthetic Chemistry, Kaunas University of Technology, K. Baršausko g. 59, LT-51423 Kaunas, Lithuania
* Correspondence: asta.zukauskaite@upol.cz (A.Ž.); egle.arbaciauskiene@ktu.lt (E.A.)

Table of Contents

1. Data of ethyl 1-(oxiran-2-ylmethyl)-1*H*-pyrazole-3(5)-carboxylates (**2a–h**, **3a**,**f–h**) 2

2. Data of 7-hydroxy-5,6,7,8-tetrahydro-4*H*-pyrazolo[1,5-*a*][1,4]diazepin-4-ones (**4a–x**) 27

3. Data of ethyl 1-(oxiran-2-ylmethyl)-1*H*-indole-2-carboxylates (**6a–e**) and ethyl 1-(oxiran-2-ylmethyl)-1*H*-benzo[*d*]imidazole-2-carboxylate (**6f**) 75

4. Data of 4-hydroxy-2,3,4,5-tetrahydro-1*H*-[1,4]diazepino[1,2-*a*]indol-1-ones (**7a–f**) and 4-hydroxy-2,3,4,5-tetrahydro-1*H*-benzo[4,5]imidazo[1,2-*a*][1,4]diazepin-1-one (**7g**) 88

5. Data of *O*-alkylated 5-substituted 7-hydroxy-2-phenyl-5,6,7,8-tetrahydro-4*H*-pyrazolo[1,5-*a*][1,4]diazepin-4-ones (8a–f) and 2-benzyl-4-hydroxy-2,3,4,5-tetrahydro-1*H*-[1,4]diazepino[1,2-*a*]indol-1-one (**9a**,**b**) 102

1. Data of ethyl 1-(oxiran-2-ylmethyl)-1*H*-pyrazole-3(5)-carboxylates (2a–h, 3a,f–h)

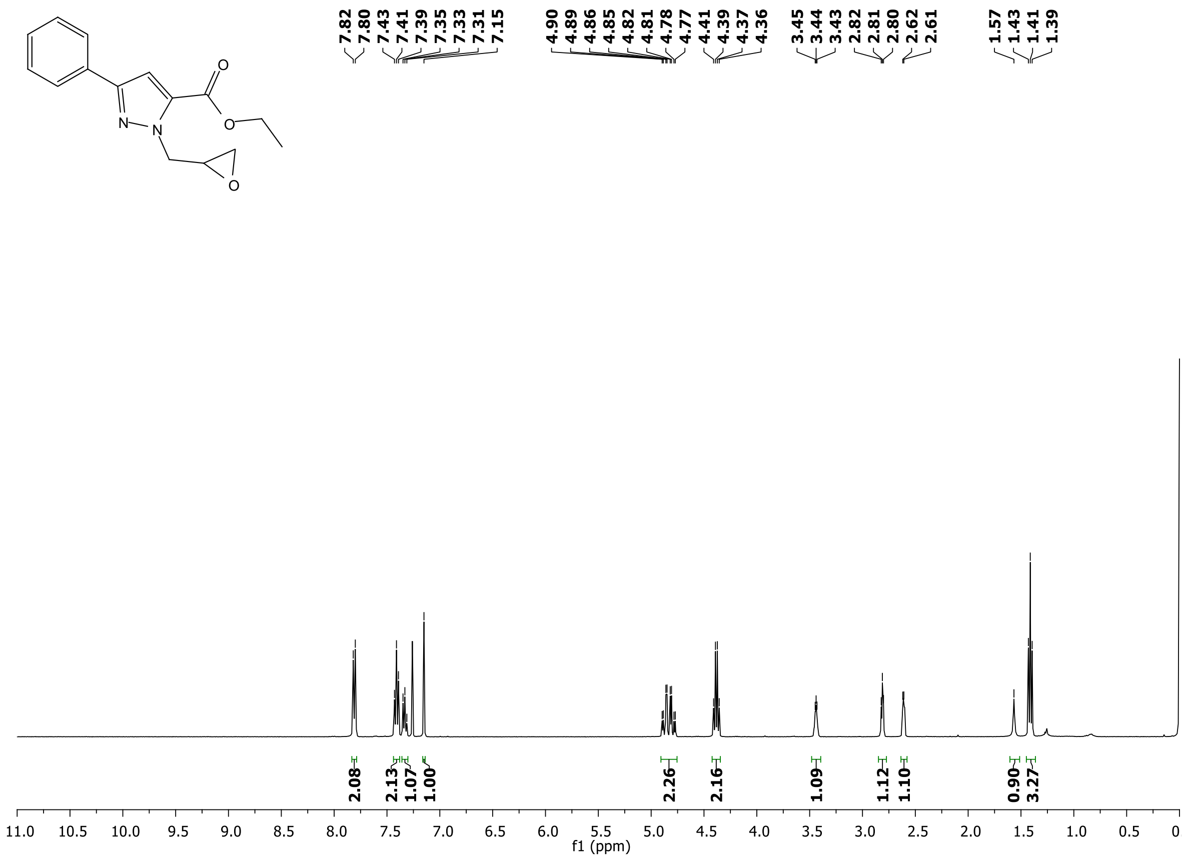

Figure S1. ^{1}H NMR spectrum (400 MHz, $CDCl_3$) of ethyl 1-(oxiran-2-ylmethyl)-3-phenyl-1*H*-pyrazole-5-carboxylate (**2a**).

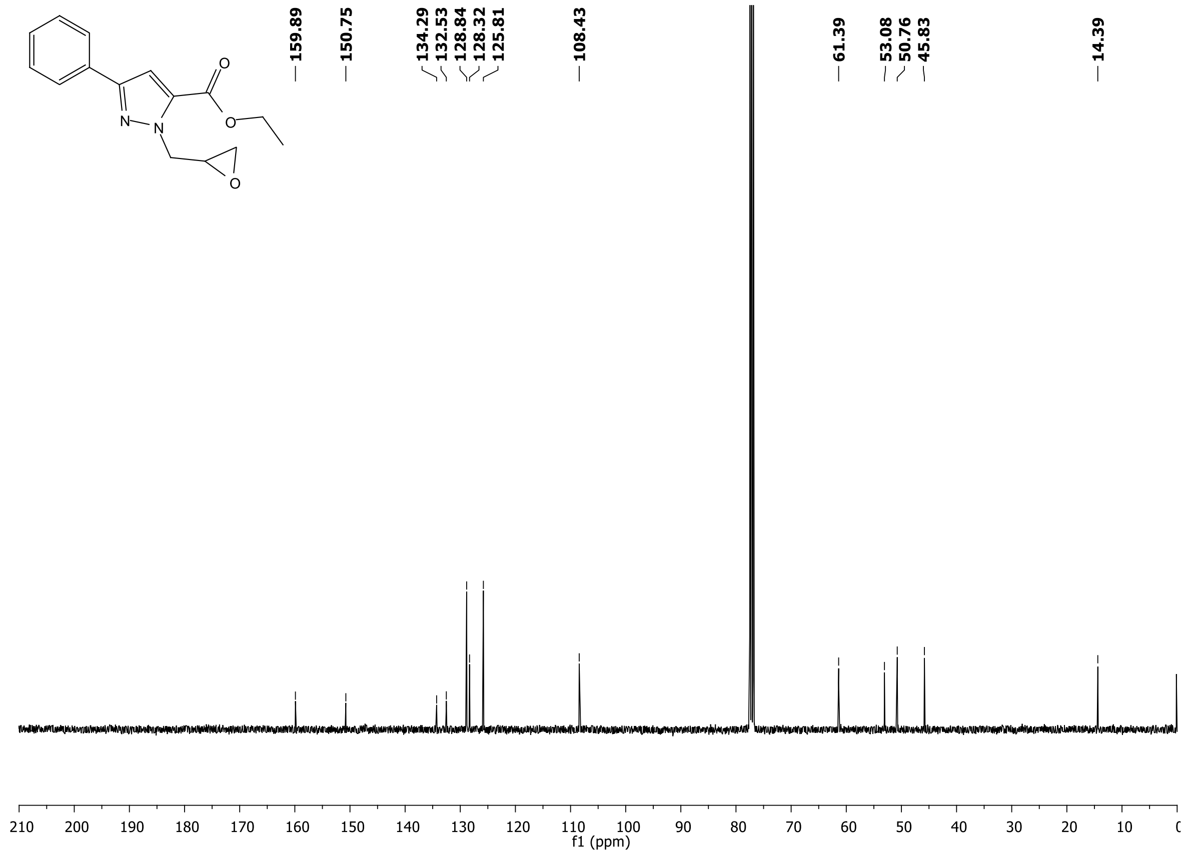

Figure S2. ^{13}C NMR spectrum (101 MHz, $CDCl_3$) of ethyl 1-(oxiran-2-ylmethyl)-3-phenyl-1*H*-pyrazole-5-carboxylate (**2a**).

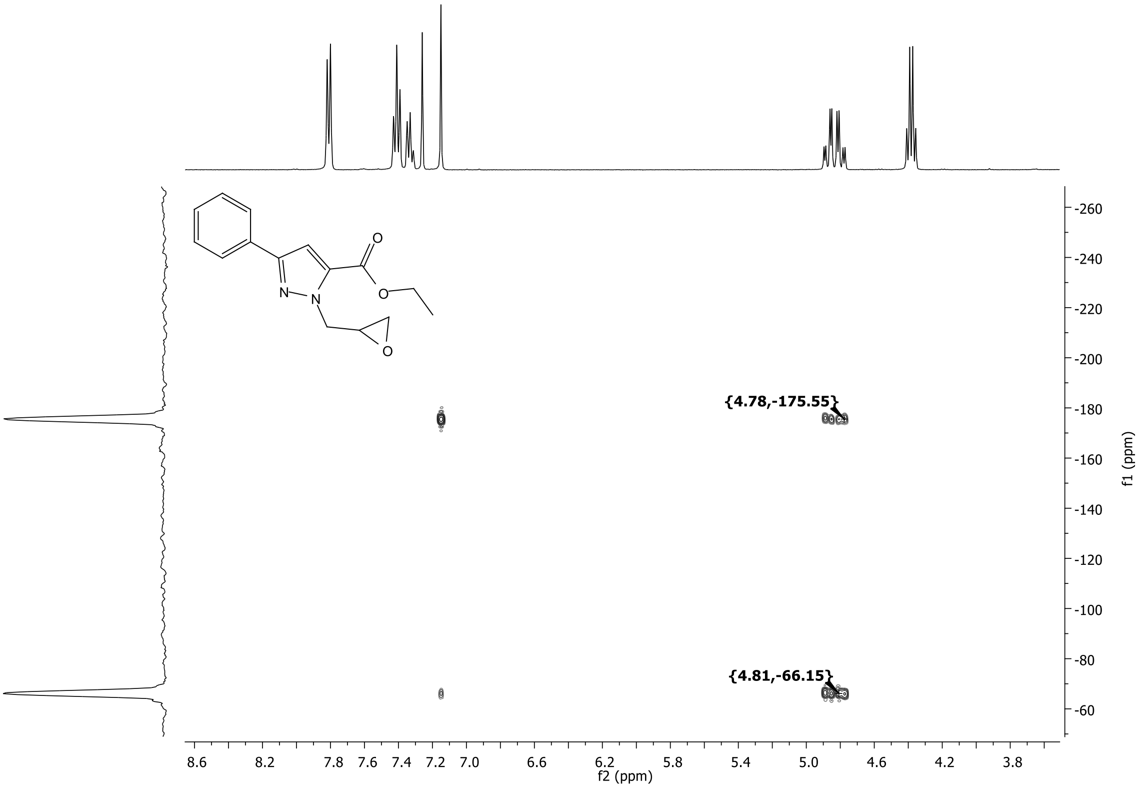

Figure S3. $^{1}H,^{15}N$-HMBC spectrum (40 MHz, $CDCl_3$) of ethyl 1-(oxiran-2-ylmethyl)-3-phenyl-1*H*-pyrazole-5-carboxylate (**2a**).

Compound Spectrum SmartFormula Report

Analysis Info

Analysis Name	D:\Data\KDD-088-1.d	Acquisition Date	6/15/2021 3:41:12 PM
Method	DirectInfusion_TuneLow_pos.m	Operator	hplc
Sample Name	KDD-088-1	Instrument	micrOTOF-Q III 8228888.20448
Comment	SB		

Acquisition Parameter

Source Type	ESI	Ion Polarity	Positive	Set Nebulizer	0.4 Bar
Focus	Not active	Set Capillary	4500 V	Set Dry Heater	180 °C
Scan Begin	50 m/z	Set End Plate Offset	-500 V	Set Dry Gas	4.0 l/min
Scan End	1000 m/z	Set Collision Cell RF	140.0 Vpp	Set Divert Valve	Waste

#	RT [min]	Area	Int. Type	I	S/N	Chromatogram	Max. m/z	FWHM [min]
n.a.	0.3	n.a.	Single spectrum	n.a.	n.a.	n.a.	226.9517	n.a.
n.a.	4.4	n.a.	Single spectrum	n.a.	n.a.	n.a.	295.1053	n.a.

+MS, 4.4min #263

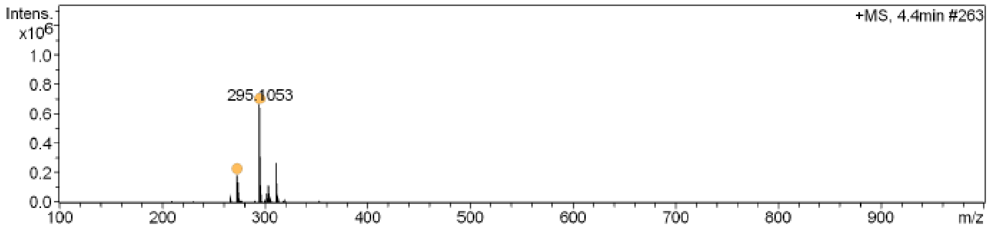

Meas. m/z	#	Ion Formula	m/z	err [ppm]	mSigma	# Sigma	Score	rdb	e⁻ Conf	N-Rule
273.1227	1	C15H17N2O3	273.1234	-2.6	24.6	1	100.00	8.5	even	ok
295.1053	1	C15H16N2NaO3	295.1053	-0.1	6.0	1	100.00	8.5	even	ok

Figure S4. HRMS (ESI-TOF) spectrum of ethyl 1-(oxiran-2-ylmethyl)-3-phenyl-1*H*-pyrazole-5-carboxylate (**2a**).

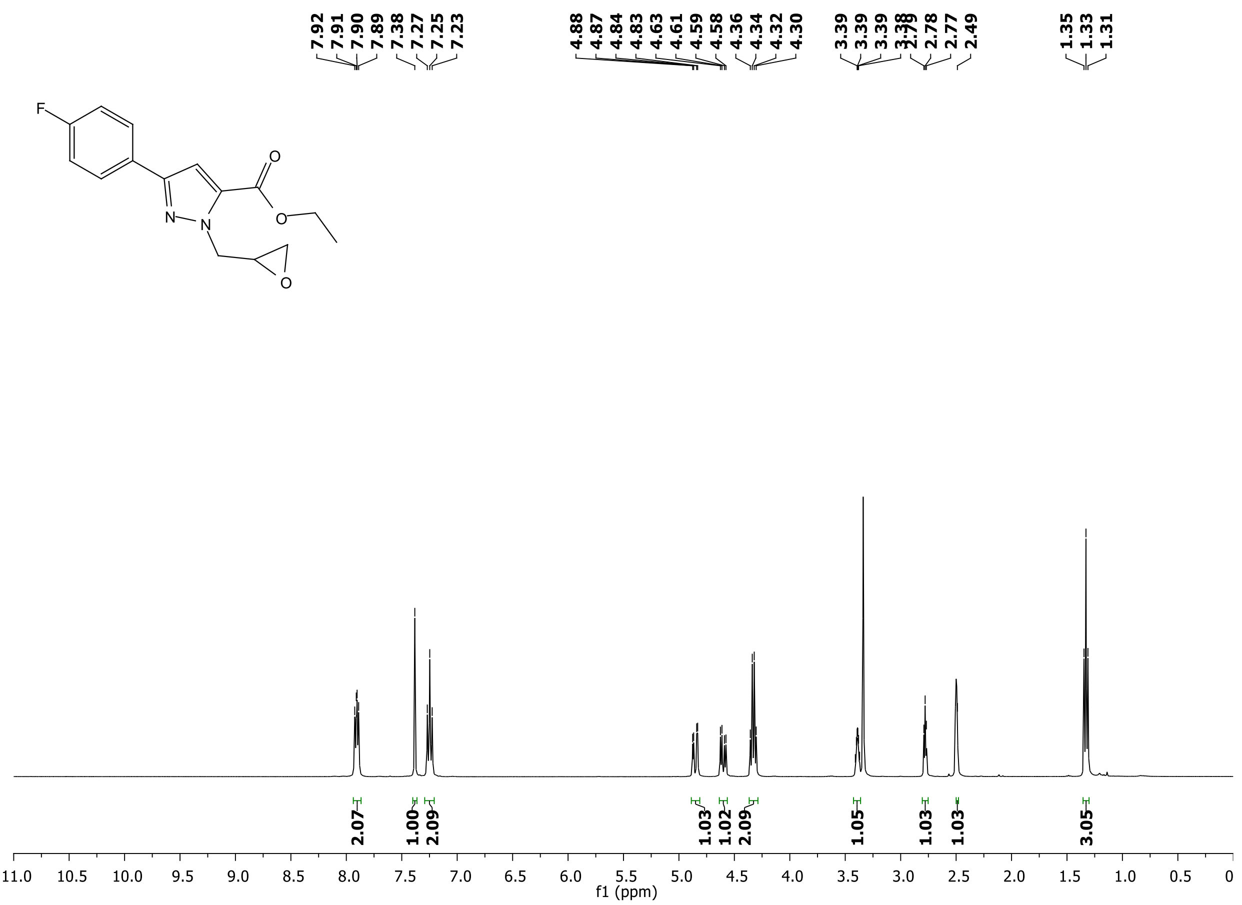

Figure S5. ^{1}H NMR spectrum (400 MHz, DMSO-d_6) of ethyl 3-(4-fluorophenyl)-1-(oxiran-2-ylmethyl)-1*H*-pyrazole-5-carboxylate (**2b**).

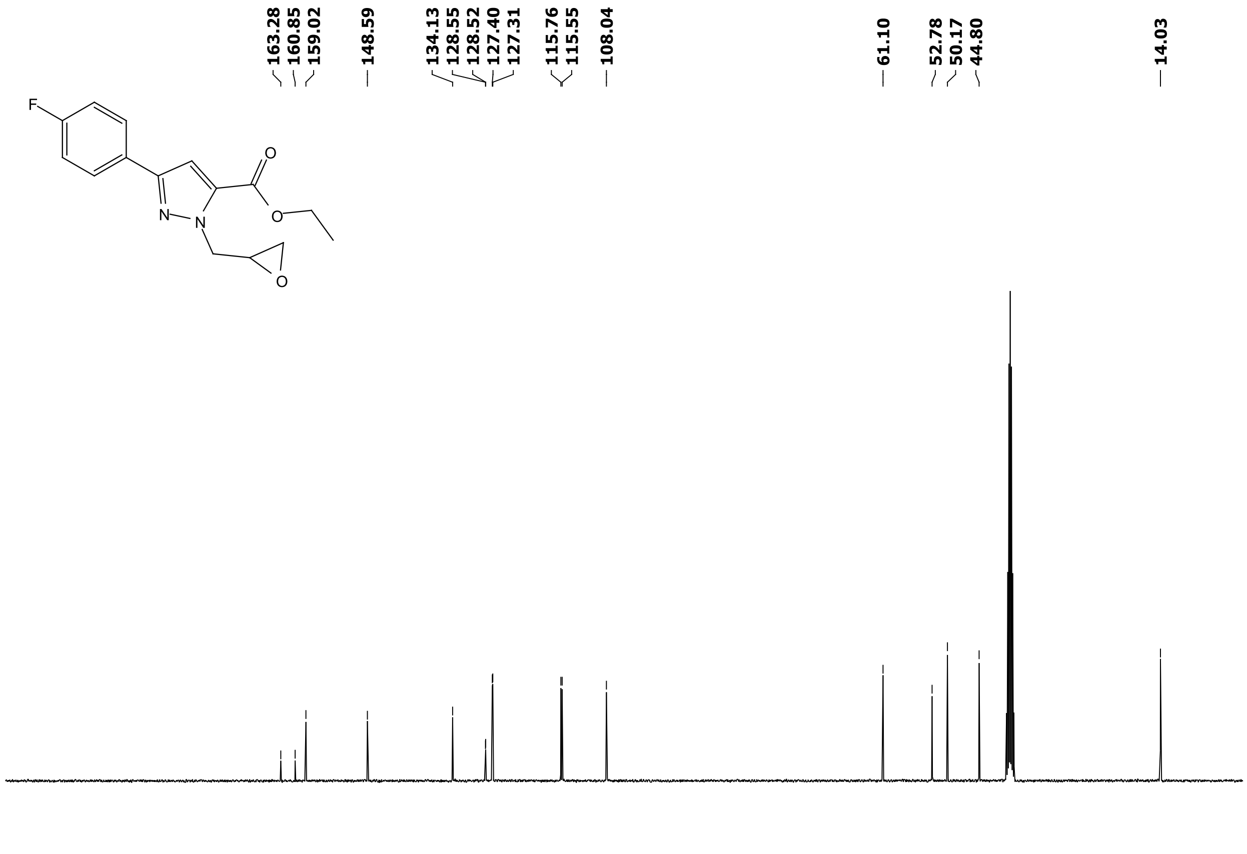

Figure S6. ^{13}C NMR spectrum (101 MHz, DMSO-d_6) of ethyl 3-(4-fluorophenyl)-1-(oxiran-2-ylmethyl)-1*H*-pyrazole-5-carboxylate (**2b**).

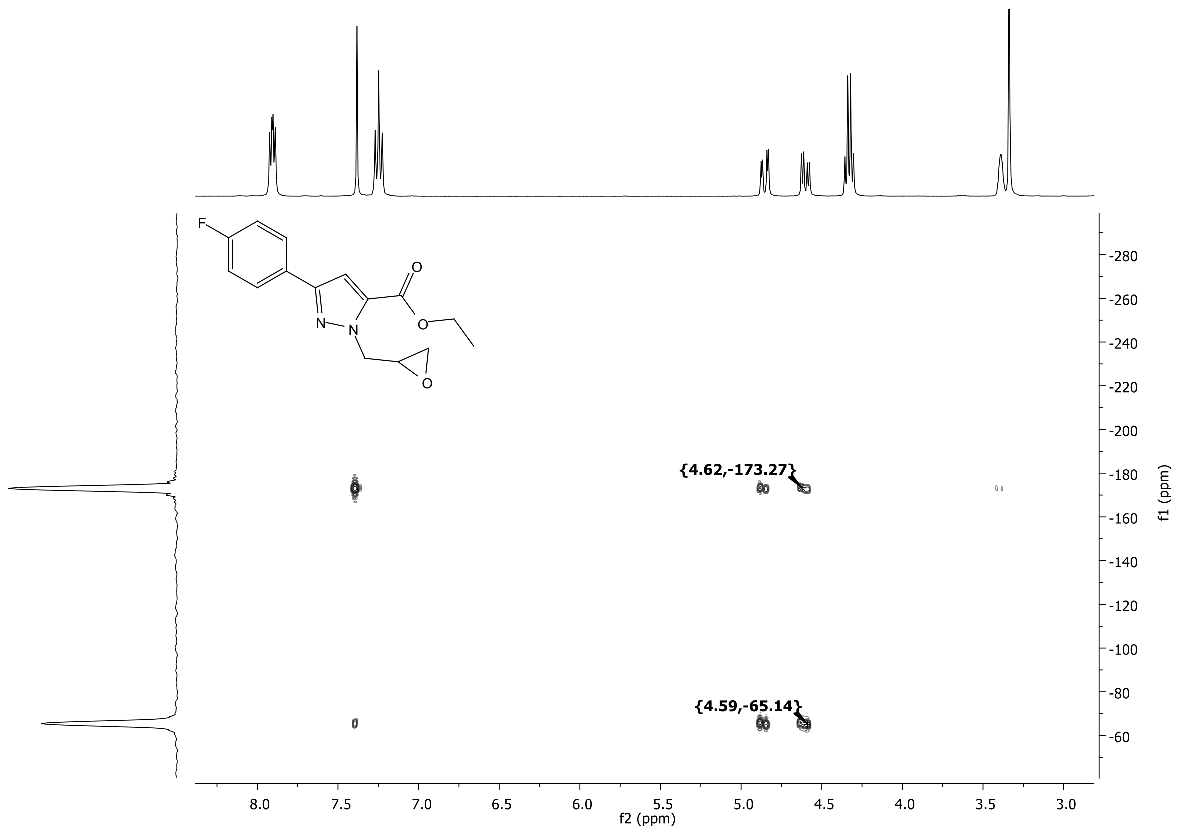

Figure S7. $^{1}H,^{15}N$-HMBC spectrum (40 MHz, DMSO-d_6) of ethyl 3-(4-fluorophenyl)-1-(oxiran-2-ylmethyl)-1*H*-pyrazole-5-carboxylate (**2b**).

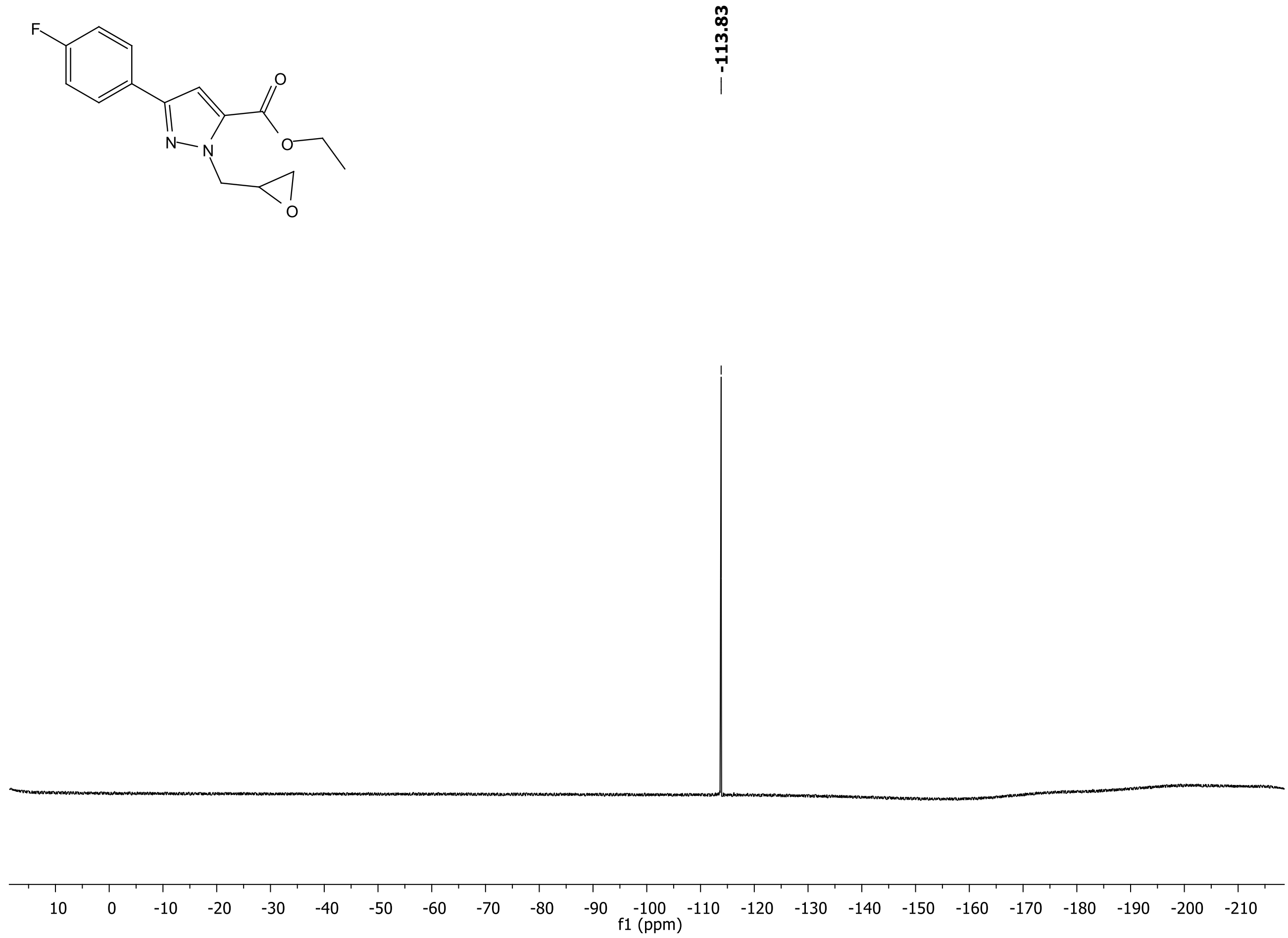

Figure S8. ^{19}F NMR spectrum (376 MHz, DMSO-d_6) of ethyl 3-(4-fluorophenyl)-1-(oxiran-2-ylmethyl)-1*H*-pyrazole-5-carboxylate (**2b**).

Compound Spectrum SmartFormula Report

Analysis Info

Analysis Name	D:\Data\KDD-122.d	Acquisition Date	4/6/2020 11:54:06 AM
Method	DirectInfusion_TuneLow_pos.m	Operator	hplc
Sample Name	KDD-122	Instrument	micrOTOF-Q III 8228888.20448
Comment	SB		

Acquisition Parameter

Source Type	ESI	Ion Polarity	Positive	Set Nebulizer	0.4 Bar
Focus	Not active	Set Capillary	4500 V	Set Dry Heater	180 °C
Scan Begin	50 m/z	Set End Plate Offset	-500 V	Set Dry Gas	4.0 l/min
Scan End	1000 m/z	Set Collision Cell RF	140.0 Vpp	Set Divert Valve	Waste

#	RT [min]	Area	Int. Type	I	S/N	Chromatogram	Max. m/z	FWHM [min]
n.a.	0.6	n.a.	Single spectrum	n.a.	n.a.	n.a.	226.9517	n.a.
n.a.	3.8	n.a.	Single spectrum	n.a.	n.a.	n.a.	313.0959	n.a.

+MS, 3.8min #227

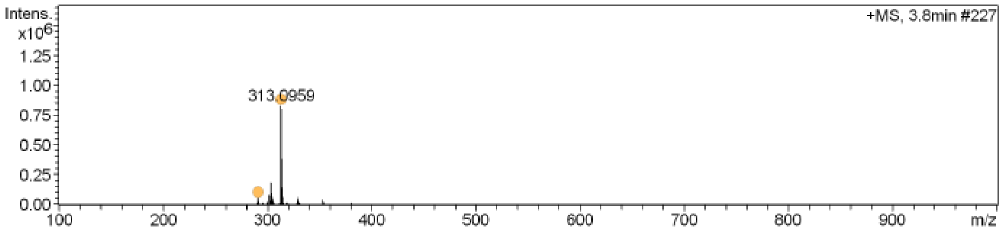

Meas. m/z	#	Ion Formula	m/z	err [ppm]	mSigma	# Sigma	Score	rdb	e⁻ Conf	N-Rule
291.1133	1	C15H16FN2O3	291.1139	-2.2	1.3	1	100.00	8.5	even	ok
313.0959	1	C15H15FN2NaO3	313.0959	0.1	5.5	1	100.00	8.5	even	ok

Figure S9. HRMS (ESI-TOF) spectrum of ethyl 3-(4-fluorophenyl)-1-(oxiran-2-ylmethyl)-1*H*-pyrazole-5-carboxylate (**2b**).

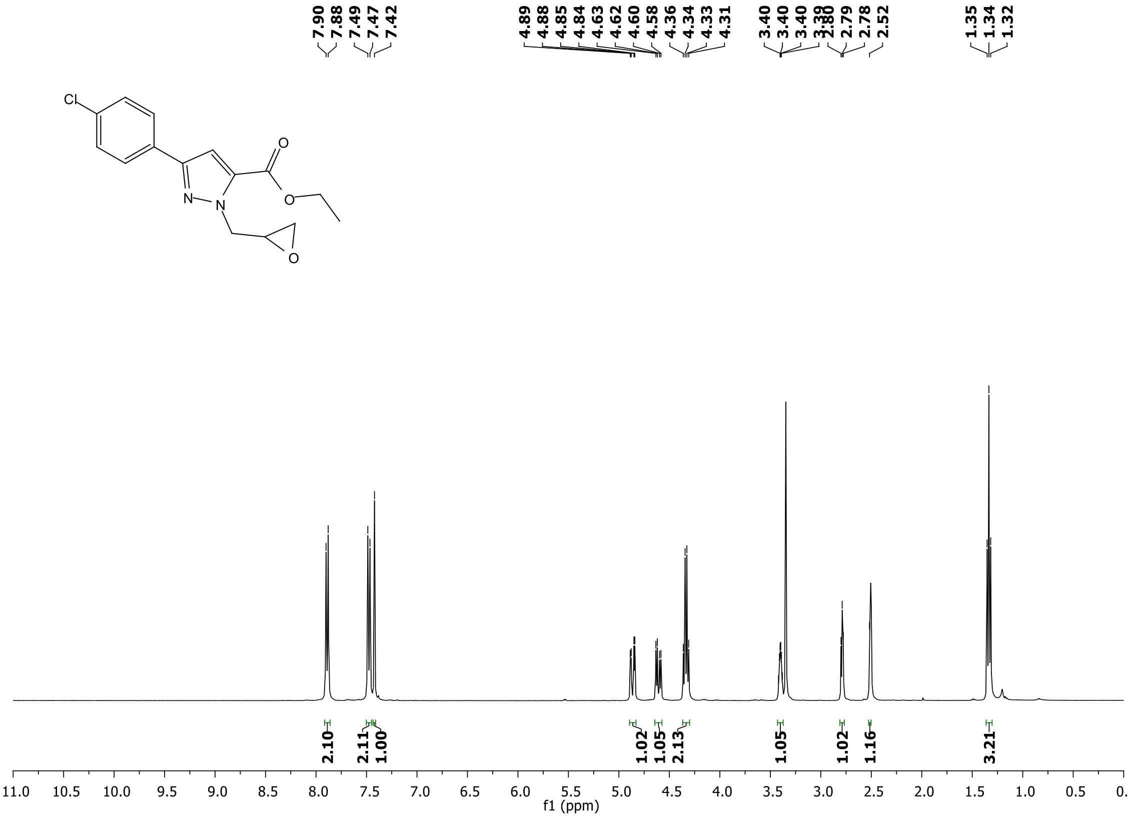

Figure S10. ^{1}H NMR spectrum (400 MHz, DMSO-d_6) of ethyl 3-(4-chlorophenyl)-1-(oxiran-2-ylmethyl)-1*H*-pyrazole-5-carboxylate (**2c**).

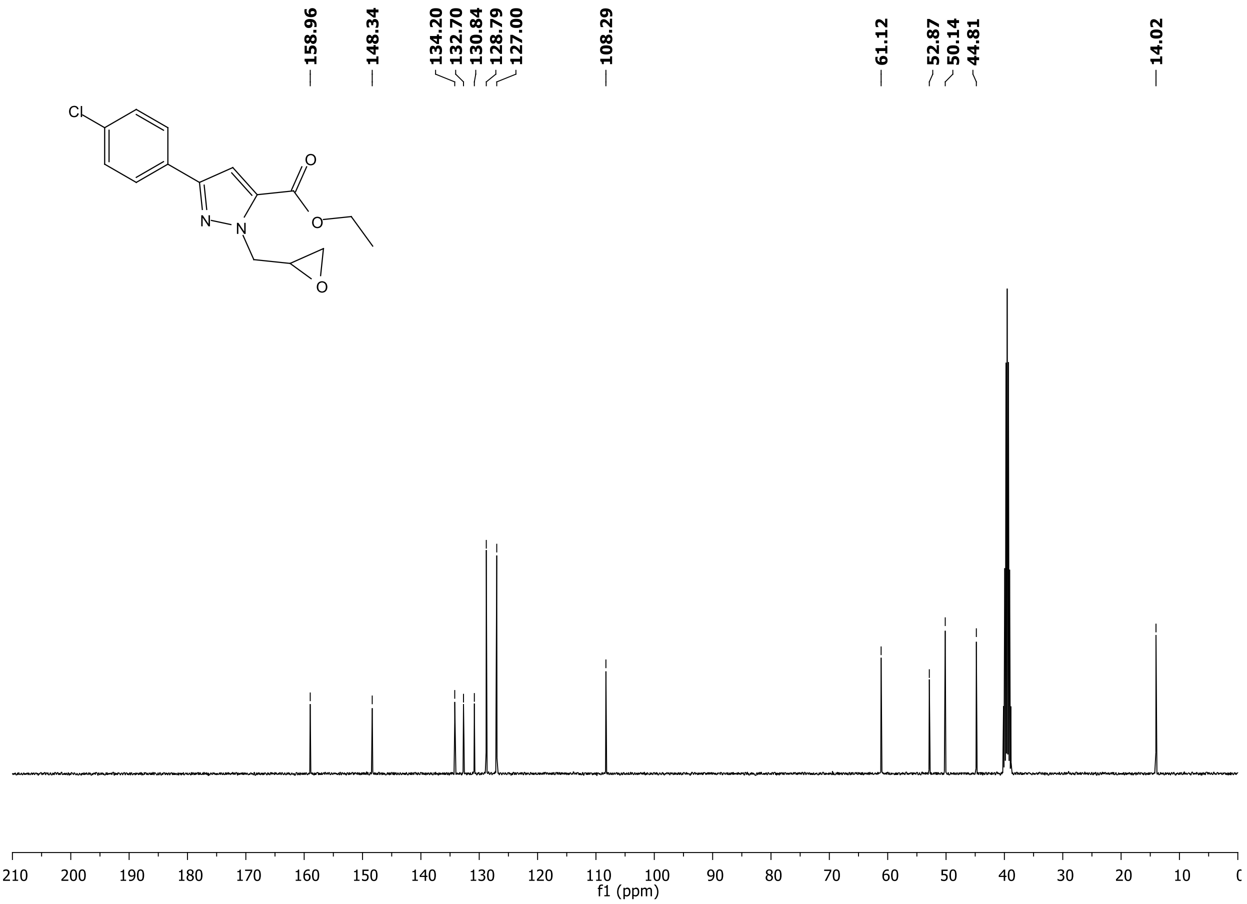

Figure S11. ^{13}C NMR spectrum (101 MHz, DMSO-d_6) of ethyl 3-(4-chlorophenyl)-1-(oxiran-2-ylmethyl)-1*H*-pyrazole-5-carboxylate (**2c**).

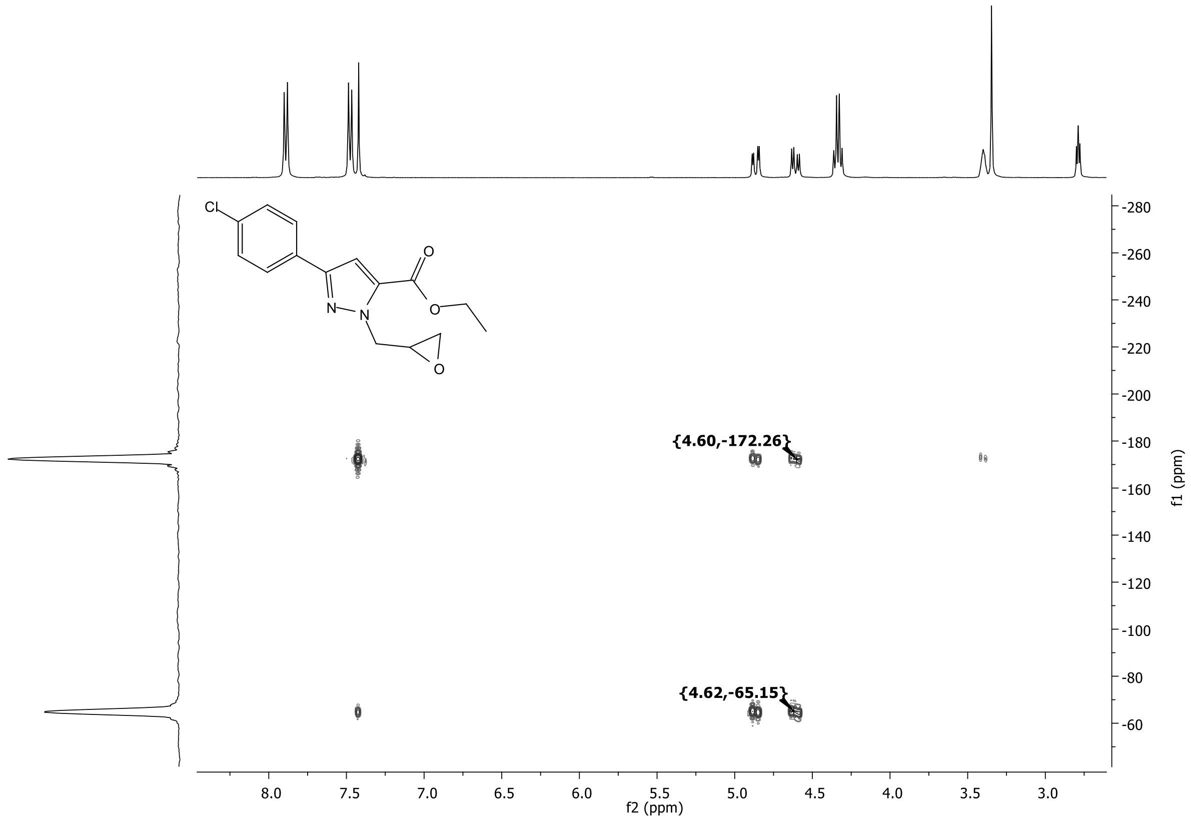

Figure S12. $^{1}H,^{15}N$-HMBC spectrum (40 MHz, DMSO-d_6) of ethyl 3-(4-chlorophenyl)-1-(oxiran-2-ylmethyl)-1*H*-pyrazole-5-carboxylate (**2c**).

Compound Spectrum SmartFormula Report

Analysis Info

Analysis Name	D:\Data\KDD-120.d	Acquisition Date	4/6/2020 11:38:03 AM
Method	DirectInfusion_TuneLow_pos.m	Operator	hplc
Sample Name	KDD-120	Instrument	micrOTOF-Q III 8228888.20448
Comment	SB		

Acquisition Parameter

Source Type	ESI	Ion Polarity	Positive	Set Nebulizer	0.4 Bar
Focus	Not active	Set Capillary	4500 V	Set Dry Heater	180 °C
Scan Begin	50 m/z	Set End Plate Offset	-500 V	Set Dry Gas	4.0 l/min
Scan End	1000 m/z	Set Collision Cell RF	140.0 Vpp	Set Divert Valve	Waste

#	RT [min]	Area	Int. Type	I	S/N	Chromatogram	Max. m/z	FWHM [min]
n.a.	1.4	n.a.	Single spectrum	n.a.	n.a.	n.a.	226.9518	n.a.
n.a.	6.1	n.a.	Single spectrum	n.a.	n.a.	n.a.	329.0663	n.a.

+MS, 6.1min #364

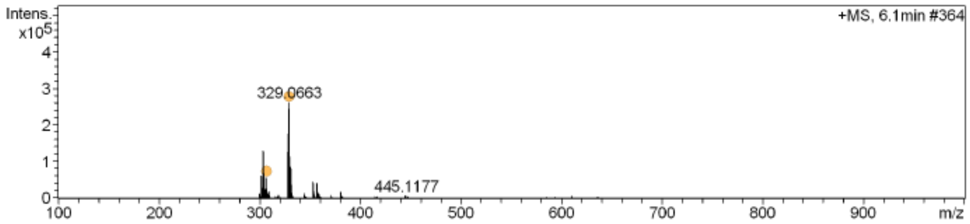

Meas. m/z	#	Ion Formula	m/z	err [ppm]	mSigma	# Sigma	Score	rdb	e¯ Conf	N-Rule
307.0843	1	C15H16ClN2O3	307.0844	-0.5	6.9	1	100.00	8.5	even	ok
329.0663	1	C15H15ClN2NaO3	329.0663	-0.0	2.6	1	100.00	8.5	even	ok

Figure S13. HRMS (ESI-TOF) spectrum of ethyl 3-(4-chlorophenyl)-1-(oxiran-2-ylmethyl)-1*H*-pyrazole-5-carboxylate (**2c**).

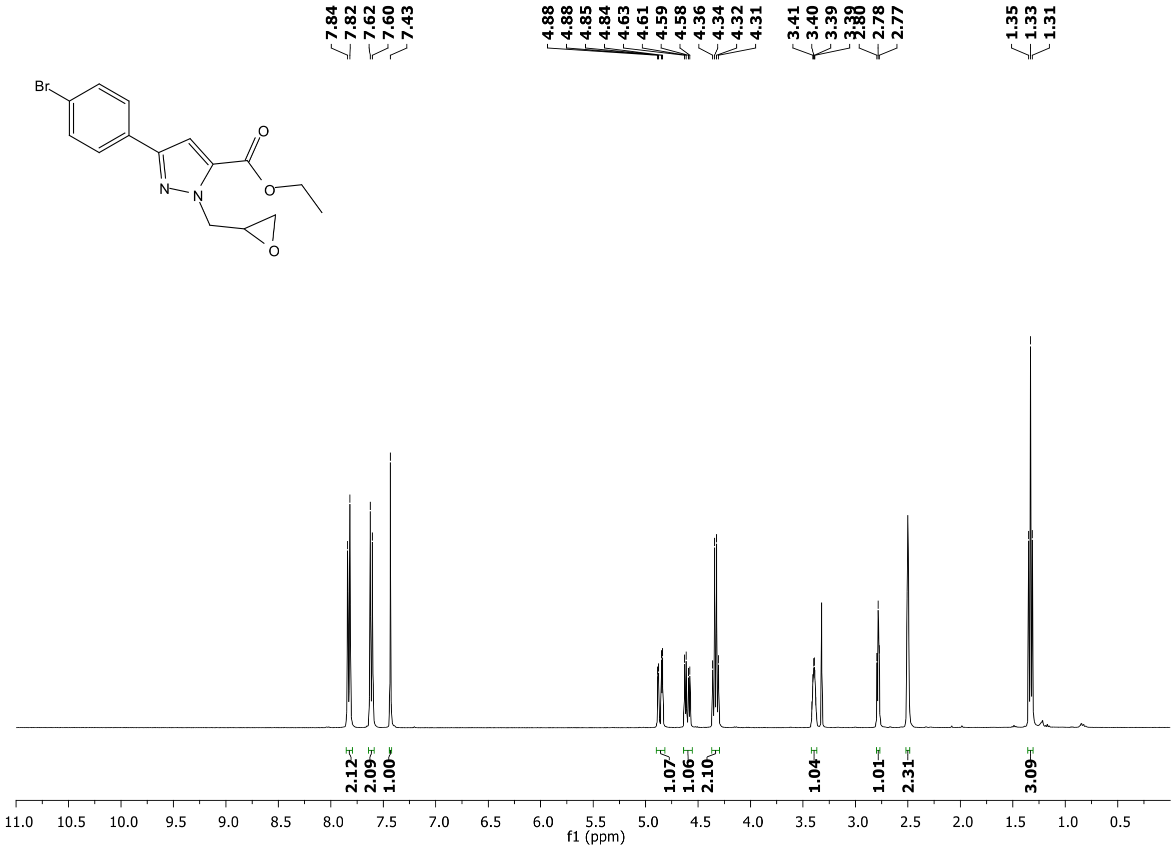

Figure S14. ^{1}H NMR spectrum (400 MHz, DMSO-d_6) of ethyl 3-(4-bromophenyl)-1-(oxiran-2-ylmethyl)-1*H*-pyrazole-5-carboxylate (**2d**).

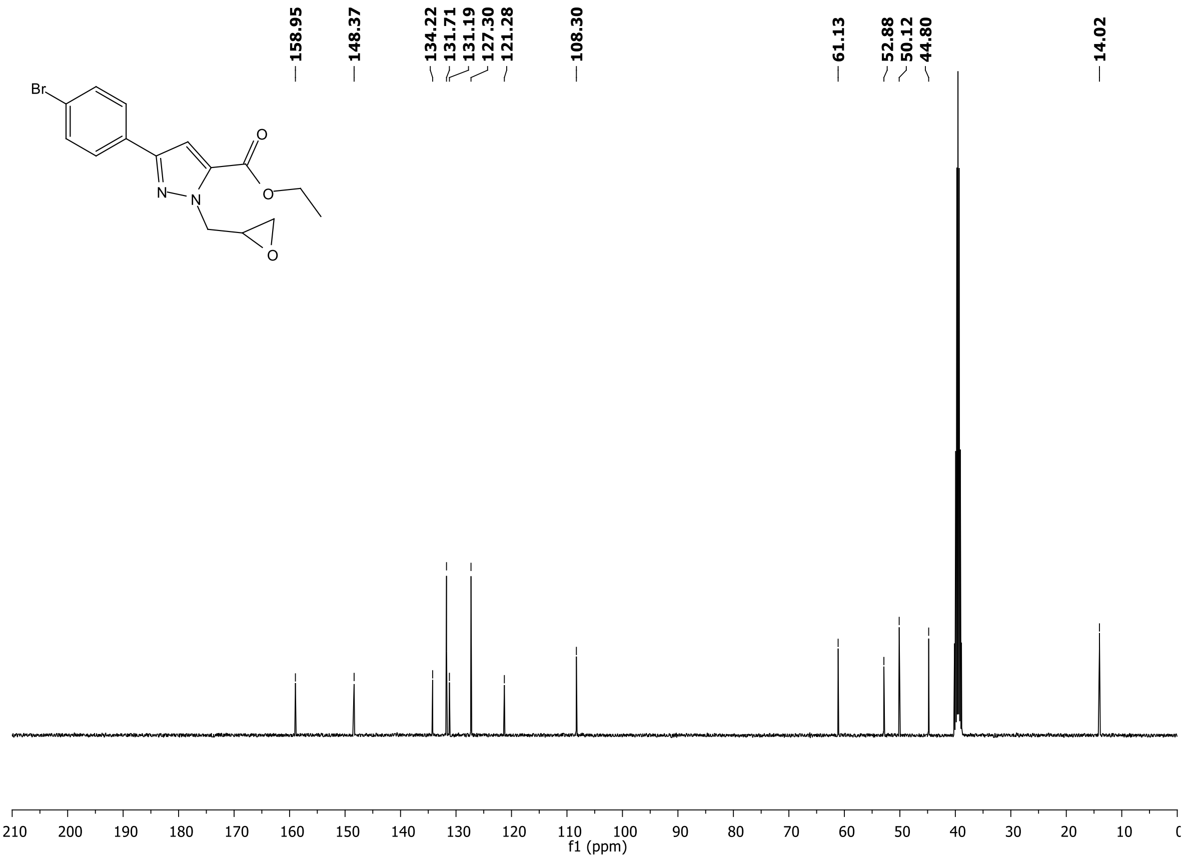

Figure S15. ^{13}C NMR spectrum (101 MHz, DMSO-d_6) of ethyl 3-(4-bromophenyl)-1-(oxiran-2-ylmethyl)-1*H*-pyrazole-5-carboxylate (**2d**).

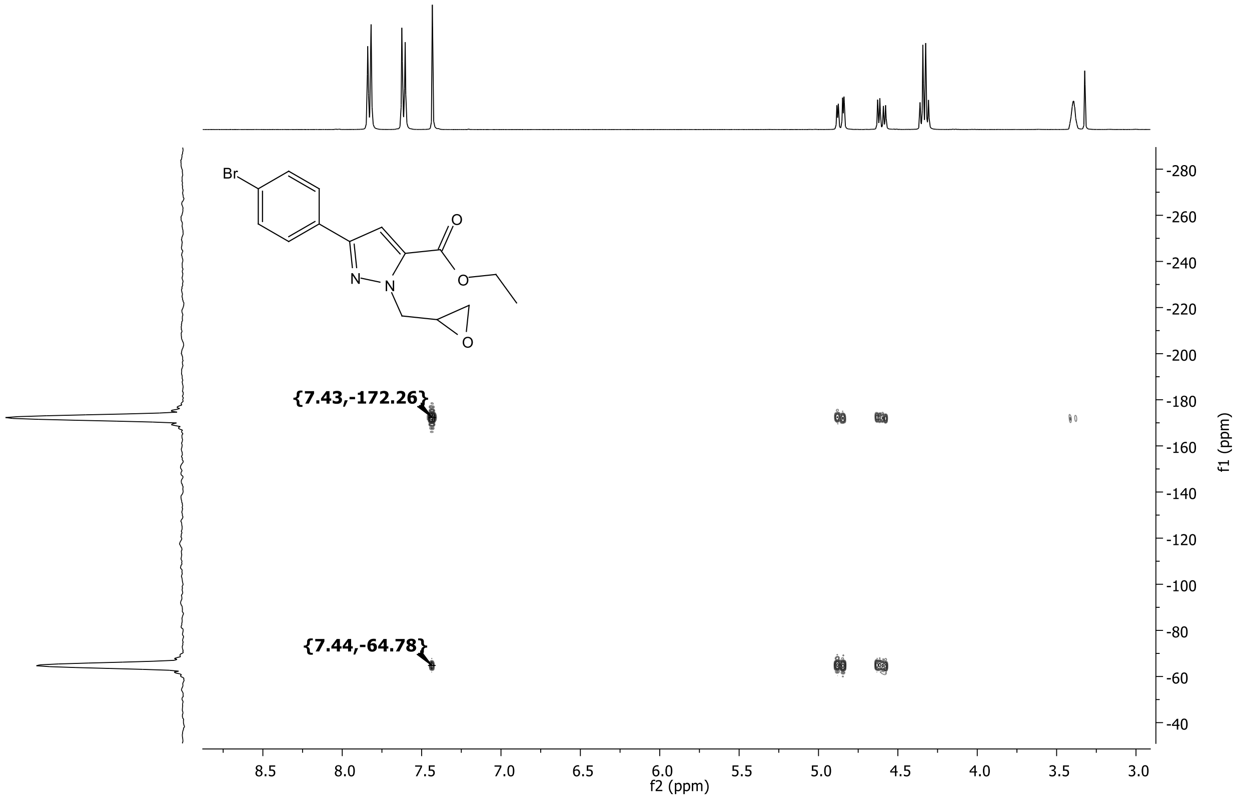

Figure S16. $^{1}H,^{15}N$-HMBC spectrum (40 MHz, DMSO-d_6) of ethyl 3-(4-bromophenyl)-1-(oxiran-2-ylmethyl)-1*H*-pyrazole-5-carboxylate (**2d**).

Compound Spectrum SmartFormula Report

Analysis Info

Analysis Name	D:\Data\KDD-108.d	Acquisition Date	4/6/2020 9:36:28 AM
Method	DirectInfusion_TuneLow_pos.m	Operator	hplc
Sample Name	KDD-108	Instrument	micrOTOF-Q III 8228888.20448
Comment	SB		

Acquisition Parameter

Source Type	ESI	Ion Polarity	Positive	Set Nebulizer	0.4 Bar
Focus	Not active	Set Capillary	4500 V	Set Dry Heater	180 °C
Scan Begin	50 m/z	Set End Plate Offset	-500 V	Set Dry Gas	4.0 l/min
Scan End	1000 m/z	Set Collision Cell RF	140.0 Vpp	Set Divert Valve	Waste

#	RT [min]	Area	Int. Type	I	S/N	Chromatogram	Max. m/z	FWHM [min]
n.a.	0.2	n.a.	Single spectrum	n.a.	n.a.	n.a.	226.9517	n.a.
n.a.	4.9	n.a.	Single spectrum	n.a.	n.a.	n.a.	353.2659	n.a.

+MS, 4.9min #294

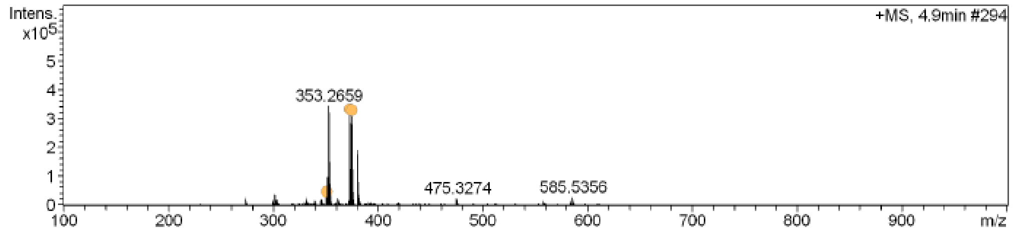

Meas. m/z	#	Ion Formula	m/z	err [ppm]	mSigma	# Sigma	Score	rdb	e⁻ Conf	N-Rule
351.0331	1	C15H16BrN2O3	351.0339	2.1	45.1	1	100.00	8.5	even	ok
373.0158	1	C15H15BrN2NaO3	373.0158	-0.2	3.5	1	100.00	8.5	even	ok
375.0136	1	C15H15BrN2NaO3	373.0158	-0.8	3.5	1	100.00	8.5	even	ok

Figure S17. HRMS (ESI-TOF) spectrum of ethyl 3-(4-bromophenyl)-1-(oxiran-2-ylmethyl)-1*H*-pyrazole-5-carboxylate (**2d**).

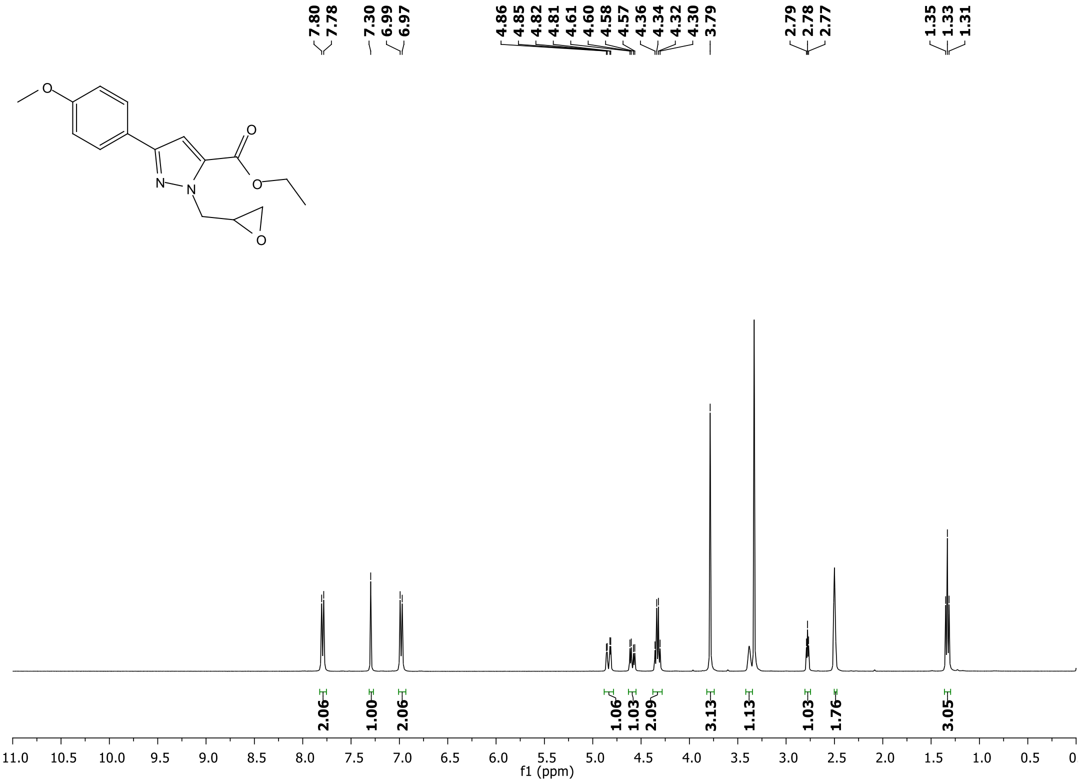

Figure S18. ¹H NMR spectrum (400 MHz, DMSO-d_6) of ethyl 3-(4-methoxyphenyl)-1-(oxiran-2-ylmethyl)-1*H*-pyrazole-5-carboxylate (**2e**).

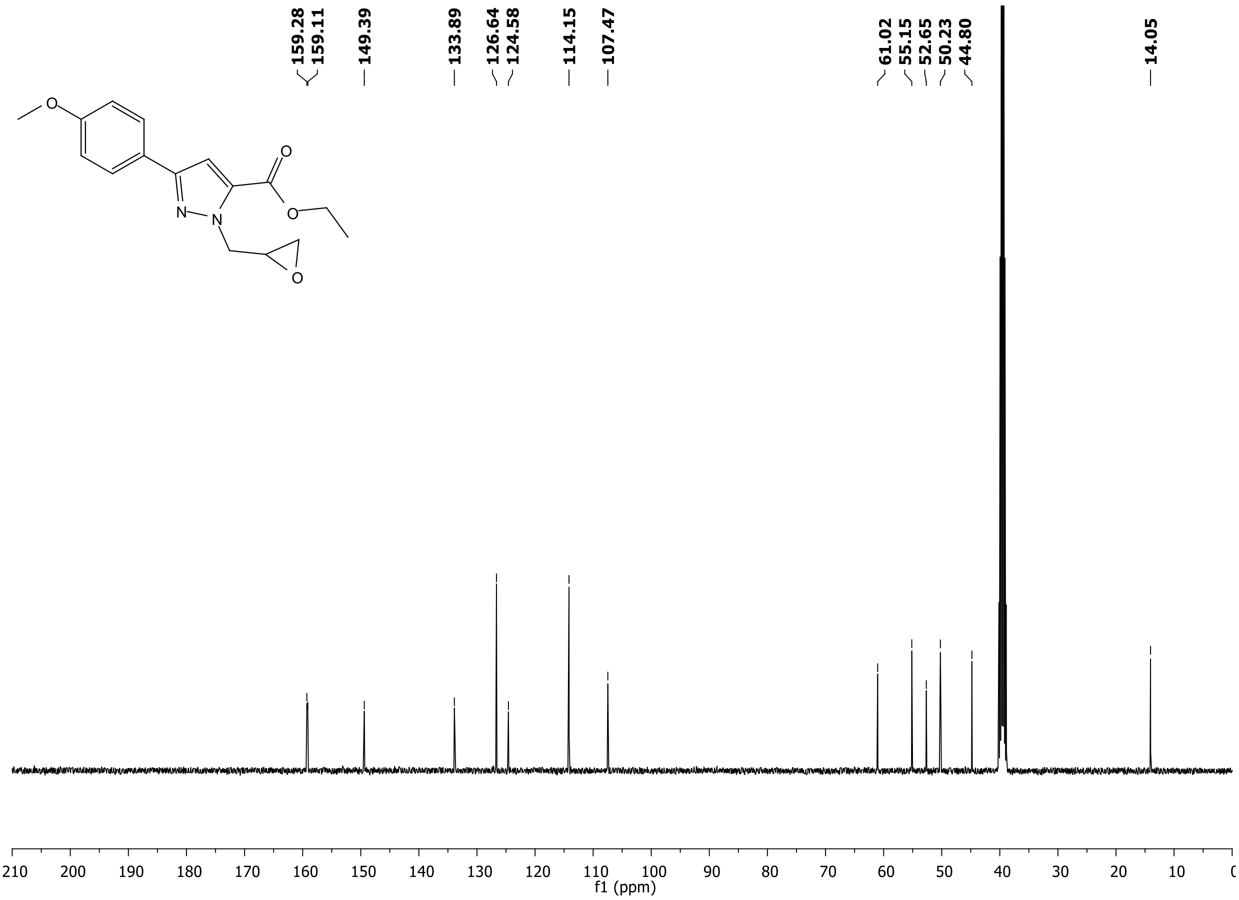

Figure S19. ^{13}C NMR spectrum (101 MHz, DMSO-d_6) of ethyl 3-(4-methoxyphenyl)-1-(oxiran-2-ylmethyl)-1*H*-pyrazole-5-carboxylate (**2e**).

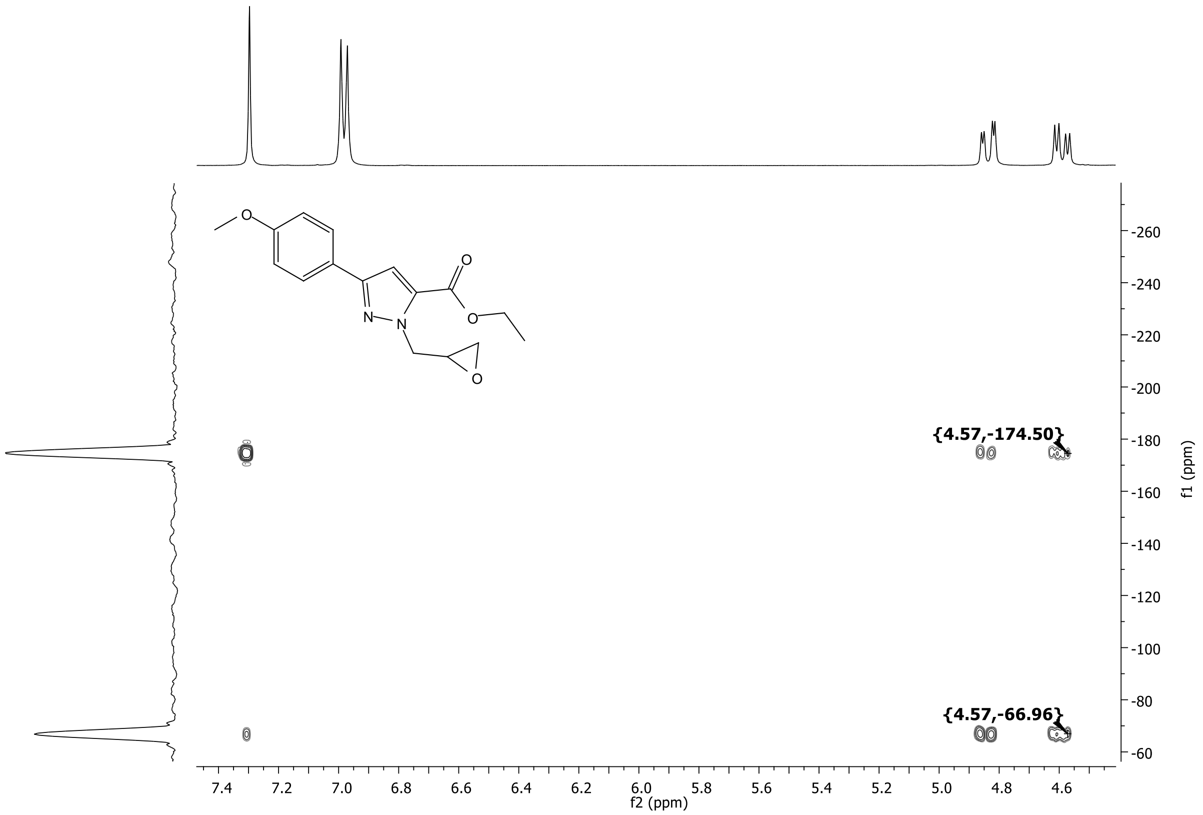

Figure S20. ^{1}H,^{15}N-HMBC spectrum (40 MHz, DMSO-d_6) of ethyl 3-(4-methoxyphenyl)-1-(oxiran-2-ylmethyl)-1*H*-pyrazole-5-carboxylate (**2e**).

Compound Spectrum SmartFormula Report

Analysis Info

Analysis Name	D:\Data\KDD-172-1.d	Acquisition Date	7/27/2021 3:58:29 PM
Method	DirectInfusion_TuneLow_pos.m	Operator	hplc
Sample Name	KDD-172-1	Instrument	micrOTOF-Q III 8228888.20448
Comment	SB		

Acquisition Parameter

Source Type	ESI	Ion Polarity	Positive	Set Nebulizer	0.4 Bar
Focus	Not active	Set Capillary	4500 V	Set Dry Heater	180 °C
Scan Begin	50 m/z	Set End Plate Offset	-500 V	Set Dry Gas	4.0 l/min
Scan End	1000 m/z	Set Collision Cell RF	140.0 Vpp	Set Divert Valve	Waste

#	RT [min]	Area	Int. Type	I	S/N	Chromatogram	Max. m/z	FWHM [min]
n.a.	0.1	n.a.	Single spectrum	n.a.	n.a.	n.a.	226.9518	n.a.
n.a.	5.3	n.a.	Single spectrum	n.a.	n.a.	n.a.	325.1159	n.a.

+MS, 5.3min #315

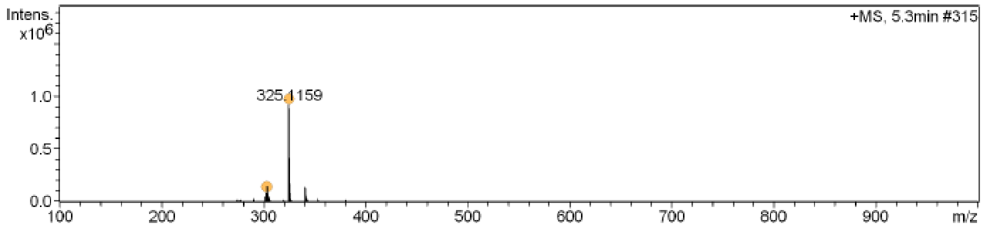

Meas. m/z	#	Ion Formula	m/z	err [ppm]	mSigma	# Sigma	Score	rdb	e⁻ Conf	N-Rule
303.1326	1	C16H19N2O4	303.1339	-4.3	9.6	1	100.00	8.5	even	ok
325.1159	1	C16H18N2NaO4	325.1159	-0.2	10.9	1	100.00	8.5	even	ok

Figure S21. HRMS (ESI-TOF) spectrum of ethyl 3-(4-methoxyphenyl)-1-(oxiran-2-ylmethyl)-1*H*-pyrazole-5-carboxylate (**2e**).

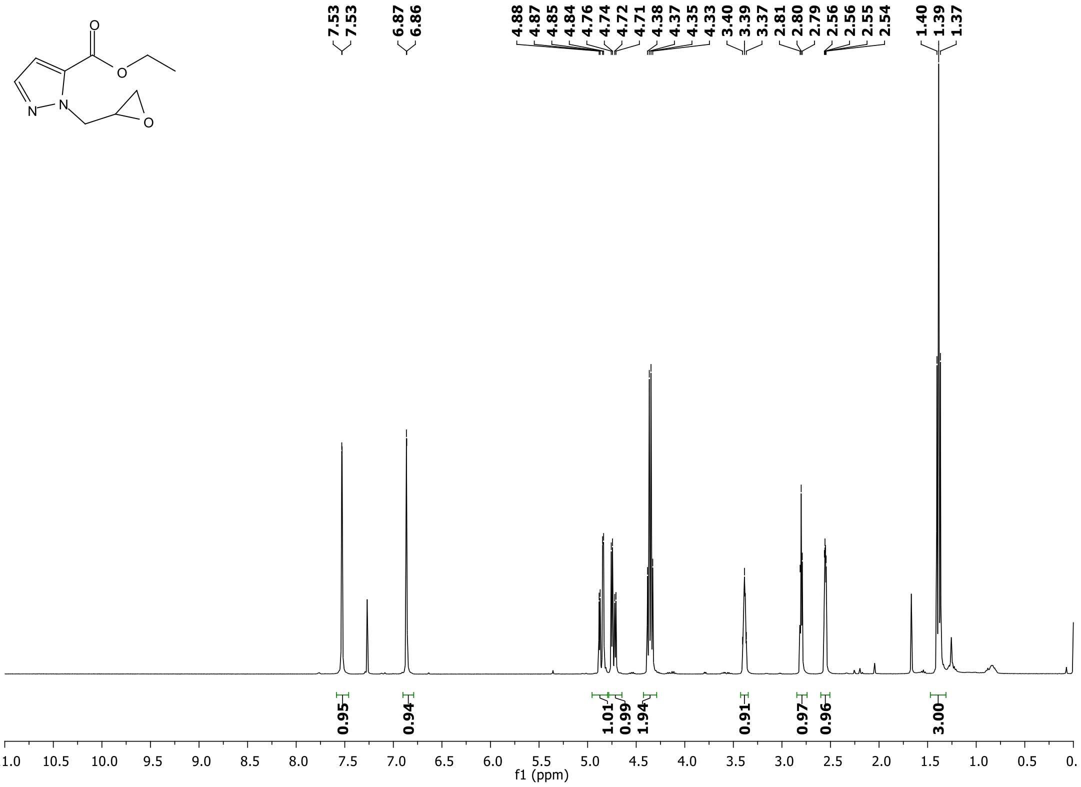

Figure S22. ^{1}H NMR spectrum (400 MHz, $CDCl_3$) of ethyl 1-(oxiran-2-ylmethyl)-1*H*-pyrazole-5-carboxylate (**2f**).

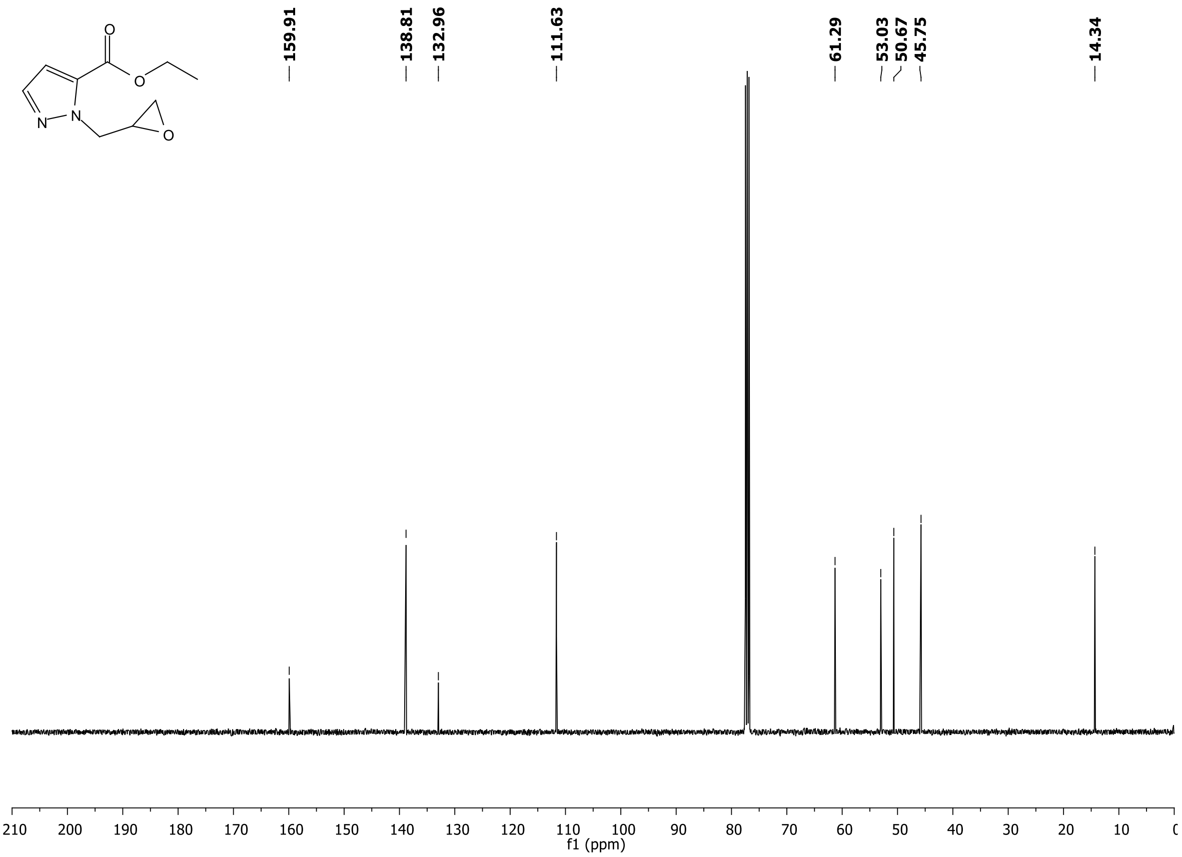

Figure S23. ^{13}C NMR spectrum (101 MHz, $CDCl_3$) of ethyl 1-(oxiran-2-ylmethyl)-1*H*-pyrazole-5-carboxylate (**2f**).

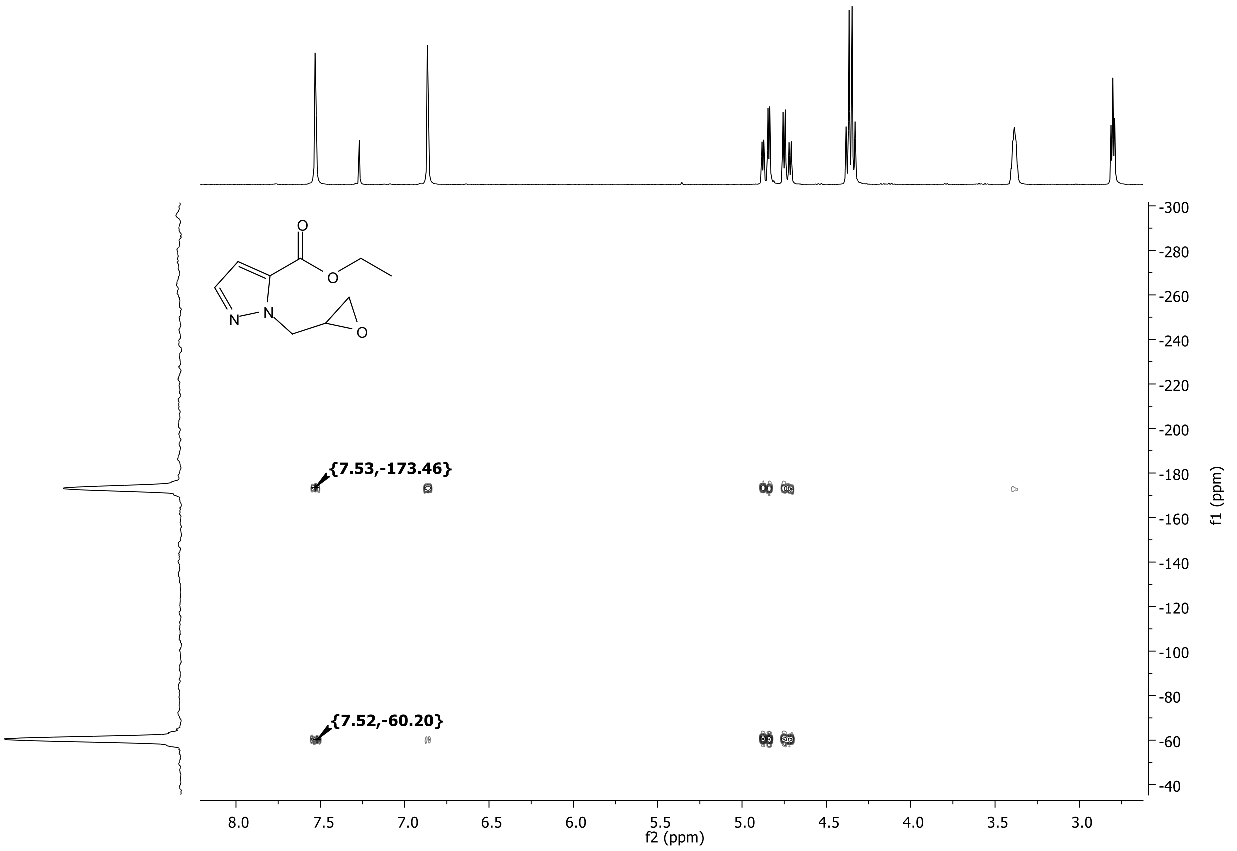

Figure S24. $^{1}H,^{15}N$-HMBC spectrum (40 MHz, $CDCl_3$) of ethyl 1-(oxiran-2-ylmethyl)-1*H*-pyrazole-5-carboxylate (**2f**).

Qualitative Compound Report

**Data File**	221111_MV-41-1_01.d	**Sample Name**	MV-41-1
**Sample Type**	Sample	**Position**	
**Instrument Name**	G6230B TOF	**User Name**	
**Acq Method**	HRMS_12min_ref.m	**Acquired Time**	11-Nov-22 10:27:10 AM
**IRM Calibration Status**	Success	**DA Method**	test.m
**Comment**			
**Sample Group**		**Info.**	
**Stream Name**		**Acquisition SW Version**	6200 series TOF/6500 series Q-TOF B.09.00 (B9044.1 SP1)

Compound Table

Compound Label	RT	Mass	Abund	Formula	Tgt Mass	Diff (ppm)
Cpd 1: C9 H12 N2 O3	8.025	196.0847	1806345	C9 H12 N2 O3	196.0848	-0.4

Compound Label	m/z	RT	Algorithm	Mass
Cpd 1: C9 H12 N2 O3	197.0916	8.025	Find By Formula	196.0847

Compound Chromatograms

MS Spectrum

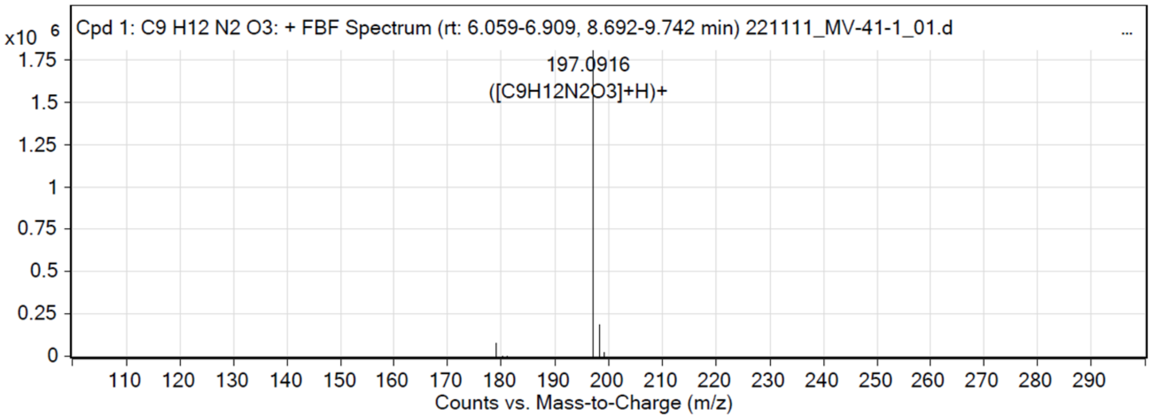

Figure S25. HRMS (ESI-TOF) spectrum of ethyl 1-(oxiran-2-ylmethyl)-1*H*-pyrazole-5-carboxylate (**2f**).

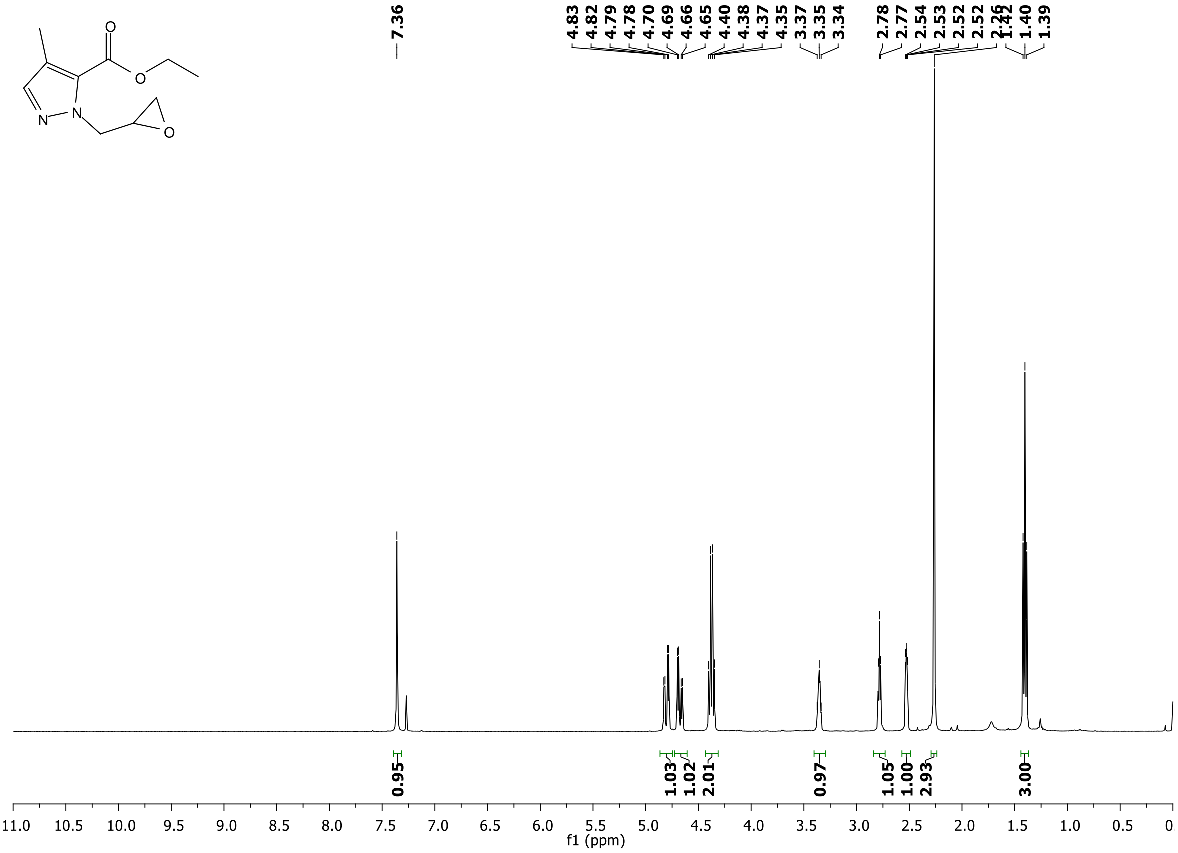

Figure S26. ^{1}H NMR spectrum (400 MHz, $CDCl_3$) of ethyl 4-methyl-1-(oxiran-2-ylmethyl)-1*H*-pyrazole-5-carboxylate (**2g**).

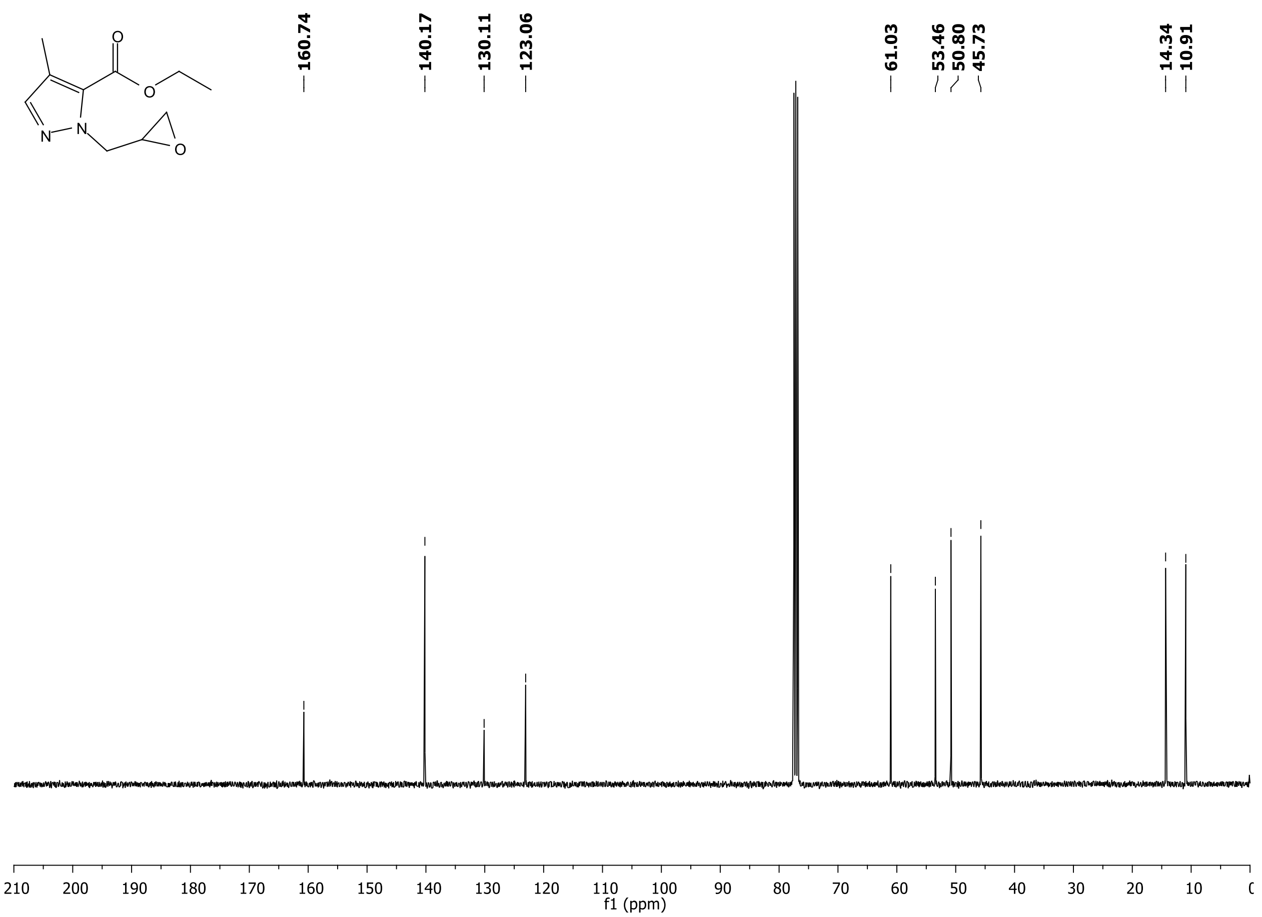

Figure S27. ^{13}C NMR spectrum (101 MHz, $CDCl_3$) of ethyl 4-methyl-1-(oxiran-2-ylmethyl)-1*H*-pyrazole-5-carboxylate (**2g**).

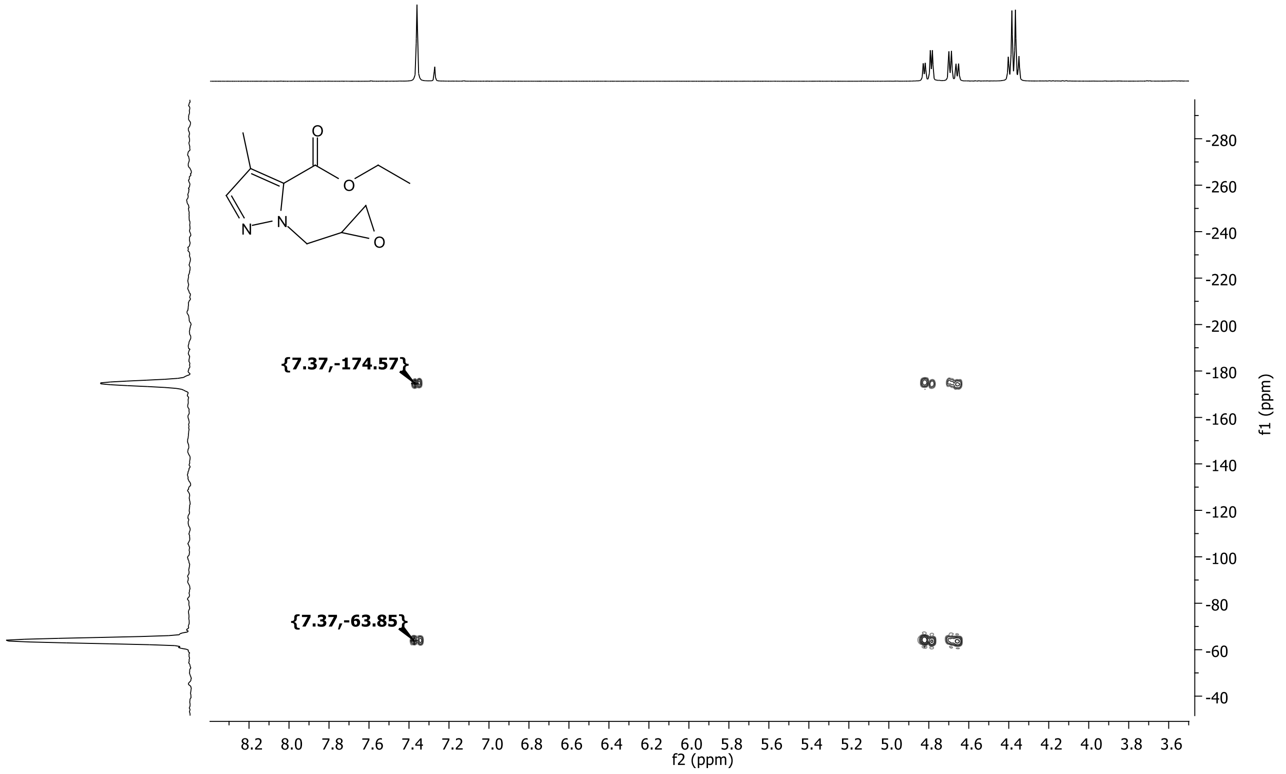

Figure S28. $^{1}H,^{15}N$-HMBC spectrum (40 MHz, $CDCl_3$) of ethyl 4-methyl-1-(oxiran-2-ylmethyl)-1H-pyrazole-5-carboxylate (**2g**).

Qualitative Compound Report

**Data File**	221111_MV-45-1_01.d	**Sample Name**	MV-45-1
**Sample Type**	Sample	**Position**	
**Instrument Name**	G6230B TOF	**User Name**	
**Acq Method**	HRMS_12min_ref.m	**Acquired Time**	11-Nov-22 10:59:10 AM
**IRM Calibration Status**	Success	**DA Method**	test.m
**Comment**			
**Sample Group**		**Info.**	
**Stream Name**		**Acquisition SW Version**	6200 series TOF/6500 series Q-TOF B.09.00 (B9044.1 SP1)

Compound Table

Compound Label	RT	Mass	Abund	Formula	Tgt Mass	Diff (ppm)
Cpd 1: C10 H14 N2 O3	7.265	210.1003	1948545	C10 H14 N2 O3	210.1004	-0.89

Compound Label	*m/z*	RT	Algorithm	Mass
Cpd 1: C10 H14 N2 O3	211.1067	7.265	Find By Formula	210.1003

Compound Chromatograms

MS Spectrum

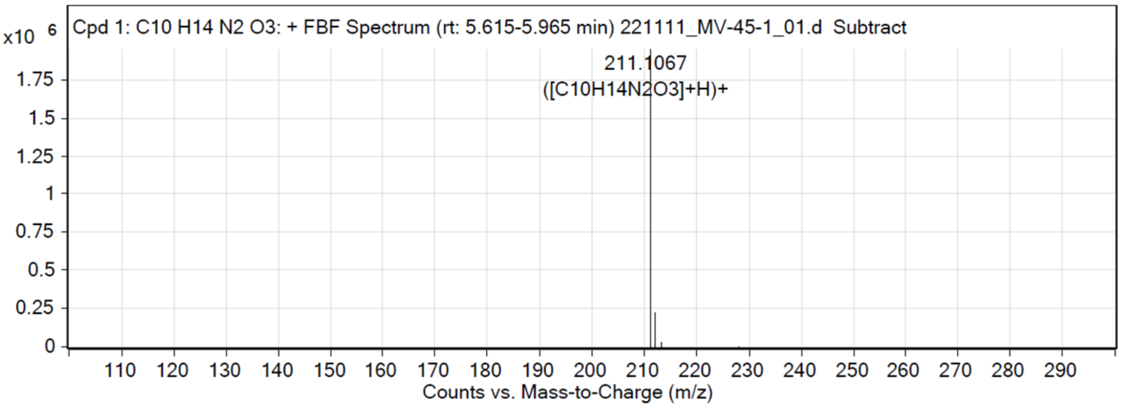

Figure S29. HRMS (ESI-TOF) spectrum of ethyl 4-methyl-1-(oxiran-2-ylmethyl)-1*H*-pyrazole-5-carboxylate (**2g**).

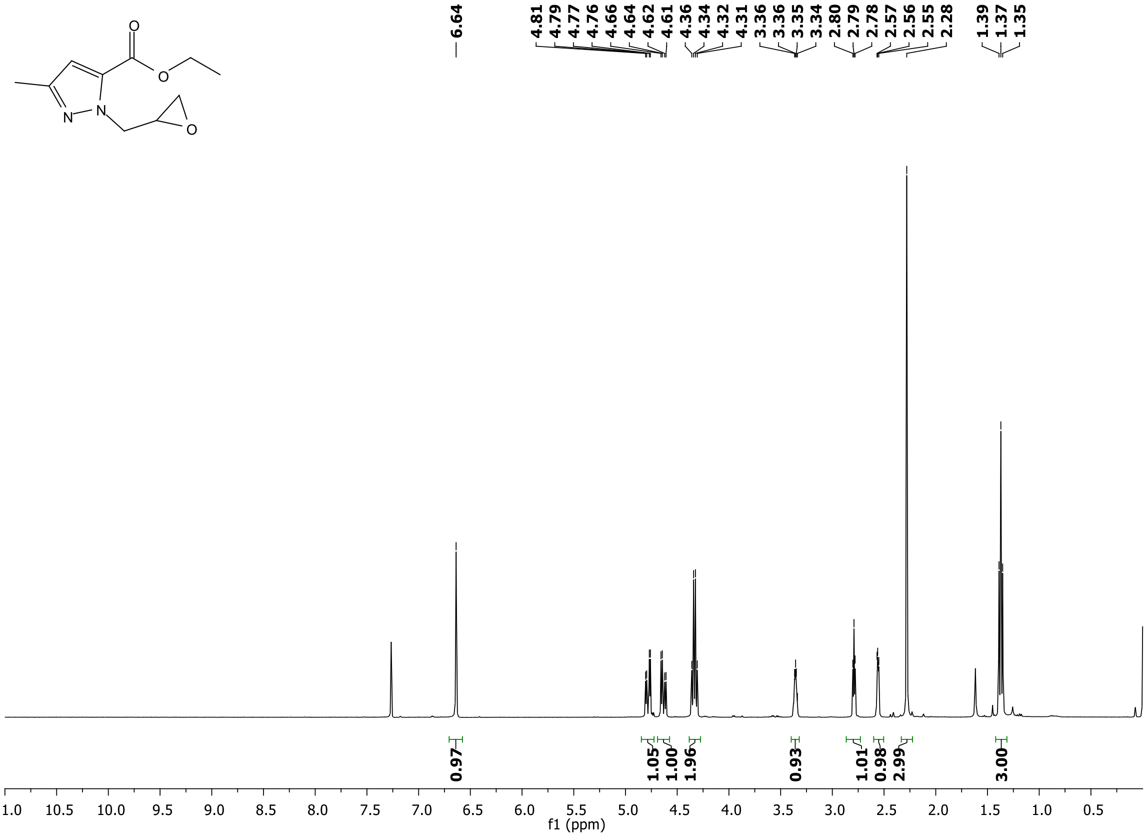

Figure S30. ^{1}H NMR spectrum (400 MHz, $CDCl_3$) of ethyl 3-methyl-1-(oxiran-2-ylmethyl)-1*H*-pyrazole-5-carboxylate (**2h**).

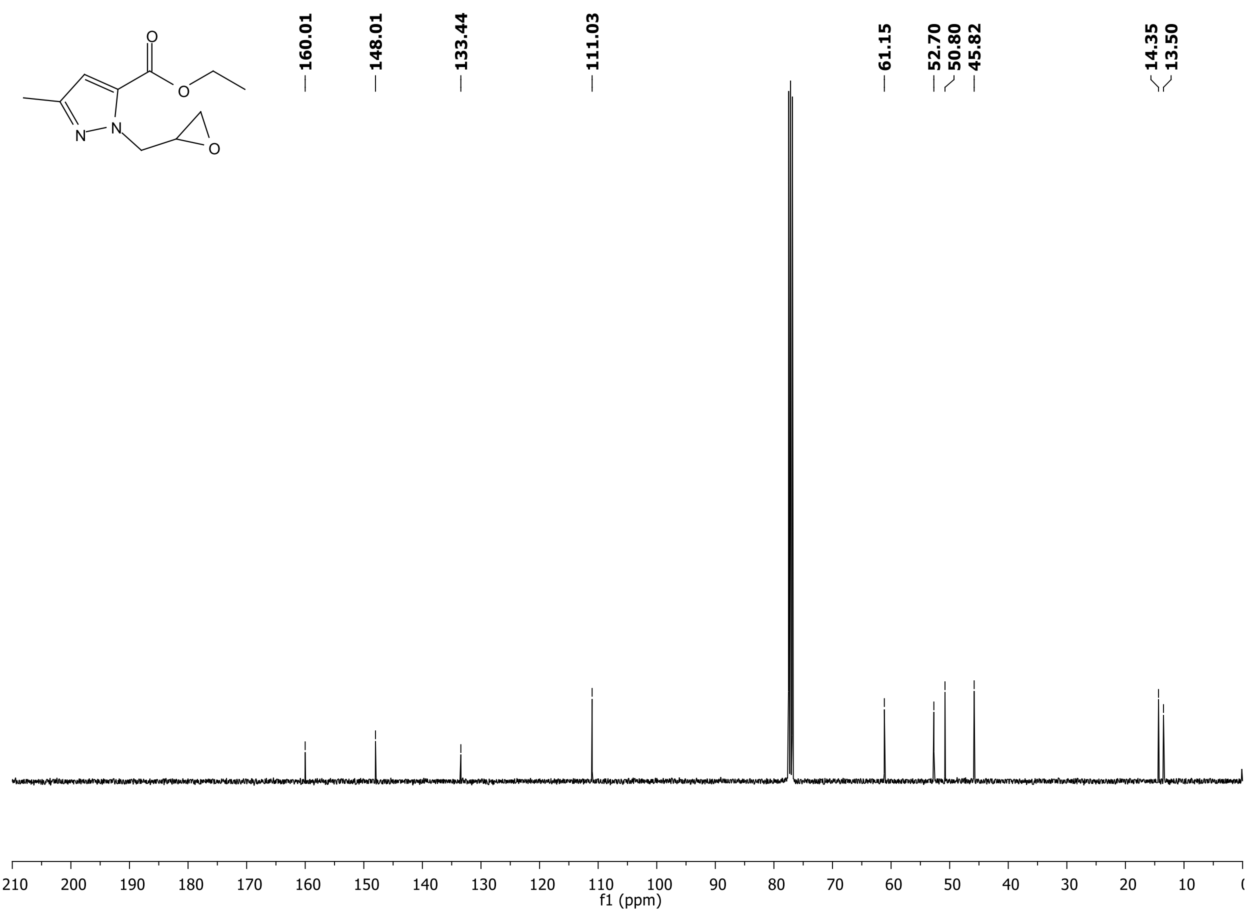

Figure S31. ^{13}C NMR spectrum (101 MHz, $CDCl_3$) of ethyl 3-methyl-1-(oxiran-2-ylmethyl)-1*H*-pyrazole-5-carboxylate (**2h**).

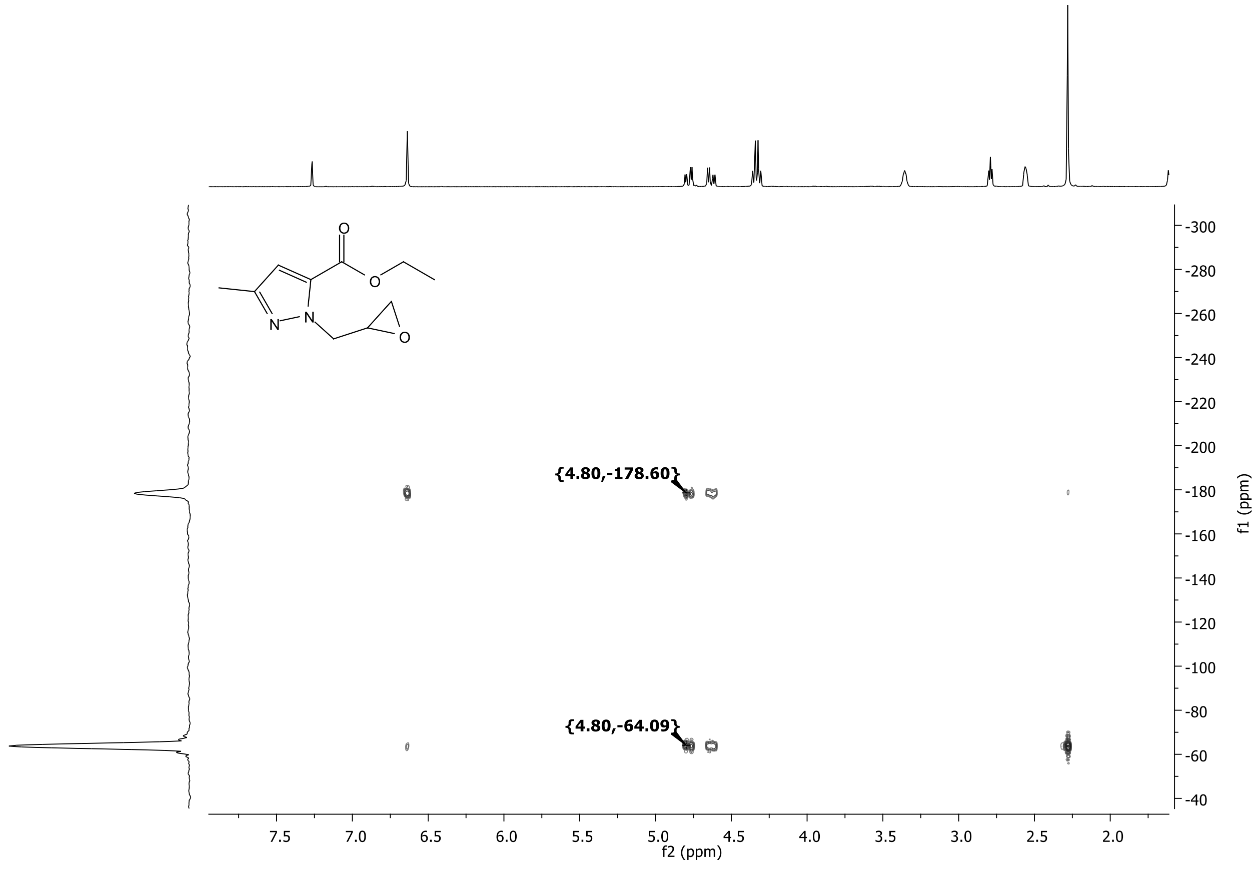

Figure S32. ^{1}H,^{15}N-HMBC spectrum (40 MHz, $CDCl_3$) of ethyl 3-methyl-1-(oxiran-2-ylmethyl)-1*H*-pyrazole-5-carboxylate (**2h**).

Qualitative Compound Report

**Data File**	221114_MV-47-1_01.d	**Sample Name**	MV-47-1
**Sample Type**	Sample	**Position**	
**Instrument Name**	G6230B TOF	**User Name**	
**Acq Method**	HRMS_12min_ref.m	**Acquired Time**	14-Nov-22 8:54:56 AM
**IRM Calibration Status**	Success	**DA Method**	test.m
**Comment**			
**Sample Group**		**Info.**	
**Stream Name**		**Acquisition SW Version**	6200 series TOF/6500 series Q-TOF B.09.00 (B9044.1 SP1)

Compound Table

Compound Label	RT	Mass	Abund	Formula	Tgt Mass	Diff (ppm)
Cpd 1: C10 H14 N2 O3	6.815	210.1003	983783	C10 H14 N2 O3	210.1004	-0.59

Compound Label	m/z	RT	Algorithm	Mass
Cpd 1: C10 H14 N2 O3	211.1075	6.815	Find By Formula	210.1003

Compound Chromatograms

MS Spectrum

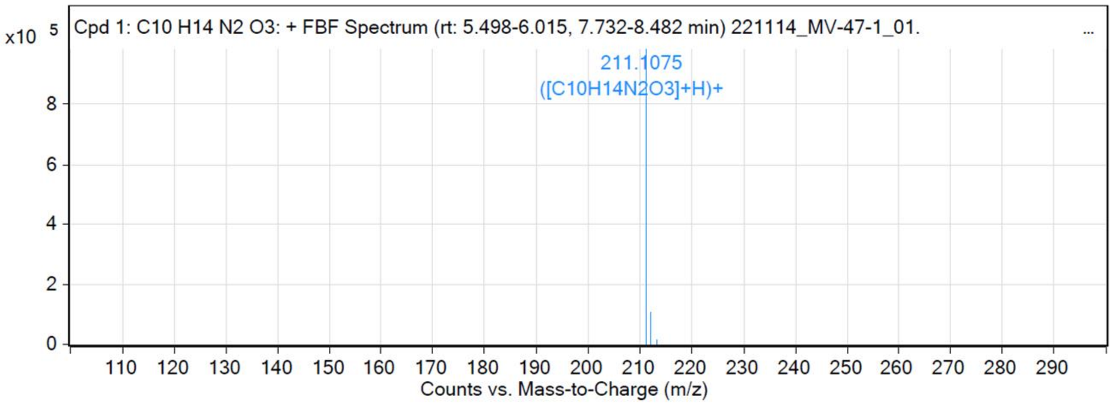

Figure S33. HRMS (ESI-TOF) spectrum of ethyl 3-methyl-1-(oxiran-2-ylmethyl)-1*H*-pyrazole-5-carboxylate (**2h**).

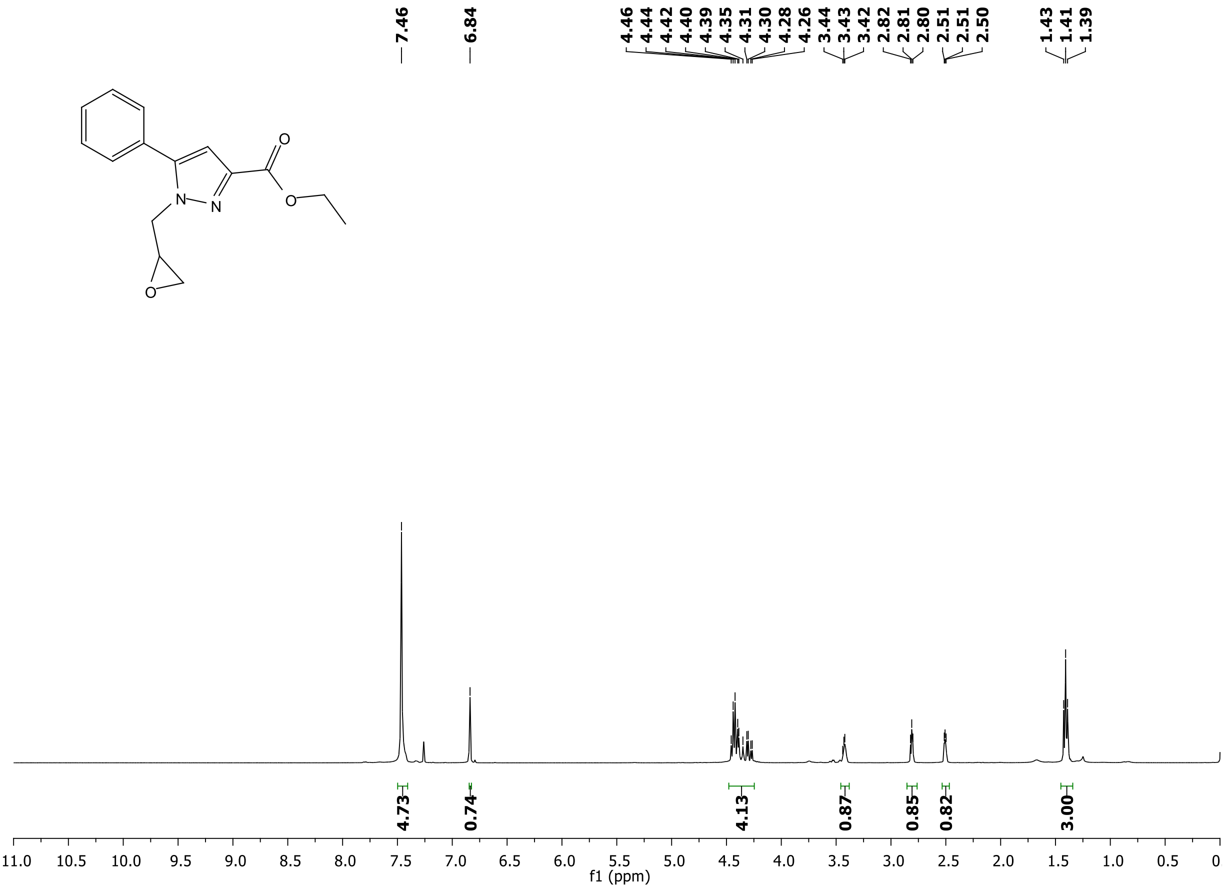

Figure S34. ¹H NMR spectrum (400 MHz, $CDCl_3$) of ethyl 1-(oxiran-2-ylmethyl)-5-phenyl-1*H*-pyrazole-3-carboxylate (**3a**).

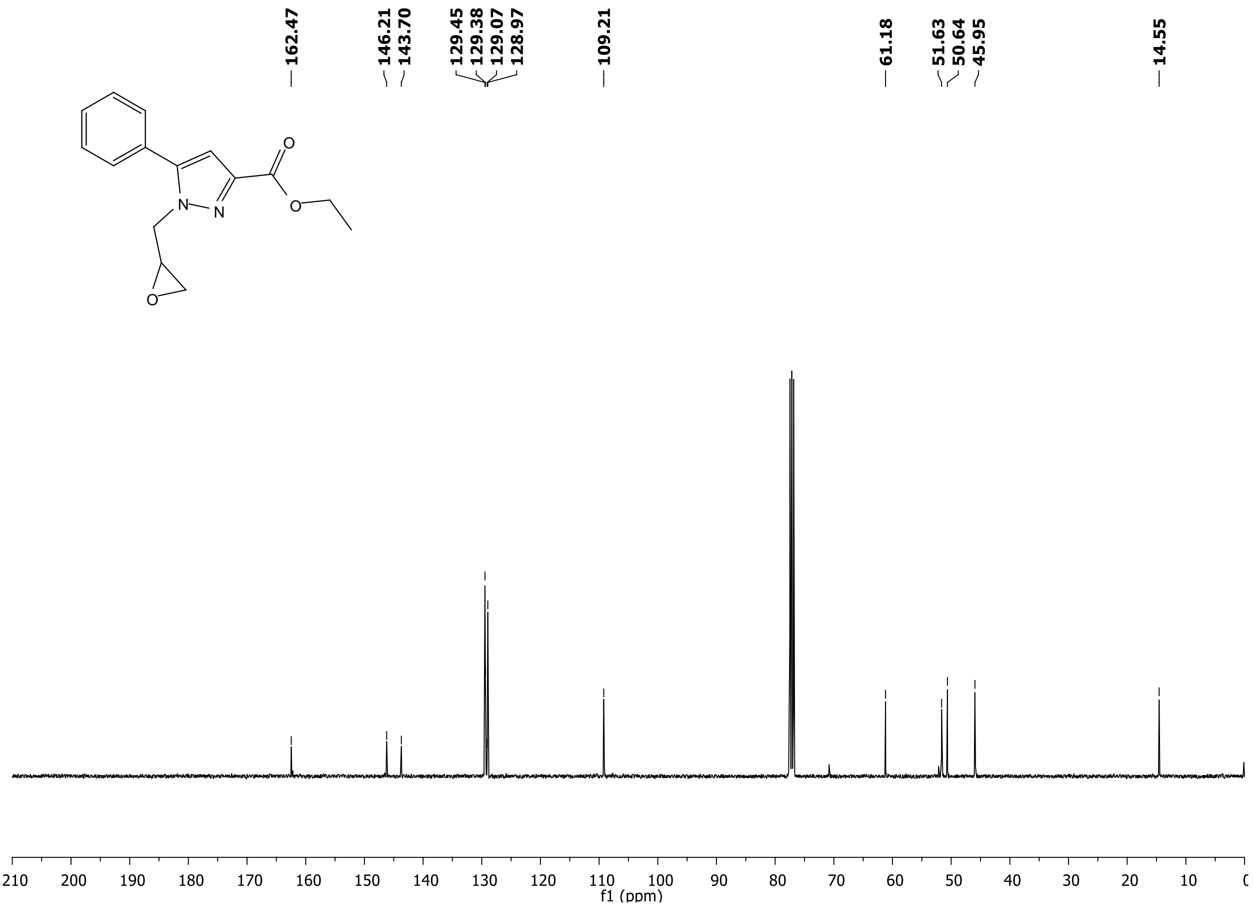

Figure S35. ^{13}C NMR spectrum (101 MHz, $CDCl_3$) of ethyl 1-(oxiran-2-ylmethyl)-5-phenyl-1*H*-pyrazole-3-carboxylate (**3a**).

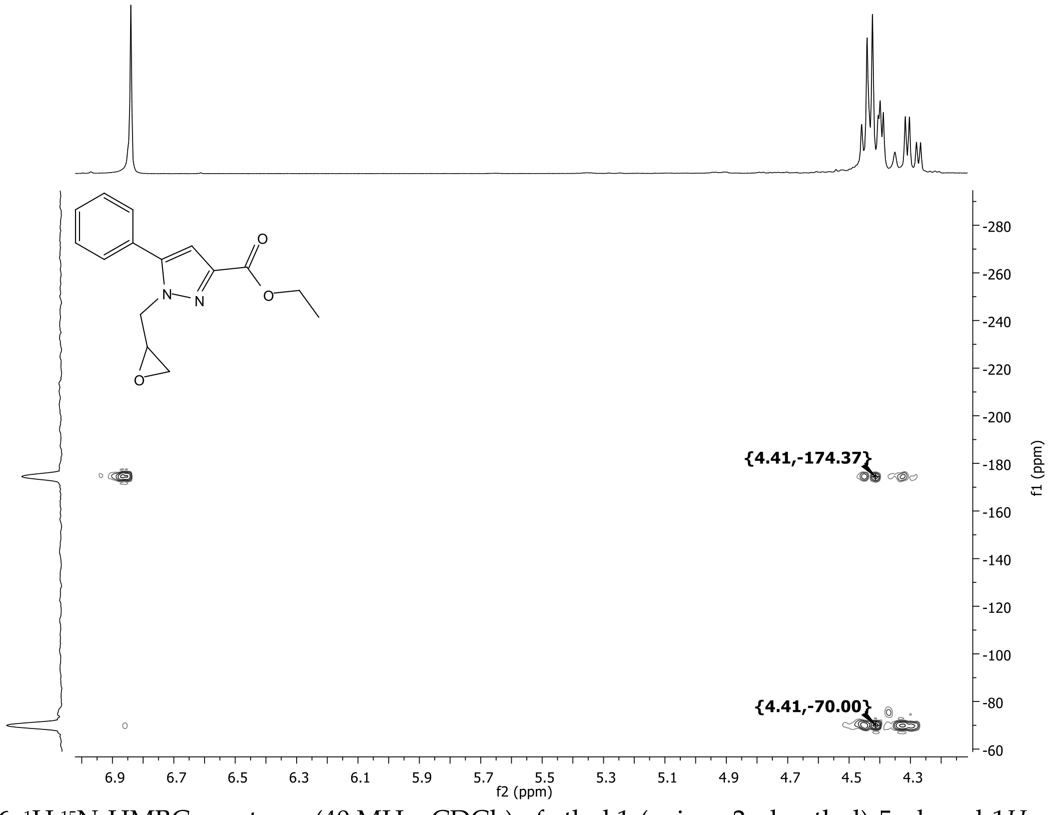

Figure S36. $^{1}H,^{15}N$-HMBC spectrum (40 MHz, $CDCl_3$) of ethyl 1-(oxiran-2-ylmethyl)-5-phenyl-1*H*-pyrazole-3-carboxylate (**3a**).

Compound Spectrum SmartFormula Report

Analysis Info

Analysis Name	D:\Data\KDD-273-1.d	Acquisition Date	12/28/2021 4:34:32 PM
Method	DirectInfusion_TuneLow_pos.m		
Sample Name	KDD-273-1	Operator	hplc
Comment	AB	Instrument	micrOTOF-Q III 8228888.20448

Acquisition Parameter

Source Type	ESI	Ion Polarity	Positive	Set Nebulizer	0.4 Bar
Focus	Not active	Set Capillary	4500 V	Set Dry Heater	180 °C
Scan Begin	50 m/z	Set End Plate Offset	-500 V	Set Dry Gas	4.0 l/min
Scan End	1000 m/z	Set Collision Cell RF	140.0 Vpp	Set Divert Valve	Waste

#	RT [min]	Area	Int. Type	I	S/N	Chromatogram	Max. m/z	FWHM [min]
n.a.	5.4	n.a.	Single spectrum	n.a.	n.a.	n.a.	295.1051	n.a.

+MS, 5.4min #324

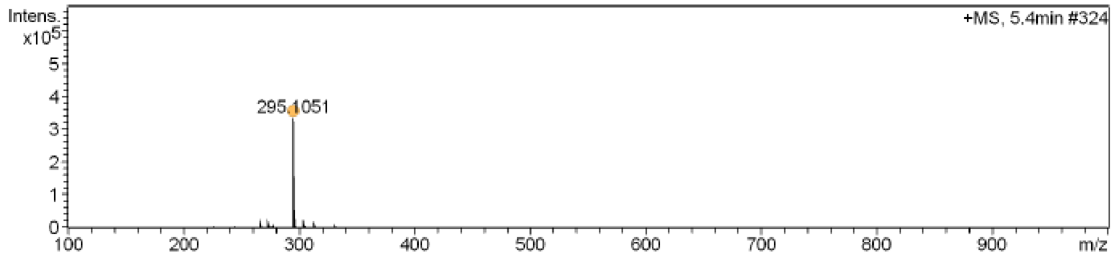

Meas. m/z	#	Ion Formula	m/z	err [ppm]	mSigma	# Sigma	Score	rdb	e⁻ Conf	N-Rule
295.1051	1	C15H16N2NaO3	295.1053	0.8	2.6	1	100.00	8.5	even	ok

Figure S37. HRMS (ESI-TOF) spectrum of ethyl 1-(oxiran-2-ylmethyl)-5-phenyl-1*H*-pyrazole-3-carboxylate (**3a**).

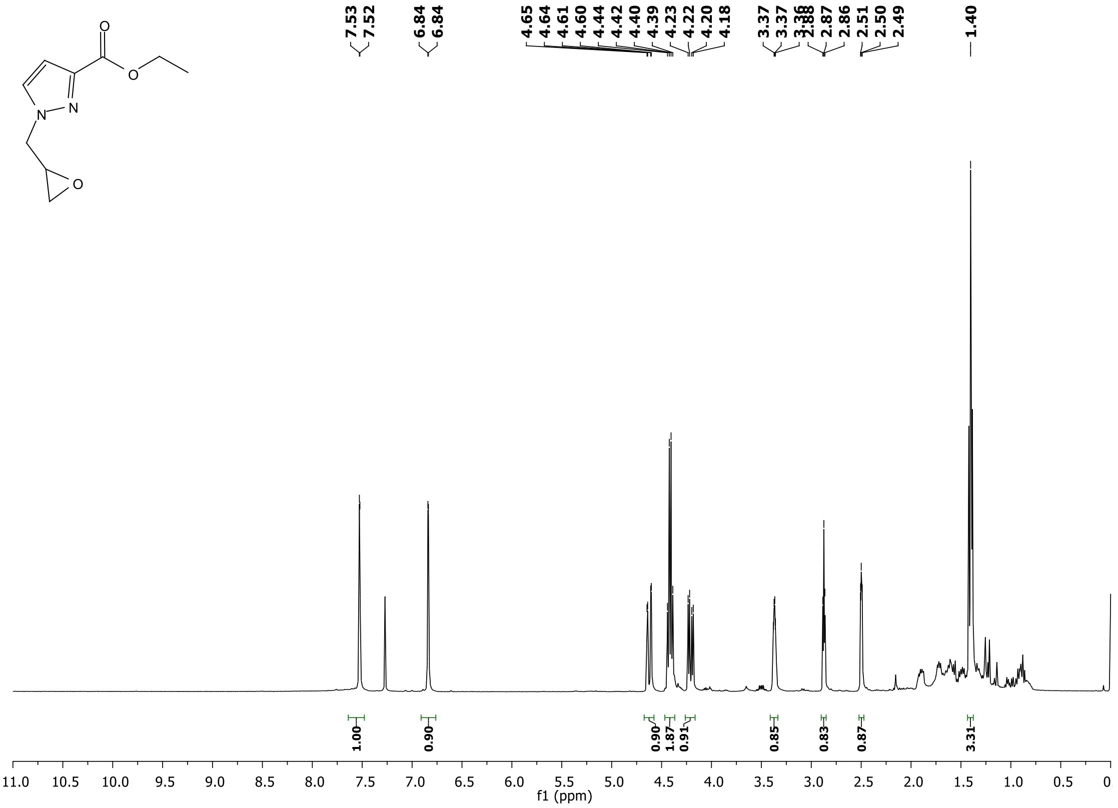

Figure S38. ^{1}H NMR spectrum (400 MHz, $CDCl_3$) of ethyl 1-(oxiran-2-ylmethyl)-1*H*-pyrazole-3-carboxylate (**3f**).

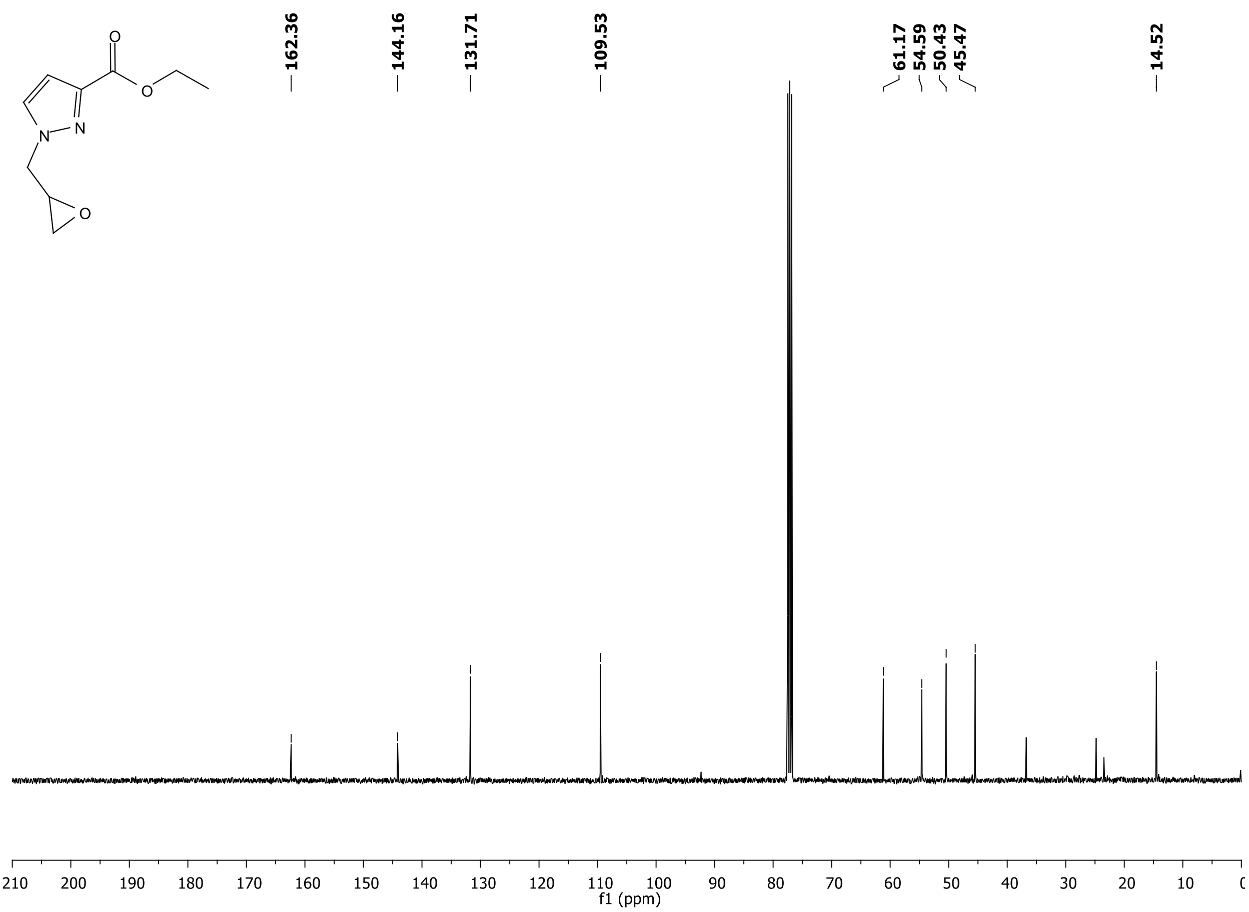

Figure S39. ^{13}C NMR spectrum (101 MHz, $CDCl_3$) of ethyl 1-(oxiran-2-ylmethyl)-1*H*-pyrazole-3-carboxylate (**3f**).

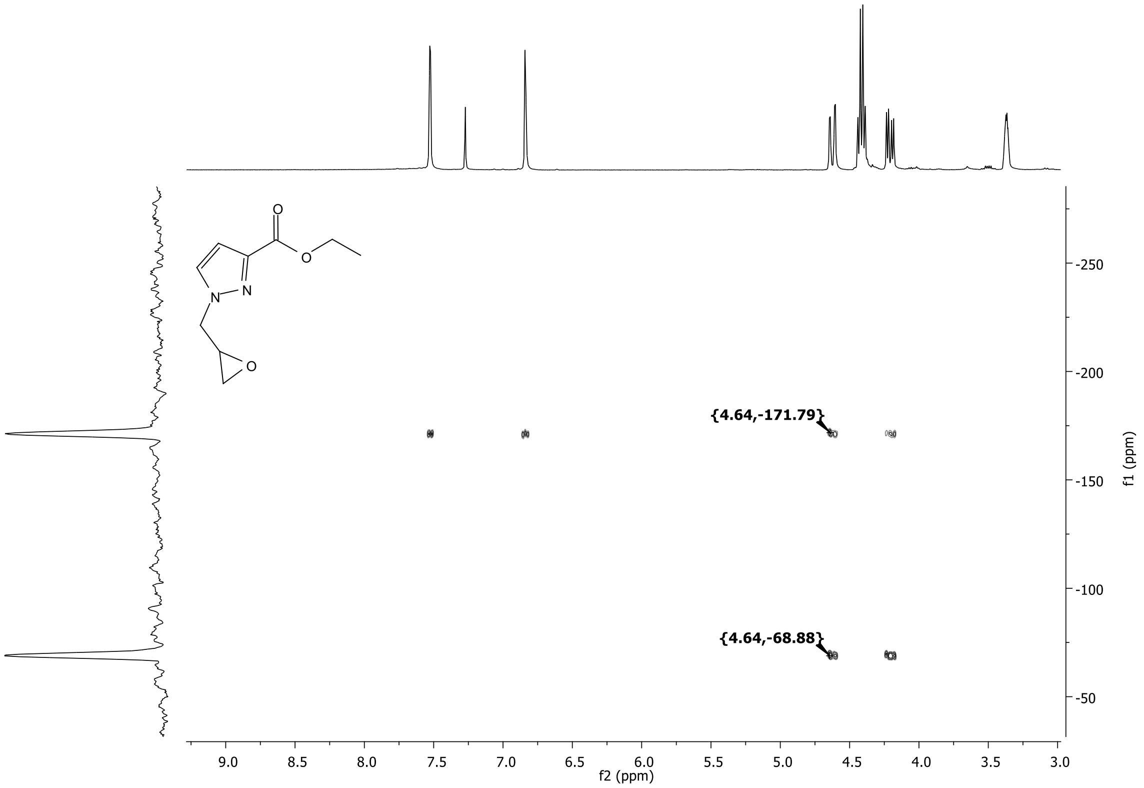

Figure S40. ^{1}H,^{15}N-HMBC spectrum (40 MHz, $CDCl_3$) of ethyl 1-(oxiran-2-ylmethyl)-1*H*-pyrazole-3-carboxylate (**3f**).

Qualitative Compound Report

**Data File**	221114_MV-41-5_02.d	**Sample Name**	MV-41-5
**Sample Type**	Sample	**Position**	
**Instrument Name**	G6230B TOF	**User Name**	
**Acq Method**	HRMS_12min_ref.m	**Acquired Time**	14-Nov-22 8:20:02 AM
**IRM Calibration Status**	Success	**DA Method**	test.m
**Comment**			
**Sample Group**		**Info.**	
**Stream Name**		**Acquisition SW Version**	6200 series TOF/6500 series Q-TOF B.09.00 (B9044.1 SP1)

Compound Table

Compound Label	RT	Mass	Abund	Formula	Tgt Mass	Diff (ppm)
Cpd 1: C9 H12 N2 O3	7.019	196.0848	3864973	C9 H12 N2 O3	196.0848	0.1

Compound Label	*m/z*	RT	Algorithm	Mass
Cpd 1: C9 H12 N2 O3	197.0915	7.019	Find By Formula	196.0848

Compound Chromatograms

MS Spectrum

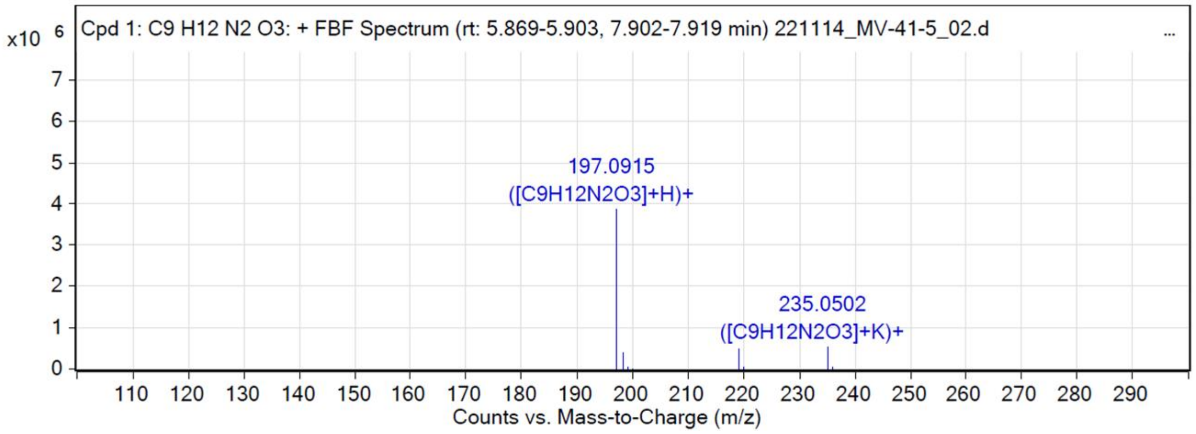

Figure S41. HRMS (ESI-TOF) spectrum of ethyl 1-(oxiran-2-ylmethyl)-1*H*-pyrazole-3-carboxylate (**3f**).

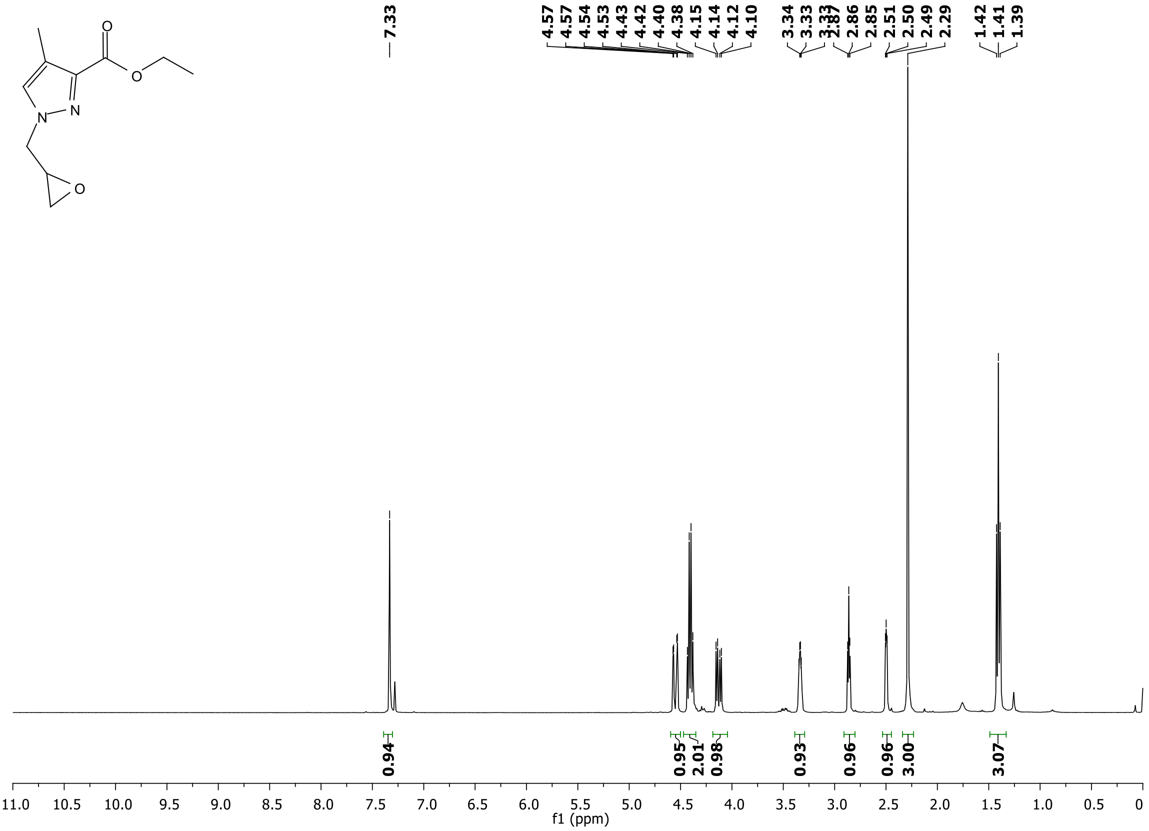

Figure S42. ^{1}H NMR spectrum (400 MHz, $CDCl_3$) of Ethyl 4-methyl-1-(oxiran-2-ylmethyl)-1*H*-pyrazole-3-carboxylate (**3g**).

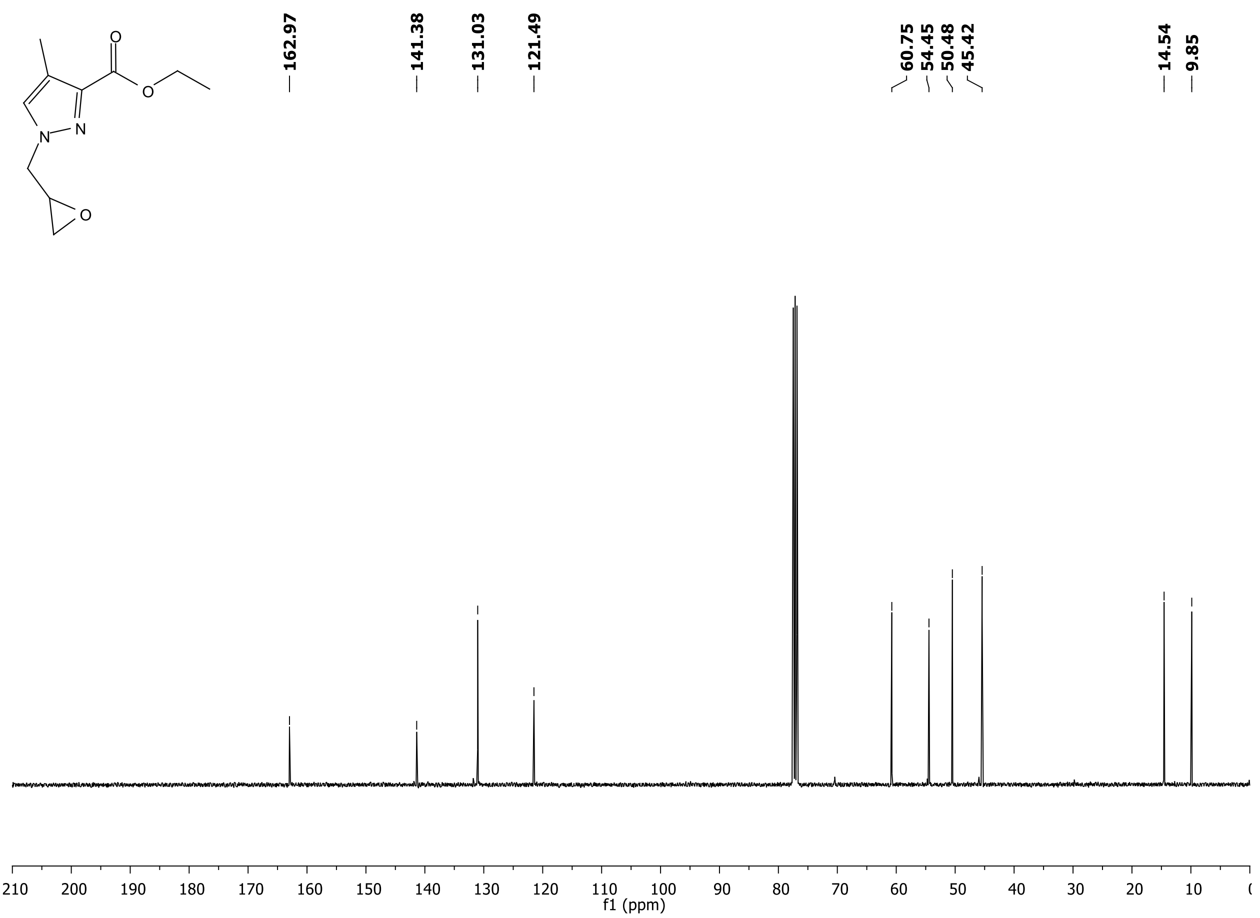

Figure S43. ^{13}C NMR spectrum (101 MHz, $CDCl_3$) of ethyl 4-methyl-1-(oxiran-2-ylmethyl)-1*H*-pyrazole-3-carboxylate (**3g**).

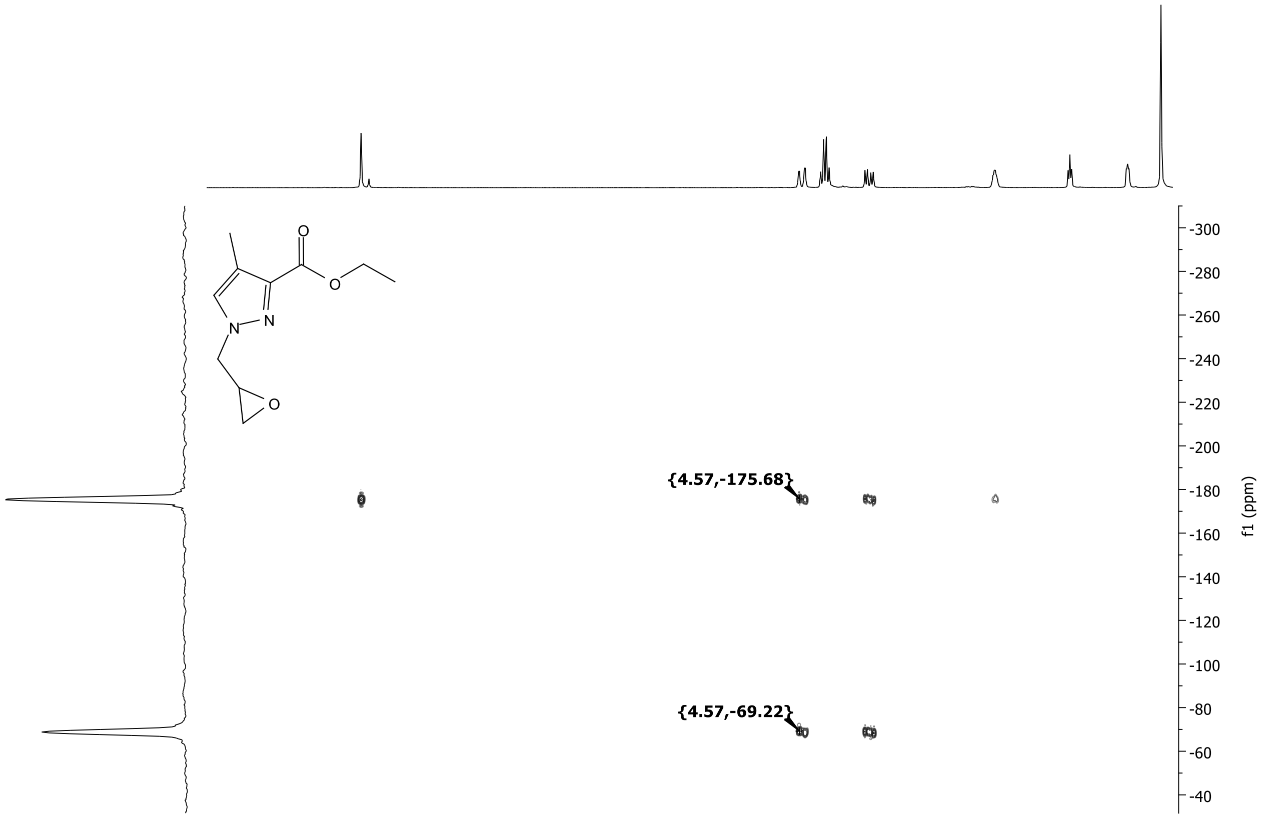

Figure S44. $^{1}H,^{15}N$-HMBC spectrum (40 MHz, $CDCl_3$) of ethyl 4-methyl-1-(oxiran-2-ylmethyl)-1*H*-pyrazole-3-carboxylate (**3g**).

Qualitative Compound Report

Data File	221111_MV-45-2_01.d	Sample Name	MV-45-2
Sample Type	Sample	Position	
Instrument Name	G6230B TOF	User Name	
Acq Method	HRMS_12min_ref.m	Acquired Time	11-Nov-22 11:24:23 AM
IRM Calibration Status	Success	DA Method	test.m
Comment			
Sample Group		Info.	
Stream Name		Acquisition SW Version	6200 series TOF/6500 series Q-TOF B.09.00 (B9044.1 SP1)

Compound Table

Compound Label	RT	Mass	Abund	Formula	Tgt Mass	Diff (ppm)
Cpd 1: C10 H14 N2 O3	6.784	210.1005	4040960	C10 H14 N2 O3	210.1004	0.36

Compound Label	m/z	RT	Algorithm	Mass
Cpd 1: C10 H14 N2 O3	211.1072	6.784	Find By Formula	210.1005

Compound Chromatograms

MS Spectrum

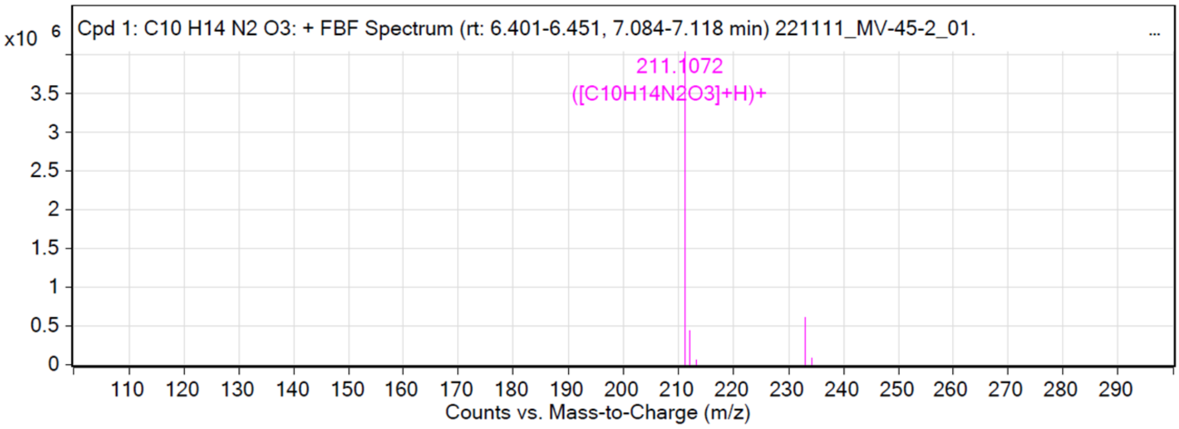

Figure S45. HRMS (ESI-TOF) spectrum of ethyl 4-methyl-1-(oxiran-2-ylmethyl)-1*H*-pyrazole-3-carboxylate (**3g**).

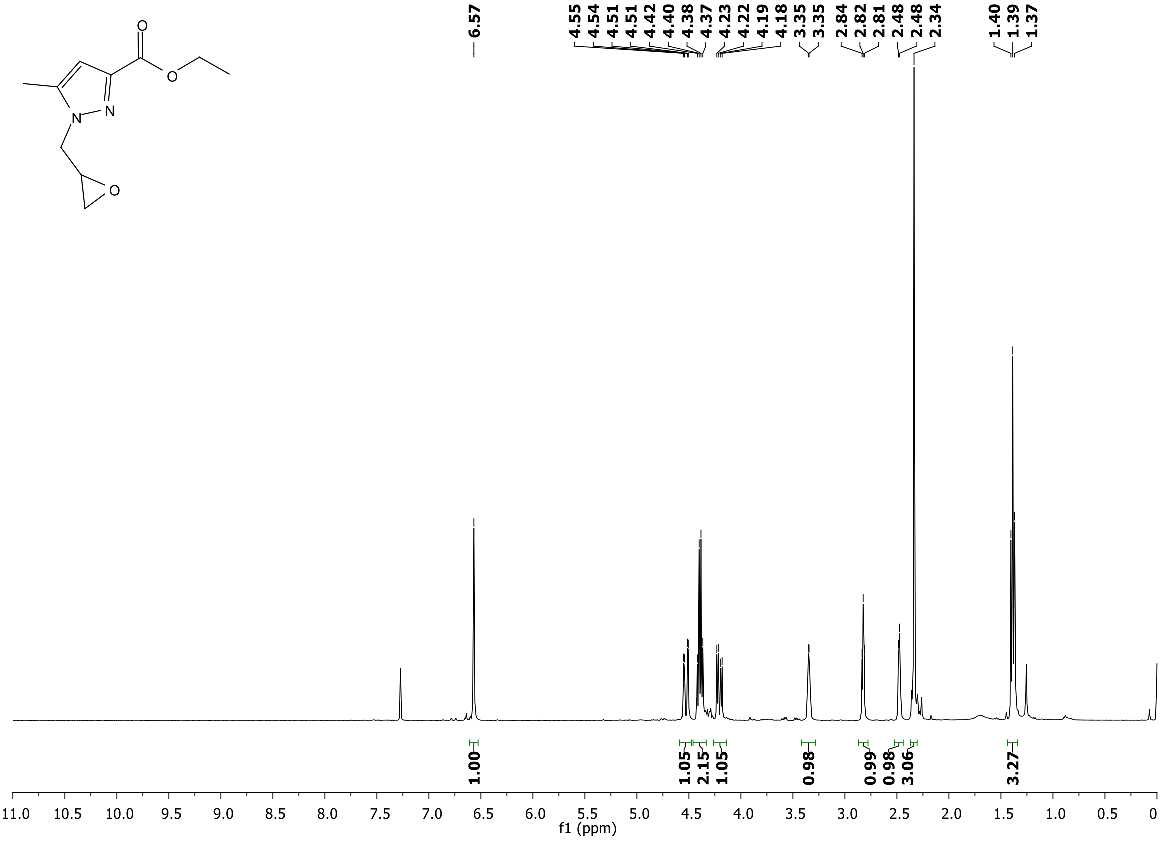

Figure S46. ^{1}H NMR spectrum (400 MHz, $CDCl_3$) of ethyl 5-methyl-1-(oxiran-2-ylmethyl)-1*H*-pyrazole-3-carboxylate (**3h**).

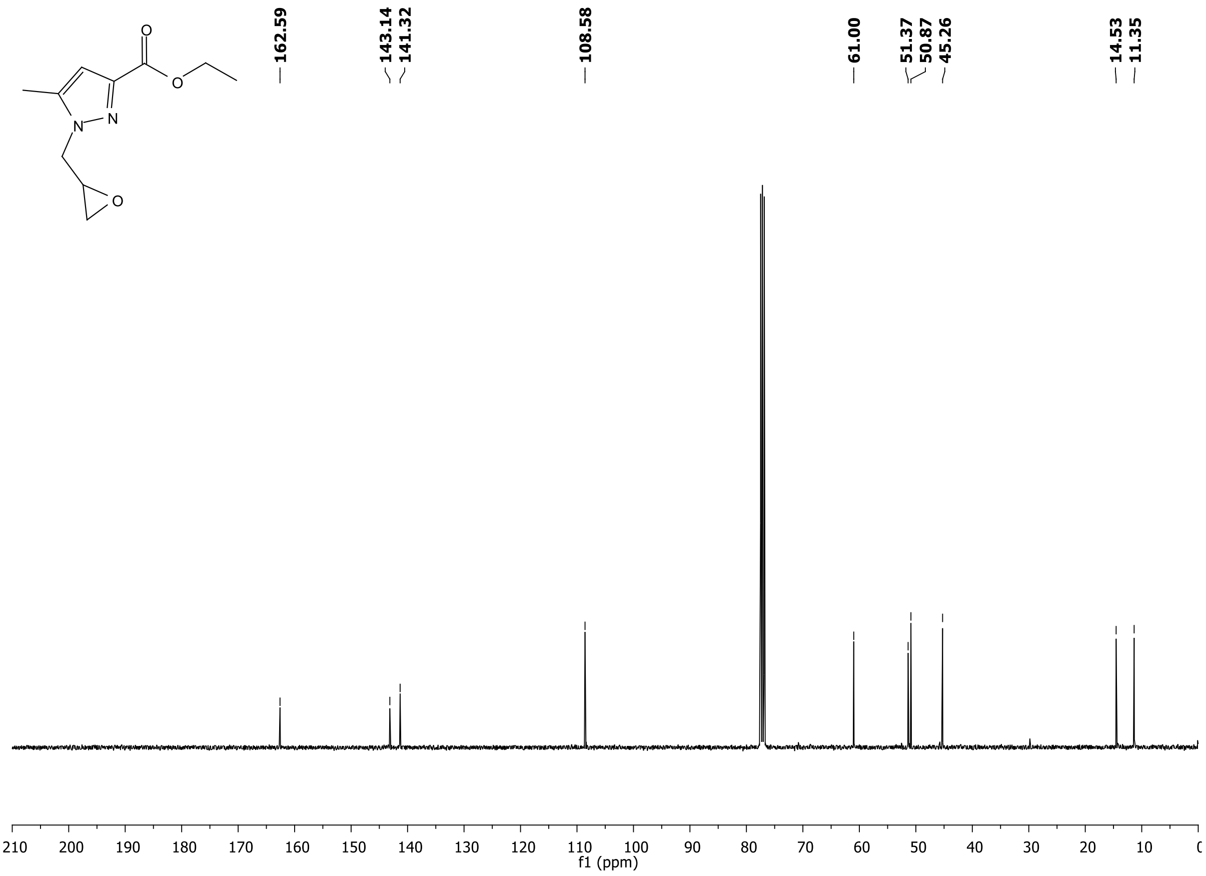

Figure S47. ^{13}C NMR spectrum (101 MHz, $CDCl_3$) of ethyl 5-methyl-1-(oxiran-2-ylmethyl)-1*H*-pyrazole-3-carboxylate (**3h**).

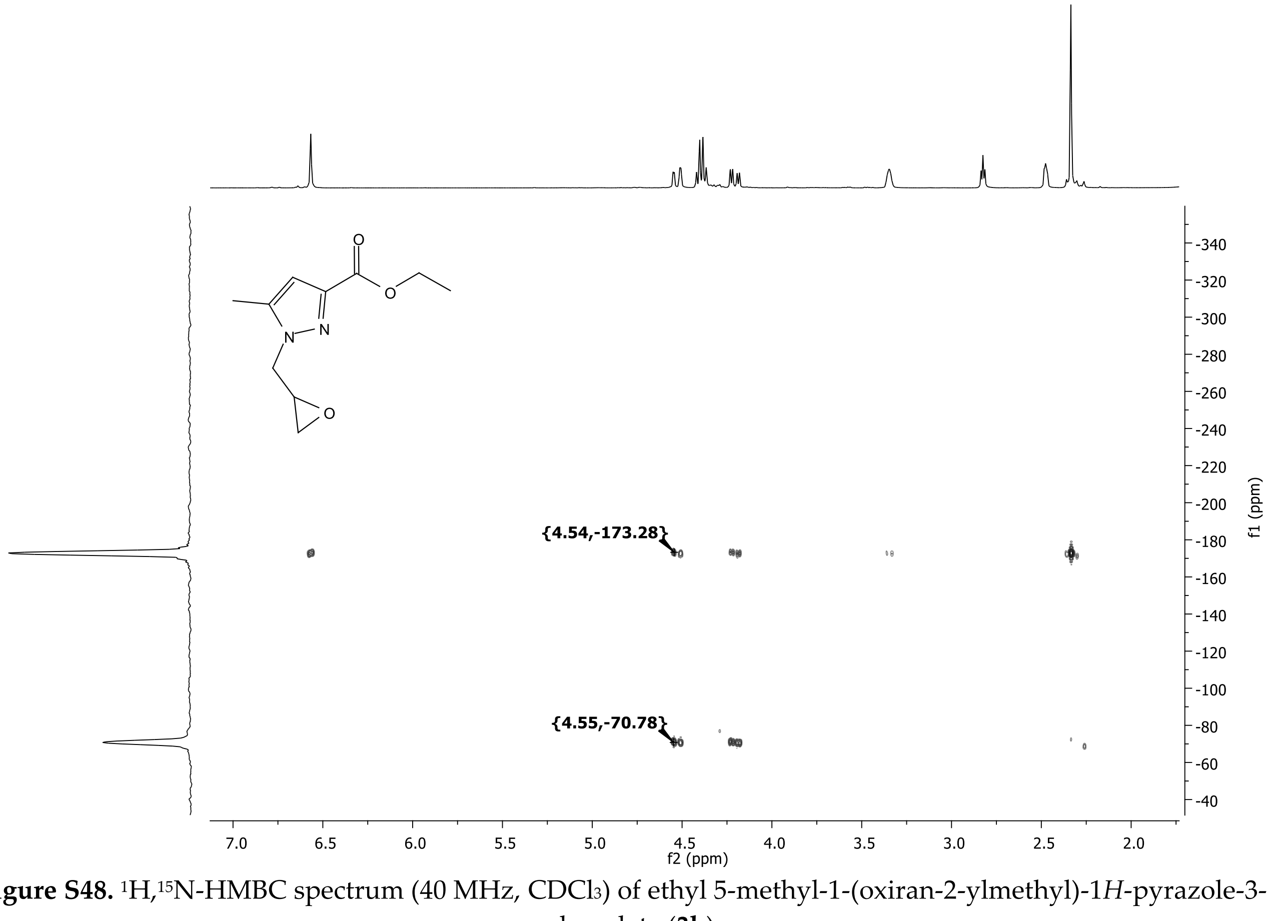

Figure S48. $^{1}H,^{15}N$-HMBC spectrum (40 MHz, $CDCl_3$) of ethyl 5-methyl-1-(oxiran-2-ylmethyl)-1*H*-pyrazole-3-carboxylate (**3h**).

Qualitative Compound Report

**Data File**	221114_MV-47-3_01.d	**Sample Name**	MV-47-3
**Sample Type**	Sample	**Position**	
**Instrument Name**	G6230B TOF	**User Name**	
**Acq Method**	HRMS_12min_ref.m	**Acquired Time**	14-Nov-22 9:05:33 AM
**IRM Calibration Status**	Success	**DA Method**	test.m
**Comment**			
**Sample Group**		**Info.**	
**Stream Name**		**Acquisition SW Version**	6200 series TOF/6500 series Q-TOF B.09.00 (B9044.1 SP1)

Compound Table

Compound Label	RT	Mass	Abund	Formula	Tgt Mass	Diff (ppm)
Cpd 1: C10 H14 N2 O3	6.758	210.1005	2825605	C10 H14 N2 O3	210.1004	0.44

Compound Label	*m/z*	RT	Algorithm	Mass
Cpd 1: C10 H14 N2 O3	211.107	6.758	Find By Formula	210.1005

Compound Chromatograms

MS Spectrum

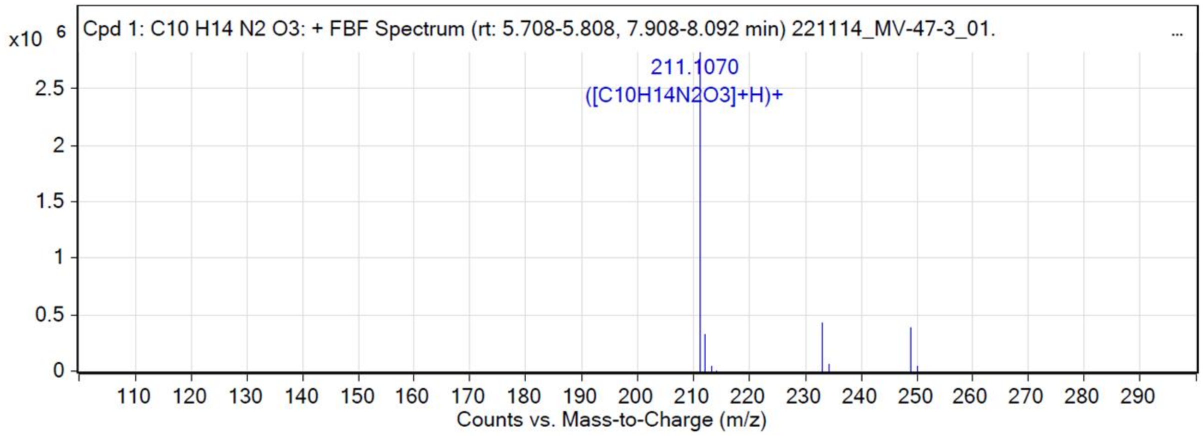

Figure S49. HRMS (ESI-TOF) spectrum of ethyl 5-methyl-1-(oxiran-2-ylmethyl)-1*H*-pyrazole-3-carboxylate (**3h**).

2. Data of 7-hydroxy-5,6,7,8-tetrahydro-4*H*-pyrazolo[1,5-*a*][1,4]diazepin-4-ones (4a–x)

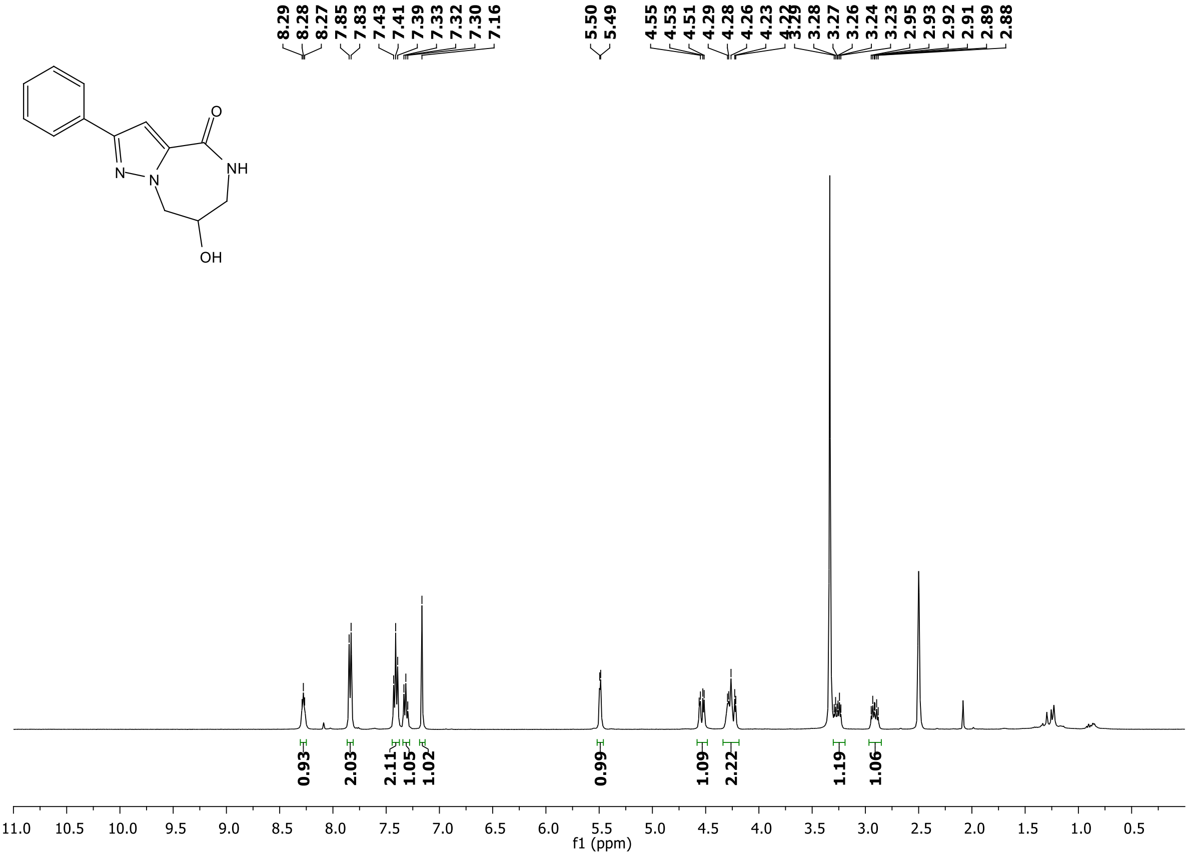

Figure S50. ^{1}H NMR spectrum (400 MHz, DMSO-d_6) of 7-hydroxy-2-phenyl-5,6,7,8-tetrahydro-4*H*-pyrazolo [1,5-*a*][1,4]diazepin-4-one (**4a**).

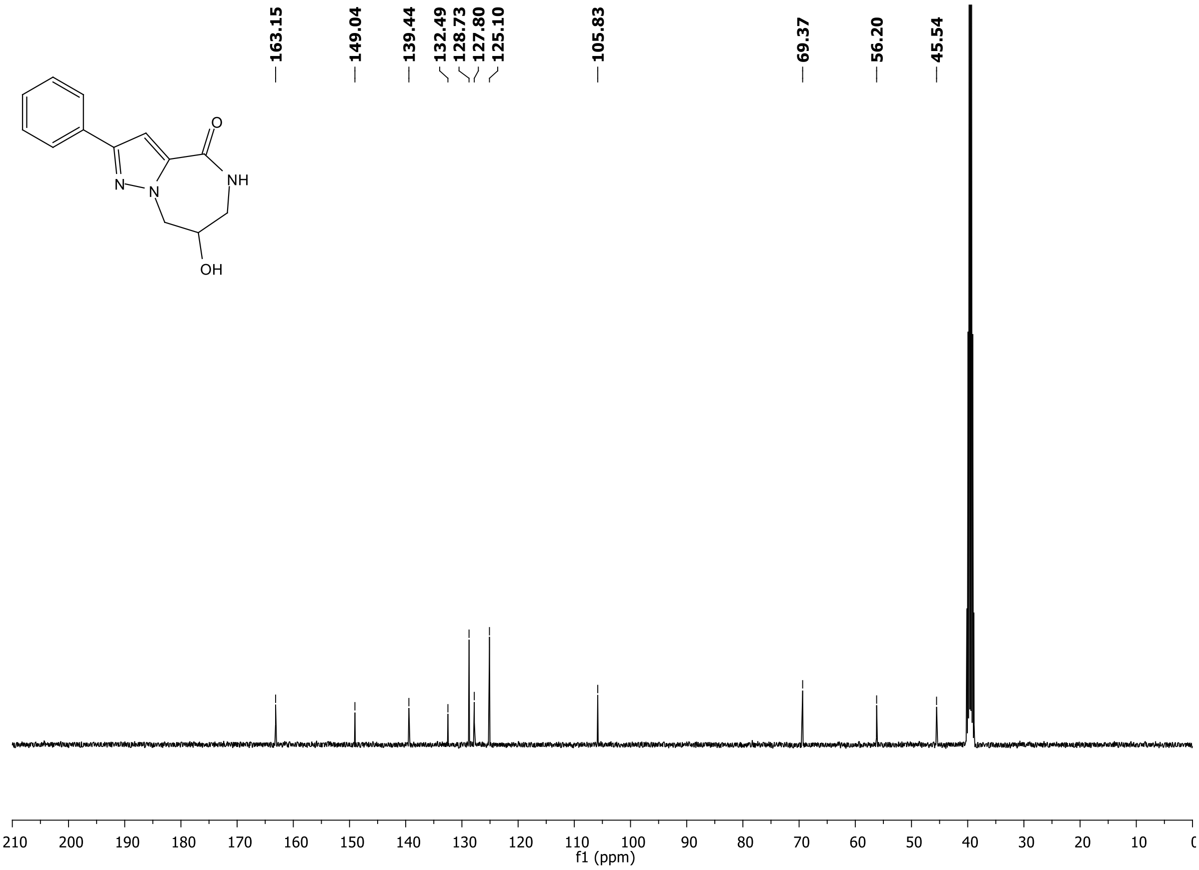

Figure S51. ^{13}C NMR spectrum (101 MHz, DMSO-d_6) of 7-hydroxy-2-phenyl-5,6,7,8-tetrahydro-4*H*-pyrazolo [1,5-*a*][1,4]diazepin-4-one (**4a**).

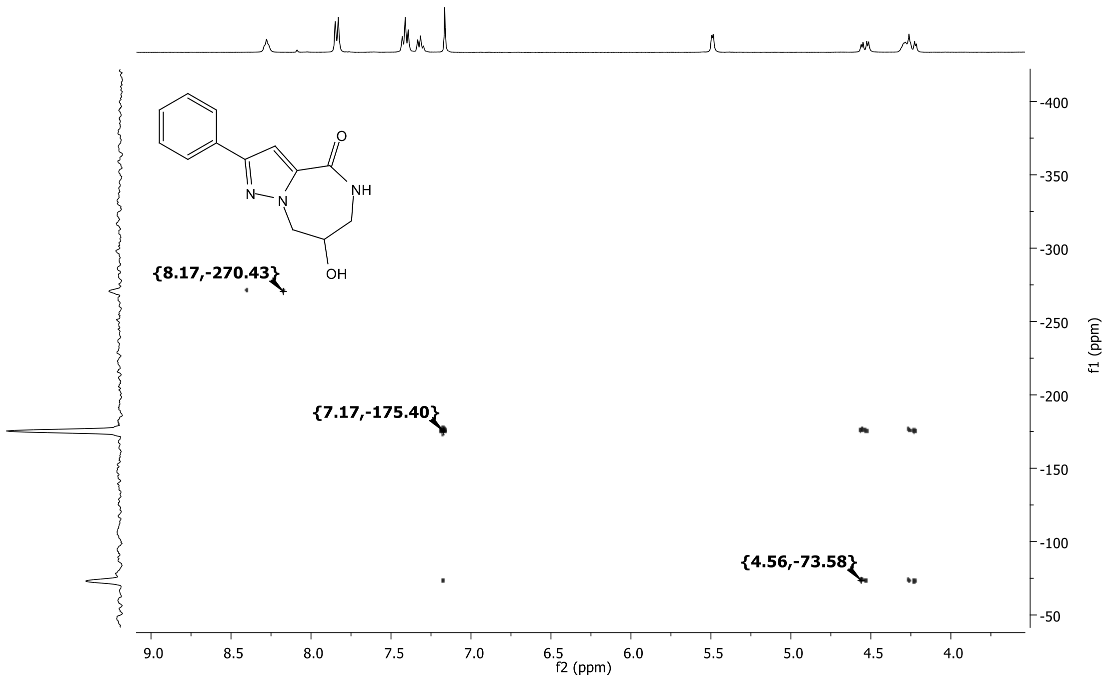

Figure S52. $^{1}H,^{15}N$-HMBC (40 MHz, DMSO-d_6) of 7-hydroxy-2-phenyl-5,6,7,8-tetrahydro-4*H*-pyrazolo[1,5-*a*][1,4]diazepin-4-one (**4a**).

Compound Spectrum SmartFormula Report

Analysis Info

Analysis Name	D:\Data\KDD-065.d	Acquisition Date	3/30/2020 4:36:29 PM
Method	DirectInfusion_TuneLow_pos.m	Operator	hplc
Sample Name	KDD-065	Instrument	microTOF-Q III 8228888.20448
Comment	SB		

Acquisition Parameter

Source Type	ESI	Ion Polarity	Positive	Set Nebulizer	0.4 Bar
Focus	Not active	Set Capillary	4500 V	Set Dry Heater	180 °C
Scan Begin	50 m/z	Set End Plate Offset	-500 V	Set Dry Gas	4.0 l/min
Scan End	1000 m/z	Set Collision Cell RF	140.0 Vpp	Set Divert Valve	Waste

#	RT [min]	Area	Int. Type	I	S/N	Chromatogram	Max. m/z	FWHM [min]
n.a.	0.0	n.a.	Single spectrum	n.a.	n.a.	n.a.	226.9519	n.a.
n.a.	5.3	n.a.	Single spectrum	n.a.	n.a.	n.a.	266.0900	n.a.

+MS, 5.3min #317

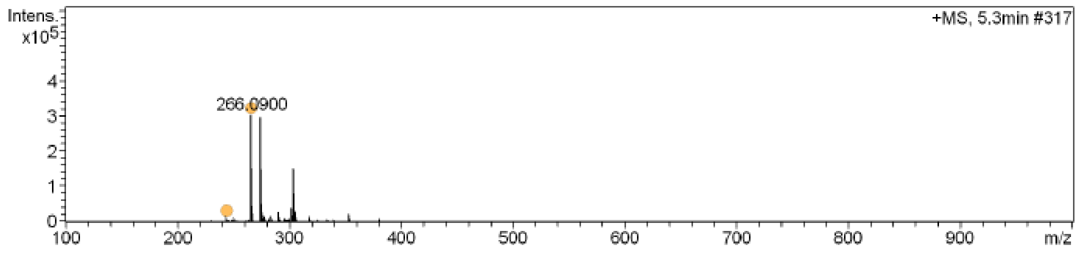

Meas. m/z	#	Ion Formula	m/z	err [ppm]	mSigma	# Sigma	Score	rdb	e⁻ Conf	N-Rule
244.1084	1	C13H14N3O2	244.1081	1.4	22.8	1	100.00	8.5	even	ok
266.0900	1	C13H13N3NaO2	266.0900	-0.1	3.4	1	100.00	8.5	even	ok

Figure S53. HRMS (ESI-TOF) spectrum of 7-hydroxy-2-phenyl-5,6,7,8-tetrahydro-4*H*-pyrazolo[1,5-*a*][1,4]diazepin-4-one (**4a**).

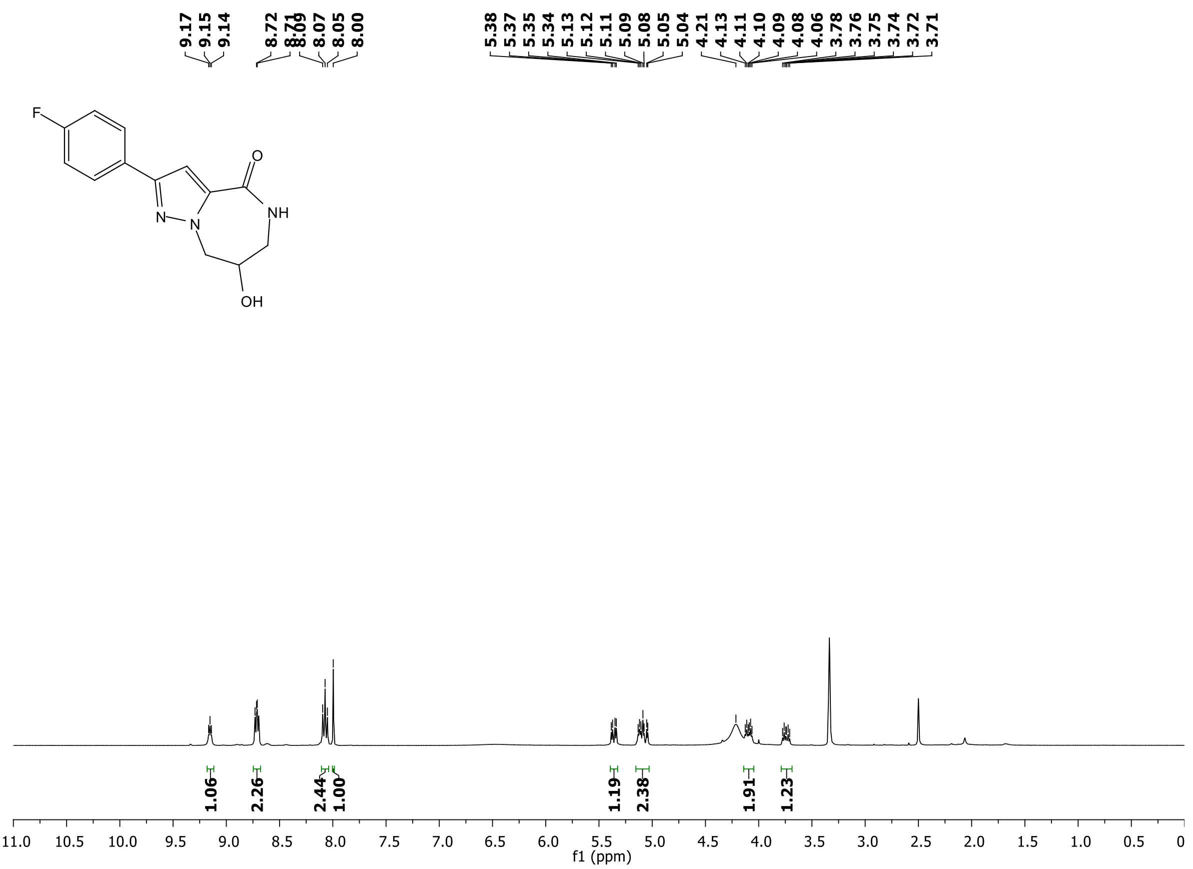

Figure S54. ^{1}H NMR spectrum (400 MHz, DMSO-d_6) of 2-(4-fluorophenyl)-7-hydroxy-5,6,7,8-tetrahydro-4*H*-pyrazolo [1,5-*a*][1,4]diazepin-4-one (**4b**).

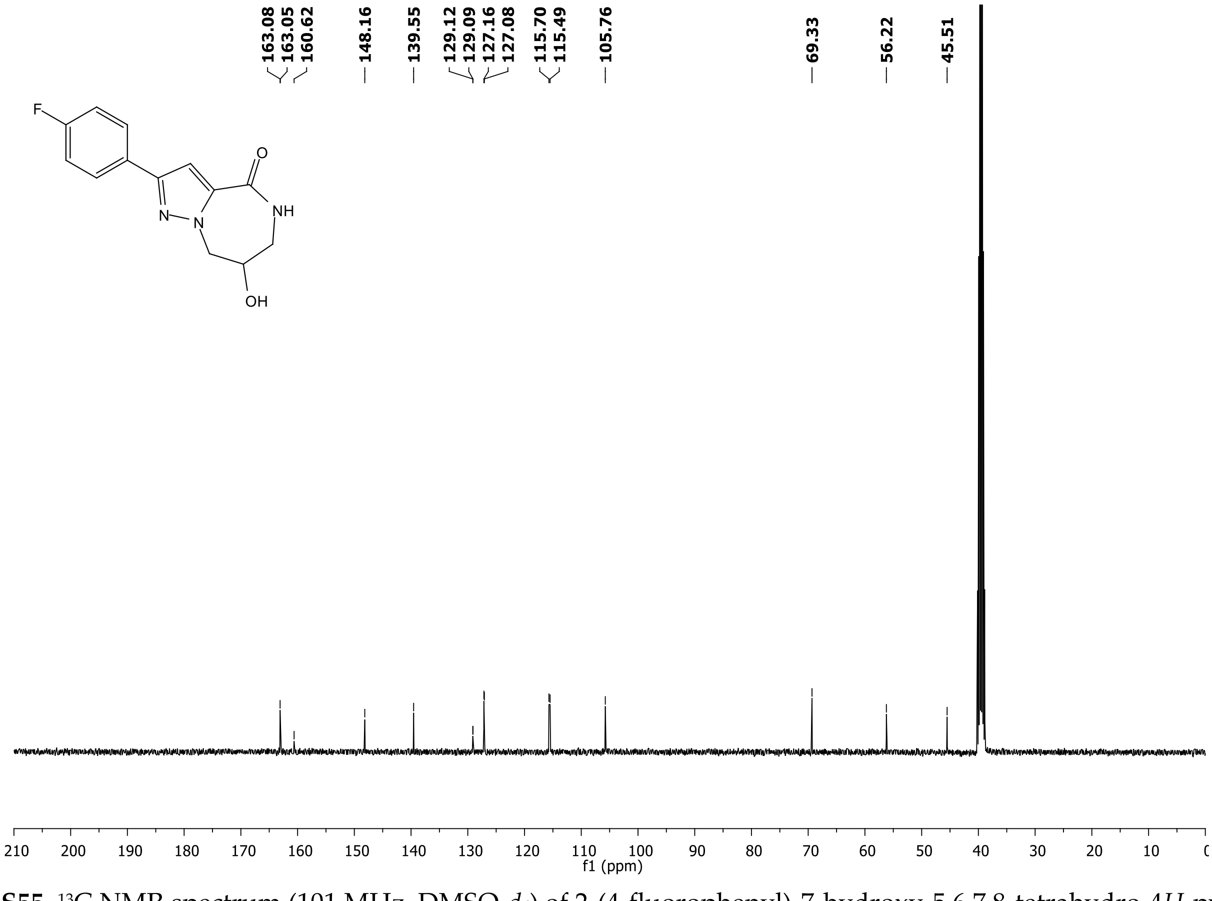

Figure S55. ^{13}C NMR spectrum (101 MHz, DMSO-d_6) of 2-(4-fluorophenyl)-7-hydroxy-5,6,7,8-tetrahydro-4*H*-pyrazolo [1,5-*a*][1,4]diazepin-4-one (**4b**).

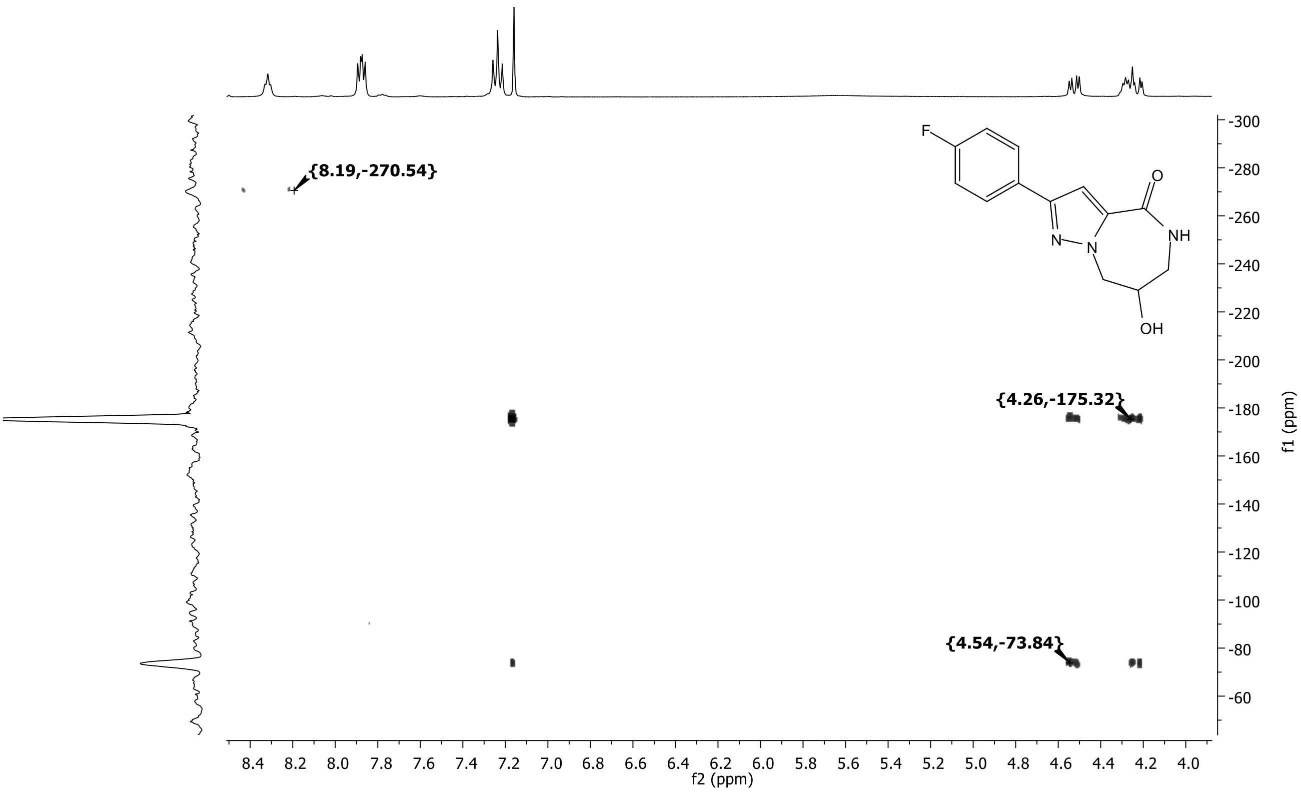

Figure S56. $^{1}H,^{15}N$-HMBC (40 MHz, DMSO-d_6) of 2-(4-fluorophenyl)-7-hydroxy-5,6,7,8-tetrahydro-4*H*-pyrazolo [1,5-*a*][1,4]diazepin-4-one (**4b**).

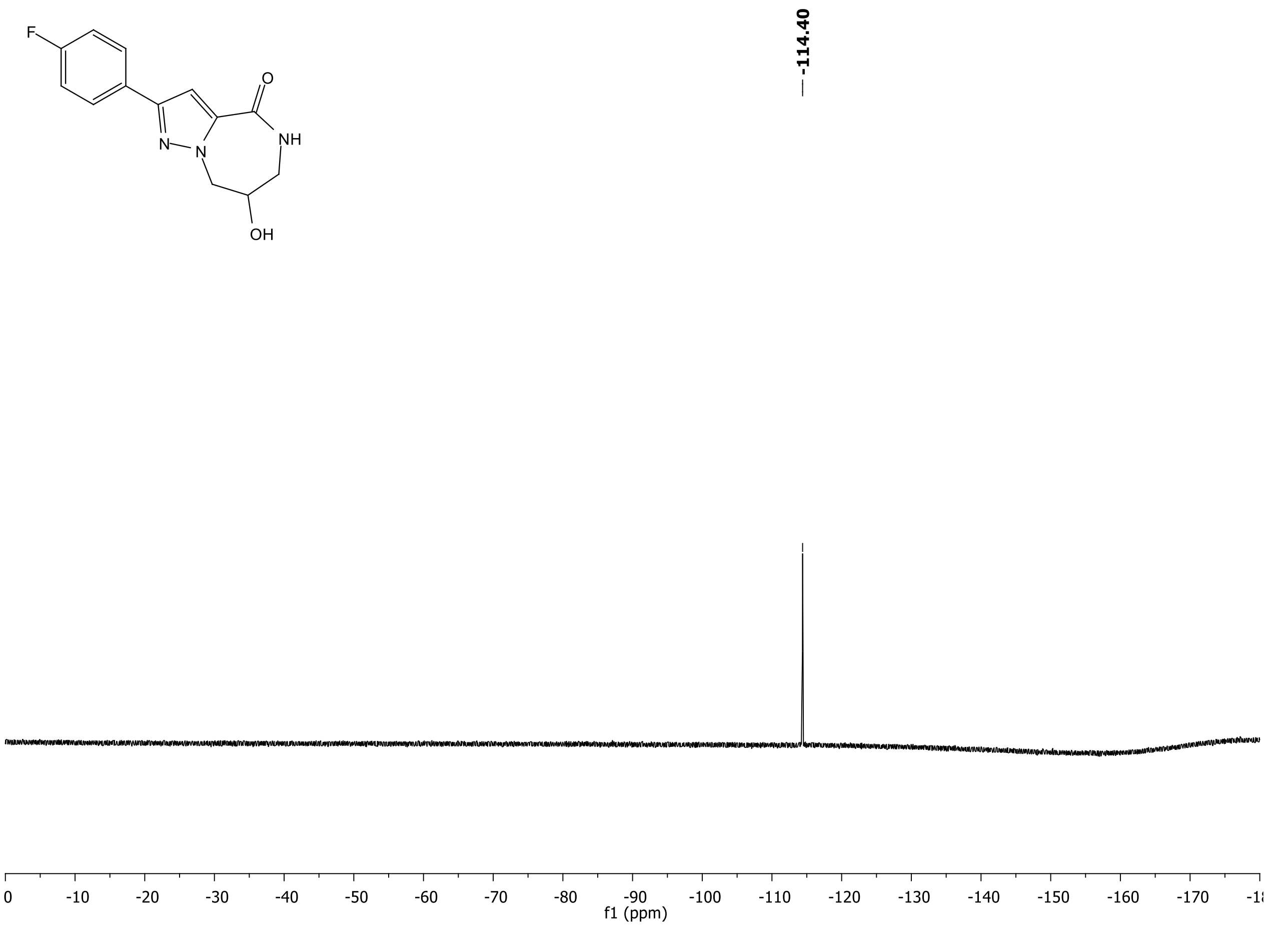

Figure S57. ^{19}F NMR spectrum (376 MHz, DMSO-d_6) of 2-(4-fluorophenyl)-7-hydroxy-5,6,7,8-tetrahydro-4*H*-pyrazolo [1,5-*a*][1,4]diazepin-4-one (**4b**).

Compound Spectrum SmartFormula Report

Analysis Info

Analysis Name	D:\Data\KDD-124.d	Acquisition Date	4/6/2020 2:23:35 PM
Method	DirectInfusion_TuneLow_pos.m	Operator	hplc
Sample Name	KDD-124	Instrument	micrOTOF-Q III 8228888.20448
Comment	SB		

Acquisition Parameter

Source Type	ESI	Ion Polarity	Positive	Set Nebulizer	0.4 Bar
Focus	Not active	Set Capillary	4500 V	Set Dry Heater	180 °C
Scan Begin	50 m/z	Set End Plate Offset	-500 V	Set Dry Gas	4.0 l/min
Scan End	1000 m/z	Set Collision Cell RF	140.0 Vpp	Set Divert Valve	Waste

#	RT [min]	Area	Int. Type	I	S/N	Chromatogram	Max. m/z	FWHM [min]
n.a.	1.0	n.a.	Single spectrum	n.a.	n.a.	n.a.	226.9518	n.a.
n.a.	6.7	n.a.	Single spectrum	n.a.	n.a.	n.a.	284.0806	n.a.

+MS, 6.7min #400

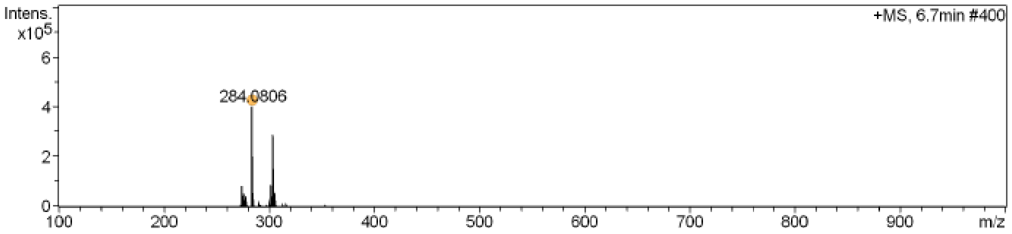

Meas. m/z	#	Ion Formula	m/z	err [ppm]	mSigma	# Sigma	Score	rdb	e⁻ Conf	N-Rule
284.0806	1	C13H12FN3NaO2	284.0806	0.2	7.3	1	100.00	8.5	even	ok

Figure S58. HRMS (ESI-TOF) spectrum of 2-(4-fluorophenyl)-7-hydroxy-5,6,7,8-tetrahydro-4*H*-pyrazolo[1,5-*a*][1,4]diazepin-4-one (**4b**).

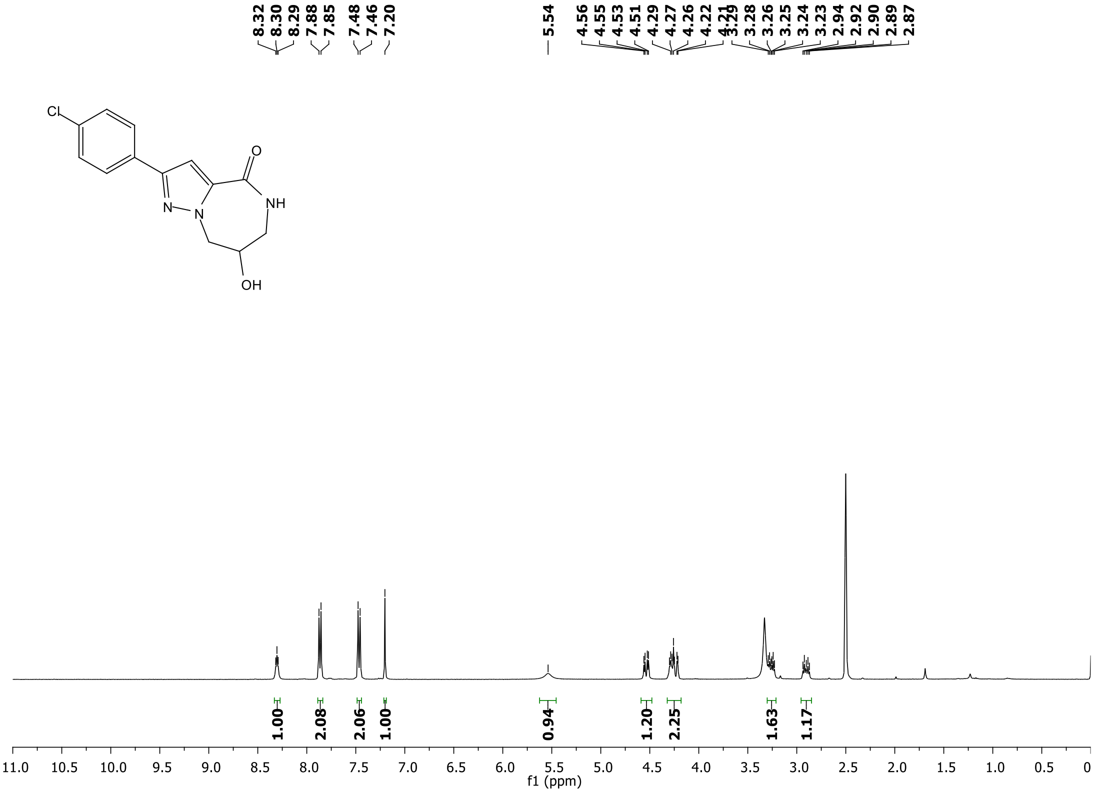

Figure S59. ^{1}H NMR spectrum (400 MHz, DMSO-*d*6) of 2-(4-chlorophenyl)-7-hydroxy-5,6,7,8-tetrahydro-4*H*-pyrazolo[1,5-*a*][1,4]diazepin-4-one (**4c**).

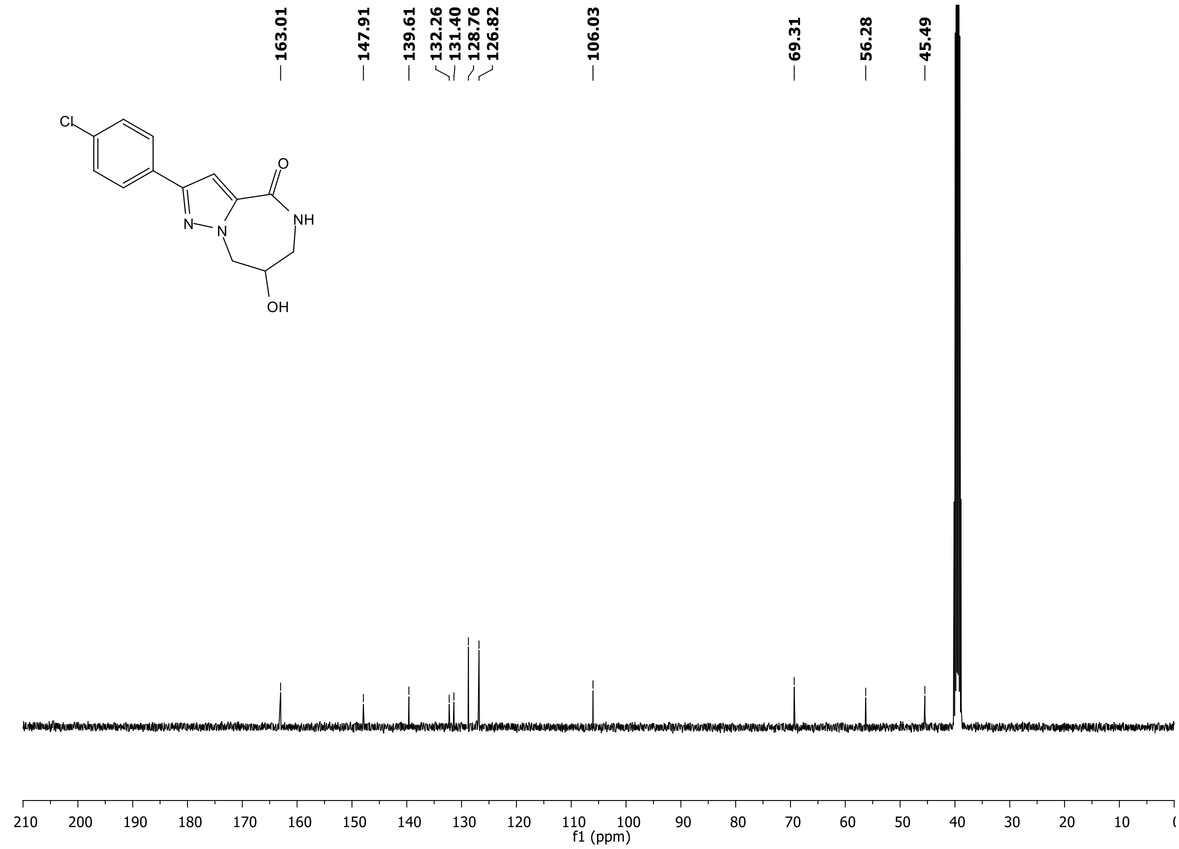

Figure S60. ^{13}C NMR spectrum (101 MHz, DMSO-d_6) of 2-(4-chlorophenyl)-7-hydroxy-5,6,7,8-tetrahydro-4*H*-pyrazolo [1,5-*a*][1,4]diazepin-4-one (**4c**).

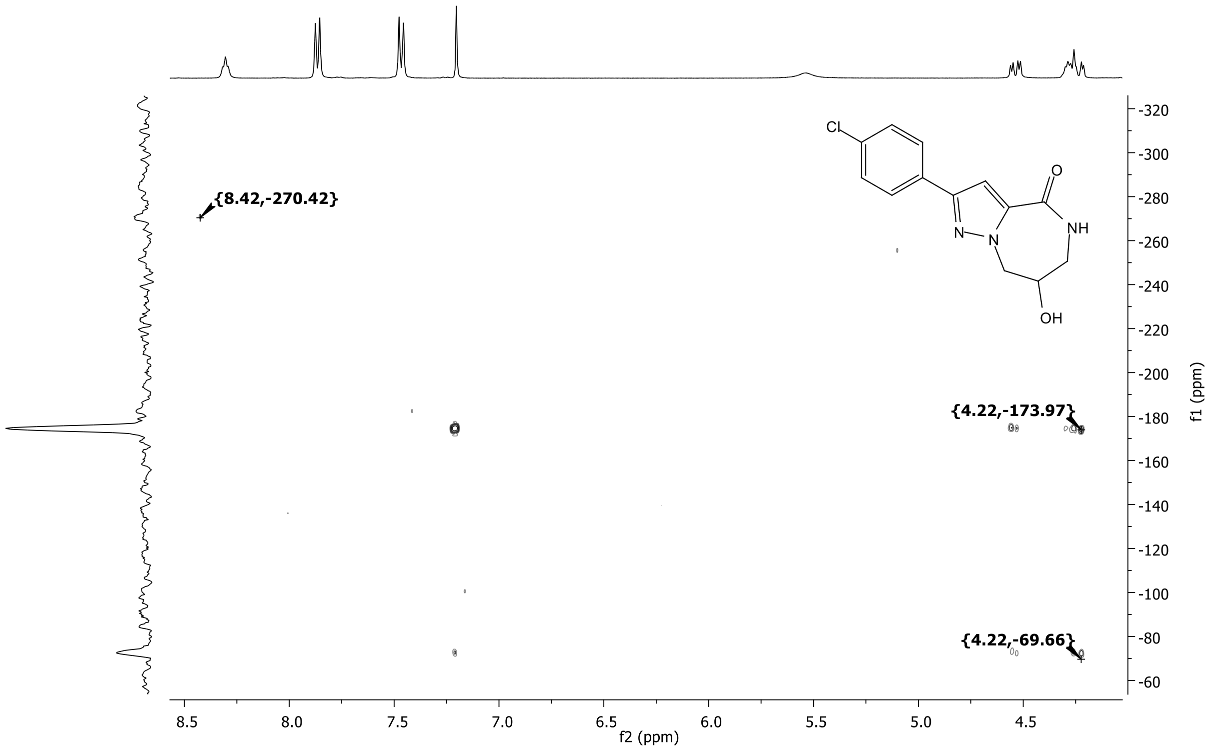

Figure S61. $^{1}H,^{15}N$-HMBC (40 MHz, DMSO-d_6) of 2-(4-chlorophenyl)-7-hydroxy-5,6,7,8-tetrahydro-4*H*-pyrazolo [1,5-*a*][1,4]diazepin-4-one (**4c**).

Compound Spectrum SmartFormula Report

Analysis Info

Analysis Name	D:\Data\KDD-123.d	Acquisition Date	4/6/2020 2:06:45 PM
Method	DirectInfusion_TuneLow_pos.m	Operator	hplc
Sample Name	KDD-123	Instrument	micrOTOF-Q III 8228888.20448
Comment	SB		

Acquisition Parameter

Source Type	ESI	Ion Polarity	Positive	Set Nebulizer	0.4 Bar
Focus	Not active	Set Capillary	4500 V	Set Dry Heater	180 °C
Scan Begin	50 m/z	Set End Plate Offset	-500 V	Set Dry Gas	4.0 l/min
Scan End	1000 m/z	Set Collision Cell RF	140.0 Vpp	Set Divert Valve	Waste

#	RT [min]	Area	Int. Type	I	S/N	Chromatogram	Max. m/z	FWHM [min]
n.a.	3.1	n.a.	Single spectrum	n.a.	n.a.	n.a.	226.9518	n.a.
n.a.	6.8	n.a.	Single spectrum	n.a.	n.a.	n.a.	304.2623	n.a.

+MS, 6.8min #406

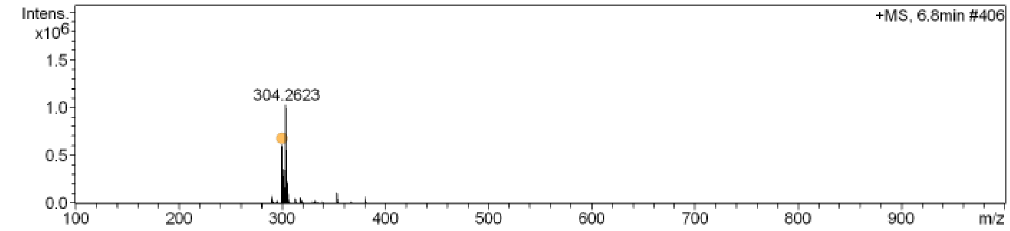

Meas. m/z	#	Ion Formula	m/z	err [ppm]	mSigma	# Sigma	Score	rdb	e⁻ Conf	N-Rule
300.0510	1	C13H12ClN3NaO2	300.0510	-0.1	7.6	1	100.00	8.5	even	ok

Figure S62. HRMS (ESI-TOF) spectrum of 2-(4-chlorophenyl)-7-hydroxy-5,6,7,8-tetrahydro-4*H*-pyrazolo [1,5-*a*][1,4]diazepin-4-one (**4c**).

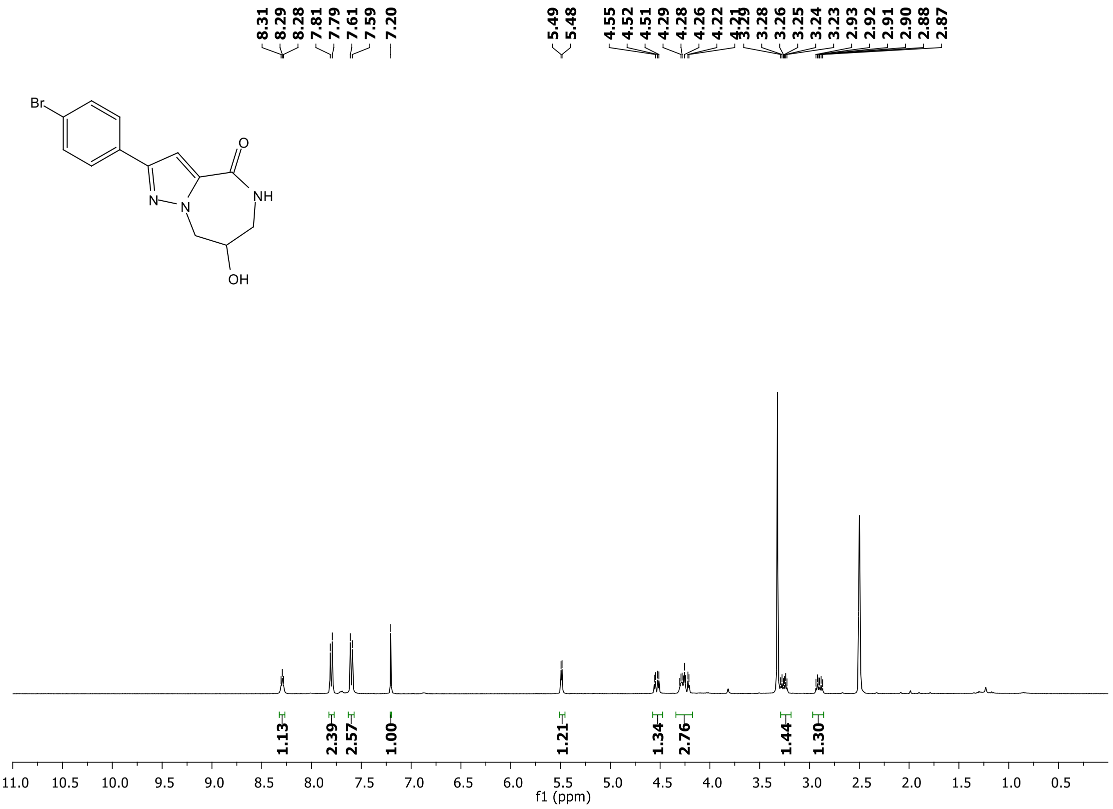

Figure S63. ¹H NMR spectrum (400 MHz, DMSO-d_6) of 2-(4-bromophenyl)-7-hydroxy-5,6,7,8-tetrahydro-4*H*-pyrazolo [1,5-*a*][1,4]diazepin-4-one (**4d**).

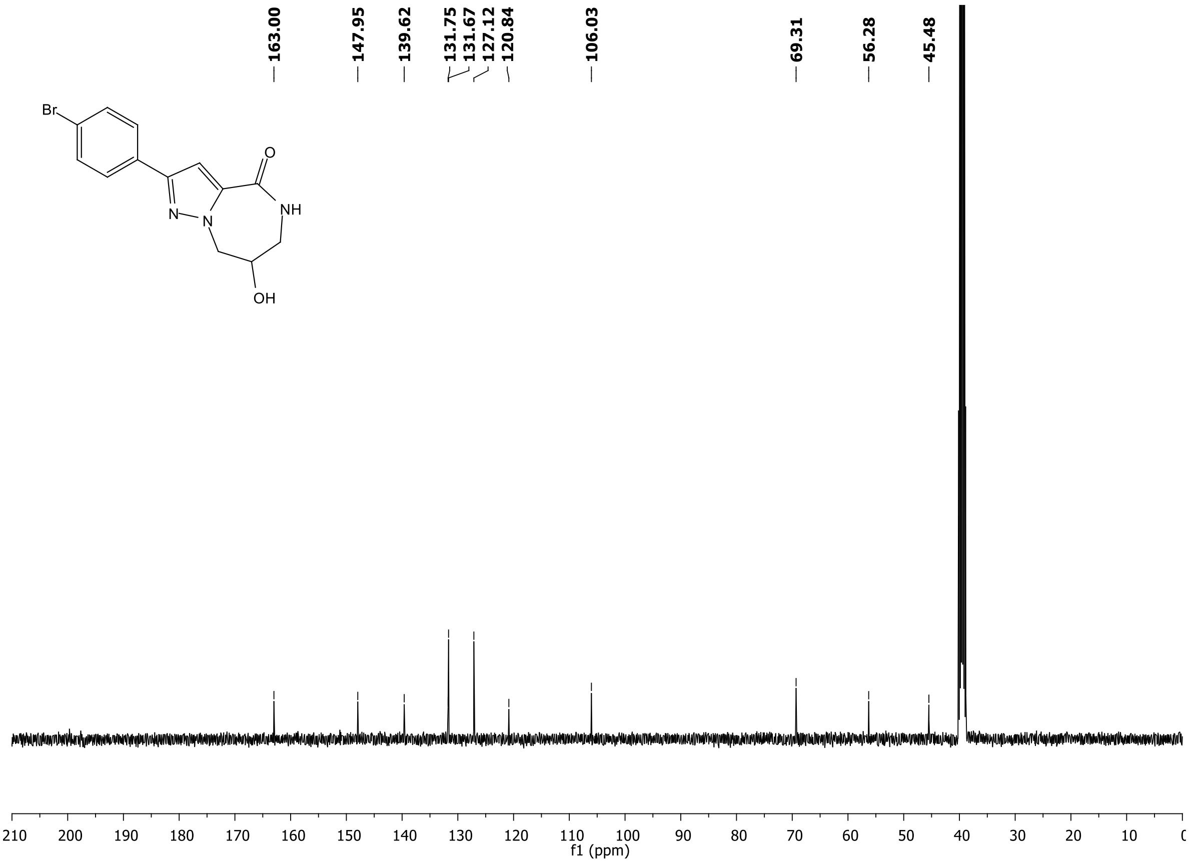

Figure S64. ^{13}C NMR spectrum (101 MHz, DMSO-d_6) of 2-(4-bromophenyl)-7-hydroxy-5,6,7,8-tetrahydro-4*H*-pyrazolo [1,5-*a*][1,4]diazepin-4-one (**4d**).

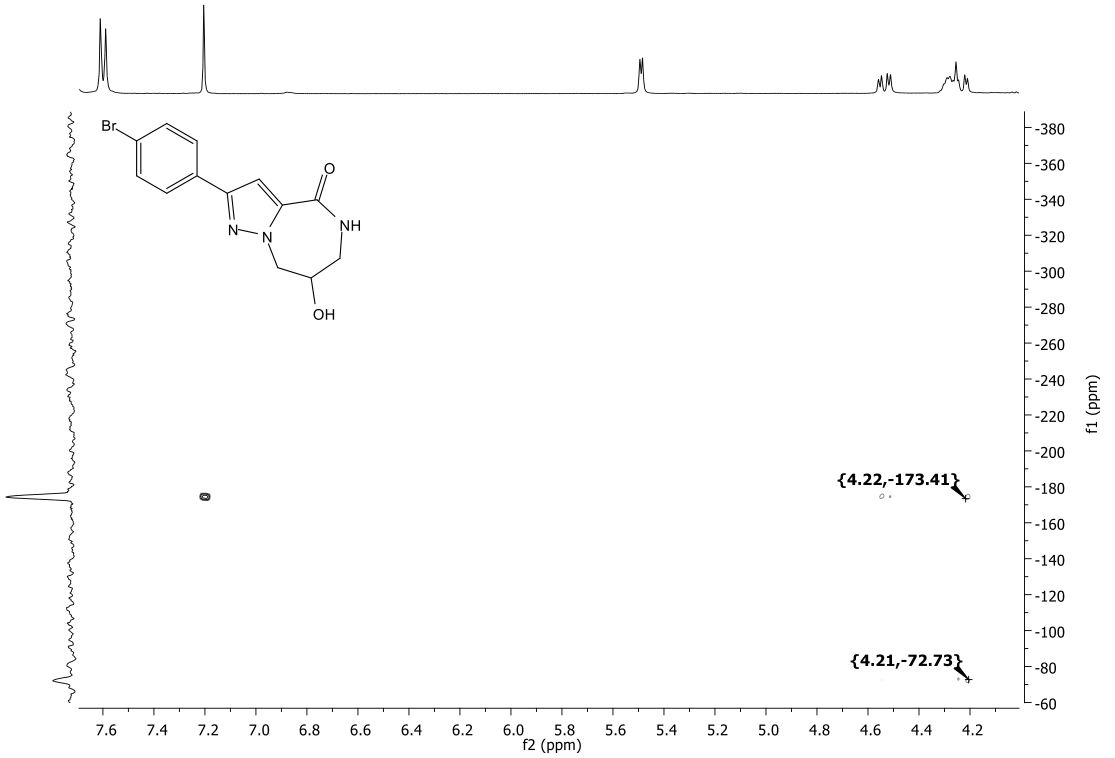

Figure S65. $^{1}H,^{15}N$-HMBC (40 MHz, DMSO-d_6) of 2-(4-bromophenyl)-7-hydroxy-5,6,7,8-tetrahydro-4*H*-pyrazolo [1,5-*a*][1,4]diazepin-4-one (**4d**).

Compound Spectrum SmartFormula Report

Analysis Info

Analysis Name	D:\Data\KDD-111.d	Acquisition Date	4/6/2020 9:53:30 AM
Method	DirectInfusion_TuneLow_pos.m	Operator	hplc
Sample Name	KDD-111	Instrument	micrOTOF-Q III 8228888.20448
Comment	SB		

Acquisition Parameter

Source Type	ESI	Ion Polarity	Positive	Set Nebulizer	0.4 Bar
Focus	Not active	Set Capillary	4500 V	Set Dry Heater	180 °C
Scan Begin	50 m/z	Set End Plate Offset	-500 V	Set Dry Gas	4.0 l/min
Scan End	1000 m/z	Set Collision Cell RF	140.0 Vpp	Set Divert Valve	Waste

#	RT [min]	Area	Int. Type	I	S/N	Chromatogram	Max. m/z	FWHM [min]
n.a.	0.1	n.a.	Single spectrum	n.a.	n.a.	n.a.	226.9518	n.a.
n.a.	5.4	n.a.	Single spectrum	n.a.	n.a.	n.a.	344.0005	n.a.

+MS, 5.4min #326

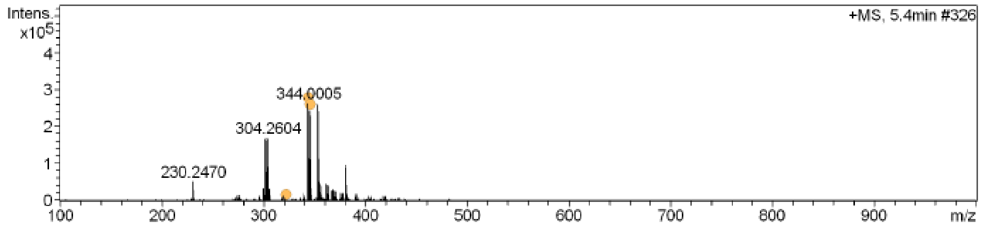

Meas. m/z	#	Ion Formula	m/z	err [ppm]	mSigma	# Sigma	Score	rdb	e⁻ Conf	N-Rule
322.0210	1	C13H13BrN3O2	322.0186	7.6	156.5	1	100.00	8.5	even	ok
344.0005	1	C13H12BrN3NaO2	344.0005	0.1	25.3	1	100.00	8.5	even	ok
345.9983	1	C13H12BrN3NaO2	344.0005	-0.8	25.3	1	100.00	8.5	even	ok

Figure S66. HRMS (ESI-TOF) spectrum of 2-(4-bromophenyl)-7-hydroxy-5,6,7,8-tetrahydro-4*H*-pyrazolo [1,5-*a*][1,4]diazepin-4-one (**4d**).

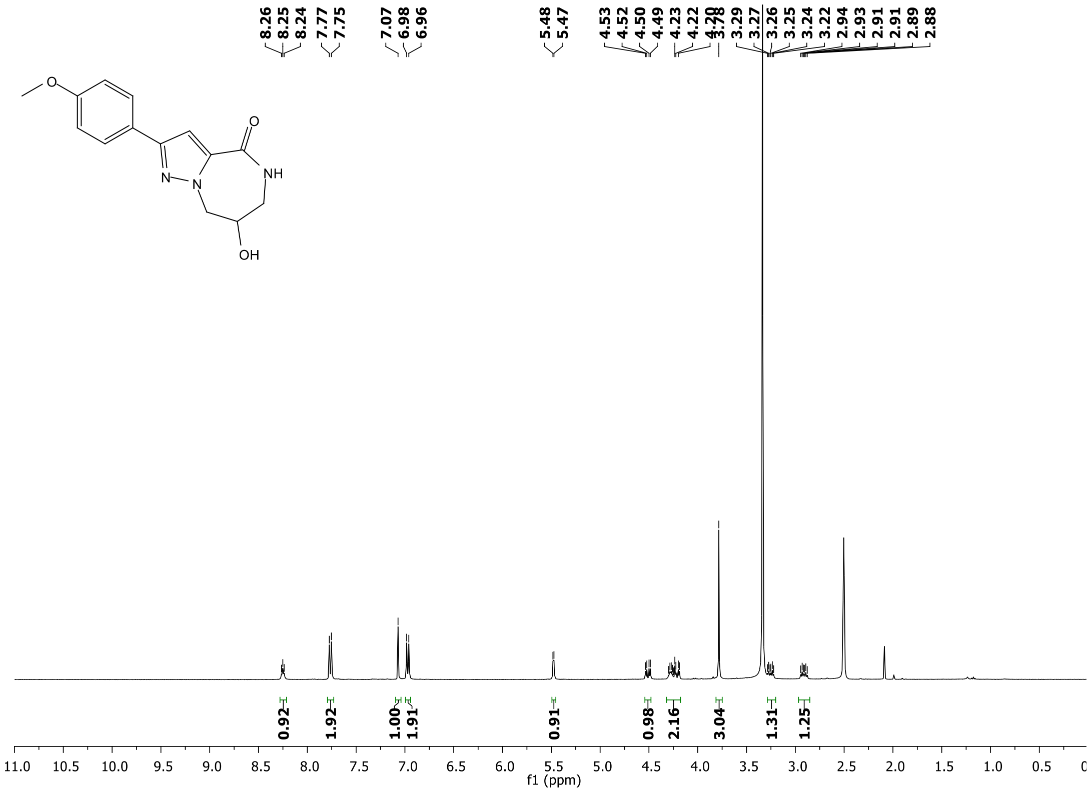

Figure S67. ^{1}H NMR spectrum (400 MHz, DMSO-d_6) of 7-hydroxy-2-(4-methoxyphenyl)-5,6,7,8-tetrahydro-4*H*-pyrazolo
[1,5-*a*][1,4]diazepin-4-one (**4e**).

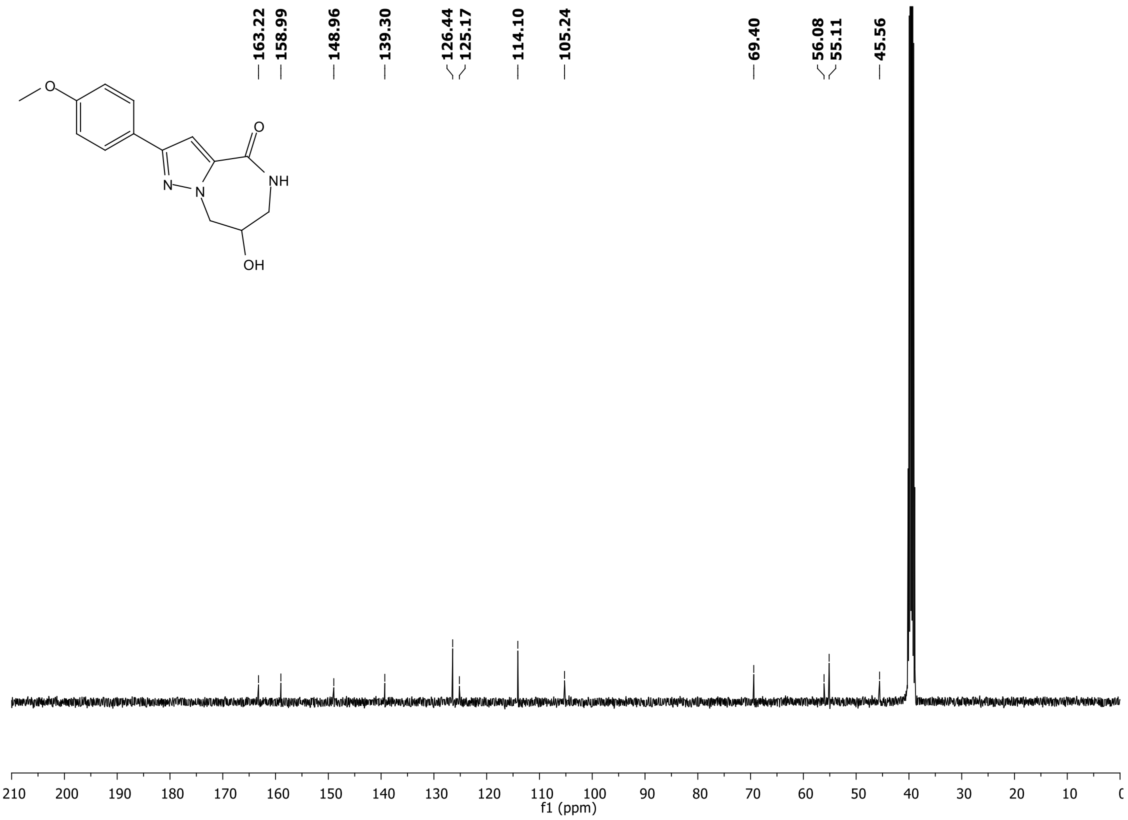

Figure S68. ^{13}C NMR spectrum (101 MHz, DMSO-d_6) of 7-hydroxy-2-(4-methoxyphenyl)-5,6,7,8-tetrahydro-4*H*-pyrazolo
[1,5-*a*][1,4]diazepin-4-one (**4e**).

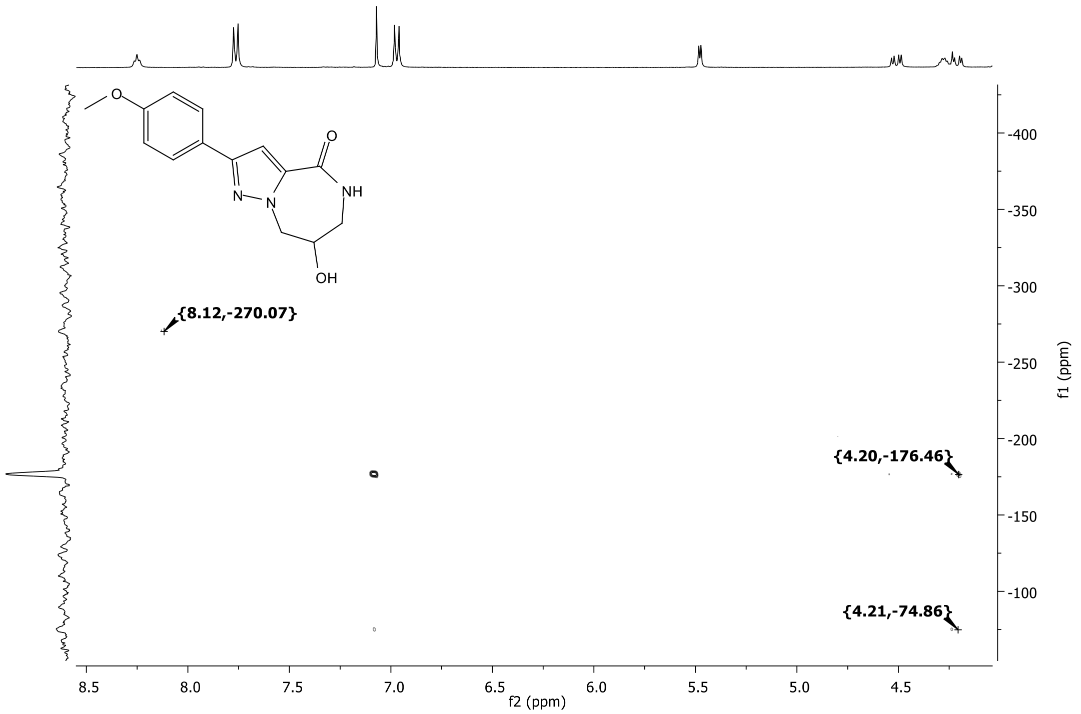

Figure S69. ^{1}H,^{15}N-HMBC (40 MHz, DMSO-d_6) of 7-hydroxy-2-(4-methoxyphenyl)-5,6,7,8-tetrahydro-4*H*-pyrazolo
[1,5-*a*][1,4]diazepin-4-one (**4e**).

Compound Spectrum SmartFormula Report

Analysis Info

Analysis Name	D:\Data\KDD-173-1.d	Acquisition Date	8/10/2021 10:17:42 AM
Method	DirectInfusion_TuneLow_pos.m	Operator	hplc
Sample Name	KDD-173-1	Instrument	micrOTOF-Q III 8228888.20448
Comment	SB		

Acquisition Parameter

Source Type	ESI	Ion Polarity	Positive	Set Nebulizer	0.4 Bar
Focus	Not active	Set Capillary	4500 V	Set Dry Heater	180 °C
Scan Begin	50 m/z	Set End Plate Offset	-500 V	Set Dry Gas	4.0 l/min
Scan End	1000 m/z	Set Collision Cell RF	140.0 Vpp	Set Divert Valve	Waste

#	RT [min]	Area	Int. Type	I	S/N	Chromatogram	Max. m/z	FWHM [min]
n.a.	0.2	n.a.	Single spectrum	n.a.	n.a.	n.a.	226.9517	n.a.
n.a.	4.5	n.a.	Single spectrum	n.a.	n.a.	n.a.	304.2606	n.a.

+MS, 4.5min #272

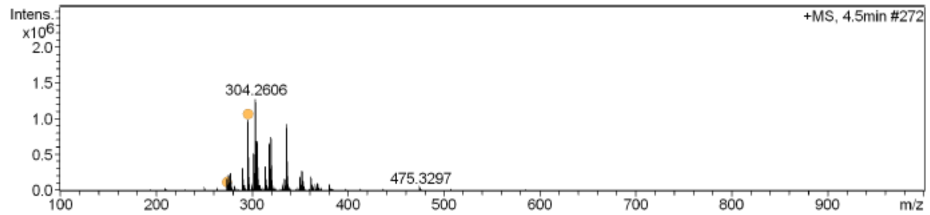

Meas. m/z	#	Ion Formula	m/z	err [ppm]	mSigma	# Sigma	Score	rdb	e⁻ Conf	N-Rule
274.1180	1	C14H16N3O3	274.1186	-2.3	31.8	1	100.00	8.5	even	ok
296.1006	1	C14H15N3NaO3	296.1006	-0.1	20.0	1	100.00	8.5	even	ok

Figure S70. HRMS (ESI-TOF) spectrum of 7-hydroxy-2-(4-methoxyphenyl)-5,6,7,8-tetrahydro-4*H*-pyrazolo [1,5-*a*][1,4]diazepin-4-one (**4e**).

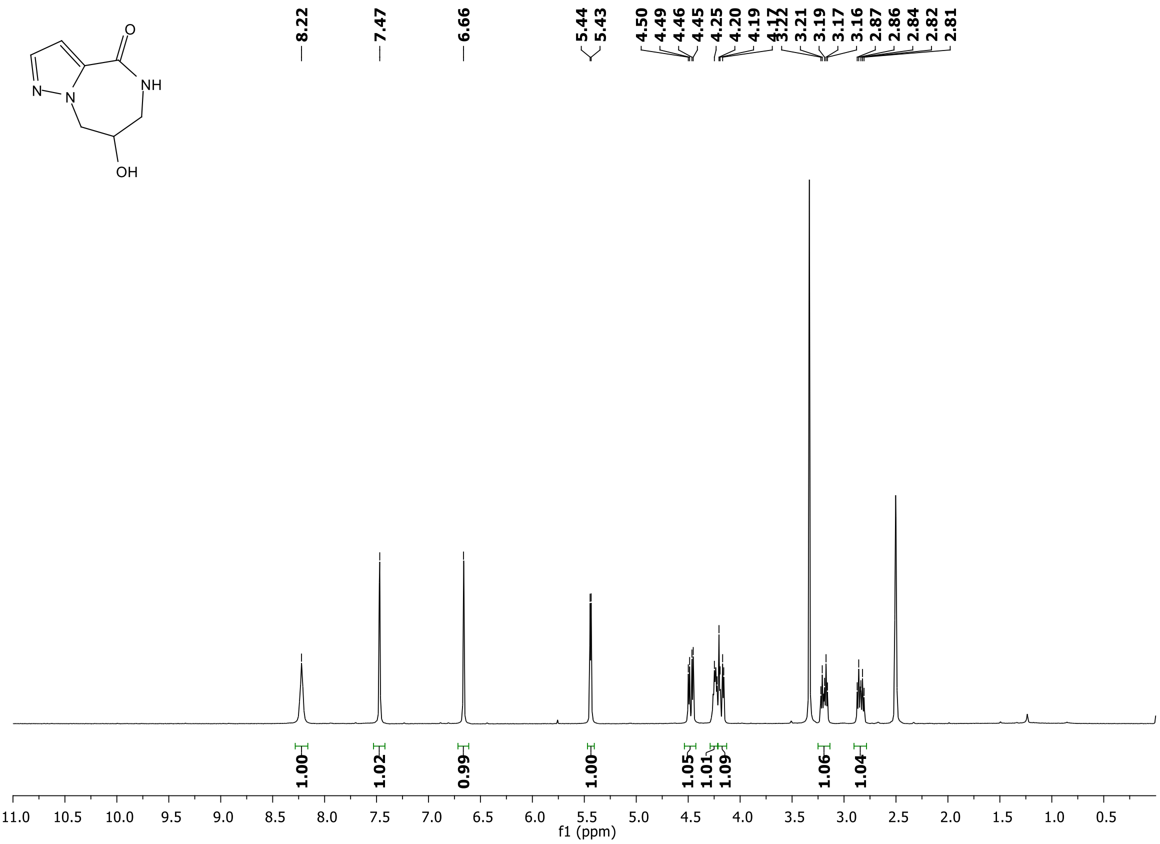

Figure S71. ^{1}H NMR spectrum (400 MHz, DMSO-d_6) of 7-hydroxy-5,6,7,8-tetrahydro-4*H*-pyrazolo[1,5-*a*][1,4]diazepin-4-one (**4f**).

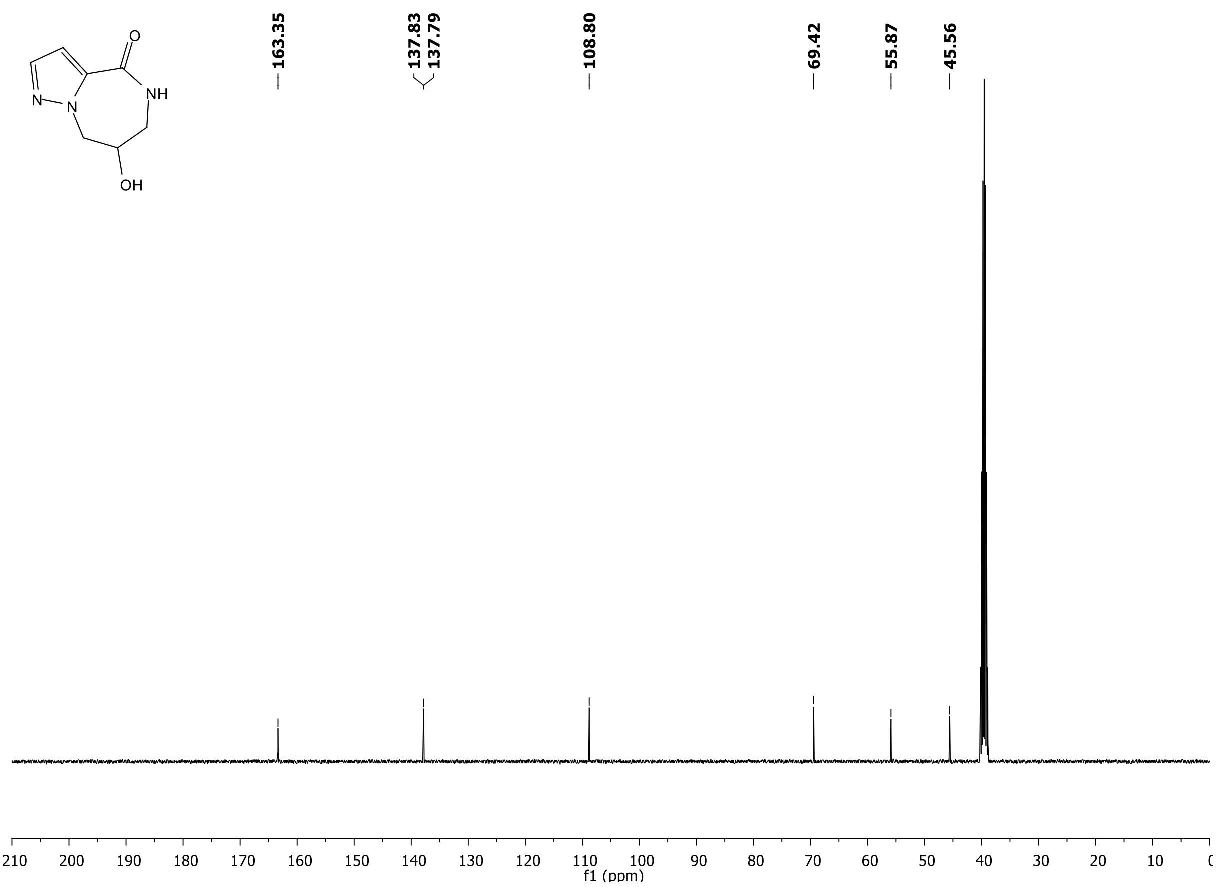

Figure S72. ^{13}C NMR spectrum (101 MHz, DMSO-d_6) of 7-hydroxy-5,6,7,8-tetrahydro-4*H*-pyrazolo[1,5-*a*][1,4]diazepin-4-one (**4f**).

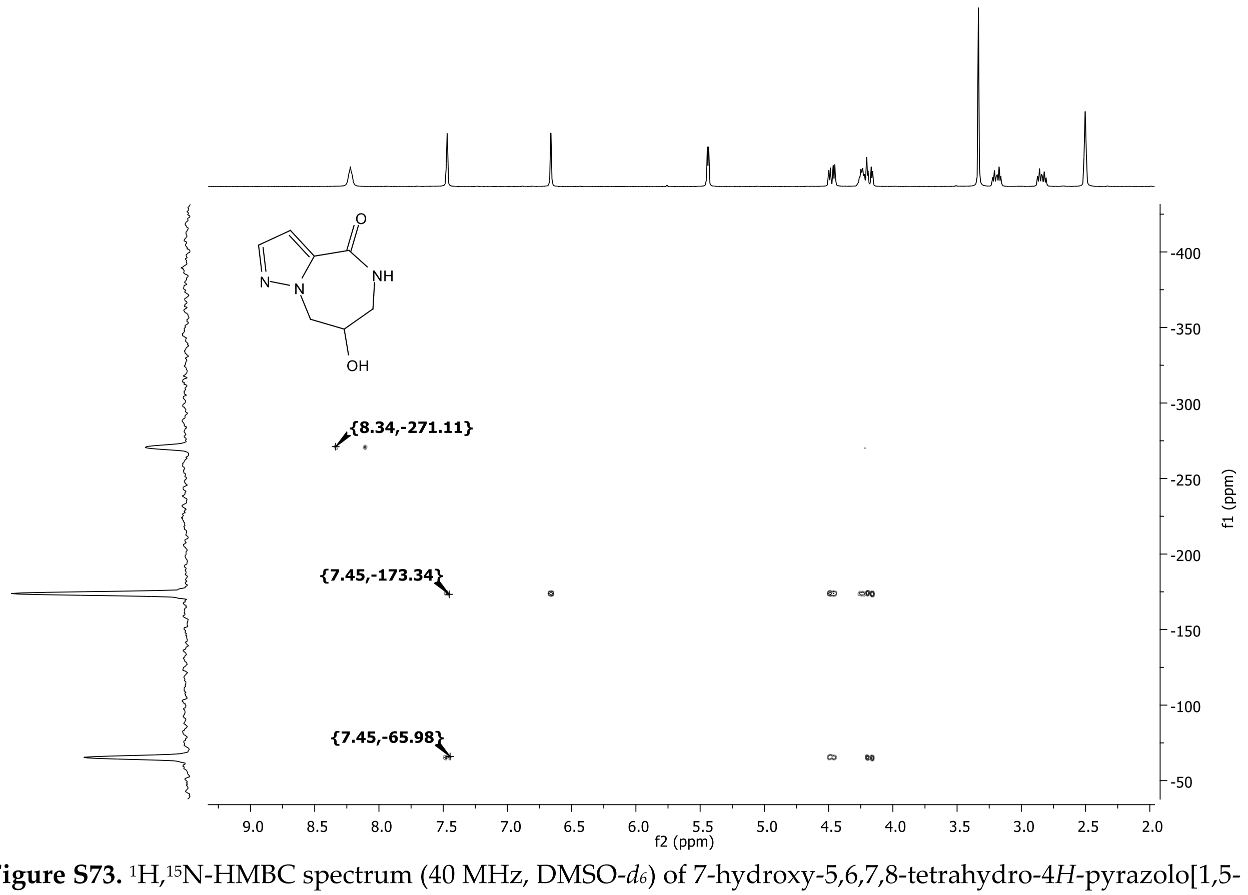

Figure S73. $^{1}H,^{15}N$-HMBC spectrum (40 MHz, DMSO-d_6) of 7-hydroxy-5,6,7,8-tetrahydro-4*H*-pyrazolo[1,5-*a*][1,4]diazepin-4-one (**4f**).

Qualitative Compound Report

**Data File**	221114_MV-46-2_02.d	**Sample Name**	MV-46-2
**Sample Type**	Sample	**Position**	
**Instrument Name**	G6230B TOF	**User Name**	
**Acq Method**	HRMS_12min_ref.m	**Acquired Time**	14-Nov-22 8:44:54 AM
**IRM Calibration Status**	Success	**DA Method**	test.m
**Comment**			
**Sample Group**		**Info.**	
**Stream Name**		**Acquisition SW Version**	6200 series TOF/6500 series Q-TOF B.09.00 (B9044.1 SP1)

Compound Table

Compound Label	RT	Mass	Abund	Formula	Tgt Mass	Diff (ppm)
Cpd 1: C7 H9 N3 O2	6.677	167.0689	4012768	C7 H9 N3 O2	167.0695	-3.46

Compound Label	m/z	RT	Algorithm	Mass
Cpd 1: C7 H9 N3 O2	168.0763	6.677	Find By Formula	167.0689

Compound Chromatograms

MS Spectrum

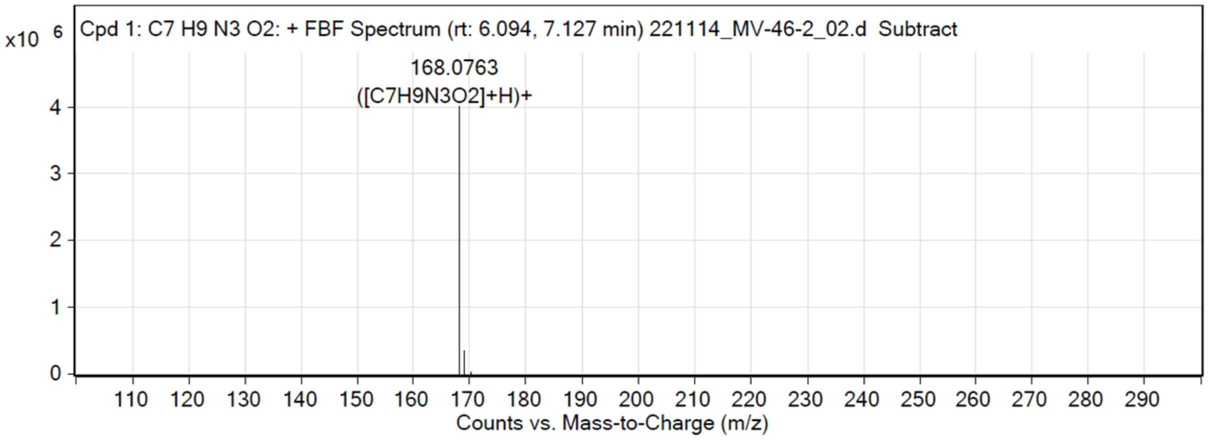

Figure S74. HRMS (ESI-TOF) spectrum of 7-hydroxy-5,6,7,8-tetrahydro-4*H*-pyrazolo[1,5-*a*][1,4]diazepin-4-one (**4f**).

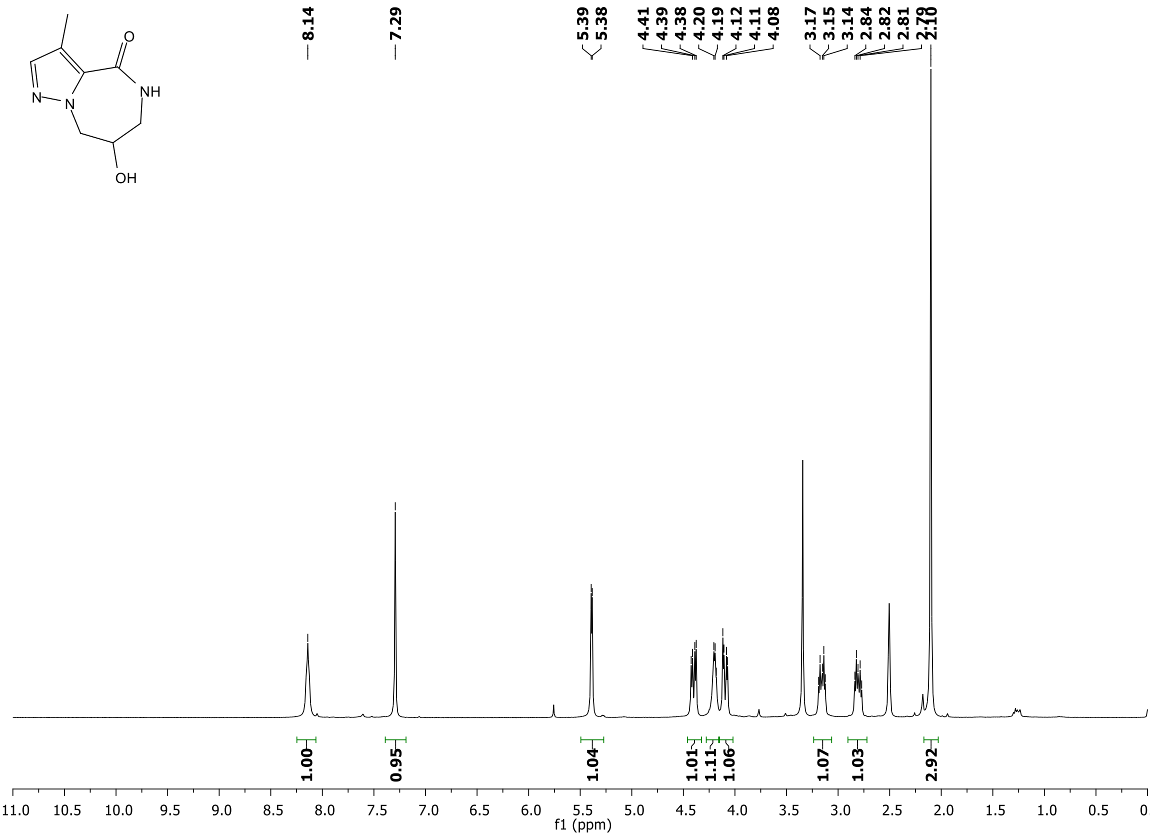

Figure S75. ¹H NMR spectrum (400 MHz, DMSO-*d*₆) of 7-hydroxy-3-methyl-5,6,7,8-tetrahydro-4*H*-pyrazolo[1,5-*a*][1,4]diazepin-4-one (**4g**).

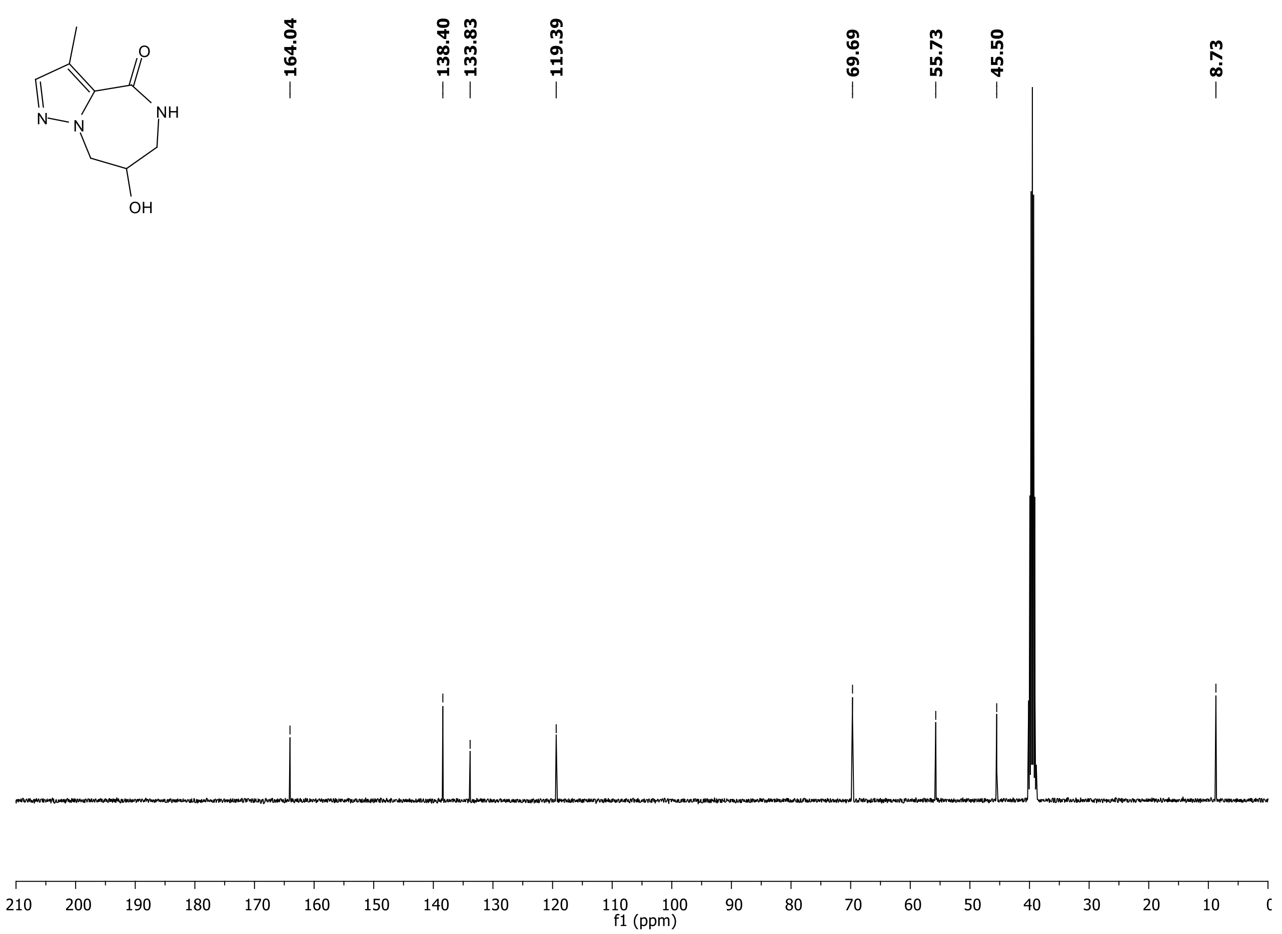

Figure S76. ^{13}C NMR spectrum (101 MHz, DMSO-d_6) of 7-hydroxy-3-methyl-5,6,7,8-tetrahydro-4*H*-pyrazolo[1,5-*a*][1,4]diazepin-4-one (**4g**).

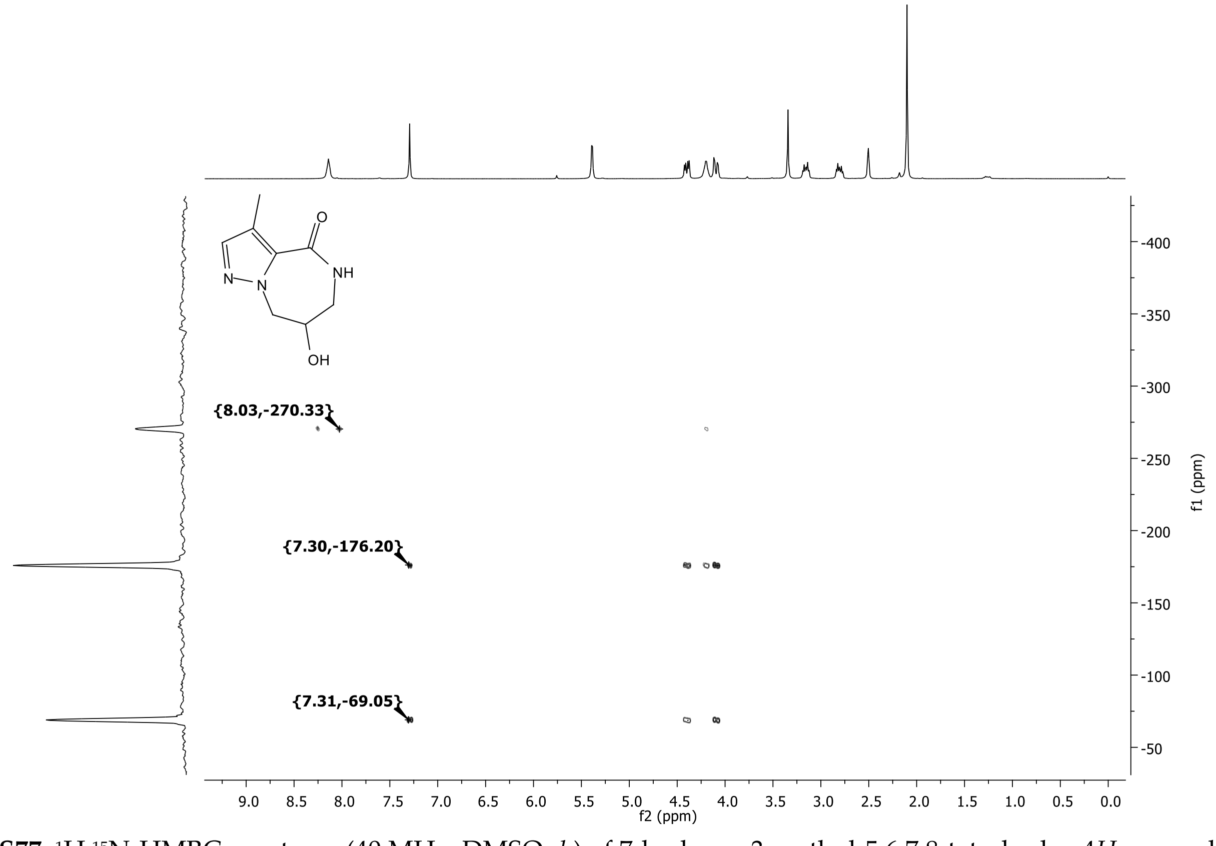

Figure S77. $^{1}H,^{15}N$-HMBC spectrum (40 MHz, DMSO-d_6) of 7-hydroxy-3-methyl-5,6,7,8-tetrahydro-4*H*-pyrazolo[1,5-*a*][1,4]diazepin-4-one (**4g**).

Qualitative Compound Report

**Data File**	221111_MV-49-1_01.d	**Sample Name**	MV-49-1
**Sample Type**	Sample	**Position**	
**Instrument Name**	G6230B TOF	**User Name**	
**Acq Method**	HRMS_12min_ref.m	**Acquired Time**	11-Nov-22 1:15:23 PM
**IRM Calibration Status**	Success	**DA Method**	test.m
**Comment**			
**Sample Group**		**Info.**	
**Stream Name**		**Acquisition SW Version**	6200 series TOF/6500 series Q-TOF B.09.00 (B9044.1 SP1)

Compound Table

Compound Label	RT	Mass	Abund	Formula	Tgt Mass	Diff (ppm)
Cpd 1: C8 H11 N3 O2	6.546	181.0852	4149684	C8 H11 N3 O2	181.0851	0.46

Compound Label	*m/z*	RT	Algorithm	Mass
Cpd 1: C8 H11 N3 O2	182.0923	6.546	Find By Formula	181.0852

Compound Chromatograms

MS Spectrum

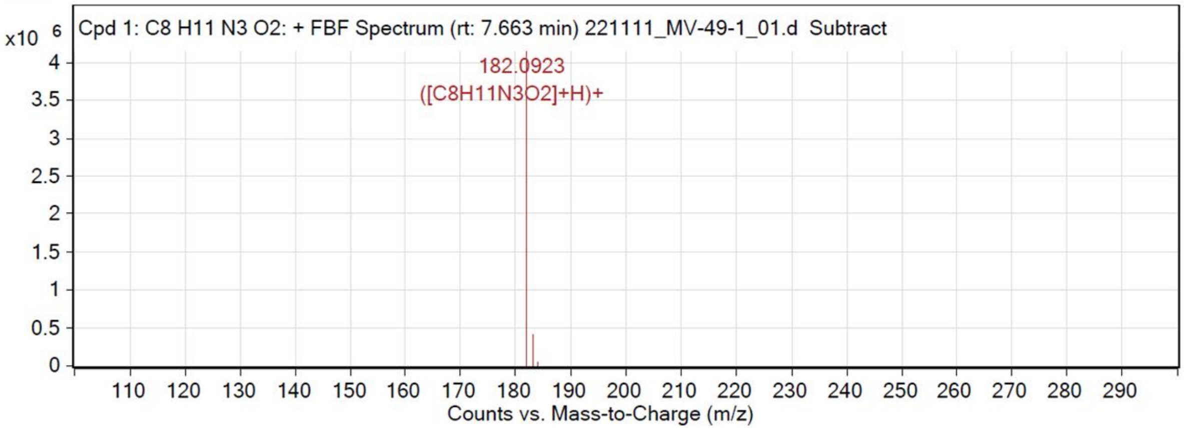

Figure S78. HRMS (ESI-TOF) spectrum of 7-hydroxy-3-methyl-5,6,7,8-tetrahydro-4*H*-pyrazolo[1,5-*a*][1,4]diazepin-4-one (**4g**).

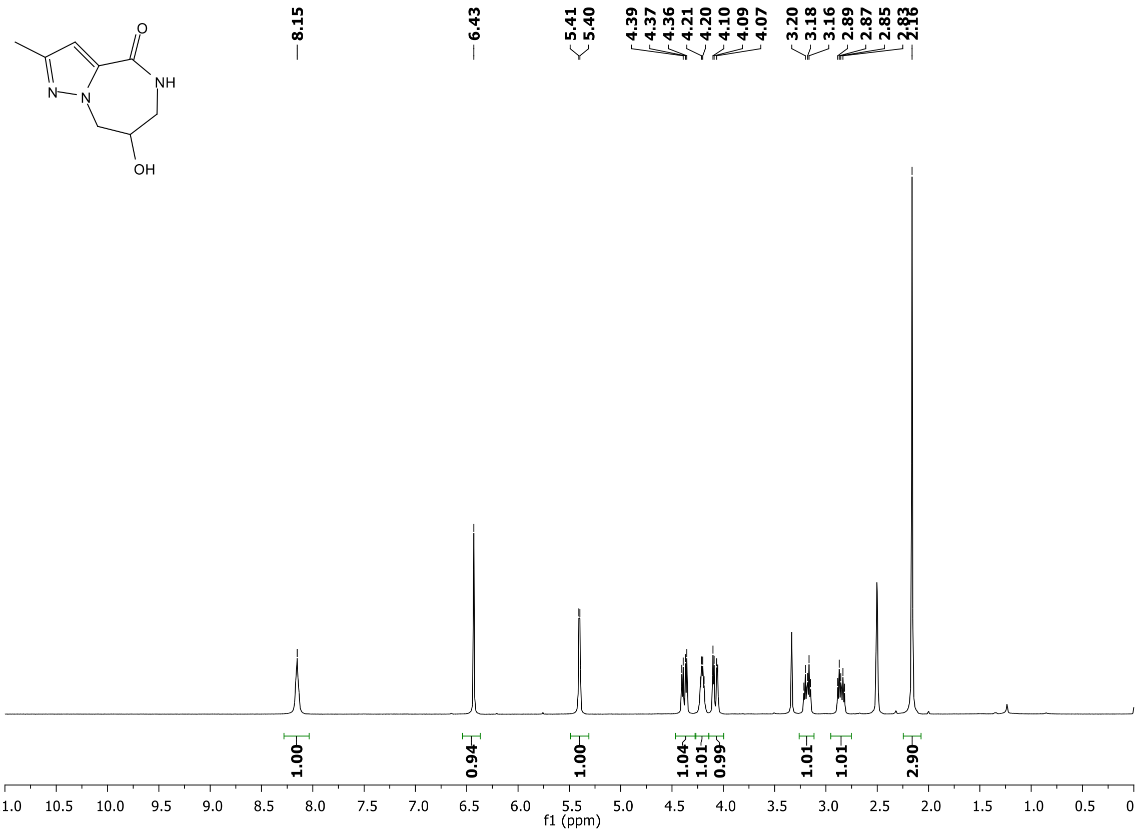

Figure S79. ^{1}H NMR spectrum (400 MHz, DMSO-*d*$_6$) of 7-hydroxy-2-methyl-5,6,7,8-tetrahydro-4*H*-pyrazolo[1,5-*a*][1,4]diazepin-4-one (**4h**).

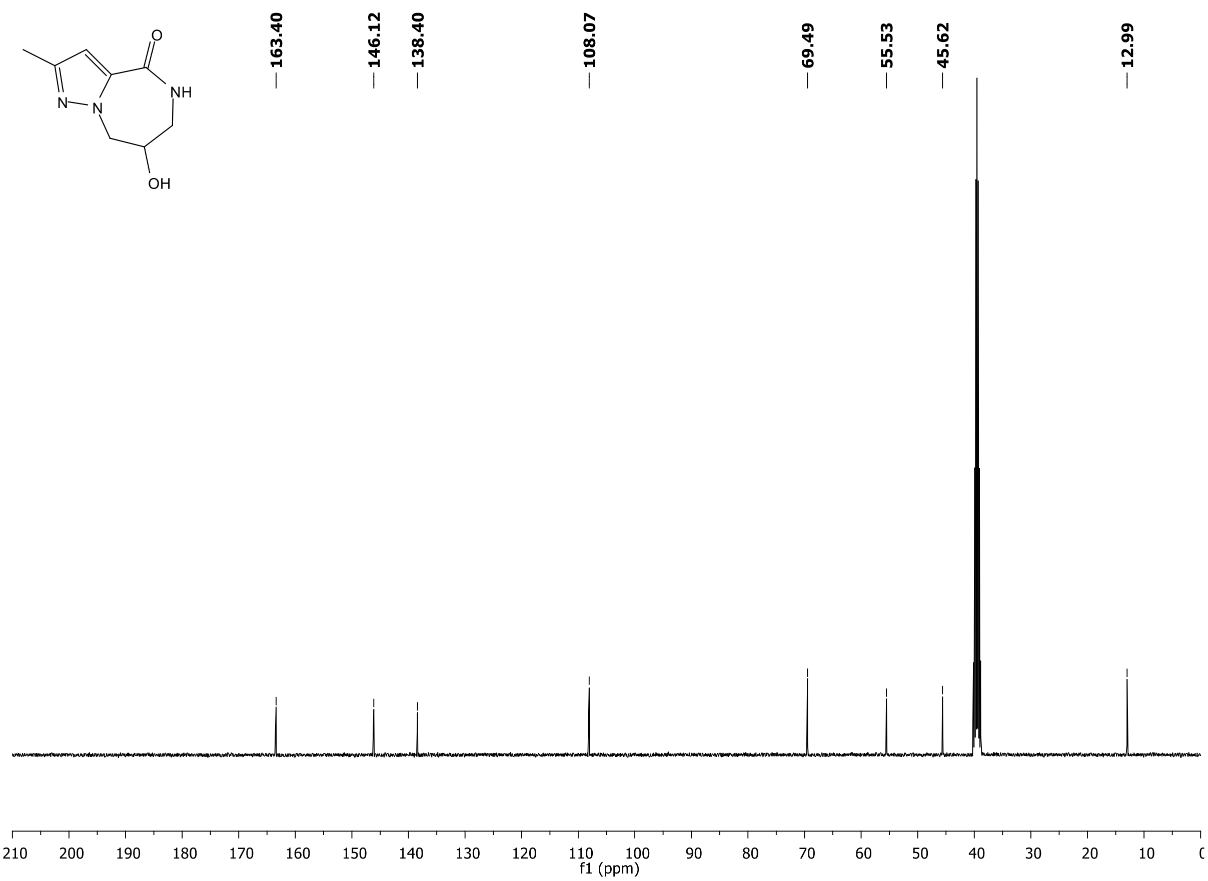

Figure S80. ^{13}C NMR spectrum (101 MHz, DMSO-d_6) of 7-hydroxy-2-methyl-5,6,7,8-tetrahydro-4*H*-pyrazolo[1,5-*a*][1,4]diazepin-4-one (**4h**).

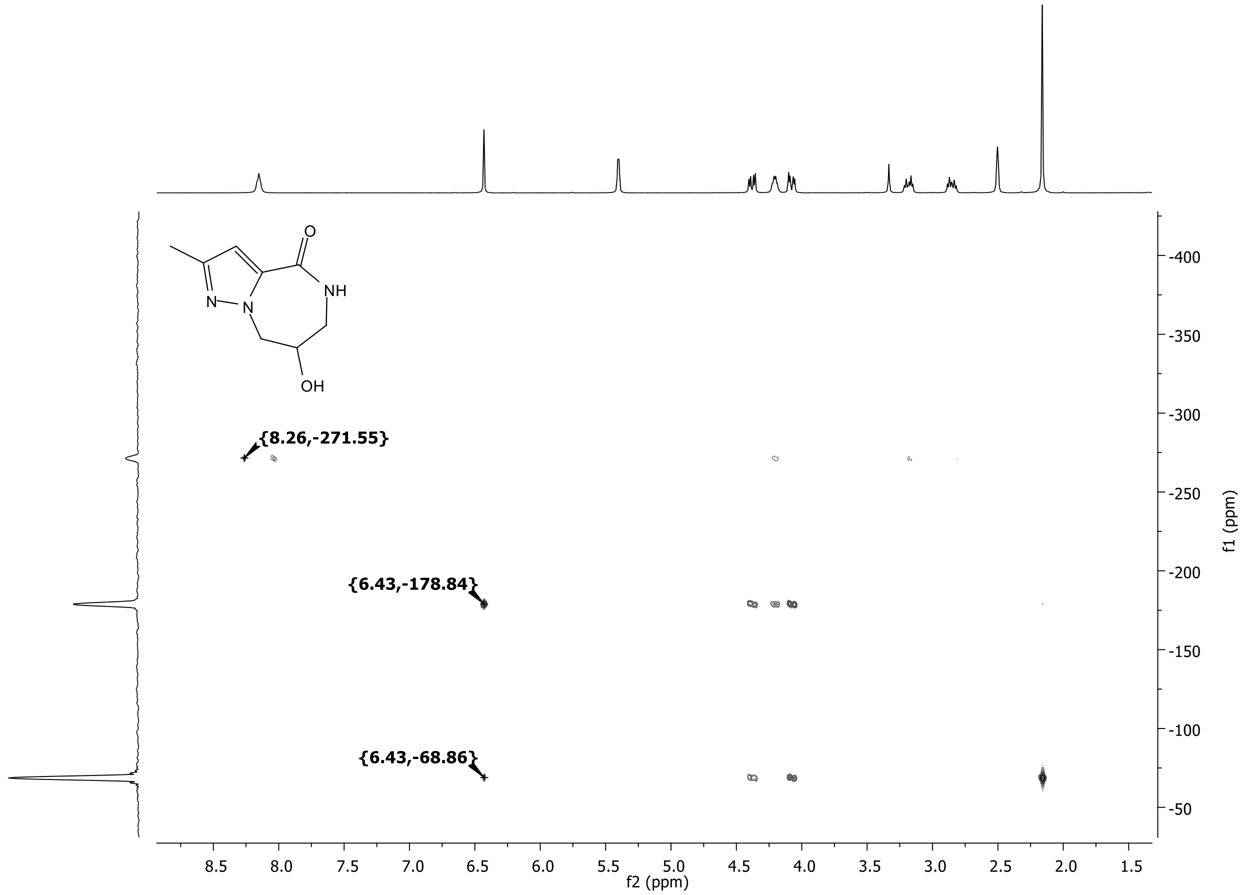

Figure S81. ^{1}H,^{15}N-HMBC spectrum (40 MHz, DMSO-d_6) of 7-hydroxy-2-methyl-5,6,7,8-tetrahydro-4*H*-pyrazolo[1,5-*a*][1,4]diazepin-4-one (**4h**).

Qualitative Compound Report

**Data File**	221111_MV-56_01.d	**Sample Name**	MV-56
**Sample Type**	Sample	**Position**	
**Instrument Name**	G6230B TOF	**User Name**	
**Acq Method**	HRMS_12min_ref.m	**Acquired Time**	11-Nov-22 12:06:00 PM
**IRM Calibration Status**	Success	**DA Method**	test.m
**Comment**			
**Sample Group**		**Info.**	
**Stream Name**		**Acquisition SW Version**	6200 series TOF/6500 series Q-TOF B.09.00 (B9044.1 SP1)

Compound Table

Compound Label	RT	Mass	Abund	Formula	Tgt Mass	Diff (ppm)
Cpd 1: C8 H11 N3 O2	6.691	181.0853	2178601	C8 H11 N3 O2	181.0851	0.71

Compound Label	*m/z*	RT	Algorithm	Mass
Cpd 1: C8 H11 N3 O2	182.0923	6.691	Find By Formula	181.0853

Compound Chromatograms

MS Spectrum

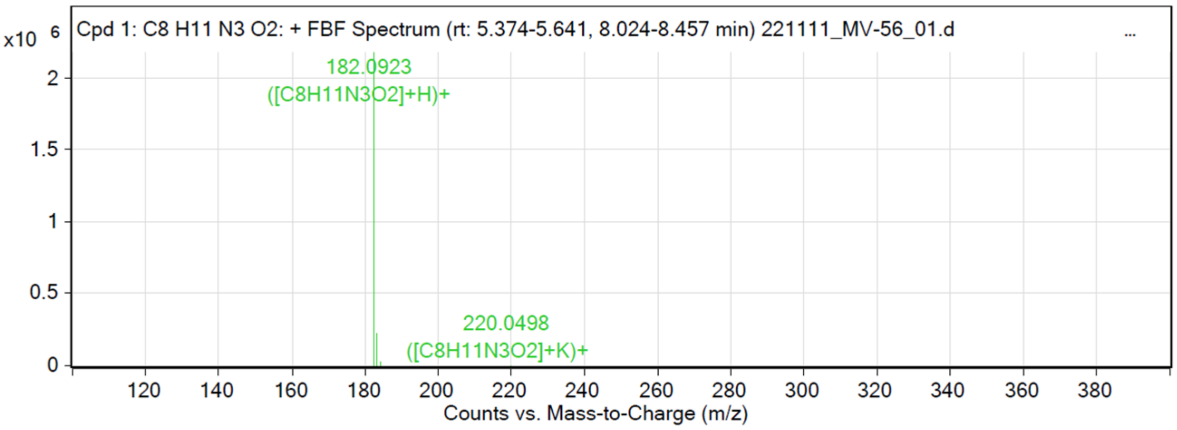

Figure S82. HRMS (ESI-TOF) spectrum of 7-hydroxy-2-methyl-5,6,7,8-tetrahydro-4*H*-pyrazolo[1,5-*a*][1,4]diazepin-4-one (**4h**).

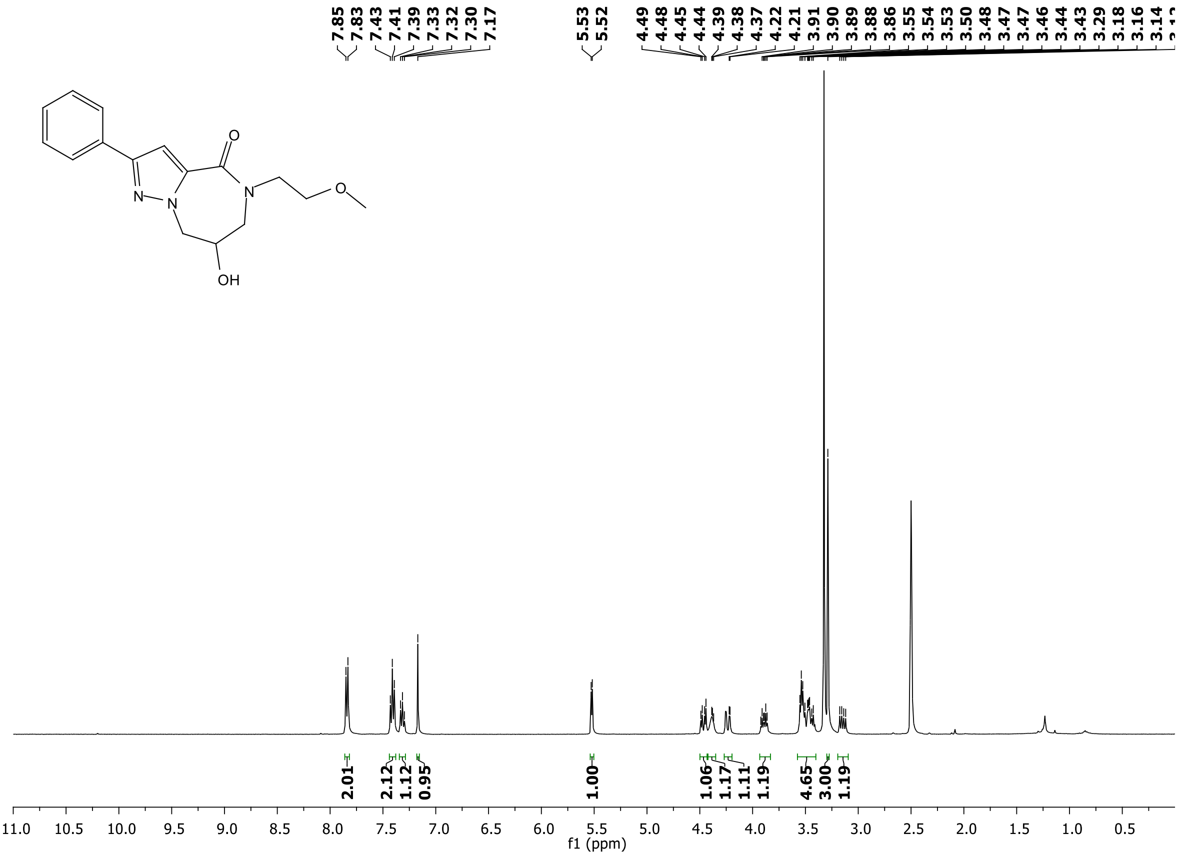

Figure S83. ^{1}H NMR spectrum (400 MHz, DMSO-d_6) of 7-hydroxy-5-(2-methoxyethyl)-2-phenyl-5,6,7,8-tetrahydro-4*H*-pyrazolo[1,5-*a*][1,4]diazepin-4-one (**4i**).

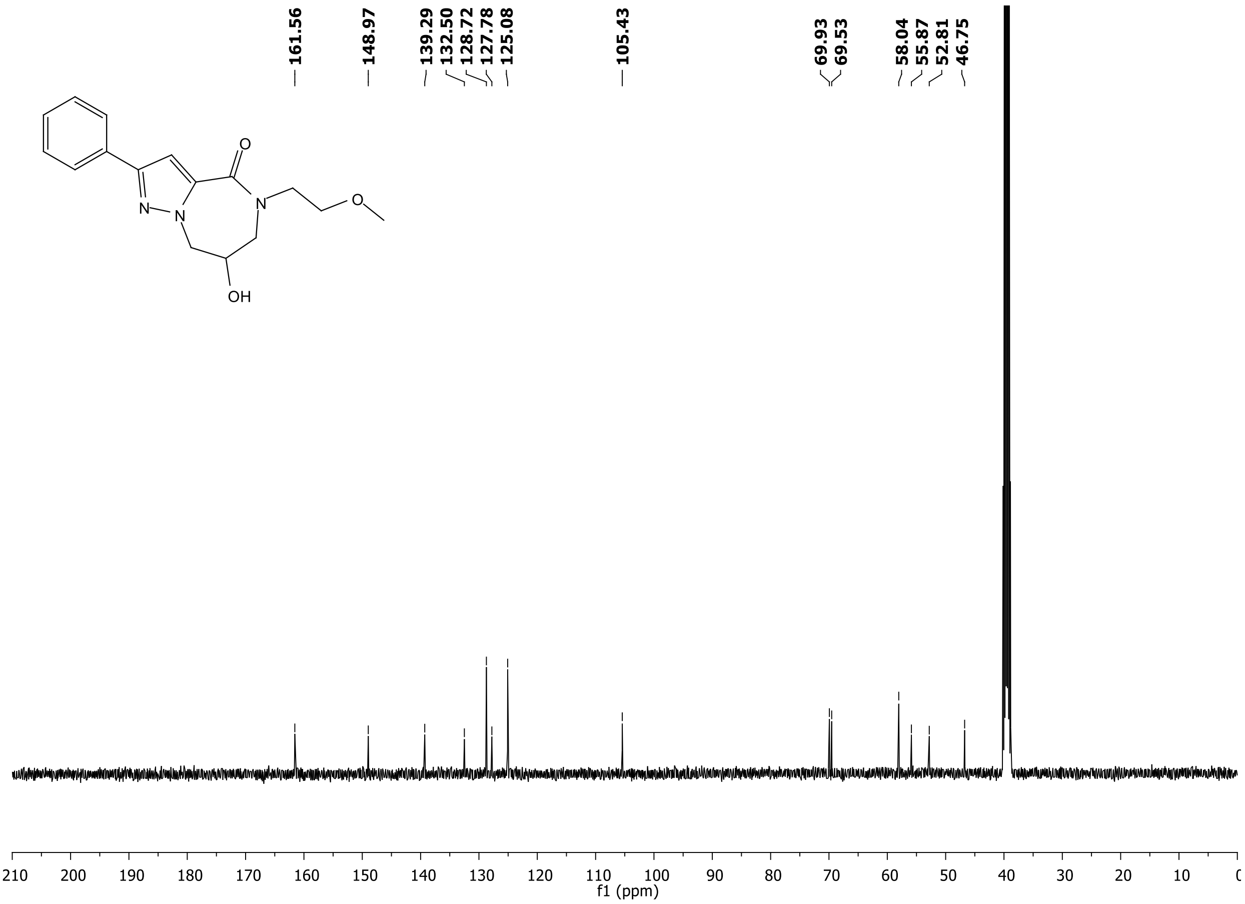

Figure S84. ^{13}C NMR spectrum (101 MHz, DMSO-d_6) of 7-hydroxy-5-(2-methoxyethyl)-2-phenyl-5,6,7,8-tetrahydro-4*H*-pyrazolo[1,5-*a*][1,4]diazepin-4-one (**4i**).

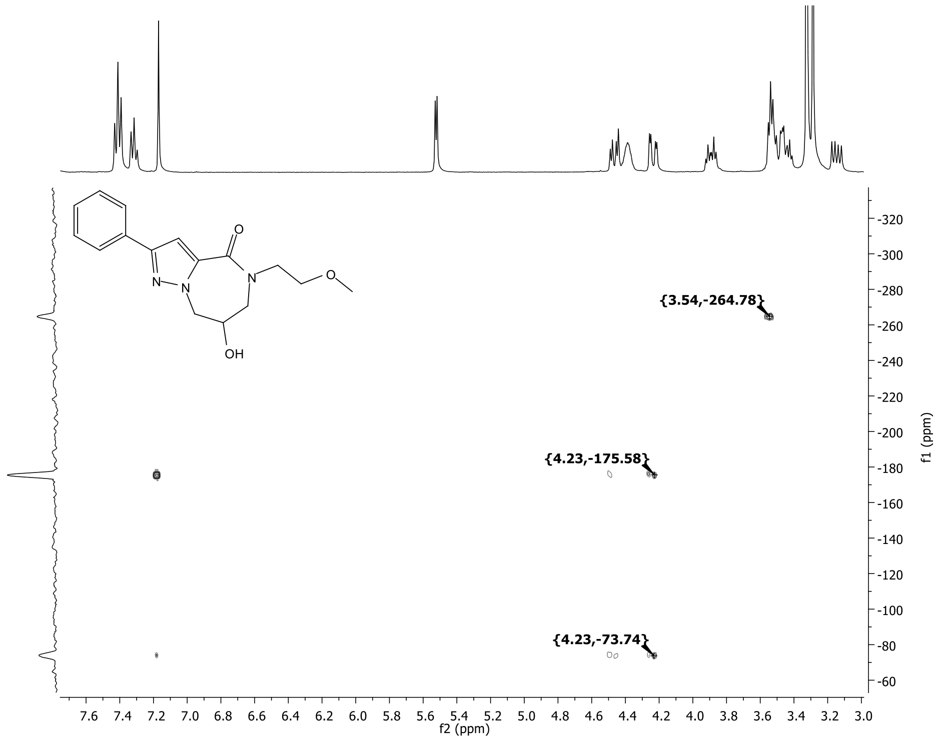

Figure S85. ^{1}H,^{15}N-HMBC (40 MHz, DMSO-d_6) of 7-hydroxy-5-(2-methoxyethyl)-2-phenyl-5,6,7,8-tetrahydro-4*H*-pyrazolo[1,5-*a*][1,4]diazepin-4-one (**4i**).

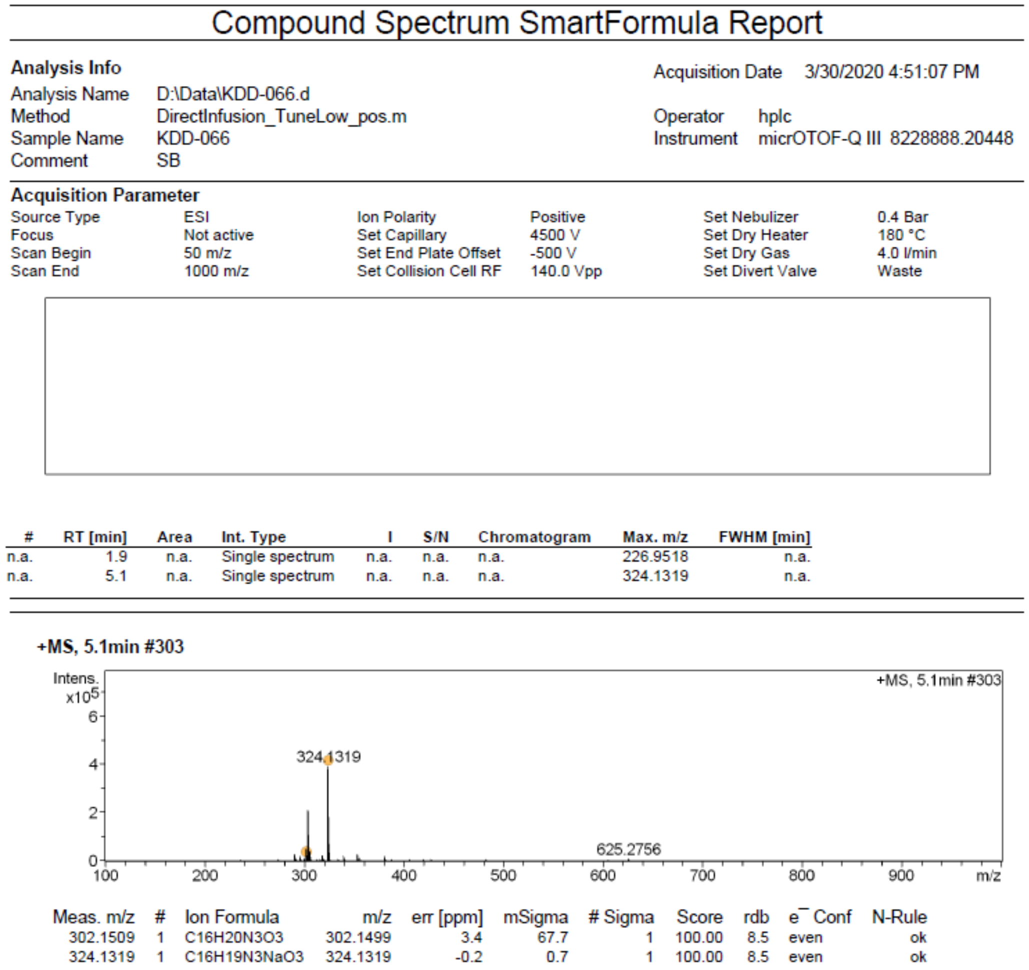

Figure S86. HRMS (ESI-TOF) spectrum of 7-hydroxy-5-(2-methoxyethyl)-2-phenyl-5,6,7,8-tetrahydro-4*H*-pyrazolo[1,5-*a*][1,4]diazepin-4-one (**4i**).

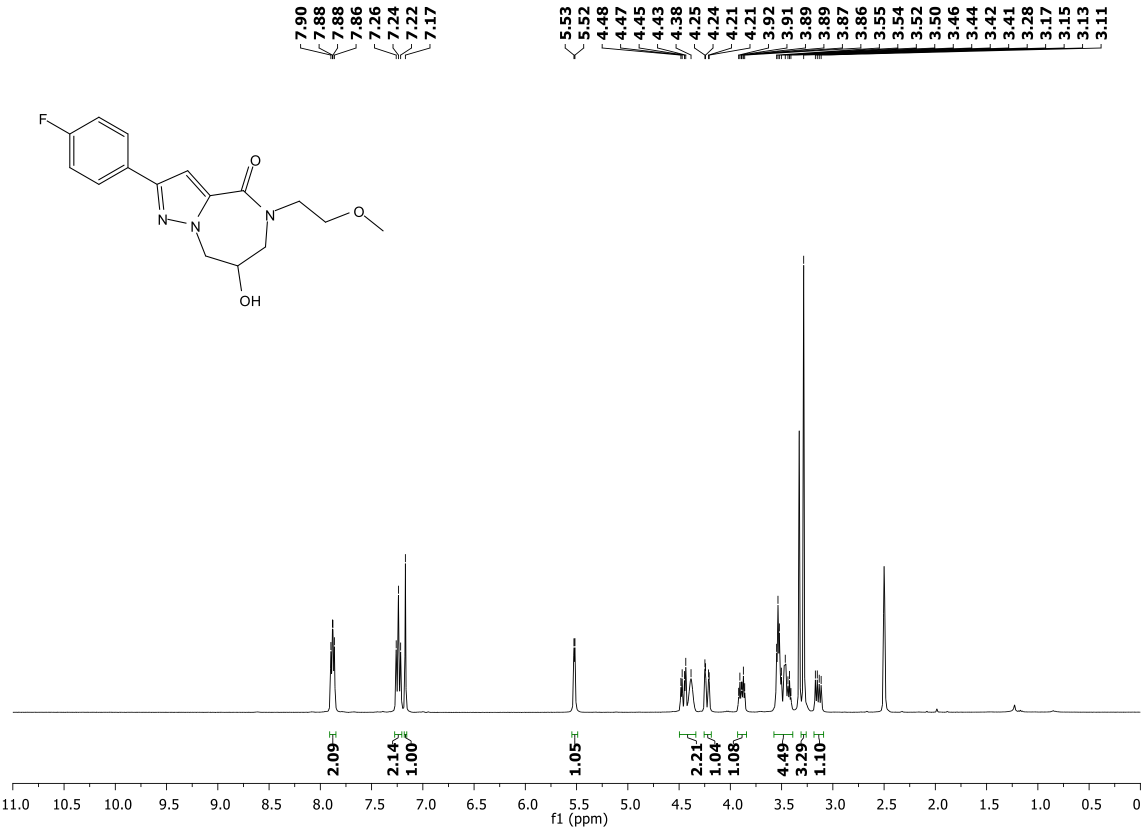

Figure S87. ¹H NMR spectrum (400 MHz, DMSO-*d*₆) of 2-(4-fluorophenyl)-7-hydroxy-5-(2-methoxyethyl)-5,6,7,8-tetrahydro-4*H*-pyrazolo[1,5-*a*][1,4]diazepin-4-one (**4j**).

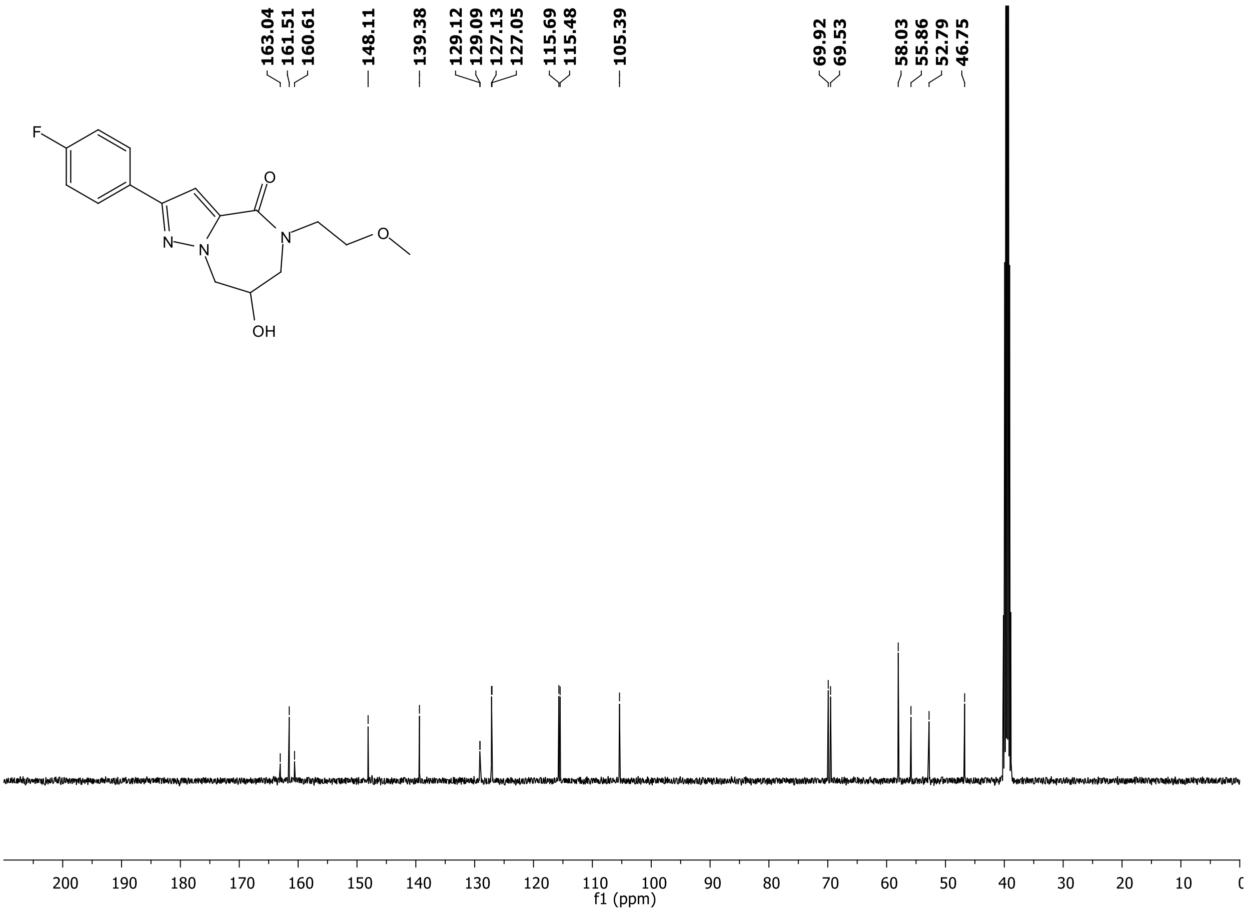

Figure S88. ^{13}C NMR spectrum (101 MHz, DMSO-d_6) of 2-(4-fluorophenyl)-7-hydroxy-5-(2-methoxyethyl)-5,6,7,8-tetrahydro-4*H*-pyrazolo[1,5-*a*][1,4]diazepin-4-one (**4j**).

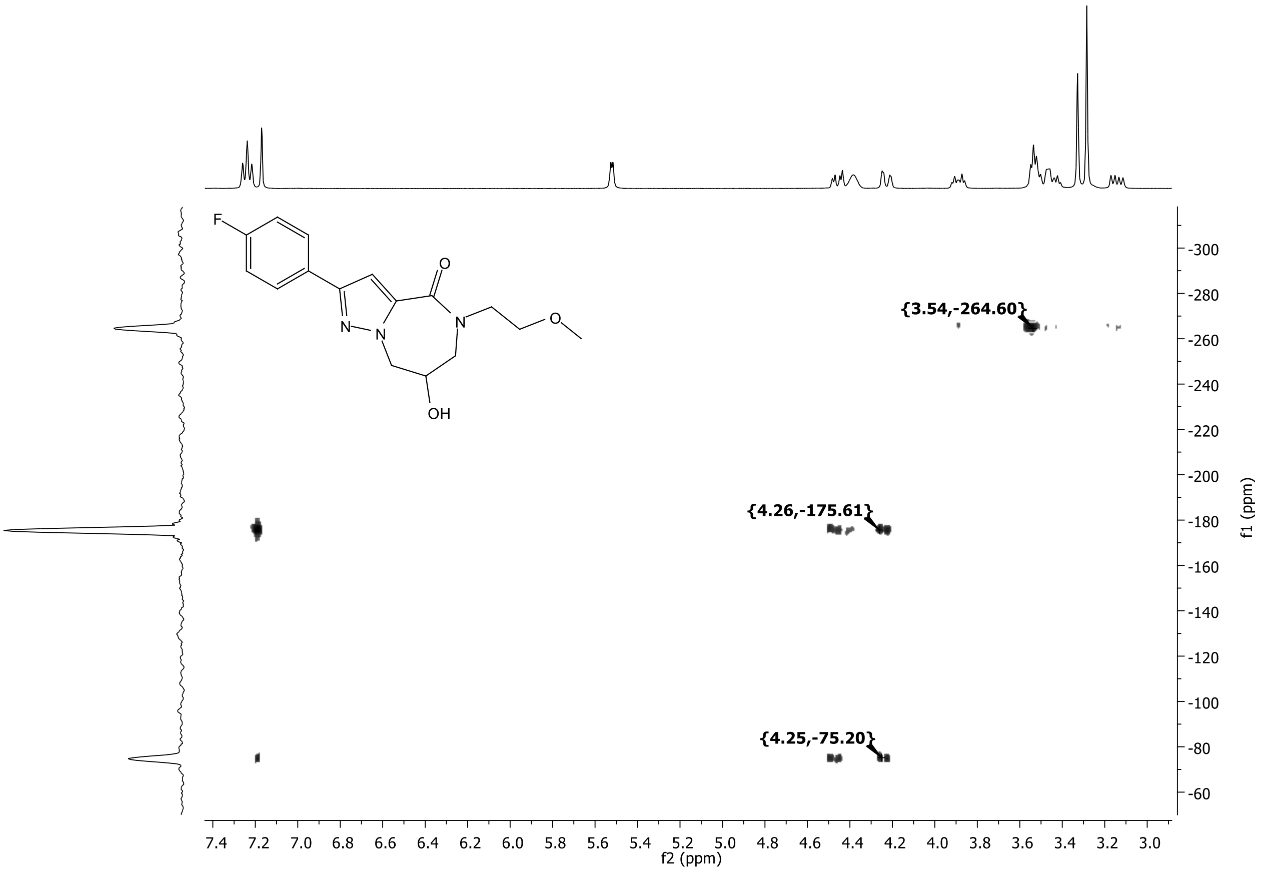

Figure S89. $^{1}H,^{15}N$-HMBC (40 MHz, DMSO-d_6) of 2-(4-fluorophenyl)-7-hydroxy-5-(2-methoxyethyl)-5,6,7,8-tetrahydro-4*H*-pyrazolo[1,5-*a*][1,4]diazepin-4-one (**4j**).

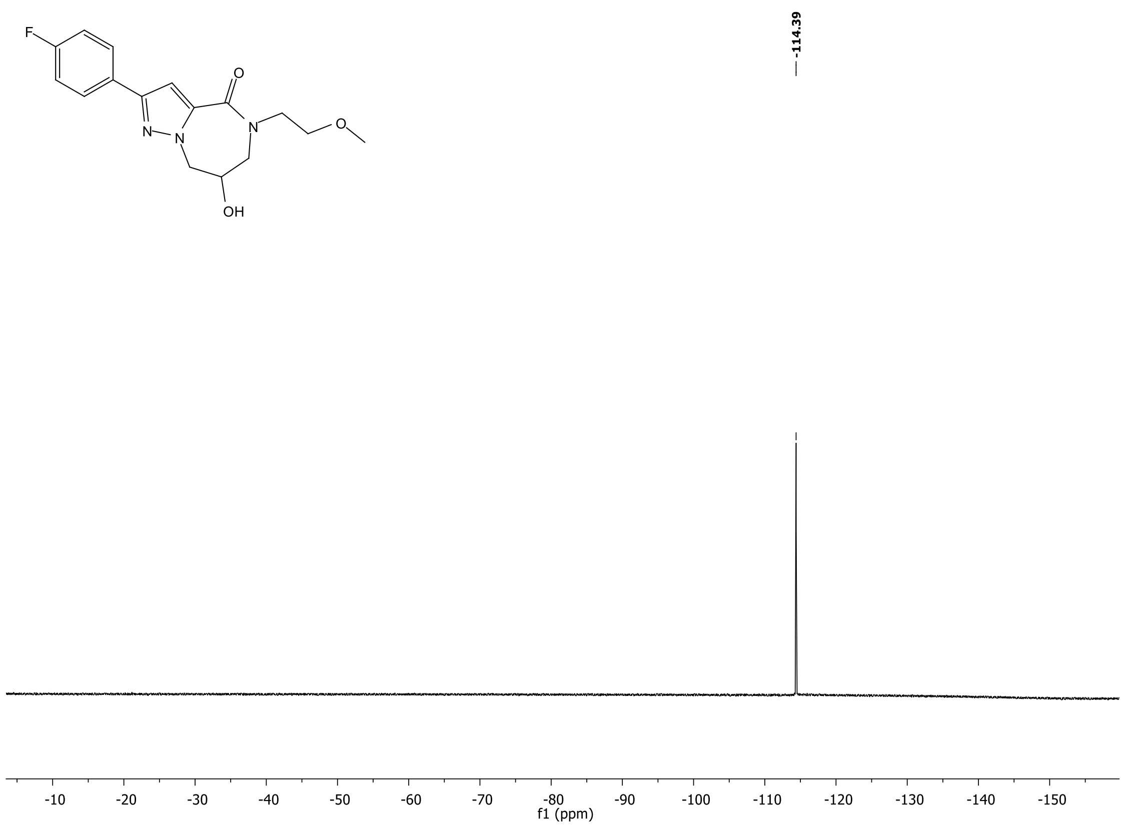

Figure S90. ^{19}F NMR spectrum (376 MHz, DMSO-d_6) of 2-(4-fluorophenyl)-7-hydroxy-5-(2-methoxyethyl)-5,6,7,8-tetrahydro-4*H*-pyrazolo[1,5-*a*][1,4]diazepin-4-one (**4j**).

Compound Spectrum SmartFormula Report

Analysis Info

Analysis Name	D:\Data\KDD-132.d	Acquisition Date	4/7/2020 3:30:32 PM
Method	DirectInfusion_TuneLow_pos.m	Operator	hplc
Sample Name	KDD-132	Instrument	micrOTOF-Q III 8228888.20448
Comment	SB		

Acquisition Parameter

Source Type	ESI	Ion Polarity	Positive	Set Nebulizer	0.4 Bar
Focus	Not active	Set Capillary	4500 V	Set Dry Heater	180 °C
Scan Begin	50 m/z	Set End Plate Offset	-500 V	Set Dry Gas	4.0 l/min
Scan End	1000 m/z	Set Collision Cell RF	140.0 Vpp	Set Divert Valve	Waste

#	RT [min]	Area	Int. Type	I	S/N	Chromatogram	Max. m/z	FWHM [min]
n.a.	0.5	n.a.	Single spectrum	n.a.	n.a.	n.a.	226.9515	n.a.
n.a.	5.9	n.a.	Single spectrum	n.a.	n.a.	n.a.	342.1224	n.a.

+MS, 5.9min #356

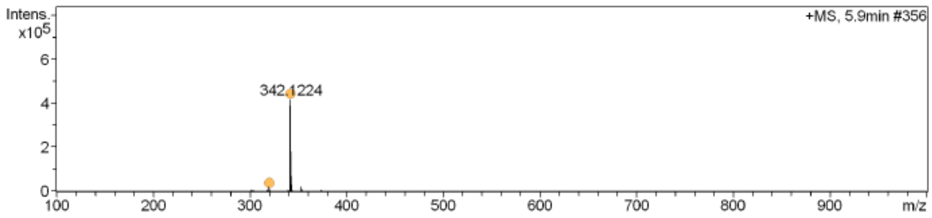

Meas. m/z	#	Ion Formula	m/z	err [ppm]	mSigma	# Sigma	Score	rdb	e⁻ Conf	N-Rule
320.1400	1	C16H19FN3O3	320.1405	1.6	22.3	1	100.00	8.5	even	ok
342.1224	1	C16H18FN3NaO3	342.1224	-0.0	5.9	1	100.00	8.5	even	ok

Figure S91. HRMS (ESI-TOF) spectrum of 2-(4-fluorophenyl)-7-hydroxy-5-(2-methoxyethyl)-5,6,7,8-tetrahydro-4*H*-pyrazolo[1,5-*a*][1,4]diazepin-4-one (**4j**).

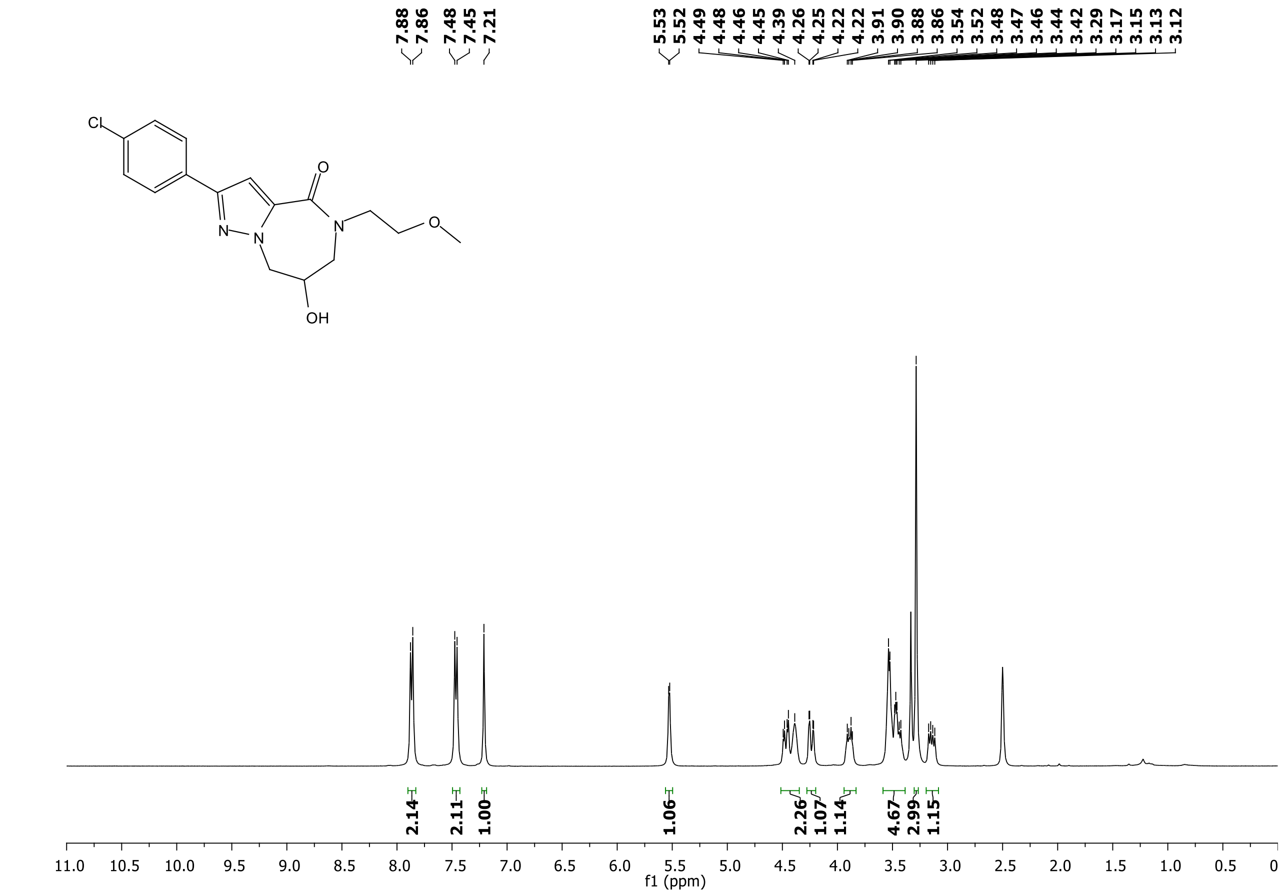

Figure S92. ^{1}H NMR spectrum (400 MHz, DMSO-*d*$_6$) of 2-(4-chlorophenyl)-7-hydroxy-5-(2-methoxyethyl)-5,6,7,8-tetrahydro-4*H*-pyrazolo[1,5-*a*][1,4]diazepin-4-one (**4k**).

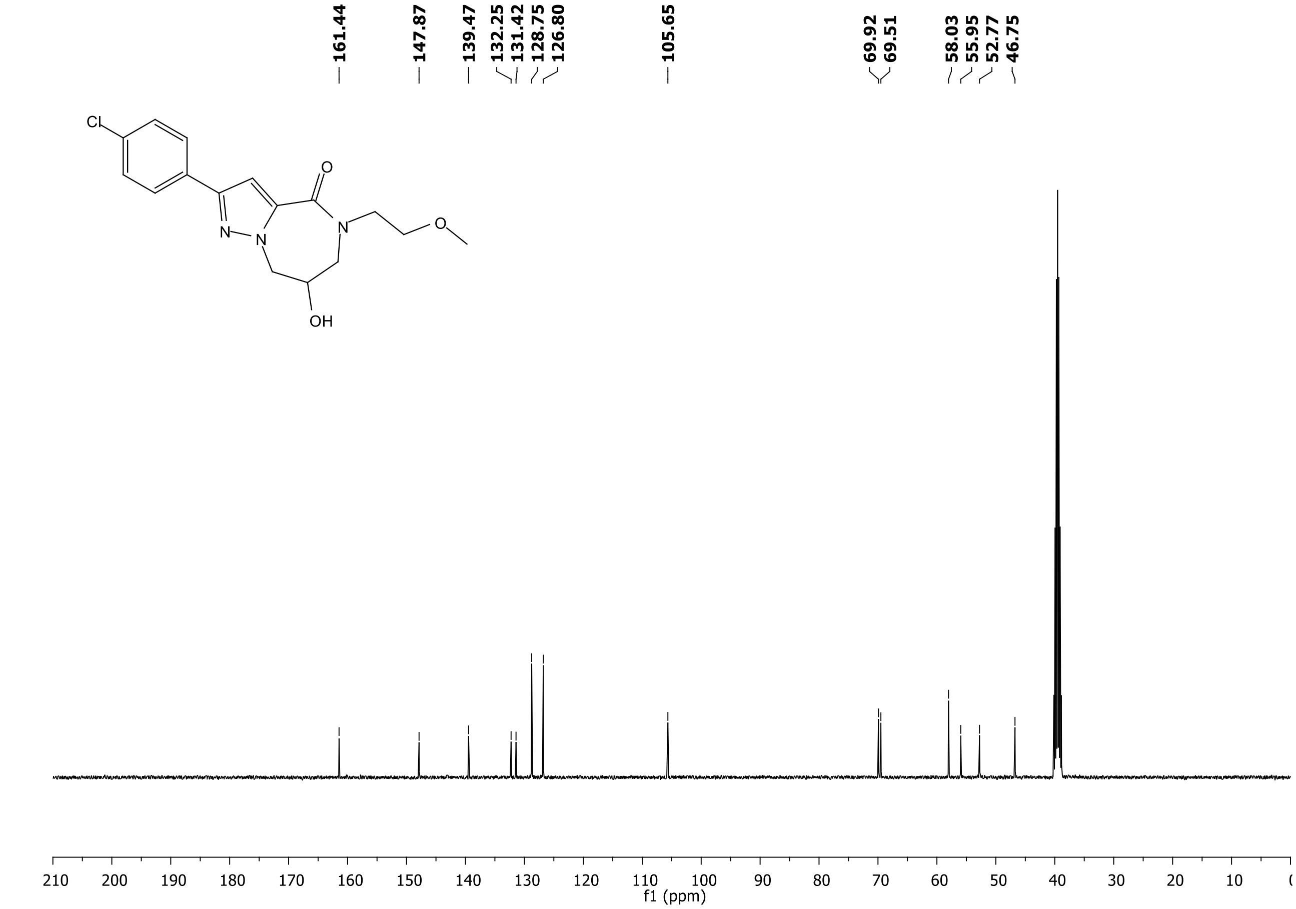

Figure S93. ^{13}C NMR spectrum (101 MHz, DMSO-*d*$_6$) of 2-(4-chlorophenyl)-7-hydroxy-5-(2-methoxyethyl)-5,6,7,8-tetrahydro-4*H*-pyrazolo[1,5-*a*][1,4]diazepin-4-one (**4k**).

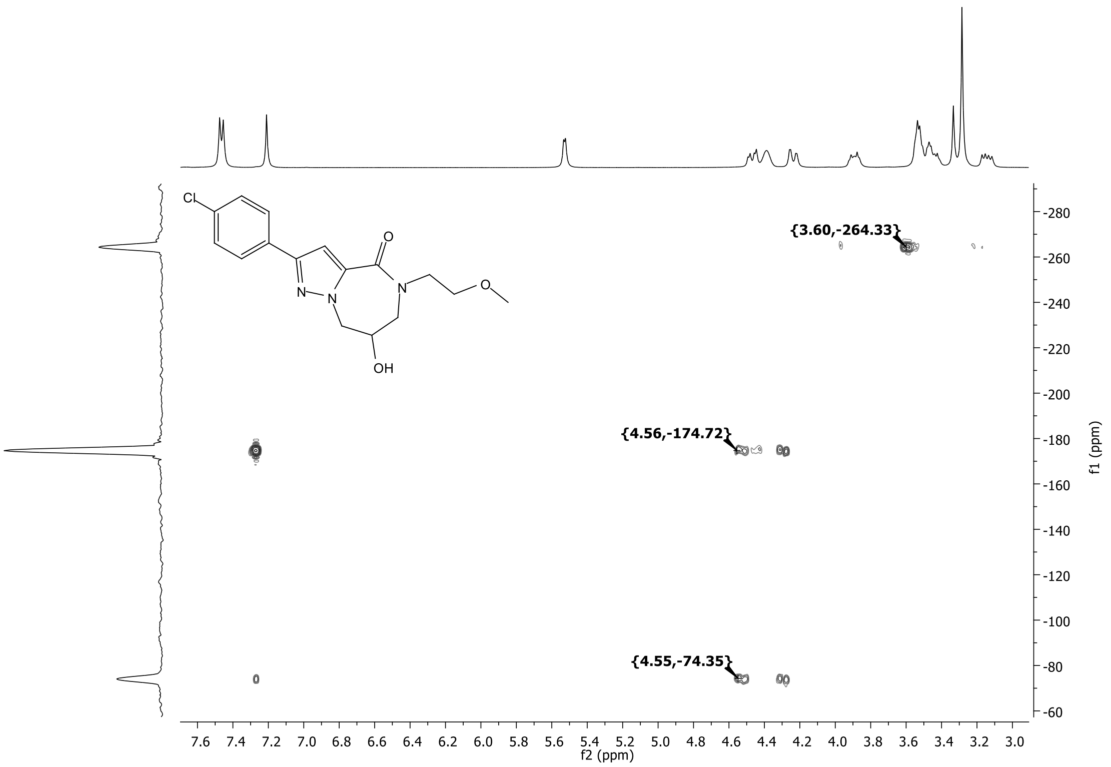

Figure S94. $^{1}H,^{15}N$-HMBC (40 MHz, DMSO-d_6) of 2-(4-chlorophenyl)-7-hydroxy-5-(2-methoxyethyl)-5,6,7,8-tetrahydro-4*H*-pyrazolo[1,5-*a*][1,4]diazepin-4-one (**4k**).

Compound Spectrum SmartFormula Report

Analysis Info

Analysis Name D:\Data\KDD-131.d
Method DirectInfusion_TuneLow_pos.m
Sample Name KDD-131
Comment SB

Acquisition Date 4/7/2020 2:57:02 PM
Operator hplc
Instrument micrOTOF-Q III 8228888.20448

Acquisition Parameter

Source Type	ESI	Ion Polarity	Positive	Set Nebulizer	0.4 Bar
Focus	Not active	Set Capillary	4500 V	Set Dry Heater	180 °C
Scan Begin	50 m/z	Set End Plate Offset	-500 V	Set Dry Gas	4.0 l/min
Scan End	1000 m/z	Set Collision Cell RF	140.0 Vpp	Set Divert Valve	Waste

#	RT [min]	Area	Int. Type	I	S/N	Chromatogram	Max. m/z	FWHM [min]
n.a.	0.3	n.a.	Single spectrum	n.a.	n.a.	n.a.	226.9516	n.a.
n.a.	5.4	n.a.	Single spectrum	n.a.	n.a.	n.a.	358.0929	n.a.

+MS, 5.4min #325

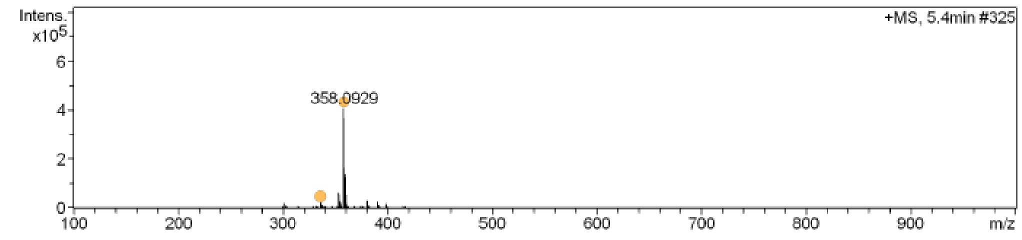

Meas. m/z	#	Ion Formula	m/z	err [ppm]	mSigma	# Sigma	Score	rdb	e⁻ Conf	N-Rule
336.1113	1	C16H19ClN3O3	336.1109	0.9	9.1	1	100.00	8.5	even	ok
358.0929	1	C16H18ClN3NaO3	358.0929	-0.1	1.9	1	100.00	8.5	even	ok

Figure S95. HRMS (ESI-TOF) spectrum of 2-(4-chlorophenyl)-7-hydroxy-5-(2-methoxyethyl)-5,6,7,8-tetrahydro-4*H*-pyrazolo[1,5-*a*][1,4]diazepin-4-one (**4k**).

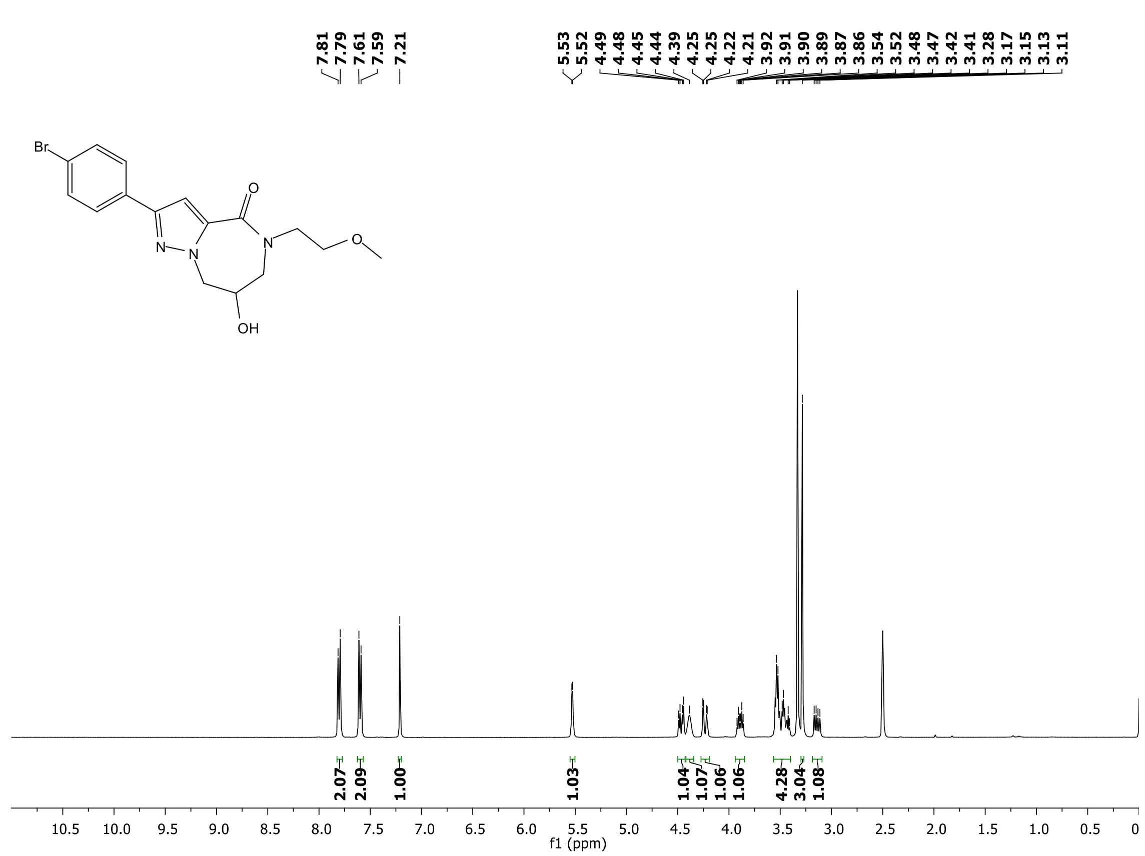

Figure S96. ^{1}H NMR spectrum (400 MHz, DMSO-*d*$_6$) of 2-(4-bromophenyl)-7-hydroxy-5-(2-methoxyethyl)-5,6,7,8-tetrahydro-4*H*-pyrazolo[1,5-*a*][1,4]diazepin-4-one (**4l**).

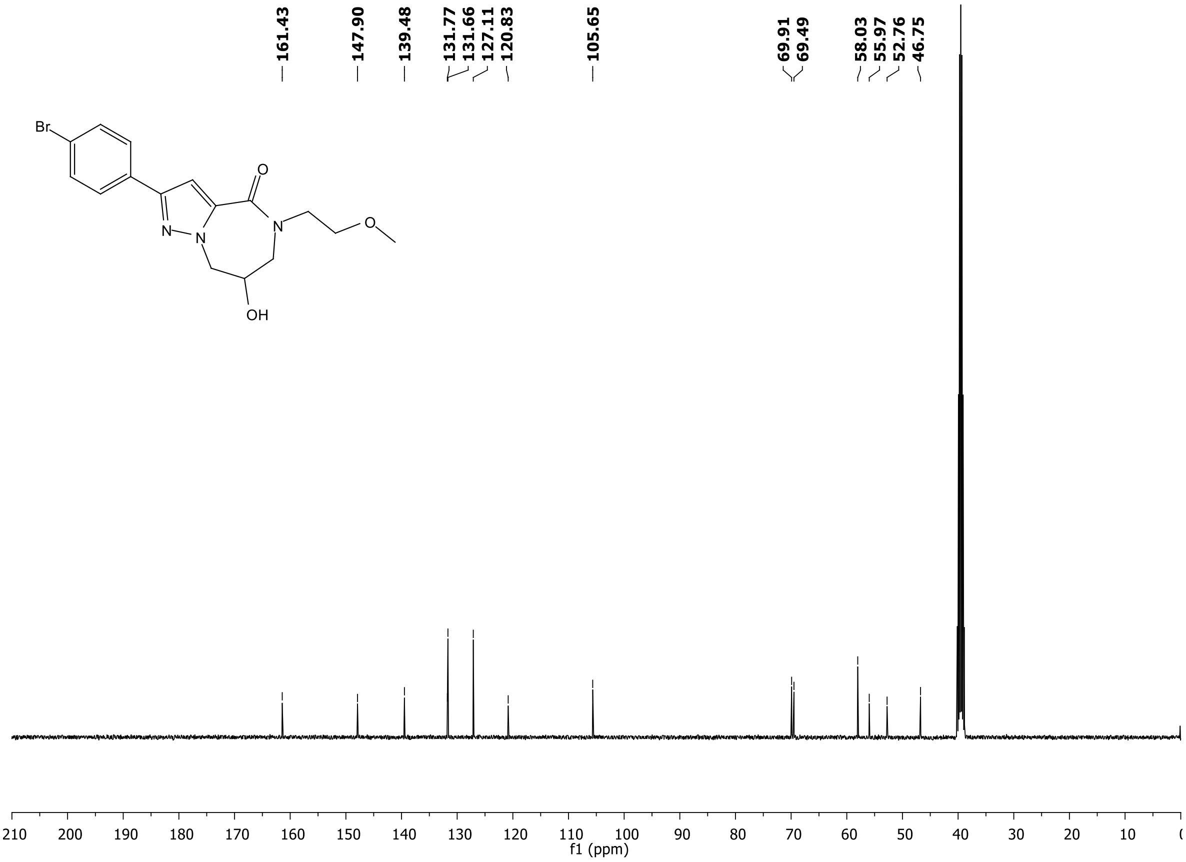

Figure S97. ^{13}C NMR spectrum (101 MHz, DMSO-*d*$_6$) of 2-(4-bromophenyl)-7-hydroxy-5-(2-methoxyethyl)-5,6,7,8-tetrahydro-4*H*-pyrazolo[1,5-*a*][1,4]diazepin-4-one (**4l**).

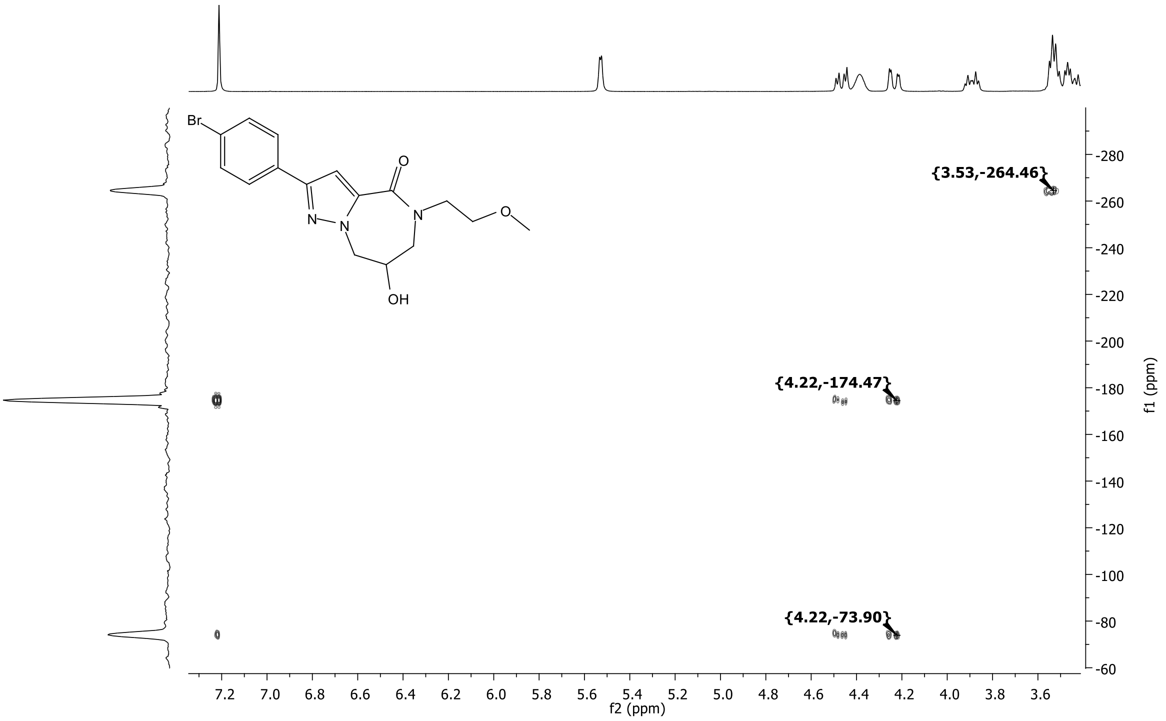

Figure S98. ^{1}H,^{15}N-HMBC (40 MHz, DMSO-d_6) of 2-(4-bromophenyl)-7-hydroxy-5-(2-methoxyethyl)-5,6,7,8-tetrahydro-4*H*-pyrazolo[1,5-*a*][1,4]diazepin-4-one (**4l**).

Compound Spectrum SmartFormula Report

Analysis Info

Analysis Name	D:\Data\KDD-116.d	Acquisition Date	4/6/2020 11:20:37 AM
Method	DirectInfusion_TuneLow_pos.m	Operator	hplc
Sample Name	KDD-116	Instrument	micrOTOF-Q III 8228888.20448
Comment	SB		

Acquisition Parameter

Source Type	ESI	Ion Polarity	Positive	Set Nebulizer	0.4 Bar
Focus	Not active	Set Capillary	4500 V	Set Dry Heater	180 °C
Scan Begin	50 m/z	Set End Plate Offset	-500 V	Set Dry Gas	4.0 l/min
Scan End	1000 m/z	Set Collision Cell RF	140.0 Vpp	Set Divert Valve	Waste

#	RT [min]	Area	Int. Type	I	S/N	Chromatogram	Max. m/z	FWHM [min]
n.a.	0.3	n.a.	Single spectrum	n.a.	n.a.	n.a.	226.9519	n.a.
n.a.	6.8	n.a.	Single spectrum	n.a.	n.a.	n.a.	402.0424	n.a.

+MS, 6.8min #409

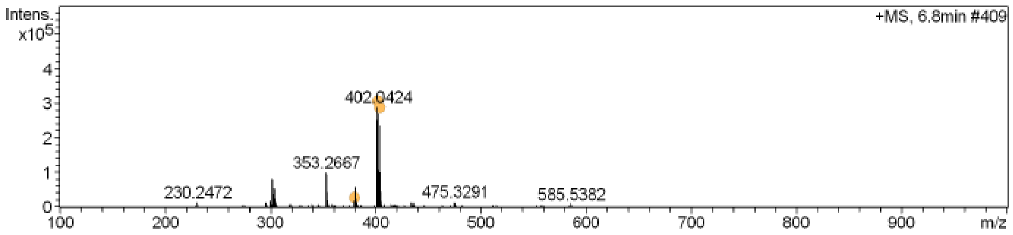

Meas. m/z	#	Ion Formula	m/z	err [ppm]	mSigma	# Sigma	Score	rdb	e⁻ Conf	N-Rule
380.0605	1	C16H19BrN3O3	380.0604	0.1	38.6	1	100.00	8.5	even	ok
402.0424	1	C16H18BrN3NaO3	402.0424	-0.1	28.5	1	100.00	8.5	even	ok
404.0409	1	C16H18BrN3NaO3	402.0424	0.9	28.5	1	100.00	8.5	even	ok

Figure S99. HRMS (ESI-TOF) spectrum of 2-(4-bromophenyl)-7-hydroxy-5-(2-methoxyethyl)-5,6,7,8-tetrahydro-4*H*-pyrazolo[1,5-*a*][1,4]diazepin-4-one (**4l**).

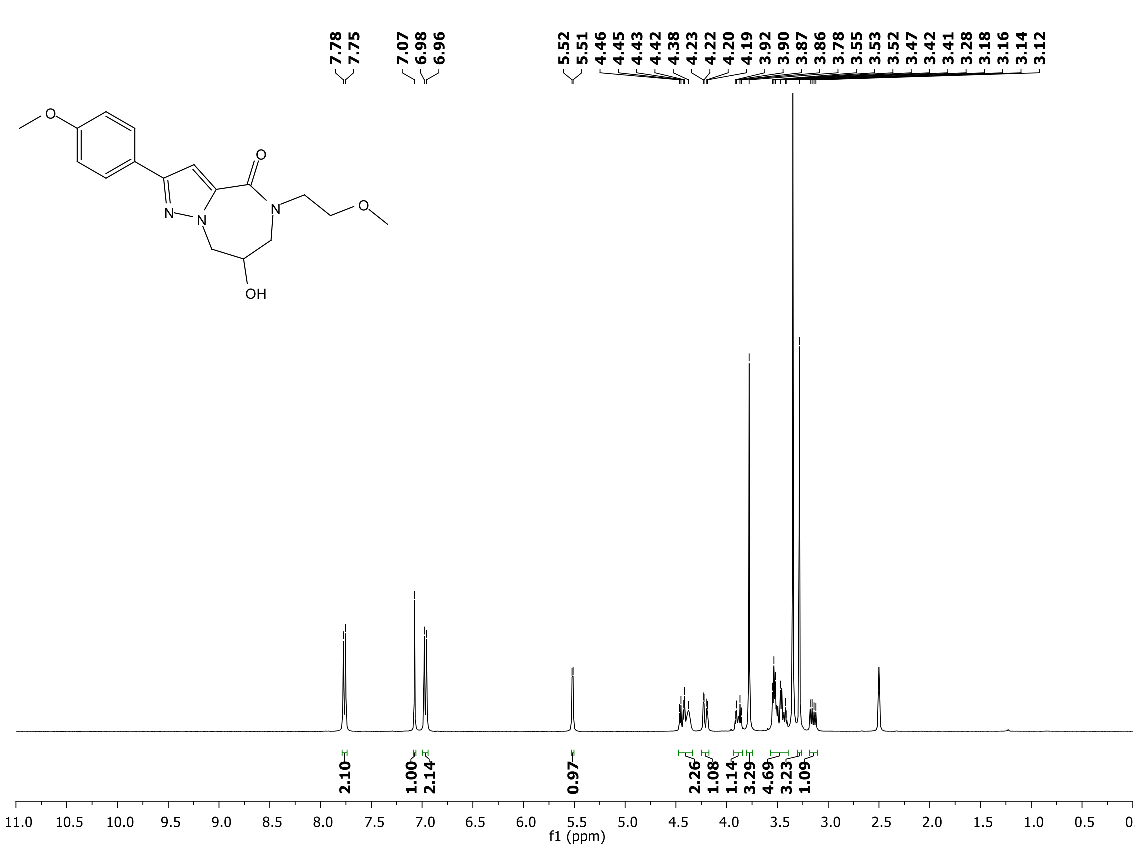

Figure S100. ^{1}H NMR spectrum (400 MHz, DMSO-d_6) of 7-hydroxy-5-(2-methoxyethyl)-2-(4-methoxyphenyl)-5,6,7,8-tetrahydro-4*H*-pyrazolo[1,5-*a*][1,4]diazepin-4-one (**4m**).

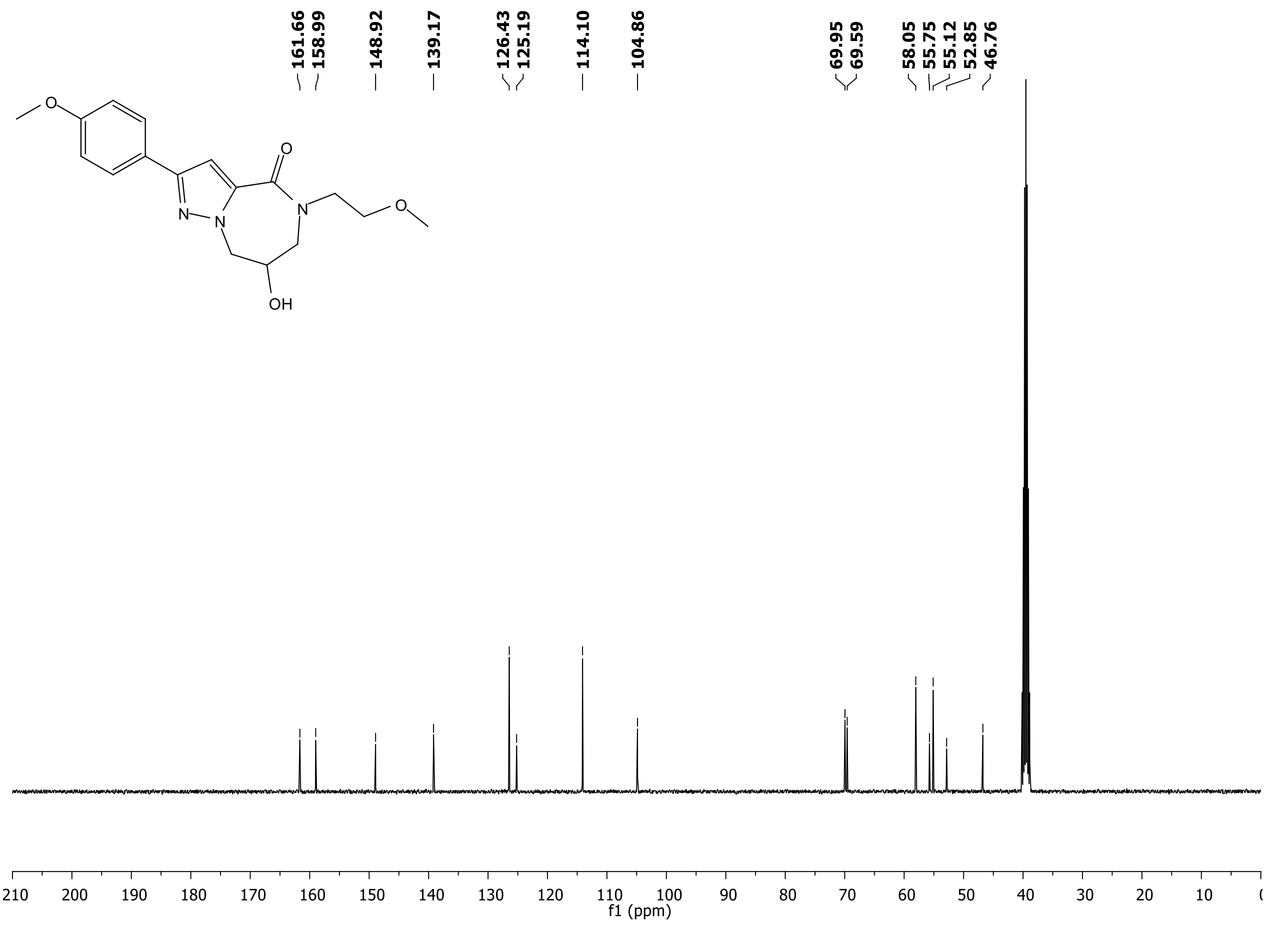

Figure S101. ^{13}C NMR spectrum (101 MHz, DMSO-d_6) of 7-hydroxy-5-(2-methoxyethyl)-2-(4-methoxyphenyl)-5,6,7,8-tetrahydro-4*H*-pyrazolo[1,5-*a*][1,4]diazepin-4-one (**4m**).

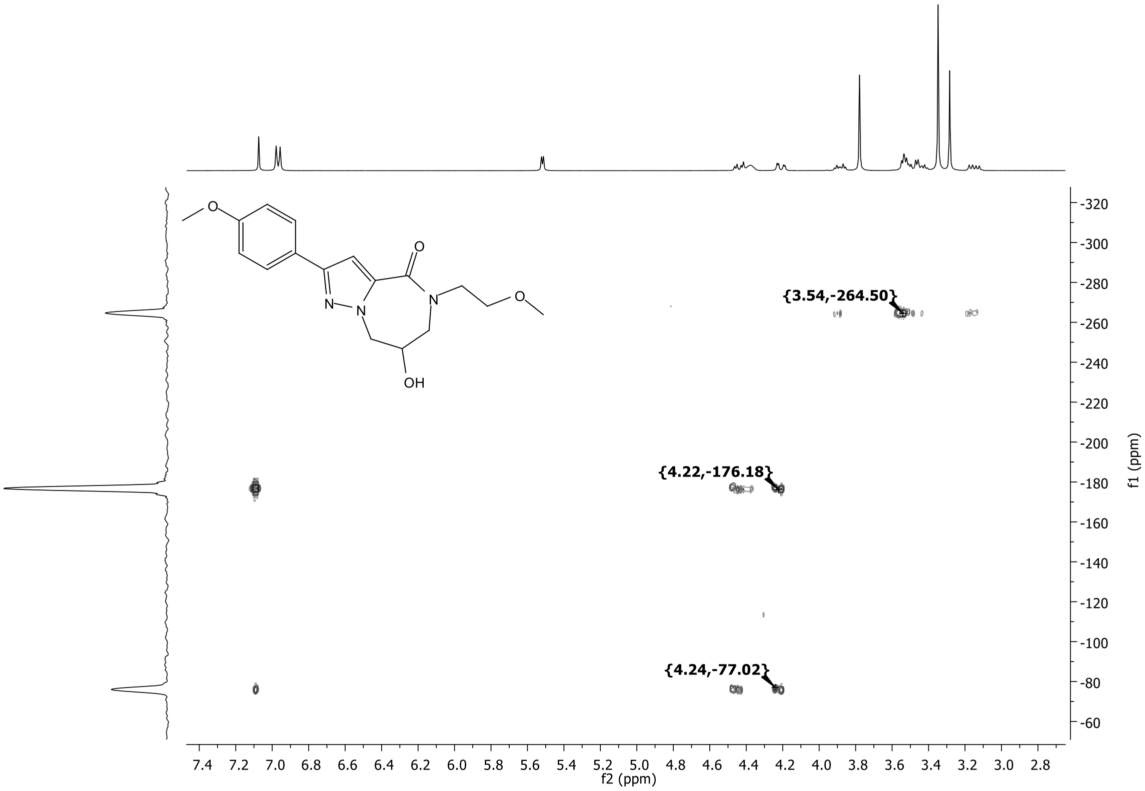

Figure S102. ^{1}H,^{15}N-HMBC (40 MHz, DMSO-*d*$_6$) of 7-hydroxy-5-(2-methoxyethyl)-2-(4-methoxyphenyl)-5,6,7,8-tetrahydro-4*H*-pyrazolo[1,5-*a*][1,4]diazepin-4-one (**4m**).

Compound Spectrum SmartFormula Report

Analysis Info

Acquisition Date 8/10/2021 11:26:07 AM

Analysis Name D:\Data\KDD-177-1.d
Method DirectInfusion_TuneLow_pos.m
Sample Name KDD-177-1
Comment SB

Operator hplc
Instrument micrOTOF-Q III 8228888.20448

Acquisition Parameter

Source Type	ESI	Ion Polarity	Positive	Set Nebulizer	0.4 Bar
Focus	Not active	Set Capillary	4500 V	Set Dry Heater	180 °C
Scan Begin	50 m/z	Set End Plate Offset	-500 V	Set Dry Gas	4.0 l/min
Scan End	1000 m/z	Set Collision Cell RF	140.0 Vpp	Set Divert Valve	Waste

#	RT [min]	Area	Int. Type	I	S/N	Chromatogram	Max. m/z	FWHM [min]
n.a.	0.5	n.a.	Single spectrum	n.a.	n.a.	n.a.	226.9517	n.a.
n.a.	4.9	n.a.	Single spectrum	n.a.	n.a.	n.a.	354.1424	n.a.

+MS, 4.9min #294

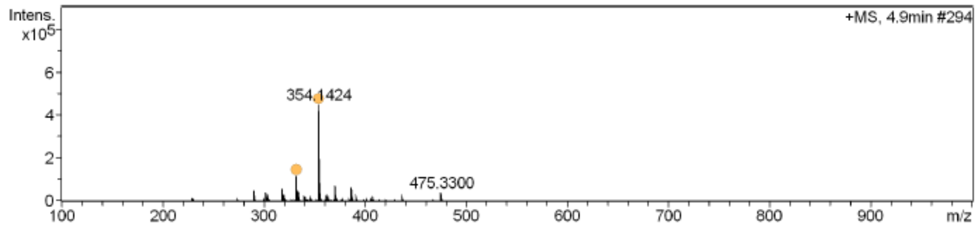

Meas. m/z	#	Ion Formula	m/z	err [ppm]	mSigma	# Sigma	Score	rdb	e⁻ Conf	N-Rule
332.1602	1	C17H22N3O4	332.1605	-0.8	8.9	1	100.00	8.5	even	ok
354.1424	1	C17H21N3NaO4	354.1424	0.1	1.7	1	100.00	8.5	even	ok

Figure S103. HRMS (ESI-TOF) spectrum of 7-hydroxy-5-(2-methoxyethyl)-2-(4-methoxyphenyl)-5,6,7,8-tetrahydro-4*H*-pyrazolo[1,5-*a*][1,4]diazepin-4-one (**4m**).

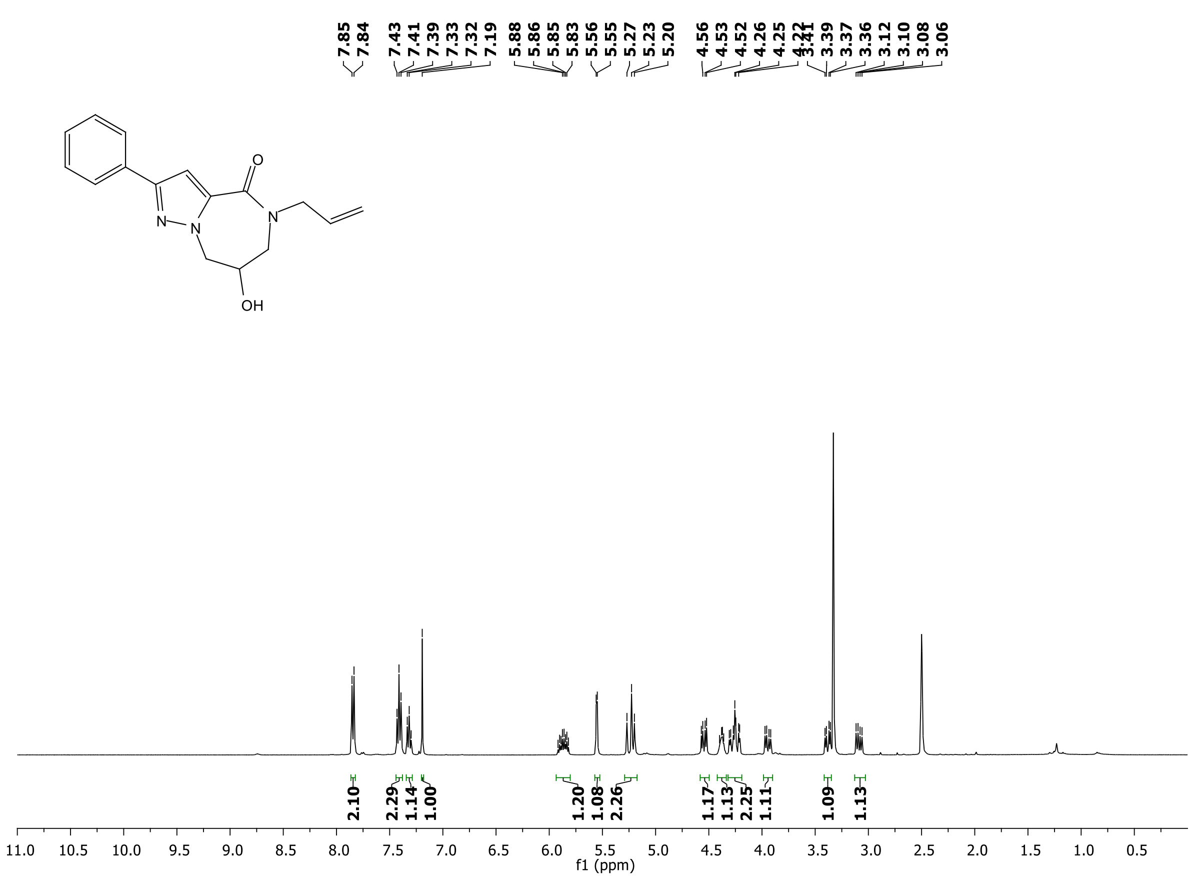

Figure S104. ^{1}H NMR spectrum (400 MHz, DMSO-d_6) of 5-allyl-7-hydroxy-2-phenyl-5,6,7,8-tetrahydro-4*H*-pyrazolo[1,5-*a*][1,4]diazepin-4-one (**4n**).

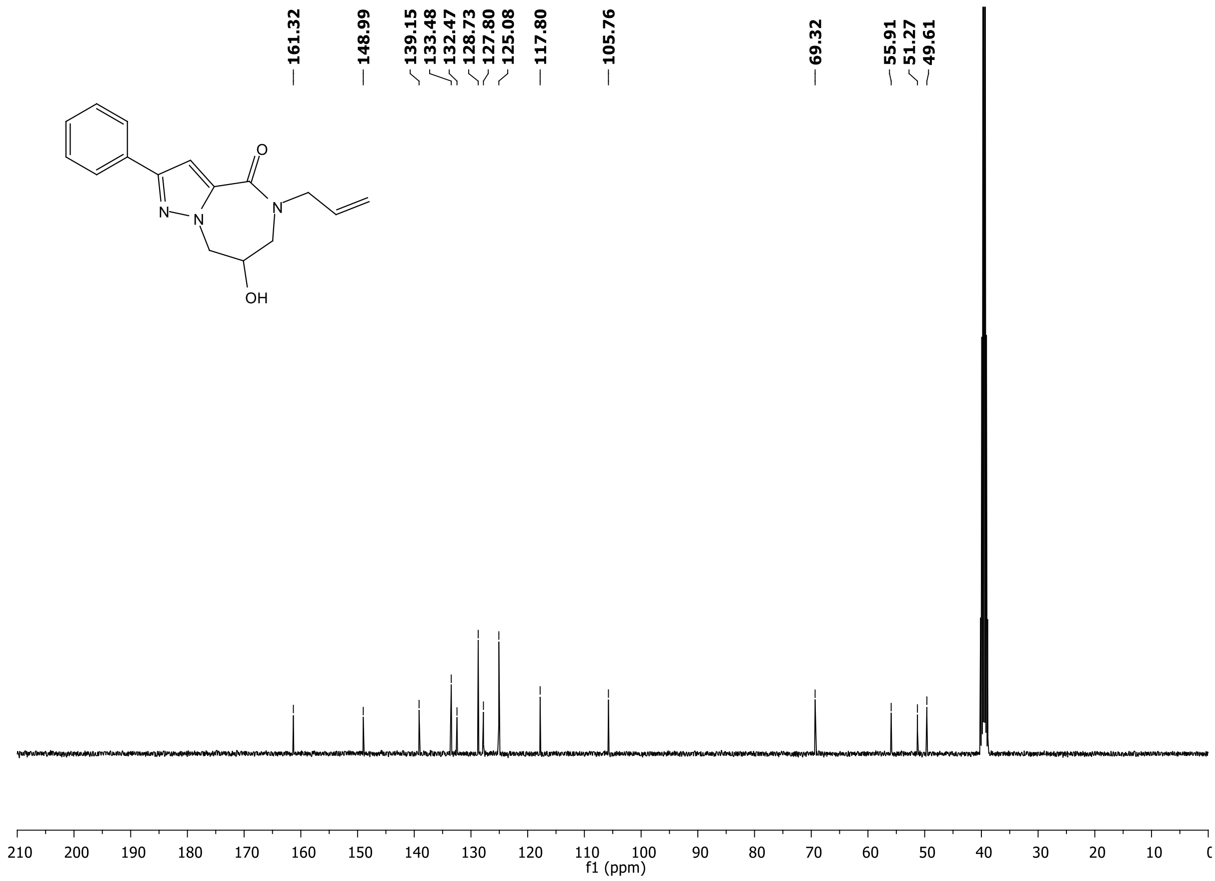

Figure S105. ^{13}C NMR spectrum (101 MHz, DMSO-d_6) of 5-allyl-7-hydroxy-2-phenyl-5,6,7,8-tetrahydro-4*H*-pyrazolo[1,5-*a*][1,4]diazepin-4-one (**4n**).

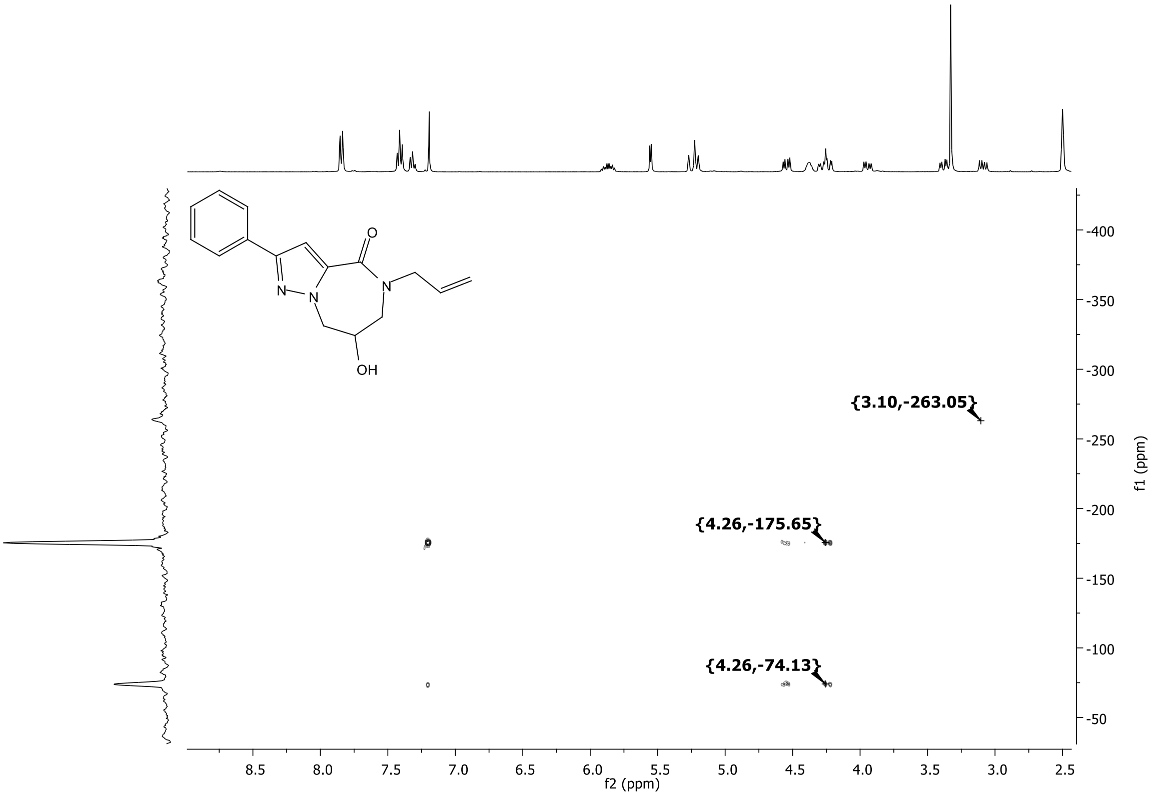

Figure S106. ^{1}H,^{15}N-HMBC (40 MHz, DMSO-d_6) of 5-allyl-7-hydroxy-2-phenyl-5,6,7,8-tetrahydro-4*H*-pyrazolo[1,5-*a*][1,4]diazepin-4-one (**4n**).

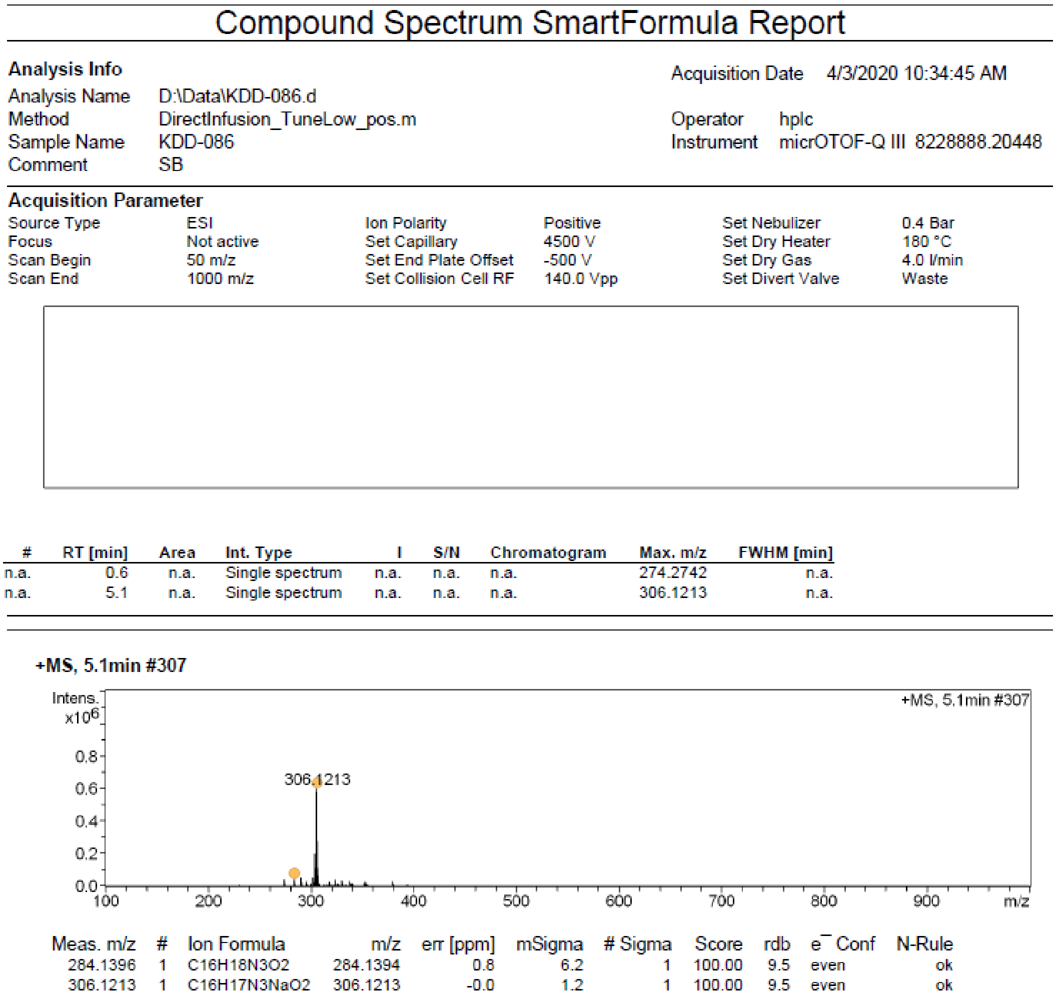

Compound Spectrum SmartFormula Report

Analysis Info

Analysis Name	D:\Data\KDD-086.d	Acquisition Date	4/3/2020 10:34:45 AM
Method	DirectInfusion_TuneLow_pos.m	Operator	hplc
Sample Name	KDD-086	Instrument	micrOTOF-Q III 8228888.20448
Comment	SB		

Acquisition Parameter

Source Type	ESI	Ion Polarity	Positive	Set Nebulizer	0.4 Bar
Focus	Not active	Set Capillary	4500 V	Set Dry Heater	180 °C
Scan Begin	50 m/z	Set End Plate Offset	-500 V	Set Dry Gas	4.0 l/min
Scan End	1000 m/z	Set Collision Cell RF	140.0 Vpp	Set Divert Valve	Waste

#	RT [min]	Area	Int. Type	I	S/N	Chromatogram	Max. m/z	FWHM [min]
n.a.	0.6	n.a.	Single spectrum	n.a.	n.a.	n.a.	274.2742	n.a.
n.a.	5.1	n.a.	Single spectrum	n.a.	n.a.	n.a.	306.1213	n.a.

Meas. m/z	#	Ion Formula	m/z	err [ppm]	mSigma	# Sigma	Score	rdb	e⁻ Conf	N-Rule
284.1396	1	C16H18N3O2	284.1394	0.8	6.2	1	100.00	9.5	even	ok
306.1213	1	C16H17N3NaO2	306.1213	-0.0	1.2	1	100.00	9.5	even	ok

Figure S107. HRMS (ESI-TOF) spectrum of 5-allyl-7-hydroxy-2-phenyl-5,6,7,8-tetrahydro-4*H*-pyrazolo[1,5-*a*][1,4]diazepin-4-one (**4n**).

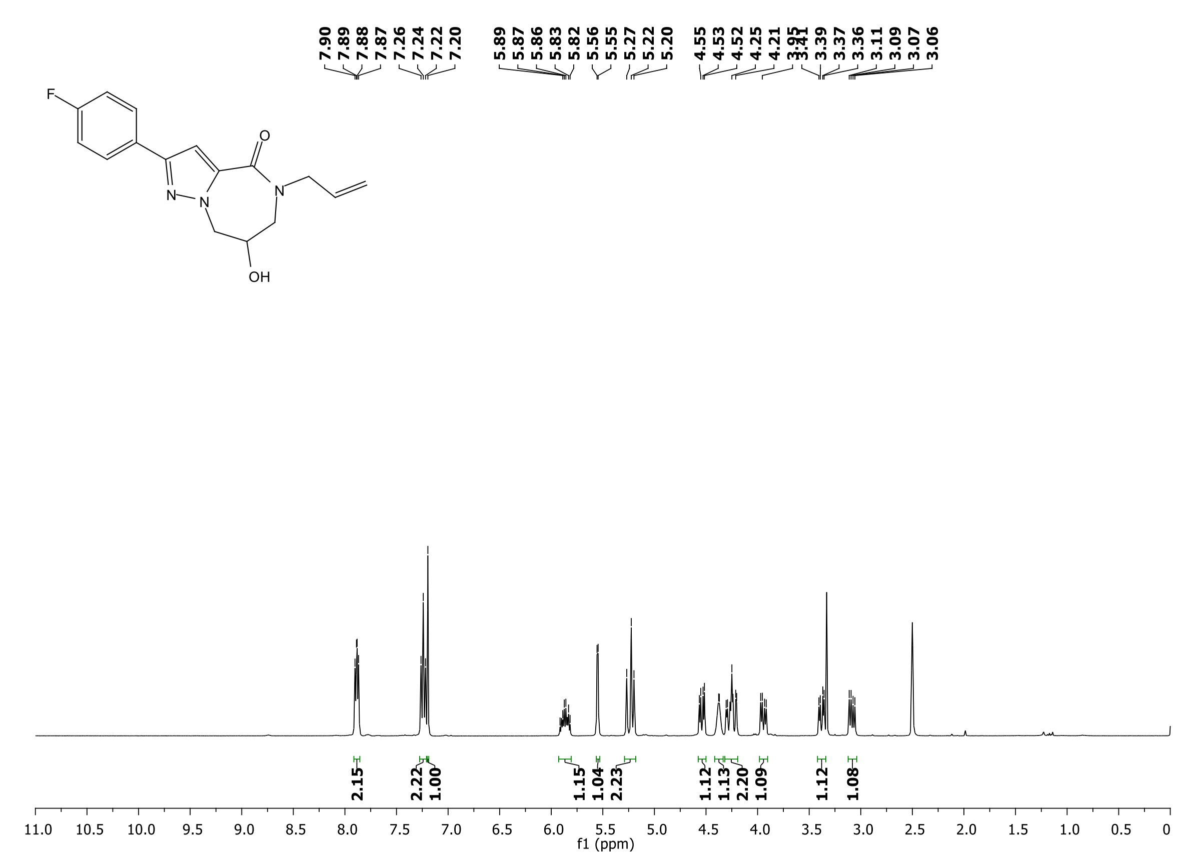

Figure S108. ^{1}H NMR spectrum (400 MHz, DMSO-d_6) of 5-allyl-2-(4-fluorophenyl)-7-hydroxy-5,6,7,8-tetrahydro-4*H*-pyrazolo[1,5-*a*][1,4]diazepin-4-one (**4o**).

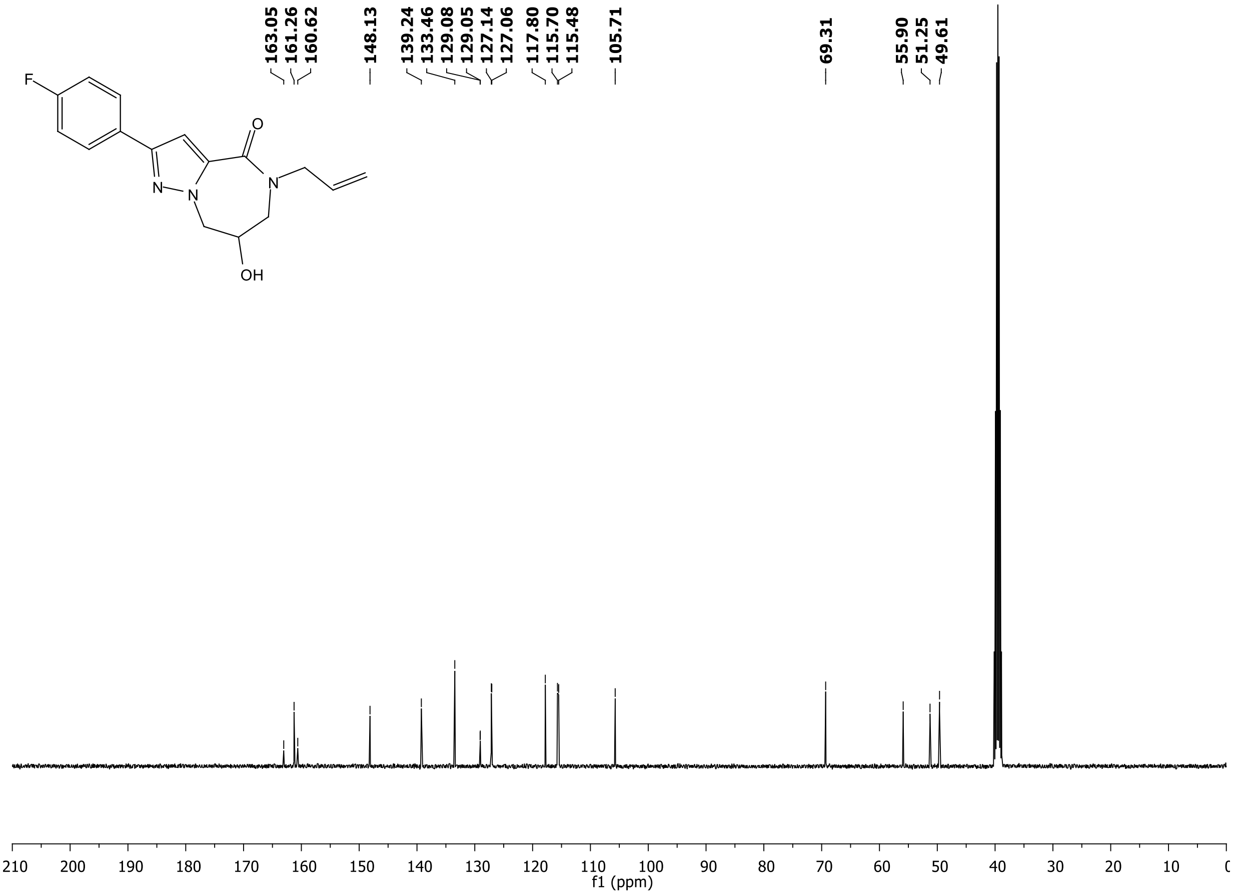

Figure S109. ^{13}C NMR spectrum (101 MHz, DMSO-d_6) of 5-allyl-2-(4-fluorophenyl)-7-hydroxy-5,6,7,8-tetrahydro-4*H*-pyrazolo[1,5-*a*][1,4]diazepin-4-one (**4o**).

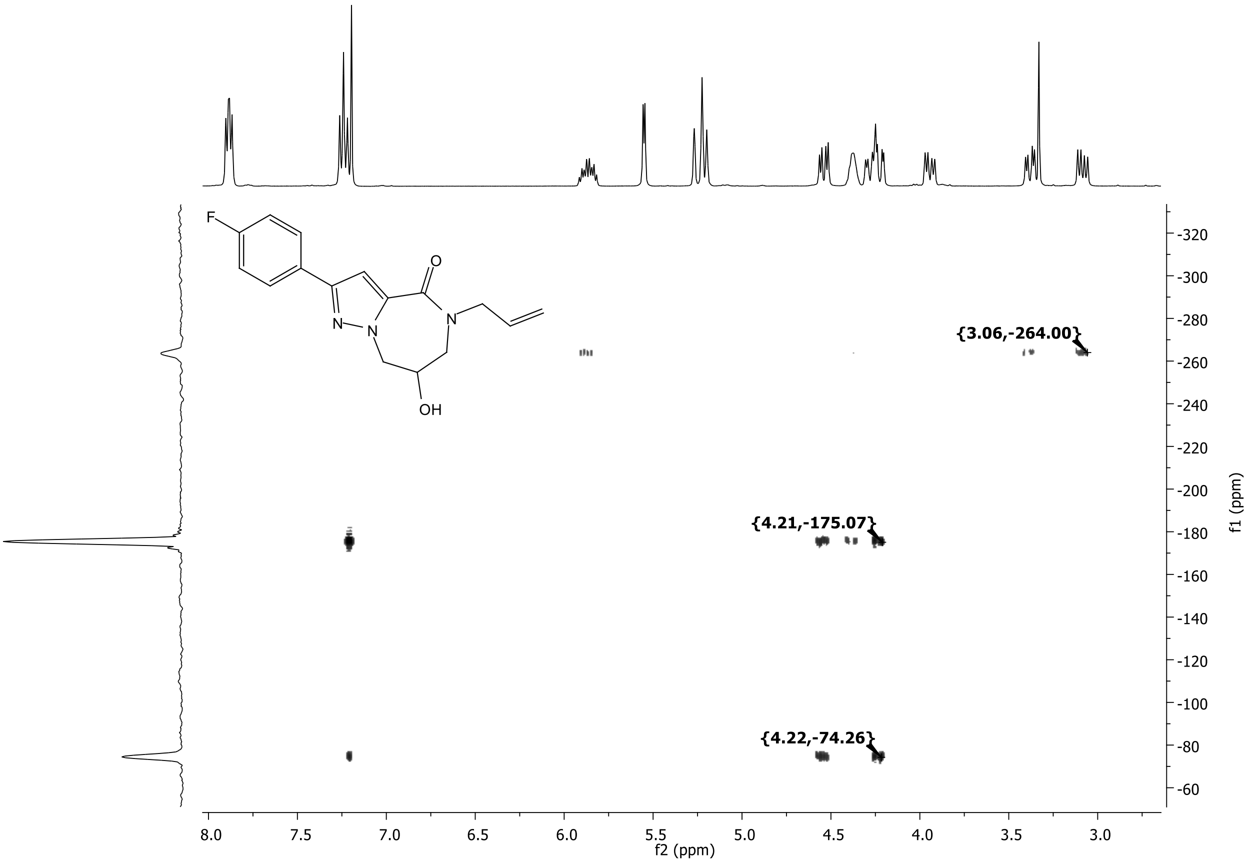

Figure S110. $^{1}H,^{15}N$-HMBC (40 MHz, DMSO-*d*$_6$) of 5-allyl-2-(4-fluorophenyl)-7-hydroxy-5,6,7,8-tetrahydro-4*H*-pyrazolo[1,5-*a*][1,4]diazepin-4-one (**4o**).

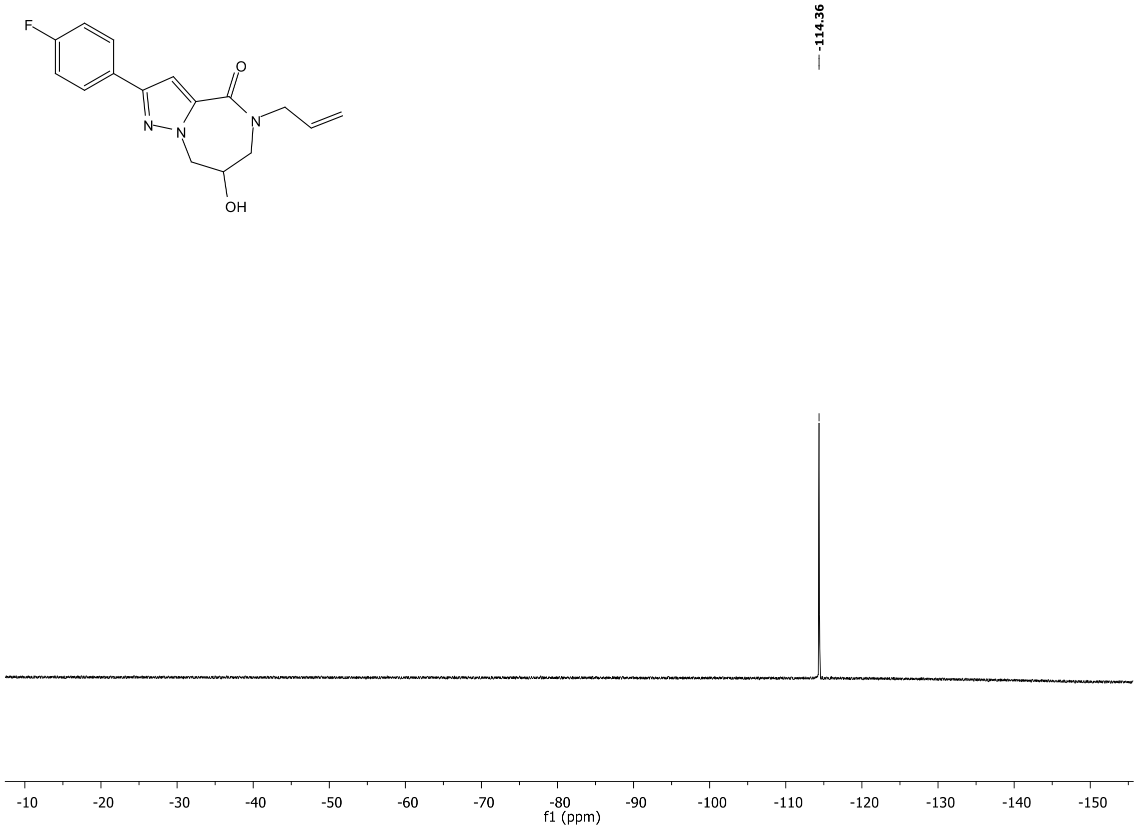

Figure S111. ^{19}F NMR spectrum (376 MHz, DMSO-*d*$_6$) of 5-allyl-2-(4-fluorophenyl)-7-hydroxy-5,6,7,8-tetrahydro-4*H*-pyrazolo[1,5-*a*][1,4]diazepin-4-one (**4o**).

Compound Spectrum SmartFormula Report

Analysis Info

Analysis Name	D:\Data\KDD-128.d	Acquisition Date	4/6/2020 3:56:54 PM
Method	DirectInfusion_TuneLow_pos.m	Operator	hplc
Sample Name	KDD-128	Instrument	micrOTOF-Q III 8228888.20448
Comment	SB		

Acquisition Parameter

Source Type	ESI	Ion Polarity	Positive	Set Nebulizer	0.4 Bar
Focus	Not active	Set Capillary	4500 V	Set Dry Heater	180 °C
Scan Begin	50 m/z	Set End Plate Offset	-500 V	Set Dry Gas	4.0 l/min
Scan End	1000 m/z	Set Collision Cell RF	140.0 Vpp	Set Divert Valve	Waste

#	RT [min]	Area	Int. Type	I	S/N	Chromatogram	Max. m/z	FWHM [min]
n.a.	1.4	n.a.	Single spectrum	n.a.	n.a.	n.a.	226.9514	n.a.
n.a.	6.5	n.a.	Single spectrum	n.a.	n.a.	n.a.	324.1119	n.a.

+MS, 6.5min #392

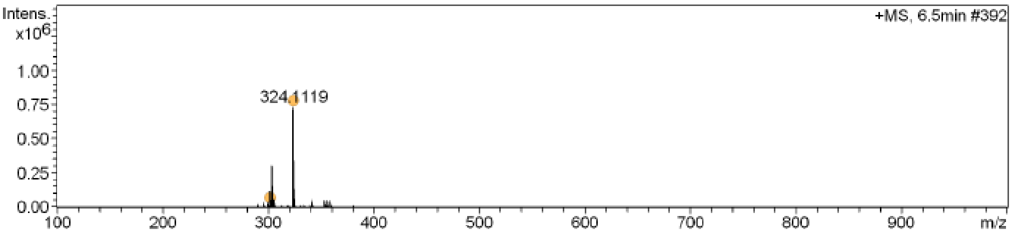

Meas. m/z	#	Ion Formula	m/z	err [ppm]	mSigma	# Sigma	Score	rdb	e⁻ Conf	N-Rule
302.1295	1	C16H17FN3O2	302.1299	1.5	57.2	1	100.00	9.5	even	ok
324.1119	1	C16H16FN3NaO2	324.1119	-0.1	4.9	1	100.00	9.5	even	ok

Figure S112. HRMS (ESI-TOF) spectrum of 5-allyl-2-(4-fluorophenyl)-7-hydroxy-5,6,7,8-tetrahydro-4*H*-pyrazolo[1,5-*a*][1,4]diazepin-4-one (**4o**).

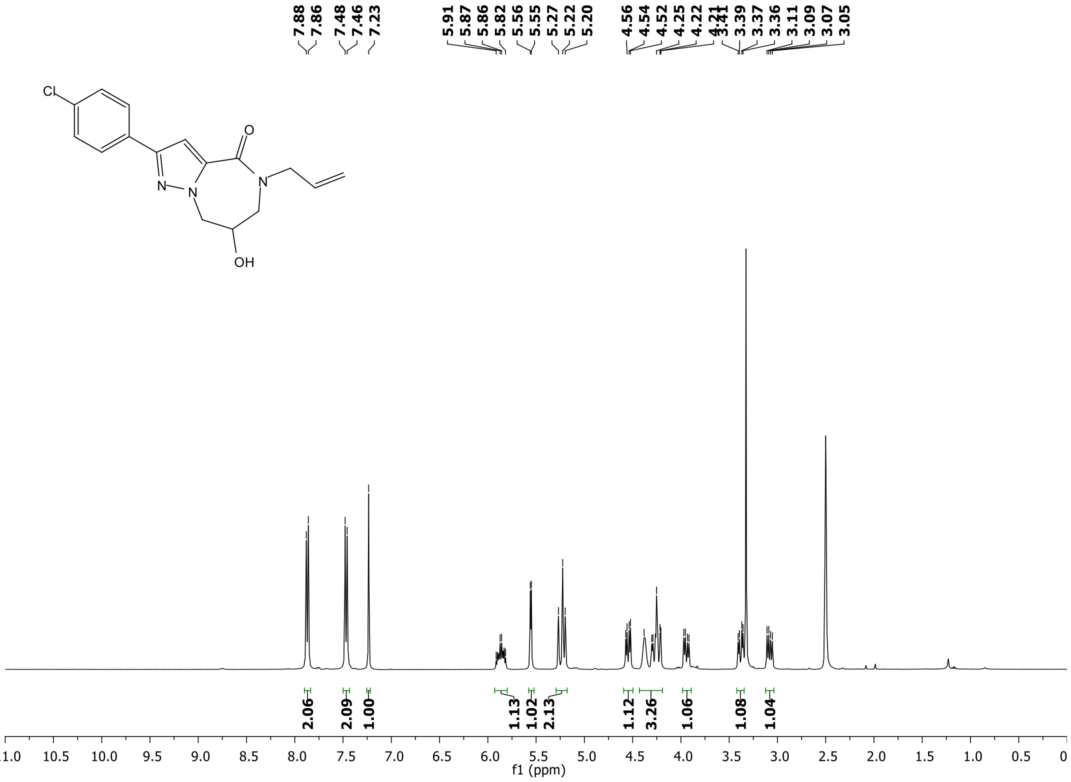

Figure S113. ^{1}H NMR spectrum (400 MHz, DMSO-d_6) of 5-allyl-2-(4-chlorophenyl)-7-hydroxy-5,6,7,8-tetrahydro-4*H*-pyrazolo[1,5-*a*][1,4]diazepin-4-one (**4p**).

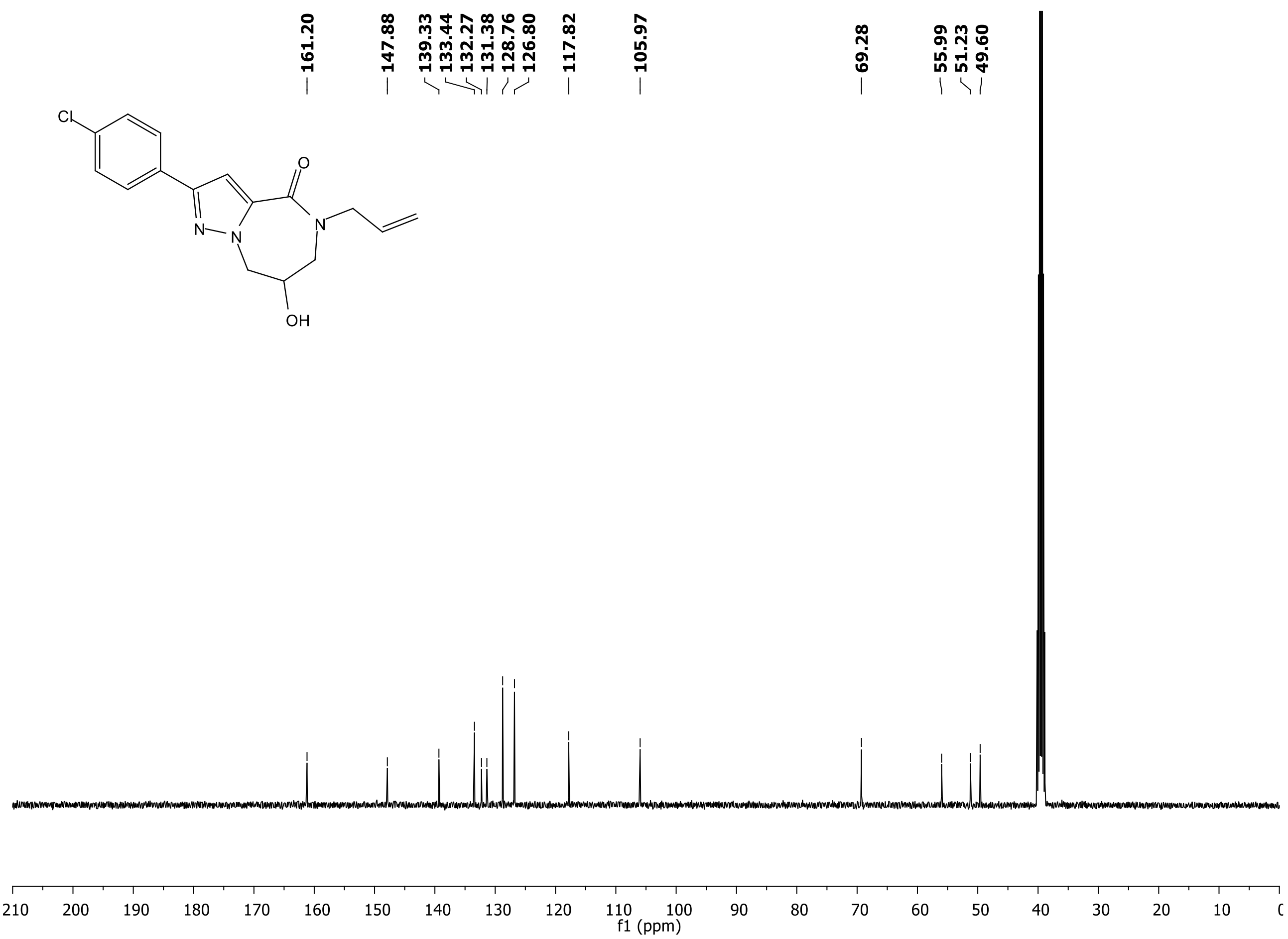

Figure S114. ^{13}C NMR spectrum (101 MHz, DMSO-d_6) of 5-allyl-2-(4-chlorophenyl)-7-hydroxy-5,6,7,8-tetrahydro-4*H*-pyrazolo[1,5-*a*][1,4]diazepin-4-one (**4p**).

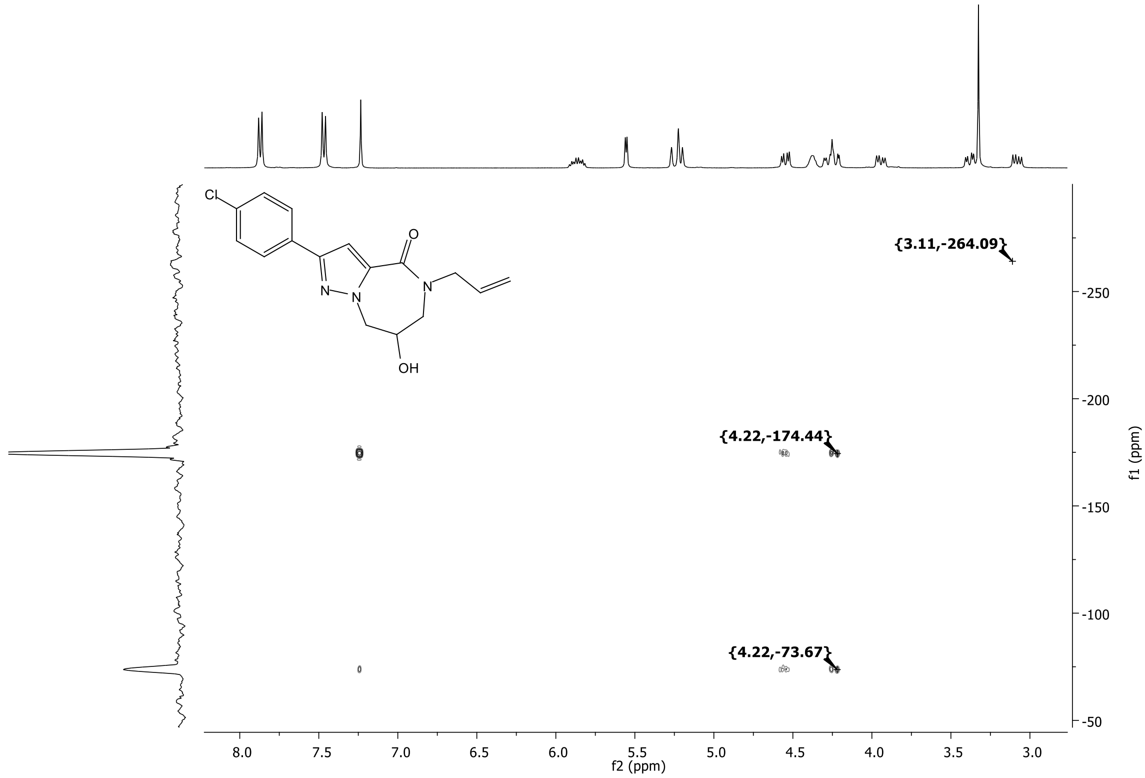

Figure S115. $^{1}H,^{15}N$-HMBC (40 MHz, DMSO-d_6) of 5-allyl-2-(4-chlorophenyl)-7-hydroxy-5,6,7,8-tetrahydro-4*H*-pyrazolo[1,5-*a*][1,4]diazepin-4-one (**4p**).

Compound Spectrum SmartFormula Report

Analysis Info

Analysis Name	D:\Data\KDD-127.d	Acquisition Date	4/6/2020 3:41:39 PM
Method	DirectInfusion_TuneLow_pos.m	Operator	hplc
Sample Name	KDD-127	Instrument	micrOTOF-Q III 8228888.20448
Comment	SB		

Acquisition Parameter

Source Type	ESI	Ion Polarity	Positive	Set Nebulizer	0.4 Bar
Focus	Not active	Set Capillary	4500 V	Set Dry Heater	180 °C
Scan Begin	50 m/z	Set End Plate Offset	-500 V	Set Dry Gas	4.0 l/min
Scan End	1000 m/z	Set Collision Cell RF	140.0 Vpp	Set Divert Valve	Waste

#	RT [min]	Area	Int. Type	I	S/N	Chromatogram	Max. m/z	FWHM [min]
n.a.	0.4	n.a.	Single spectrum	n.a.	n.a.	n.a.	226.9515	n.a.
n.a.	6.1	n.a.	Single spectrum	n.a.	n.a.	n.a.	340.0823	n.a.

+MS, 6.1min #363

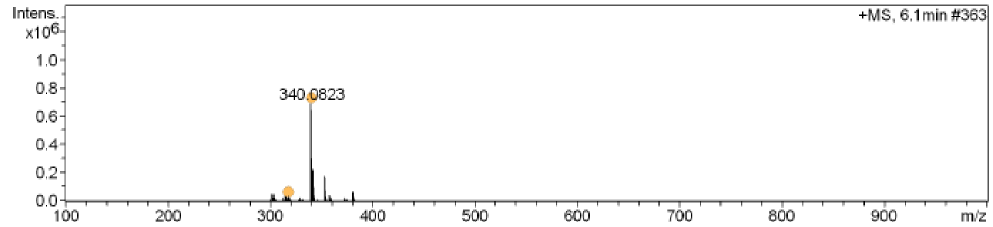

Meas. m/z	#	Ion Formula	m/z	err [ppm]	mSigma	# Sigma	Score	rdb	e⁻ Conf	N-Rule
318.0981	1	C16H17ClN3O2	318.1004	7.2	14.8	1	100.00	9.5	even	ok
340.0823	1	C16H16ClN3NaO2	340.0823	-0.0	4.6	1	100.00	9.5	even	ok

Figure S116. HRMS (ESI-TOF) spectrum of 5-allyl-2-(4-chlorophenyl)-7-hydroxy-5,6,7,8-tetrahydro-4*H*-pyrazolo[1,5-*a*][1,4]diazepin-4-one (**4p**).

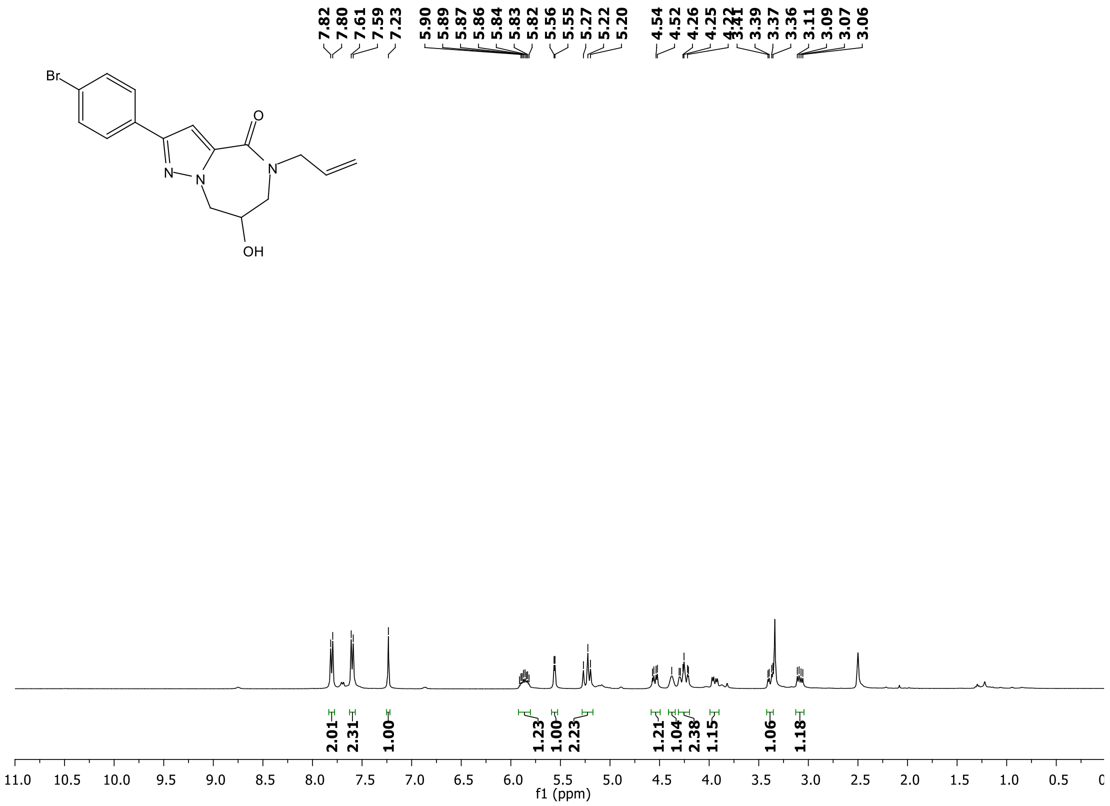

Figure S117. ^{1}H NMR spectrum (400 MHz, DMSO-d_6) of 5-allyl-2-(4-bromophenyl)-7-hydroxy-5,6,7,8-tetrahydro-4*H*-pyrazolo[1,5-*a*][1,4]diazepin-4-one (**4r**).

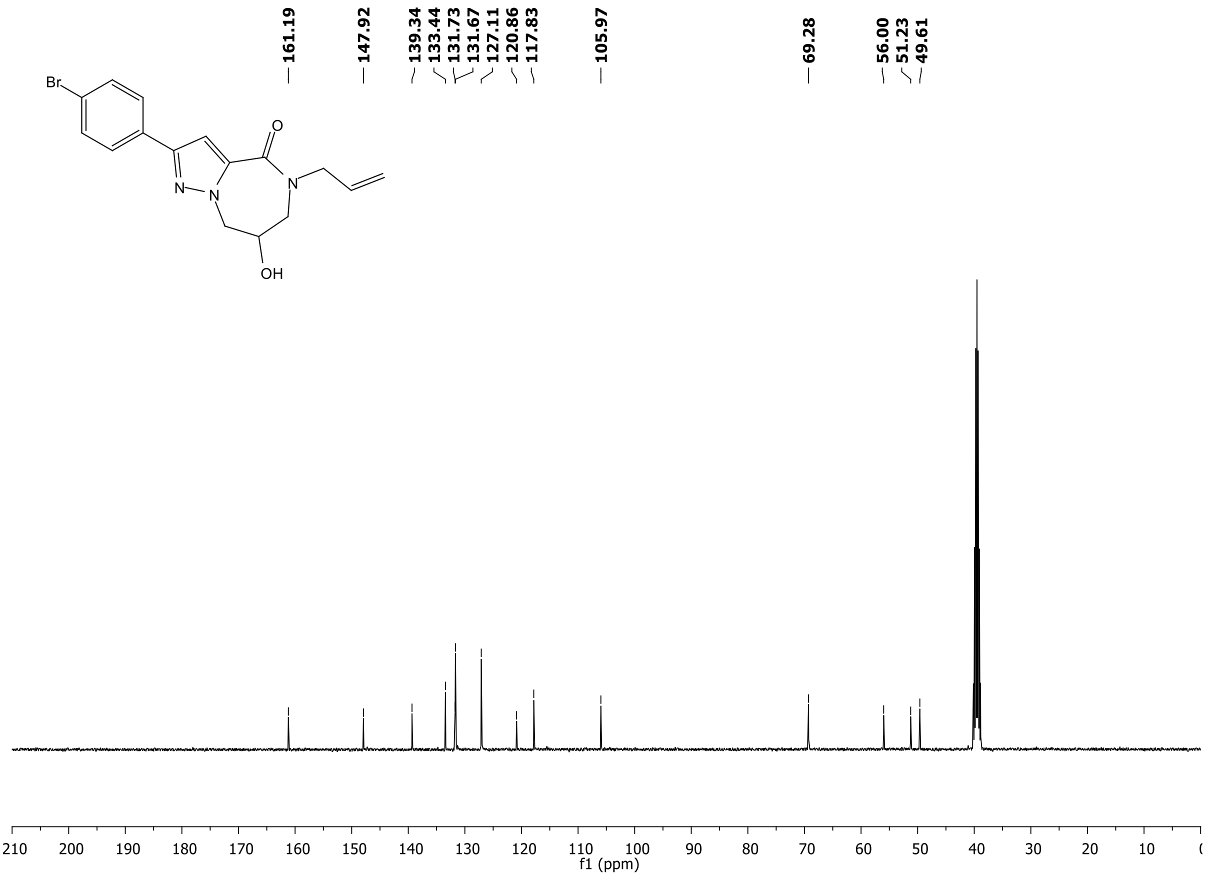

Figure S118. ^{13}C NMR spectrum (101 MHz, DMSO-d_6) of 5-allyl-2-(4-bromophenyl)-7-hydroxy-5,6,7,8-tetrahydro-4*H*-pyrazolo[1,5-*a*][1,4]diazepin-4-one (**4r**).

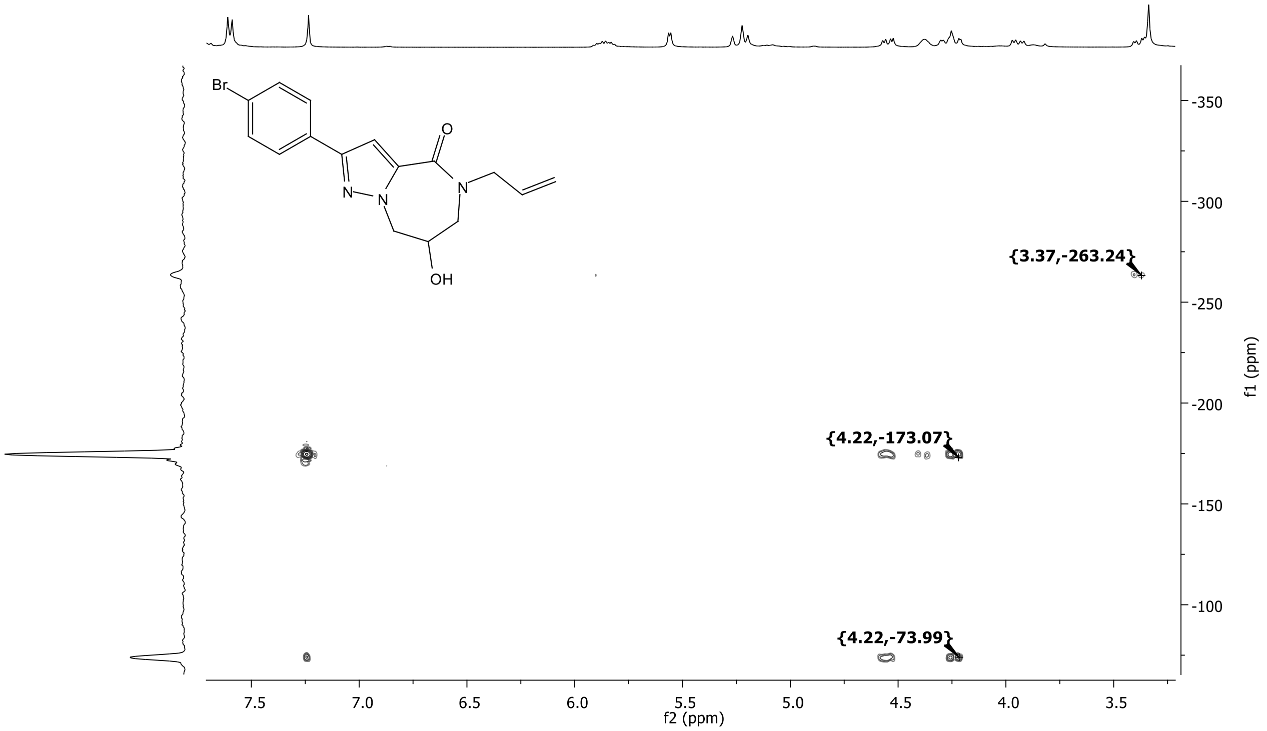

Figure S119. ^{1}H,^{15}N-HMBC (40 MHz, DMSO-d_6) of 5-allyl-2-(4-bromophenyl)-7-hydroxy-5,6,7,8-tetrahydro-4*H*-pyrazolo[1,5-*a*][1,4]diazepin-4-one (**4r**).

Compound Spectrum SmartFormula Report

Analysis Info

Analysis Name	D:\Data\KDD-113.d	Acquisition Date	4/6/2020 10:37:27 AM
Method	DirectInfusion_TuneLow_pos.m	Operator	hplc
Sample Name	KDD-113	Instrument	micrOTOF-Q III 8228888.20448
Comment	SB		

Acquisition Parameter

Source Type	ESI	Ion Polarity	Positive	Set Nebulizer	0.4 Bar
Focus	Not active	Set Capillary	4500 V	Set Dry Heater	180 °C
Scan Begin	50 m/z	Set End Plate Offset	-500 V	Set Dry Gas	4.0 l/min
Scan End	1000 m/z	Set Collision Cell RF	140.0 Vpp	Set Divert Valve	Waste

#	RT [min]	Area	Int. Type	I	S/N	Chromatogram	Max. m/z	FWHM [min]
n.a.	0.3	n.a.	Single spectrum	n.a.	n.a.	n.a.	226.9517	n.a.
n.a.	6.8	n.a.	Single spectrum	n.a.	n.a.	n.a.	384.0318	n.a.

+MS, 6.8min #406

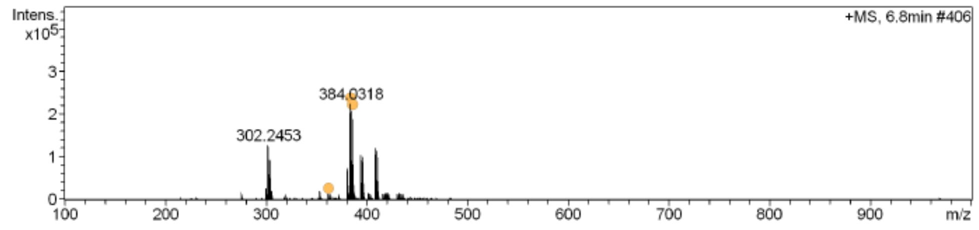

Meas. m/z	#	Ion Formula	m/z	err [ppm]	mSigma	# Sigma	Score	rdb	e⁻ Conf	N-Rule
362.0494	1	C16H17BrN3O2	362.0499	-1.4	21.5	1	100.00	9.5	even	ok
384.0318	1	C16H16BrN3NaO2	384.0318	-0.1	30.6	1	100.00	9.5	even	ok
386.0303	1	C16H16BrN3NaO2	384.0318	0.9	30.6	1	100.00	9.5	even	ok

Figure S120. HRMS (ESI-TOF) spectrum of 5-allyl-2-(4-bromophenyl)-7-hydroxy-5,6,7,8-tetrahydro-4*H*-pyrazolo[1,5-*a*][1,4]diazepin-4-one (**4r**).

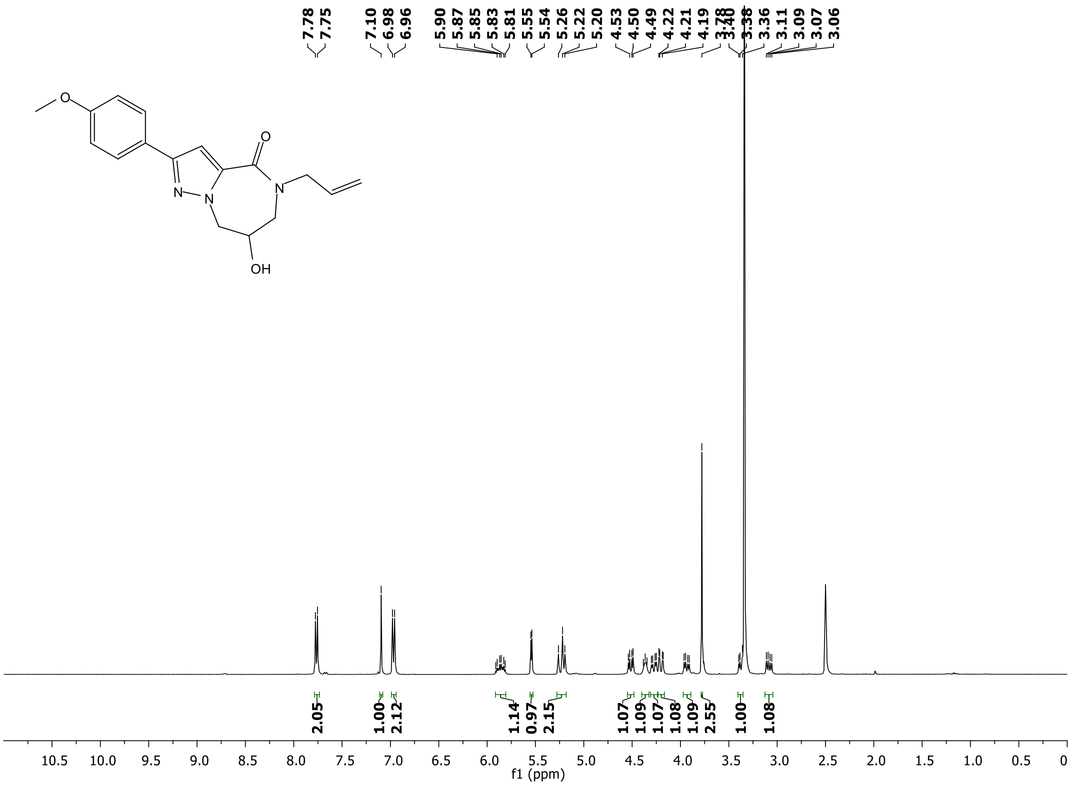

Figure S121. ^{1}H NMR spectrum (400 MHz, DMSO-d_6) of 5-allyl-7-hydroxy-2-(4-methoxyphenyl)-5,6,7,8-tetrahydro-4*H*-pyrazolo[1,5-*a*][1,4]diazepin-4-one (**4s**).

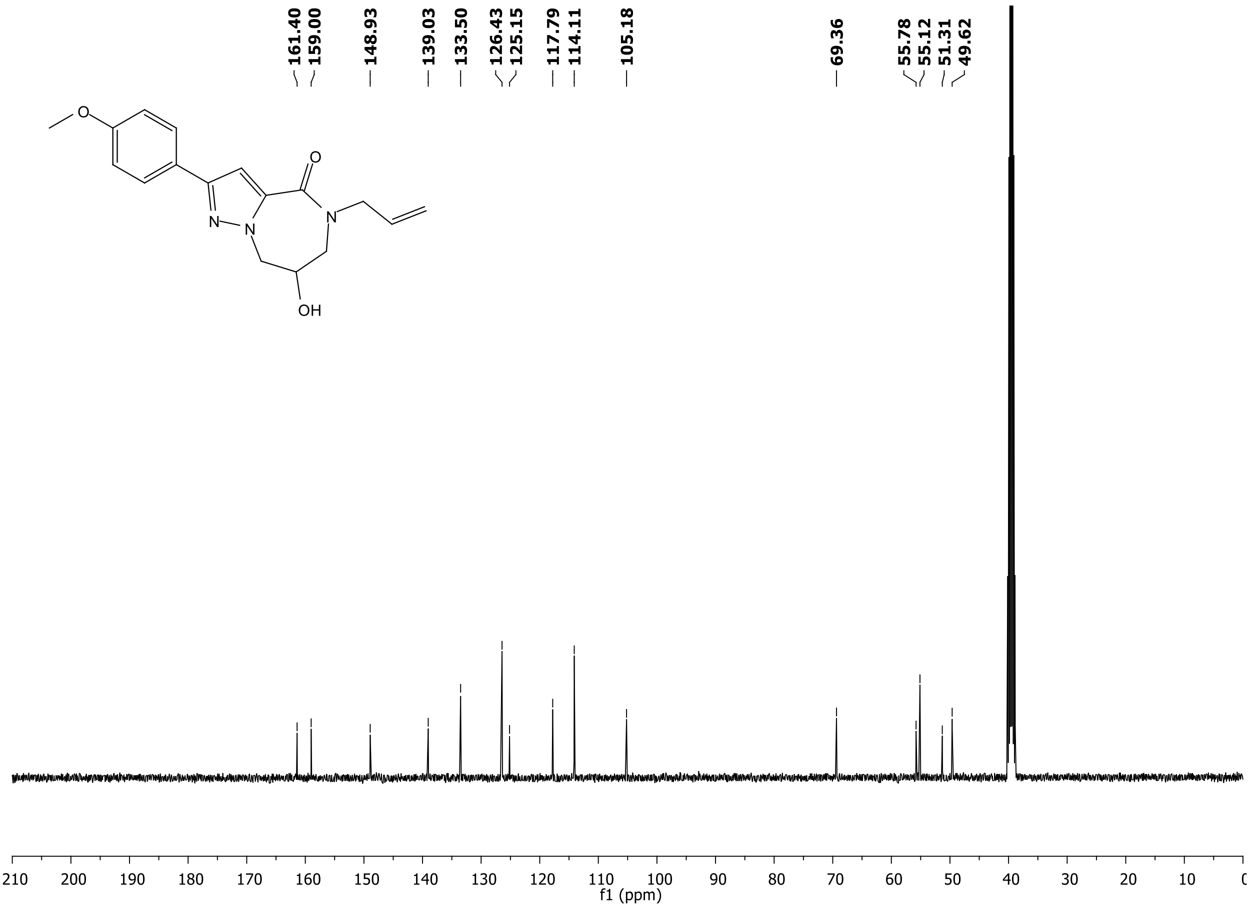

Figure S122. ^{13}C NMR spectrum (101 MHz, DMSO-d_6) of 5-allyl-7-hydroxy-2-(4-methoxyphenyl)-5,6,7,8-tetrahydro-4*H*-pyrazolo[1,5-*a*][1,4]diazepin-4-one (**4s**).

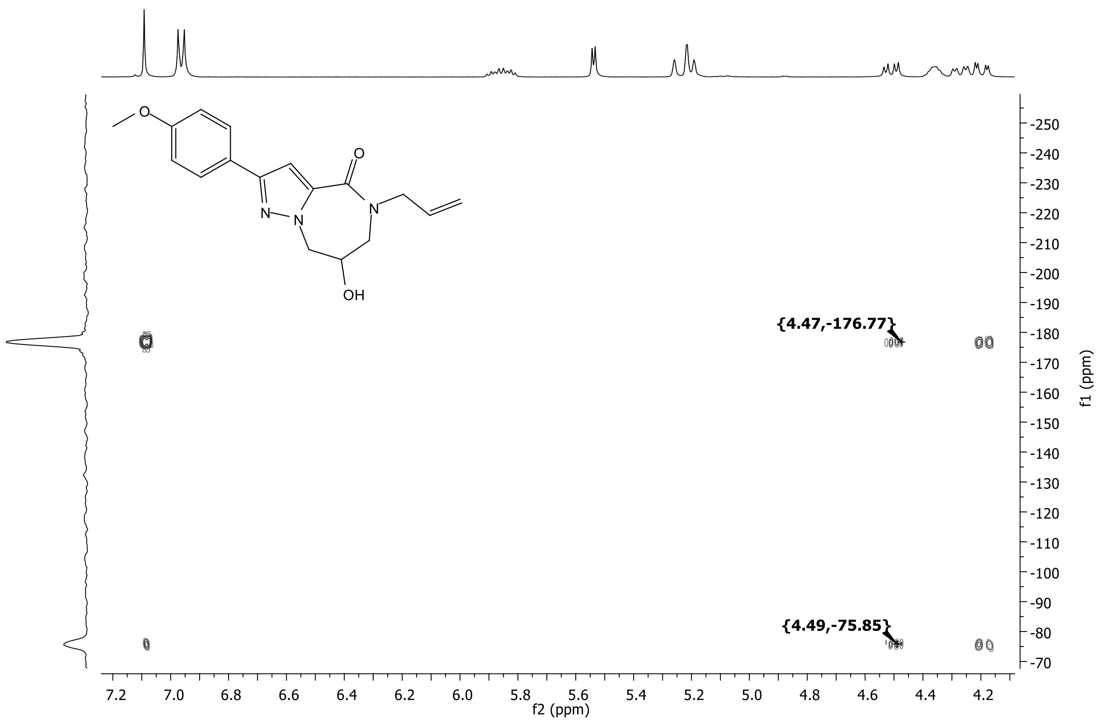

Figure S123. ^{1}H,^{15}N-HMBC (40 MHz, DMSO-d_6) of 5-allyl-7-hydroxy-2-(4-methoxyphenyl)-5,6,7,8-tetrahydro-4*H*-pyrazolo[1,5-*a*][1,4]diazepin-4-one (**4s**).

Compound Spectrum SmartFormula Report

Analysis Info

Analysis Name	D:\Data\KDD-180-1.d	Acquisition Date	8/10/2021 1:59:24 PM
Method	DirectInfusion_TuneLow_pos.m	Operator	hplc
Sample Name	KDD-180-1	Instrument	micrOTOF-Q III 8228888.20448
Comment	SB		

Acquisition Parameter

Source Type	ESI	Ion Polarity	Positive	Set Nebulizer	0.4 Bar
Focus	Not active	Set Capillary	4500 V	Set Dry Heater	180 °C
Scan Begin	50 m/z	Set End Plate Offset	-500 V	Set Dry Gas	4.0 l/min
Scan End	1000 m/z	Set Collision Cell RF	140.0 Vpp	Set Divert Valve	Waste

#	RT [min]	Area	Int. Type	I	S/N	Chromatogram	Max. m/z	FWHM [min]
n.a.	0.6	n.a.	Single spectrum	n.a.	n.a.	n.a.	226.9519	n.a.
n.a.	4.8	n.a.	Single spectrum	n.a.	n.a.	n.a.	336.1319	n.a.

+MS, 4.8min #290

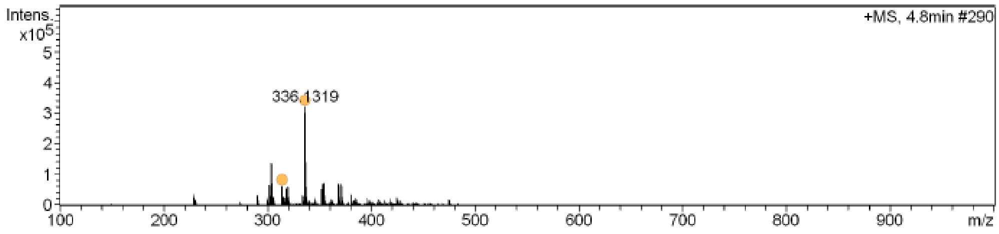

Meas. m/z	#	Ion Formula	m/z	err [ppm]	mSigma	# Sigma	Score	rdb	e⁻ Conf	N-Rule
314.1495	1	C17H20N3O3	314.1499	-1.2	15.2	1	100.00	9.5	even	ok
336.1319	1	C17H19N3NaO3	336.1319	0.0	3.6	1	100.00	9.5	even	ok

Figure S124. HRMS (ESI-TOF) spectrum of 5-allyl-7-hydroxy-2-(4-methoxyphenyl)-5,6,7,8-tetrahydro-4*H*-pyrazolo[1,5-*a*][1,4]diazepin-4-one (**4s**).

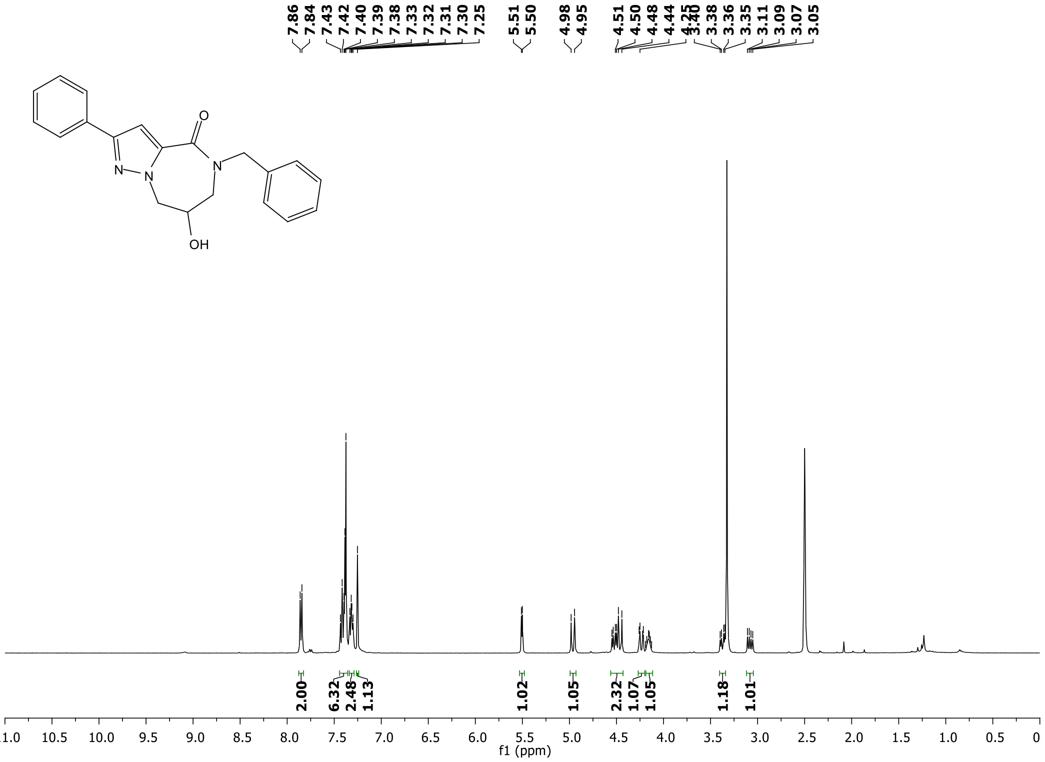

Figure S125. ^{1}H NMR spectrum (400 MHz, DMSO-d_6) of 5-benzyl-7-hydroxy-2-phenyl-5,6,7,8-tetrahydro-4*H*-pyrazolo[1,5-*a*][1,4]diazepin-4-one (**4t**).

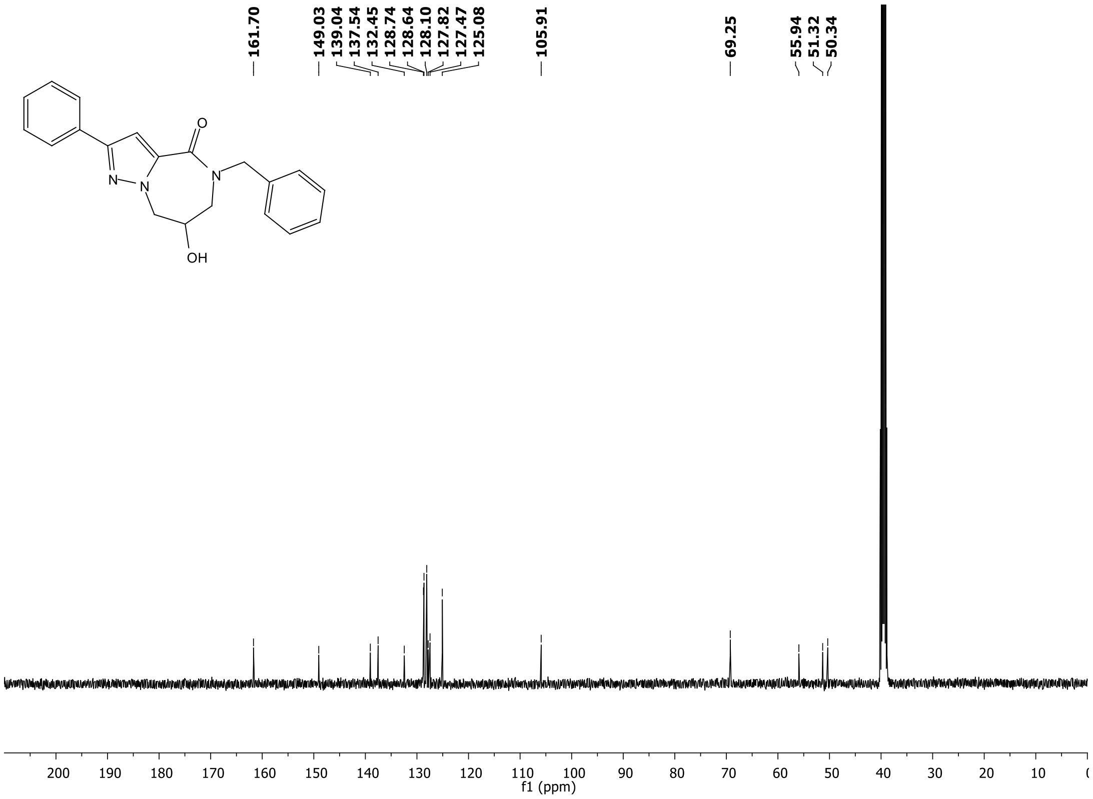

Figure S126. ^{13}C NMR spectrum (101 MHz, DMSO-d_6) of 5-benzyl-7-hydroxy-2-phenyl-5,6,7,8-tetrahydro-4*H*-pyrazolo[1,5-*a*][1,4]diazepin-4-one (**4t**).

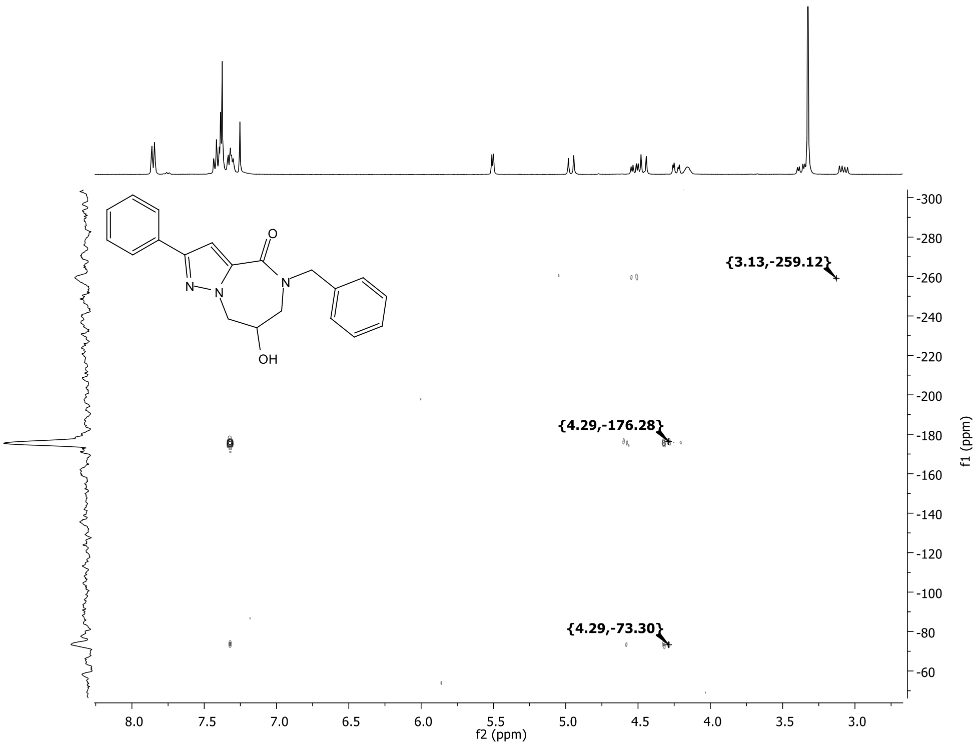

Figure S127. $^{1}H,^{15}N$-HMBC (40 MHz, DMSO-d_6) of 5-benzyl-7-hydroxy-2-phenyl-5,6,7,8-tetrahydro-4*H*-pyrazolo[1,5-*a*][1,4]diazepin-4-one (**4t**).

Compound Spectrum SmartFormula Report

Analysis Info

Analysis Name	D:\Data\KDD-071.d	Acquisition Date	3/30/2020 5:33:03 PM
Method	DirectInfusion_TuneLow_pos.m	Operator	hplc
Sample Name	KDD-071	Instrument	micrOTOF-Q III 8228888.20448
Comment	SB		

Acquisition Parameter

Source Type	ESI	Ion Polarity	Positive	Set Nebulizer	0.4 Bar
Focus	Not active	Set Capillary	4500 V	Set Dry Heater	180 °C
Scan Begin	50 m/z	Set End Plate Offset	-500 V	Set Dry Gas	4.0 l/min
Scan End	1000 m/z	Set Collision Cell RF	140.0 Vpp	Set Divert Valve	Waste

#	RT [min]	Area	Int. Type	I	S/N	Chromatogram	Max. m/z	FWHM [min]
n.a.	1.2	n.a.	Single spectrum	n.a.	n.a.	n.a.	226.9517	n.a.
n.a.	5.1	n.a.	Single spectrum	n.a.	n.a.	n.a.	356.1370	n.a.

+MS, 5.1min #305

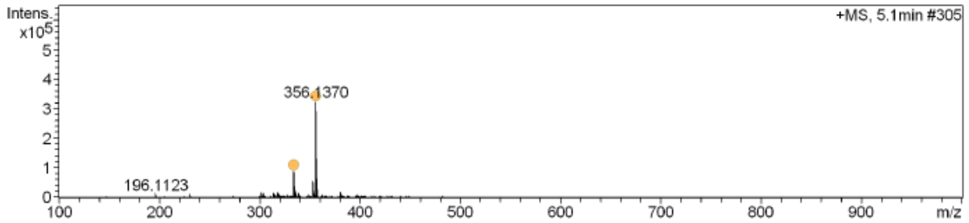

Meas. m/z	#	Ion Formula	m/z	err [ppm]	mSigma	# Sigma	Score	rdb	e⁻ Conf	N-Rule
334.1551	1	C20H20N3O2	334.1550	-0.1	62.4	1	100.00	12.5	even	ok
356.1370	1	C20H19N3NaO2	356.1369	-0.0	3.2	1	100.00	12.5	even	ok

Figure S128. HRMS (ESI-TOF) spectrum of 5-benzyl-7-hydroxy-2-phenyl-5,6,7,8-tetrahydro-4*H*-pyrazolo[1,5-*a*][1,4]diazepin-4-one (**4t**).

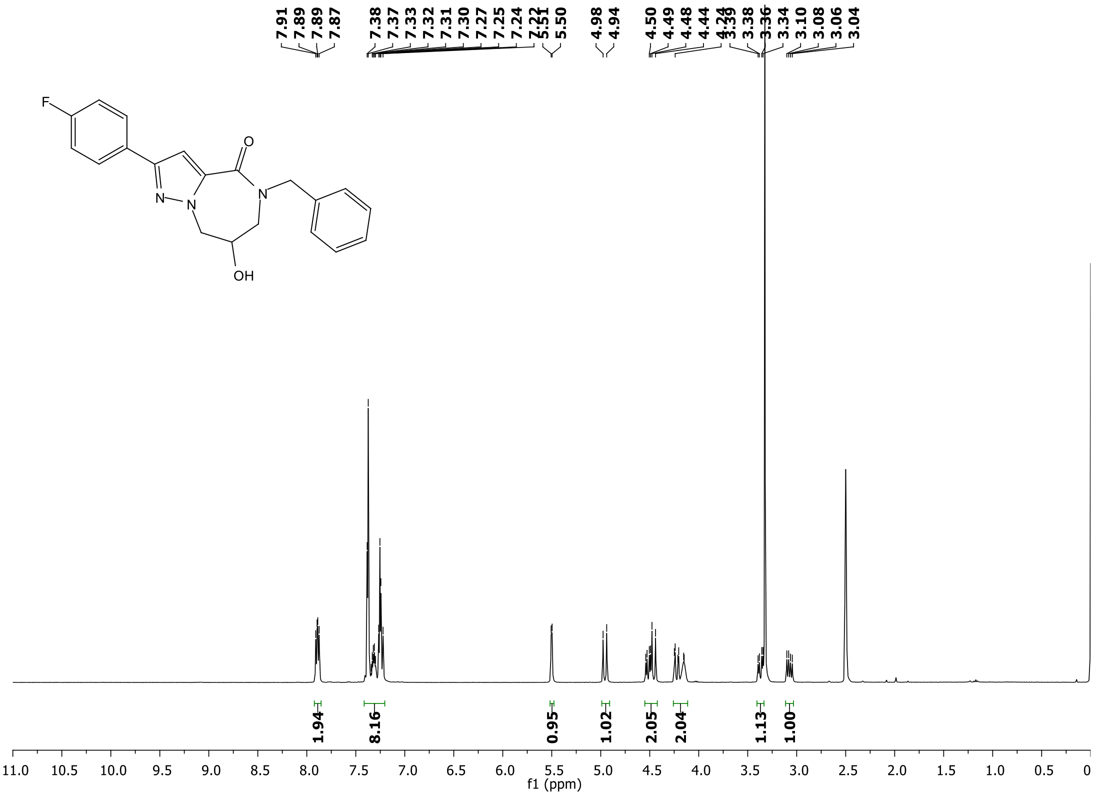

Figure S129. ^{1}H NMR spectrum (400 MHz, DMSO-d_6) of 5-benzyl-2-(4-fluorophenyl)-7-hydroxy-5,6,7,8-tetrahydro-4*H*-pyrazolo[1,5-*a*][1,4]diazepin-4-one (**4u**).

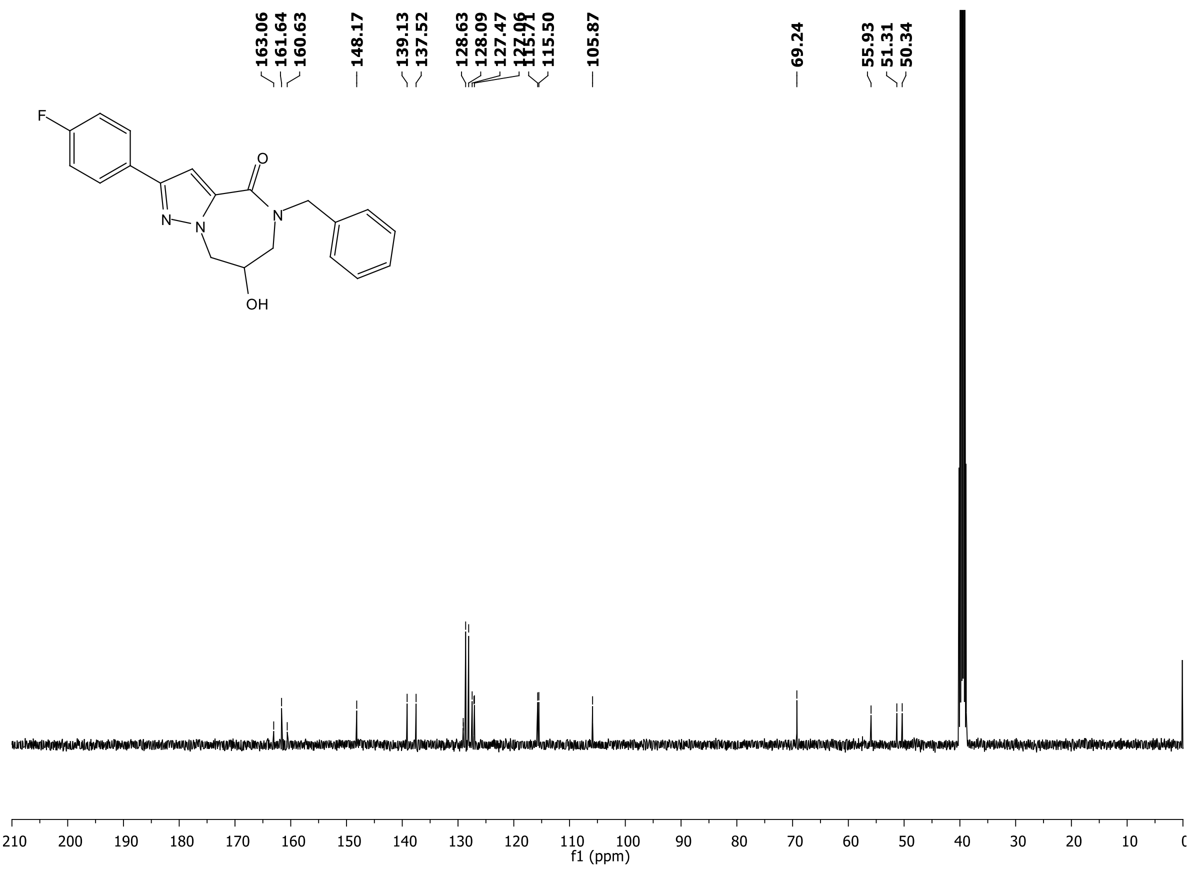

Figure S130. ^{13}C NMR spectrum (101 MHz, DMSO-d_6) of 5-benzyl-2-(4-fluorophenyl)-7-hydroxy-5,6,7,8-tetrahydro-4*H*-pyrazolo[1,5-*a*][1,4]diazepin-4-one (**4u**).

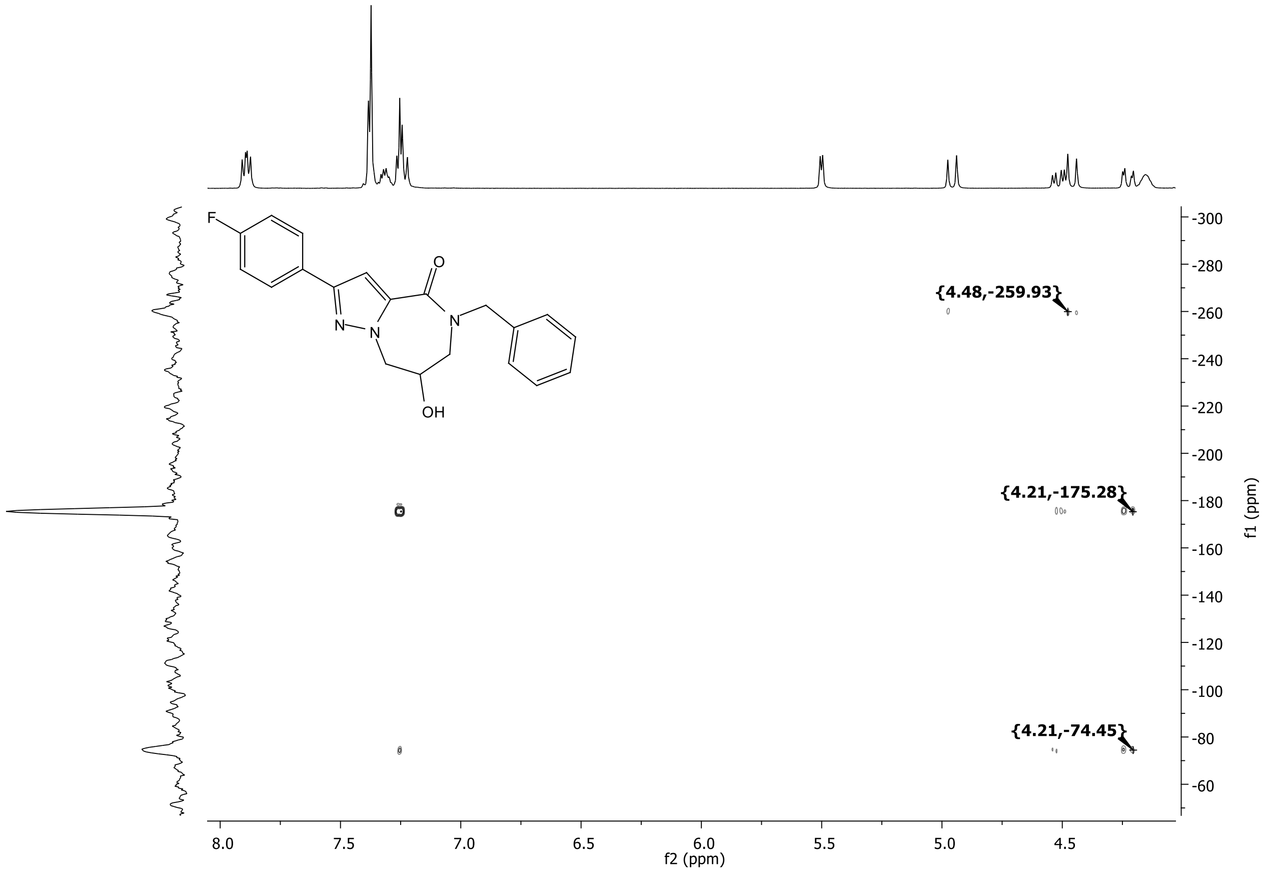

Figure S131. ^{1}H,^{15}N-HMBC (40 MHz, DMSO-d_6) of 5-benzyl-2-(4-fluorophenyl)-7-hydroxy-5,6,7,8-tetrahydro-4*H*-pyrazolo[1,5-*a*][1,4]diazepin-4-one (**4u**).

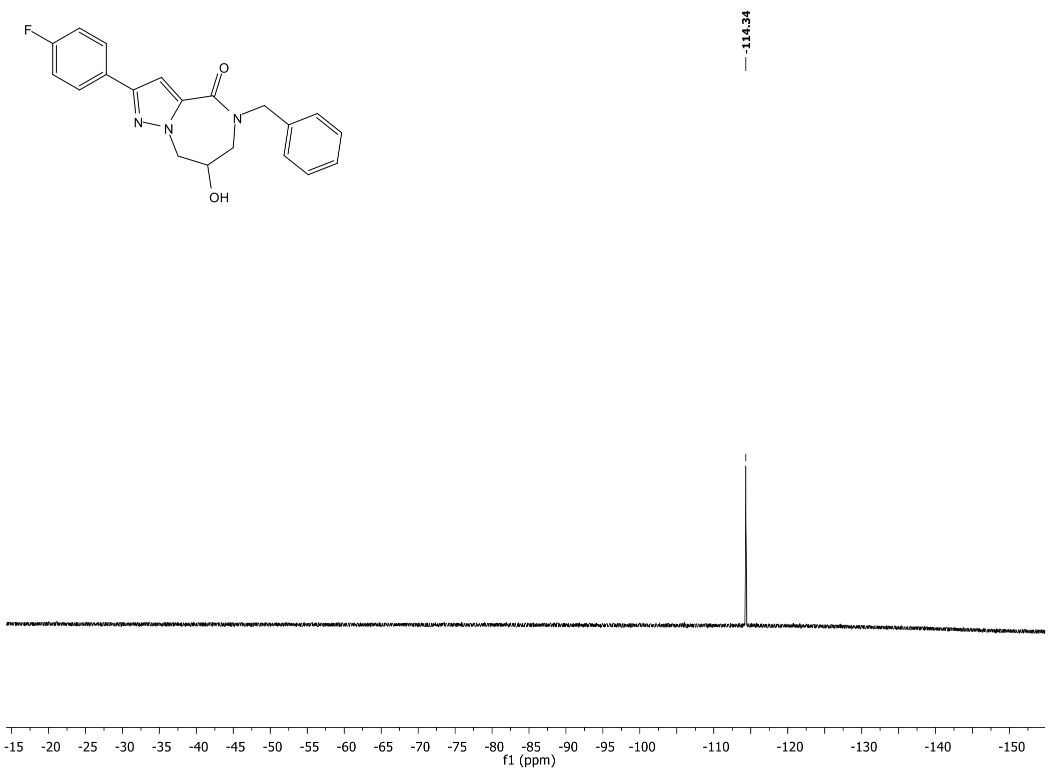

Figure S132. ^{19}F NMR spectrum (376 MHz, DMSO-d_6) of 5-benzyl-2-(4-fluorophenyl)-7-hydroxy-5,6,7,8-tetrahydro-4*H*-pyrazolo[1,5-*a*][1,4]diazepin-4-one (**4u**).

Compound Spectrum SmartFormula Report

Analysis Info

Analysis Name	D:\Data\KDD-130.d	Acquisition Date	4/7/2020 2:42:38 PM
Method	DirectInfusion_TuneLow_pos.m	Operator	hplc
Sample Name	KDD-130	Instrument	micrOTOF-Q III 8228888.20448
Comment	SB		

Acquisition Parameter

Source Type	ESI	Ion Polarity	Positive	Set Nebulizer	0.4 Bar
Focus	Not active	Set Capillary	4500 V	Set Dry Heater	180 °C
Scan Begin	50 m/z	Set End Plate Offset	-500 V	Set Dry Gas	4.0 l/min
Scan End	1000 m/z	Set Collision Cell RF	140.0 Vpp	Set Divert Valve	Waste

#	RT [min]	Area	Int. Type	I	S/N	Chromatogram	Max. m/z	FWHM [min]
n.a.	0.3	n.a.	Single spectrum	n.a.	n.a.	n.a.	226.9516	n.a.
n.a.	4.3	n.a.	Single spectrum	n.a.	n.a.	n.a.	374.1275	n.a.

+MS, 4.3min #260

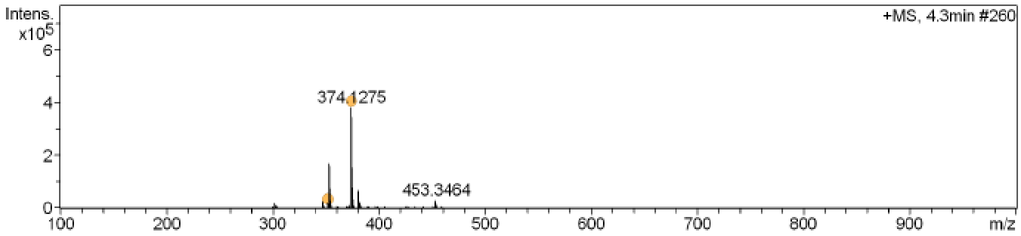

Meas. m/z	#	Ion Formula	m/z	err [ppm]	mSigma	# Sigma	Score	rdb	e⁻ Conf	N-Rule
352.1457	1	C20H19FN3O2	352.1456	0.4	51.3	1	100.00	12.5	even	ok
374.1275	1	C20H18FN3NaO2	374.1275	0.0	2.0	1	100.00	12.5	even	ok

Figure S133. HRMS (ESI-TOF) spectrum of 5-benzyl-2-(4-fluorophenyl)-7-hydroxy-5,6,7,8-tetrahydro-4*H*-pyrazolo[1,5-*a*][1,4]diazepin-4-one (**4u**).

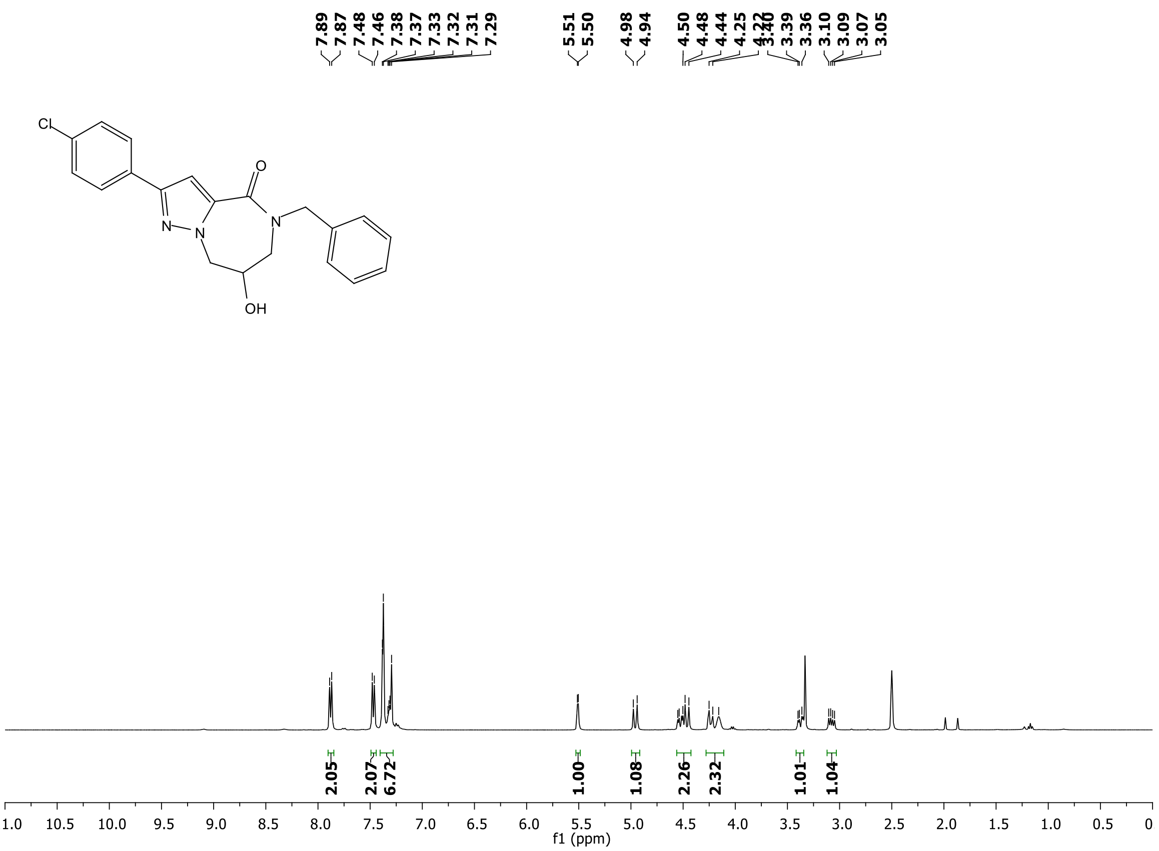

Figure S134. ^{1}H NMR spectrum (400 MHz, DMSO-d_6) of 5-benzyl-2-(4-chlorophenyl)-7-hydroxy-5,6,7,8-tetrahydro-4*H*-pyrazolo[1,5-*a*][1,4]diazepin-4-one (**4v**).

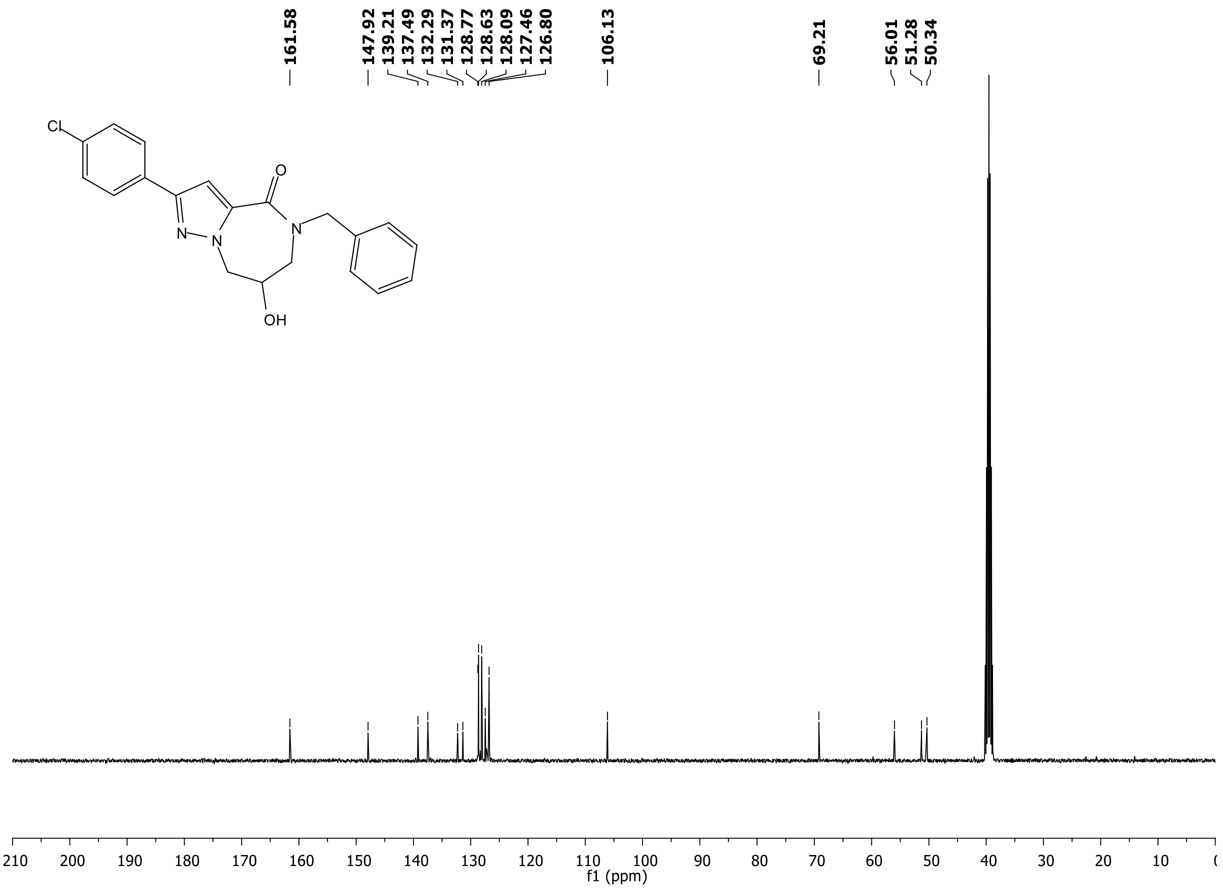

Figure S135. ^{13}C NMR spectrum (101 MHz, DMSO-d_6) of 5-benzyl-2-(4-chlorophenyl)-7-hydroxy-5,6,7,8-tetrahydro-4*H*-pyrazolo[1,5-*a*][1,4]diazepin-4-one (**4v**).

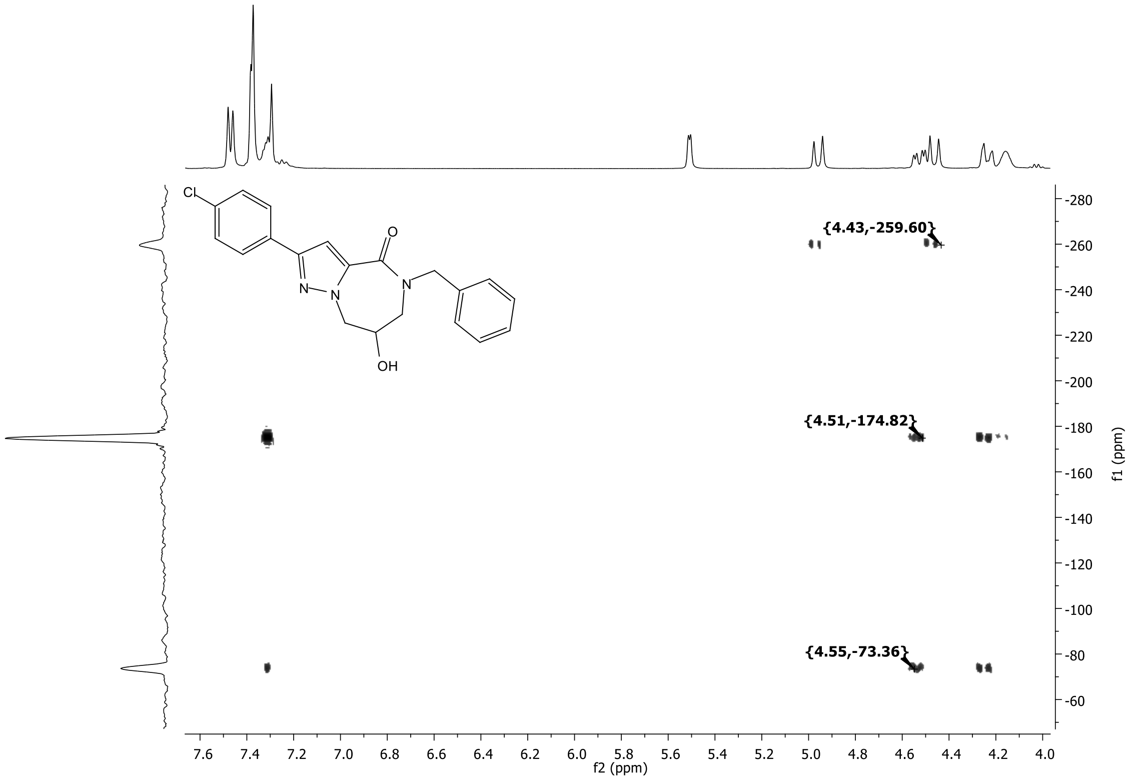

Figure S136. $^{1}H,^{15}N$-HMBC (40 MHz, DMSO-d_6) of 5-benzyl-2-(4-chlorophenyl)-7-hydroxy-5,6,7,8-tetrahydro-4*H*-pyrazolo[1,5-*a*][1,4]diazepin-4-one (**4v**).

Compound Spectrum SmartFormula Report

Analysis Info

Analysis Name D:\Data\KDD-129.d
Method DirectInfusion_TuneLow_pos.m
Sample Name KDD-129
Comment SB

Acquisition Date 4/6/2020 4:10:58 PM
Operator hplc
Instrument micrOTOF-Q III 8228888.20448

Acquisition Parameter

Source Type	ESI	Ion Polarity	Positive	Set Nebulizer	0.4 Bar
Focus	Not active	Set Capillary	4500 V	Set Dry Heater	180 °C
Scan Begin	50 m/z	Set End Plate Offset	-500 V	Set Dry Gas	4.0 l/min
Scan End	1000 m/z	Set Collision Cell RF	140.0 Vpp	Set Divert Valve	Waste

#	RT [min]	Area	Int. Type	I	S/N	Chromatogram	Max. m/z	FWHM [min]
n.a.	0.3	n.a.	Single spectrum	n.a.	n.a.	n.a.	226.9515	n.a.
n.a.	5.7	n.a.	Single spectrum	n.a.	n.a.	n.a.	390.0980	n.a.

+MS, 5.7min #344

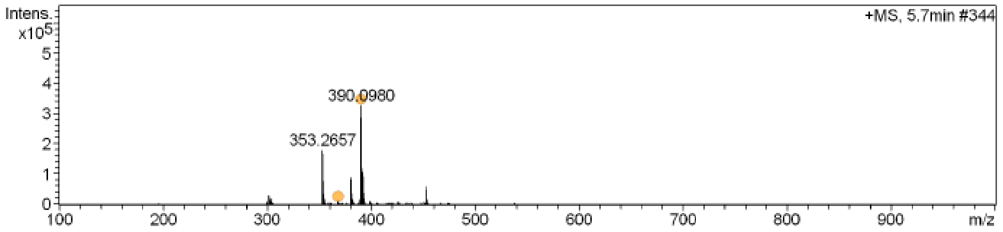

Meas. m/z	#	Ion Formula	m/z	err [ppm]	mSigma	# Sigma	Score	rdb	e⁻ Conf	N-Rule
368.1166	1	C20H19ClN3O2	368.1160	-1.5	30.3	1	100.00	12.5	even	ok
390.0980	1	C20H18ClN3NaO2	390.0980	-0.0	6.7	1	100.00	12.5	even	ok

Figure S137. HRMS (ESI-TOF) spectrum of 5-bnzyl-2-(4-chlorophenyl)-7-hydroxy-5,6,7,8-tetrahydro-4*H*-pyrazolo[1,5-*a*][1,4]diazepin-4-one (**4v**).

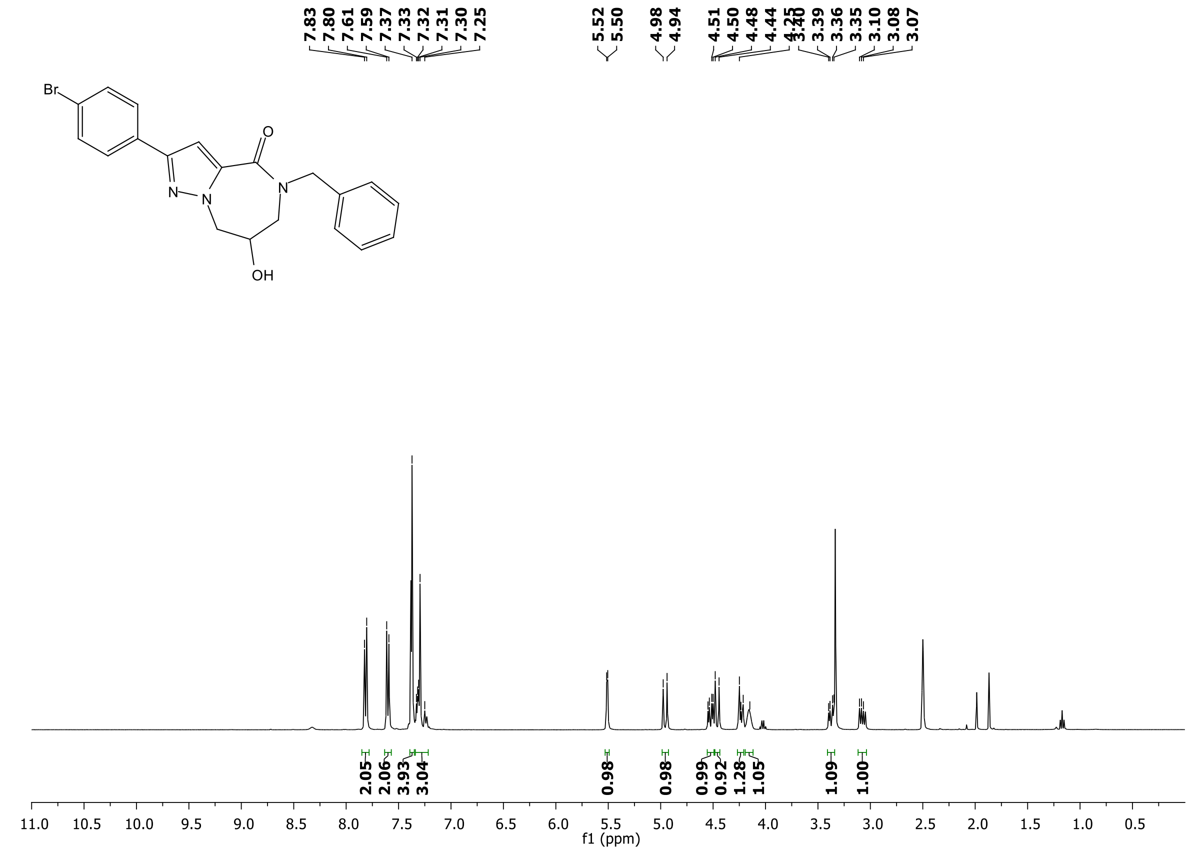

Figure S138. ^{1}H NMR spectrum (400 MHz, DMSO-d_6) of 5-benzyl-2-(4-bromophenyl)-7-hydroxy-5,6,7,8-tetrahydro-4*H*-pyrazolo[1,5-*a*][1,4]diazepin-4-one (**4w**).

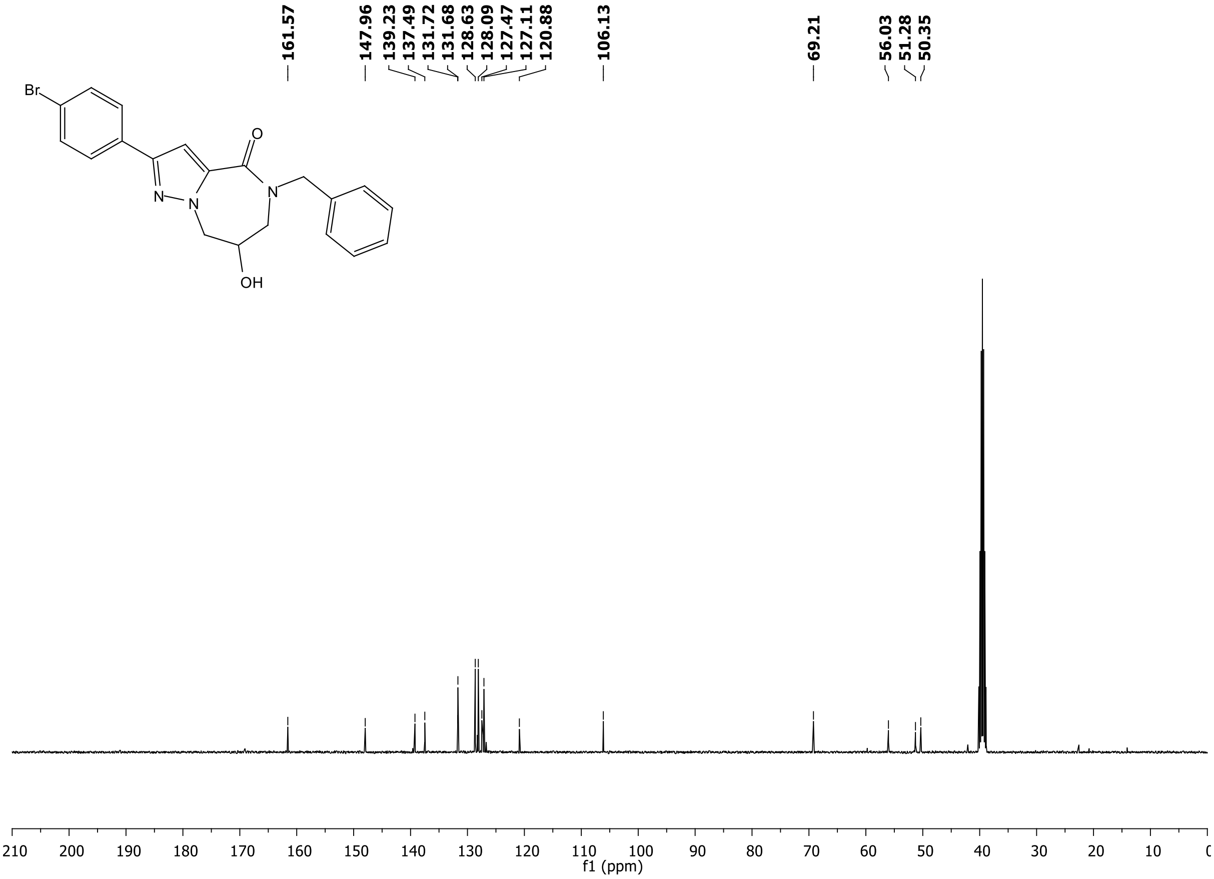

Figure S139. ^{13}C NMR spectrum (101 MHz, DMSO-d_6) of 5-benzyl-2-(4-bromophenyl)-7-hydroxy-5,6,7,8-tetrahydro-4*H*-pyrazolo[1,5-*a*][1,4]diazepin-4-one (**4w**).

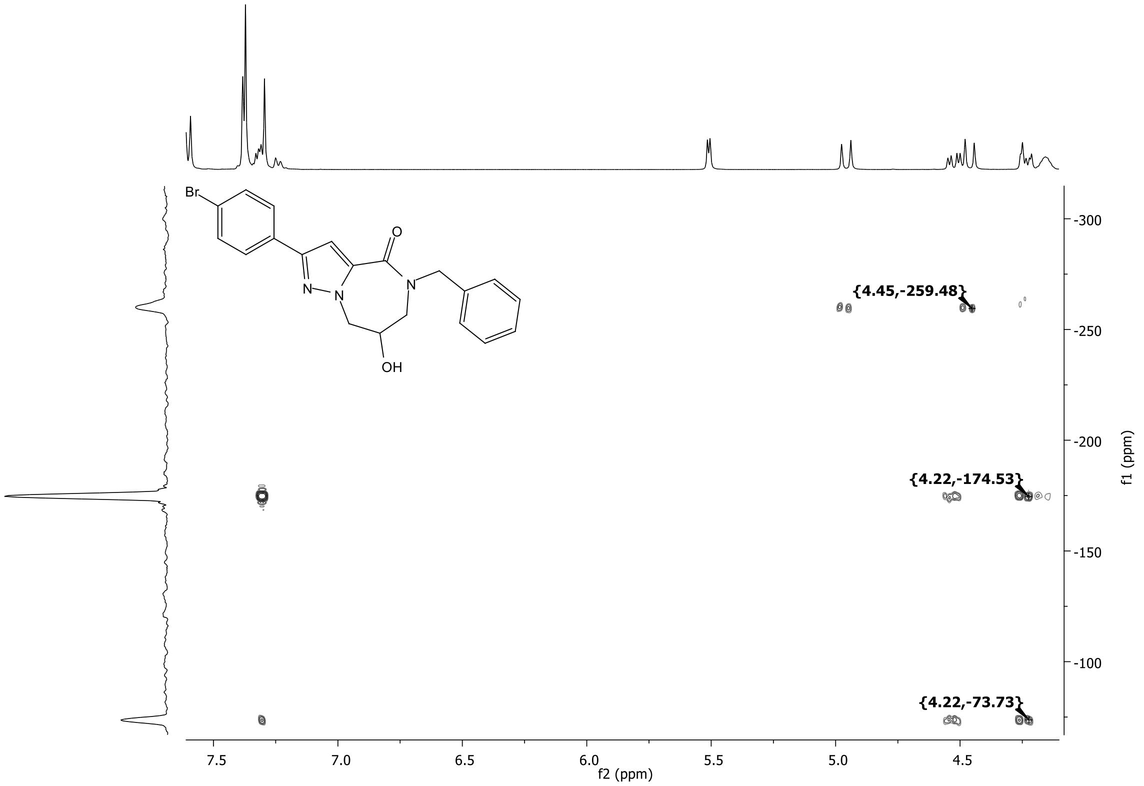

Figure S140. ^{1}H,^{15}N-HMBC (40 MHz, DMSO-d_6) of 5-benzyl-2-(4-bromophenyl)-7-hydroxy-5,6,7,8-tetrahydro-4*H*-pyrazolo[1,5-*a*][1,4]diazepin-4-one (**4w**).

Compound Spectrum SmartFormula Report

Analysis Info

Analysis Name	D:\Data\KDD-115.d	Acquisition Date	4/6/2020 11:01:46 AM
Method	DirectInfusion_TuneLow_pos.m	Operator	hplc
Sample Name	KDD-115	Instrument	micrOTOF-Q III 8228888.20448
Comment	SB		

Acquisition Parameter

Source Type	ESI	Ion Polarity	Positive	Set Nebulizer	0.4 Bar
Focus	Not active	Set Capillary	4500 V	Set Dry Heater	180 °C
Scan Begin	50 m/z	Set End Plate Offset	-500 V	Set Dry Gas	4.0 l/min
Scan End	1000 m/z	Set Collision Cell RF	140.0 Vpp	Set Divert Valve	Waste

#	RT [min]	Area	Int. Type	I	S/N	Chromatogram	Max. m/z	FWHM [min]
n.a.	1.8	n.a.	Single spectrum	n.a.	n.a.	n.a.	226.9519	n.a.
n.a.	6.4	n.a.	Single spectrum	n.a.	n.a.	n.a.	304.2597	n.a.

+MS, 6.4min #383

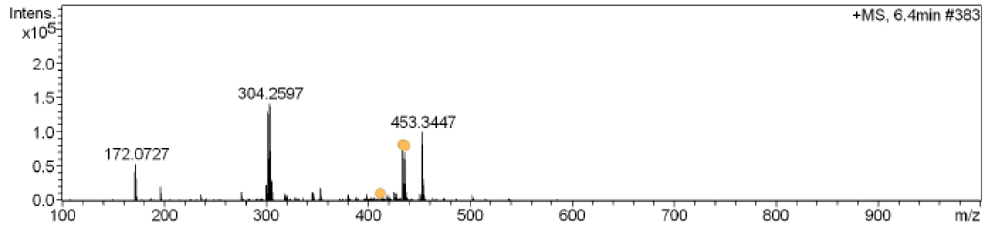

Meas. m/z	#	Ion Formula	m/z	err [ppm]	mSigma	# Sigma	Score	rdb	e⁻ Conf	N-Rule
412.0647	1	C20H19BrN3O2	412.0655	-1.9	471.4	1	100.00	12.5	even	ok
434.0475	1	C20H18BrN3NaO2	434.0475	-0.1	12.1	1	100.00	12.5	even	ok
436.0445	1	C20H18BrN3NaO2	434.0475	-2.7	12.1	1	100.00	12.5	even	ok

Figure S141. HRMS (ESI-TOF) spectrum of 5-benzyl-2-(4-bromophenyl)-7-hydroxy-5,6,7,8-tetrahydro-4*H*-pyrazolo[1,5-*a*][1,4]diazepin-4-one (**4w**).

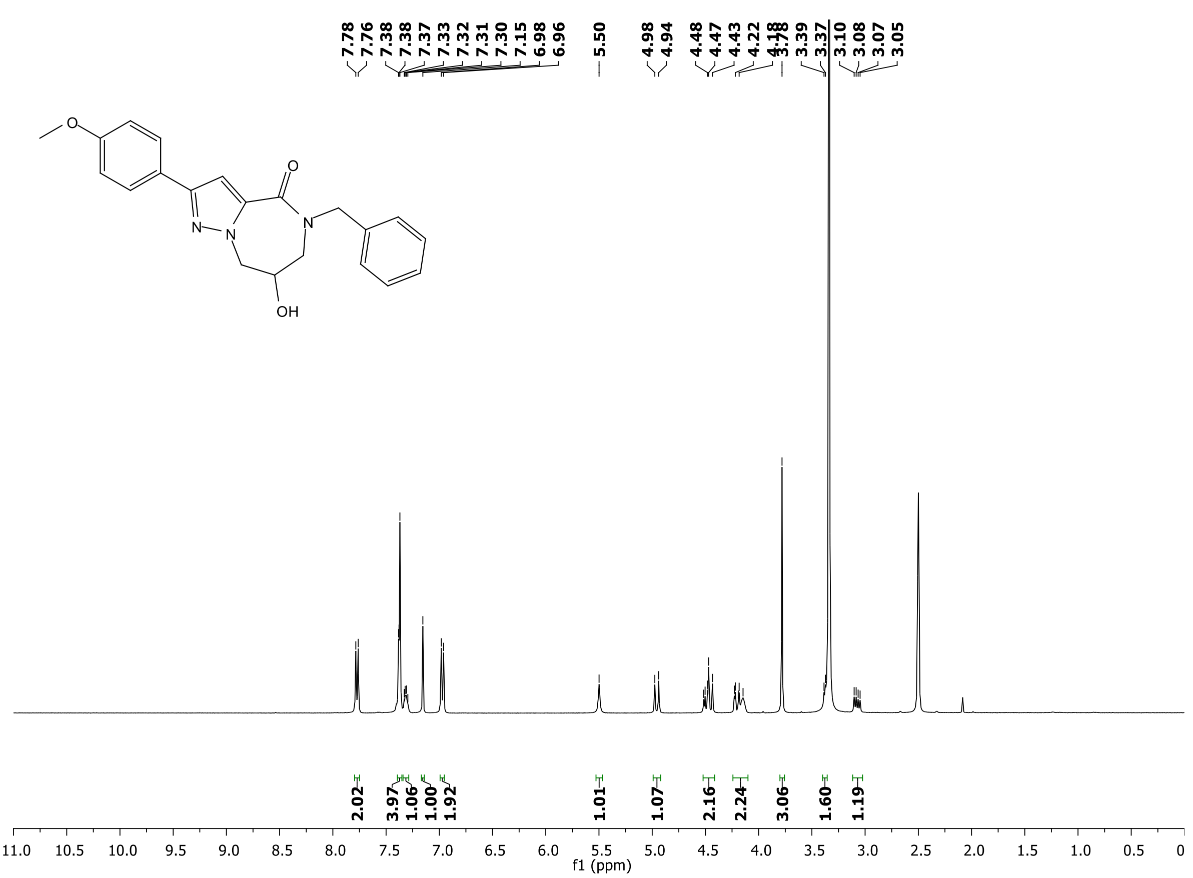

Figure S142. ^{1}H NMR spectrum (400 MHz, DMSO-d_6) of 5-benzyl-7-hydroxy-2-(4-methoxyphenyl)-5,6,7,8-tetrahydro-4*H*-pyrazolo[1,5-*a*][1,4]diazepin-4-one (**4x**).

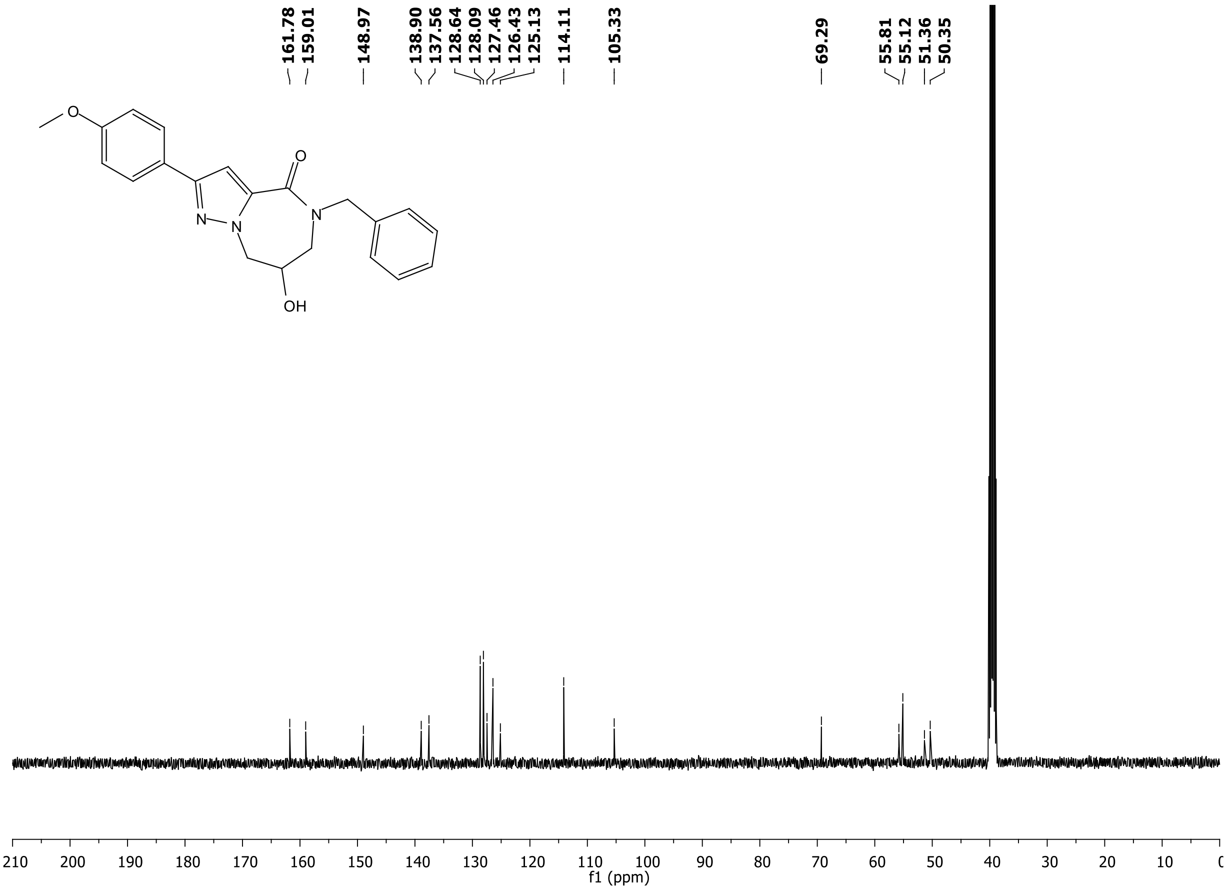

Figure S143. ^{13}C NMR spectrum (101 MHz, DMSO-d_6) of 5-benzyl-7-hydroxy-2-(4-methoxyphenyl)-5,6,7,8-tetrahydro-4*H*-pyrazolo[1,5-*a*][1,4]diazepin-4-one (**4x**).

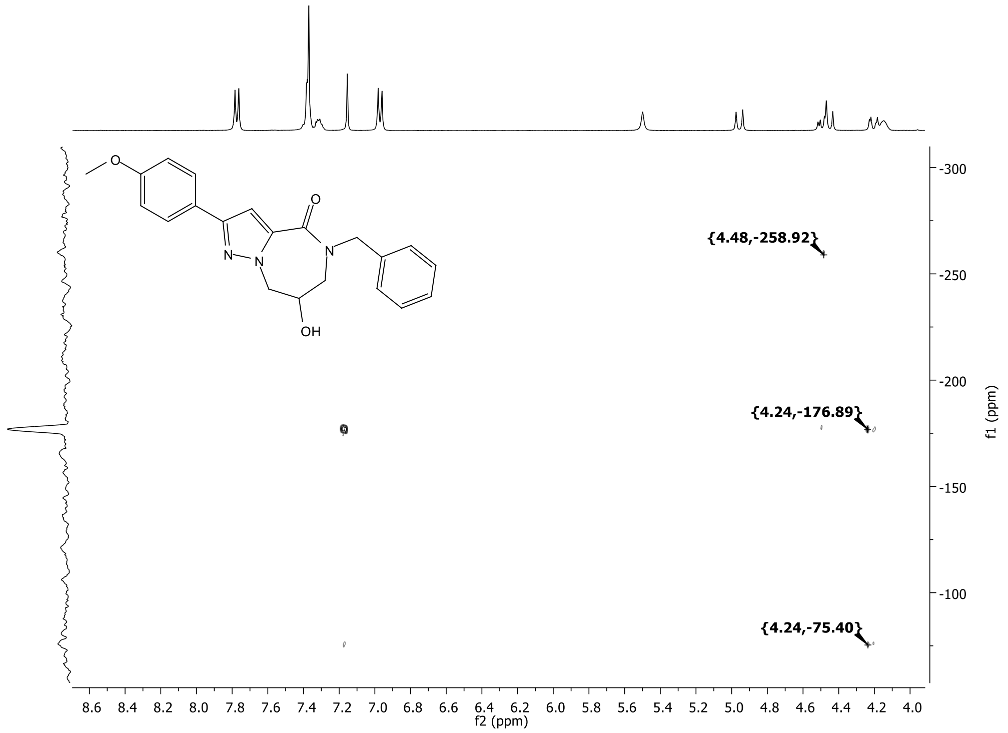

Figure S144. $^{1}H,^{15}N$-HMBC (40 MHz, DMSO-d_6) of 5-benzyl-7-hydroxy-2-(4-methoxyphenyl)-5,6,7,8-tetrahydro-4*H*-pyrazolo[1,5-*a*][1,4]diazepin-4-one (**4x**).

Compound Spectrum SmartFormula Report

Analysis Info

Analysis Name	D:\Data\KDD-183-1.d	Acquisition Date	8/10/2021 2:44:57 PM
Method	DirectInfusion_TuneLow_pos.m	Operator	hplc
Sample Name	KDD-183-1	Instrument	micrOTOF-Q III 8228888.20448
Comment	SB		

Acquisition Parameter

Source Type	ESI	Ion Polarity	Positive	Set Nebulizer	0.4 Bar
Focus	Not active	Set Capillary	4500 V	Set Dry Heater	180 °C
Scan Begin	50 m/z	Set End Plate Offset	-500 V	Set Dry Gas	4.0 l/min
Scan End	1000 m/z	Set Collision Cell RF	140.0 Vpp	Set Divert Valve	Waste

#	RT [min]	Area	Int. Type	I	S/N	Chromatogram	Max. m/z	FWHM [min]
n.a.	0.1	n.a.	Single spectrum	n.a.	n.a.	n.a.	226.9517	n.a.
n.a.	5.6	n.a.	Single spectrum	n.a.	n.a.	n.a.	386.1475	n.a.

+MS, 5.6min #333

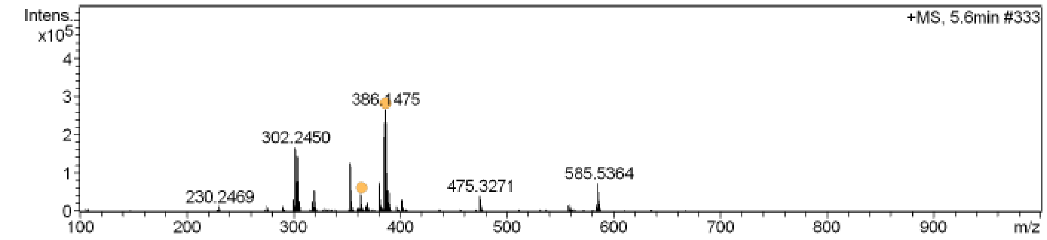

Meas. m/z	#	Ion Formula	m/z	err [ppm]	mSigma	# Sigma	Score	rdb	e⁻ Conf	N-Rule
364.1652	1	C21H22N3O3	364.1656	1.0	29.0	1	100.00	12.5	even	ok
386.1475	1	C21H21N3NaO3	386.1475	-0.0	1.4	1	100.00	12.5	even	ok

Figure S145. HRMS (ESI-TOF) spectrum of 5-benzyl-7-hydroxy-2-(4-methoxyphenyl)-5,6,7,8-tetrahydro-4*H*-pyrazolo[1,5-*a*][1,4]diazepin-4-one (**4x**).

3. Data of ethyl 1-(oxiran-2-ylmethyl)-1*H*-indole-2-carboxylates (6a–e) and ethyl 1-(oxiran-2-ylmethyl)-1*H*-benzo[*d*]imidazole-2-carboxylate (6f)

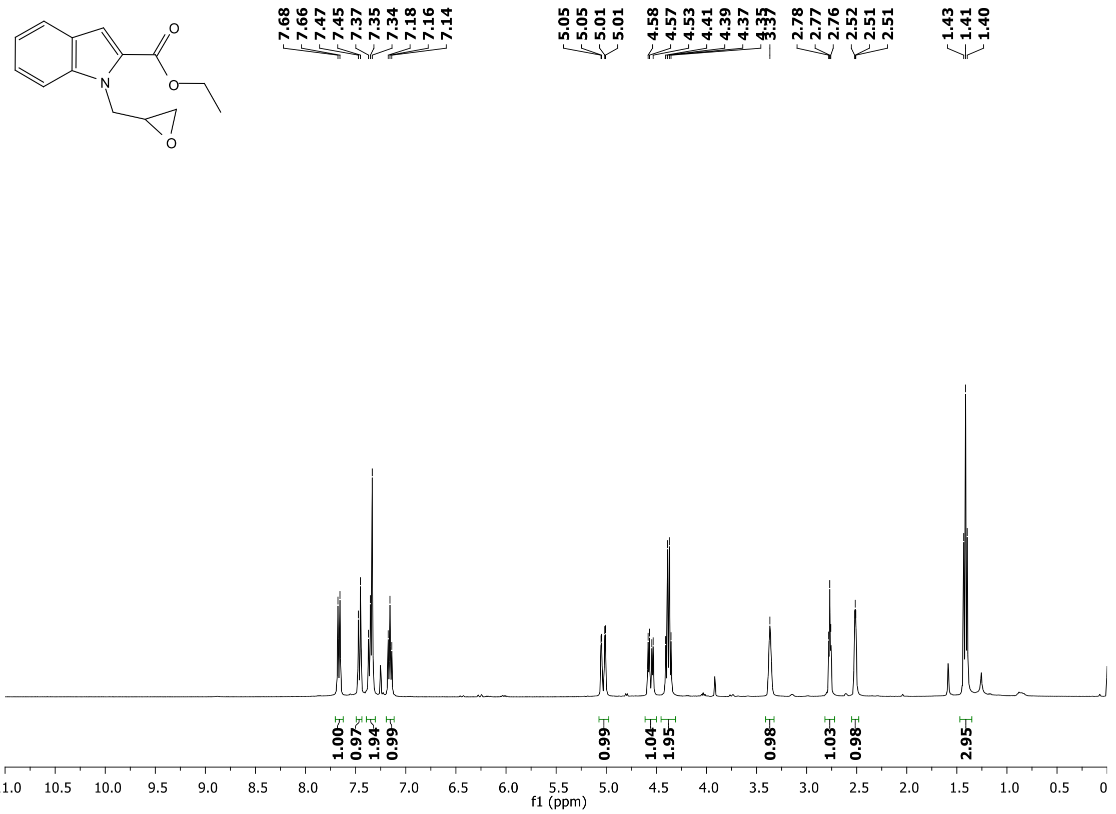

Figure S146. ^{1}H NMR spectrum (400 MHz, $CDCl_3$) of ethyl 1-(oxiran-2-ylmethyl)-1*H*-indole-2-carboxylate (**6a**).

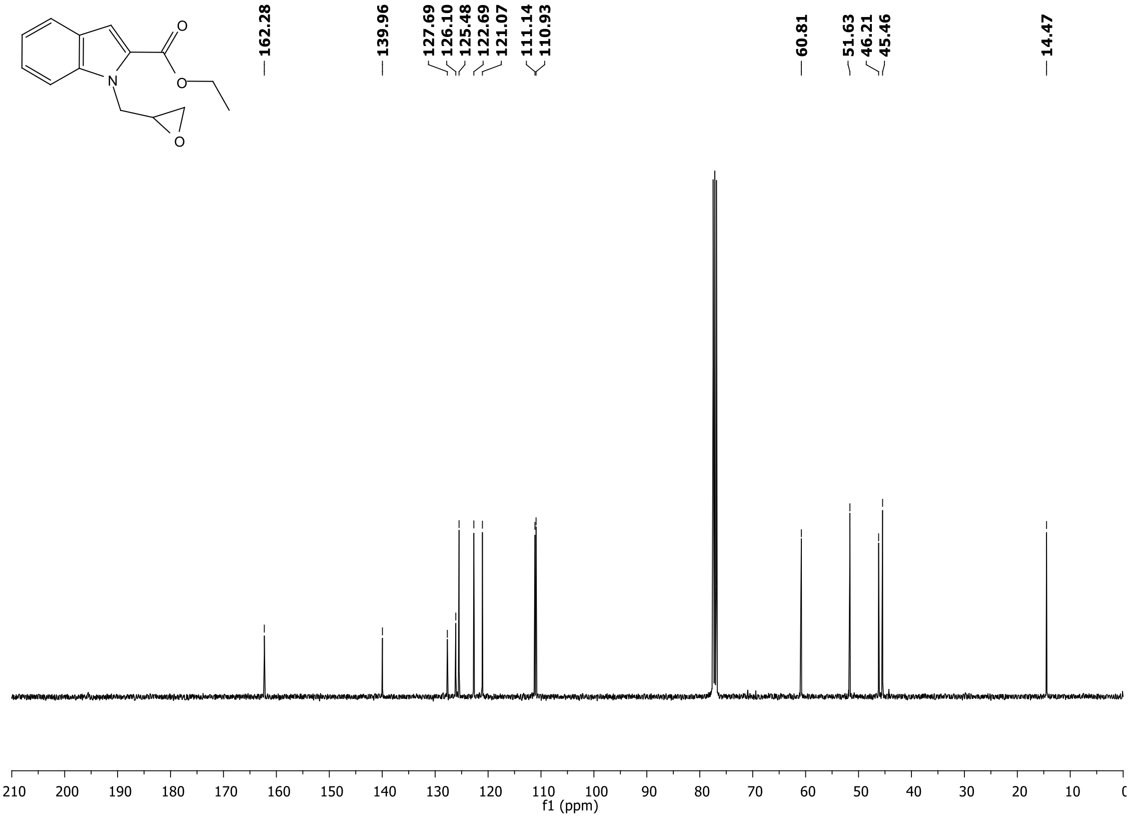

Figure S147. ^{13}C NMR spectrum (101 MHz, $CDCl_3$) of ethyl 1-(oxiran-2-ylmethyl)-1*H*-indole-2-carboxylate (**6a**).

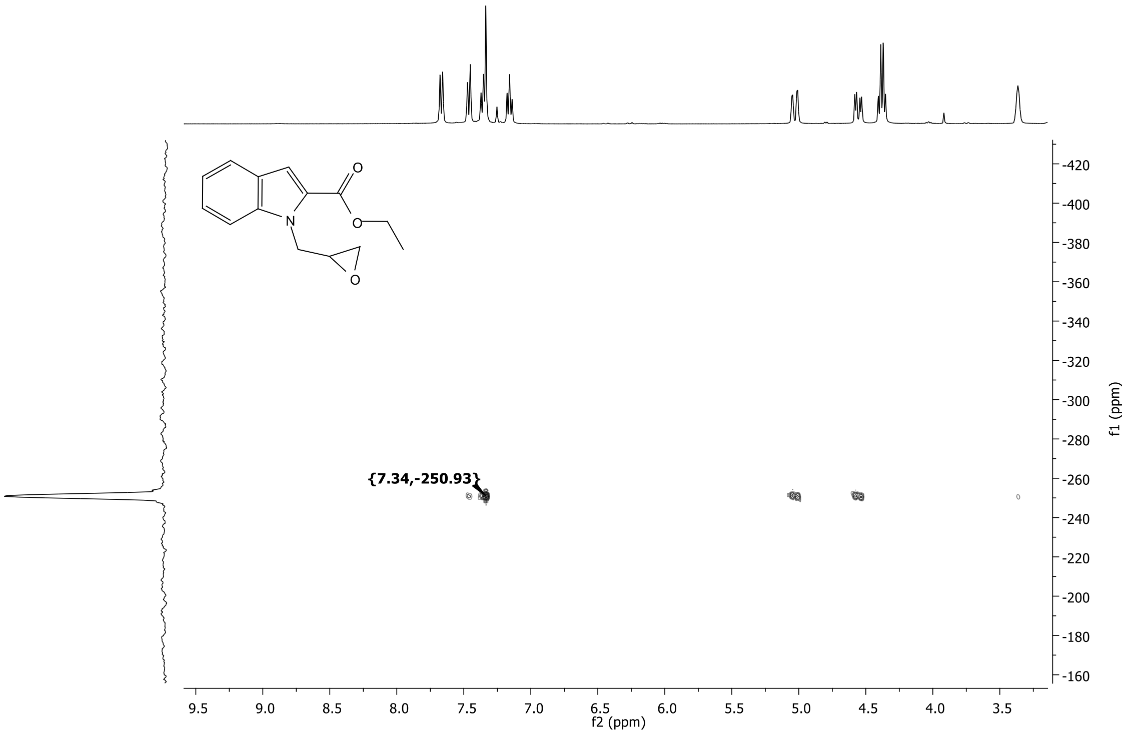

Figure S148. $^{1}H,^{15}N$-HMBC spectrum (40 MHz, $CDCl_3$) of ethyl 1-(oxiran-2-ylmethyl)-1*H*-indole-2-carboxylate (**6a**).

Qualitative Compound Report

**Data File**	221111_MV-55-2_01.d	**Sample Name**	MV-55-2
**Sample Type**	Sample	**Position**	
**Instrument Name**	G6230B TOF	**User Name**	
**Acq Method**	HRMS_12min_ref.m	**Acquired Time**	11-Nov-22 1:27:23 PM
**IRM Calibration Status**	Success	**DA Method**	test.m
**Comment**			
**Sample Group**		**Info.**	
**Stream Name**		**Acquisition SW Version**	6200 series TOF/6500 series Q-TOF B.09.00 (B9044.1 SP1)

Compound Table

Compound Label	RT	Mass	Abund	Formula	Tgt Mass	Diff (ppm)
Cpd 1: C14 H15 N O3	6.806	245.1049	1521739	C14 H15 N O3	245.1052	-1.16

Compound Label	m/z	RT	Algorithm	Mass
Cpd 1: C14 H15 N O3	246.1119	6.806	Find By Formula	245.1049

Compound Chromatograms

MS Spectrum

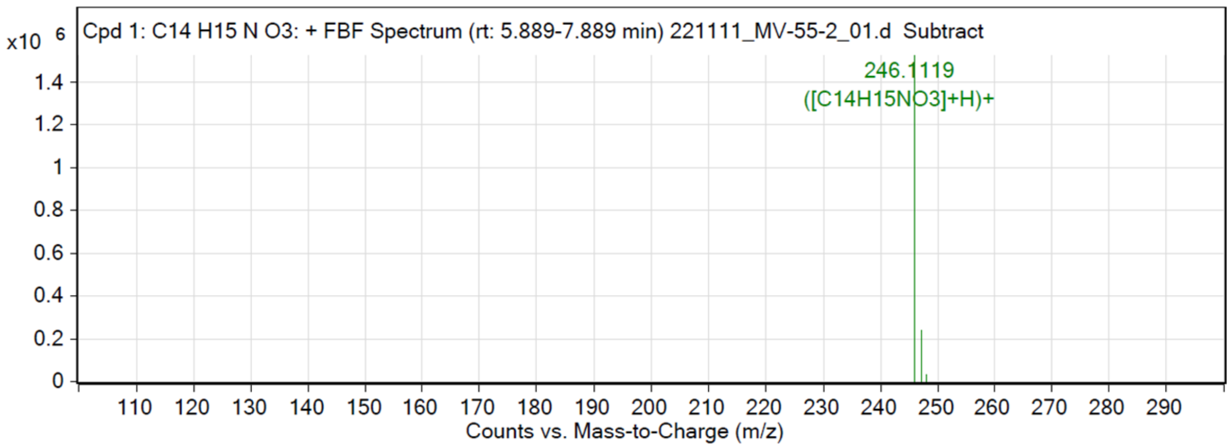

Figure S149. HRMS (ESI-TOF) spectrum of ethyl 1-(oxiran-2-ylmethyl)-1*H*-indole-2-carboxylate (**6a**).

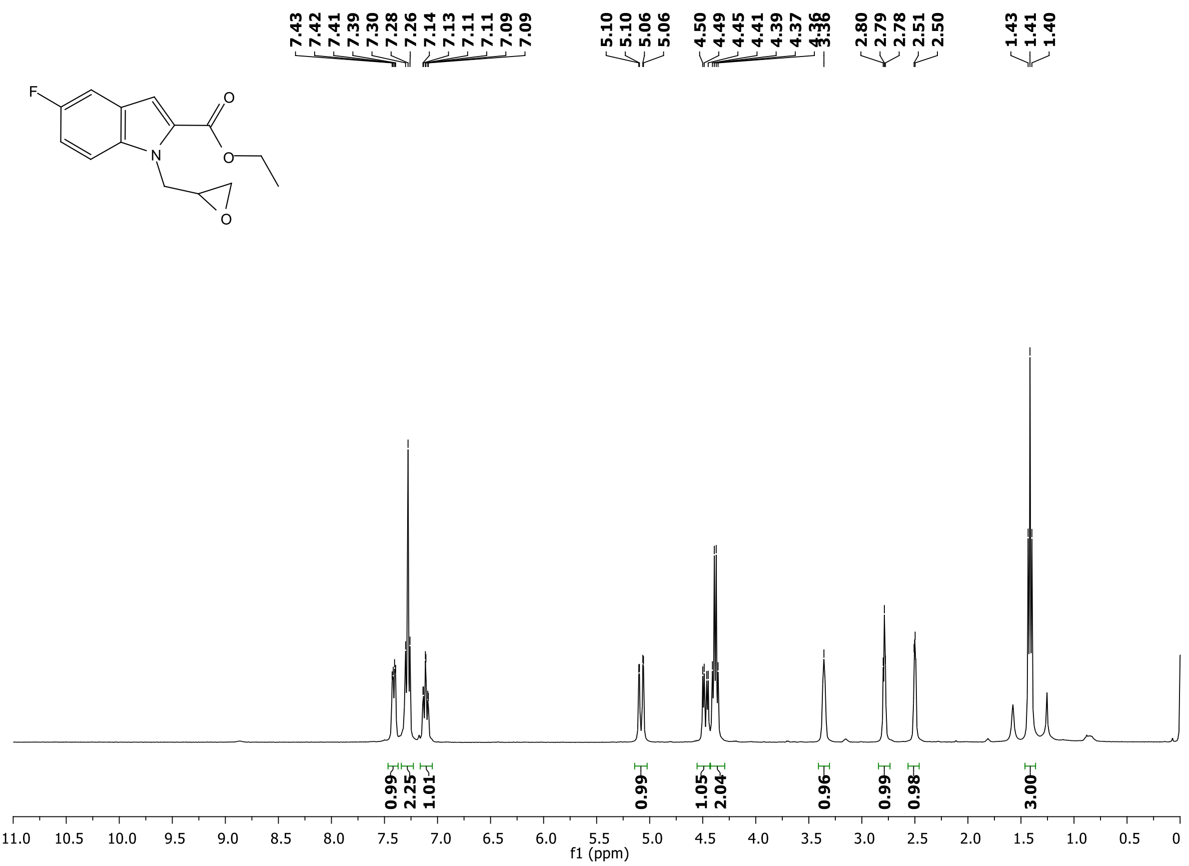

Figure S150. ^{1}H NMR spectrum (400 MHz, $CDCl_3$) of ethyl 5-fluoro-1-(oxiran-2-ylmethyl)-1*H*-indole-2-carboxylate (**6b**).

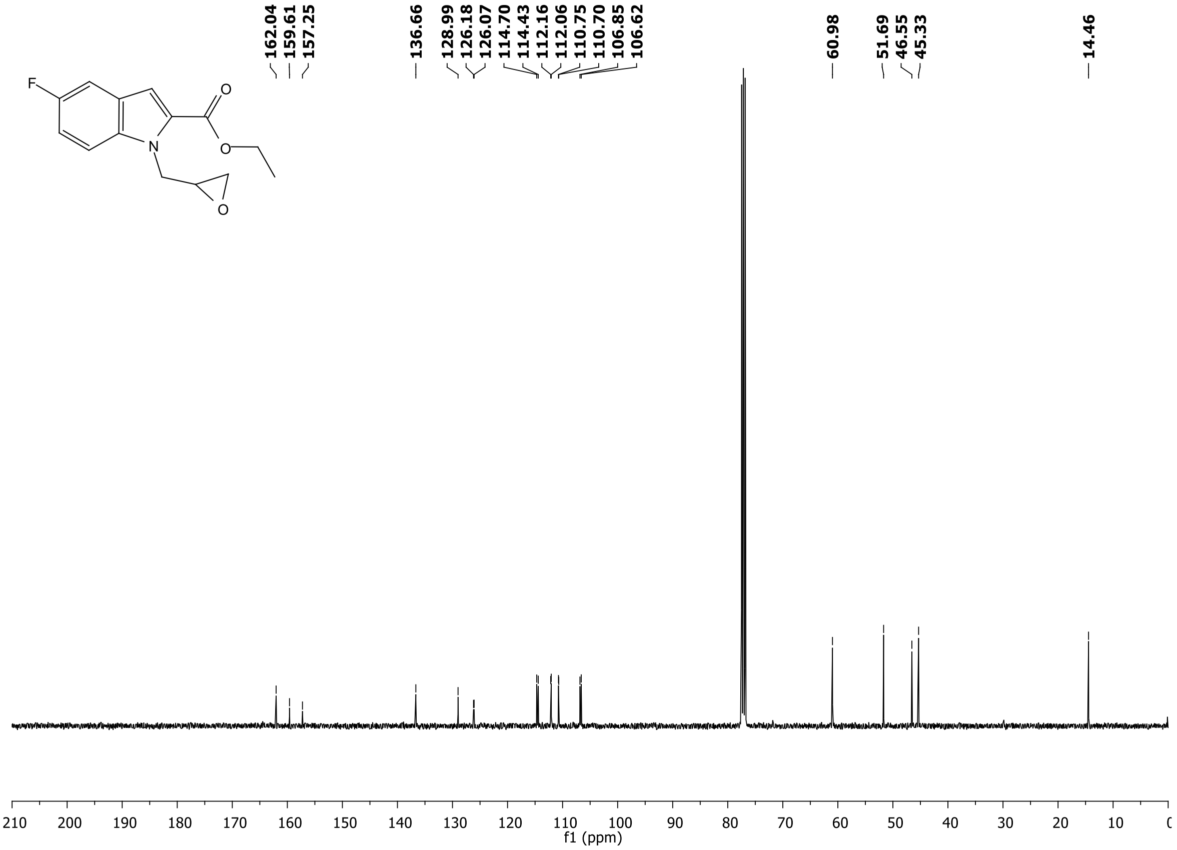

Figure S151. ^{13}C NMR spectrum (101 MHz, $CDCl_3$) of ethyl 5-fluoro-1-(oxiran-2-ylmethyl)-1*H*-indole-2-carboxylate (**6b**).

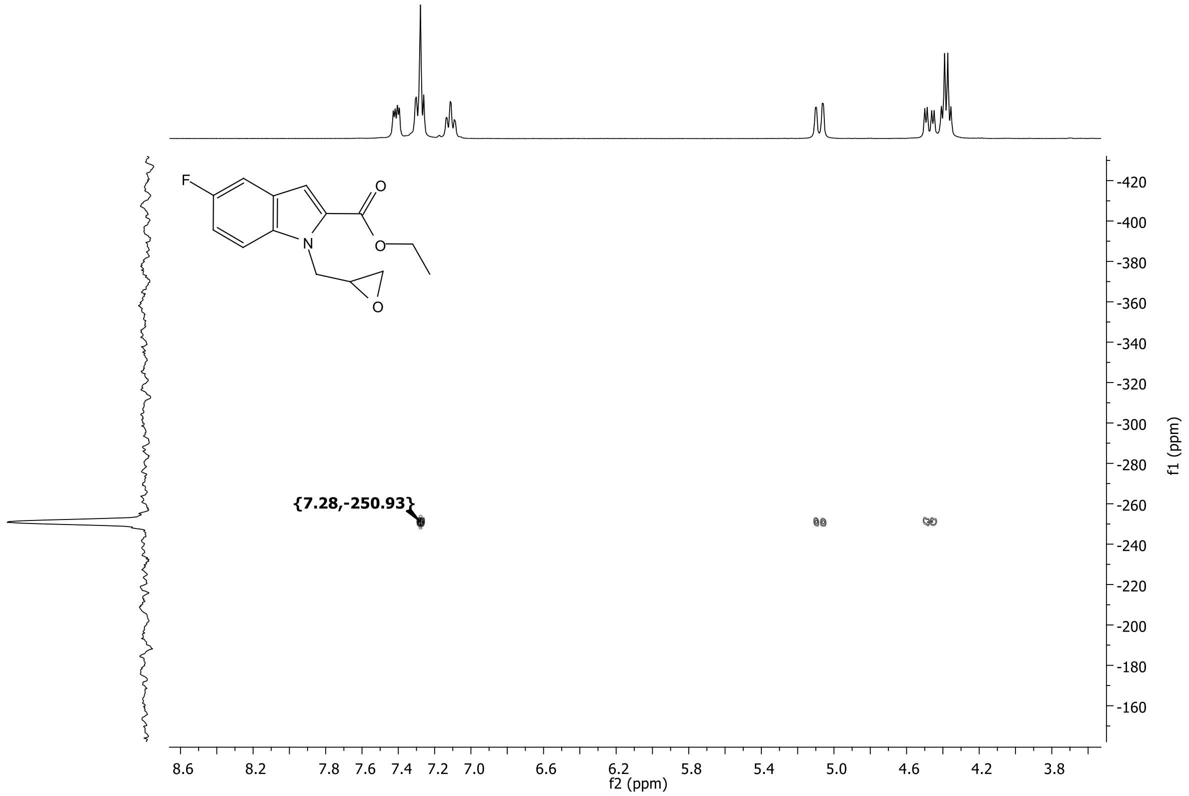

Figure S152. $^{1}H,^{15}N$-HMBC spectrum (40 MHz, $CDCl_3$) of ethyl 5-fluoro-1-(oxiran-2-ylmethyl)-1*H*-indole-2-carboxylate (**6b**).

Figure S153. ^{19}F NMR spectrum (376 MHz, DMSO-d_6) of ethyl 5-fluoro-1-(oxiran-2-ylmethyl)-1*H*-indole-2-carboxylate (**6b**).

Qualitative Compound Report

**Data File**	221114_MV-66-4_01.d	**Sample Name**	MV-66-4
**Sample Type**	Sample	**Position**	
**Instrument Name**	G6230B TOF	**User Name**	
**Acq Method**	HRMS_12min_ref.m	**Acquired Time**	14-Nov-22 9:37:36 AM
**IRM Calibration Status**	Success	**DA Method**	test.m
**Comment**			
**Sample Group**		**Info.**	
**Stream Name**		**Acquisition SW Version**	6200 series TOF/6500 series Q-TOF B.09.00 (B9044.1 SP1)

Compound Table

Compound Label	RT	Mass	Abund	Formula	Tgt Mass	Diff (ppm)
Cpd 1: C14 H14 F N O3	7.996	263.096	1518870	C14 H14 F N O3	263.0958	0.71

Compound Label	*m/z*	RT	Algorithm	Mass
Cpd 1: C14 H14 F N O3	264.1031	7.996	Find By Formula	263.096

Compound Chromatograms

MS Spectrum

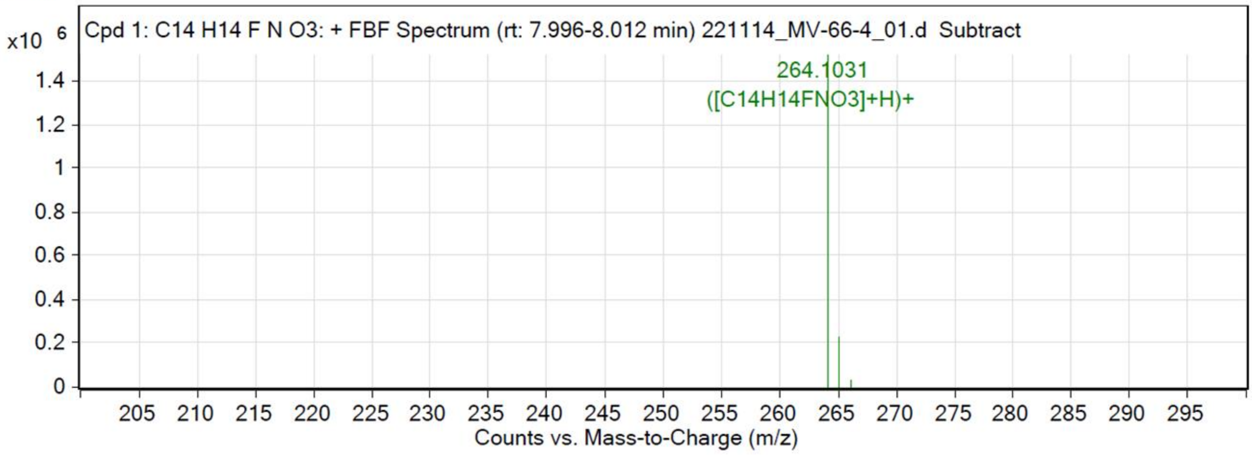

Figure S154. HRMS (ESI-TOF) spectrum of ethyl 5-fluoro-1-(oxiran-2-ylmethyl)-1*H*-indole-2-carboxylate (**6b**).

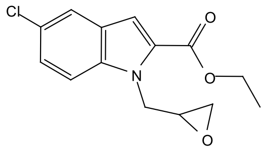

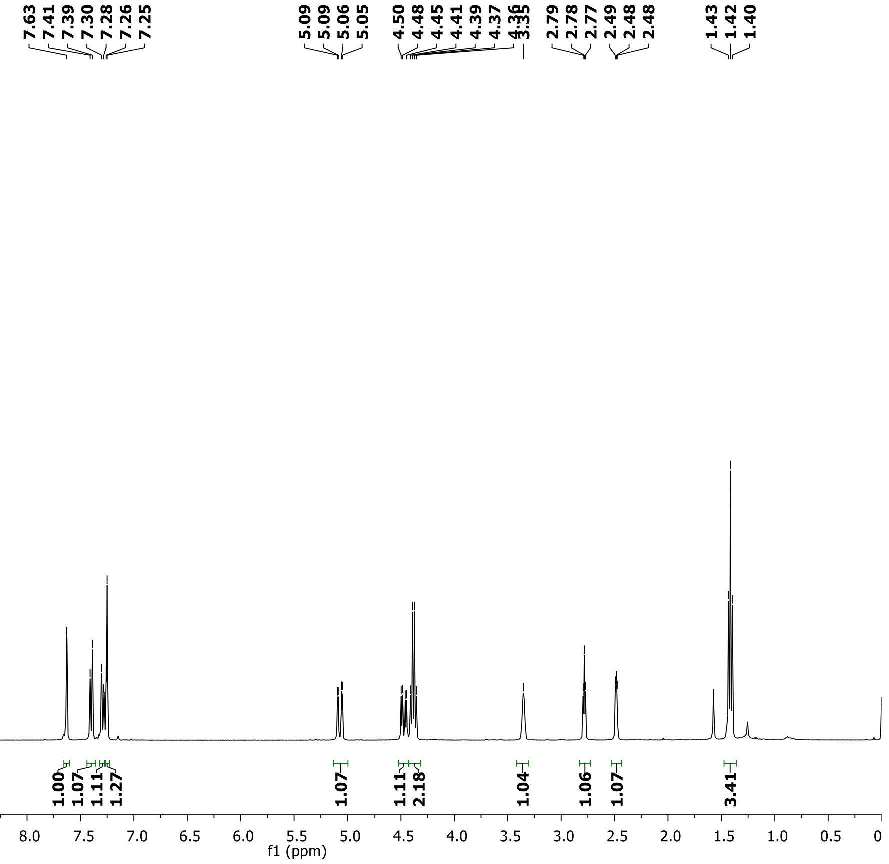

Figure S155. ^{1}H NMR spectrum (400 MHz, $CDCl_3$) of ethyl 5-chloro-1-(oxiran-2-ylmethyl)-1*H*-indole-2-carboxylate (**6c**).

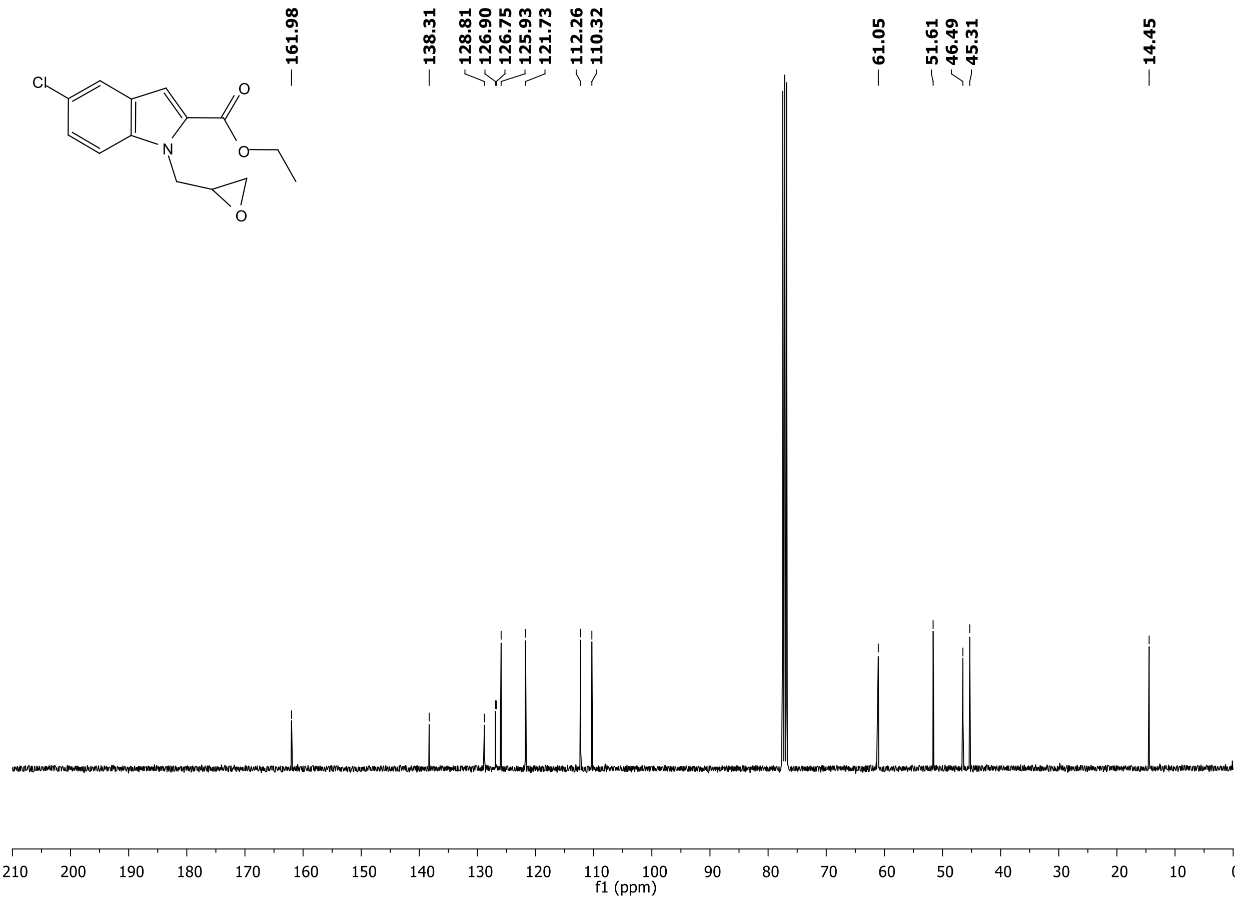

Figure S156. ^{13}C NMR spectrum (101 MHz, $CDCl_3$) of ethyl 5-chloro-1-(oxiran-2-ylmethyl)-1*H*-indole-2-carboxylate (**6c**).

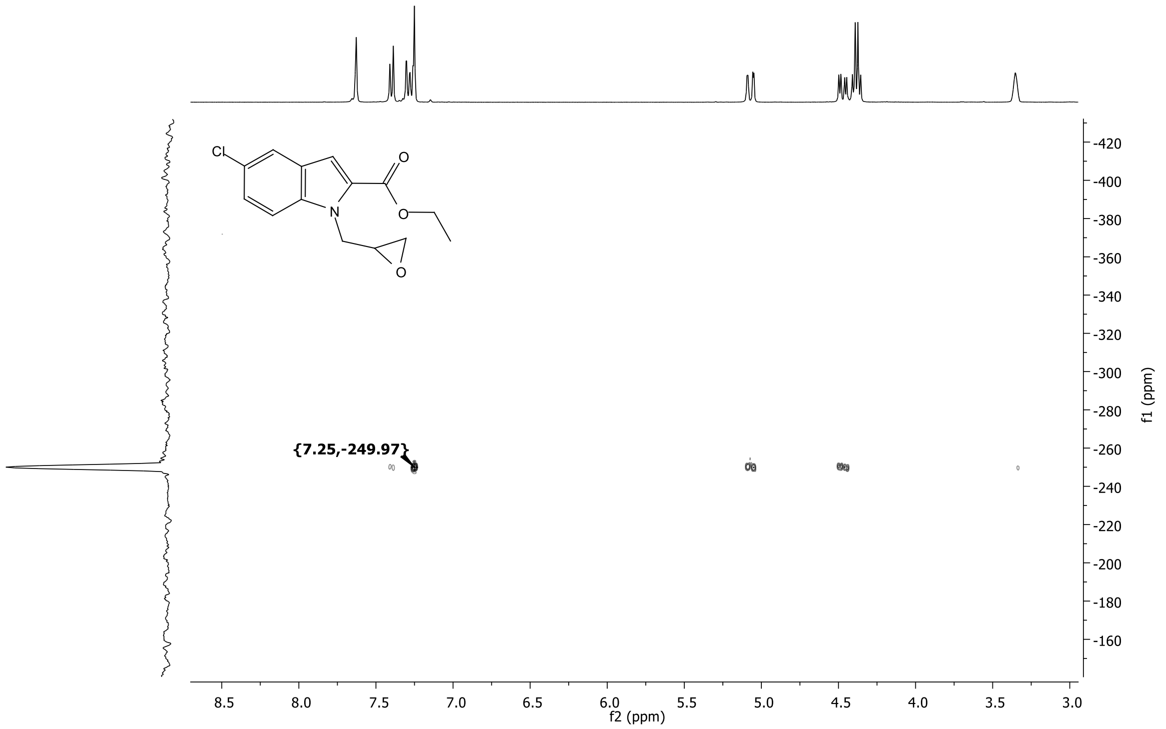

Figure S157. ^{1}H,^{15}N-HMBC spectrum (40 MHz, $CDCl_3$) of ethyl 5-chloro-1-(oxiran-2-ylmethyl)-1*H*-indole-2-carboxylate (**6c**).

Qualitative Compound Report

**Data File**	221114_MV-57-1_01.d	**Sample Name**	MV-57-1
**Sample Type**	Sample	**Position**	
**Instrument Name**	G6230B TOF	**User Name**	
**Acq Method**	HRMS_12min_ref.m	**Acquired Time**	14-Nov-22 9:16:13 AM
**IRM Calibration Status**	Success	**DA Method**	test.m
**Comment**			
**Sample Group**		**Info.**	
**Stream Name**		**Acquisition SW Version**	6200 series TOF/6500 series Q-TOF B.09.00 (B9044.1 SP1)

Compound Table

Compound Label	RT	Mass	Abund	Formula	Tgt Mass	Diff (ppm)
Cpd 1: C14 H14 Cl N O3	8.091	279.0664	1722346	C14 H14 Cl N O3	279.0662	0.63

Compound Label	*m/z*	RT	Algorithm	Mass
Cpd 1: C14 H14 Cl N O3	280.0733	8.091	Find By Formula	279.0664

Compound Chromatograms

MS Spectrum

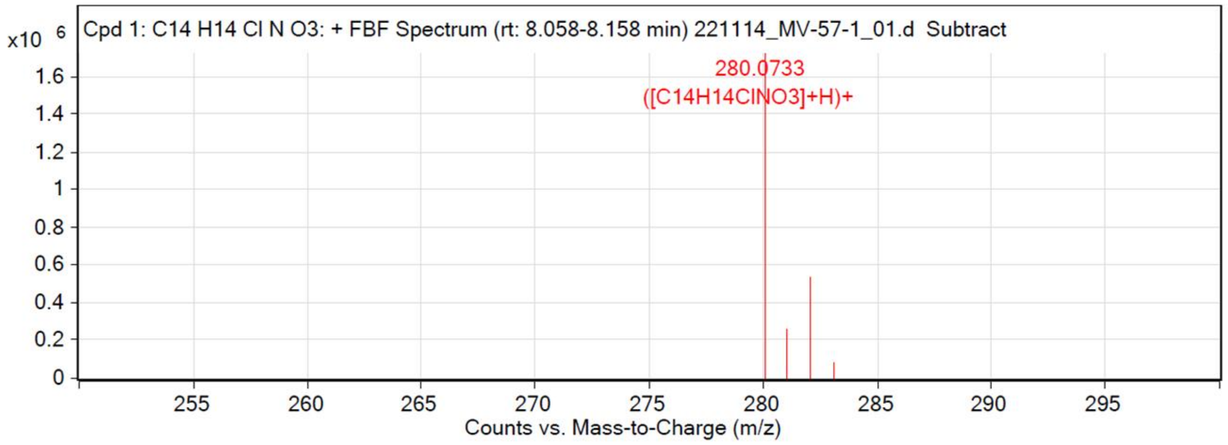

Figure S158. HRMS (ESI-TOF) spectrum of ethyl 5-chloro-1-(oxiran-2-ylmethyl)-1*H*-indole-2-carboxylate (**6c**).

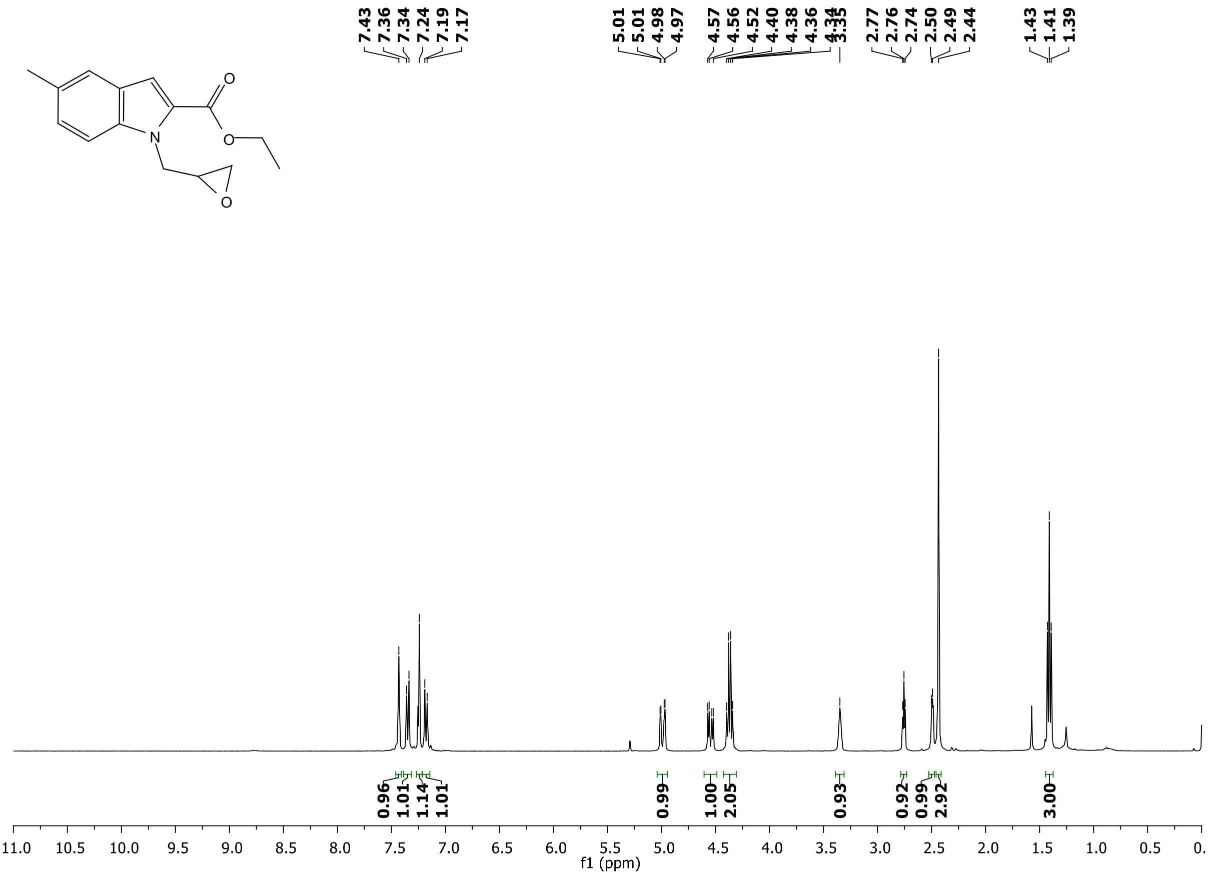

Figure S159. ^{1}H NMR spectrum (400 MHz, $CDCl_3$) of ethyl 5-methyl-1-(oxiran-2-ylmethyl)-1*H*-indole-2-carboxylate (**6d**).

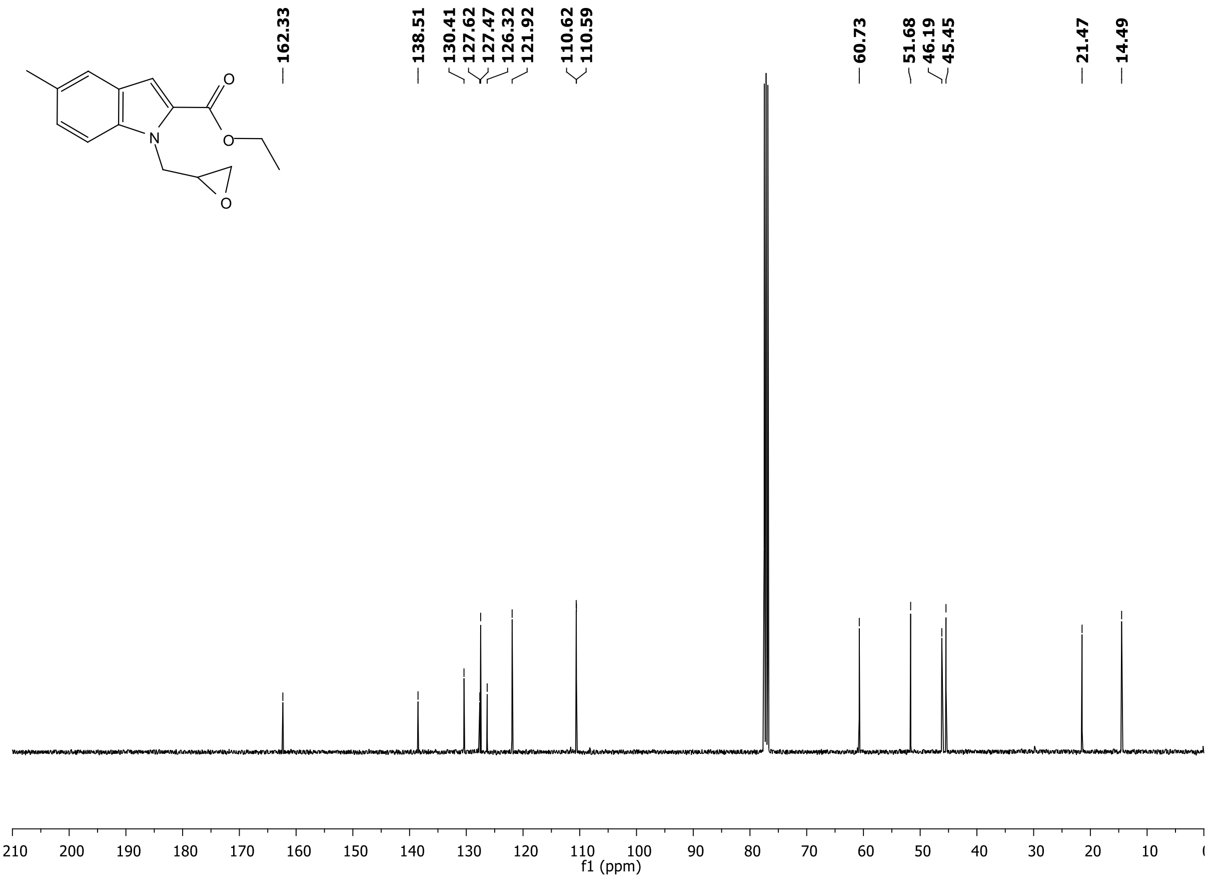

Figure S160. ^{13}C NMR spectrum (101 MHz, $CDCl_3$) of ethyl 5-methyl-1-(oxiran-2-ylmethyl)-1*H*-indole-2-carboxylate (**7d**).

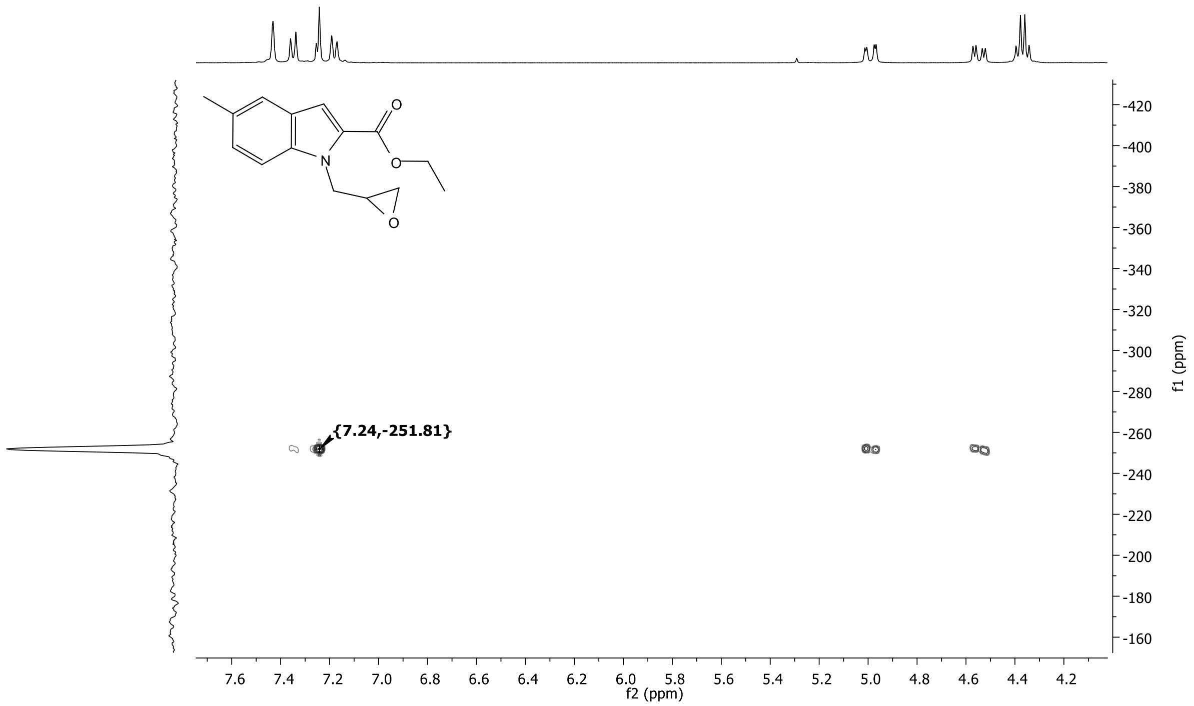

Figure S161. ^{1}H,^{15}N-HMBC spectrum (40 MHz, $CDCl_3$) of ethyl 5-methyl-1-(oxiran-2-ylmethyl)-1*H*-indole-2-carboxylate (**6d**).

Qualitative Compound Report

**Data File**	221111_MV-63-1_01.d	**Sample Name**	MV-63-1
**Sample Type**	Sample	**Position**	
**Instrument Name**	G6230B TOF	**User Name**	
**Acq Method**	HRMS_12min_ref.m	**Acquired Time**	11-Nov-22 2:10:08 PM
**IRM Calibration Status**	Success	**DA Method**	test.m
**Comment**			
**Sample Group**		**Info.**	
**Stream Name**		**Acquisition SW Version**	6200 series TOF/6500 series Q-TOF B.09.00 (B9044.1 SP1)

Compound Table

Compound Label	RT	Mass	Abund	Formula	Tgt Mass	Diff (ppm)
Cpd 1: C15 H17 N O3	6.628	259.1207	6611854	C15 H17 N O3	259.1208	-0.7

Compound Label	m/z	RT	Algorithm	Mass
Cpd 1: C15 H17 N O3	260.1277	6.628	Find By Formula	259.1207

Compound Chromatograms

MS Spectrum

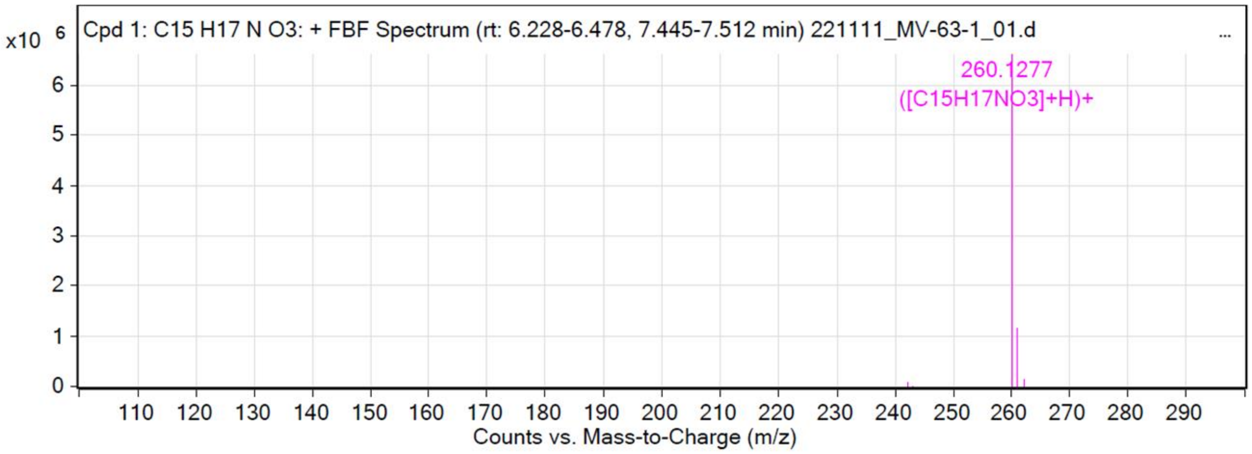

Figure S162. HRMS (ESI-TOF) spectrum of ethyl 5-methyl-1-(oxiran-2-ylmethyl)-1*H*-indole-2-carboxylate (**6d**).

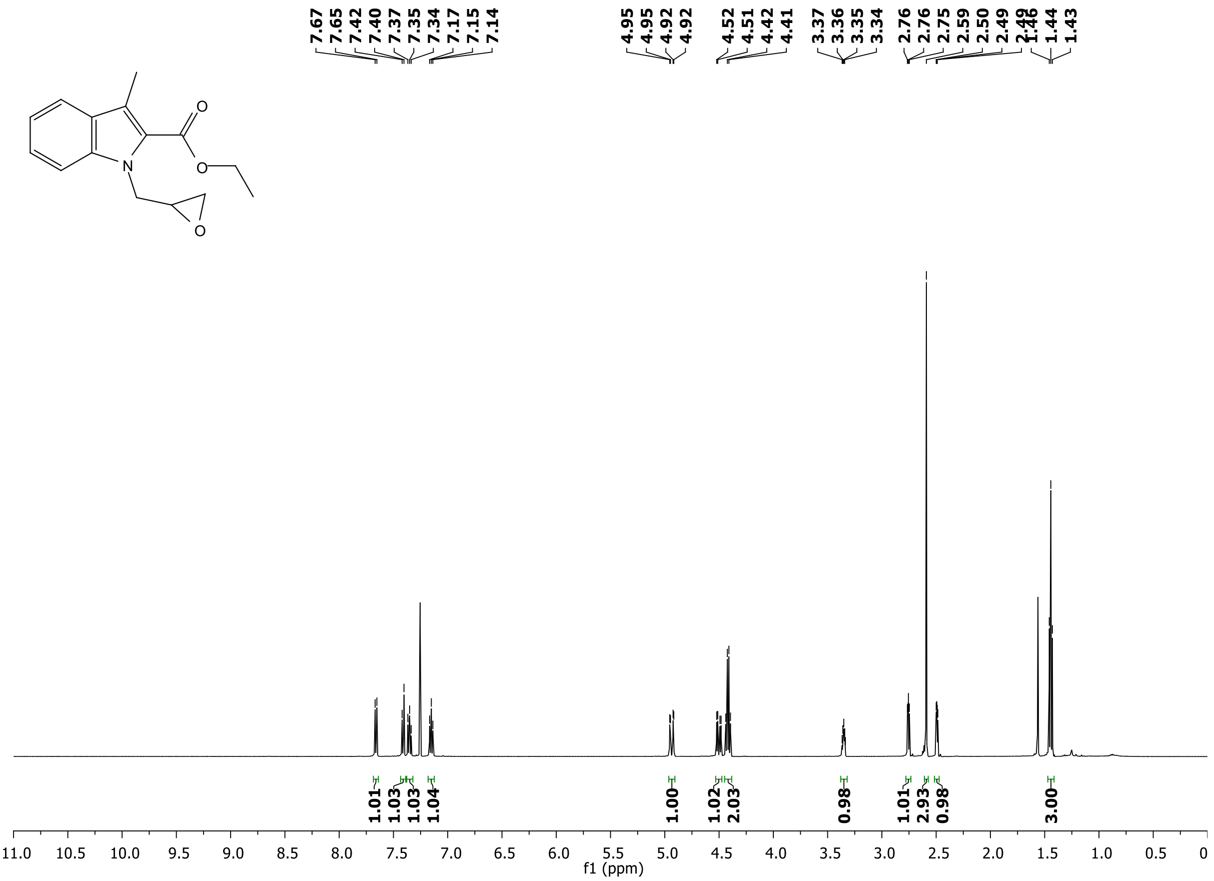

Figure S163. ^{1}H NMR spectrum (500 MHz, $CDCl_3$) of ethyl 3-methyl-1-(oxiran-2-ylmethyl)-1*H*-indole-2-carboxylate (**6e**).

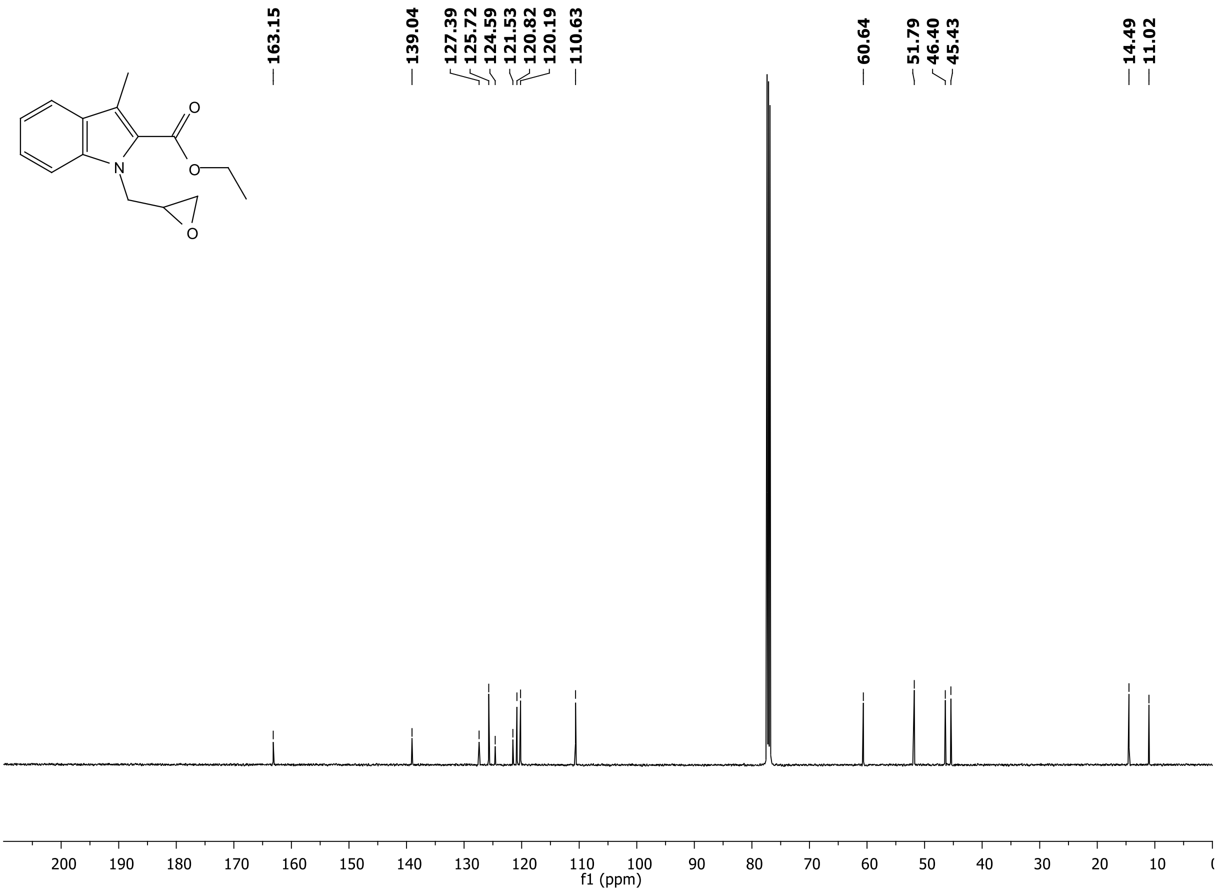

Figure S164. ^{13}C NMR spectrum (125 MHz, $CDCl_3$) of ethyl 3-methyl-1-(oxiran-2-ylmethyl)-1*H*-indole-2-carboxylate (**6e**).

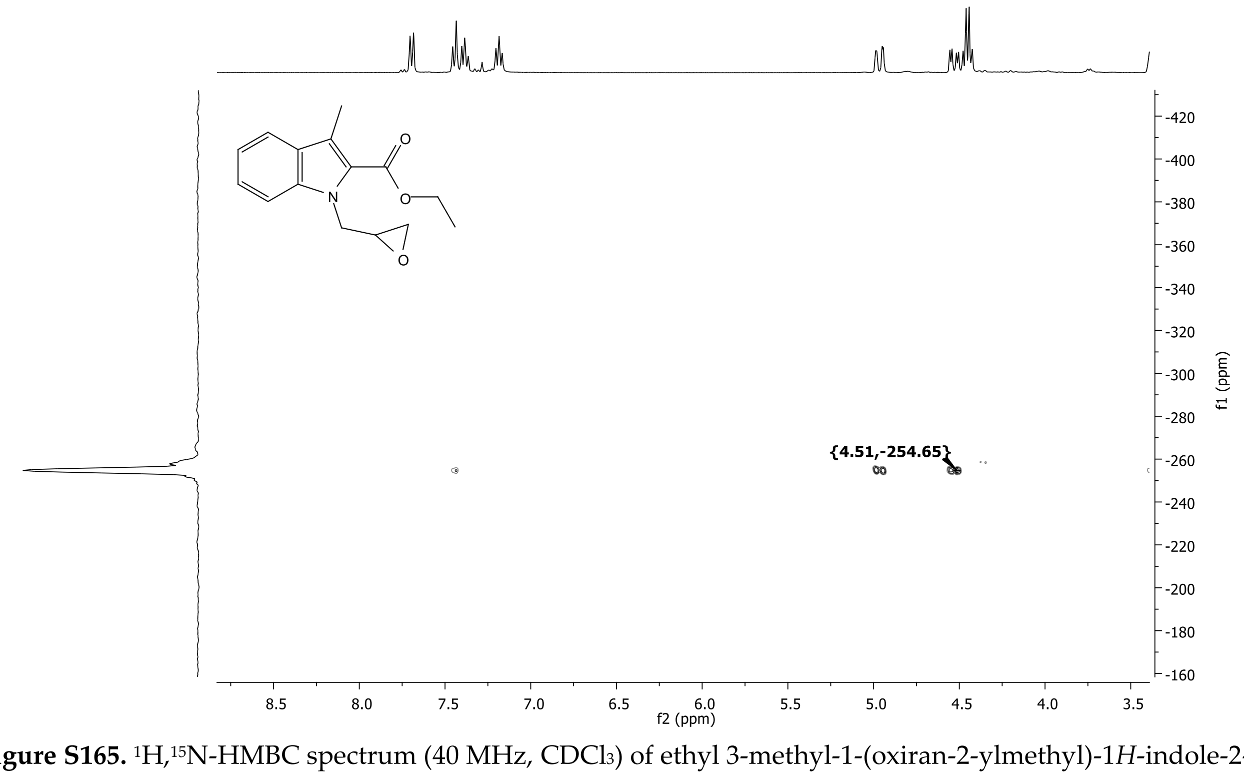

Figure S165. ^{1}H,^{15}N-HMBC spectrum (40 MHz, $CDCl_3$) of ethyl 3-methyl-1-(oxiran-2-ylmethyl)-1*H*-indole-2-carboxylate (**6e**).

Qualitative Compound Report

**Data File**	220720_VMO-epi4_02.d	**Sample Name**	VMO-epi4
**Sample Type**	Sample	**Position**	
**Instrument Name**	G6230B TOF	**User Name**	
**Acq Method**	HRMS_12min_ref.m	**Acquired Time**	20-Jul-22 9:56:23 AM
**IRM Calibration Status**	Success	**DA Method**	test.m
**Comment**			
**Sample Group**		**Info.**	
**Stream Name**		**Acquisition SW Version**	6200 series TOF/6500 series Q-TOF B.09.00 (B9044.1 SP1)

Compound Table

Compound Label	RT	Mass	Abund	Formula	Tgt Mass	Diff (ppm)
Cpd 1: C15 H17 N O3	2.479	259.1206	1044294	C15 H17 N O3	259.1208	-0.91

Compound Label	*m/z*	RT	Algorithm	Mass
Cpd 1: C15 H17 N O3	260.1276	2.479	Find By Formula	259.1206

Compound Chromatograms

MS Spectrum

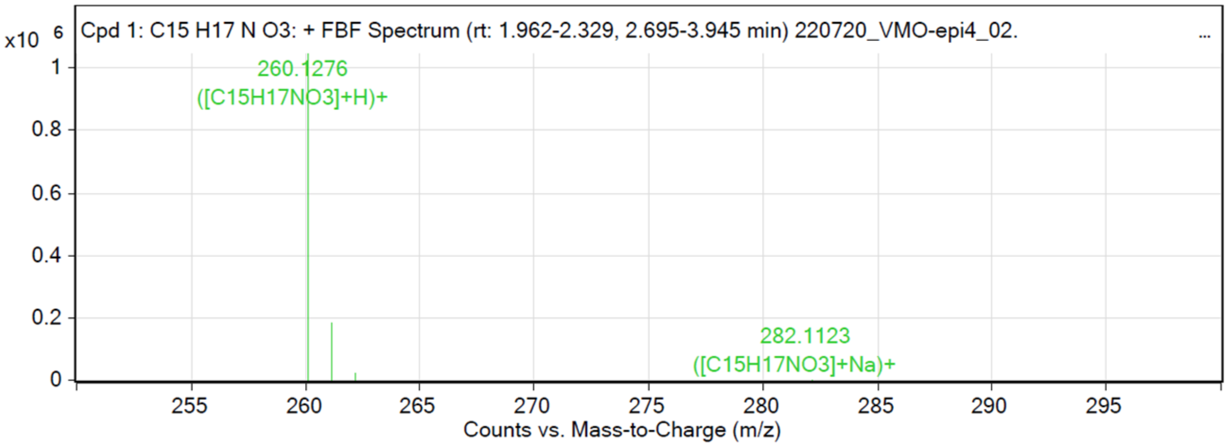

Figure S166. HRMS (ESI-TOF) spectrum of ethyl 3-methyl-1-(oxiran-2-ylmethyl)-1*H*-indole-2-carboxylate (**6e**).

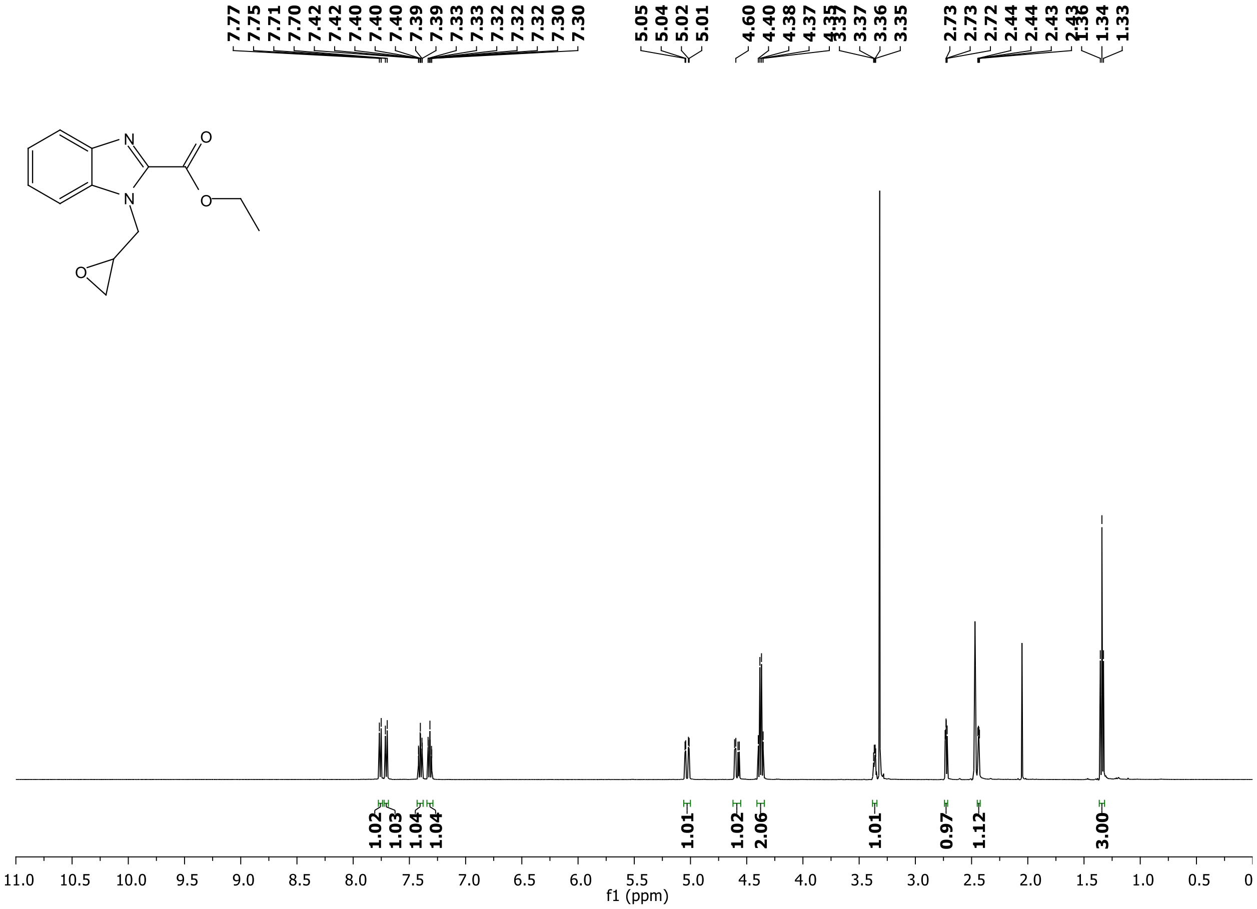

Figure S167. ^{1}H NMR spectrum (500 MHz, DMSO-d_6) of ethyl 1-(oxiran-2-ylmethyl)-1*H*-benzo[*d*]imidazole-2-carboxylate (**6f**).

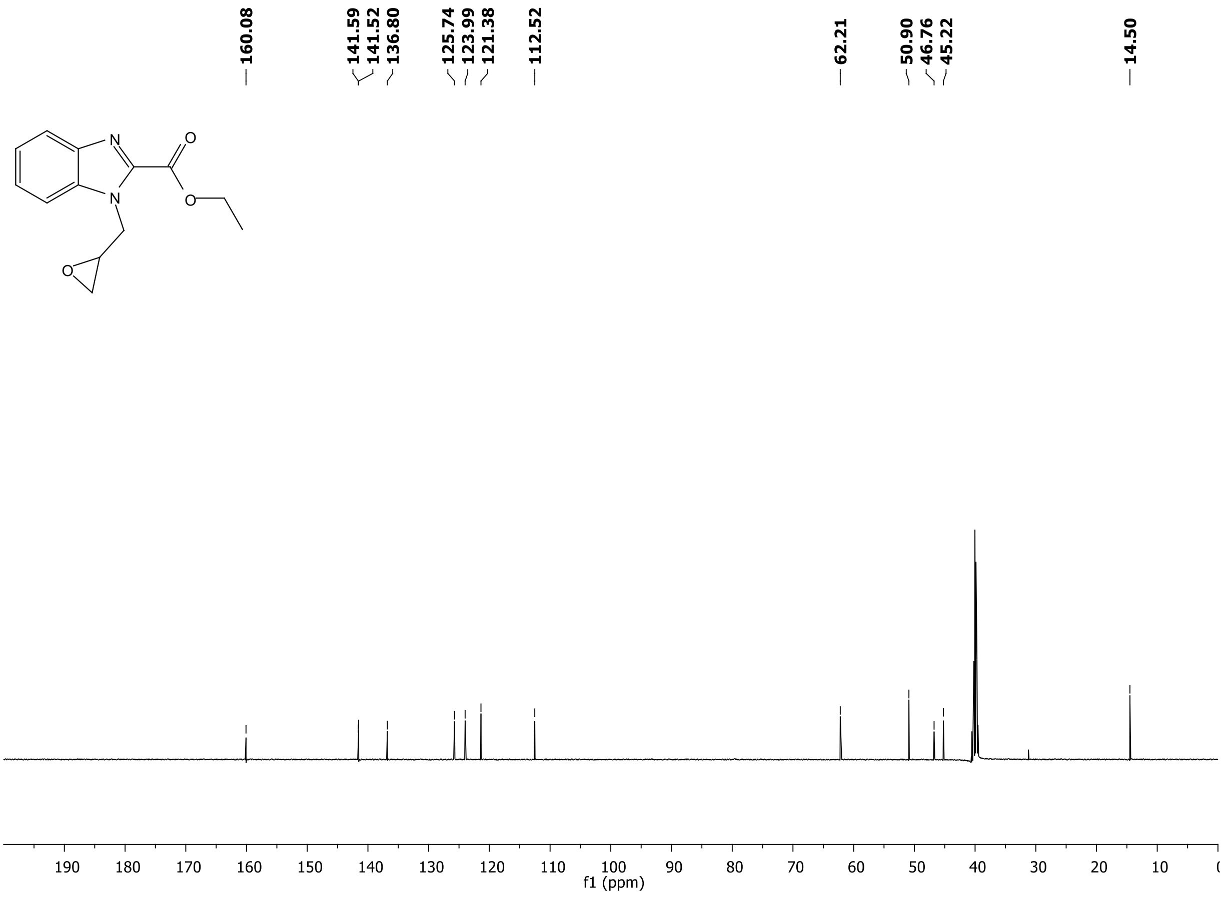

Figure S168. ^{13}C NMR spectrum (125 MHz, DMSO-d_6) of ethyl 1-(oxiran-2-ylmethyl)-1*H*-benzo[*d*]imidazole-2-carboxylate (**6f**).

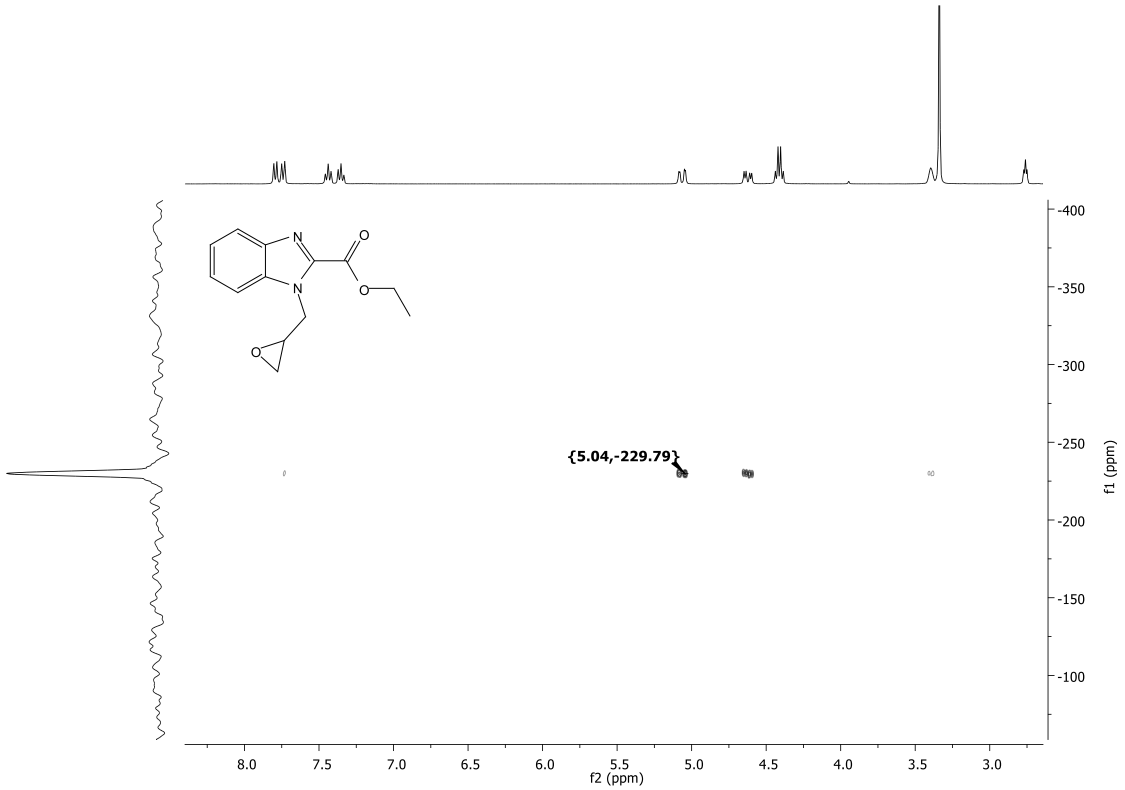

Figure S169. ^{1}H,^{15}N-HMBC spectrum (40 MHz, DMSO-d_6) of ethyl 1-(oxiran-2-ylmethyl)-1*H*-benzo[*d*]imidazole-2-carboxylate (**6f**).

Qualitative Compound Report

**Data File**	221101_VMO-031_01.d	**Sample Name**	VMO-031
**Sample Type**	Sample	**Position**	
**Instrument Name**	G6230B TOF	**User Name**	
**Acq Method**	HRMS_12min_ref.m	**Acquired Time**	01-Nov-22 8:59:56 AM
**IRM Calibration Status**	Success	**DA Method**	test.m
**Comment**			
**Sample Group**		**Info.**	
**Stream Name**		**Acquisition SW Version**	6200 series TOF/6500 series Q-TOF B.09.00 (B9044.1 SP1)

Compound Table

Compound Label	RT	Mass	Abund	Formula	Tgt Mass	Diff (ppm)
Cpd 1: C13 H14 N2 O3	2.556	246.1001	1400537	C13 H14 N2 O3	246.1004	-1.58

Compound Label	*m/z*	RT	Algorithm	Mass
Cpd 1: C13 H14 N2 O3	247.1071	2.556	Find By Formula	246.1001

Compound Chromatograms

MS Spectrum

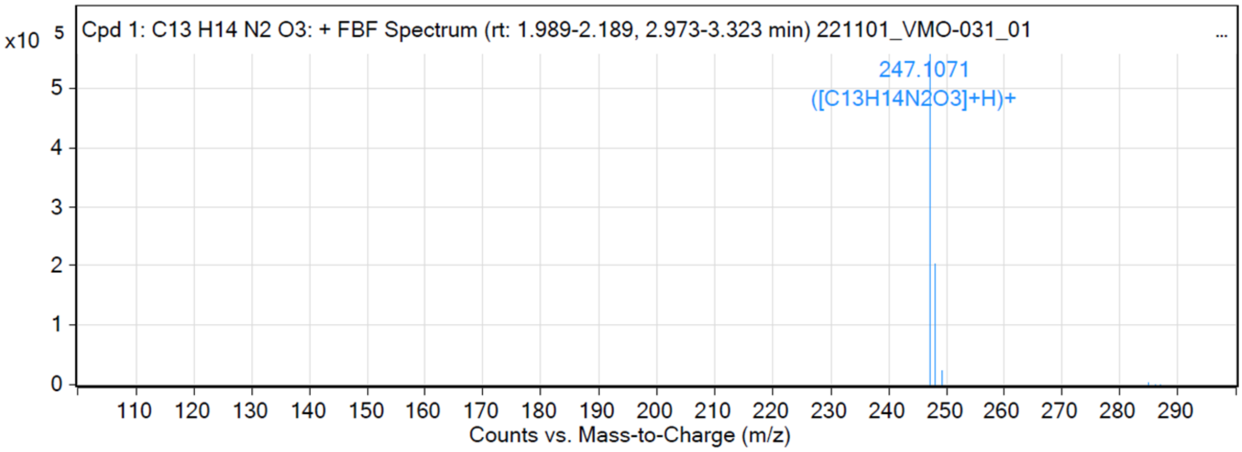

Figure S170. HRMS (ESI-TOF) spectrum of ethyl 1-(oxiran-2-ylmethyl)-1*H*-benzo[*d*]imidazole-2-carboxylate (**6f**).

4. Data of 4-hydroxy-2,3,4,5-tetrahydro-1*H*-[1,4]diazepino[1,2-*a*]indol-1-ones (7a–f) and 4-hydroxy-2,3,4,5-tetrahydro-1*H*-benzo[4,5]imidazo[1,2-*a*][1,4]diazepin-1-one (7g)

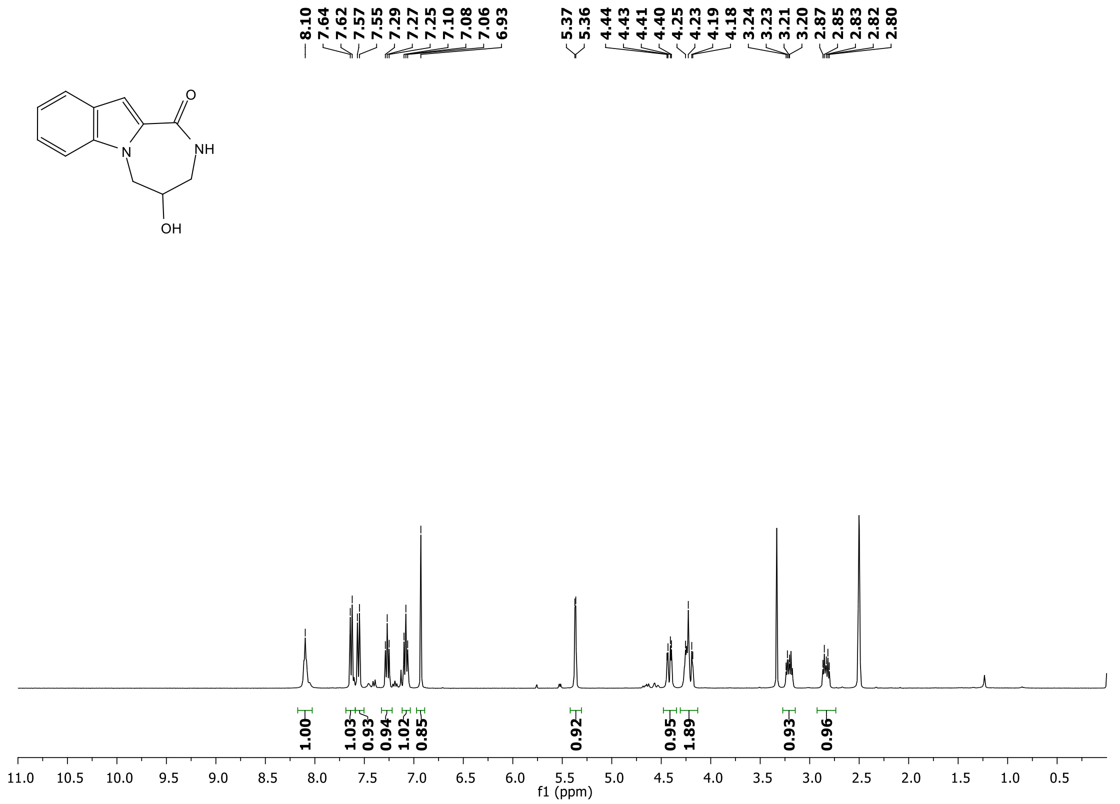

Figure S171. ^{1}H NMR spectrum (400 MHz, DMSO-d_6) of 4-hydroxy-2,3,4,5-tetrahydro-1*H*-[1,4]diazepino[1,2-*a*]indol-1-one (**7a**).

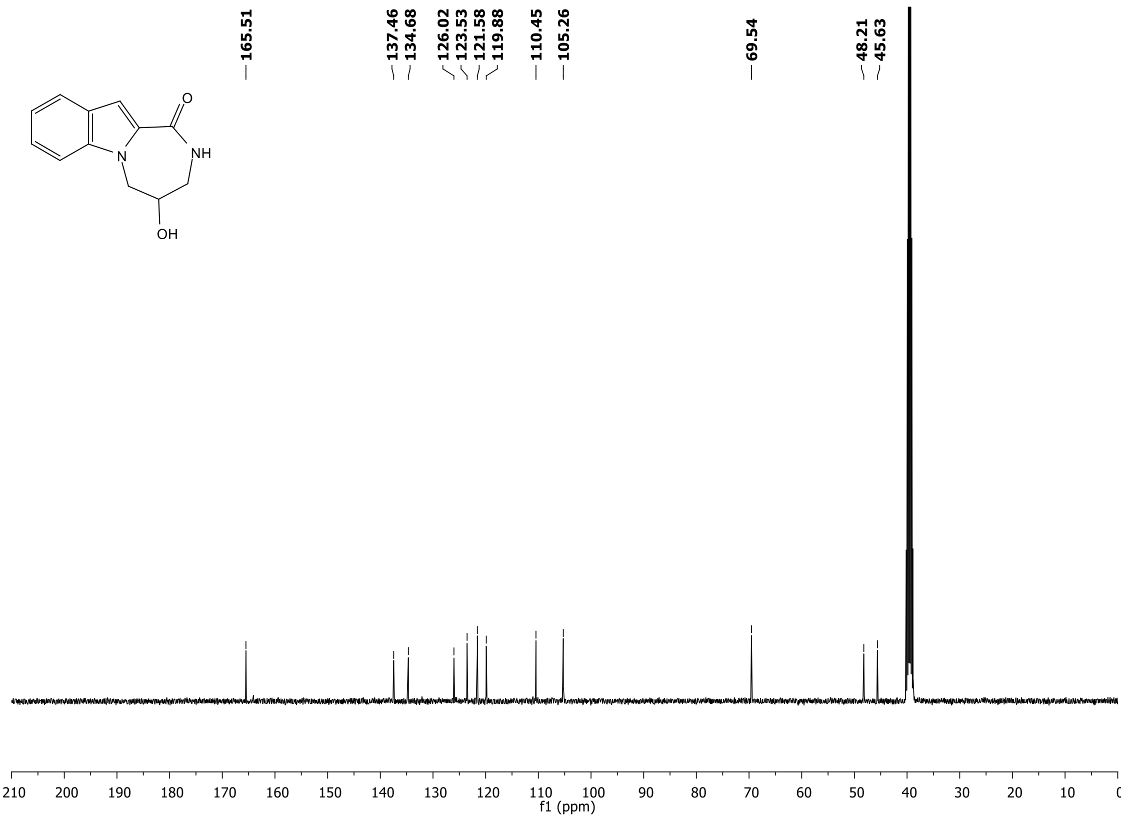

Figure S172. ^{13}C NMR spectrum (101 MHz, DMSO-d_6) of 4-hydroxy-2,3,4,5-tetrahydro-1*H*-[1,4]diazepino[1,2-*a*]indol-1-one (**7a**).

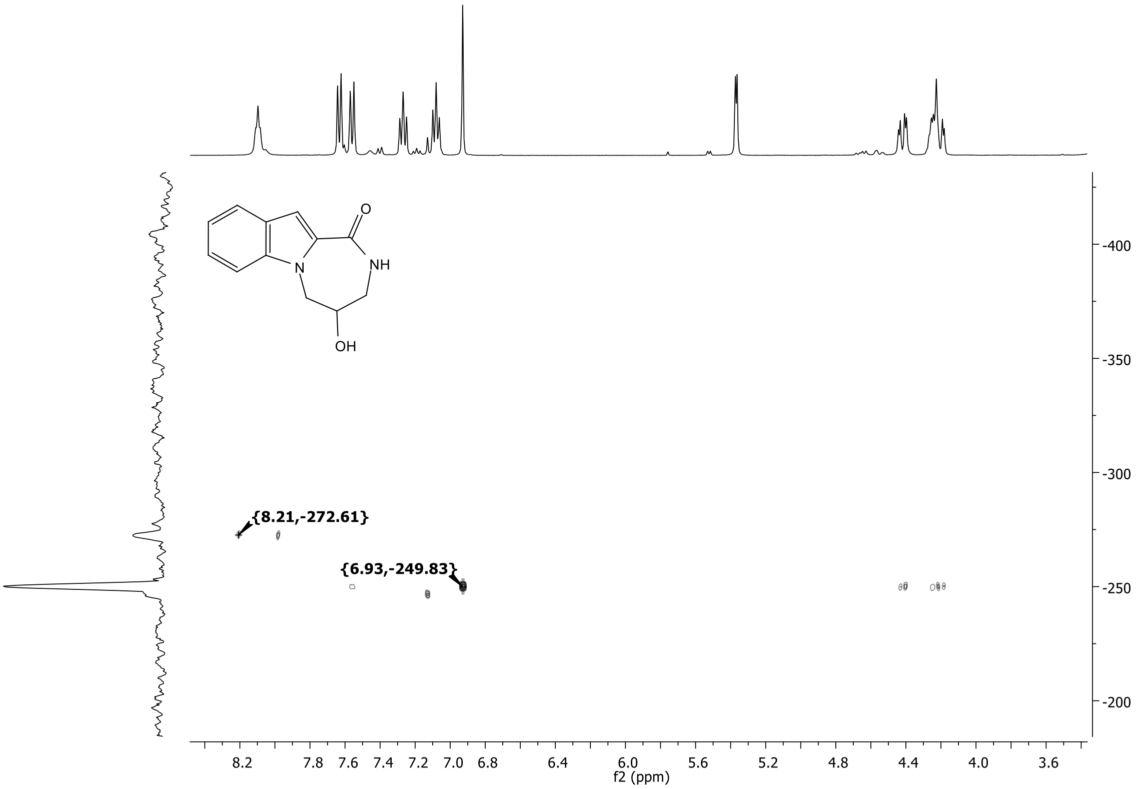

Figure S173. $^{1}H,^{15}N$-HMBC spectrum (40 MHz, DMSO-d_6) of 4-hydroxy-2,3,4,5-tetrahydro-1*H*-[1,4]diazepino[1,2-*a*]indol-1-one (**7a**).

Qualitative Compound Report

**Data File**	221111_MV-54_01.d	**Sample Name**	MV-54
**Sample Type**	Sample	**Position**	
**Instrument Name**	G6230B TOF	**User Name**	
**Acq Method**	HRMS_12min_ref.m	**Acquired Time**	11-Nov-22 11:34:53 AM
**IRM Calibration Status**	Success	**DA Method**	test.m
**Comment**			
**Sample Group**		**Info.**	
**Stream Name**		**Acquisition SW Version**	6200 series TOF/6500 series Q-TOF B.09.00 (B9044.1 SP1)

Compound Table

Compound Label	RT	Mass	Abund	Formula	Tgt Mass	Diff (ppm)
Cpd 1: C12 H12 N2 O2	6.669	216.0899	2118759	C12 H12 N2 O2	216.0899	-0.07

Compound Label	*m/z*	RT	Algorithm	Mass
Cpd 1: C12 H12 N2 O2	217.0969	6.669	Find By Formula	216.0899

Compound Chromatograms

MS Spectrum

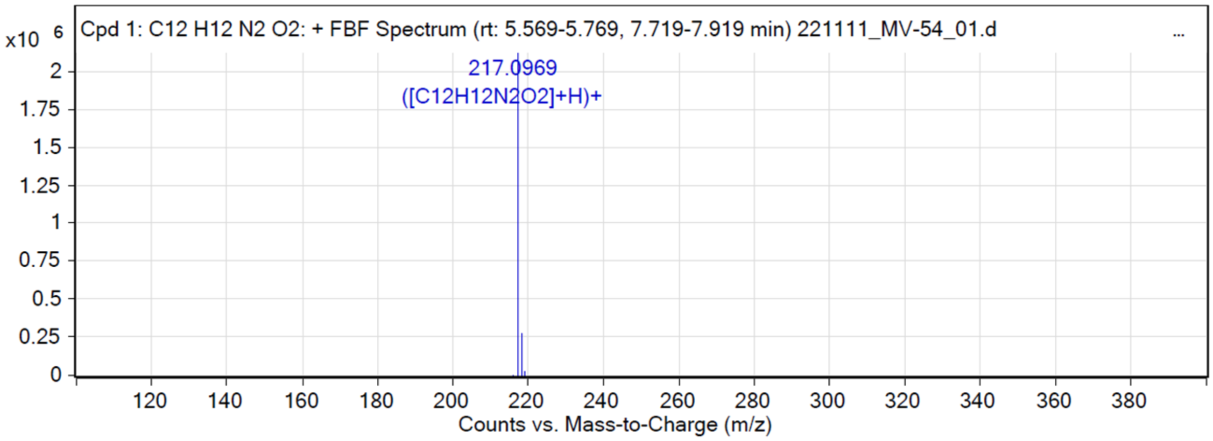

Figure S174. HRMS (ESI-TOF) spectrum of 4-hydroxy-2,3,4,5-tetrahydro-1*H*-[1,4]diazepino[1,2-*a*]indol-1-one (**7a**).

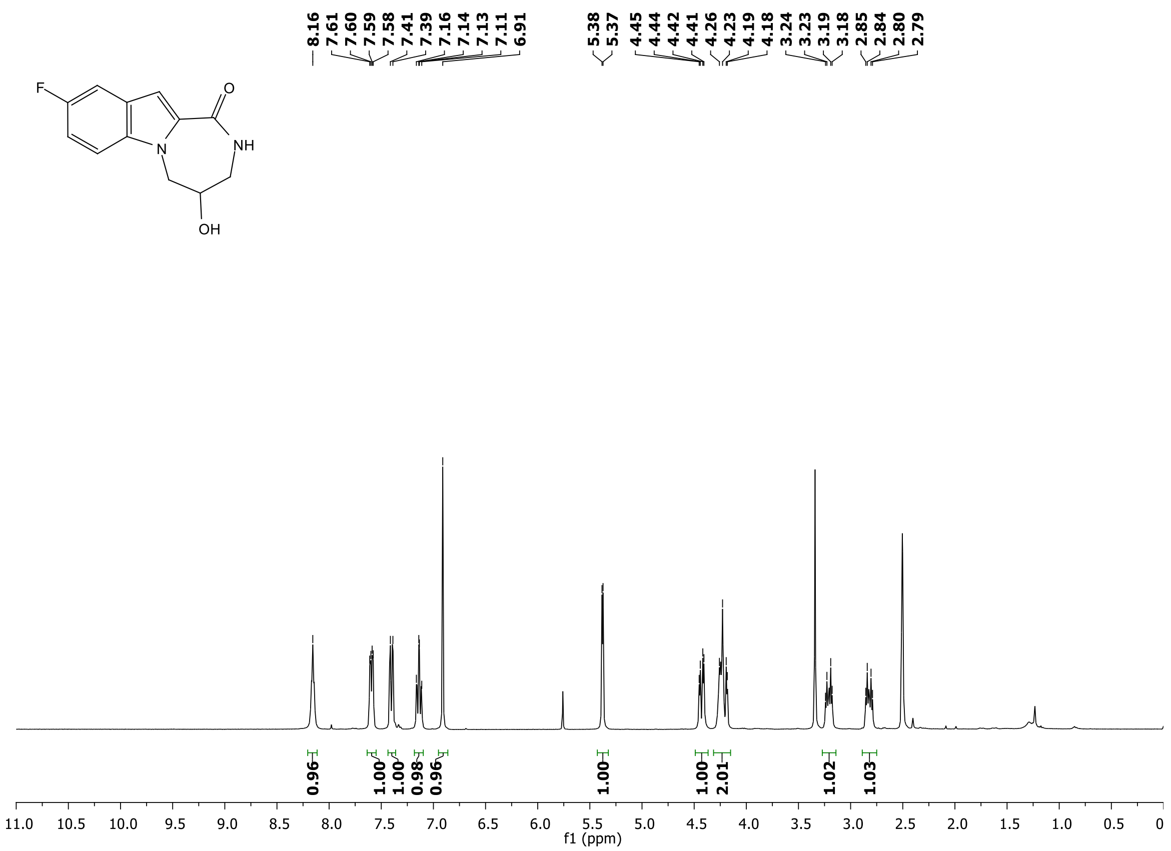

Figure S175. ^{1}H NMR spectrum (400 MHz, DMSO-d_6) of 9-fluoro-4-hydroxy-2,3,4,5-tetrahydro-1*H*-[1,4]diazepino[1,2-*a*]indol-1-one (**7b**).

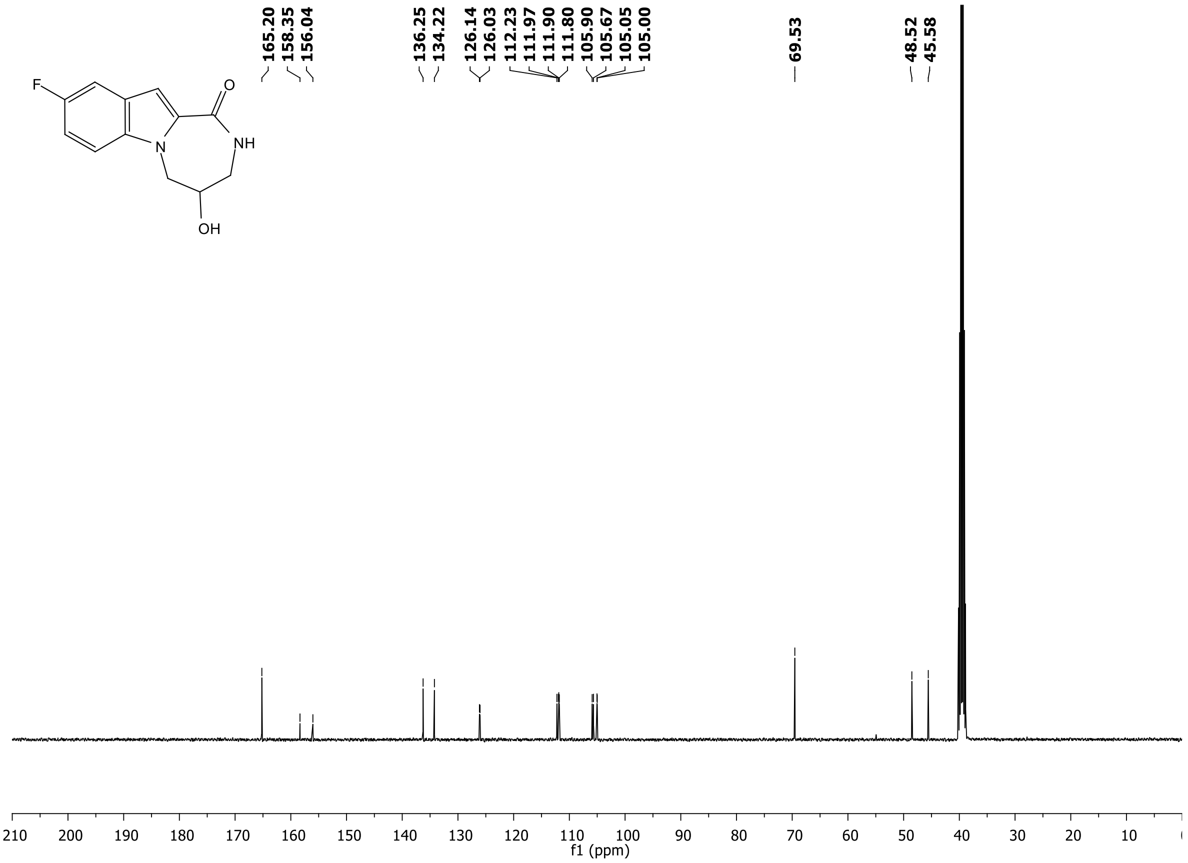

Figure S176. ^{13}C NMR spectrum (101 MHz, DMSO-d_6) of 9-fluoro-4-hydroxy-2,3,4,5-tetrahydro-1*H*-[1,4]diazepino[1,2-*a*]indol-1-one (**7b**).

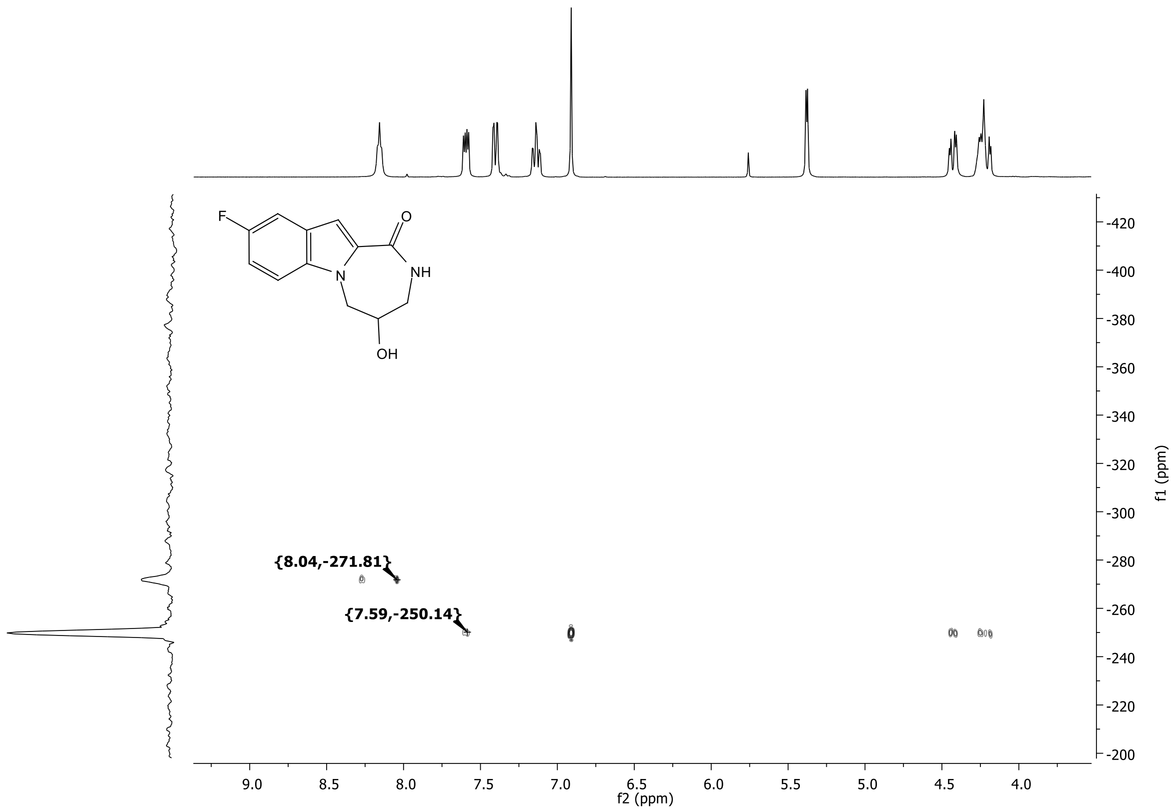

Figure S177. ^{1}H,^{15}N-HMBC spectrum (40 MHz, DMSO-*d*$_6$) of 9-fluoro-4-hydroxy-2,3,4,5-tetrahydro-1*H*-[1,4]diazepino[1,2-*a*]indol-1-one (**7b**).

Qualitative Compound Report

**Data File**	221111_MV-62-2_01.d	**Sample Name**	MV-62-2
**Sample Type**	Sample	**Position**	
**Instrument Name**	G6230B TOF	**User Name**	
**Acq Method**	HRMS_12min_ref.m	**Acquired Time**	11-Nov-22 1:59:37 PM
**IRM Calibration Status**	Success	**DA Method**	test.m
**Comment**			
**Sample Group**		**Info.**	
**Stream Name**		**Acquisition SW Version**	6200 series TOF/6500 series Q-TOF B.09.00 (B9044.1 SP1)

Compound Table

Compound Label	RT	Mass	Abund	Formula	Tgt Mass	Diff (ppm)
Cpd 1: C12 H11 F N2 O2	6.659	234.0804	883246	C12 H11 F N2 O2	234.0805	-0.05

Compound Label	m/z	RT	Algorithm	Mass
Cpd 1: C12 H11 F N2 O2	235.0876	6.659	Find By Formula	234.0804

Compound Chromatograms

MS Spectrum

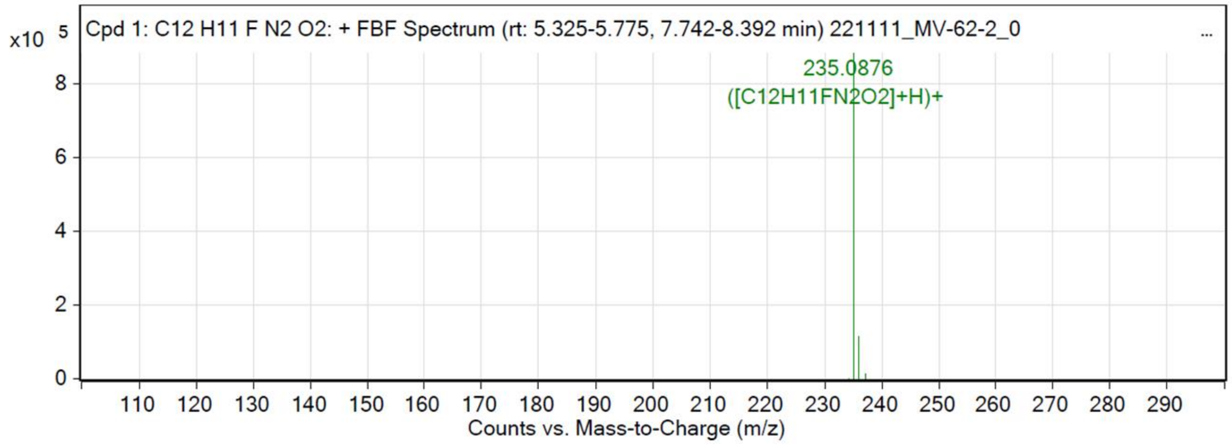

Figure S178. HRMS (ESI-TOF) spectrum of 9-fluoro-4-hydroxy-2,3,4,5-tetrahydro-1*H*-[1,4]diazepino[1,2-*a*]indol-1-one (**7b**).

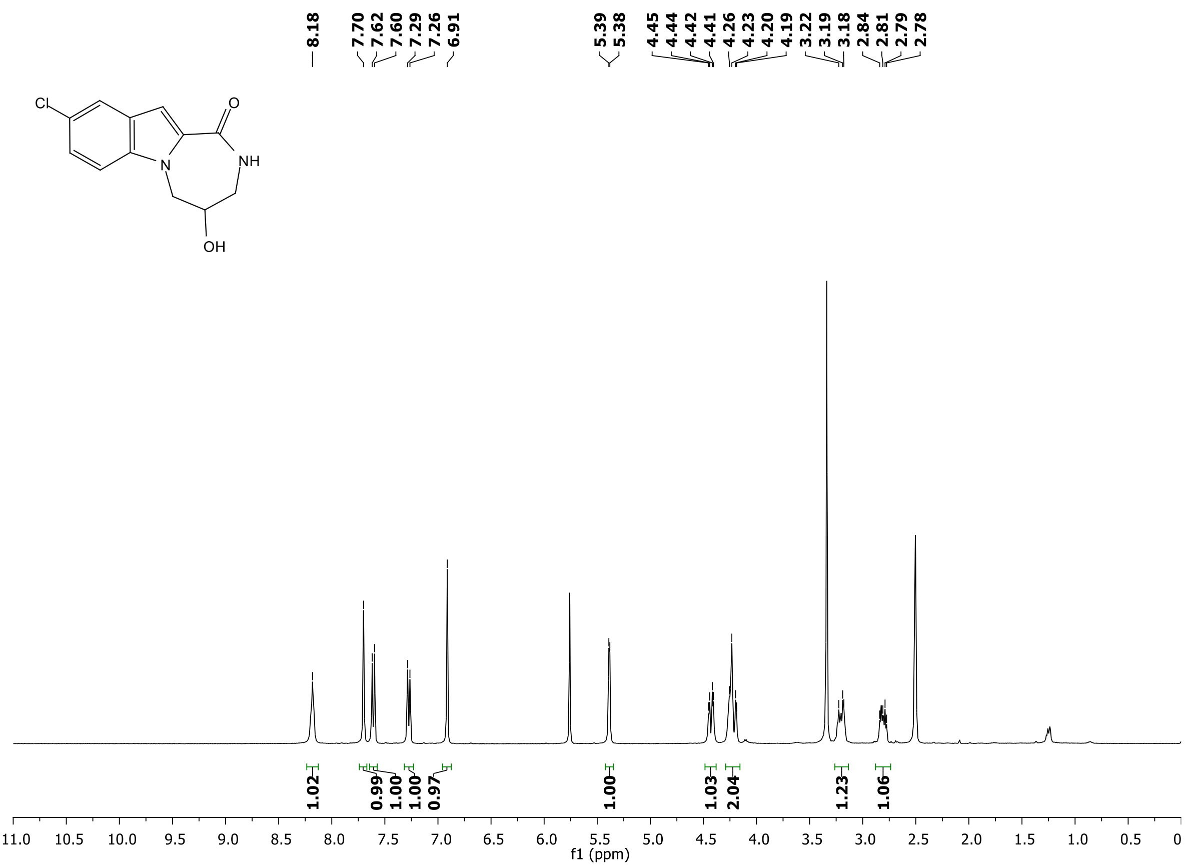

Figure S179. ^{1}H NMR spectrum (400 MHz, DMSO-*d*$_6$) of 9-chloro-4-hydroxy-2,3,4,5-tetrahydro-1*H*-[1,4]diazepino[1,2-*a*]indol-1-one (**7c**).

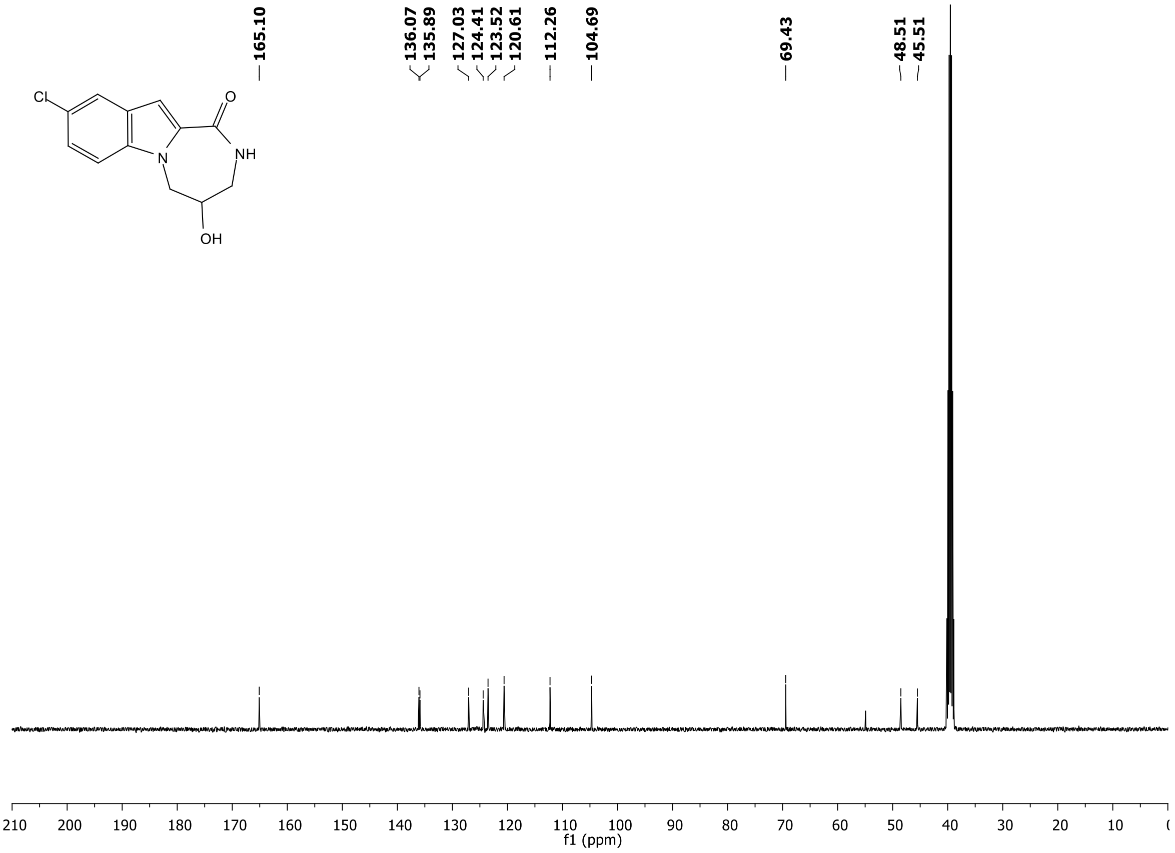

Figure S180. ^{13}C NMR spectrum (101 MHz, DMSO-*d*$_6$) of 9-chloro-4-hydroxy-2,3,4,5-tetrahydro-1*H*-[1,4]diazepino[1,2-*a*]indol-1-one (**7c**).

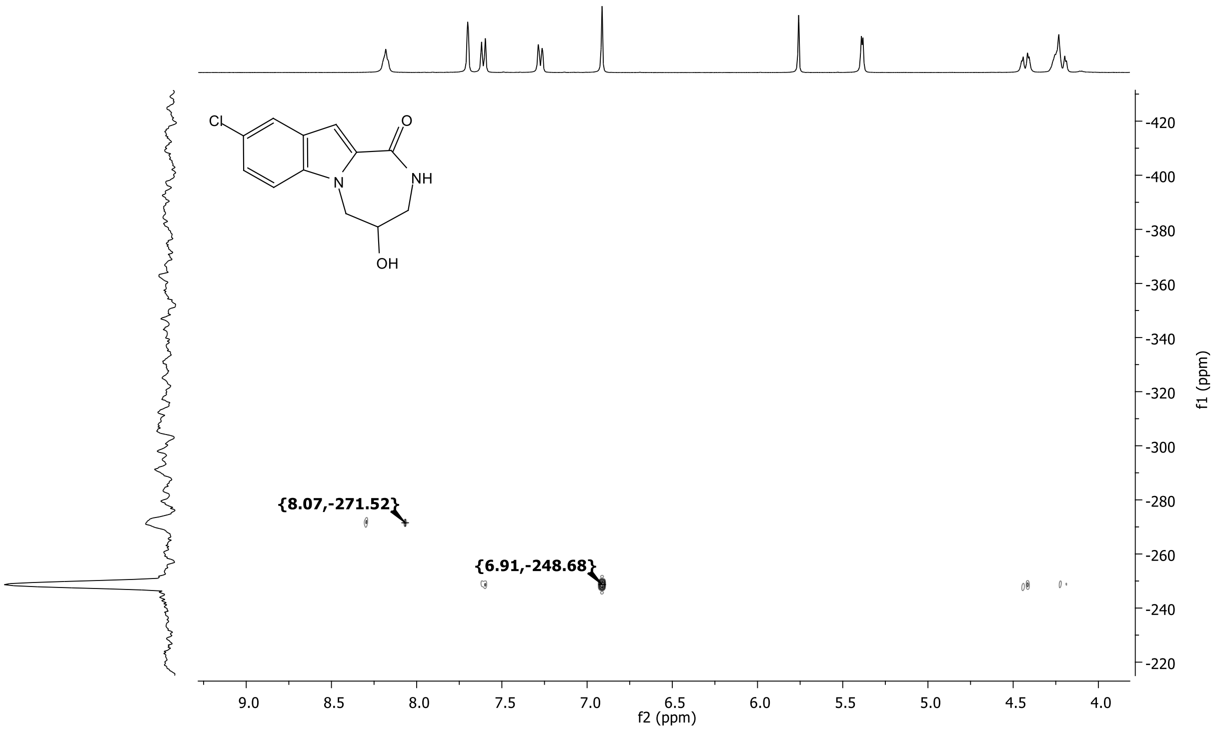

Figure S181. $^{1}H,^{15}N$-HMBC spectrum (40 MHz, DMSO-d_6) of 9-chloro-4-hydroxy-2,3,4,5-tetrahydro-1*H*-[1,4]diazepino[1,2-*a*]indol-1-one (**7c**).

Qualitative Compound Report

**Data File**	221111_MV-61-1_01.d	**Sample Name**	MV-61-1
**Sample Type**	Sample	**Position**	
**Instrument Name**	G6230B TOF	**User Name**	
**Acq Method**	HRMS_12min_ref.m	**Acquired Time**	11-Nov-22 1:49:24 PM
**IRM Calibration Status**	Success	**DA Method**	test.m
**Comment**			
**Sample Group**		**Info.**	
**Stream Name**		**Acquisition SW Version**	6200 series TOF/6500 series Q-TOF B.09.00 (B9044.1 SP1)

Compound Table

Compound Label	RT	Mass	Abund	Formula	Tgt Mass	Diff (ppm)
Cpd 1: C12 H11 Cl N2 O2	6.702	250.051	909078	C12 H11 Cl N2 O2	250.0509	0.34

Compound Label	*m/z*	RT	Algorithm	Mass
Cpd 1: C12 H11 Cl N2 O2	251.058	6.702	Find By Formula	250.051

Compound Chromatograms

MS Spectrum

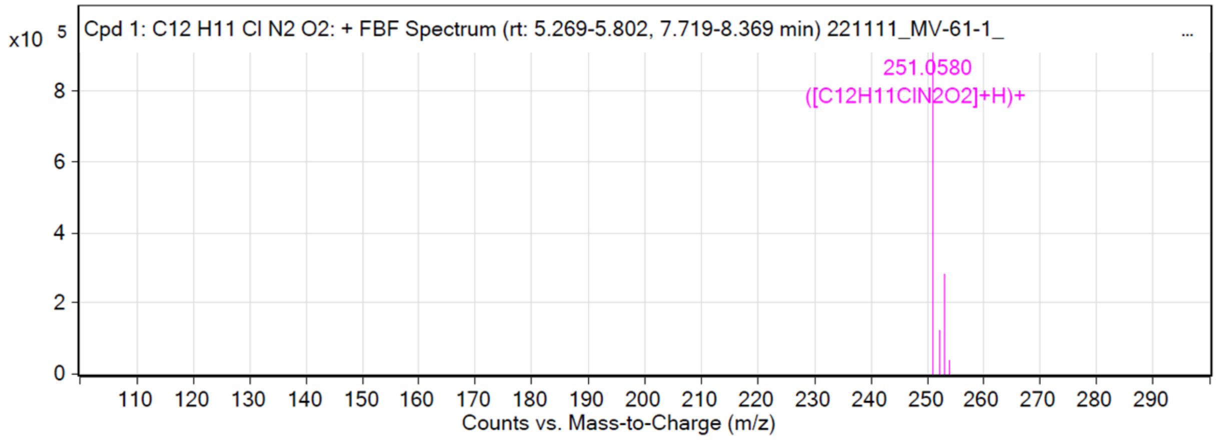

Figure S182. HRMS (ESI-TOF) spectrum of 9-chloro-4-hydroxy-2,3,4,5-tetrahydro-1*H*-[1,4]diazepino[1,2-*a*]indol-1-one (**7c**).

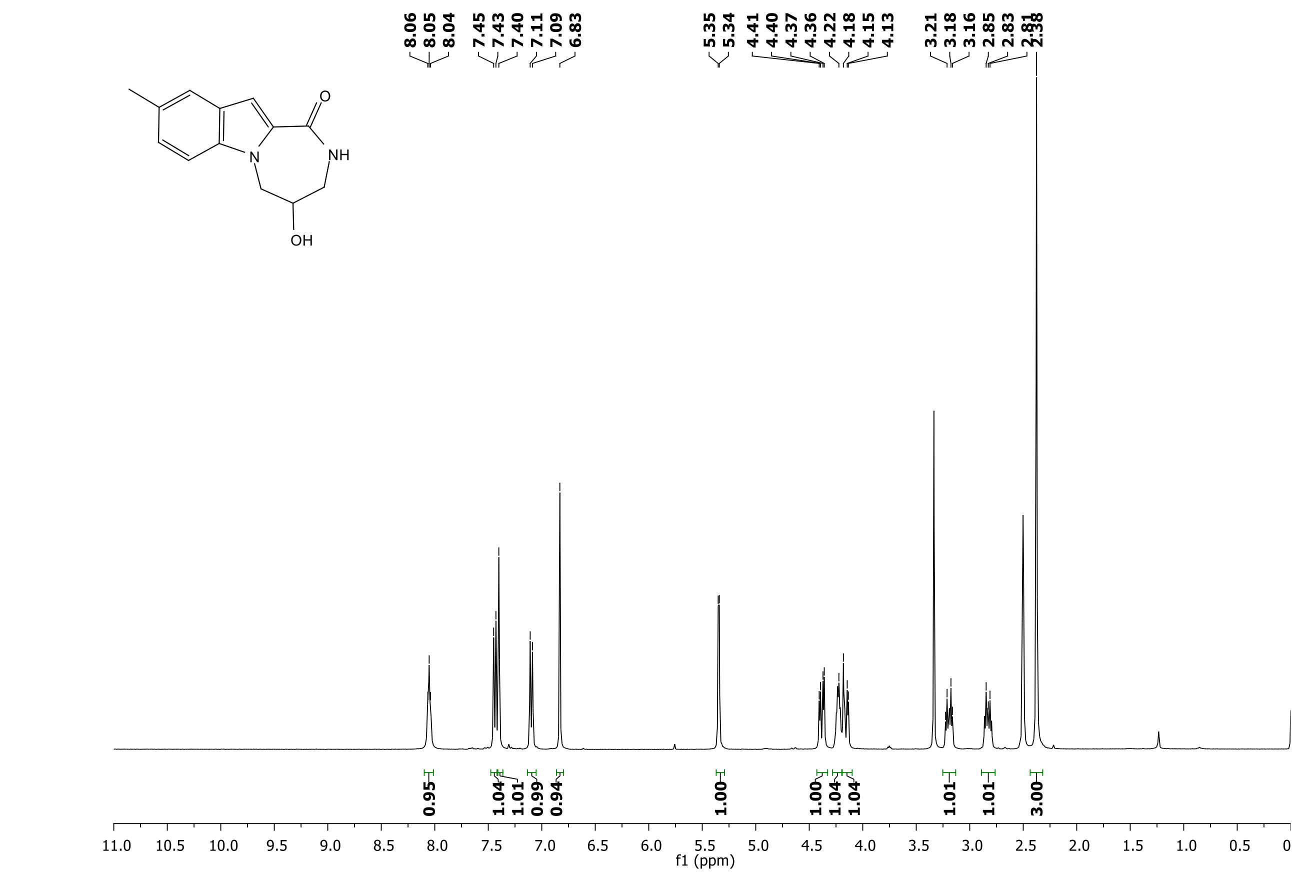

Figure S183. ^{1}H NMR spectrum (400 MHz, DMSO-d_6) of 4-hydroxy-9-methyl-2,3,4,5-tetrahydro-1*H*-[1,4]diazepino[1,2-*a*]indol-1-one (**7d**).

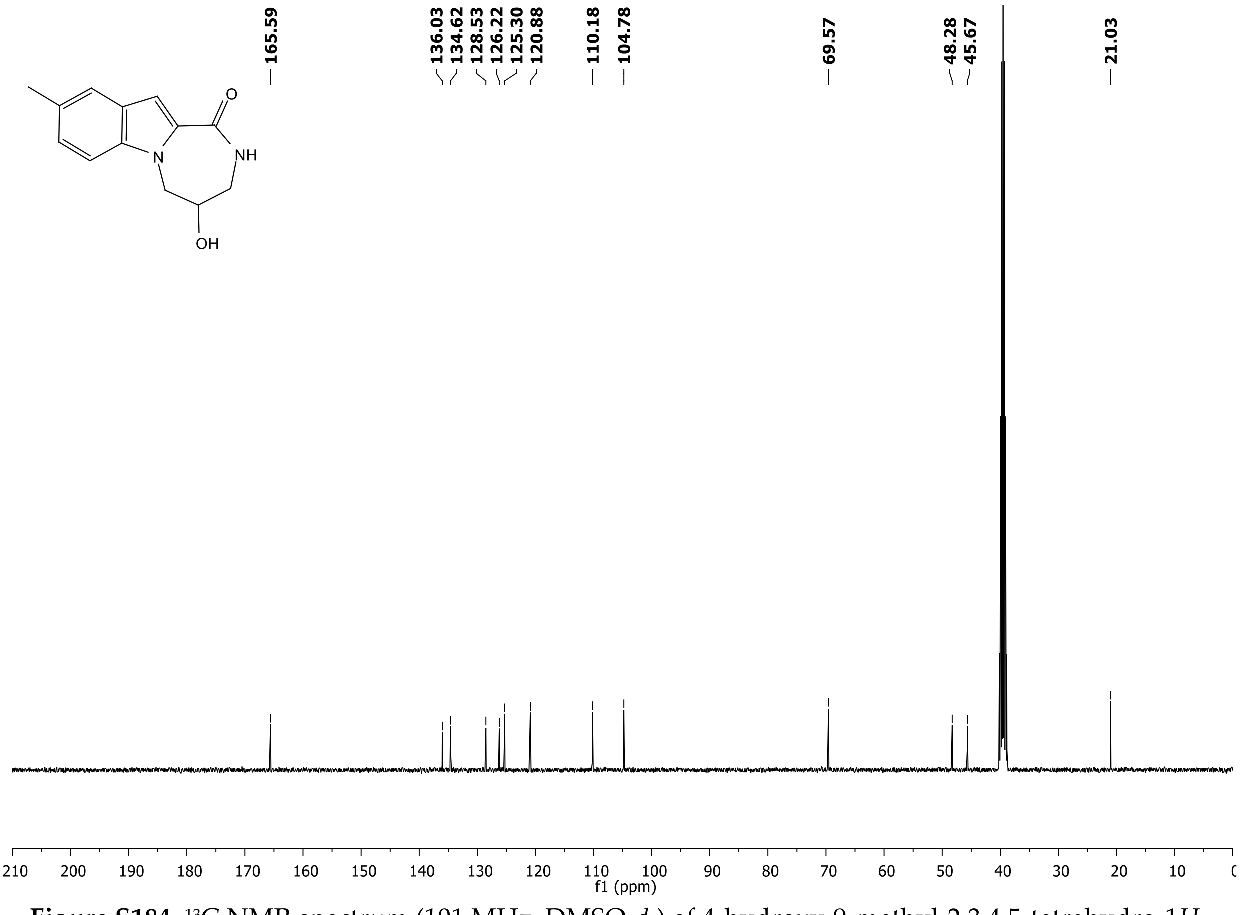

Figure S184. ^{13}C NMR spectrum (101 MHz, DMSO-d_6) of 4-hydroxy-9-methyl-2,3,4,5-tetrahydro-1*H*-[1,4]diazepino[1,2-*a*]indol-1-one (**7d**).

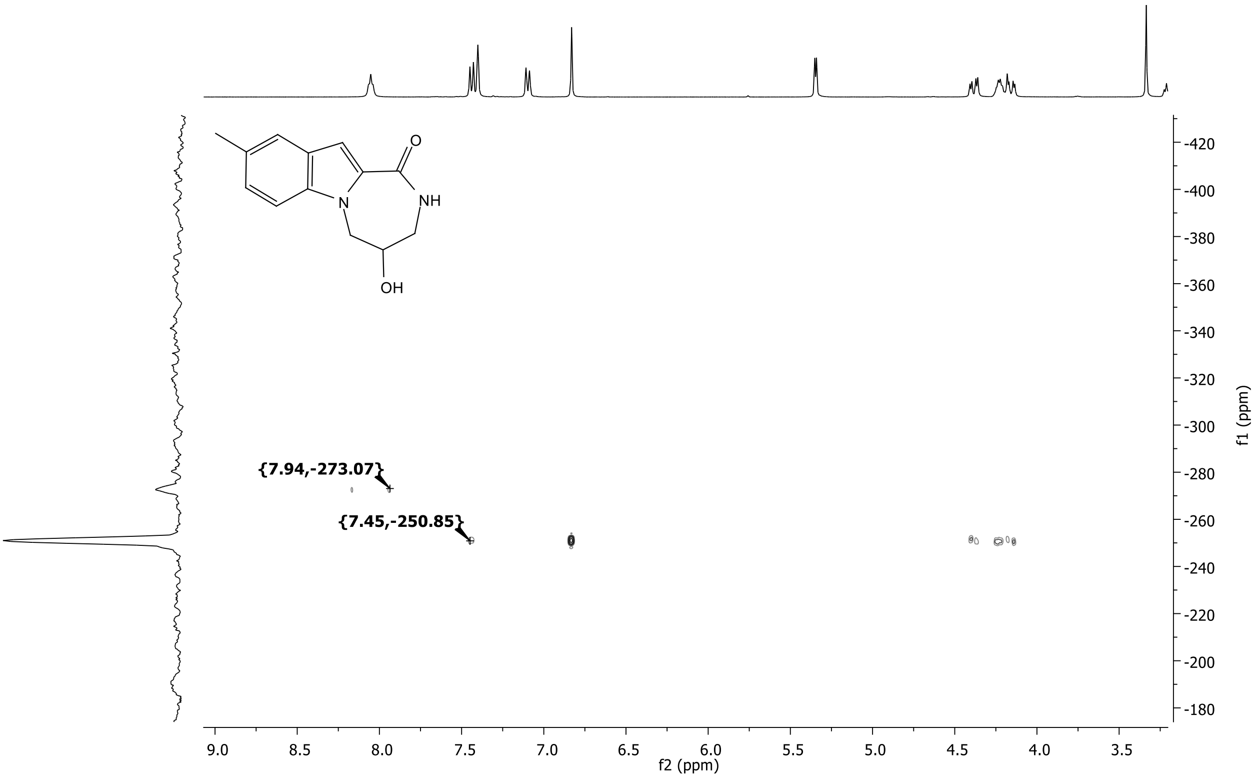

Figure S185. $^{1}H,^{15}N$-HMBC spectrum (40 MHz, DMSO-*d*$_6$) of 4-hydroxy-9-methyl-2,3,4,5-tetrahydro-1*H*-[1,4]diazepino[1,2-*a*]indol-1-one (**7d**).

Qualitative Compound Report

Data File	221111_MV-64-1_01.d	Sample Name	MV-64-1
Sample Type	Sample	Position	
Instrument Name	G6230B TOF	User Name	
Acq Method	HRMS_12min_ref.m	Acquired Time	11-Nov-22 12:28:41 PM
IRM Calibration Status	Success	DA Method	test.m
Comment			
Sample Group		Info.	
Stream Name		Acquisition SW Version	6200 series TOF/6500 series Q-TOF B.09.00 (B9044.1 SP1)

Compound Table

Compound Label	RT	Mass	Abund	Formula	Tgt Mass	Diff (ppm)
Cpd 1: C13 H14 N2 O2	6.64	230.1053	964422	C13 H14 N2 O2	230.1055	-1.02

Compound Label	m/z	RT	Algorithm	Mass
Cpd 1: C13 H14 N2 O2	231.1124	6.64	Find By Formula	230.1053

Compound Chromatograms

MS Spectrum

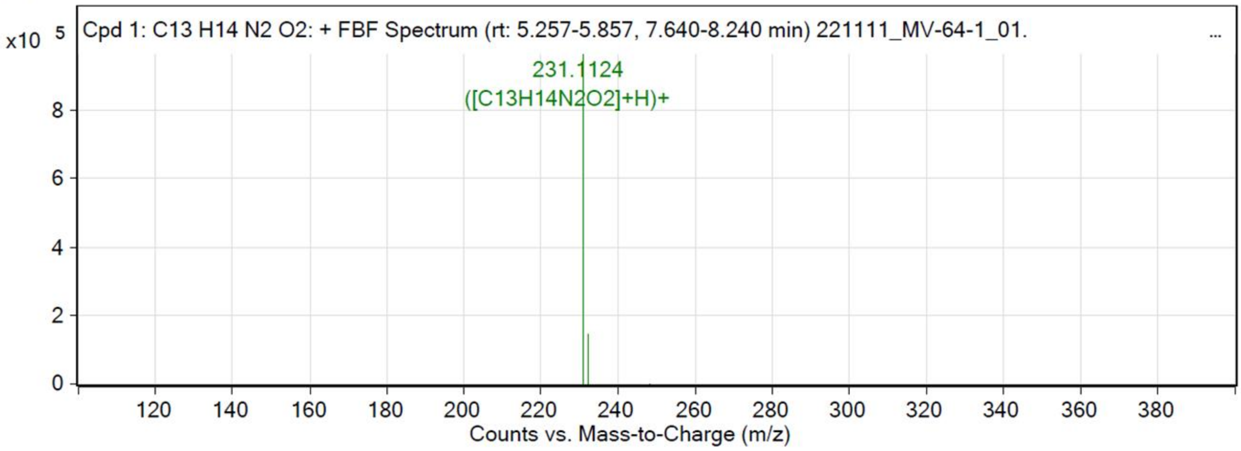

Figure S186. HRMS (ESI-TOF) spectrum of 4-hydroxy-9-methyl-2,3,4,5-tetrahydro-1*H*-[1,4]diazepino[1,2-*a*]indol-1-one (**7d**).

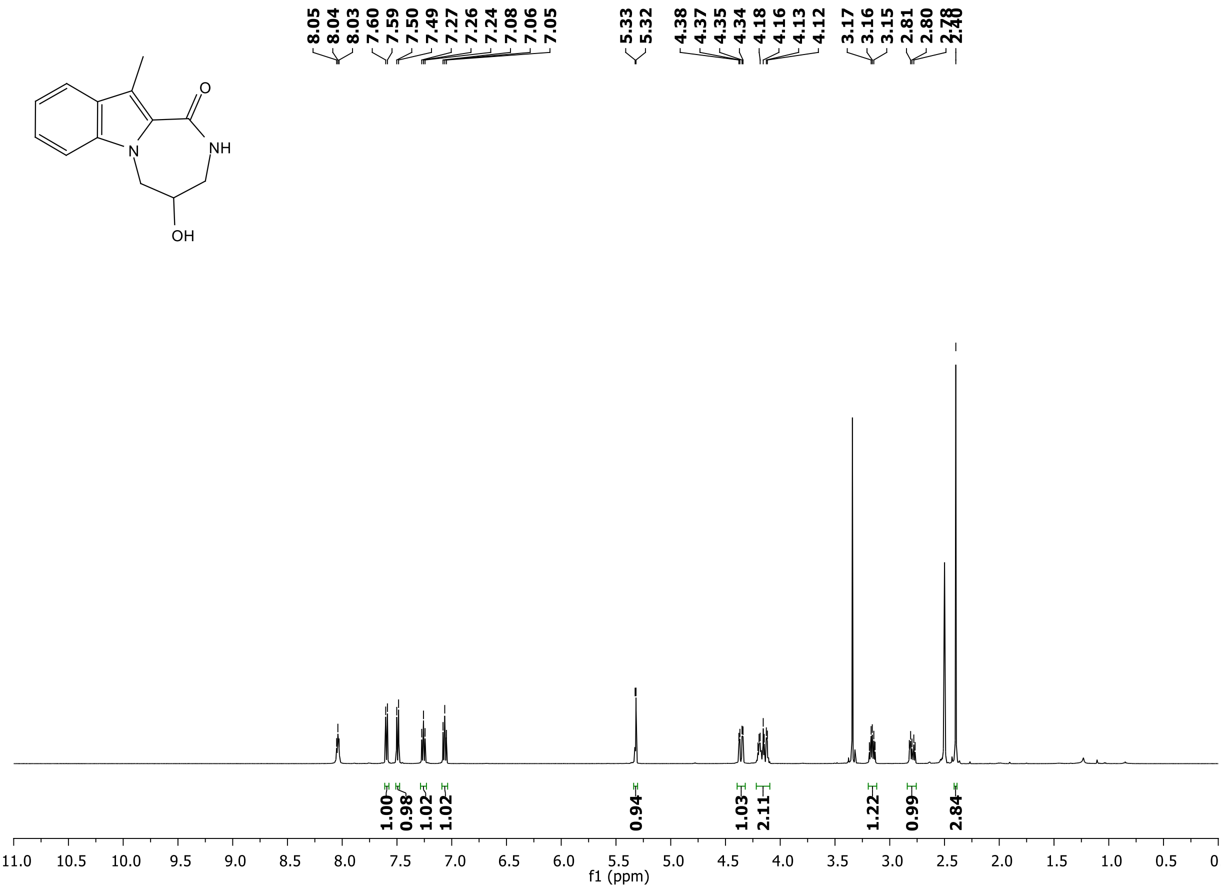

Figure S187. ^{1}H NMR spectrum (500 MHz, DMSO-d_6) of 4-hydroxy-11-methyl-2,3,4,5-tetrahydro-1*H*-[1,4]diazepino[1,2-*a*]indol-1-one (**7e**).

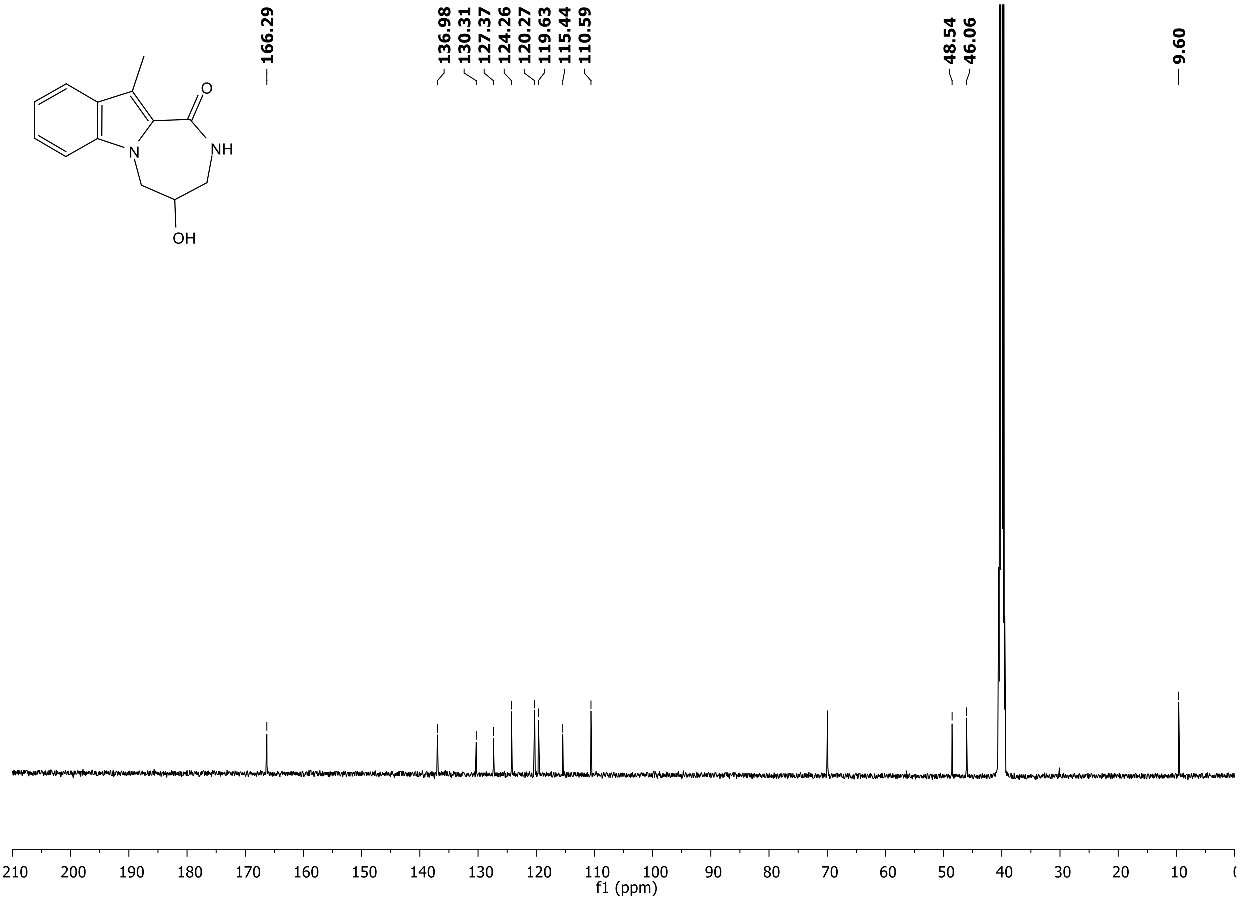

Figure S188. ^{13}C NMR spectrum (125 MHz, DMSO-d_6) of 4-hydroxy-11-methyl-2,3,4,5-tetrahydro-1*H*-[1,4]diazepino[1,2-*a*]indol-1-one (**7e**).

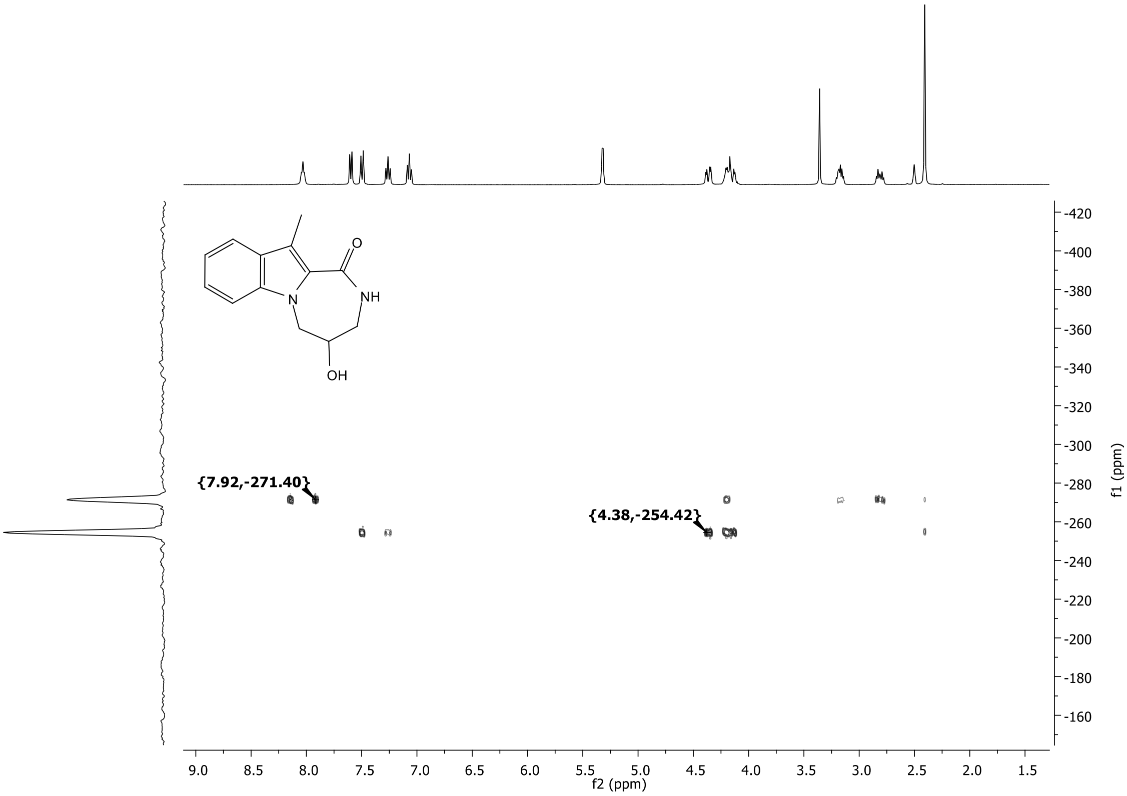

Figure S189. ^{1}H,^{15}N-HMBC spectrum (40 MHz, DMSO-d_6) of 4-hydroxy-11-methyl-2,3,4,5-tetrahydro-1*H*-[1,4]diazepino[1,2-*a*]indol-1-one (**7e**).

Qualitative Compound Report

**Data File**	220720_VMO-epi3b_03.d	**Sample Name**	VMO-epi3b
**Sample Type**	Sample	**Position**	
**Instrument Name**	G6230B TOF	**User Name**	
**Acq Method**	HRMS_12min_ref.m	**Acquired Time**	20-Jul-22 10:05:35 AM
**IRM Calibration Status**	Success	**DA Method**	test.m
**Comment**			
**Sample Group**		**Info.**	
**Stream Name**		**Acquisition SW Version**	6200 series TOF/6500 series Q-TOF B.09.00 (B9044.1 SP1)

Compound Table

Compound Label	RT	Mass	Abund	Formula	Tgt Mass	Diff (ppm)
Cpd 1: C13 H14 N2 O2	2.432	230.1056	113726	C13 H14 N2 O2	230.1055	0.14

Compound Label	*m/z*	RT	Algorithm	Mass
Cpd 1: C13 H14 N2 O2	483.2007	2.432	Find By Formula	230.1056

Compound Chromatograms

MS Spectrum

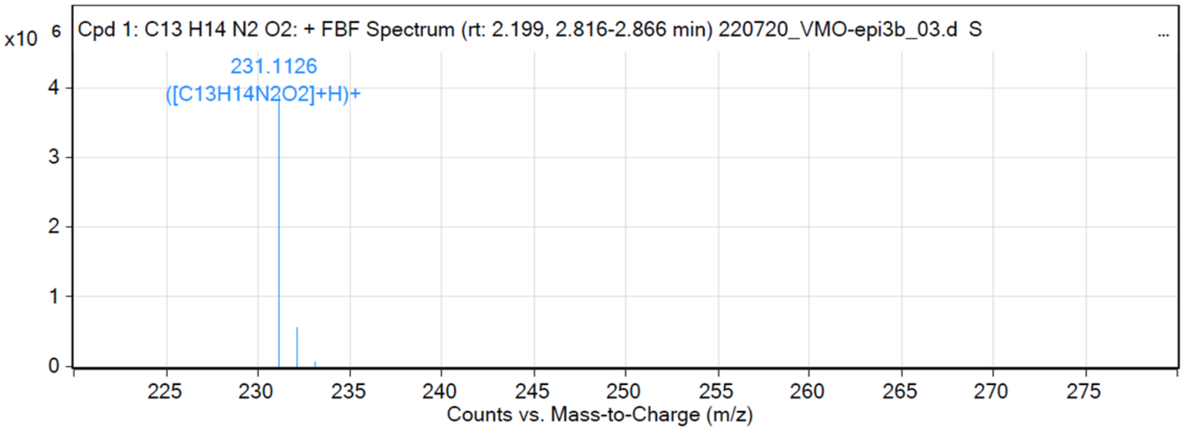

Figure S190. HRMS (ESI-TOF) spectrum of 4-hydroxy-11-methyl-2,3,4,5-tetrahydro-1*H*-[1,4]diazepino[1,2-*a*]indol-1-one (**7e**).

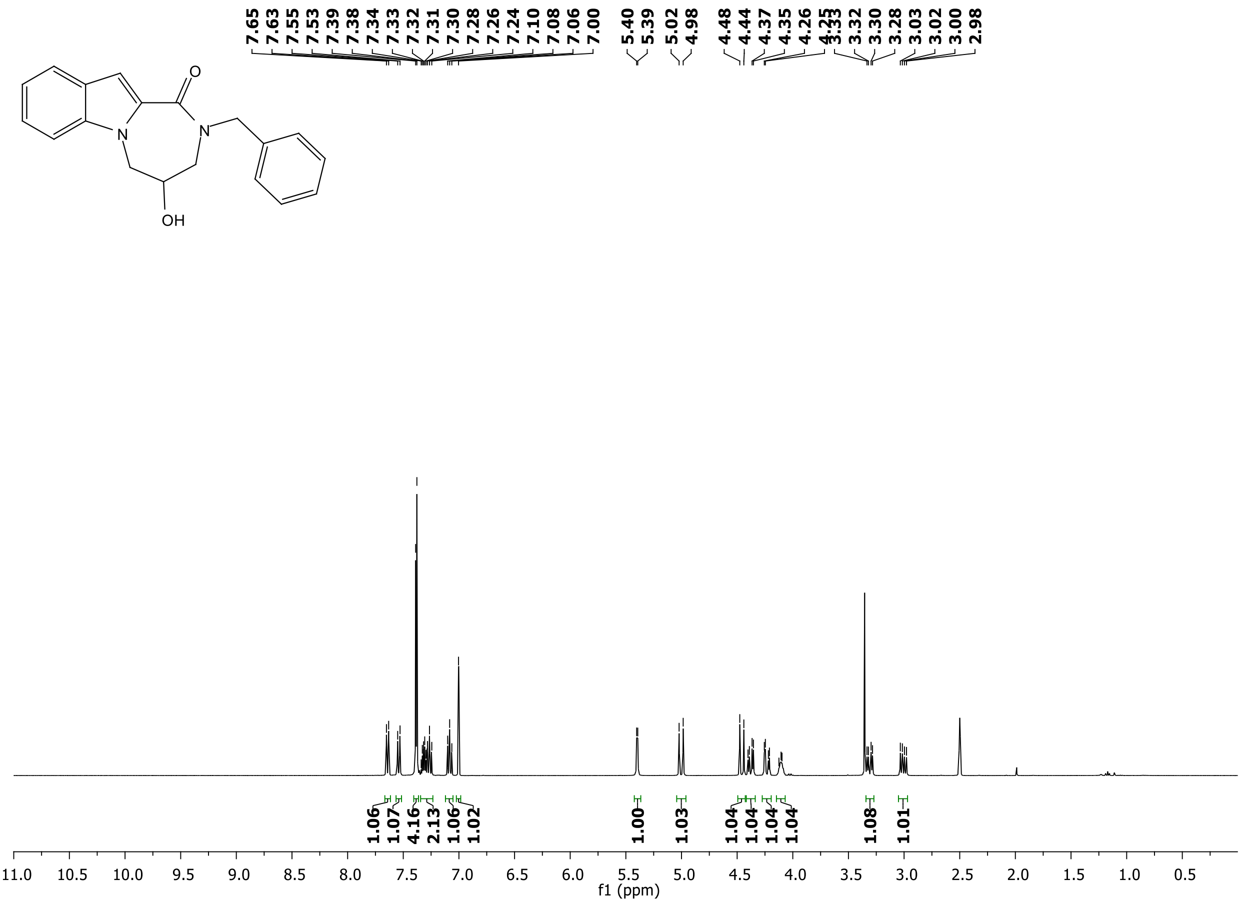

Figure S191. ^{1}H NMR spectrum (400 MHz, DMSO-d_6) of 2-benzyl-4-hydroxy-2,3,4,5-tetrahydro-1*H*-[1,4]diazepino[1,2-*a*]indol-1-one (**7f**).

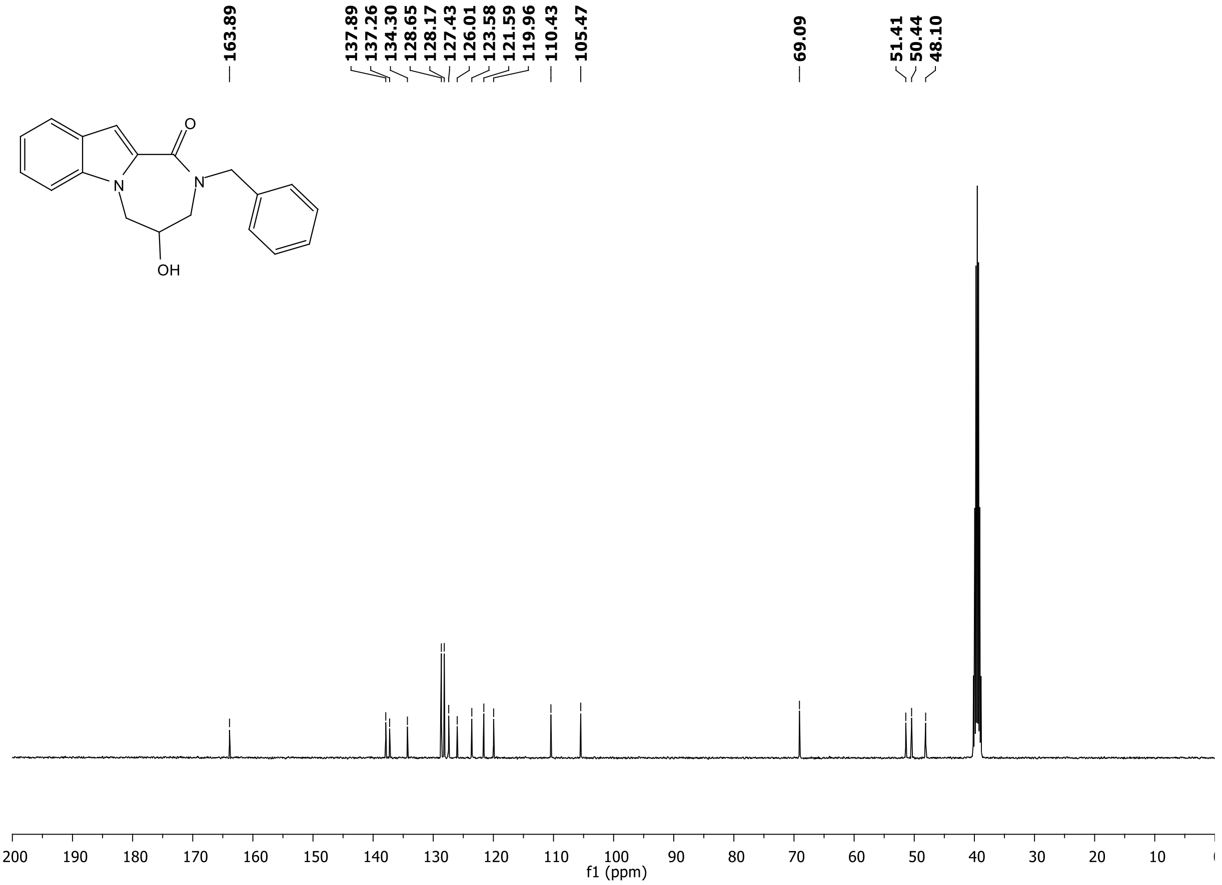

Figure S192. ^{13}C NMR spectrum (101 MHz, DMSO-d_6) of 2-benzyl-4-hydroxy-2,3,4,5-tetrahydro-1*H*-[1,4]diazepino[1,2-*a*]indol-1-one (**7f**).

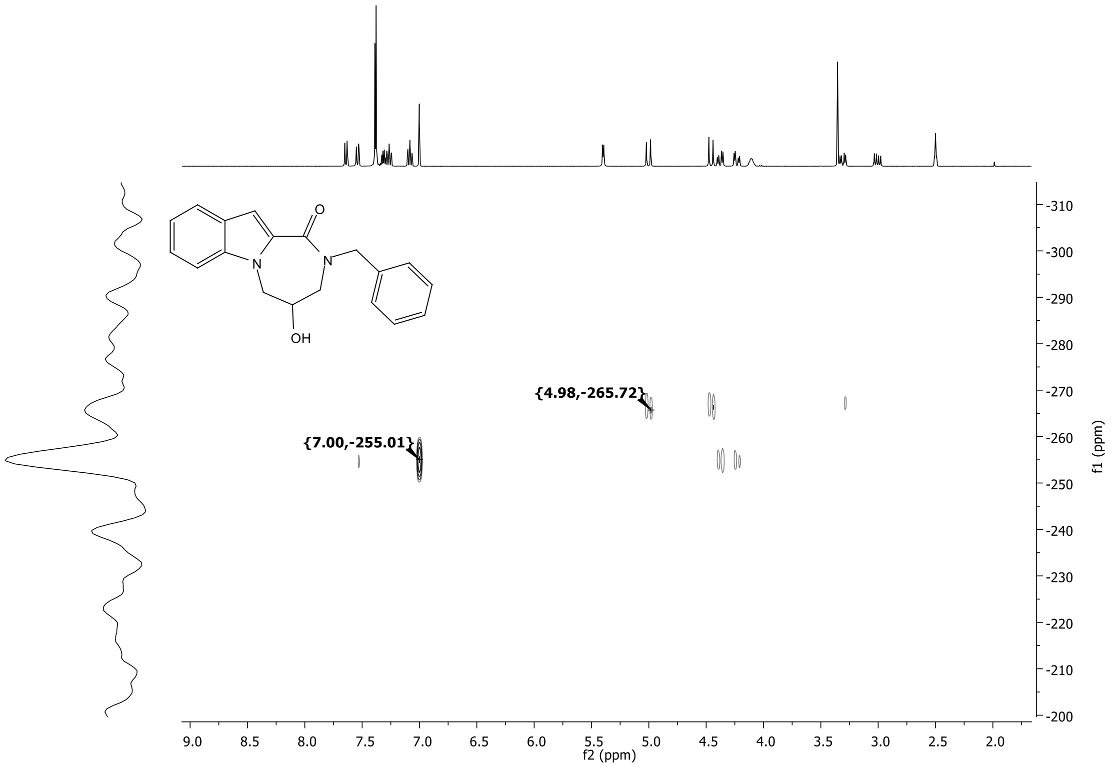

Figure S193. $^{1}H,^{15}N$-HMBC spectrum (40 MHz, DMSO-d_6) of 2-benzyl-4-hydroxy-2,3,4,5-tetrahydro-1*H*-[1,4]diazepino[1,2-*a*]indol-1-one (**7f**).

Qualitative Compound Report

**Data File**	221202_KDUP-019-1_02.d	**Sample Name**	Unavailable
**Sample Type**	Unavailable	**Position**	Unavailable
**Instrument Name**	Unavailable	**User Name**	Unavailable
**Acq Method**		**Acquired Time**	Unavailable
**IRM Calibration Status**	Success	**DA Method**	test.m
**Comment**	Sample information is unavailable		

Compound Table

Compound Label	RT	Mass	Abund	Formula	Tgt Mass	Diff (ppm)
Cpd 1: C19 H18 N2 O2	11.115	306.1378	258729	C19 H18 N2 O2	306.1368	3.25

Compound Label	*m/z*	RT	Algorithm	Mass
Cpd 1: C19 H18 N2 O2	613.2823	11.115	Find By Formula	306.1378

Compound Chromatograms

MS Spectrum

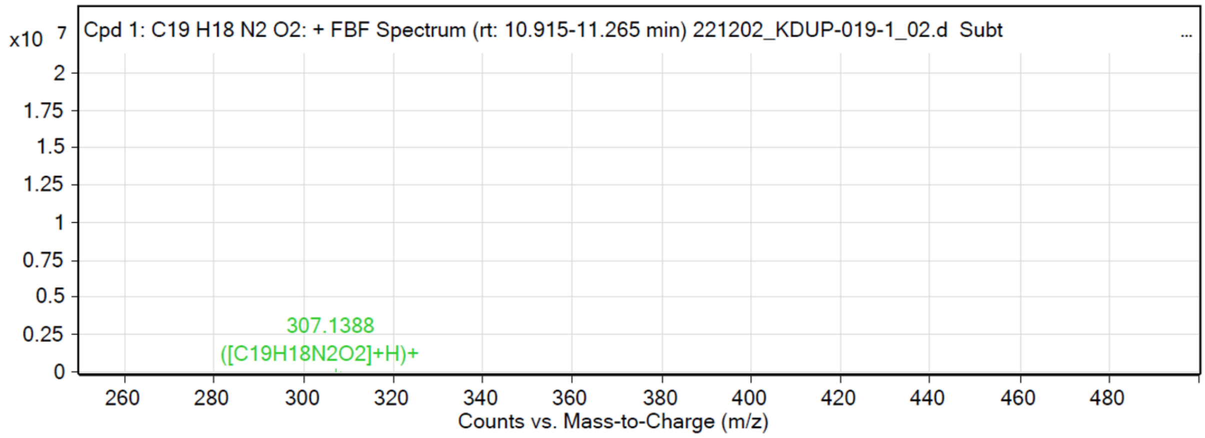

Figure S194. HRMS (ESI-TOF) spectrum of 2-benzyl-4-hydroxy-2,3,4,5-tetrahydro-1*H*-[1,4]diazepino[1,2-*a*]indol-1-one (**7f**).

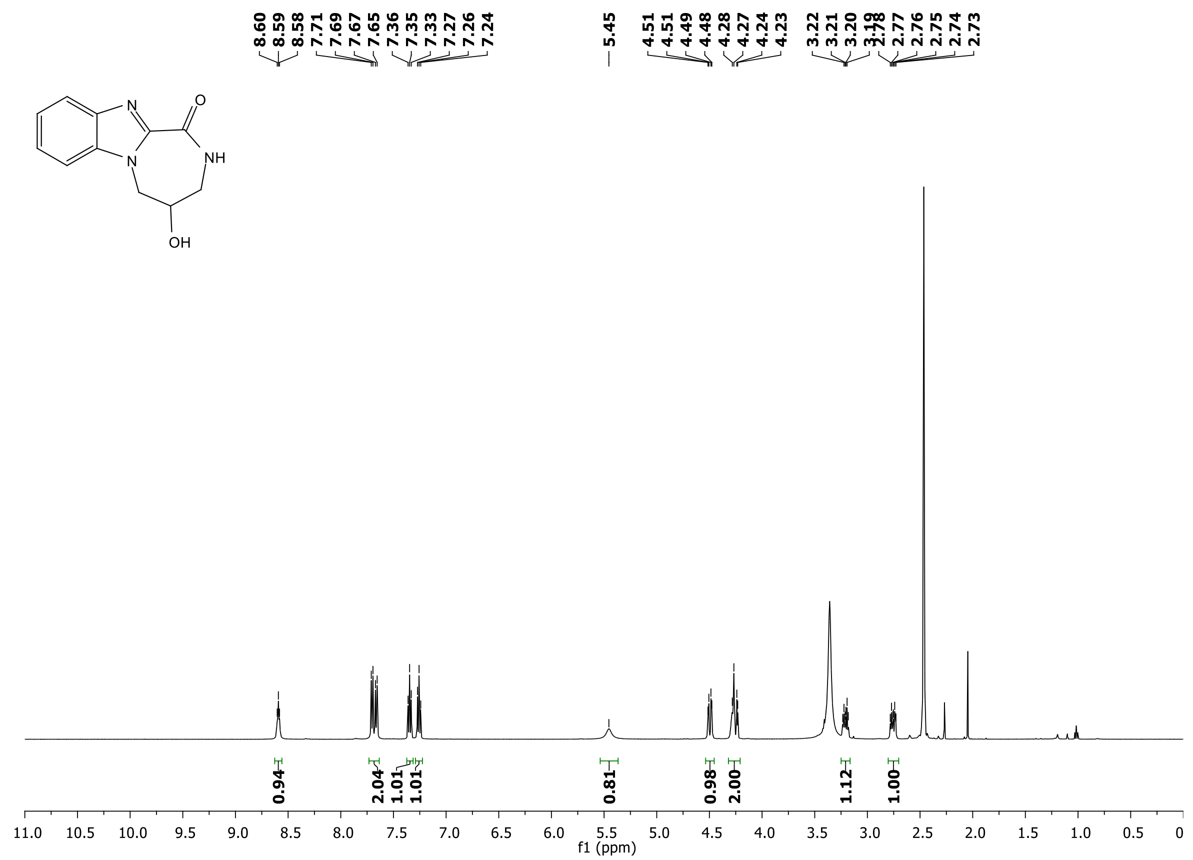

Figure S195. ^{1}H NMR spectrum (500 MHz, DMSO-*d*6) of 4-hydroxy-2,3,4,5-tetrahydro-1*H*-benzo[4,5]imidazo[1,2-*a*][1,4]diazepin-1-one (**7g**).

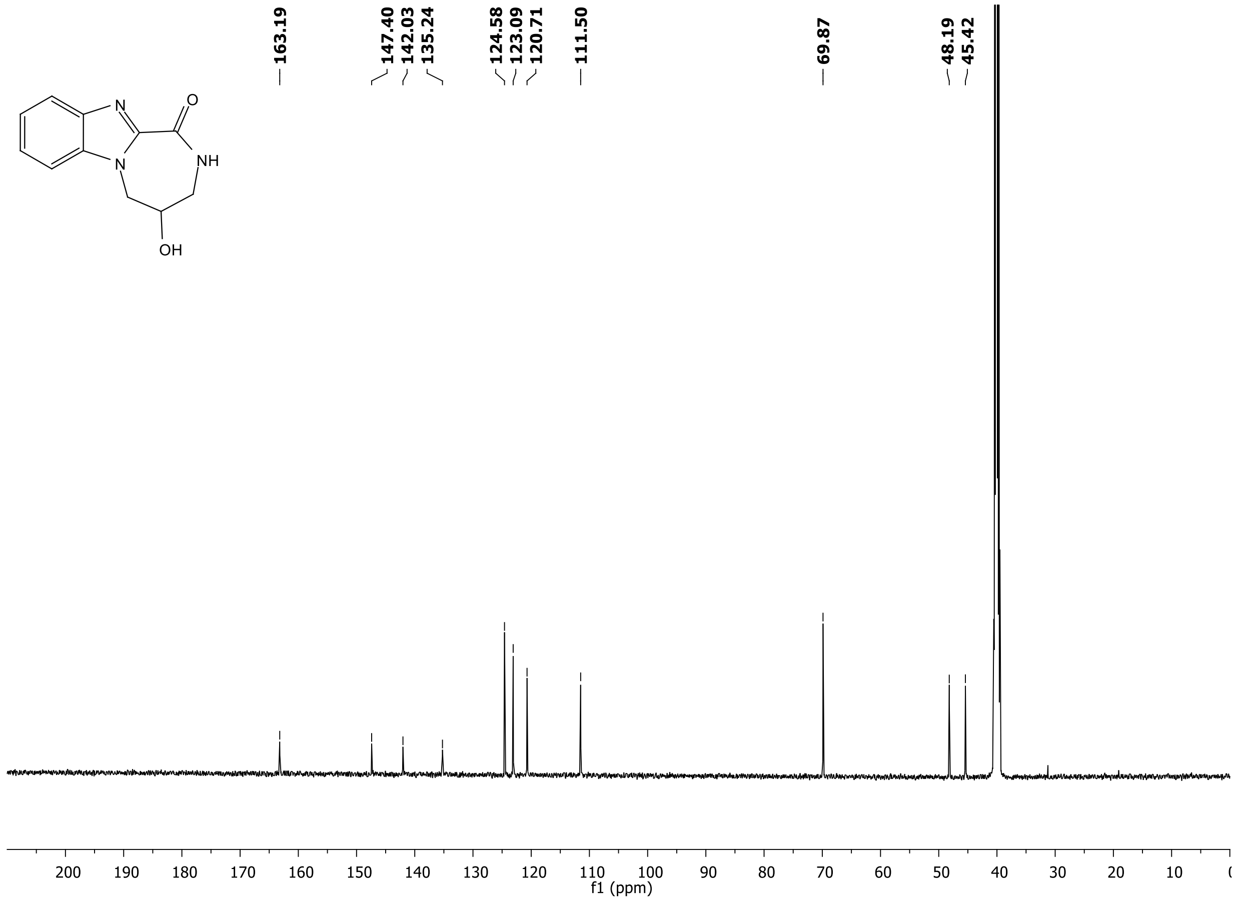

Figure S196. ^{13}C NMR spectrum (125 MHz, DMSO-*d*6) of 4-hydroxy-2,3,4,5-tetrahydro-1*H*-benzo[4,5]imidazo[1,2-*a*][1,4]diazepin-1-one (**7g**).

Qualitative Compound Report

**Data File**	221101_VMO-051_01.d	**Sample Name**	Unavailable
**Sample Type**	Unavailable	**Position**	Unavailable
**Instrument Name**	Unavailable	**User Name**	Unavailable
**Acq Method**		**Acquired Time**	Unavailable
**IRM Calibration Status**	Success	**DA Method**	test.m
**Comment**	Sample information is unavailable		

Compound Table

Compound Label	RT	Mass	Abund	Formula	Tgt Mass	Diff (ppm)
Cpd 1: C11 H11 N3 O2	2.565	217.0849	1152171	C11 H11 N3 O2	217.0851	-0.88

Compound Label	m/z	RT	Algorithm	Mass
Cpd 1: C11 H11 N3 O2	218.0918	2.565	Find By Formula	217.0849

Compound Chromatograms

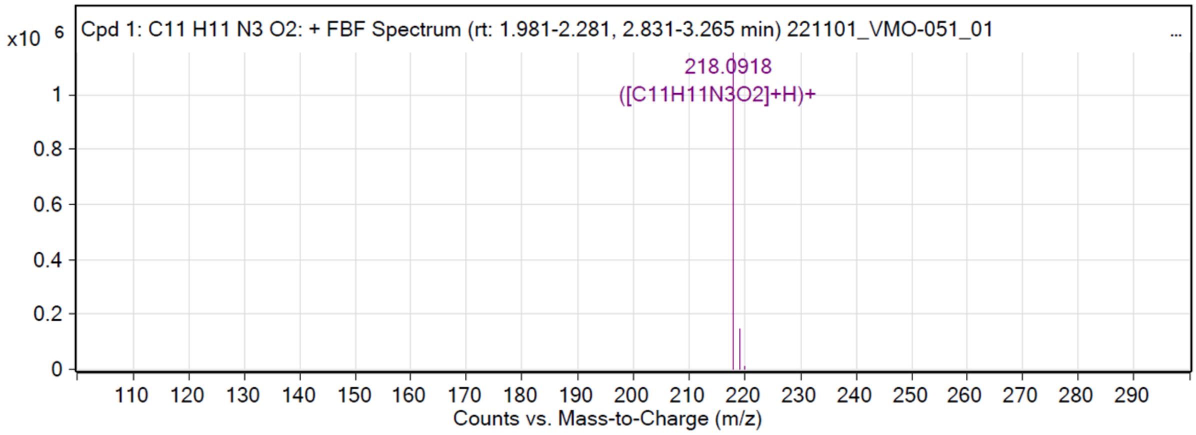

Figure S197. HRMS (ESI-TOF) spectrum of 4-hydroxy-2,3,4,5-tetrahydro-1*H*-benzo[4,5]imidazo[1,2-*a*][1,4]diazepin-1-one (**7g**).

5. Data of *O*-alkylated 5-substituted 7-hydroxy-2-phenyl-5,6,7,8-tetrahydro-4*H*-pyrazolo[1,5-*a*][1,4]diazepin-4-ones (8a–f) and 2-benzyl-4-hydroxy-2,3,4,5-tetrahydro-1*H*-[1,4]diazepino[1,2-*a*]indol-1-one (9a,b)

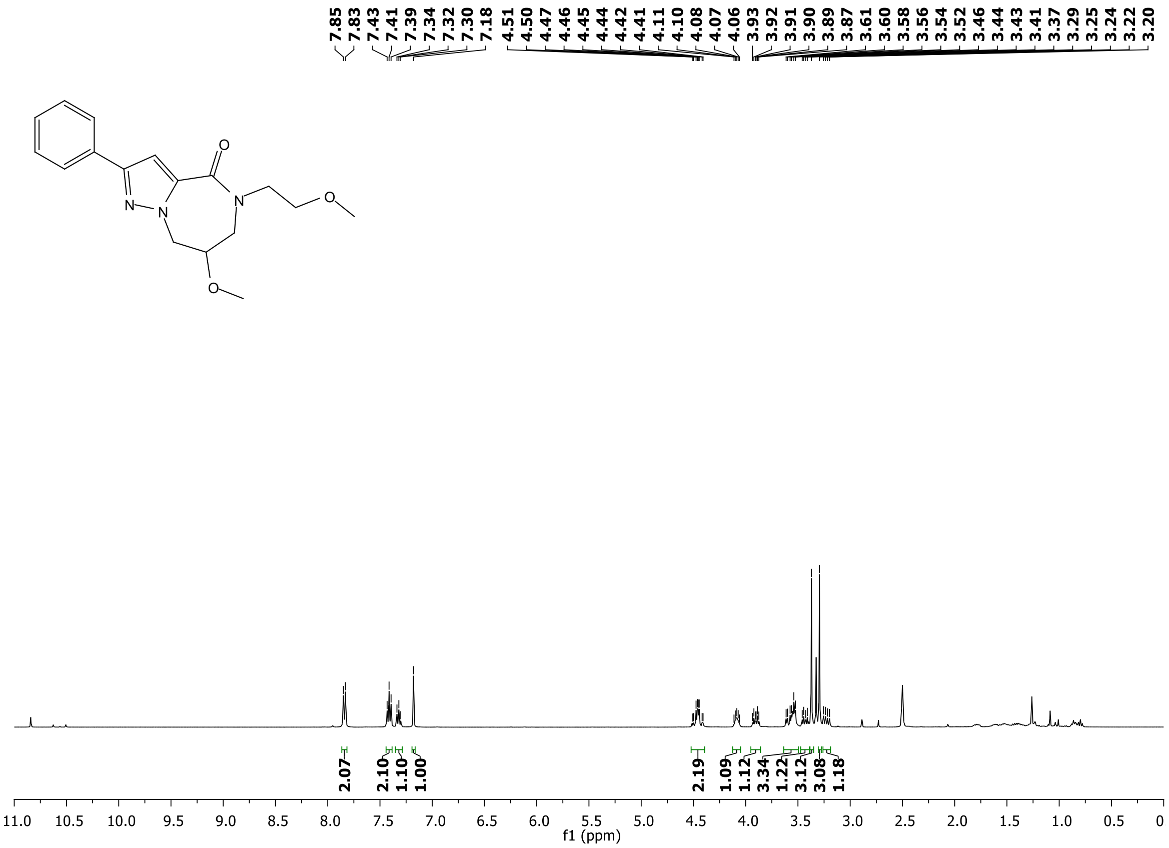

Figure S198. ^{1}H NMR spectrum (400 MHz, DMSO-d_6) of 7-methoxy-5-(2-methoxyethyl)-2-phenyl-5,6,7,8-tetrahydro-4*H*-pyrazolo[1,5-*a*][1,4]diazepin-4-one (**8a**).

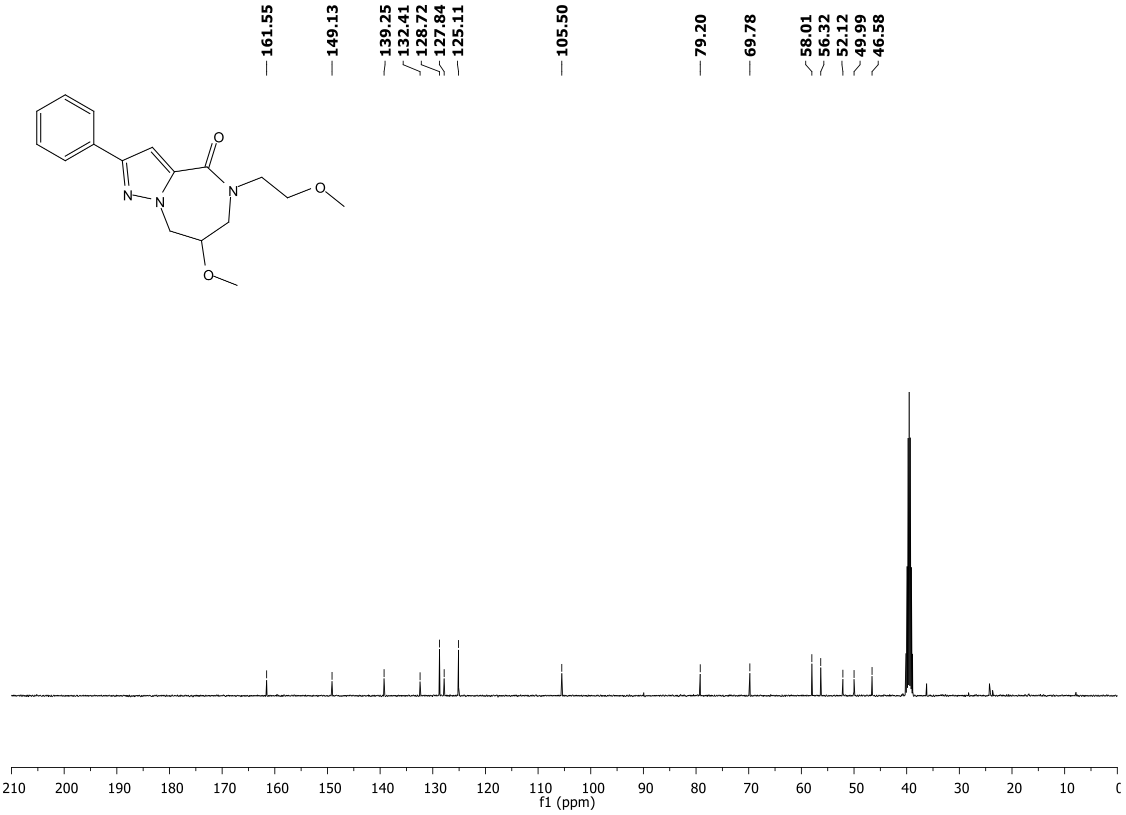

Figure S199. ^{13}C NMR spectrum (101 MHz, DMSO-d_6) of 7-methoxy-5-(2-methoxyethyl)-2-phenyl-5,6,7,8-tetrahydro-4*H*-pyrazolo[1,5-*a*][1,4]diazepin-4-one (**8a**).

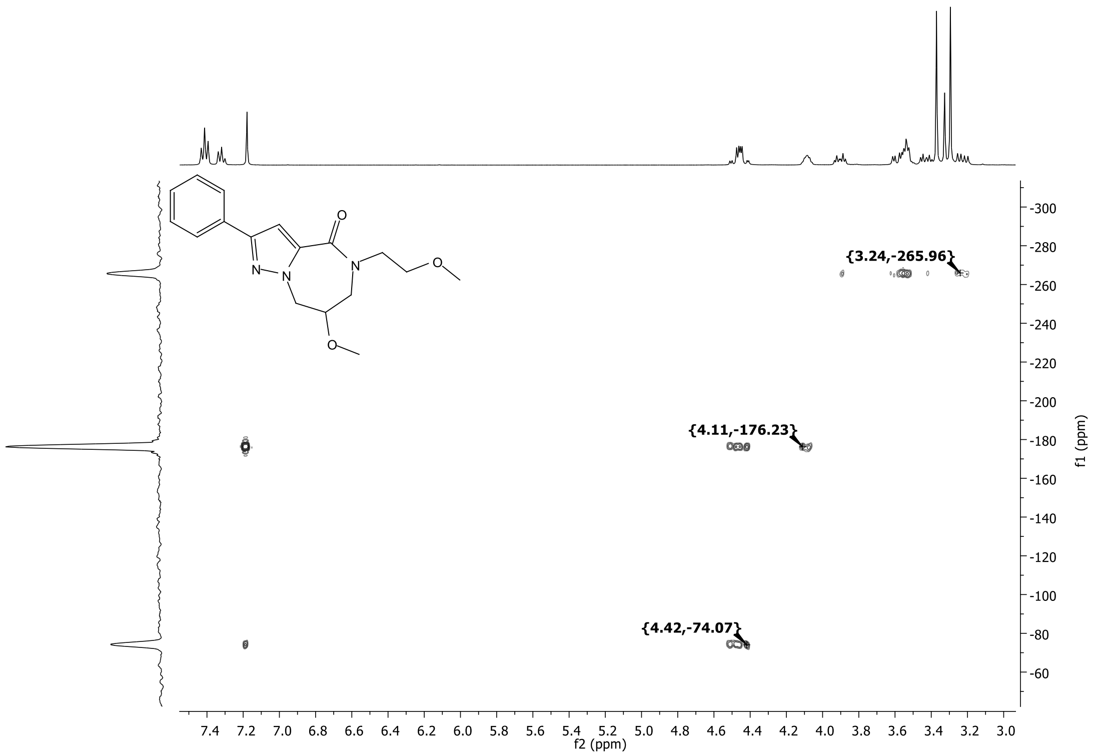

Figure S200. $^{1}H,^{15}N$-HMBC spectrum (40 MHz, DMSO-d_6) of 7-methoxy-5-(2-methoxyethyl)-2-phenyl-5,6,7,8-tetrahydro-4*H*-pyrazolo[1,5-*a*][1,4]diazepin-4-one (**8a**).

Compound Spectrum SmartFormula Report

Analysis Info

Analysis Name	D:\Data\KDD-094.d	Acquisition Date	4/3/2020 1:51:37 PM
Method	DirectInfusion_TuneLow_pos.m	Operator	hplc
Sample Name	KDD-094	Instrument	micrOTOF-Q III 8228888.20448
Comment	SB		

Acquisition Parameter

Source Type	ESI	Ion Polarity	Positive	Set Nebulizer	0.4 Bar
Focus	Not active	Set Capillary	4500 V	Set Dry Heater	180 °C
Scan Begin	50 m/z	Set End Plate Offset	-500 V	Set Dry Gas	4.0 l/min
Scan End	1000 m/z	Set Collision Cell RF	140.0 Vpp	Set Divert Valve	Waste

#	RT [min]	Area	Int. Type	I	S/N	Chromatogram	Max. m/z	FWHM [min]
n.a.	0.4	n.a.	Single spectrum	n.a.	n.a.	n.a.	274.2738	n.a.
n.a.	4.5	n.a.	Single spectrum	n.a.	n.a.	n.a.	338.1475	n.a.

+MS, 4.5min #267

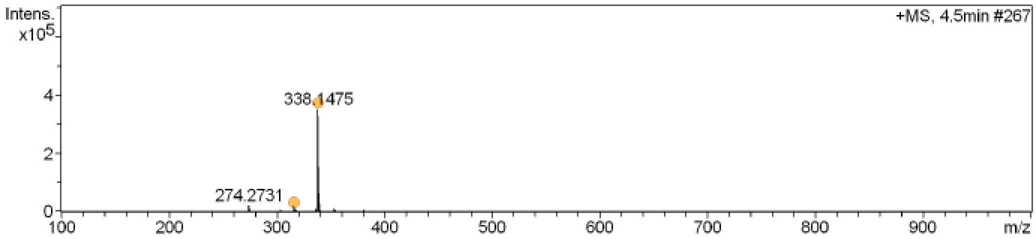

Meas. m/z	#	Ion Formula	m/z	err [ppm]	mSigma	# Sigma	Score	rdb	e⁻ Conf	N-Rule
316.1658	1	C17H22N3O3	316.1656	-0.7	28.3	1	100.00	8.5	even	ok
338.1475	1	C17H21N3NaO3	338.1475	-0.0	3.3	1	100.00	8.5	even	ok

Figure S201. HRMS (ESI-TOF) spectrum of 7-methoxy-5-(2-methoxyethyl)-2-phenyl-5,6,7,8-tetrahydro-4*H*-pyrazolo[1,5-a][1,4]diazepin-4-one (**8a**).

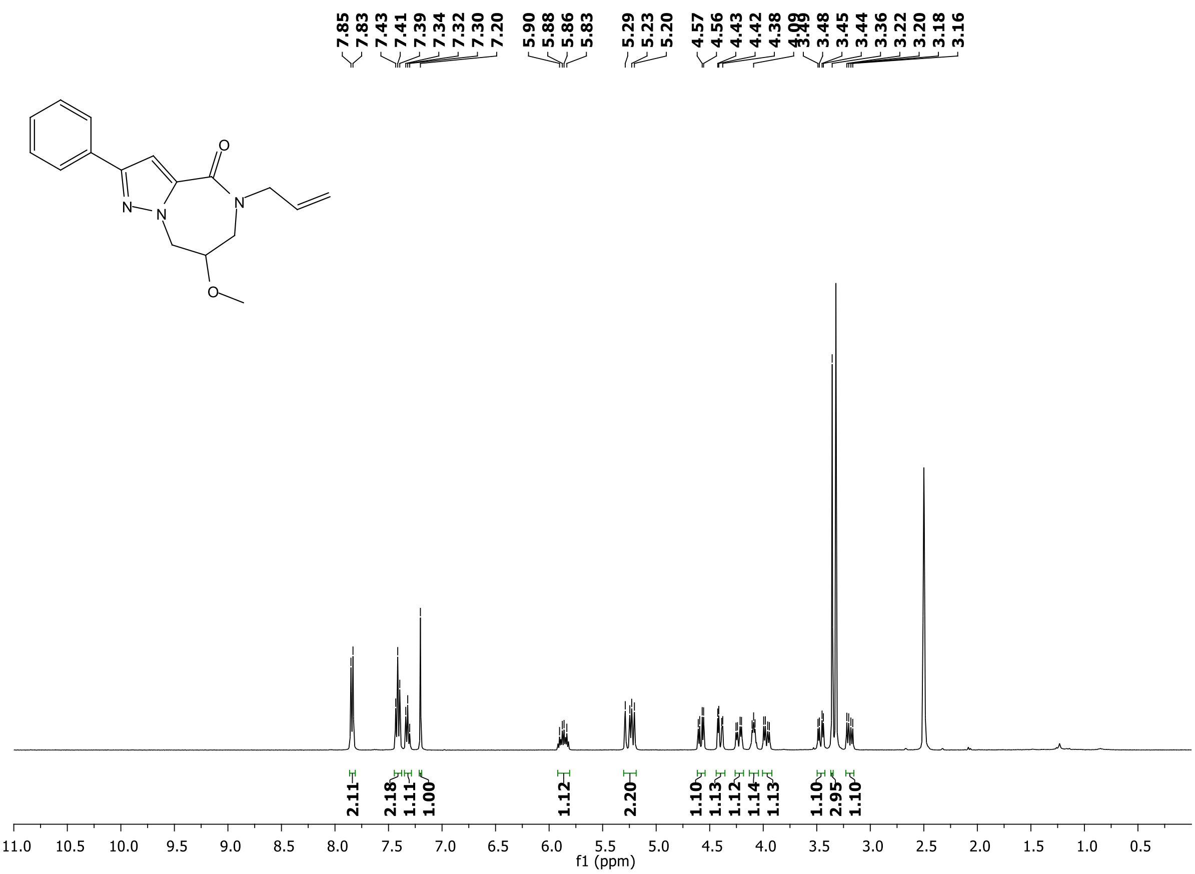

Figure S202. ^{1}H NMR spectrum (400 MHz, DMSO-*d*6) of 5-allyl-7-methoxy-2-phenyl-5,6,7,8-tetrahydro-4*H*-pyrazolo[1,5-*a*][1,4]diazepin-4-one (**8b**).

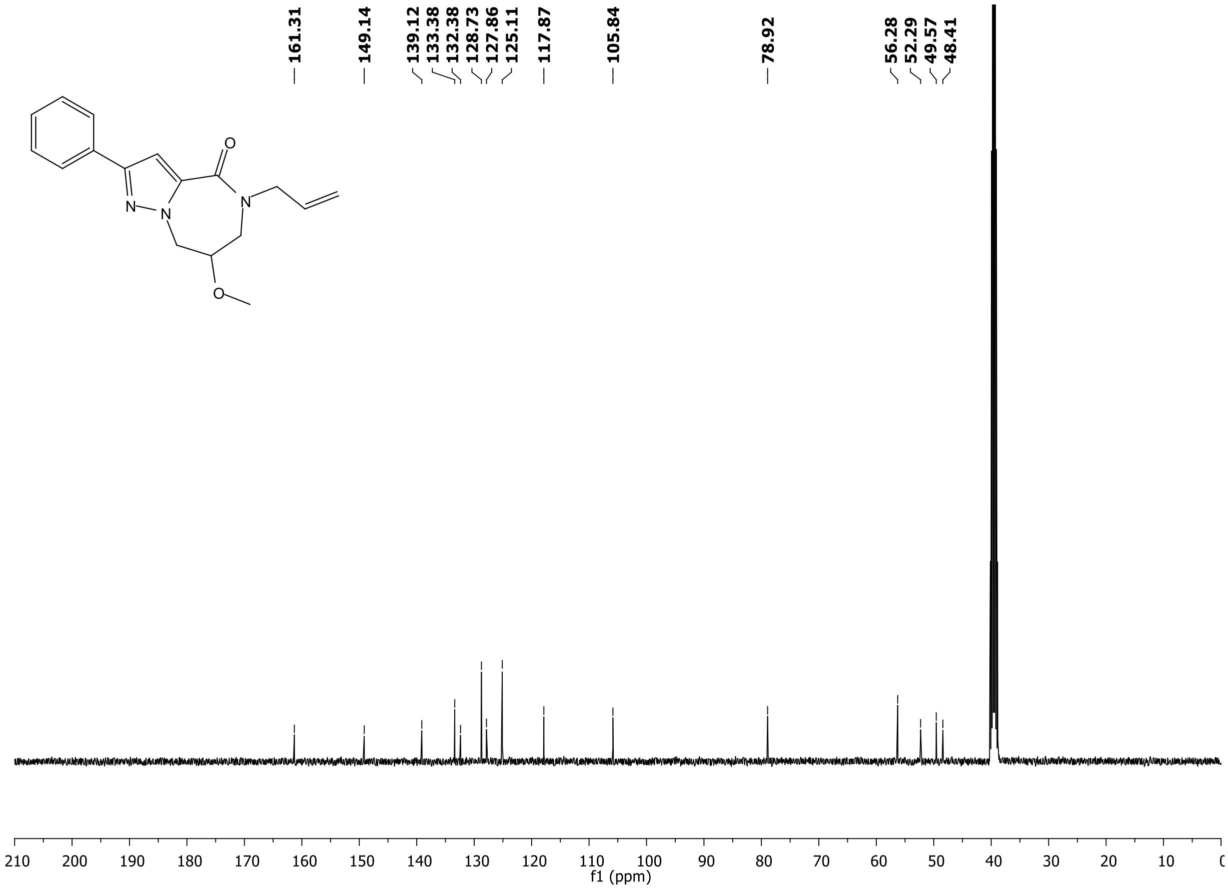

Figure S203. ^{13}C NMR spectrum (101 MHz, DMSO-*d*6) of 5-allyl-7-methoxy-2-phenyl-5,6,7,8-tetrahydro-4*H*-pyrazolo[1,5-*a*][1,4]diazepin-4-one (**8b**).

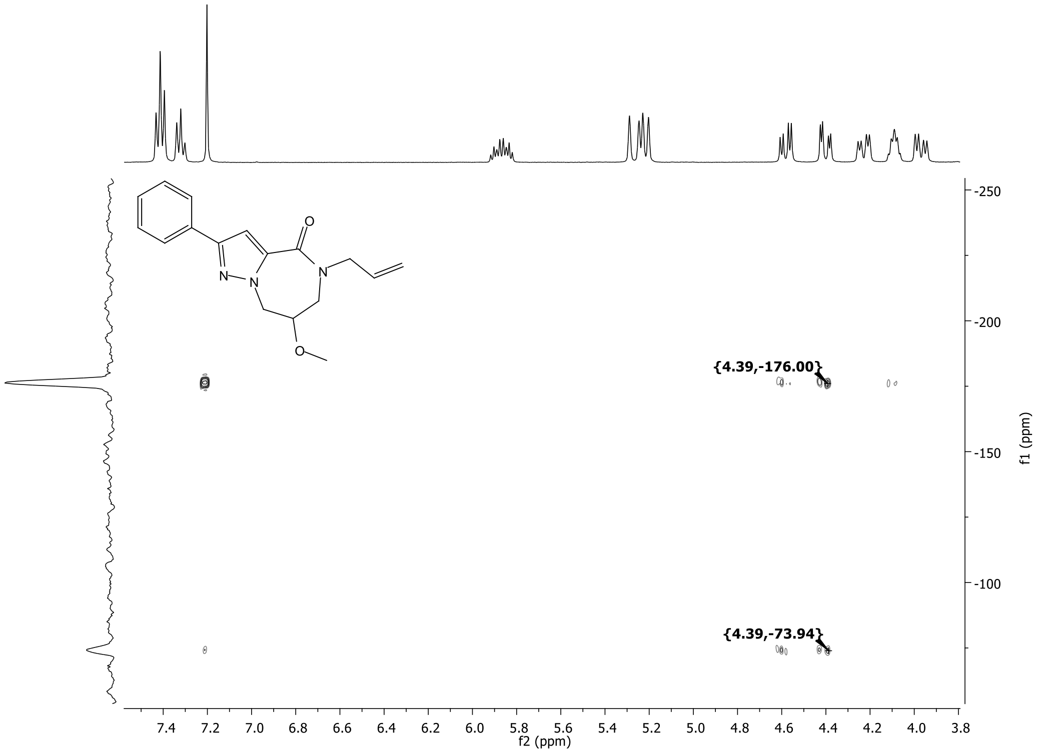

Figure S204. ^{1}H,^{15}N-HMBC spectrum (40 MHz, DMSO-d_6) of 5-allyl-7-methoxy-2-phenyl-5,6,7,8-tetrahydro-4*H*-pyrazolo[1,5-*a*][1,4]diazepin-4-one (**8b**).

Compound Spectrum SmartFormula Report

Analysis Info

Analysis Name	D:\Data\KDD-093.d	Acquisition Date	4/3/2020 11:11:05 AM
Method	DirectInfusion_TuneLow_pos.m	Operator	hplc
Sample Name	KDD-093	Instrument	micrOTOF-Q III 8228888.20448
Comment	SB		

Acquisition Parameter

Source Type	ESI	Ion Polarity	Positive	Set Nebulizer	0.4 Bar
Focus	Not active	Set Capillary	4500 V	Set Dry Heater	180 °C
Scan Begin	50 m/z	Set End Plate Offset	-500 V	Set Dry Gas	4.0 l/min
Scan End	1000 m/z	Set Collision Cell RF	140.0 Vpp	Set Divert Valve	Waste

#	RT [min]	Area	Int. Type	I	S/N	Chromatogram	Max. m/z	FWHM [min]
n.a.	0.5	n.a.	Single spectrum	n.a.	n.a.	n.a.	274.2744	n.a.
n.a.	5.3	n.a.	Single spectrum	n.a.	n.a.	n.a.	320.1370	n.a.

+MS, 5.3min #318

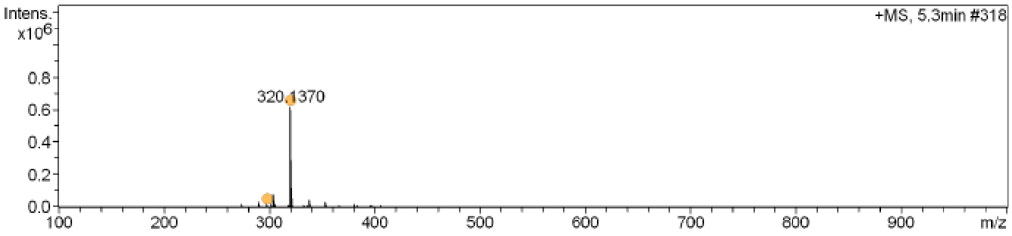

Meas. m/z	#	Ion Formula	m/z	err [ppm]	mSigma	# Sigma	Score	rdb	e⁻ Conf	N-Rule
298.1542	1	C17H20N3O2	298.1550	-2.6	43.2	1	100.00	9.5	even	ok
320.1370	1	C17H19N3NaO2	320.1369	0.1	1.1	1	100.00	9.5	even	ok

Figure S205. HRMS (ESI-TOF) spectrum of 5-allyl-7-methoxy-2-phenyl-5,6,7,8-tetrahydro-4*H*-pyrazolo[1,5-*a*][1,4]diazepin-4-one (**8b**).

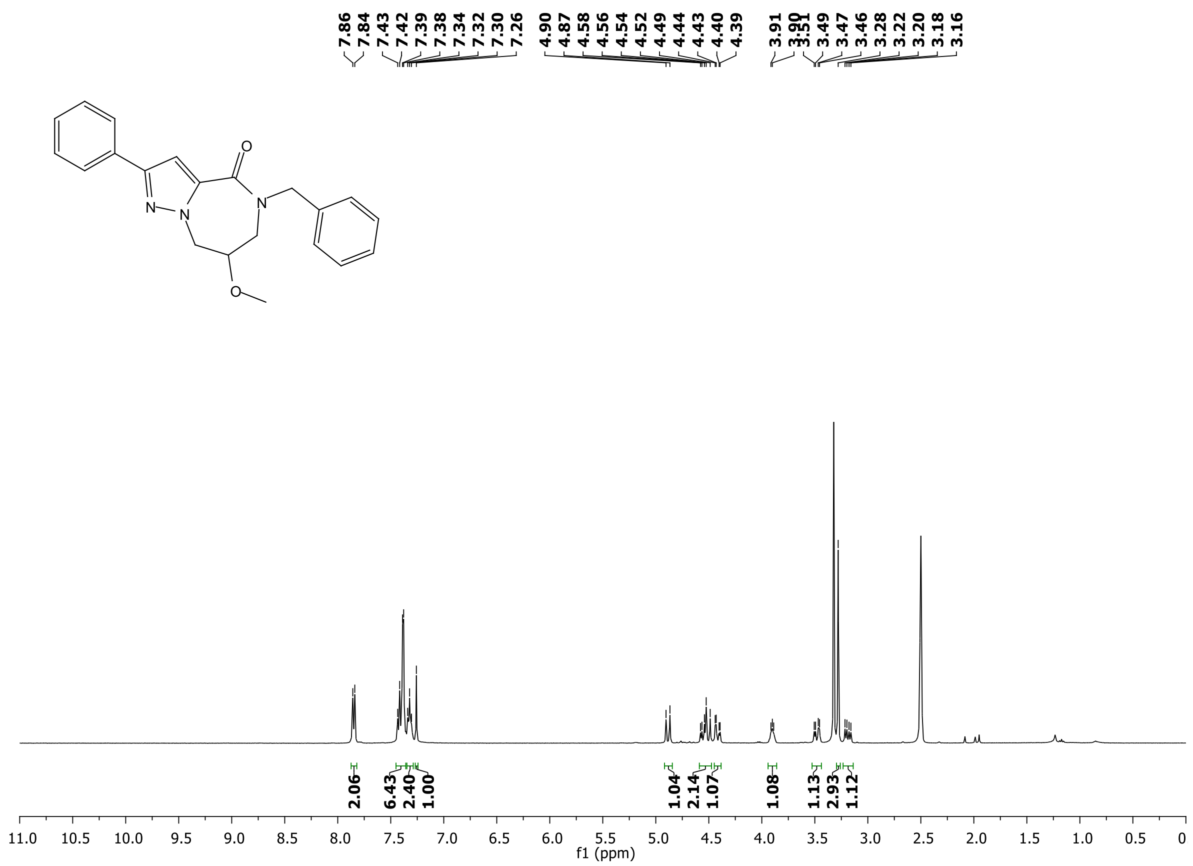

Figure S206. ^{1}H NMR spectrum (400 MHz, DMSO-d_6) of 5-benzyl-7-methoxy-2-phenyl-5,6,7,8-tetrahydro-4*H*-pyrazolo[1,5-*a*][1,4]diazepin-4-one (**8c**).

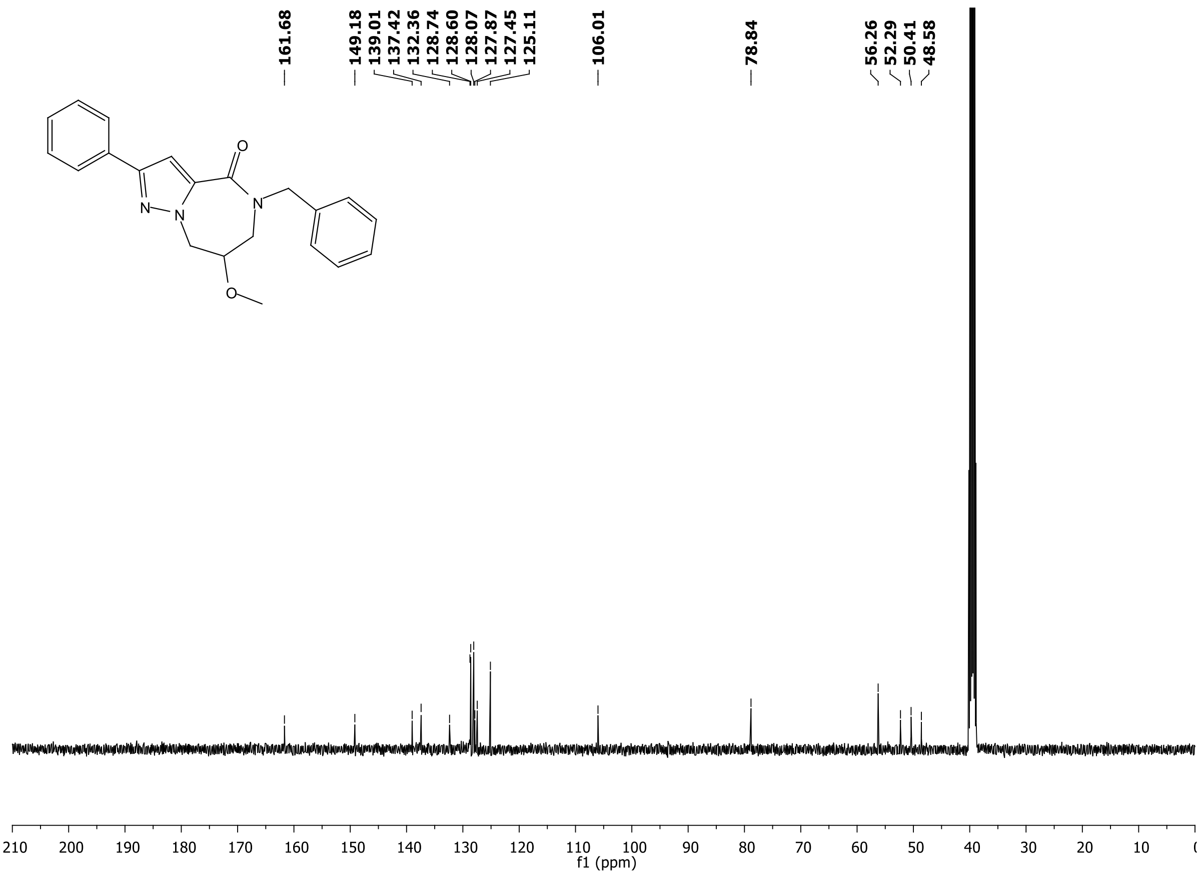

Figure S207. ^{13}C NMR spectrum (101 MHz, DMSO-d_6) of 5-benzyl-7-methoxy-2-phenyl-5,6,7,8-tetrahydro-4*H*-pyrazolo[1,5-*a*][1,4]diazepin-4-one (**8c**).

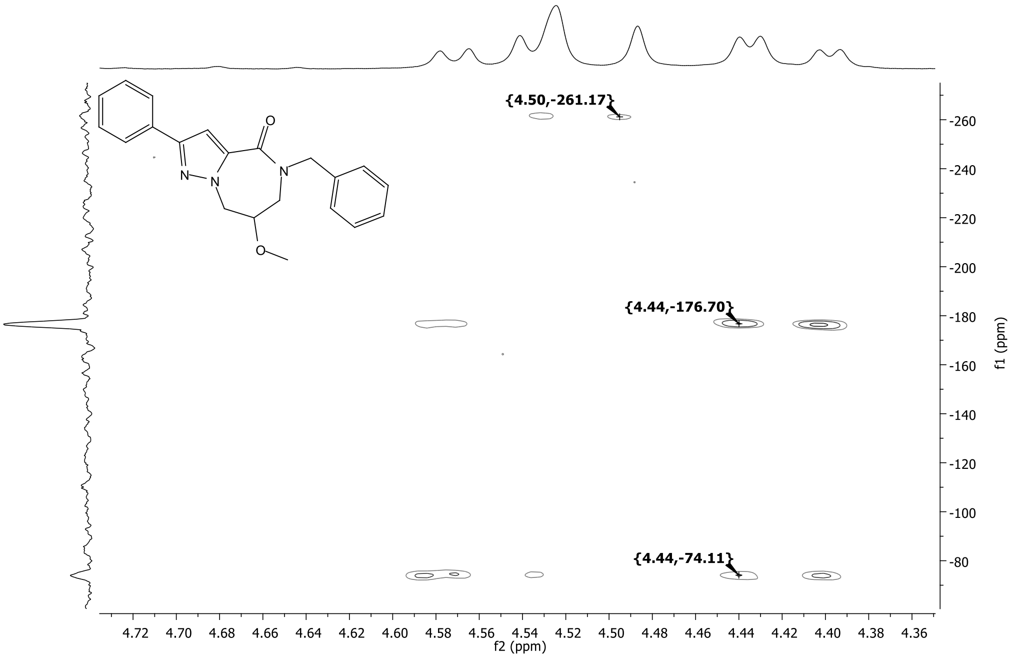

Figure S208. $^{1}H,^{15}N$-HMBC spectrum (40 MHz, DMSO-d_6) of 5-benzyl-7-methoxy-2-phenyl-5,6,7,8-tetrahydro-4*H*-pyrazolo[1,5-*a*][1,4]diazepin-4-one (**8c**).

Compound Spectrum SmartFormula Report

Analysis Info

Analysis Name	D:\Data\KDD-096.d	Acquisition Date	4/3/2020 11:53:43 AM
Method	DirectInfusion_TuneLow_pos.m	Operator	hplc
Sample Name	KDD-096	Instrument	micrOTOF-Q III 8228888.20448
Comment	SB		

Acquisition Parameter

Source Type	ESI	Ion Polarity	Positive	Set Nebulizer	0.4 Bar
Focus	Not active	Set Capillary	4500 V	Set Dry Heater	180 °C
Scan Begin	50 m/z	Set End Plate Offset	-500 V	Set Dry Gas	4.0 l/min
Scan End	1000 m/z	Set Collision Cell RF	140.0 Vpp	Set Divert Valve	Waste

#	RT [min]	Area	Int. Type	I	S/N	Chromatogram	Max. m/z	FWHM [min]
n.a.	0.3	n.a.	Single spectrum	n.a.	n.a.	n.a.	274.2743	n.a.
n.a.	3.3	n.a.	Single spectrum	n.a.	n.a.	n.a.	370.1526	n.a.

+MS, 3.3min #199

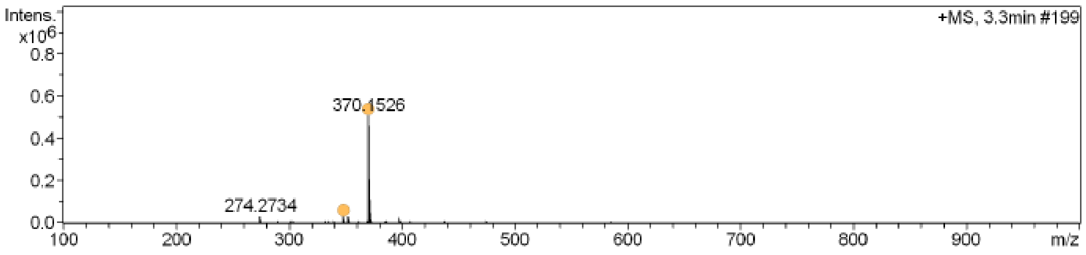

Meas. m/z	#	Ion Formula	m/z	err [ppm]	mSigma	# Sigma	Score	rdb	e⁻ Conf	N-Rule
348.1709	1	C21H22N3O2	348.1707	0.6	15.3	1	100.00	12.5	even	ok
370.1526	1	C21H21N3NaO2	370.1526	-0.0	2.1	1	100.00	12.5	even	ok

Figure S209. HRMS (ESI-TOF) spectrum of 5-benzyl-7-methoxy-2-phenyl-5,6,7,8-tetrahydro-4*H*-pyrazolo[1,5-*a*][1,4]diazepin-4-one (**8c**).

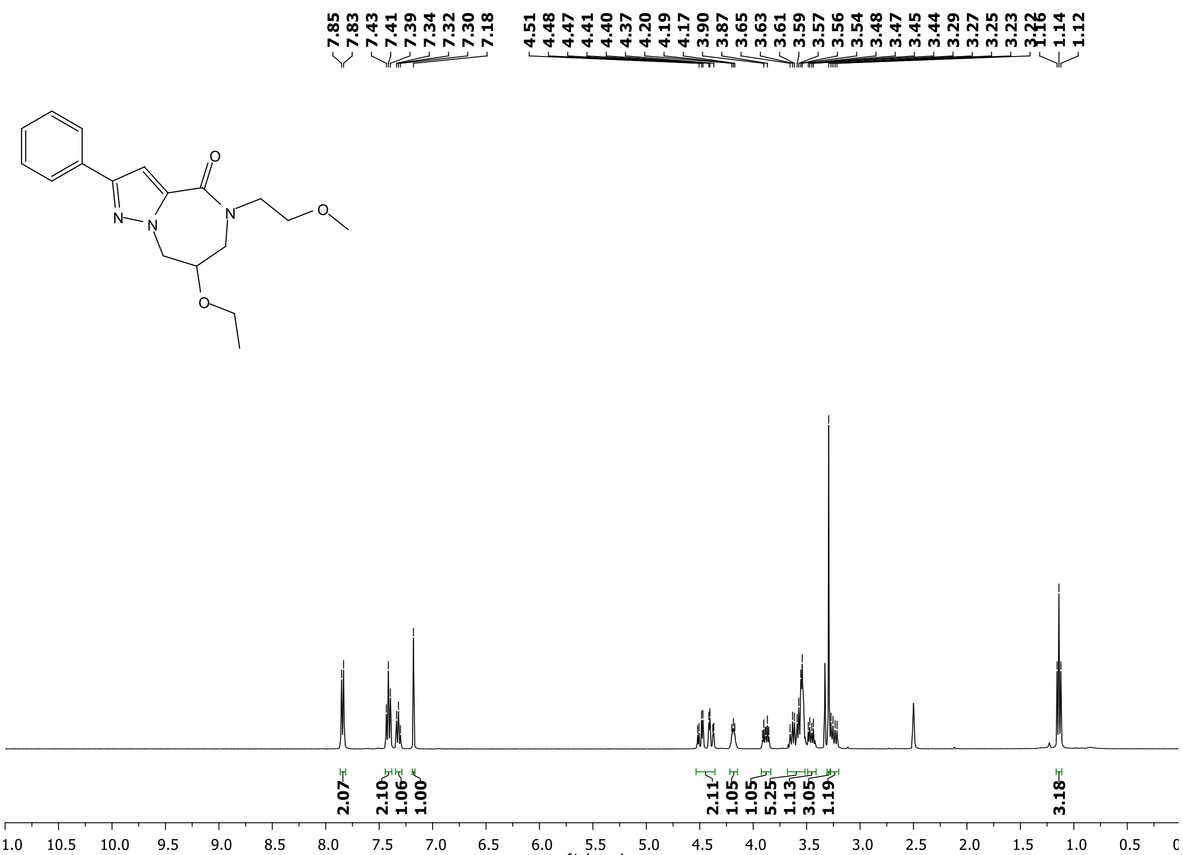

Figure S210. ^{1}H NMR spectrum (400 MHz, DMSO-*d*$_6$) of 7-ethoxy-5-(2-methoxyethyl)-2-phenyl-5,6,7,8-tetrahydro-4*H*-pyrazolo[1,5-*a*][1,4]diazepin-4-one (**8d**).

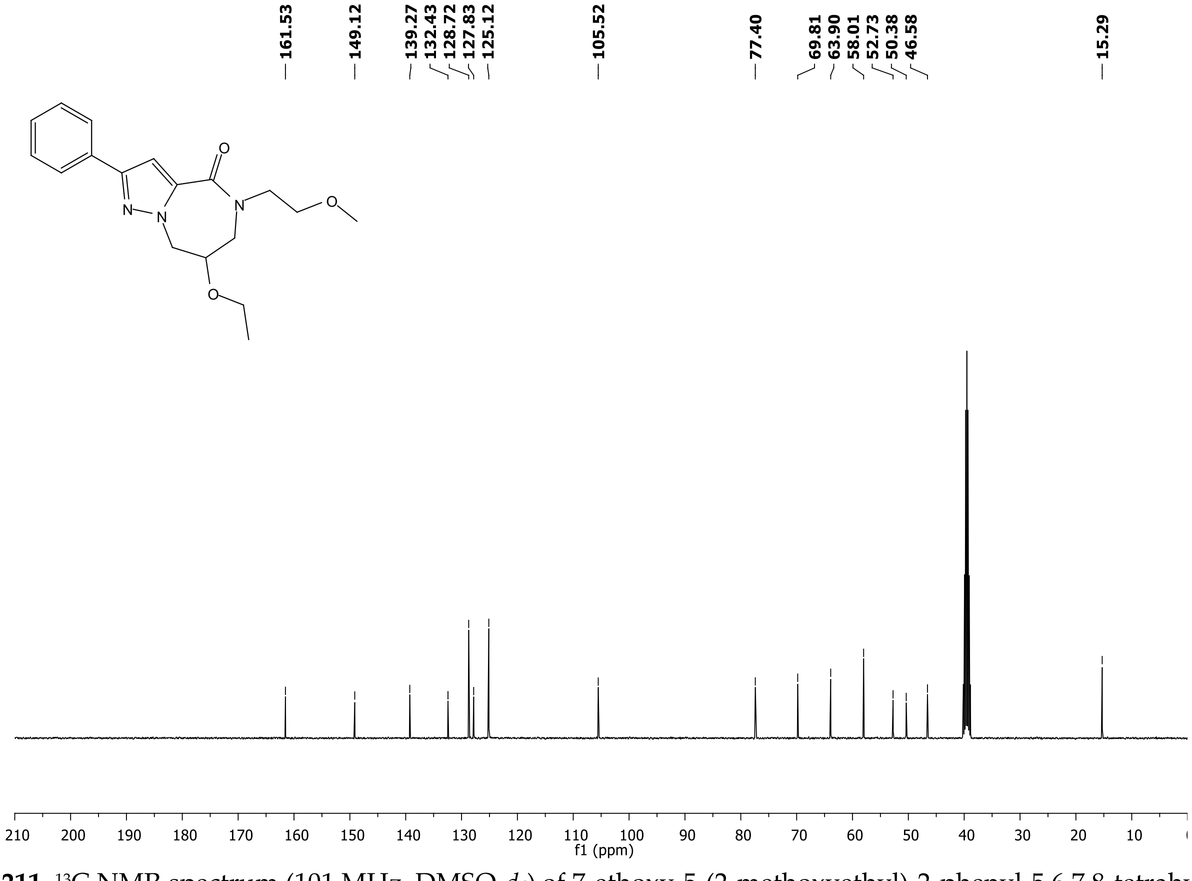

Figure S211. ^{13}C NMR spectrum (101 MHz, DMSO-*d*$_6$) of 7-ethoxy-5-(2-methoxyethyl)-2-phenyl-5,6,7,8-tetrahydro-4*H*-pyrazolo[1,5-*a*][1,4]diazepin-4-one (**8d**).

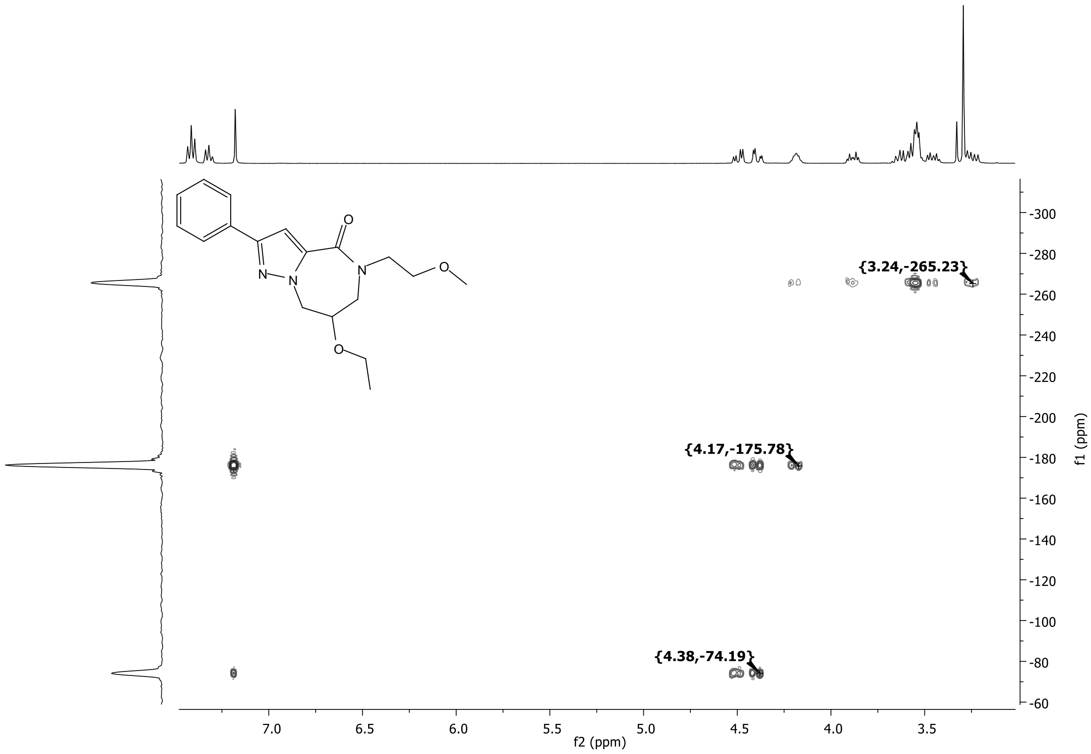

Figure S212. ^{1}H,^{15}N-HMBC spectrum (40 MHz, DMSO-d_6) of 7-ethoxy-5-(2-methoxyethyl)-2-phenyl-5,6,7,8-tetrahydro-4*H*-pyrazolo[1,5-*a*][1,4]diazepin-4-one (**8d**).

Compound Spectrum SmartFormula Report

Analysis Info

Analysis Name	D:\Data\KDD-095.d	Acquisition Date	4/3/2020 11:30:03 AM
Method	DirectInfusion_TuneLow_pos.m	Operator	hplc
Sample Name	KDD-095	Instrument	micrOTOF-Q III 8228888.20448
Comment	SB		

Acquisition Parameter

Source Type	ESI	Ion Polarity	Positive	Set Nebulizer	0.4 Bar
Focus	Not active	Set Capillary	4500 V	Set Dry Heater	180 °C
Scan Begin	50 m/z	Set End Plate Offset	-500 V	Set Dry Gas	4.0 l/min
Scan End	1000 m/z	Set Collision Cell RF	140.0 Vpp	Set Divert Valve	Waste

#	RT [min]	Area	Int. Type	I	S/N	Chromatogram	Max. m/z	FWHM [min]
n.a.	1.6	n.a.	Single spectrum	n.a.	n.a.	n.a.	274.2742	n.a.
n.a.	6.7	n.a.	Single spectrum	n.a.	n.a.	n.a.	352.1632	n.a.

+MS, 6.7min #403

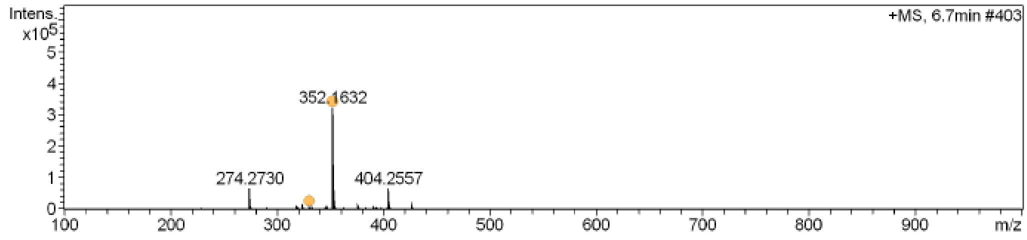

Meas. m/z	#	Ion Formula	m/z	err [ppm]	mSigma	# Sigma	Score	rdb	e⁻ Conf	N-Rule
330.1810	1	C18H24N3O3	330.1812	-0.6	90.2	1	100.00	8.5	even	ok
352.1632	1	C18H23N3NaO3	352.1632	0.2	4.5	1	100.00	8.5	even	ok

Figure S213. HRMS (ESI-TOF) spectrum of 7-ethoxy-5-(2-methoxyethyl)-2-phenyl-5,6,7,8-tetrahydro-4*H*-pyrazolo[1,5-*a*][1,4]diazepin-4-one (**8d**).

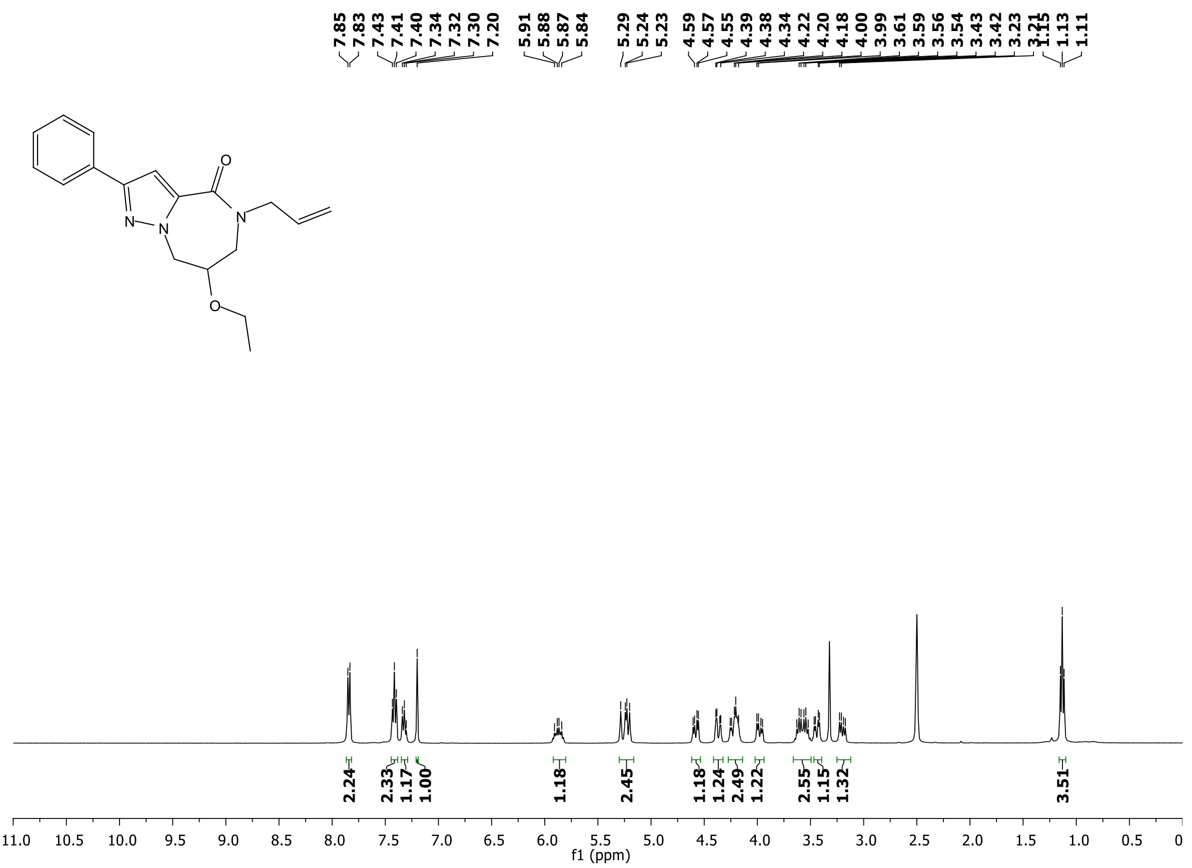

Figure S214. ^{1}H NMR spectrum (400 MHz, DMSO-d_6) of 5-allyl-7-ethoxy-2-phenyl-5,6,7,8-tetrahydro-4*H*-pyrazolo[1,5-*a*][1,4]diazepin-4-one (**8e**).

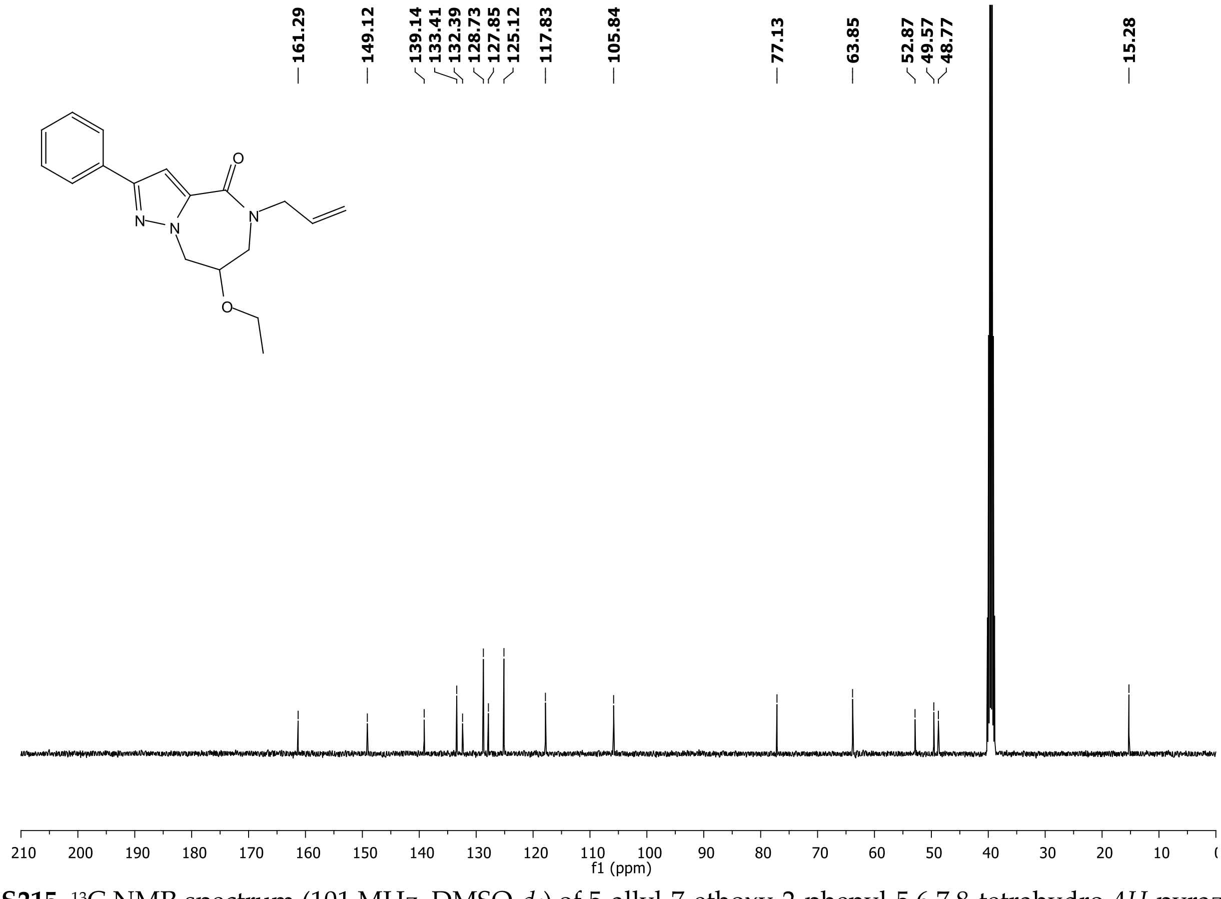

Figure S215. ^{13}C NMR spectrum (101 MHz, DMSO-d_6) of 5-allyl-7-ethoxy-2-phenyl-5,6,7,8-tetrahydro-4*H*-pyrazolo[1,5-*a*][1,4]diazepin-4-one (**8e**).

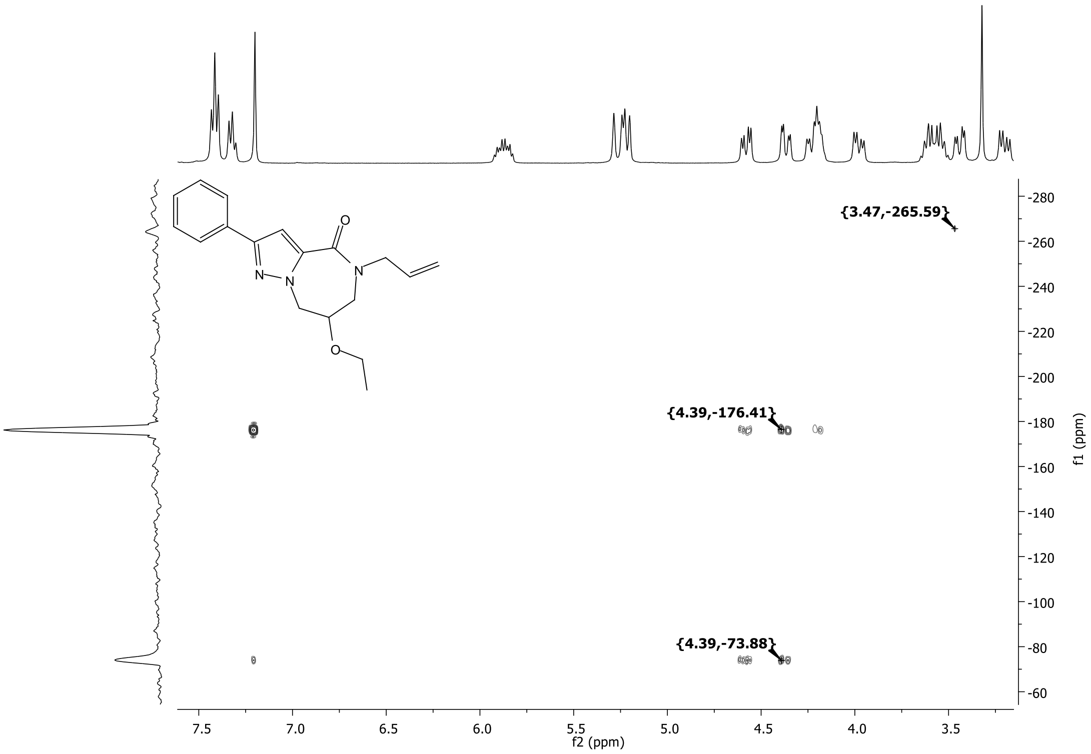

Figure S216. $^{1}H,^{15}N$-HMBC spectrum (40 MHz, DMSO-d_6) of 5-allyl-7-ethoxy-2-phenyl-5,6,7,8-tetrahydro-4*H*-pyrazolo[1,5-*a*][1,4]diazepin-4-one (**8e**).

Compound Spectrum SmartFormula Report

Analysis Info

Analysis Name	D:\Data\KDD-099.d	Acquisition Date	4/3/2020 12:22:38 PM
Method	DirectInfusion_TuneLow_pos.m	Operator	hplc
Sample Name	KDD-099	Instrument	micrOTOF-Q III 8228888.20448
Comment	SB		

Acquisition Parameter

Source Type	ESI	Ion Polarity	Positive	Set Nebulizer	0.4 Bar
Focus	Not active	Set Capillary	4500 V	Set Dry Heater	180 °C
Scan Begin	50 m/z	Set End Plate Offset	-500 V	Set Dry Gas	4.0 l/min
Scan End	1000 m/z	Set Collision Cell RF	140.0 Vpp	Set Divert Valve	Waste

#	RT [min]	Area	Int. Type	I	S/N	Chromatogram	Max. m/z	FWHM [min]
n.a.	0.3	n.a.	Single spectrum	n.a.	n.a.	n.a.	274.2741	n.a.
n.a.	5.0	n.a.	Single spectrum	n.a.	n.a.	n.a.	334.1526	n.a.

+MS, 5.0min #301

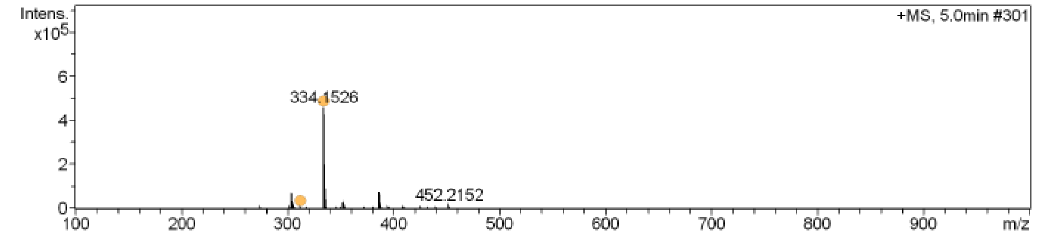

Meas. m/z	#	Ion Formula	m/z	err [ppm]	mSigma	# Sigma	Score	rdb	e⁻ Conf	N-Rule
312.1702	1	C18H22N3O2	312.1707	1.4	14.5	1	100.00	9.5	even	ok
334.1526	1	C18H21N3NaO2	334.1526	-0.1	0.7	1	100.00	9.5	even	ok

Figure S217. HRMS (ESI-TOF) spectrum of 5-allyl-7-ethoxy-2-phenyl-5,6,7,8-tetrahydro-4*H*-pyrazolo[1,5-*a*][1,4]diazepin-4-one (**8e**).

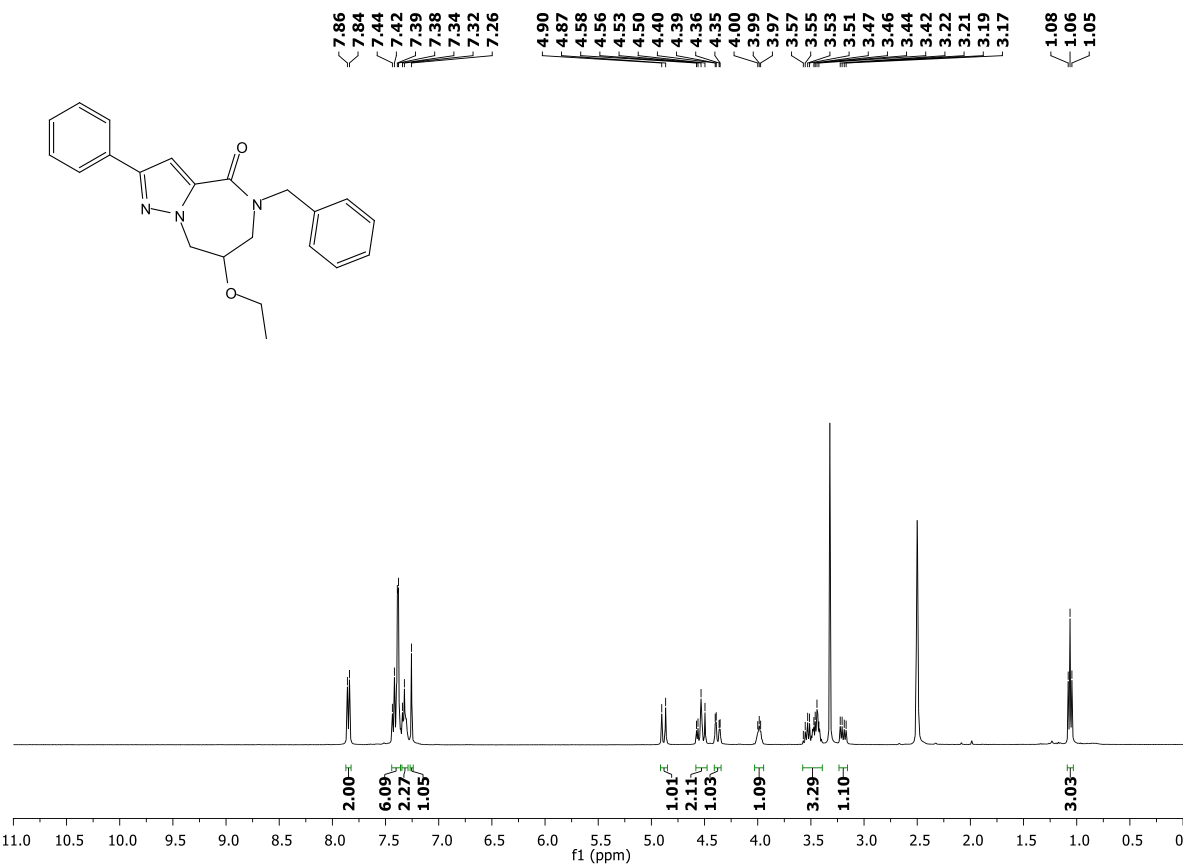

Figure S218. ^{1}H NMR spectrum (400 MHz, DMSO-d_6) of 5-benzyl-7-ethoxy-2-phenyl-5,6,7,8-tetrahydro-4*H*-pyrazolo[1,5-*a*][1,4]diazepin-4-one (**8f**).

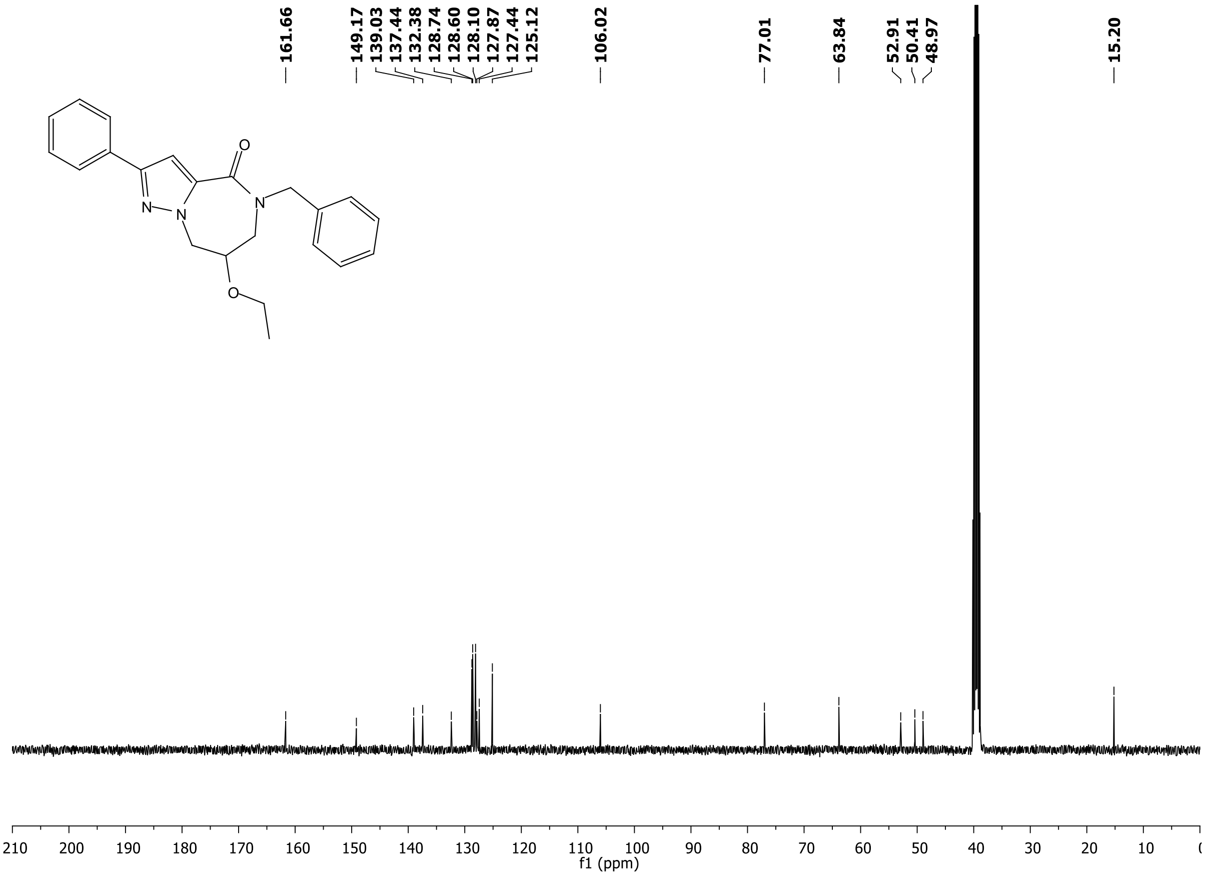

Figure S219. ^{13}C NMR spectrum (101 MHz, DMSO-d_6) of 5-benzyl-7-ethoxy-2-phenyl-5,6,7,8-tetrahydro-4*H*-pyrazolo[1,5-*a*][1,4]diazepin-4-one (**8f**).

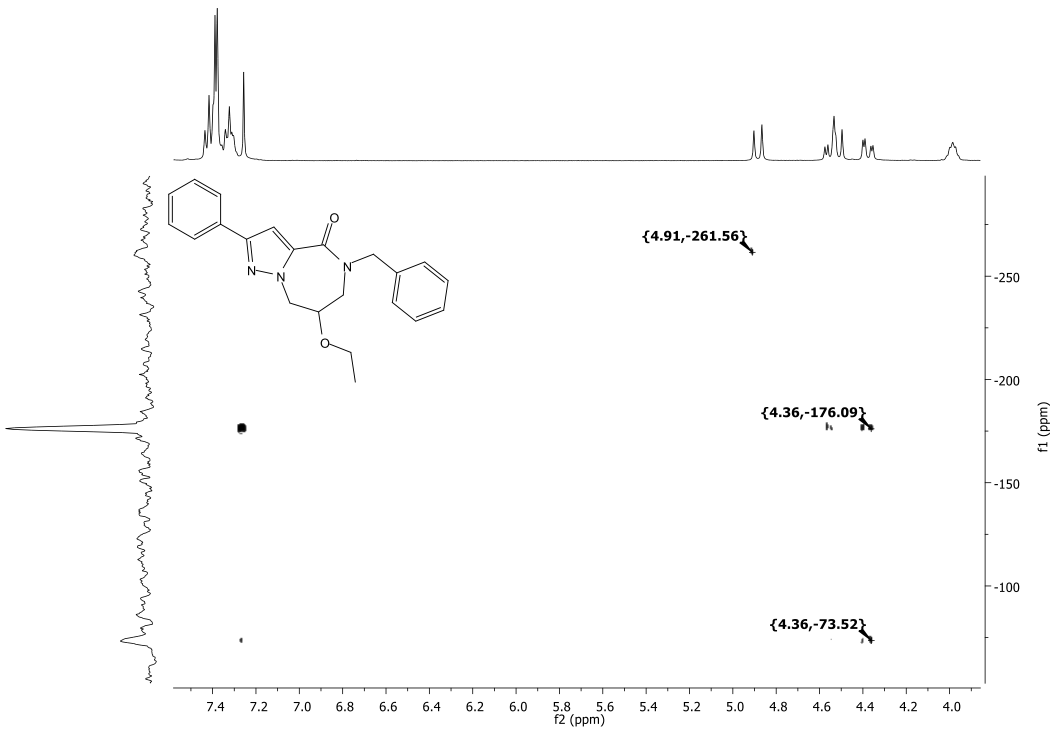

Figure S220. ^{1}H,^{15}N-HMBC spectrum (40 MHz, DMSO-d_6) of 5-benzyl-7-ethoxy-2-phenyl-5,6,7,8-tetrahydro-4*H*-pyrazolo[1,5-*a*][1,4]diazepin-4-one (**8f**).

Compound Spectrum SmartFormula Report

Analysis Info

Analysis Name	D:\Data\KDD-097.d	Acquisition Date	4/3/2020 12:07:06 PM
Method	DirectInfusion_TuneLow_pos.m	Operator	hplc
Sample Name	KDD-097	Instrument	micrOTOF-Q III 8228888.20448
Comment	SB		

Acquisition Parameter

Source Type	ESI	Ion Polarity	Positive	Set Nebulizer	0.4 Bar
Focus	Not active	Set Capillary	4500 V	Set Dry Heater	180 °C
Scan Begin	50 m/z	Set End Plate Offset	-500 V	Set Dry Gas	4.0 l/min
Scan End	1000 m/z	Set Collision Cell RF	140.0 Vpp	Set Divert Valve	Waste

#	RT [min]	Area	Int. Type	I	S/N	Chromatogram	Max. m/z	FWHM [min]
n.a.	0.3	n.a.	Single spectrum	n.a.	n.a.	n.a.	274.2739	n.a.
n.a.	3.6	n.a.	Single spectrum	n.a.	n.a.	n.a.	384.1682	n.a.

+MS, 3.6min #215

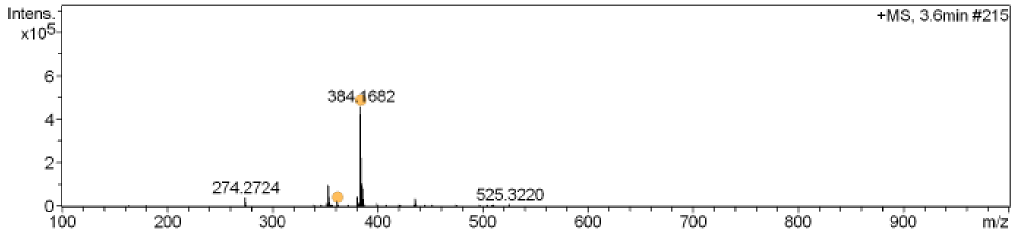

Meas. m/z	#	Ion Formula	m/z	err [ppm]	mSigma	# Sigma	Score	rdb	e⁻ Conf	N-Rule
362.1892	1	C22H24N3O2	362.1863	-7.9	21.6	1	100.00	12.5	even	ok
384.1682	1	C22H23N3NaO2	384.1682	-0.0	4.1	1	100.00	12.5	even	ok

Figure S221. HRMS (ESI-TOF) spectrum of 5-benzyl-7-ethoxy-2-phenyl-5,6,7,8-tetrahydro-4*H*-pyrazolo[1,5-*a*][1,4]diazepin-4-one (**8f**).

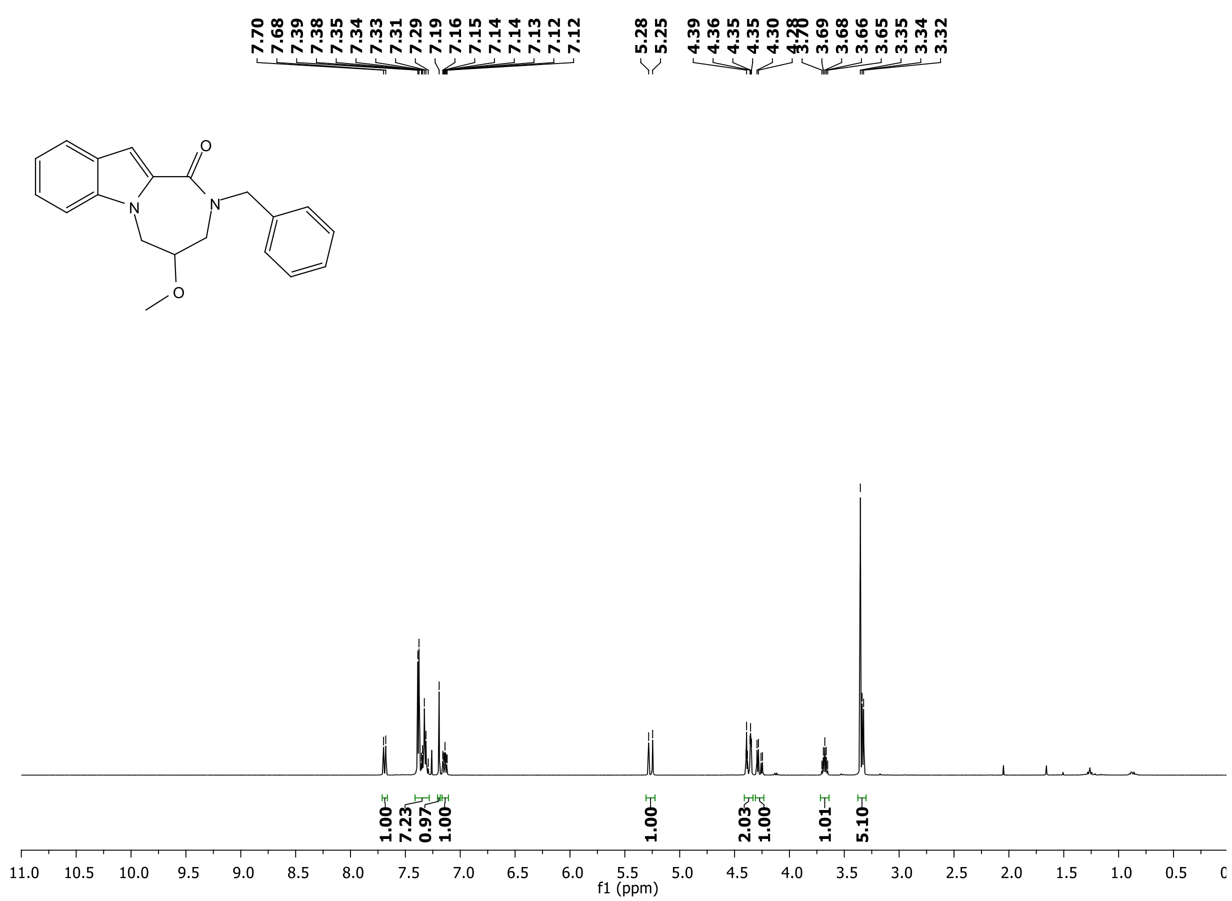

Figure S222. ^{1}H NMR spectrum (400 MHz, DMSO-d_6) of 2-benzyl-4-methoxy-2,3,4,5-tetrahydro-1*H*-[1,4]diazepino[1,2-*a*]indol-1-one (**9a**).

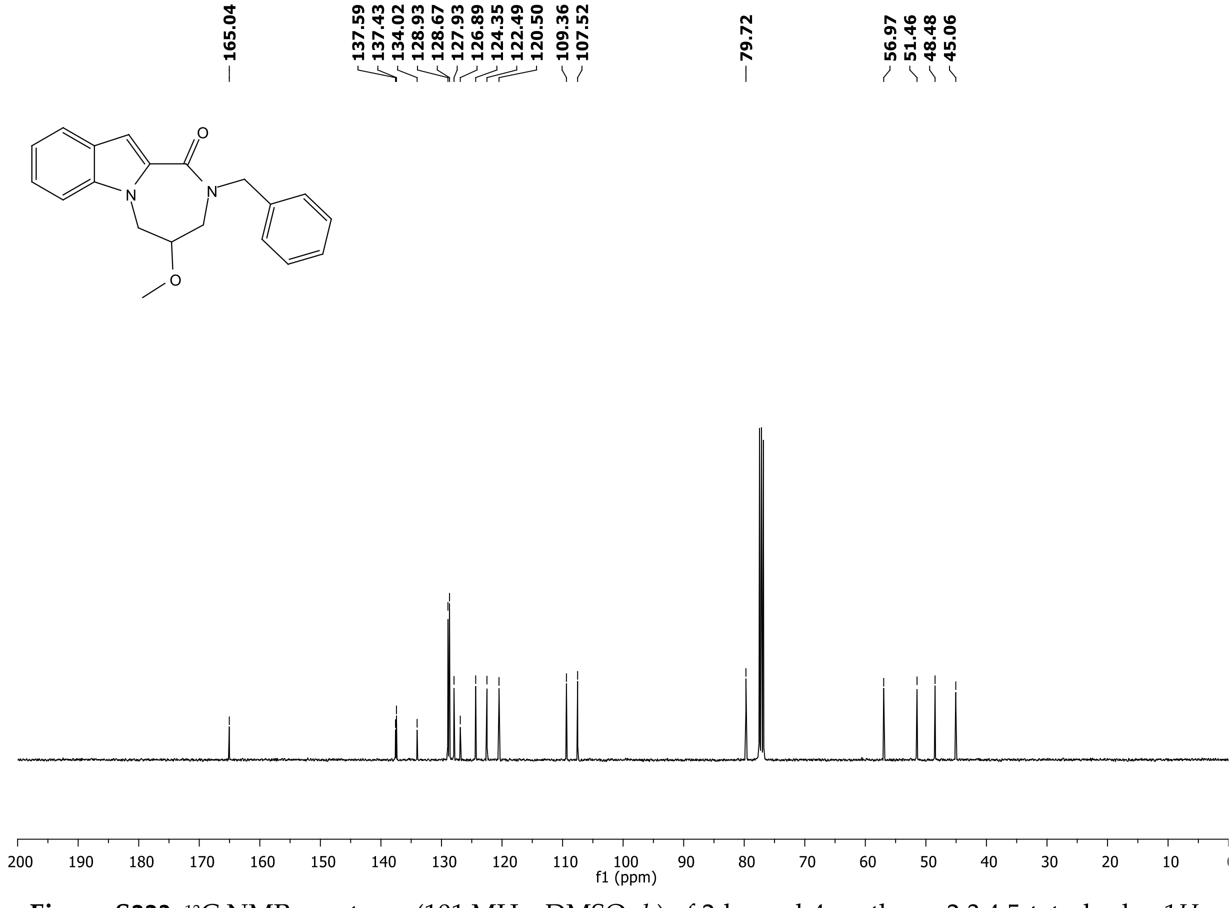

Figure S223. ^{13}C NMR spectrum (101 MHz, DMSO-d_6) of 2-benzyl-4-methoxy-2,3,4,5-tetrahydro-1*H*-[1,4]diazepino[1,2-*a*]indol-1-one (**9a**).

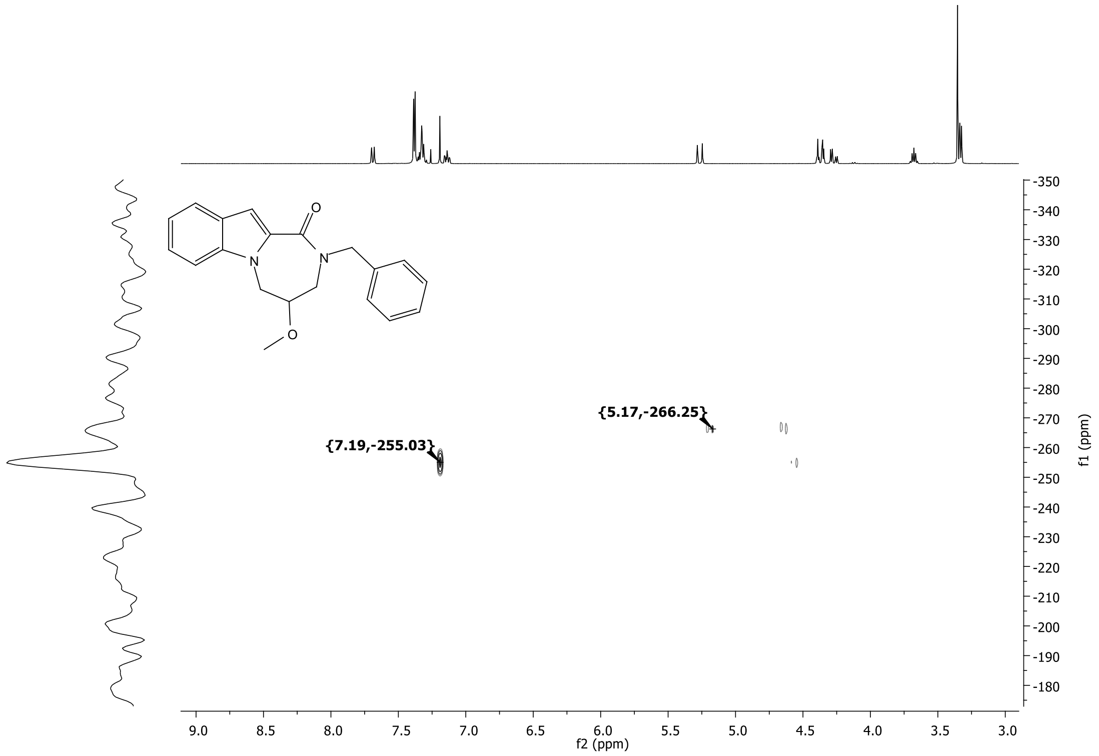

Figure S224. $^{1}H,^{15}N$-HMBC spectrum (40 MHz, DMSO-d_6) of 2-benzyl-4-methoxy-2,3,4,5-tetrahydro-1*H*-[1,4]diazepino[1,2-*a*]indol-1-one (**9a**).

Qualitative Compound Report

**Data File**	221202_KDUP-020-1_02.d	**Sample Name**	KDUP-020-1
**Sample Type**	Sample	**Position**	
**Instrument Name**	G6230B TOF	**User Name**	
**Acq Method**	HRMS_12min_ref_5.m	**Acquired Time**	02-Dec-22 1:08:06 PM
**IRM Calibration Status**	Success	**DA Method**	test.m
**Comment**			
**Sample Group**		**Info.**	
**Stream Name**		**Acquisition SW Version**	6200 series TOF/6500 series Q-TOF B.09.00 (B9044.1 SP1)

Compound Table

Compound Label	RT	Mass	Abund	Formula	Tgt Mass	Diff (ppm)
Cpd 1: C20 H20 N2 O2	8.648	320.1525	186652	C20 H20 N2 O2	320.1525	0.21

Compound Label	*m/z*	RT	Algorithm	Mass
Cpd 1: C20 H20 N2 O2	321.1596	8.648	Find By Formula	320.1525

Compound Chromatograms

MS Spectrum

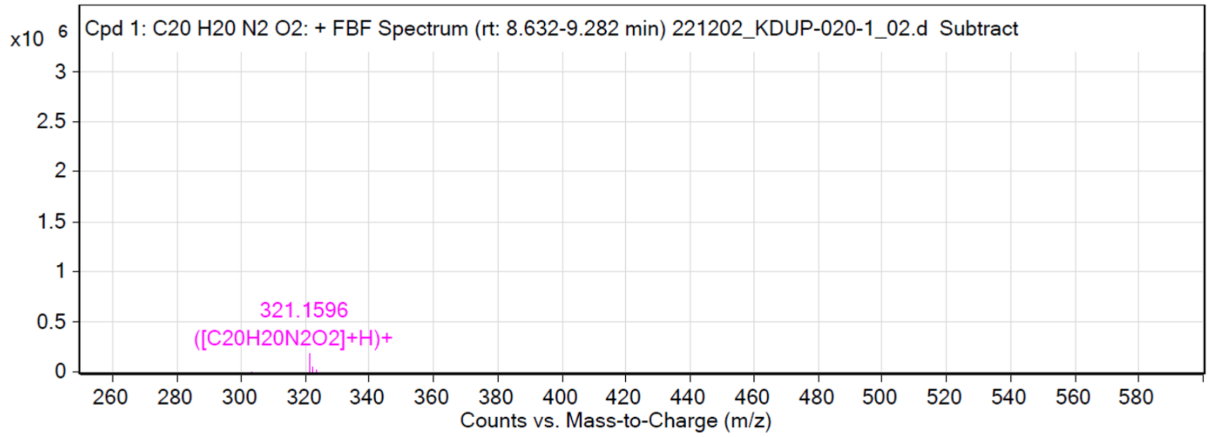

Figure S225. HRMS (ESI-TOF) spectrum of 2-benzyl-4-methoxy-2,3,4,5-tetrahydro-1*H*-[1,4]diazepino[1,2-*a*]indol-1-one (**9a**).

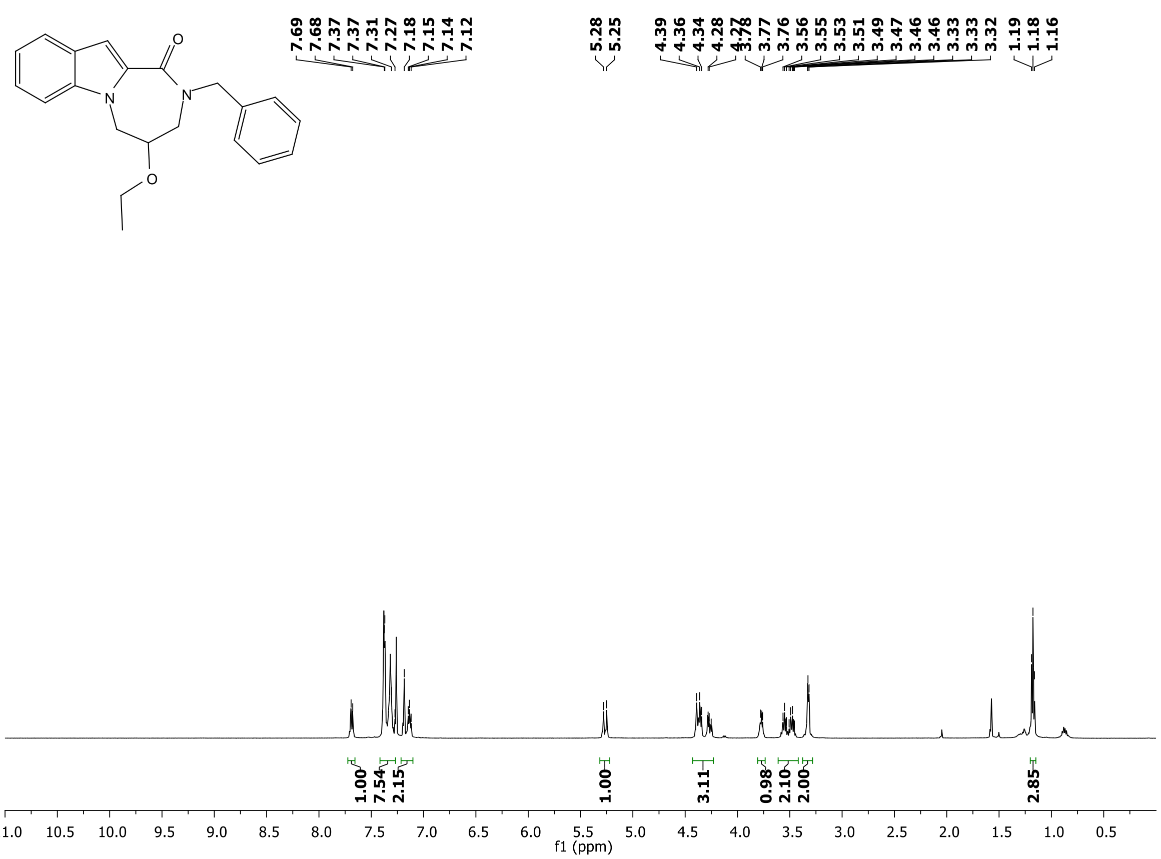

Figure S226. ^{1}H NMR spectrum (400 MHz, DMSO-d_6) of 2-benzyl-4-ethoxy-2,3,4,5-tetrahydro-1*H*-[1,4]diazepino[1,2-*a*]indol-1-one (**9b**).

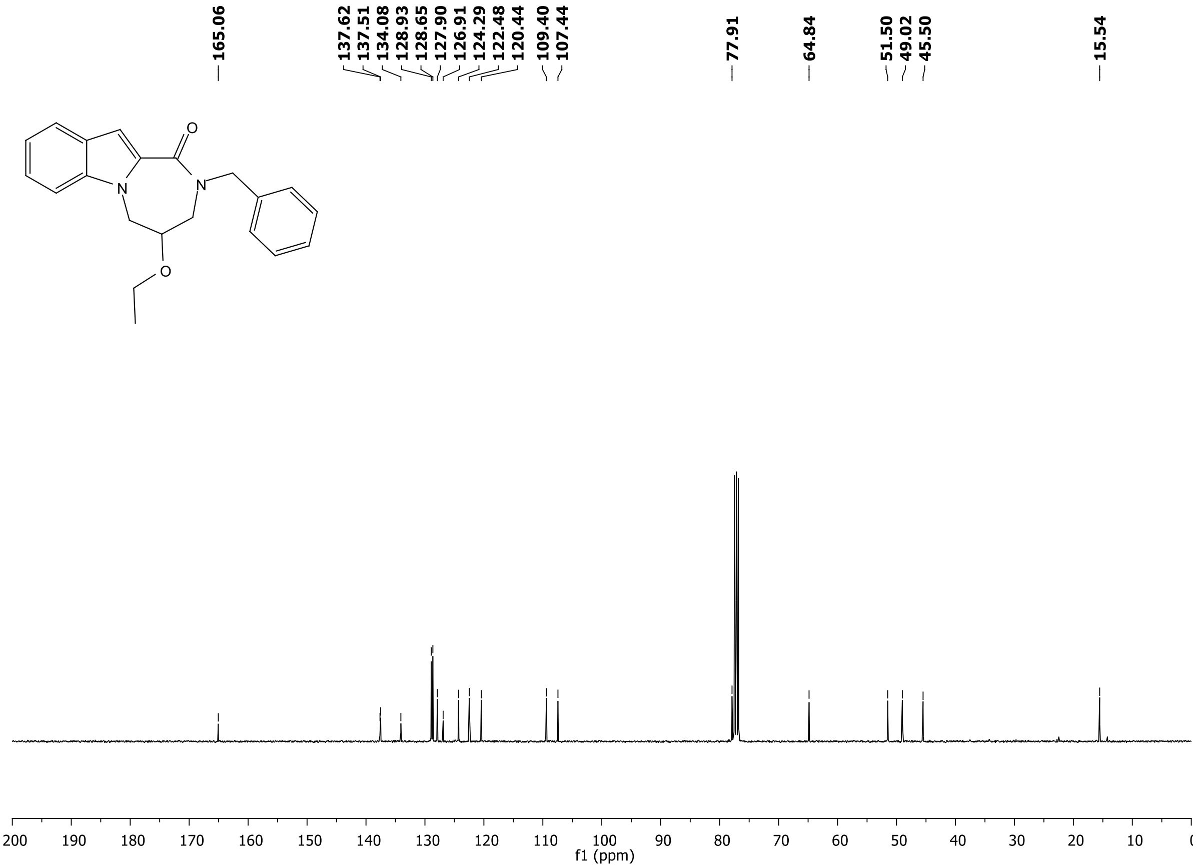

Figure S227. ^{13}C NMR spectrum (101 MHz, DMSO-d_6) of 2-benzyl-4-ethoxy-2,3,4,5-tetrahydro-1*H*-[1,4]diazepino[1,2-*a*]indol-1-one (**9b**).

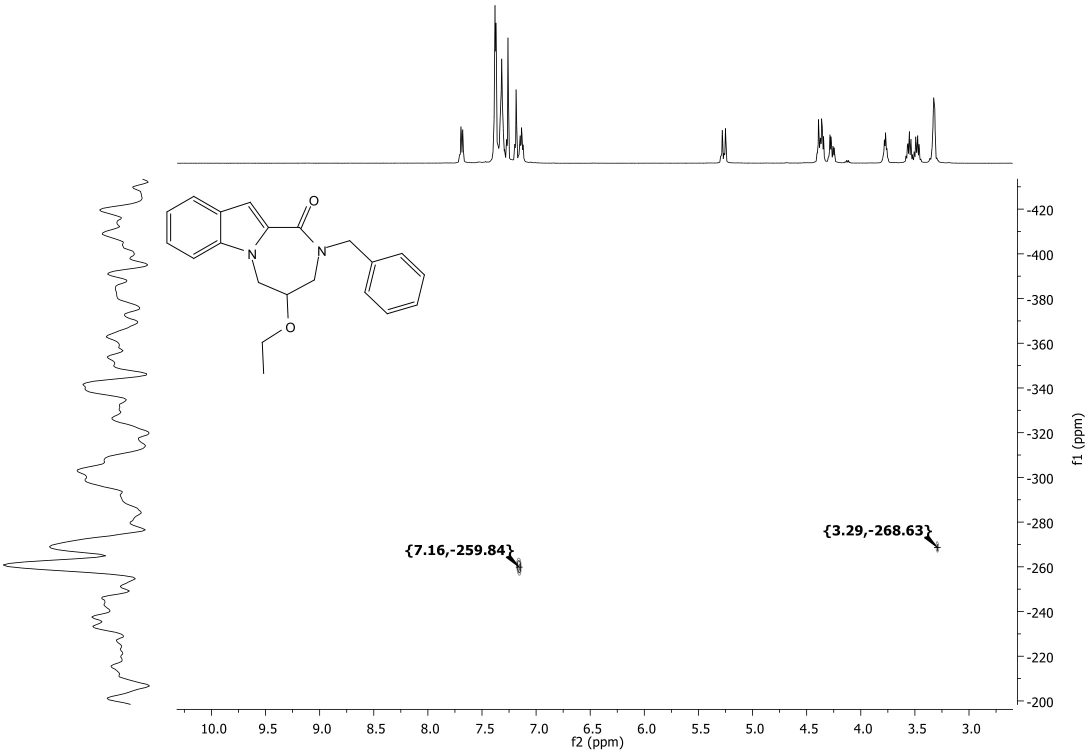

Figure S228. $^{1}H,^{15}N$-HMBC spectrum (40 MHz, DMSO-d_6) of 2-benzyl-4-ethoxy-2,3,4,5-tetrahydro-1*H*-[1,4]diazepino[1,2-*a*]indol-1-one (**9b**).

Qualitative Compound Report

**Data File**	221202_KDUP-022-1_04.d	**Sample Name**	KDUP-022-1
**Sample Type**	Sample	**Position**	
**Instrument Name**	G6230B TOF	**User Name**	
**Acq Method**	HRMS_12min_ref_5.m	**Acquired Time**	02-Dec-22 1:59:57 PM
**IRM Calibration Status**	Success	**DA Method**	test.m
**Comment**			
**Sample Group**		**Info.**	
**Stream Name**		**Acquisition SW Version**	6200 series TOF/6500 series Q-TOF B.09.00 (B9044.1 SP1)

Compound Table

Compound Label	RT	Mass	Abund	Formula	Tgt Mass	Diff (ppm)
Cpd 1: C21 H22 N2 O2	6.83	334.1709	80369	C21 H22 N2 O2	334.1681	8.34

Compound Label	*m/z*	RT	Algorithm	Mass
Cpd 1: C21 H22 N2 O2	669.3476	6.83	Find By Formula	334.1709

Compound Chromatograms

MS Spectrum

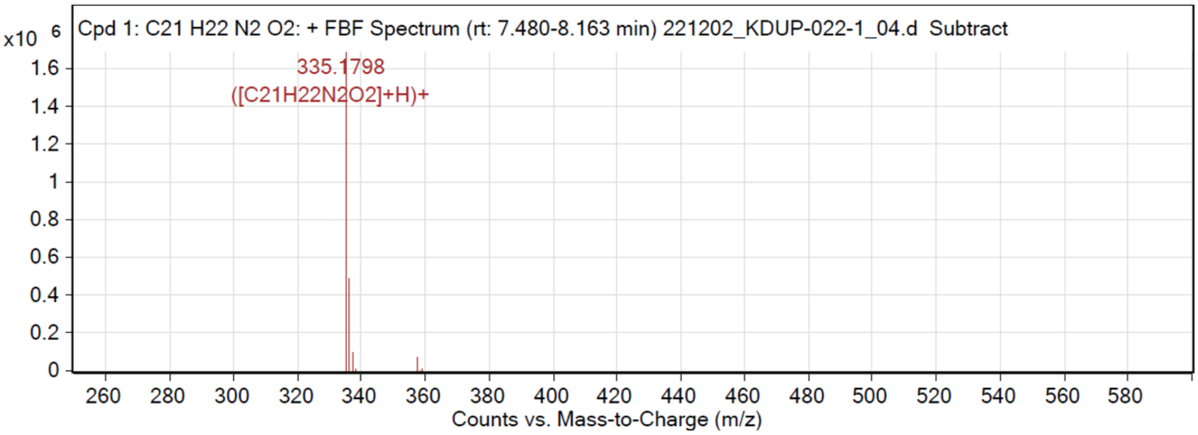

Figure S229. HRMS (ESI-TOF) spectrum of 2-benzyl-4-ethoxy-2,3,4,5-tetrahydro-1*H*-[1,4]diazepino[1,2-*a*]indol-1-one (**9b**).
